# Diversification of Ferredoxins across Living Organisms

**DOI:** 10.3390/cimb43030098

**Published:** 2021-09-30

**Authors:** Nomfundo Nzuza, Tiara Padayachee, Wanping Chen, Dominik Gront, David R. Nelson, Khajamohiddin Syed

**Affiliations:** 1 Department of Biochemistry and Microbiology, Faculty of Science and Agriculture, University of Zululand, KwaDlangezwa 3886, South Africa; nomfundonzuza11@gmail.com (N.N.); teez07padayachee@gmail.com (T.P.); 2 Department of Molecular Microbiology and Genetics, University of Göttingen, 37077 Göttingen, Germany; chenwanping1@foxmail.com; 3 Faculty of Chemistry, Biological and Chemical Research Center, University of Warsaw, Pasteura 1, 02-093 Warsaw, Poland; dgront@gmail.com; 4 Department of Microbiology, Immunology and Biochemistry, University of Tennessee Health Science Center, Memphis, TN 38163, USA; drnelson1@gmail.com

**Keywords:** Archaea, Bacteria, domains of life, Eukarya, evolution, ferredoxins, lateral gene transfer, iron-sulfur proteins

## Abstract

Ferredoxins, iron-sulfur (Fe-S) cluster proteins, play a key role in oxidoreduction reactions. To date, evolutionary analysis of these proteins across the domains of life have been confined to observing the abundance of Fe-S cluster types (2Fe-2S, 3Fe-4S, 4Fe-4S, 7Fe-8S (3Fe-4s and 4Fe-4S) and 2[4Fe-4S]) and the diversity of ferredoxins within these cluster types was not studied. To address this research gap, here we propose a subtype classification and nomenclature for ferredoxins based on the characteristic spacing between the cysteine amino acids of the Fe-S binding motif as a subtype signature to assess the diversity of ferredoxins across the living organisms. To test this hypothesis, comparative analysis of ferredoxins between bacterial groups, *Alphaproteobacteria* and *Firmicutes* and ferredoxins collected from species of different domains of life that are reported in the literature has been carried out. Ferredoxins were found to be highly diverse within their types. Large numbers of alphaproteobacterial species ferredoxin subtypes were found in *Firmicutes* species and the same ferredoxin subtypes across the species of Bacteria, Archaea, and Eukarya, suggesting shared common ancestral origin of ferredoxins between Archaea and Bacteria and lateral gene transfer of ferredoxins from prokaryotes (Archaea/Bacteria) to eukaryotes. This study opened new vistas for further analysis of diversity of ferredoxins in living organisms.

## 1. Introduction

Ferredoxins, iron-sulfur (Fe-S) cluster proteins, are ubiquitously present in all domains of life due to their involvement in fundamental metabolic processes such as photosynthesis, nitrogen fixation, and assimilation of hydrogen, nitrogen, and sulfur [1]. These proteins are primarily involved in the transfer of electrons in oxidation-reduction reactions [1]. Fe-S clusters are the simplest electron-transfer groups found in biological systems and are believed to have evolved early during chemical evolution [2,3]. Due to their rudimentary function and internal sequence symmetry, ferredoxins are considered to be a living protein fossil [4,5]. Based on the analysis of ferredoxin sequences, it has been proposed that all proteins evolved through tandem duplications of shorter proteins, which themselves may have emerged through the duplication of even shorter and simpler ancestral peptides [4,5,6].

The electron-transfer ability of ferredoxins to many proteins, including cytochrome P450 monooxygenases (CYPs/P450s) has been well established [7,8,9,10]. P450s are heme-thiolate proteins ubiquitously present in organisms across biological kingdoms [11]. With respect to ferredoxins binding partners, functional diversification and redundancy was observed. The ferredoxins from the hyperthermophilic Archaeon, *Thermococcus kodakarensis* [12], and the three model photosynthetic organisms: *Synechocystic* sp. PCC6803 [13], *Zea mays* [14], and *Chlamydomonas reinhardtii* [15] were found to have diverse binding partners despite being paralogs indicating their functional diversification. Interestingly, despite belongings to different Fe-S cluster types, ferredoxins in myxobacteria, *Sorangium cellulosum* So ce56 [16] and actinomycobacteria, *Mycobacterium tuberculosis* H37Rv is reported [17] to have complementary functions indicating their functional redundancy. Apart from their primary electron transfer function, ferredoxins are also reported to have regulatory functions [18,19]. 

Ever since the inception of the name “ferredoxin” in 1962 following the isolation of ferredoxin from *Clostridium pasteurianum* [20], to date, a large number of ferredoxin are reported in all domains of life [21]. The well-known and well-studied ferredoxins include human ferredoxin known as adrenodoxin [22,23], ferredoxin from *Pseudomonas putida* known as putidaredoxin [24] and ferredoxin 1 (FDX1) from plants [25]. Ferredoxins belonging to different Fe-S cluster types have been crystallized, and some progress in understanding their interactions with P450s has been reported [7,8,9,26]. 

Ferredoxins like other Fe-S cluster proteins are classified into different types based on the number of Fe-atoms in their cluster. To date, 2Fe-2S, 3Fe-4S, 4Fe-4S, 7Fe-8S (3Fe-4S and 4Fe-4S) and 2[4Fe-4S] cluster types are reported for ferredoxins [7]. Each of the Fe-S cluster types have their own characteristic Fe-S binding motif where Fe-atom binding cysteine amino acids reside. The characteristic Fe-S binding motif includes four cysteines for 2Fe-2S, three cysteines and a proline that follows after the third cysteine for 3Fe-4S, four cysteines and a proline that follows after the fourth cysteine for 4Fe-4S and the 7Fe-8S ferredoxins having both 3Fe-4S and 4Fe-4S clusters features. The 2[4Fe-4S] ferredoxins have two 4Fe-4S motifs but the spacing between Fe-atom binding cysteines are different compared to 4Fe-4S cluster type motif. The 2[4Fe-4S] proteins are divided into two subfamilies with small proteins (approximately 55 amino acids) having isopotential Fe-S clusters and larger proteins, known as Alvin (Alv) ferredoxins, having clusters with different potentials [27]. Sequence analysis revealed the presence of an extra cysteine in 2[4Fe-4S]Alv ferredoxins exactly after three amino acids following the last cysteine of the second 4Fe-4S binding cluster [27]. Based on the arrangement of cysteines in the Fe-S binding motif, 2Fe-2S cluster proteins are also classified as plant-type, mitochondrial-type, bacterial-type and thioredoxin-type [28,29]. 

It is believed that among Fe-S cluster types, 4Fe-4S clusters are the first to have evolved as the conditions that mimic the primitive earth resulted in the formation of 4Fe-4S clusters [2,30,31]. Studies indicated that the 4Fe-4S cluster is sensitive to oxygen compared to the 2Fe-2S cluster [32,33,34] and thus, it is predicted that after the Great Oxidation Event, organisms preferred 2F-2S clusters due their oxygen tolerance. This phenomenon was also observed in a study where comparative analysis of 2Fe-2S and 4Fe-4S cluster proteins and flavodoxin proteins across the domains of life was carried out, and it has been found that 4Fe-4S cluster type proteins are abundantly present in anaerobic organisms whereas 2Fe-2S cluster type proteins are abundant in aerobic organisms [21]. A study dating back to 1966 on observing the symmetrical origin of 2[4F-4S] ferredoxin from *C. pasteurianum* by Eck and Dayhoff led to the proposal that all proteins emerged through the duplication of even shorter and simpler ancestral peptides [5]. Recently, it has been shown that indeed 2[4Fe-4S] ferredoxins have drifted from their symmetric roots via gene duplication followed by mutations [35]. Numerous studies reporting the gene duplication events as the source to the growth and diversification of Fe-S proteins has been listed in a recent article [21]. 

Studies on the evolution of ferredoxins are rare, and from the available data, one can understand that ferredoxins originated from a common ancestor, but divergent evolution played a critical role in their diversity [3,36,37,38]. In addition to this, ferredoxins are believed to be evolved from different ancestral genes that are diversified from a common ancestor [3,6,36,37,38]. Only a handful of studies reported lateral (or horizontal) gene transfer (LGT) of ferredoxins [18,39,40]. The ferredoxin domain of cyanobacterial origin was found to be acquired by photosynthetic eukaryotes thorough HGT in chloroplast DnaJ-like proteins [18]. Eukaryotic protists such as *Giardia lamblia* and *Entamoeba histolytica* have ferredoxins predicted to be acquired from anaerobic bacteria by HGT [39]. Based on the percentage similarity, *Halobacterium salinarium* ferredoxin is predicted to be acquired by LGT from cyanobacterial species [40]. 

Apart from the above handful of studies that are confined to a small number of ferredoxins and a few species indicating LGT of ferredoxins, to date, diversity of ferredoxins within the cluster types was not studied. The current genome sequencing era has resulted in the availability of a large number of organisms’ genomes that have been sequenced. This gives us an opportunity to look into ferredoxin protein sequences and better understand their diversity across the domains of life. Thus, this study aimed to address this research gap by performing genome data mining, annotation and comparative analysis of ferredoxins between the ancient bacterial group *Alphaproteobacteria* and *Firmicutes* and ferredoxins from the species belonging to different domains of life that are reported in the literature. In order to understand the diversity of ferredoxins within Fe-S cluster types in organisms, we here propose a subtype classification of the Fe-S cluster types based on the characteristic spacing between the cysteine amino acids of Fe-S binding motif as a subtype signature, considering the fact that this motif is conserved in ferredoxins per se in Fe-S cluster proteins. Furthermore, we also propose a nomenclature system for easy identification of cluster type and subtype for a ferredoxin.

## 2. Methodology

### 2.1. Species and Database

In this study, 241 alphaproteobacterial species and 227 *Firmicutes* species genomes that are available for public use at the Kyoto Encyclopedia of Genes and Genomes (KEGG) database [41] were used (Table S1). These species are known to have P450s in their genome [42,43] and thus were selected for ferredoxins analysis considering P450s need ferredoxins for their function and so, as one can expect, there is the presence of a higher number of ferredoxins in these species. The list of species used in the study along with their species codes are presented in Table S1. 

### 2.2. Genome Data Mining and Annotation of Ferredoxins

Ferredoxins belonging to different Fe-S cluster types reported in the literature (Table 1) were used as reference proteins. Protein BLAST [44] was performed using these reference proteins against each of the bacterial species genomes at KEGG [41]. The hit proteins were manually checked for the presence of ferredoxin Fe-S cluster type canonical motif and the proteins that have the Fe-S binding motif were selected. The selected proteins were then subjected to protein BLAST [44] at the National Center for Biotechnology and Information (NCBI) [45] against the Protein Data Bank (PDB) database [46] and analyzed for the presence of characteristic motif of ferredoxins at Pfam database [47], InterPro database [48] and NCBI Conserved Domains Database (CDD) [49]. Proteins that had a hit against ferredoxins at PDB database and have ferredoxin motifs as indicated by different databases were selected for further analysis. The rationale for using the PDB database as the first priority to sort hit proteins is that the BLAST against this database not only helped in identifying ferredoxin proteins but also helped in accurately identifying Fe-atom binding cysteine amino acids based on the alignment of the hit protein sequence with the crystallized ferredoxin sequence. In addition to this, in some cases, BLAST against PDB database also helped to sort proteins accurately whether they were 3Fe-4S or 4Fe-4S cluster proteins and 7Fe-8S or 2[4Fe-4S] cluster proteins. Hit proteins that were found to be dehydrogenases, oxidases or reductases with Fe-S binding motif were not considered as ferredoxins as the Fe-S clusters in these proteins exceeded more than two and also, they belong to a different protein family. Furthermore, sequences that had hits to ferredoxins but not in full-length were also not considered for further analysis. We also noted that manual sorting of Fe-S cluster proteins into different types by consulting as many as possible databases such as PDB database [46], Pfam database [47], InterPro database [48] and NCBI CDD [49] is important for accurate assigning of Fe-S proteins to different types and also accurately identifying the Fe-atom binding cysteine amino acids. In this study, great care was taken to select only ferredoxins for further analysis.

### 2.3. Ferredoxins Subtype Classification and Nomenclature 

**Figure 1:**
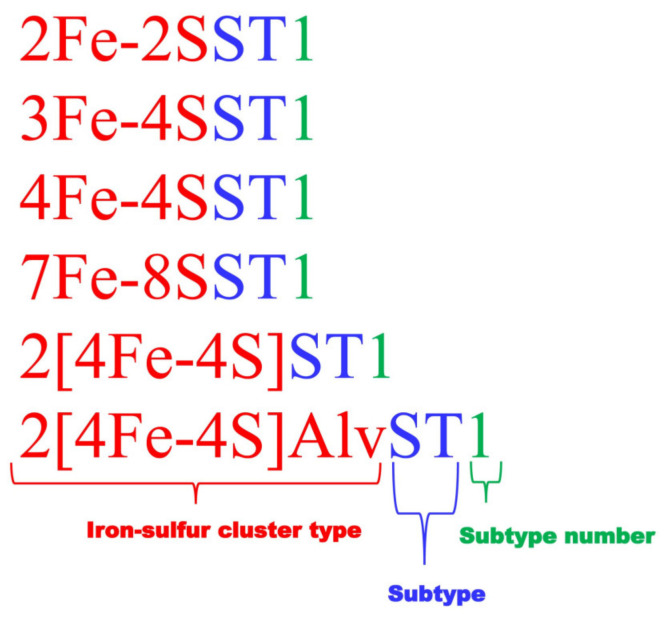
Ferredoxin nomenclature based on the spacing between the cysteine amino acids of the Fe-S cluster binding motif. Ferredoxins start with their Fe-S cluster type, followed by their ST indicating subtype and then the numeral indicating its subtype number in that type. Proteins grouped into different subtypes have the same characteristic spacing between the cysteine amino acids of the Fe-S cluster binding motif.

Ferredoxins selected under different Fe-S cluster types were then subjected to multiple sequence alignment (MSA) at Clustal Omega database [61]. Based on the MSA patterns proteins were separated into different groups to such an extent that there were no amino acid gaps between the cysteine amino acids of the Fe-S cluster binding motif. Ferredoxins that were not aligned with other proteins were then individually subjected to analysis of spacing between cysteine amino acids of the Fe-S cluster binding motif. Then the spacing patterns between cysteine amino acids of the Fe-S cluster binding motif were presented as the characteristic signature of a subtype. Analysis of ferredoxins revealed that proline amino acid is not always conserved in 2[4Fe-4S] ferredoxins and thus is not included in the Fe-S binding motif signature for this type ferredoxins. Although proline was included for 7Fe-8S cluster proteins, only cysteine residues and the amino acid spacing between these residues can be taken as a signature as non-conservation of proline will be expected when more ferredoxins are analyzed. In order to represent different subtypes in a type, a nomenclature system was developed such that each protein starts with its Fe-S cluster type followed by the abbreviation “ST” for “subtype” and then a numeral indicating its subtype number within a type (Figure 1). Subtype numbers were assigned following the occurrence of new ferredoxins in the study. Ferredoxins identified in the bacterial species of *Alphaproteobacteria* and *Firmicutes* and retrieved from the literature are presented along with their nomenclature in Table S2. 

### 2.4. Assigning Fe-S Cluster Subtypes to the Ferredoxins Retrieved from the Literature

Ferredoxins that are reported in the literature were collected by going through the published articles and also data mining at PDB database [46]. Most of the ferredoxins belonging to the species of Archaea and some Eukarya were retrieved from an article published by Campbell and co-workers [21]. A search of the PDB database was carried out using Fe-S cluster type name, and the ferredoxin sequences were collected after manually looking at the Fe-S clusters of the ferredoxin. These ferredoxins were then subjected to subtype nomenclature as described in the Section 2.3. Ferredoxins were retrieved from literature and the data mined from the PDB database were presented along with their nomenclature in Table S3. 

### 2.5. Phylogenetic Analysis of Ferredoxins

Phylogenetic analysis of ferredoxins was carried out following the procedure described recently by our laboratory [43,62]. The phylogenetic tree of ferredoxins was constructed using protein sequences. Firstly, the MAFFT v6.864 [63] was used to align the protein sequences that are part of the Trex web server [64]. The alignments were then used to interpret the best tree by the Trex web server [64]. Lastly, a web-based tool, VisuaLife, was used to create, visualize, and color the tree [65].

### 2.6. Generation of Ferredoxin Subtype Profile Heat Maps

Ferredoxin subtype profile heat maps were generated following the procedure described recently by our laboratory [43,62]. The heat map was generated using the ferredoxin subtype data to show the presence or absence of ferredoxin subtypes in three domains of life, Archaea, Bacteria, and Eukarya. The data were represented as (−3) for subtype absence (green) and (3) for subtype presence (red). A tab-delimited file was imported into Mev (Multi-experiment viewer) [66]. Hierarchical clustering using a Euclidean distance metric was used to cluster the data. Ferredoxin subtypes formed the vertical axis and three domains of life formed the horizontal axis.

## 3. Results and Discussion

### 3.1. Alphaproteobacterial and *Firmicutes* Species Have Different Fe-S Cluster Type Ferredoxins in Their Genomes

**Figure 2:**
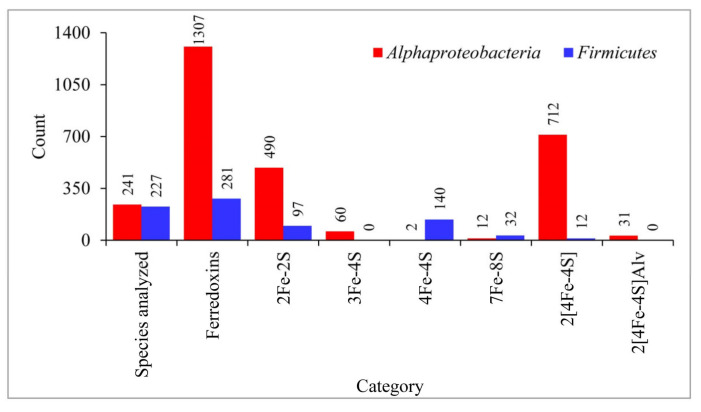
Comparative analysis of ferredoxins iron-sulfur (Fe-S) cluster features between alphaproteobacterial species and *Firmicutes* species. The number next to the bar indicates the count for that category. Detailed information on species and their ferredoxins is presented in Table S1.

Genome data mining of 241 alphaproteobacterial species and 227 *Firmicutes* species revealed presence of 1307 and 281 ferredoxins in their genomes (Figure 2 and Table S1). This suggests that alphaproteobacterial species have four and a half times more ferredoxins in their genomes compared to *Firmicutes* species (Figure 2). Among alphaproteobacterial species *Sphingomonas wittichii* have the highest number of twelve ferredoxins (Table S1) and in *Firmicutes* species *Kyrpidia spormannii* have the highest number of six ferredoxins (Table S1). Six different Fe-S cluster type ferredoxins were found in alphaproteobacterial species whereas only four Fe-S cluster type ferredoxins were found in *Firmicutes* species (Figure 2). The 3Fe-4S and 2[4Fe-4S]Alv cluster type ferredoxins were not identified in the *Firmicutes* species analyzed in this study. 

Analysis of ferredoxin Fe-S cluster types in alphaproteobacterial species revealed that 2[4Fe-4S] cluster type ferredoxins was found to be most abundant with 712 ferredoxins followed by 2Fe-2S with 490 ferredoxins, 3Fe-4S with 60 ferredoxins, 2[4Fe-4S]Alv with 31 ferredoxins), 7Fe-8S with 12 ferredoxins and 4Fe-4S cluster with two ferredoxins (Figure 2). Of the four Fe-S cluster types found in *Firmicutes* species, the 4Fe-4S was the most abundant with 140 ferredoxins followed by 2Fe-2S with 97 ferredoxins, 7Fe-8S with 32 ferredoxins and 2[4Fe-4S] with 12 ferredoxins (Figure 2). Comparison of ferredoxin Fe-S cluster types between these two bacterial groups revealed that alphaproteobacterial species have more ferredoxins belonging to the clusters 2[4Fe-4S] and 2Fe-2S whereas *Firmicutes* species have more ferredoxins belonging to the clusters 4Fe-4S and 7Fe-8S (Figure 2). This suggests that these two bacterial groups have different preferences with respect to Fe-S cluster type. A point to be noted is that the results of this study regarding the presence of more 4Fe-4S cluster type ferredoxins in *Firmicutes* species corroborate with a previous study where only 2Fe-2S and 4Fe-4S cluster types were analyzed as part of global protein electron carrier proteins analysis across the domain of life [21].

### 3.2. Highly Diverse and Common Ancestral Origin of Ferredoxins between Alphaproteobacteria and *Firmicutes*

In this study, we tested the amino acid spacing pattern between the cysteine amino acids of Fe-S cluster binding motif as a signature for ferredoxins subtype classification, which was therefore developed to understand diversity of ferredoxins using alphaproteobacterial species and *Firmicutes* species as model organisms. This took into consideration the fact that alphaproteobacterial species are ancient species compared to *Firmicutes* species [67] and thus they serve as the best models for understanding the diversity of ferredoxins within Fe-S cluster types. Furthermore, ferredoxins that are common to these species will be a good indication of their common ancestral origin. Analysis of ferredoxin Fe-S cluster subtypes revealed that alphaproteobacterial species and *Firmicutes* species have highly diverse ferredoxins in their genomes and some ferredoxin were found to share a common ancestor as ferredoxin belonging to the same subtype were found in both groups. This suggests that the subtype classification is helpful in understanding the diversity of ferredoxins within a cluster type. A detailed analysis on ferredoxin Fe-S cluster subtypes between these two bacterial groups is presented below: 

#### 3.2.1. 2Fe-2S

Based on the amino acid spacing pattern between the cysteine amino acids of the Fe-S cluster binding motif, 490 2Fe-2S ferredoxins of alphaproteobacterial species can be grouped into 29 subtypes, whereas 97 2Fe-2S ferredoxins of *Firmicutes* species can be grouped into 11 subtypes (Table 2). Among alphaproteobacterial 2Fe-2S ferredoxin subtypes, subtype 1 has the most ferredoxins followed by subtype 2 (Table 2), indicating subtype 1 ferredoxins are highly preferred by these species. *Firmicutes* species have the highest number of subtype 20 ferredoxins in their genomes followed by subtype 18 (Table 2). Comparative analysis of subtypes revealed that eight subtypes were found to be common between the alphaproteobacterial species and the *Firmicutes* species (Table 2), suggesting the common ancestral origin of these eight subtype ferredoxins.

#### 3.2.2. 3Fe-4S

Sixty 3Fe-4S ferredoxins found in alphaproteobacterial species can be grouped into five different subtypes (Table 2). Among the five subtypes, subtype 1 ferredoxins present in higher numbers followed by subtypes 2 and 3 (Table 2), indicating ferredoxins with subtype 1 are preferred by alphaproteobacterial species. Interestingly, 3Fe-4S ferredoxin were not found in *Firmicutes* species analysed in this study. 

#### 3.2.3. 4Fe-4S

The two 4Fe-4S ferredoxins found in alphaproteobacterial species can be grouped into the same subtype 1 and 140 4Fe-4S ferredoxins found in *Firmicutes* species can be grouped into five different subtypes (Table 2). Of the five subtypes, subtype 2 ferredoxins were found in higher numbers in *Firmicutes* species indicating their preference for these species (Table 2). Contrary to the 2Fe-2S ferredoxin subtypes, no common 4Fe-4S subtypes were found between alphaproteobacterial species and *Firmicutes* species (Table 2), indicating 4Fe-4S ferredoxins are highly diverse between these two bacterial groups. 

#### 3.2.4. 7Fe-8S

Twelve 7Fe-8S ferredoxins found in alphaproteobacterial species grouped into four different subtypes, whereas all the 32 *Firmicutes* species ferredoxins belongs to the subtype 1 (Table 2). Subtype 1 ferredoxins were found to be common between these two bacterial groups (Table 2) indicating not only a common ancestral origin of these ferredoxin subtypes but also, they are wide-spread in *Firmicutes* species. 

#### 3.2.5. 2[4Fe-4S]

Seven hundred and eleven 2[4Fe-4S] ferredoxins found in alphaproteobacterial species can be grouped into 16 subtypes whereas the 12 2[4Fe-4S] ferredoxins of *Firmicutes* species can be grouped into three different subtypes (Table 2). Subtype 1 ferredoxins are more abundant in alphaproteobacterial species followed by subtypes 2 and 3 (Table 2). All the three ferredoxin subtypes of *Firmicutes* species can be found in alphaproteobacterial species (Table 2) indicating a common ancestral origin of these ferredoxin subtypes from alphaproteobacterial species to *Firmicutes* species. 

#### 3.2.6. 2[4Fe-4S]Alv

2[4Fe-4S]Alv ferredoxin was identified only in alphaproteobacterial species. The 31 2[4Fe-4S]Alv ferredoxins of alphaproteobacterial species were grouped into eight different subtypes (Table 2). Of the eight subtypes, subtypes 1 and 2 have more ferredoxins than the rest of the subtypes (Table 2). 

### 3.3. Ferredoxin Fe-S Cluster Types Canonical Motifs 

Based on the ferredoxins analyzed in the study and their Fe-S binding motif patterns, three different canonical motifs can be deduced for 2Fe-2S ferredoxins, CX_3–5_CX_1–2_CX_22–82_C, CX_2–12_CX_30–44_CX_3_C and CX_4–7_CX_29–35_C. Among the three canonical motifs, the majority of the ferredoxin subtypes fall under the canonical motif CX_3–5_CX_1–2_CX_22–82_C followed by CX_2–12_CX_30–44_CX_3_C and CX_4–7_CX_29–35_C (Table 2). This suggests that CX_3–5_CX_1–2_CX_22–82_C canonical motif for 2Fe-2S ferredoxins is highly prevalent in organisms. Contrary to 2Fe-2S ferredoxins, only one Fe-S binding canonical motif was observed for the rest of the ferredoxin Fe-S cluster types. CX_5_CX_35–49_CP for 3Fe-4S; CX_2–5_CX_2–3_CX_30–45_CP for 4Fe-4S; CX_3–10_CX_3_CPX_17–40_CX_2_CX_2_CX_3_CP for 7Fe-8S; CX_2–7_CX_2–4_CX_2–3_CX_14–42_CX_1–2_CX_2–8_CX_3_C for 2[4Fe-4S] and CX_2_CX_2_CX_3_CX_18–46_CX_2_CX_2–8_CX_3_CX_3_C for 2[4Fe-4S]Alv. Analysis of Fe-S cluster binding motif amino acid patterns revealed that in all ferredoxins subtypes, the cysteine amino acid is invariantly conserved and the proline amino acid is not always conserved in 2[4Fe-4S] ferredoxins (Table 2). However, proline is invariantly conserved in 3Fe-4S and 4Fe-4S ferredoxins following the final cysteine amino acid of the Fe-S cluster binding motif, and this conservation makes these ferredoxins distinct compared to 2Fe-2S ferredoxins (Table 2). In addition to this, the amino acid spacing between the first and second cysteine amino acid is five in the Fe-S cluster binding motif in 3Fe-4S ferredoxins compared to 2Fe-2S ferredoxins (Table 2). With the exception of *Mycobacterium tuberculosis* H37Rv ferredoxin (Rv2007c), all the 7Fe-8S ferredoxins analyzed in this study have proline after the final cysteine amino acid in their Fe-S cluster binding motif. An interesting observation is that some subtypes have the same number of amino acids in the Fe-S cluster binding motif but the amino acid patterns especially the positional arrangement of cysteine amino acids-are different, indicating the amino acid patterns of Fe-S cluster binding motif is indeed a characteristic signature for a ferredoxin subtype (Table 2).

### 3.4. Evolutionary Linkage of Ferredoxins Subtype Classification

**Figure 3:**
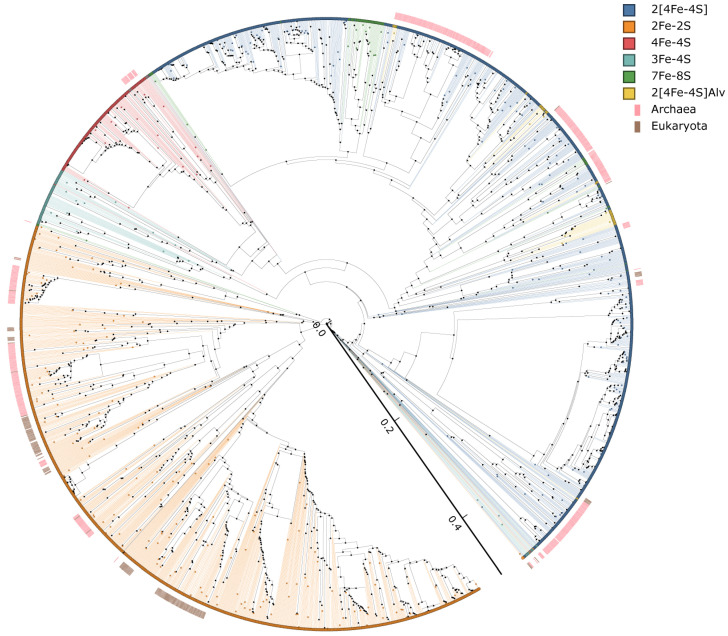
Phylogenetic analysis of ferredoxins. Ferredoxins belonging to different Fe-S cluster types were highlighted in different colors and indicated in the figure. Archaea and Eukaryota ferredoxin sequences were marked with pink and brown stripe, respectively. All other sequences originate from Bacteria. Ferredoxin protein sequences used to construct the phylogenetic tree are presented in Tables S2 and S3.

The subtype classification of ferredoxins proposed in this study is solely based on the arrangement of cysteine amino acids in the Fe-S cluster binding motif and to use this subtype classification as a criterion for assessing the diversity of ferredoxins one should evaluate the evolutionary linkage of subtypes, if any. Thus, here we performed evolutionary analysis of ferredoxins (Figure 3). As shown in Figure 3, with the exception of some ferredoxins, most of the ferredoxin were grouped as per their types and also as per their subtypes suggesting our subtype classification criteria certainly followed the evolutionary trend. This indicates that the amino acid spacing pattern between the cysteine amino acids of Fe-S cluster binding motifs is evolutionarily conserved and passed across the species. One interesting aspect that can be drawn from the evolutionary analysis is that although 4Fe-4S ferredoxin aligned independently on the tree, their placement with 2[4Fe-4S] suggests the domains of 4Fe-4S ferredoxins certainly passed to 2[4Fe-4S] ferredoxins. This observation also strongly supports the hypothesis proposed by Eck and Dayhoff that all proteins emerged through the duplication of even shorter and simpler ancestral peptides [5]. Some of the ferredoxin Fe-S cluster types branched on the tree and aligned with other Fe-S cluster types, indicating they are evolutionarily linked to each other by sharing high sequence similarity in certain parts of the Fe-S cluster domain. 

### 3.5. LGT of Ferredoxins

The current genomic era resulted in genome sequencing of large numbers of organisms and thus the availability of ferredoxin sequences from all domains of life. This has given us an opportunity to look into the LGT of ferredoxins. The subtype classification of ferredoxins as proposed in this study will certainly help in easily understanding the LGT of ferredoxins in domains of life as one can assume the ferredoxins belonging to the same subtype are indeed evolutionarily linked (as discussed in the above section). In order to understand LGT of ferredoxins across the living organisms, we have collected and annotated 538, 95 and 171 ferredoxins from Archaea, Bacteria (excluding ferredoxins from *Alphaproteobacteria*/*Firmicutes*) and Eukarya, respectively (Table 2 and Table S3). 

**Figure 4:**
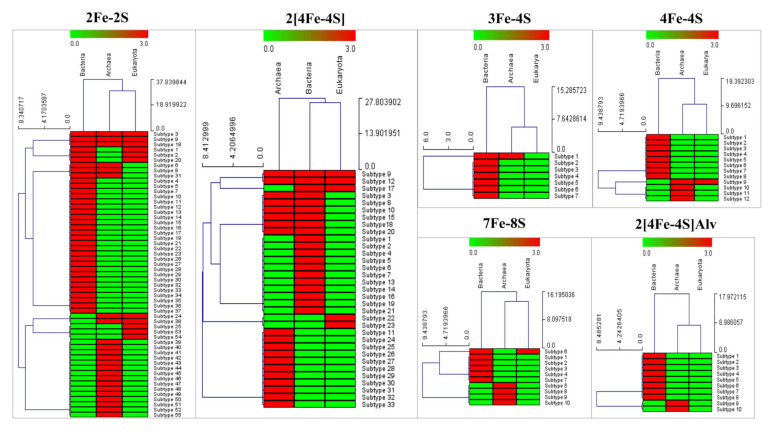
Heat map figure representing the presence (red) and absence (green) of ferredoxin subtypes in Archaea, Bacteria and Eukarya. Ferredoxin subtypes form the vertical axis and domains of life forms the horizontal axis.

Comparative analysis of ferredoxin subtypes revealed LGT of ferredoxins across the domains of life (Figure 4). Subtypes 3, 9 and 18 in 2Fe-2S and subtypes 9 and 12 in 2[4Fe-4S] were found to be present across Archaea, Bacteria and Eukarya, indicating their LGT from Archaea/Bacteria to Eukarya (Figure 4). LGT of 2Fe-2S subtypes 24 and 38 between Archaea and Eukarya was observed (Figure 4). LGT of 4Fe-4S subtype 9 between Archaea and Eukarya, 7Fe-8S subtype 6 between Bacteria and Eukarya was observed (Figure 4). LGT of 2[4Fe-FS] subtype 17 between Bacteria and Eukarya was also observed (Figure 4). The above ferredoxin subtypes were found to be aligned together on phylogenetic tree (Figure 3) strongly indicating the LGT of these ferredoxins from prokaryotes (Archaea/Bacteria) to eukaryotes. 

This strongly suggests that ferredoxins from prokaryotes certainly passed to eukaryotes and subtype classification based on cysteine spacing signature motif amino acid patterns can be used to understand the LGT and the diversity of ferredoxins within a Fe-S cluster type.

## 4. Conclusions

Fe-S cluster proteins such as ferredoxins are the simplest known electron transfer proteins in biology. They evolved in such a way that they are capable of tuning both reduction potential and partner binding ability [21,35,68,69]. Due to the diversity in the primary amino acid sequences, to date, classification of ferredoxins was limited to the Fe-S cluster types. This led to the observation of different ferredoxin cluster types across the living organisms, but how these proteins evolved within these clusters and their distribution patterns across the living organisms were rarely reported. In this study we proposed ferredoxins subtype classification based on the amino acid spacing pattern between the cysteine amino acids of the Fe-S cluster binding motif as a subtype signature to understand the diversity and lateral (or horizontal) gene transfer of ferredoxins across the domains of life. We tested this hypothesis and found that subtype classification indeed serves as an effective tool to understand the diversity (based on the presence of number of subtypes within a Fe-S cluster type) and evolution of ferredoxins as the presence of the same ferredoxin subtypes across the domains of life was identified. For easy identification of ferredoxins belonging to different subtypes, a nomenclature system was also developed. Work is in progress on comprehensive analysis of ferredoxins from other bacterial, archaeal and eukaryotic species for better understanding the evolution of ferredoxins across the domains of life and also to deduce the definitive characteristic signatures for ferredoxin subtypes.

## Figures and Tables

**Table 1:** Information on ferredoxins that are used as reference proteins for datamining of ferredoxins in the bacterial species of *Alphaproteobacteria* and *Firmicutes*.

2Fe-2S.		
GenBank Accession Number or PDB Code	Species Name	Reference
NP_004100.1 (Adrenodoxin)	*Homo sapiens*	[22]
1PDX (Putidaredoxin)	*Pseudomonas putida*	[24]
ABB56370.1	*Synechococcus elongatus* PCC 7942 = FACHB-805	[50]
ABB56928.1	*Synechococcus elongatus* PCC 7942 = FACHB-805	[50]
WP_013424358.1 (FraEuI1c_3227)	*Frankia* sp. EuI1c (*Frankia inefficax* sp.)	[51]
WP_012394830.1 (mmi:MMAR_3155)	*Mycobacterium marinum*	[52]
3Fe-4S/4Fe-4S		
CAB59502.1	*Streptomyces coelicolor* A3(2)	[53]
WP_013425251.1 (FraEuI1c_4132)	*Frankia* sp. EuI1c (*Frankia inefficax* sp.)	[51]
WP_013426476.1 (FraEuI1c_5370)	*Frankia* sp. EuI1c (*Frankia inefficax* sp.)	[51]
WP_011740769.1 (Mmar_2879)	*Mycobacterium marinum*	[52]
WP_012395565.1 (Mmar_3973)	*Mycobacterium marinum*	[52]
WP_012396301.1 (Mmar_4763)	*Mycobacterium marinum*	[52]
NP_215277.1 (FdX-Rv0763c)	*Mycobacterium tuberculosis* H37Rv	[17,54]
NP_216302.1 (FdxE-Rv1786)	*Mycobacterium tuberculosis* H37Rv	[17,54]
ABB57779.1	*Synechococcus elongatus* PCC 7942 = FACHB-805	[50]
7Fe-8S		
NP_215693 (FdxC-Rv1177),	*Mycobacterium tuberculosis* H37Rv	[17]
NP_216523.1 (FdxA-Rv2007c)	*Mycobacterium tuberculosis* H37Rv	[17]
2VKR	*Acidianus ambivalens*	[55]
1H98	*Thermus thermophilus*	[56]
ABB56846.1	*Synechococcus elongatus* PCC 7942 = FACHB-805	[50]
2[4Fe-4S]		
2ZVS	*Escherichia coli* K-12	[27]
2FDN	*Clostridium acidurici*	[57]
WP_013068980.1 (FDI)	*Rhodobacter capsulatus*	[58]
2[4Fe-4S]Alv		
1BLU	*Allochromatium vinosum*	[59]
2FGO	*Pseudomonas aeruginosa*	[57]
WP_023923722.1 (FDIII)	*Rhodobacter capsulatus*	[58]
1RGV	*Thauera aromatica* K172	[60]

Note: For easy identification purpose for some ferredoxins their most popular literary names were included in parenthesis right after their GenBank accession number or PDB code.

**Table 2:** Subtype classification of ferredoxin across the domains of life. Ferredoxins collected from species belonging to different biological kingdoms that are reported in the literature and data mined at Protein Data Bank (PDB) also presented in the table as per their subtypes under the domains: Archaea, Bacteria, and Eukarya. Ferredoxin protein sequences as per their nomenclature are presented in Tables S2 and S3.

Subtypes	Cysteine Spacing Signature	No. AA	No of Ferredoxins				
			*Alphaproteobacteria*	*Firmicutes*	From Literature		
					Archaea	Bacteria	Eukarya
2Fe-2S							
Subtype 1	CX_5_CX_2_CX_36_C	47	296			2	64
Subtype 2	CX_5_CX_2_CX_37_C	48	81	1		4	22
Subtype 3	CX_4_CX_2_CX_29_C	39	11	1	7	11	55
Subtype 4	CX_5_CX_2_CX_35_C	46	12	3		2	
Subtype 5	CX_5_CX_2_CX_38_C	49	12	1			
Subtype 6	CX_4_CX_2_CX_34_C	44	11	11	7	1	
Subtype 7	CX_4_CX_2_CX_51_C	61	8			1	
Subtype 8	CX_4_CX_2_CX_31_C	41	7	6	1	1	
Subtype 9	CX_4_CX_2_CX_33_C	43	2	3	19		2
Subtype 10	CX_5_CX_2_CX_39_C	50	2			1	
Subtype 11	CX_4_CX_2_CX_35_C	46	1				
Subtype 12	CX_4_CX_2_CX_25_C	35	1				
Subtype 13	CX_7_CX_34_CX_3_C	48	1				
Subtype 14	CX_4_CX_29_C	36	1				
Subtype 15	CX_7_CX_29_C	39	1				
Subtype 16	CX_7_CX_35_C	45	1				
Subtype 17	CX_5_CX_2_CX_33_C	44	1				
Subtype 18	CX_5_CX_2_CX_34_C	45	2	20	2	1	1
Subtype 19	CX_5_CX_2_CX_35_C	46	1				
Subtype 20	CX_5_CX_2_CX_32_C	43		44		1	5
Subtype 21	CX_4_CX_31_CX_3_C	42		5			
Subtype 22	CX_2_CX_41_CX_3_C	50		2			
Subtype 23	CX_7_CX_38_CX_3_C	52				1	
Subtype 24	CX_4_CX_2_CX_30_C	40			70		1
Subtype 25	CX_5_CX_2_CX_30_C	41					1
Subtype 26	CX_12_CX_30_CX_3_C	49	21				
Subtype 27	CX_12_CX_31_CX_3_C	50	4				
Subtype 28	CX_8_CX_44_CX_3_C	59	3				
Subtype 29	CX_8_CX_33_CX_3_C	48	3				
Subtype 30	CX_8_CX_32_CX_3_C	47	2				
Subtype 31	CX_4_CX_36_CX_3_C	47	1		1		
Subtype 32	CX_8_CX_38_CX_3_C	53	1				
Subtype 33	CX_8_CX_39_CX_3_C	54	1				
Subtype 34	CX_9_CX_33_CX_3_C	49	1				
Subtype 35	CX_12_CX_33_CX_3_C	52	1				
Subtype 36	CX_3_CX_1_CX_38_C	46				1	
Subtype 37	CX_12_CX_32_CX_3_C	51				1	
Subtype 38	CX_4_CX_2_CX_28_C	38			40		3
Subtype 39	CX_4_CX_2_CX_46_C	56			2		
Subtype 40	CX_4_CX_2_CX_49_C	59			3		
Subtype 41	CX_4_CX_2_CX_45_C	55			2		
Subtype 42	CX_4_CX_2_CX_65_C	75			1		
Subtype 43	CX_4_CX_2_CX_50_C	60			2		
Subtype 44	CX_4_CX_2_CX_47_C	57			2		
Subtype 45	CX_4_CX_2_CX_48_C	58			1		
Subtype 46	CX_5_CX_2_CX_52_C	63			2		
Subtype 47	CX_5_CX_2_CX_31_C	42			1		
Subtype 48	CX_5_CX_2_CX_28_C	39			2		
Subtype 49	CX_5_CX_2_CX_27_C	38			10		
Subtype 50	CX_5_CX_2_CX_82_C	93			2		
Subtype 51	CX_5_CX_2_CX_29_C	40			1		
Subtype 52	CX_4_CX_2_CX_32_C	42			1		
Subtype 53	CX_5_CX_2_CX_42_C	53					1
Subtype 54	CX_4_CX_2_CX_22_C	32					2
Subtype 55	CX_4_CX_2_CX_29_C	39			2		
3Fe-4S							
Subtype 1	CX_5_CX_38_CP	47	26		2	7	
Subtype 2	CX_5_CX_37_CP	46	16			13	
Subtype 3	CX_5_CX_36_CP	45	14			2	
Subtype 4	CX_5_CX_40_CP	49	3				
Subtype 5	CX_5_CX_36_CP	45	1				
Subtype 6	CX_5_CX_35_CP	44				5	
Subtype 7	CX_5_CX_49_CP	58				2	
4Fe-4S							
Subtype 1	CX_5_CX_3_CX_33_CP	46	2				
Subtype 2	CX_2_CX_2_CX_43_CP	52		107			
Subtype 3	CX_2_CX_2_CX_45_CP	54		24		1	
Subtype 4	CX_2_CX_2_CX_37_CP	46		6			
Subtype 5	CX_2_CX_2_CX_44_CP	53		2			
Subtype 6	CX_2_CX_2_CX_39_CP	48		1			
Subtype 7	CX_2_CX_2_CX_36_CP	45				2	
Subtype 8	CX_2_CX_2_CX_34_CP	43				1	
Subtype 9	CX_2_CX_2_CX_38_CP	47			1		1
Subtype 10	CX_5_CX_3_CX_32_CP	45			1		
Subtype 11	CX_5_CX_3_CX_30_CP	43			12		
Subtype 12	CX_5_CX_3_CX_31_CP	44			2		
7Fe-8S							
Subtype 1	CX_7_CX_3_CPX_17_CX_2_CX_2_CX_3_CP ^*^	43	6	32		13	
Subtype 2	CX_5_CX_3_CPX_40_CX_2_CX_2_CX_3_CP	64	4				
Subtype 3	CX_5_CX_3_CPX_20_CX_2_CX_2_CX_3_CP	44	1				
Subtype 4	CX_10_CX_3_CPX_22_CX_2_CX_2_CX_3_CP	51	1				
Subtype 5	CX_5_CX_3_CPX_26_CX_2_CX_2_CX_3_CP	50			1		
Subtype 6	CX_5_CX_3_CPX_24_CX_2_CX_2_CX_3_CP	48				1	1
Subtype 7	CX_10_CX_3_CPX_17_CX_2_CX_2_CX_3_CP	46				1	
Subtype 8	CX_5_CX_3_CPX_22_CX_2_CX_2_CX_3_CP	46			9		
Subtype 9	CX_5_CX_3_CPX_18_CX_2_CX_2_CX_3_C	42			1		
Subtype 10	CX_3_CX_3_CPX_22_CX_2_CX_2_CX_3_CP	44			2		
2[4Fe-4S]							
Subtype 1	CX_2_CX_4_CX_3_CX_18_CX_2_CX_2_CX_3_C	42	267				
Subtype 2	CX_2_CX_2_CX_3_CX_18_CX_2_CX_8_CX_3_C	46	90			4	
Subtype 3	CX_2_CX_2_CX_3_CX_20_CX_2_CX_2_CX_3_C	42	33	2	52	1	
Subtype 4	CX_7_CX_2_CX_3_CX_23_CX_2_CX_2_CX_3_C	50	5				
Subtype 5	CX_2_CX_2_CX_3_CX_42_CX_2_CX_2_CX_3_C	64	3				
Subtype 6	CX_2_CX_2_CX_3_CX_18_CX_2_CX_7_CX_3_C	45	2				
Subtype 7	CX_2_CX_2_CX_3_CX_18_CX_2_CX_6_CX_3_C	44	2				
Subtype 8	CX_2_CX_2_CX_3_CX_24_CX_2_CX_2_CX_3_C	46	2		2		
Subtype 9	CX_2_CX_2_CX_3_CX_18_CX_2_CX_2_CX_3_C	40	1	6	78	3	1
Subtype 10	CX_2_CX_2_CX_3_CX_21_CX_2_CX_2_CX_3_C	43	6		2		
Subtype 11	CX_2_CX_2_CX_3_CX_18_CX_3_CX_2_CX_3_C	42			2		
Subtype 12	CX_2_CX_2_CX_3_CX_28_CX_2_CX_2_CX_3_C	50	132		1		2
Subtype 13	CX_2_CX_2_CX_3_CX_27_CX_2_CX_2_CX_3_C	49	2				
Subtype 14	CX_5_CX_2_CX_3_CX_20_CX_2_CX_2_CX_3_C	45	21				
Subtype 15	CX_2_CX_2_CX_3_CX_19_CX_2_CX_2_CX_3_C	41	1	4	58		
Subtype 16	CX_2_CX_2_CX_3_CX_40_CX_2_CX_2_CX_3_C	50	1				
Subtype 17	CX_2_CX_2_CX_3_CX_29_CX_2_CX_2_CX_3_C	51	144				2
Subtype18	CX_4_CX_2_CX_3_CX_18_CX_2_CX_2_CX_3_C	42			1	1	
Subtype 19	CX_3_CX_2_CX_3_CX_20_CX_2_CX_2_CX_3_C	43				2	
Subtype 20	CX_2_CX_2_CX_3_CX_17_CX_2_CX_2_CX_3_C	39			24	1	
Subtype 21	CX_3_CX_3_CX_3_CX_37_CX_1_CX_3_CX_3_C	61				1	
Subtype 22	CX_2_CX_2_CX_3_CX_26_CX_2_CX_2_CX_3_C	48					6
Subtype 23	CX_2_CX_2_CX_3_CX_30_CX_2_CX_2_CX_3_C	52					1
Subtype 24	CX_2_CX_2_CX_3_CX_33_CX_2_CX_2_CX_3_C	55			22		
Subtype 25	CX_2_CX_2_CX_3_CX_32_CX_2_CX_2_CX_3_C	54			23		
Subtype 26	CX_2_CX_2_CX_3_CX_23_CX_2_CX_2_CX_3_C	45			2		
Subtype 27	CX_2_CX_2_CX_3_CX_34_CX_2_CX_2_CX_3_C	56			1		
Subtype 28	CX_2_CX_2_CX_3_CX_14_CX_2_CX_2_CX_3_C	36			2		
Subtype 29	CX_2_CX_2_CX_3_CX_22_CX_2_CX_2_CX_3_C	44			2		
Subtype 30	CX_2_CX_2_CX_2_CX_38_CX_2_CX_2_CX_3_C	59			1		
Subtype 31	CX_4_CX_2_CX_3_CX_19_CX_2_CX_2_CX_3_C	43			24		
Subtype 32	CX_5_CX_2_CX_3_CX_19_CX_2_CX_2_CX_3_C	44			3		
Subtype 33	CX_2_CX_2_CX_3_CX_16_CX_2_CX_2_CX_3_C	38			16		
2[4Fe-4S]Alv							
Subtype 1	CX_2_CX_2_CX_3_CX_18_CX_2_CX_8_CX_3_CX_3_C	50	10			5	
Subtype 2	CX_2_CX_2_CX_3_CX_39_CX_2_CX_2_CX_3_CX_3_C	65	9				
Subtype 3	CX_2_CX_2_CX_3_CX_43_CX_2_CX_2_CX_3_CX_3_C	69	5			1	
Subtype 4	CX_2_CX_2_CX_3_CX_42_CX_2_CX_2_CX_3_CX_3_C	68	1				
Subtype 5	CX_2_CX_2_CX_3_CX_40_CX_2_CX_2_CX_3_CX_3_C	66	2				
Subtype 6	CX_2_CX_2_CX_3_CX_38_CX_2_CX_2_CX_3_CX_3_C	64	1				
Subtype 7	CX_2_CX_2_CX_3_CX_46_CX_2_CX_2_CX_3_CX_3_C	72	2				
Subtype 8	CX_2_CX_2_CX_3_CX_44_CX_2_CX_2_CX_3_CX_3_C	70	1				
Subtype 9	CX_2_CX_2_CX_3_CX_30_CX_2_CX_2_CX_3_CX_3_C	56			2		
Subtype 10	CX_2_CX_2_CX_3_CX_19_CX_2_CX_2_CX_3_CX_3_C	45			8		

Note: *, Only 7Fe-8S ferredoxins from *M. tuberculosis* H37Rv (Rv2007c) were found to have “arginine (R)” instead of “proline (P)”. Proline is not conserved in 2[4Fe-4S] cluster ferredoxins and thus not included in the signature. Although proline was included for 7Fe-8S cluster ferredoxins, only cysteine residues and the amino acid spacing between these residues can be taken as a signature. No AA indicates number of amino acids in cysteine spacing signature motif.

## Data Availability

Not applicable.

## Supplementary Materials

The following are available online at https://www.mdpi.com/article/10.3390/cimb43030098/s1, Table S1: Comparative analysis of ferredoxins in the bacterial groups *Alphaproteobacteria* and *Firmicutes*. Table S2: Ferredoxin protein sequences identified in 241 alphaproteobacterial species and 227 *Firmicutes* species. Ferredoxins were sorted as per their Fe-S cluster type and subtypes. Name of the ferredoxins were followed as mentioned in methodology. The name of the ferredoxins includes their nomenclature name followed by protein ID from KEGG database and species name, Table S3: Ferredoxin protein sequences identified by data mining of published articles and Protein Data Bank (PDB). Ferredoxins were sorted as per their Fe-S cluster type and subtypes. The name of the ferredoxins includes their nomenclature name as described in the methodology followed by protein ID (either GenBank accession number or PDB ID) database and species name. Cysteine amino acids that bind to the Fe-atom of Fe-S cluster is highlighted in the sequences. In case of two Fe-S clusters, cysteine amino acids highlighted with different colors representing their binding to different Fe-S clusters.
