# Supplementary material for: Diversification of Ferredoxins across Living Organisms
Source: Curr Issues Mol Biol. 2021 Sep 30;43(3):1374–90. doi: 10.3390/cimb43030098 (PMC8928951; doi:10.3390/cimb43030098)
Supplement: Supplementary file 1 [file cimb-43-00098-s001.zip › cimb-1373744-supplementary.pdf]

Article

Diversification of Ferredoxins across Living Organisms

Nomfundo Nzuza [1,†], Tiara Padayachee [1,†], Wanping Chen [2], Dominik Gront [3], David R Nelson [4] and
Khajamohiddin Syed [1,*]

1 Department of Biochemistry and Microbiology, Faculty of Science and Agriculture, University of Zululand, KwaDlangezwa 3886, South Africa; nomfundonzuza11@gmail.com (N.N.); teez07padayachee@gmail.com (T.P.)
2 Department of Molecular Microbiology and Genetics, University of Göttingen, 37077 Göttingen, Germany; chenwanping1@foxmail.com
3 Faculty of Chemistry, Biological and Chemical Research Center, University of Warsaw, Pasteura 1, 02-093 Warsaw, Poland; dgront@gmail.com
4 Department of Microbiology, Immunology and Biochemistry, University of Tennessee Health Science Center, Memphis, TN 38163, USA; drnelson1@gmail.com
* Correspondence: khajamohiddinsyed@gmail.com; Tel.: +27-(035)-902-6857
† These authors contributed equally to the work.

Citation: Nzuza, N.; Padayachee, T.; Chen, W.; Gront, D.; Nelson, D.R.; Syed, K. Diversification of Ferredoxins across Living Organisms. *Curr. Issues Mol. Biol.* **2021**, *43*, 1374–1390. https://doi.org/10.3390/cimb43030098

Academic Editor: Hidayat Hussain

Received: 30 August 2021
Accepted: 23 September 2021
Published: 30 September 2021

Publisher's Note: MDPI stays neutral with regard to jurisdictional claims in published maps and institutional affiliations.

Copyright: © 2021 by the authors. Submitted for possible open access publication under the terms and conditions of the Creative Commons Attribution (CC BY) license (http://creativecommons.org/licenses/by/4.0/).

Table S1. Comparative analysis of ferredoxins in the bacterial groups *Alphaproteobacteria* and *Firmicutes*.

Alphaproteobacteria

**Species name**	**Species code**	**Ferredoxin types**					
		**2Fe-2S**	**3Fe-4S**	**4Fe-4S**	**7Fe-8S**	**2[4Fe-4S]**	**2[4Fe-4S]Alv**
Rhizobium favelukesii	rhl	1				3	
Mesorhizobium japonicum MAFF 303099	mlo	1				4	
Mesorhizobium loti NZP2037	mln	2				4	
Mesorhizobium ciceri (biovar Biserrulae)	mci	1				4	
Mesorhizobium opportunistum	mop	1	1			3	
Mesorhizobium australicum	mam	1	1			4	
Mesorhizobium amorphae	mamo	4	1			3	
Mesorhizobium sp. B7	meso	1				2	
Chelativorans sp. BNC1	mes	1				2	
Hoeflea sp. IMCC20628	hoe	2				2	
Aminobacter aminovorans	aak	7				4	
Parvibaculum lavamentivorans	pla	5				2	
Sinorhizobium meliloti 1021	sme					3	
Sinorhizobium meliloti AK83	smk	1				5	1
Sinorhizobium meliloti BL225C	smq	1				5	
Sinorhizobium meliloti SM11	smx					5	1
Sinorhizobium meliloti Rm41	smi	1				5	
Sinorhizobium meliloti GR4	smeg	1				5	
Sinorhizobium meliloti 2011	smel	1				5	
Sinorhizobium meliloti RMO17	smer	1				5	
Sinorhizobium medicae	smd	1				4	
Sinorhizobium fredii NGR234	rhi	4	1			4	

Sinorhizobium fredii HH103	sfh	2				3	
Sinorhizobium fredii USDA 257	sfd	2				3	
Sinorhizobium sp. RAC02	six	1				6	
Sinorhizobium americanum	same	2	1			7	
Ensifer adhaerens OV14	ead	2				3	
Ensifer adhaerens Casida A	eah	2			1	6	
Agrobacterium fabrum	atu	1				3	
Agrobacterium radiobacter	ara	1				3	
Agrobacterium tumefaciens Ach5	atf	1				3	
Agrobacterium tumefaciens S33	ata	2				3	
Agrobacterium vitis	avi	1				3	
Agrobacterium sp. H13-3	agr	1				5	
Agrobacterium sp. RAC06	agc	1				1	
Agrobacterium rhizogenes	aro	1				3	
Rhizobium etli CFN 42	ret	1	1			4	
Rhizobium etli CIAT 652	rec	1	1			4	
Rhizobium etli bv. *mimosae* Mim1	rel	1	1			5	
Rhizobium etli bv. *phaseoli* IE4803	rep	2				4	
Rhizobium sp. IE4771	rei	2	1			5	
Rhizobium leguminosarum bv. *viciae* 3841	rle	2			1	2	1
Rhizobium leguminosarum bv. *trifolii* WSM2304	rlt	1				4	
Rhizobium leguminosarum bv. *trifolii* WSM1325	rlg	1				3	
Rhizobium leguminosarum bv. *trifolii* WSM1689	rlb	1				2	
Rhizobium leguminosarum bv. *trifolii* CB782	rlu	1				3	
Rhizobium tropici	rtr	2	2			3	
Rhizobium sp. IRBG74	rir	1				4	
Rhizobium gallicum	rga	2	2			4	
Rhizobium sp. N1341	rhn	1	1		1	4	
Rhizobium phaseoli	rpha	1	1			4	

Rhizobium sp. NT-26	rht	1				2	
Rhizobium sp. N731	rhx	1	1		1	4	
Neorhizobium galegae bv. *officinalis* bv. *officinalis* HAMBI 1141	ngl	2			1	3	1
Neorhizobium galegae bv. *orientalis* HAMBI 540	ngg	2			1	3	
Liberibacter crescens	lcc					1	
Shinella sp. HZN7	shz	2				3	
Brucella melitensis bv. 1 16M	bme	1				2	
Brucella melitensis bv. 1 16M	bmel	1				2	
Brucella melitensis ATCC 23457	bmi	1				2	
Brucella melitensis M28	bmz	1				2	
Brucella melitensis M5-90	bmg	1				2	
Brucella melitensis NI	bmw	1				2	
Brucella melitensis bv. 3 Ether	bmee	1				2	
Brucella abortus 2308	bmf					2	
Brucella abortus S19	bmc	1				2	
Brucella abortus A13334	baa	1				2	
Brucella abortus bv. 2 86/8/59	babo	1				2	
Brucella abortus bv. 6 870	babr	1				2	
Brucella abortus 63 75	babt	1				2	
Brucella abortus BDW	babb	1				2	
Brucella abortus bv. 9 C68	babu	1				2	
Brucella abortus BER	babs					2	
Brucella abortus NCTC 10505	babc	1				2	
Brucella suis bv. 2 Bs143CITA	bsui					2	
Brucella suis bv. 2 PT09143	bsup					2	
Brucella suis bv. 2 PT09172	bsuv					2	
Brucella suis bv. 2 Bs364CITA	bsuc	1				2	
Brucella suis bv. 3	bsz	1				2	

Brucella suis ZW043	bsw	1				2	
Brucella suis ZW046	bsg	1				3	
Brucella canis ATCC 23365	bcs	1				2	
Brucella canis HSK A52141	bsk	1				2	
Brucella canis RM6/66	bcar	1				2	
Brucella canis SVA13	bcas	1				2	
Brucella pinnipedialis 6/566	bpv	1				2	
Brucella ceti TE10759-12	bcet	1				2	
Ochrobactrum anthropi ATCC 49188	oan	1				2	
Ochrobactrum anthropi OAB	oah	1				2	
Ochrobactrum pseudogrignonense	ops	1				2	
Bradyrhizobium diazoefficiens USDA 110	bja	3	1			5	1
Bradyrhizobium japonicum USDA 6	bju	2	2			4	
Bradyrhizobium japonicum E109	bjp	4	2			5	
Bradyrhizobium sp. ORS 278	bra	4				4	1
Bradyrhizobium sp. BTAi1	bbt	6			1	7	1
Bradyrhizobium sp. S23321	brs	3	1			4	1
Bradyrhizobium oligotrophicum	aol	6			1	5	1
Bradyrhizobium sp. CCGE-LA001	brc	3	1			2	1
Bradyrhizobium sp. BF49	brad	1	1			3	
Bradyrhizobium icense	bic	3	2		1	6	1
Rhodopseudomonas palustris CGA009	rpa	2	1			5	1
Rhodopseudomonas palustris HaA2	rpb	2	1			6	1
Rhodopseudomonas palustris BisB18	rpc	1				5	1
Rhodopseudomonas palustris BisB5	rpd	1	1			6	1
Rhodopseudomonas palustris BisA53	rpe	1				5	1
Rhodopseudomonas palustris TIE-1	rpt	2	1			6	1
Rhodopseudomonas palustris DX-1	rpx	2				6	1
Nitrobacter winogradskyi	nwi	1				4	

Nitrobacter hamburgensis	nha	1	1			3	
Bosea sp. RAC05	bos	1				2	
Bosea vaviloviae	bvv	3				2	
Xanthobacter autotrophicus	xau	3				7	1
Azorhizobium caulinodans	azc	4				5	1
Methylorubrum extorquens PA1	mex	1	1			3	
Methylorubrum extorquens AM1	mea	1	1			3	
Methylorubrum extorquens DM4	mdi	1	1			3	
Methylorubrum extorquens CM4	mch	1	1			5	
Methylorubrum populi	mpo	1	1			3	
Methylobacterium radiotolerans	mrd	1	2			3	
Methylobacterium sp. 4-46	met	1	1			3	
Methylobacterium nodulans	mno	2	1			3	
Methylobacterium oryzae	mor	1	2			2	
Methylobacterium sp. AMS5	meta	1	1			2	
Methylobacterium aquaticum	maqu	1	2			2	
Beijerinckia indica	bid	7				5	
Methylocella silvestris	msl	6				3	1
Hyphomicrobium denitrificans ATCC 51888	hdn	1				3	
Hyphomicrobium denitrificans 1NES1	hdt	1				3	
Hyphomicrobium sp. MC1	hmc	4				3	
Hyphomicrobium nitrativorans	hni	1				3	
Pelagibacterium halotolerans	phl	2				2	
Candidatus Filomicrobium marinum W	fil	1				3	
Candidatus Filomicrobium marinum Y	fiy	1				3	
Devosia sp. H5989	deq	2				2	
Rhodoplanes sp. Z2-YC6860	rhz	2				3	
Methylocystis sp. SC2	msc	6				3	1
Methylocystis bryophila	mbry	6				5	1

Martelella endophytica	mey	1				5	
Methyloceanibacter caenitepidi	mcg	2				2	
Pseudorhodoplanes sinuspersici	psin	6				3	
Caulobacter vibrioides CB15	ccr	1				2	
Caulobacter vibrioides NA1000	ccs	1				2	
Caulobacter sp. K31	cak	1	1			2	
Caulobacter segnis	cse	2				3	
Phenylobacterium zucineum	pzu	1				2	
Asticcacaulis excentricus	aex	2				2	
Ruegeria pomeroyi	sil	3				2	
Ruegeria sp. TM1040	sit	1				2	
Rhodobacter sphaeroides 2.4.1	rsp	2				6	
Rhodobacter sphaeroides ATCC 17029	rsh	2				4	
Rhodobacter sphaeroides ATCC 17025	rsq	1				4	
Rhodobacter sphaeroides KD131	rsk	2				5	
Rhodobacter capsulatus	rcp	3				3	1
Jannaschia sp. CCS1	jan	1			1	2	
Roseobacter denitrificans	rde	2				2	
Roseobacter litoralis	rli	2				2	
Paracoccus denitrificans	pde	2				2	
Paracoccus aminophilus	pami	1				4	
Paracoccus yeei	pye	1				2	
Dinoroseobacter shibae	dsh	1				2	
Ketogulonicigenium vulgare Y25	kvu	1				2	
Ketogulonicigenium vulgare WSH-001	kvl	1				2	
Ketogulonicigenium robustum	kro	1				2	
Pseudovibrio sp. FO-BEG1	psf	1				2	
Phaeobacter inhibens DSM 17395	pga	1				2	
Phaeobacter inhibens 2.10	pgl	1				2	

Phaeobacter gallaeciensis DSM 26640	pgd	1				2	
Phaeobacter porticola	php	1				2	
Octadecabacter arcticus	oar	2				3	
Octadecabacter temperatus	otm	1			1	5	
Leisingera methylohalidivorans	lmd	2				2	
Roseibacterium elongatum	red	1				3	
Planktomarina temperata	ptp	2				2	
Celeribacter indicus	cid	4				5	
Celeribacter marinus	cmar	1				2	
Marinovum algicola	malg	1				2	
Confluentimicrobium sp. EMB200-NS6	con	7				4	
Rhodovulum sulfidophilum	rsu	2				3	1
Rhodovulum sp. P5	rhc	2				4	1
Pannonibacter phragmitetus	pphr	4				2	
Labrenzia sp. CP4	lap	2				2	
Labrenzia aggregata	lagg	2			1	2	
Defluviimonas alba	daa	3				2	1
Yangia sp. CCB-MM3	yan	3				3	
Sulfitobacter sp. AM1-D1	suam	1				2	
Salipiger profundus	tpro	1				2	
Tateyamaria omphalii	tom	1				2	
Pelagibaca abyssi	paby	1				3	
Thioclava nitratireducens	thw	2				2	
Roseovarius mucosus	rmm	1				3	
Yoonia vestfoldensis	lvs	2				2	
Maricaulis maris	mmr	1				2	
Hyphomonas neptunium	hne	1				2	
Hirschia baltica	hba	2				2	
Hyphomonadaceae bacterium UKL13-1	hbc	1				2	

Novosphingobium aromaticivorans	nar	7	1			2	
Novosphingobium sp. PP1Y	npp	4				3	
Novosphingobium pentaromativorans	npn	3				2	
Sphingopyxis alaskensis	sal	2				2	
Sphingopyxis fribergensis	sphk	6				2	
Sphingopyxis sp. 113P3	sphp	2				2	
Sphingopyxis macrogoltabida 203	smaz	3	2			2	
Sphingopyxis terrae	ster	4				2	
Sphingopyxis granuli	sgi	4	1			2	
Sphingopyxis sp. LPB0140	sphl	2				2	
Sphingomonas wittichii	swi	8	2			2	
Sphingomonas sp. MM-1	sphm	3	1			2	
Sphingomonas sp. WHSC-8	sphi	5				4	
Sphingobium japonicum	sjp	2	1			2	
Sphingobium chlorophenolicum	sch	5	2			2	
Sphingobium sp. SYK-6	ssy	3				2	
Sphingobium sp. YBL2	syb	6	1	1		3	
Sphingobium baderi	sbd	6				2	
Sphingobium sp. MI1205	spmi	4				2	
Sphingobium sp. EP60837	sphb	3	2			2	
Sphingobium sp. RAC03	sphr	3	1			2	
Sphingobium indicum	sinb	2				2	
Citromicrobium sp. JL477	cij	3				2	
Sphingorhabdus sp. M41	sphg	3				2	
Blastomonas sp. RAC04	blas	5		1		2	
Erythrobacter litoralis HTCC2594	eli	4				2	
Erythrobacter litoralis DSM 8509	elq	3				2	
Altererythrobacter atlanticus	aay	3				2	
Altererythrobacter marensis	amx	3				2	

Altererythrobacter epoxidivorans	aep	4				2	
Altererythrobacter namhicola	anh	4				2	
Altererythrobacter dongtanensis	ado	3				2	
Croceicoccus naphthovorans	cna	4				2	
Porphyrobacter neustonensis	pns	3				2	
Porphyrobacter sp. LM 6	porl	3				2	
Gluconacetobacter diazotrophicus PA1 5 (Brazil)	gdi	1				4	
Gluconacetobacter diazotrophicus PA1 5 (JGI)	gdj	1				4	
Acetobacter aceti	aace	1				3	
Magnetospirillum gryphiswaldense MSR-1 v2	mgy	4				4	1
Azospirillum sp. B510	azl	4				2	
Azospirillum brasilense Sp245	abs	4				4	1
Azospirillum brasilense Az39	abq	4				4	1
Tistrella mobilis	tmo	1				2	
Magnetospira sp. QH-2	magq	5				3	1
Polymorphum gilvum	pgv	4				2	
Candidatus Puniceispirillum marinum	apb	2				2	
**241**		**490**	**60**	**2**	**12**	**712**	**31**

Firmicutes

**Species name**	**Species code**	**2Fe-2S**	**4Fe-4S**	**7Fe-8S**	**2[4Fe-4S]**
Alicyclobacillus acidocaldarius subsp. *acidocaldarius* DSM 446	aac			1	
Alicyclobacillus acidocaldarius subsp. *acidocaldarius* Tc-4-1	aad			1	
Aneurinibacillus soli	asoc	1	1		
Aneurinibacillus sp. XH2	anx	1	1		
Anoxybacillus amylolyticus	aamy				
Abyssicoccus albus	sbac				
Bacillus altitudinis	balt		1		
Bacillus amyloliquefaciens CC178	bamc				
Bacillus amyloliquefaciens DSM 7	bao				
Bacillus amyloliquefaciens IT-45	bami				
Bacillus amyloliquefaciens LFB112	bamf				
Bacillus amyloliquefaciens LL3	bql				
Bacillus amyloliquefaciens TA208	baz				
Bacillus amyloliquefaciens XH7	bxh				
Bacillus amyloliquefaciens Y2	bgy				
Bacillus anthracis A0248	bai	1	1		
Bacillus anthracis A16	bant		1		
Bacillus anthracis A16R	banr		1		
Bacillus anthracis Ames	ban		1		
Bacillus anthracis Ames Ancestor	bar	1	1		
Bacillus anthracis CDC 684	bah	1	1		
Bacillus anthracis H9401	bax	1			
Bacillus anthracis HYU01	banh		1		

Bacillus anthracis Sterne	bat	1	1		
Bacillus anthracis SVA11	bans	1			
Bacillus anthracis Vollum	banv		1		
Bacillus atrophaeus	bae				
Bacillus bombysepticus	bby		1		
Bacillus cellulosilyticus	bco	1			
Bacillus cereus 03BB102	bcx	1	1		
Bacillus cereus AH820	bcu	1	1		
Bacillus cereus ATCC 10987	bca	1	2		
Bacillus cereus ATCC 14579	bce	1	1		
Bacillus cereus B4264	bcb	1	1		
Bacillus cereus biovar anthracis CI	bal	1	1		
Bacillus cereus E33L	bcz	1	1		
Bacillus cereus F837/76	bcf	1	1		
Bacillus cereus FRI-35	bcer	1	2		
Bacillus cereus FT9	bcef	1	2		
Bacillus cereus G9842	bcg	1	1		
Bacillus cereus NC7401	bnc	1	1		
Bacillus cereus Q1	bcq	1	1		
Bacillus clausii	bcl	1	1	2	
Bacillus coagulans 36D1	bag		1		
Bacillus cytotoxicus	bcy	1	1		
Priestia flexa	bfx		1		
Bacillus gibsonii	bgi				
Bacillus glycinifermentans	bgy				
Bacillus halodurans	bha		2		
Bacillus infantis	bif				
Bacillus lehensis	ble	1	1	1	
Bacillus licheniformis ATCC 14580	bli				

Bacillus licheniformis DSM 13 = ATCC 14580	bld				
Bacillus megaterium DSM 319	bmd	1	2		
Bacillus megaterium NBRC 15308 = ATCC 14581	bmeg		2		
Bacillus megaterium QM B1551	bmq		2		
Bacillus megaterium WSH-002	bmh	1			
Bacillus mycoides ATCC 6462	bmyo		2		
Bacillus mycoides KBAB4	bwe	1	1		
Bacillus mycoides WSBC 10204	bww	1	1		
Bacillus oceanisediminis	bon	2	1		
Bacillus paralicheniformis	blh				
Bacillus pseudofirmus	bpf		1		
Bacillus pseudomycoides 219298	bmyc		1		
Bacillus pumilus MTCC B6033	bpum		1		
Bacillus pumilus SAFR-032	bpu		1		
Bacillus pumilus SH-B9	bpus		1		
Bacillus selenitireducens	bse				
Peribacillus simplex	bsj		1		
Bacillus smithii	bsm		1		
Bacillus sp. BH072	bacb				
Bacillus sp. BS34A	bacl				
Bacillus sp. JS	bjs				
Bacillus sp. LM 4-2	balm				
Bacillus sp. OxB-1	baco		1		
Bacillus sp. Pc3	bacp				
Bacillus sp. SDLI1	bacs				
Bacillus sp. WP8	bacw		1		
Bacillus sp. YP1	bacy				
Bacillus subtilis BSn5	bsn				
Bacillus subtilis PY79	bsp				

Bacillus subtilis QB928	bsq				
Bacillus subtilis subsp. *natto* BEST195	bso				
Bacillus subtilis subsp. *spizizenii* TU-B-10	bst				
Bacillus subtilis subsp. *spizizenii* W23	bss				
Bacillus subtilis subsp. *subtilis 1*68	bsu				
Bacillus subtilis subsp. *subtilis* 6051-HGW	bsh				
Bacillus subtilis subsp. *subtilis* AG1839	bsut				
Bacillus subtilis subsp. *subtilis* BAB-1	bsy				
Bacillus subtilis subsp. *subtilis* BSP1	bsl				
Bacillus subtilis subsp. *subtilis* JH642	bsul				
Bacillus subtilis subsp. *subtilis* OH 131.1	bsus				
Bacillus subtilis subsp. *subtilis* RO-NN-1	bsr				
Bacillus subtilis XF-1	bsx				
Bacillus thuringiensis Al Hakam	btl	1			
Bacillus thuringiensis BMB171	btb	1	1		
Bacillus thuringiensis Bt407	btg	1	1		
Bacillus thuringiensis HD1011	btw		1		
Bacillus thuringiensis HD-771	bti	1	1		
Bacillus thuringiensis HD-789	btn	1	1		
Bacillus thuringiensis MC28	btm	1	1		
Bacillus thuringiensis serovar chinensis CT-43	btc	1	1		
Bacillus thuringiensis serovar finitimus YBT-020	btf	1	1		
Bacillus thuringiensis serovar kurstaki HD-1	bthi	1	1		
Bacillus thuringiensis serovar kurstaki HD73	btt	1	1		
Bacillus thuringiensis serovar kurstaki YBT-1520	bthr	1	1		
Bacillus thuringiensis serovar thuringiensis IS5056	btht	1	1		
Bacillus thuringiensis YBT-1518	bthu	1	1		
Bacillus thuringiensis YWC2-8	bthy		1		
Bacillus toyonensis	bty	1	1		

Bacillus vallismortis	bvm				
Bacillus velezensis JS25R	bmp				
Bacillus velezensis AS43.3	bamp				
Bacillus velezensis CAU B946	baq				
Bacillus velezensis FZB42	bay				
Bacillus velezensis NAU-B3	bamb				
Bacillus velezensis SQR9	bamy				
*Bacillus velezensis TrigoCor*1448	bamt				
Bacillus velezensis UCMB5033	bama				
Bacillus velezensis UCMB5036	baml				
Bacillus velezensis UCMB5113	bamn				
Bacillus velezensis YAU B9601-Y2	bya				
Bacillus xiamenensis	bxi		1		
Brevibacillus brevis	bbe	1	1		
Brevibacillus laterosporus	blr	1	1		
Carnobacterium sp. CP1	carc				
Cellulosilyticum lentocellum	cle				
Clostridium acetobutylicum ATCC 824	cac		1		1
Clostridium acetobutylicum DSM 1731	cae		1		1
Clostridium acetobutylicum EA 2018	cay		1		1
Clostridium bornimense	clt				
Clostridium botulinum A ATCC 19397	cba				
Clostridium botulinum A ATCC 3502	cbo				
Clostridium botulinum A Hall	cbh				
Clostridium botulinum A2	cby				
Clostridium botulinum A3 Loch Maree	cbl				
Clostridium botulinum B1 Okra	cbb				
Clostridium botulinum Ba4	cbi				
Clostridium botulinum F 230613	cbm				

Clostridium botulinum F Langeland	cbf				
Clostridium botulinum H04402 065	cbj		1		
Clostridium pasteurianum BC1	cpas	1	1		
Clostridium pasteurianum DSM 525 = ATCC 6013	cpae	1			1
Clostridium pasteurianum DSM 525 = ATCC 6013	cpat				
Clostridium saccharolyticum WM1	csh		1		1
Clostridium sporogenes	cld		1		
Enterococcus faecium ATCC 8459 = NRRL B-2354	efm				
Enterococcus faecium Aus0004	efc				
Enterococcus faecium Aus0085	efau				
Enterococcus faecium DO	efu				
Enterococcus faecium T110	eft				
Enterococcus gilvus	egv		1		
Enterococcus hirae	ehr				
Enterococcus mundtii	emu				
Exiguobacterium sp. AT1b	eat		1		
Halobacillus halophilus	hhd		1	2	
Halobacillus mangrovi	hmn		1	1	
Herbinix luporum	hsd				
Jeotgalibaca dankookensis	jda		1		
Jeotgalibaca ciconiae	jeh		1		
Kyrpidia spormannii	kyr	4	1	1	
Laceyella sacchari	lfb	1	1		
Lachnoclostridium phytofermentans	cpy				
Lentibacillus amyloliquefaciens	lao		1	3	
Limnochorda pilosa	lpil	1		1	
Macrococcus caseolyticus	mcl				
Macrococcus sp. IME1552	macr		1		
Marinilactibacillus sp. 15R	marr				

Oceanobacillus iheyensis	oih			2	
Oceanobacillus sp. 160	ocn		1	3	
Paenibacillus beijingensis	pbj	4	1		
Paenibacillus bovis	pbv	1	1		
Paenibacillus donghaensis	pdh	1	2		
Paenibacillus ihbetae	pib	2	1	1	
Paenibacillus larvae	plv	2	1		
Paenibacillus lautus	plw	1	1		
Paenibacillus mucilaginosus 3016	pmq	2	1		1
Paenibacillus mucilaginosus K02	pmw	2	1		2
Paenibacillus mucilaginosus KNP414	pms	2	1		1
Paenibacillus naphthalenovorans	pnp	1	1		1
Paenibacillus peoriae	ppeo	2	1		
Paenibacillus polymyxa CR1	ppol	2	1		
Paenibacillus polymyxa M1	ppo	1	1		
Paenibacillus polymyxa Sb3-1	ppoy	2	1		
Paenibacillus polymyxa SC2	ppm	2	1		
Paenibacillus polymyxa SQR-21	ppq	1	1		
Paenibacillus riograndensis	pri	1	2		
Paenibacillus sp. 32O-W	pow	2	1		1
Paenibacillus sp. IHBB 10380	pih	1	1		
Paenibacillus sp. Y412MC10	gym	2	1		
Paenibacillus swuensis	pswu	2	1		
Paenibacillus xylanexedens	pxl	1	1		
Planococcus antarcticus	pana	1	1		
Planococcus halocryophilus	phc		1		
Planococcus maritimus	pmar		1		
Planococcus plakortidis	ppla		1		
Planococcus rifietoensis	prt		1		

Planococcus sp. MB-3u-03	plx				
Ruminococcaceae bacterium CPB6	rbp				1
Rummeliibacillus stabekisii	rst		2		
Salimicrobium jeotgali	sje		1	3	
Salinicoccus halodurans	shv			1	
Solibacillus silvestris DSM 12223	ssil		1		
Solibacillus silvestris StLB046	siv		1		
Sporosarcina sp. P33	spor		1		
Sporosarcina sp. P37	spop		2		
Sporosarcina ureae	sure		1		
Staphylococcus agnetis	sagq				
Staphylococcus epidermidis SEI	seps		1		
Staphylococcus felis	sfq				
Staphylococcus haemolyticus JCSC1435	sha		1		
Staphylococcus hyicus	shu				
Staphylococcus lutrae	slz		1		
Staphylococcus pseudintermedius ED99	sdt		1		
Staphylococcus pseudintermedius HKU10-03	ssd				
Staphylococcus sciuri	sscu				
Sulfobacillus acidophilus DSM 10332	sap	1			
Sulfobacillus acidophilus TPY	say	1			
Terribacillus goriensis	tap			1	
Tetragenococcus halophilus NBRC 12172	thl		1		
Tetragenococcus koreensis	tkr		1		
Tetragenococcus osmophilus	too				
Virgibacillus halodenitrificans	vhl	1	1	2	
Virgibacillus necropolis	vne		1	2	
Virgibacillus phasianinus	vil		1	2	
Virgibacillus sp. SK37	vir	1	1	2	

Weissella jogaejeotgali	wjo				
**227**		**97**	**140**	**32**	**12**

Table S2. Ferredoxin protein sequences identified in 241 alphaproteobacterial species and 227 *Firmicutes* species. Ferredoxins were sorted as per their Fe-S cluster type and subtypes. Name of the ferredoxins were followed as mentioned in methodology. The name of the ferredoxins includes their nomenclature name followed by protein ID from KEGG database and species name.

Alphaproteobacteria

2Fe-2S

Subtype 1

>2Fe-2SST1(rhl:LPU83_2264)Rhizobium favelukesii

MPKLTIVAFDGTRFDLDVDQGSTVMENAVRNSVPGIEAECGGACACATCHVYVDDEWTER

VGPPEAMEEDMLDFAFDVRPTSRLSCQIRMKAALDGLTVHVPERQA

>2Fe-2SST1(mlo:msl0793)Mesorhizobium japonicum MAFF 303099

MTKLTFIAHDGTQFDVDAENGSTVMENAIRNAVPGIEAECGGACACATCHVYVDEAWTAE

VGEPEAMEEDMLDFAYDVQPNSRLSCQIKVRDALDGLVVRVPERQG

>2Fe-2SST1(mln:A9174_21805)Mesorhizobium loti NZP2037

MTKLTFIAHDGTQFDVDAENGSTVMENAIRNAVPGIEAECGGACACATCHVYVDEAWTAE

VGEPEAMEEDMLDFAYDVQPNSRLSCQIKVRDALDGLVVRVPERQG

>2Fe-2SST1(mci:Mesci_4101)Mesorhizobium ciceri (biovar Biserrulae)

MTKLTFIAHDGTHFDMDAENGSTVMENAIRNAVPGIEAECGGACACATCHVYVDEAWTAE

VGEPEAMEEDMLDFAYDVQPNSRLSCQIKVRDALDGLVVRVPARQG

>2Fe-2SST1(mop:Mesop_4144)Mesorhizobium opportunistum

MTKLTFIAHDGTHFDVDAENGSTVMENAIRNAVPGIEAECGGACACATCHVYVDEAWTAE

VGEPEAMEEDMLDFAYEVQPNSRLSCQIKVRDALDGLVVRVPERQG

>2Fe-2SST1(mam:Mesau_03907)Mesorhizobium australicum

MTKLTFIAHDGTHFDVDAENGSTVMENAIRNAVPGIEAECGGACACATCHVYVDEAWTAE
VGEPEAMEEDMLDFAYDVQPNSRLSCQIKVRDALDGLIVRVPERQG
>2Fe-2SST1(mamo:A6B35_18150)Mesorhizobium amorphae
MTKLTFIAHDGTQFDVDAENGSTVMENAIRNAVPGIEAECGGACACATCHVYVDEAWTAE
VGEPEAMEEDMLDFAYEVQPNSRLSCQIKVRDALDGLVVRVPARQG
>2Fe-2SST1(meso:BSQ44_12710)Mesorhizobium sp. B7
MTKITYIAHDGSKFEVEAENGSTVMENAIRNAVPGIEAECGGACACATCHVYVDEAWSAA
VGEPEAMEEDMLDFAYDVRPTSRLSCQIRVSDELDGLVVQVPERQA
>2Fe-2SST1(mes:Meso_1410)Chelativorans sp. BNC1
MPRLKFIAFDGTEFDIQADNGSTLMQNAVRNGVPGIEAECGGACACATCHVYVDEAWAEI
VGPPEPMEEDMLDFAYDVRPTSRLSCQVRVREELDGLTVRIPERQG
>2Fe-2SST1(hoe:IMCC20628_01555)Hoeflea sp. IMCC20628
MTKITLVAYDGTRFEVAAENGSTVMENAIRNSVPGIEAECGGACACATCHVYVDEQWVDA
VGAPAPMEEDMLDFAFDVRPTSRLSCQIKVRSELDGLVVHVPERQA
>2Fe-2SST1(aak:AA2016_0623)Aminobacter aminovorans
MYRVTFVDADGQATELTANEGQSVMSVAVANGIDQILAECGGSCACGTCHCYVDESWTEK
LPAADDSEQGMIECLMNPTERSRLTCQLKMSAALDGLVVHLPREQY
>2Fe-2SST1(aak:AA2016_3872)Aminobacter aminovorans
MTKLTYIAHDGTRFEVEAENGSTVMENAIRNAVPGIEAECGGACACATCHVYVDDAWTAI
VGEPEAMEEDMLDFAYEVRPSSRLSCQIKVRDELDGLVVSIPERQG
>2Fe-2SST1(aak:AA2016_5379)Aminobacter aminovorans
MIEINFTEADGNTRILSADPGSTLLNAILGAGIKGIVAECGGGCSCGTCHVHVALDWTGR
LPAPADDETDMLSLAEDVTEKSRLACQIRLAPELNGLSVTIPGAA
>2Fe-2SST1(pla:Plav_2520)Parvibaculum lavamentivorans
MAKITYIEHNGTEHTIDVENGMTVMEGAIKNSIPGIDADCGGACACATCHVYVAEGWTEK
VGPAESMEEDMLDFAFDVRENSRLSCQIKVTDELDGLKVNLPEKQF

>2Fe-2SST1(pla:Plav_0126)Parvibaculum lavamentivorans

MAKITYIEHDGTEHTIEVANGITLMEAAVKASIPGIDGDCGGACACATCMVYVPDEWKPK

LPEVETMEETMLDFCEHTEANSRLSCQLVASDELDGIRLQMPESQH

>2Fe-2SST1(smk:Sinme_1719)Sinorhizobium meliloti AK83

MTKLTIVAFDGARHELDVENGSTVMENAVRNSVPGIEAECGGACACATCHVYVDDAWAAQ

VGTPEAMEEDMLDFAYDVRPTSRLSCQIKMSEALDGLVVHVPERQA

>2Fe-2SST1(smq:SinmeB_1564)Sinorhizobium meliloti BL225C

MTKLTIVAFDGARHELDVENGSTVMENAVRNSVPGIEAECGGACACATCHVYVDDAWAAQ

VGTPEAMEEDMLDFAYDVRPTSRLSCQIKMSEALDGLVVHVPERQA

>2Fe-2SST1(smi:BN406_01547)Sinorhizobium meliloti Rm41

MTKLTIVAFDGARHELDVENGSTVMENAVRNSVPGIEAECGGACACATCHVYVDDAWAAQ

VGTPEAMEEDMLDFAYDVRPTSRLSCQIKMSEALDGLVVHVPERQA

>2Fe-2SST1(smeg:C770_GR4Chr1864)Sinorhizobium meliloti GR4

MTKLTIVAFDGARHELDVENGSTVMENAVRNSVPGIEAECGGACACATCHVYVDDAWAAQ

VGTPEAMEEDMLDFAYDVRPTSRLSCQIKMSEALDGLVVHVPERQA

>2Fe-2SST1(smel:SM2011_c00192)Sinorhizobium meliloti 2011

MTKLTIVAFDGARHELDVENGSTVMENAVRNSVPGIEAECGGACACATCHVYVDDAWAAQ

VGTPEAMEEDMLDFAYDVRPTSRLSCQIKMSEALDGLVVHVPERQ

>2Fe-2SST1(smer:DU99_09905)Sinorhizobium meliloti RMO17

MTKLTIVAFDGARHELDVENGSTVMENAVRNSVPGIEAECGGACACATCHVYVDDAWAAQ

VGTPEAMEEDMLDFAYDVRPTSRLSCQIKMSEALDGLVVHVPERQA

>2Fe-2SST1(smd:Smed_1536) Sinorhizobium medicae

MTKLTIVAFDGARHELDVENGSTVMENAVRNSVPGIEAECGGACACATCHVYVDDAWAAQ

VGAPEAMEEDMLDFAYDVRPTSRLSCQIKVSEALDGLIVHVPERQA

>2Fe-2SST1(rhi:NGR_c16660)Sinorhizobium fredii NGR234

MTKLTIVAFDGTRHDLDVENGSTVMENAVRNSVPGIEAECGGACACATCHVYVDDAWAAA

VGAPEAMEEDMLDFAYDVRPTSRLSCQIKIGEALDGLVVHVPERQA
>2Fe-2SST1(sfh:SFHH103_01547)Sinorhizobium fredii HH103
MTKLTIVAFDGTRHDLDVENGSTVMENAVRNSVPGIEAECGGACACATCHVYVDDAWAAA
VGAPEAMEEDMLDFAYDVRPTSRLSCQIKIGEALDGLVVHVPERQA
>2Fe-2SST1(sfd:USDA257_c40890)Sinorhizobium fredii USDA 257
MTKLTIVAFDGTRHDLDVENGSTVMENAVRNSVPGIEAECGGACACATCHVYVDDAWAAA
VGAPEAMEEDMLDFAYDLRATSRLSCQIKMSEALDGLVVHVPERQA
>2Fe-2SST1(six:BSY16_912)Sinorhizobium sp. RAC02
MSKLTIVAFDGTRHELDVAKGSTVMENAVRNSVPGIEAECGGACACATCHVYVDDAWSAI
VGPPEAMEEDMLDFAYDVRPTSRLSCQIKMSDAFDGLVVHVPERQA
>2Fe-2SST1(ead:OV14_a0405)Ensifer adhaerens OV14
MDAEGKSTEVEAEIGQSVMSAAVQNGIDAIAAECGGSCACGTCHCYIDGTWAGRLPSPDY
QEQDMLDCVVNPDERSRLSCQITVSDALDGLVVHLPAGQY
>2Fe-2SST1(ead:OV14_2992)Ensifer adhaerens OV14
MTKLTIVAFDGTRHELDVENGSTVMENAVRNSVPGVEAECGGACACATCHVYVDDAWTAV
VGAPEAMEEDMLDFAYDVRPTSRLSCQIKVSDALDGLVVHVPERQA
>2Fe-2SST1(eah:FA04_22110)Ensifer adhaerens Casida A
MPKIVFIDADGTTKEIHAEIGQSLMSAAVQNGIDAIAAECGGSCACGTCHCYIDGEWSDR
LPAPEFQEQDMIECVVNPNERSRLSCQITVSAELDGLVVHLPAGQY
>2Fe-2SST1(eah:FA04_07845)Ensifer adhaerens Casida A
MTKLTIVAFDGARHELDVENGSTVMENAVRNSVPGIEAECGGACACATCHVYVDDAWAQT
VGAPEAMEEDMLDFAYDVRPTSRLSCQIKMSDALDGLVVHVPERQA
>2Fe-2SST1(atu:Atu1350)Agrobacterium fabrum
MTKLTIVAFDGTPHELDVSNGSTVMENAVRNSVPGIDAECGGACACATCHVYVDDAWAER
VGGPEPMEEDMLDFAFEVRPTSRLSCQIKMNDELDGLVVHVPERQG
>2Fe-2SST1(ara:Arad_2639)Agrobacterium radiobacter

MTKLTIVAFDGTRFDLNADDGSTVMENAVRNSVPGVDAECGGACACATCHVYVDDNWVEK
VGGPAPMEEDMLDFAFDVRPSSRLSCQIKMTQALDGLVVHVPERQA
>2Fe-2SST1(atf:Ach5_12480)Agrobacterium tumefaciens Ach5
MTKLTIVAFDGTPHELDVSNGSTVMENAVRNSIPGIDAECGGACACATCHVYVDDAWAER
VGGPEPMEEDMLDFAFEVRPTSRLSCQIKMKDELDGLVVHVPERQG
>2Fe-2SST1(ata:AWN88_12060)Agrobacterium tumefaciens S33
MTKLTIVAFDGTPHELDVSNGSTVMENAVRNSIPGIDAECGGACACATCHVYVDDAWSER
VGGPEPMEEDMLDFAFDVRPTSRLSCQIKMSDDLDGLVVHVPERQG
>2Fe-2SST1(avi:Avi_2243)Agrobacterium vitis
MTQLTIVAFDGTRFDLDAADGSTVMENAVRNSVPGIDAECGGACACATCHVYVDEAWVEK
TGGPAPMEEDMLDFATDVRPNSRLSCQIKIAPALAGLVVHVPERQG
>2Fe-2SST1(agr:AGROH133_05800)Agrobacterium sp. H13-3
MTKLTIVAFDGTPHELDVSNGSTVMENAVRNSIPGIDAECGGACACATCHVYVDDAWAER
VGGPEPMEEDMLDFAFEVRPTSRLSCQIKMKDELDGLVVHVPERQG
>2Fe-2SST1(agc:BSY240_360)Agrobacterium sp. RAC06
MANISIIAFDGTRFDIAAADGSTVMENAVRNSVPGIDAECGGACACATCHVYVDDSWSEK
VGQPSAMEEDMLDFAVDVRPTSRLSCQIKVTAALEGLVVHVPERQG
>2Fe-2SST1(aro:B0909_06650)Agrobacterium rhizogenes
MTKLTIVAFDGTPHELDVSNGSTVMENAVRNSIPGIDAECGGACACATCHVYVDDAWTER
VGGPEPMEEDMLDFAFDVRPTSRLSCQIKMNDELDGLVVHVPERQG
>2Fe-2SST1(ret:RHE_CH02338A)Rhizobium etli CFN 42
MPKLTIVAFDGTRFDLDVDQGSTVMENAVRNSVPGIEAECGGACACATCHVYVDEEWTEK
VGQPEAMEEDMLDFAFDVRPTSRLSCQIRMKAAYDGLVVHVPERQA
>2Fe-2SST1(rec:RHECIAT_CH0002436)Rhizobium etli CIAT 652
MPKLTIVAFDGTHFDLDVDQGSTVMENAVRNSVPGIEAECGGACACATCHVYVDEEWTEK
VGQPEAMEEDMLDFAFDVRPTSRLSCQIRMKAAYDGLVVHVPERQA

>2Fe-2SST1(rel:REMIM1_CH02350)Rhizobium etli bv. mimosae Mim1
MPKLTIVAFDGTRFDLDVDQGSTVMENAVRNSVPGIEAECGGACACATCHVYVDEEWTEK
VGQPEAMEEDMLDFAFDVRPTSRLSCQIRMKAAYDGLVVHVPERQA
>2Fe-2SST1(rep:IE4803_CH02381)Rhizobium etli bv. phaseoli IE4803
MPKLTIVAFDGTRFDLDVDQGSTVMENAVRNSVPGIEAECGGACACATCHVYVDEEWTEK
VGQPEAMEEDMLDFAFDVRPTSRLSCQIRMKAAYDGLIVHVPERQA
>2Fe-2SST1(rei:IE4771_CH02468)Rhizobium sp. IE4771
MPKLTIVAFDGTRFDLDVDQGSTVMENAVRNSVPGIEAECGGACACATCHVYVDEEWTEK
VGQPEAMEEDMLDFAFDVRPTSRLSCQIRMKAAYDGLIVHVPERQA
>2Fe-2SST1(rle:RL2652)Rhizobium leguminosarum bv. viciae 3841
MPKLTIVAFDGTRFDLDVDQGSTVMENAVRNSVPGIEAECGGACACATCHVYVDEEWTEK
VGQPEAMEEDMLDFAFDVRPTSRLSCQIRMKAVYDGLVVHVPERQA
>2Fe-2SST1(rlt:Rleg2_1973)Rhizobium leguminosarum bv. trifolii WSM2304
MPKLTIVAFDGTRFDLDVDQGSTVMENAVRNSVPGIEAECGGACACATCHVYVDEAWTEQ
VGQPEAMEEDMLDFAFDVRPTSRLSCQIRMKAVYDGLVVHVPERQA
>2Fe-2SST1(rlg:Rleg_2185)Rhizobium leguminosarum bv. trifolii WSM1325
MPKLTIVAFDGTRFDLDVDQGSTVMENAVRNSVPGIEAECGGACACATCHVYVDEEWTEK
VGQPEAMEEDMLDFAFDVRPTSRLSCQIRMKAVYDGLVVHVPERQA
>2Fe-2SST1(rlb:RLEG3_21580)Rhizobium leguminosarum bv. trifolii WSM1689
MPKLTIVAFDGTRFDLDVDQGSTVMENAVRNSVPGIEAECGGACACATCHVYVDEEWTEK
VGQPEAMEEDMLDFAFDVRPTSRLSCQIRMKAVYDGLVVHVPERQA
>2Fe-2SST1(rlu:RLEG12_21290)Rhizobium leguminosarum bv. trifolii CB782
MPKLTIVAFDGTRFDLDVDQGSTVMENAVRNSVPGIEAECGGACACATCHVYVDEAWTEQ
VGQPEAMEEDMLDFAFDVRPTSRLSCQIRMKAVYDGLVVHVPERQA
>2Fe-2SST1(rtr:RTCIAT899_CH10015)Rhizobium tropici
MTKLTIVAFDGTRFDLNVDEGSTVMENAVRNSVPGIEAECGGACACATCHVYVDEEWTEK

VGGPEAMEEDMLDFAFDVRPNSRLSCQIKMKSALDGLVVHVPERQA

>2Fe-2SST1(rir:BN877_I1333)Rhizobium sp. IRBG74

MTKLTIVAFDGTPHELDVSNGSTVMENAVRNSIPGIDAECGGACACATCHVYVDDAWSER

VGGPEPMEEDMLDFAFDVRPTSRLSCQIKMSDDLDGLVVHVPERQG

>2Fe-2SST1(rga:RGR602_CH02127)Rhizobium gallicum

MPKLTIVAFDGTRFDLDVDQGSTVMENAVRNSVPGIEAECGGACACATCHVYVDEEWTER

VGPPEAMEEDMLDFAFDVRPTSRLSCQIRMKAALDGLIVQVPERQA

>2Fe-2SST1(rhn:AMJ98_CH02396)Rhizobium sp. N1341

MPKLTIVAFDGTRFDLDVDQGSTVMENAVRNSVPGIEAECGGACACATCHVYVDEEWTEK

VGQPEAMEEDMLDFAFDVRPTSRLSCQIRMKAAYDGLVVHVPERQA

>2Fe-2SST1(rpha:AMC79_CH02467)Rhizobium phaseoli

MPKLTIVAFDGTRFDLDVDQGSTVMENAVRNSVPGIEAECGGACACATCHVYVDEEWTEK

VGQPEAMEEDMLDFAFDVRPTSRLSCQIRMKAAYDGLVVHVPERQA

>2Fe-2SST1(rht:NT26_1928)Rhizobium sp. NT-26

MTKLTIVAFDGTRFDIDASNGSTVMENAVRNSVPGIEAECGGACACATCHVYVDDAWAEA

VGAPSAMEEDMLDFAHDVRPTSRLSCQIKMTDALEGLVVQVPERQG

>2Fe-2SST1(rhx:AMK02_CH02411) Rhizobium sp. N731

MPKLTIVAFDGTRFDLDVDQGSTVMENAVRNSVPGIEAECGGACACATCHVYVDEEWTEK

VGQPEAMEEDMLDFAFDVRPTSRLSCQIRMKAAYDGLVVHVPERQA

>2Fe-2SST1(ngl:RG1141_CH16740)Neorhizobium galegae bv. officinalis bv. officinalis HAMBI 1141

MTKLTIVAFDGTRFDIEAEPGSTVMENAVRNSVPGIEAECGGACACATCHVYVDEAWTEK

VGPPAPMEEDMLDFAFEVKPNSRLSCQIKVKADLDGLVVNVPERQA

>2Fe-2SST1(ngl:RG1141_PB00350)Neorhizobium galegae bv. officinalis bv. officinalis HAMBI 1141

MTTIHFQSPSGEVTTLKGVVGQSIMELATSNGVSAIEADCGGACACATCHVIVDQSWTER

LPAPGVMERDMLDFVAEPTSTSRLSCQLHITDDHDGLLLHLPSKQS

>2Fe-2SST1(ngg:RG540_CH17130)Neorhizobium galegae bv. orientalis HAMBI 540

MTKLTIVAFDGTRFDIEAEPGSTVMENAVRNSVPGIEAECGGACACATCHVYVDEAWTEK
VGPPAPMEEDMLDFAFEVKPNSRLSCQIKVKADLDGLVVNVPERQA
>2Fe-2SST1(shz:shn_09835)Shinella sp. HZN7
MSKLTIVAFDGTRHELDVANGSTVMENAVRNSVPGIEAECGGACACATCHVYVDDAWSAI
VGPPEAMEEDMLDFAYEVRPTSRLSCQIKMSDALDGLVVHVPERQA
>2Fe-2SST1(bme:BMEI0959)Brucella melitensis bv. 1 16M
MSQGIKMTKIVFVSADGATRTEVEADSGSSVMEAAIRNGIPGIDAECGGACACATCHVYV
DDDWADTVGGPDPMEEDMLDFAYEVRPTSRLSCQIRVTDDLEGLVVQVPERQN
>2Fe-2SST1(bmel:DK63_462)Brucella melitensis bv. 1 16M
MTKIVFVSADGATRTEVEADSGSSVMEAAIRNGIPGIDAECGGACACATCHVYVDDDWAD
TVGGPDPMEEDMLDFAYEVRPTSRLSCQIRVTDDLEGLVVQVPERQN
>2Fe-2SST1(bmi:BMEA_A1066)Brucella melitensis ATCC 23457
MTKIVFVSADGATRTEVEADSGSSVMEAAIRNGIPGIDAECGGACACATCHVYVDDDWAD
TVGGPDPMEEDMLDFAYEVRPTSRLSCQIRVTDDLEGLVVQVPERQN
>2Fe-2SST1(bmz:BM28_A1035)Brucella melitensis M28
MTKIVFVSADGATRTEVEADSGSSVMEAAIRNGIPGIDAECGGACACATCHVYVDDDWAD
TVGGPDPMEEDMLDFAYEVRPTSRLSCQIRVTDDLEGLVVQVPERQN
>2Fe-2SST1(bmw:BMNI_I1000)Brucella melitensis NI
MTKIVFVSADGATRTEVEADSGSSVMEAAIRNGIPGIDAECGGACACATCHVYVDDDWAD
TVGGPDPMEEDMLDFAYEVRPTSRLSCQIRVTDDLEGLVVQVPERQN
>2Fe-2SST1(bmee:DK62_399)Brucella melitensis bv. 3 Ether
MTKIVFVSADGATRTEVEADSGSSVMEAAIRNGIPGIDAECGGACACATCHVYVDDDWAD
TVGGPDPMEEDMLDFAYEVRPTSRLSCQIRVTDDLEGLVVQVPERQN
>2Fe-2SST1(bmc:BAbS19_I09700)Brucella abortus S19
MTKIVFVSADGATRTEVEADSGSSVMEAAIRNGIPGIDAECGGACACATCHVYVDDDWAD
TVGGPDPMEEDMLDFAYEVRPTSRLSCQIRVTDDLEGLVVQVPERQN

>2Fe-2SST1(baa:BAA13334_I02338)Brucella abortus A13334
MTKIVFVSADGATRTEVEADSGSSVMEAAIRNGIPGIDAECGGACACATCHVYVDDDWAD
TVGGPDPMEEDMLDFAYEVRPTSRLSCQIRVTDDLEGLVVQVPERQN
>2Fe-2SST1(babo:DK55_1030)Brucella abortus bv. 2 86/8/59
MTKIVFVSADGATRTEVEADSGSSVMEAAIRNGIPGIDAECGGACACATCHVYVDDDWAD
TVGGPDPMEEDMLDFAYEVRPTSRLSCQIRVTDDLEGLVVQVPERQN
>2Fe-2SST1(babr:DO74_860)Brucella abortus bv. 6 870
MTKIVFVSADGATRTEVEADSGSSVMEAAIRNGIPGIDAECGGACACATCHVYVDDDWAD
TVGGPDPMEEDMLDFAYEVRPTSRLSCQIRVTDDLEGLVVQVPERQN
>2Fe-2SST1(babt:DK49_786)Brucella abortus 63 75
MTKIVFVSADGATRTEVEADSGSSVMEAAIRNGIPGIDAECGGACACATCHVYVDDDWAD
TVGGPDPMEEDMLDFAYEVRPTSRLSCQIRVTDDLEGLVVQVPERQN
>2Fe-2SST1(babb:DK48_1089)Brucella abortus BDW
MTKIVFVSADGATRTEVEADSGSSVMEAAIRNGIPGIDAECGGACACATCHVYVDDDWAD
TVGGPDPMEEDMLDFAYEVRPTSRLSCQIRVTDDLEGLVVQVPERQN
>2Fe-2SST1(babu:DK53_1012)Brucella abortus bv. 9 C68
MTKIVFVSADGATRTEVEADSGSSVMEAAIRNGIPGIDAECGGACACATCHVYVDDDWAD
TVGGPDPMEEDMLDFAYEVRPTSRLSCQIRVTDDLEGLVVQVPERQN
>2Fe-2SST1(babc:DO78_935)Brucella abortus NCTC 10505
MTKIVFVSADGATRTEVEADSGSSVMEAAIRNGIPGIDAECGGACACATCHVYVDDDWAD
TVGGPDPMEEDMLDFAYEVRPTSRLSCQIRVTDDLEGLVVQVPERQN
>2Fe-2SST1(bsuc:BSSP2_I1041)Brucella suis bv. 2 Bs364CITA
MTKIVFVSADGATRTEVEADNGSSVMEAAIRNGIPGIDAECGGACACATCHVYVDDDWAD
TVGGPDPMEEDMLDFAYEVRPTSRLSCQIRVTDDLEGLVVQVPERQN
>2Fe-2SST1(bsz:DK67_1919)Brucella suis bv. 3
MTKIVFVSADGATRTEVEADNGSSVMEAAIRNGIPGIDAECGGACACATCHVYVDDDWAD

TVGGPDPMEEDMLDFAYEVRPTSRLSCQIRVTDDLEGLVVQVPERQN
>2Fe-2SST1(bsw:IY71_05090)Brucella suis ZW043
MTKIVFVSADGATRTEVEADNGSSVMEAAIRNGIPGIDAECGGACACATCHVYVDDDWAD
TVGGPDPMEEDMLDFAYEVRPTSRLSCQIRVTDDLEGLVVQVPERQN
>2Fe-2SST1(bsg:IY72_04830)Brucella suis ZW046
MTKIVFVSADGATRTEVEADNGSSVMEAAIRNGIPGIDAECGGACACATCHVYVDDDWAD
TVGGPDPMEEDMLDFAYEVRPTSRLSCQIRVTDDLEGLVVQVPERQN
>2Fe-2SST1(bcs:BCAN_A1040)Brucella canis ATCC 23365
MTKIVFVSADGATRTEVEADNGSSVMEAAIRNGIPGIDAECGGACACATCHVYVDDDWAD
TVGGPDPMEEDMLDFAYEVRPTSRLSCQIRVTDDLEGLVVQVPERQN
>2Fe-2SST1(bsk:BCA52141_I0089)Brucella canis HSK A52141
MTKIVFVSADGATRTEVEADNGSSVMEAAIRNGIPGIDAECGGACACATCHVYVDDDWAD
TVGGPDPMEEDMLDFAYEVRPTSRLSCQIRVTDDLEGLVVQVPERQN
>2Fe-2SST1(bcar:DK60_1077)Brucella canis RM6/66
MTKIVFVSADGATRTEVEADNGSSVMEAAIRNGIPGIDAECGGACACATCHVYVDDDWAD
TVGGPDPMEEDMLDFAYEVRPTSRLSCQIRVTDDLEGLVVQVPERQN
>2Fe-2SST1(bcas:DA85_04885)Brucella canis SVA13
MTKIVFVSADGATRTEVEADNGSSVMEAAIRNGIPGIDAECGGACACATCHVYVDDDWAD
TVGGPDPMEEDMLDFAYEVRPTSRLSCQIRVTDDLEGLVVQVPERQN
>2Fe-2SST1(bpv:DK65_352)Brucella pinnipedialis 6/566
MTKIVFVSADGATRTEVEADNGSSVMEAAIRNGIPGIDAECGGACACATCHVYVDDDWAD
TVGGPDPMEEDMLDFAYEVRPTSRLSCQIRVTDDLEGLVVQVPERQN
>2Fe-2SST1(bcet:V910_100966)Brucella ceti TE10759-12
MTKIVFVSADGATRTEVEADNGSSVMEAAIRNGIPGIDAECGGACACATCHVYVDDDWAD
TVGGPDPMEEDMLDFAYEVRPTSRLSCQIRVTDDLEGLVVQVPERQN
>2Fe-2SST1(oan:Oant_2112)Ochrobactrum anthropi ATCC 49188

MTKIVFVSADGATRTEVEADNGSSVMEAAIRNGIPGIDAECGGACACATCHVYVDEDWTD

TVGGPDAMEEDMLDFAYEVRPTSRLSCQIRVSDDIDGLVVQVPERQN

>2Fe-2SST1(oah:DR92_1606)Ochrobactrum anthropi OAB

MTKIVFVSADGATRTEVEADNGSSVMEAAIRNGIPGIDAECGGACACATCHVYVDEDWTD

TVGGPDAMEEDMLDFAYEVRPTSRLSCQIRVSDDIDGLVVQVPERQN

>2Fe-2SST1(ops:A8A54_04740)Ochrobactrum pseudogrignonense

MTKIIFVSADGASRTEVEADNGSSVMEAAIRNGIPGIDAECGGACACATCHVYVDEDWTE

TVGGPDAMEEDMLDFAYEVQPNSRLSCQIRVSDEIDGLVVKVPERQN

>2Fe-2SST1(bja:bll5885)Bradyrhizobium diazoefficiens USDA 110

MAKIHFVDHKGETRTVEIENGATVMEAAIRNSIPGIEAECGGACACATCHVYVDEAWREK

VGSPTPMEEDMLDFGFDVRPNSRLSCQIKVSDELDGLVVSTPERQA

>2Fe-2SST1(bju:BJ6T_38260)Bradyrhizobium japonicum USDA 6

MAKINFVDHKGETRTVEVENGATVMEAAIRNSIPGIEAECGGACACATCHVYVDEAWREK

VGSPTPMEEDMLDFGFDVRPNSRLSCQIKVSDELDGLVVATPERQA

>2Fe-2SST1(bjp:RN69_18555)Bradyrhizobium japonicum E109

MAKINFVDHKGETRTVEVENGATVMEAAIRNSIPGIEAECGGACACATCHVYVDEAWREK

VGSPTPMEEDMLDFGFDVRPNSRLSCQIKVSDELDGLVVATPERQA

>2Fe-2SST1(bra:BRADO5081)Bradyrhizobium sp. ORS 278

MAKITFVDHTGESRTVDIENGATVMEAAIRNAIPGIEAECGGACACATCHVYVDEAWREK

VGPPTPMEEDMLDFGYDVRPNSRLACQIKVSDELDGLVVATPERQA

>2Fe-2SST1(bbt:BBta_5552)Bradyrhizobium sp. BTAi1

MAKITFVDHTGESRTVEIENGATVMEAAIRNAIPGIEAECGGACACATCHVYVDEAWREK

VGPPTPMEEDMLDFGYEVRPNSRLSCQIKVSDELDGLVVATPERQA

>2Fe-2SST1(brs:S23_23680)Bradyrhizobium sp. S23321

MAKIHFVDHKGETRTVEIENGATVMEAAIRNSIPGIEAECGGACACATCHVYVDEAWREK

VGSPTPMEEDMLDFGFDVRPNSRLSCQIKVSDELDGLVVATPERQA

>2Fe-2SST1(aol:S58_26040)Bradyrhizobium oligotrophicum

MAKITFVDHTGESRTVDVENGATVMEAAIRNAIPGIEAECGGACACATCHVYVDEAWREK

VGPPTPMEEDMLDFGYDVRPNSRLSCQIKVSDELNGLVVSTPERQA

>2Fe-2SST1(brc:BCCGELA001_25050)Bradyrhizobium sp. CCGE-LA001

MAKIHFVDHKGETRTVEIENGATVMEAAIRNSIPGIEAECGGACACATCHVYVDEAWREK

VGSPTPMEEDMLDFGFDVRPNSRLSCQIKISDELDGLVVATPERQA

>2Fe-2SST1(brad:BF49_5756)Bradyrhizobium sp. BF49

MAKIHFVDHKGETRTVDVENGATVMEAAIRNSIPGIEAECGGACACATCHVYVDEAWREK

VGSPTPMEEDMLDFGFDVRPNSRLSCQIKVSDDLDGLVVATPERQA

>2Fe-2SST1(bic:LMTR13_28045)Bradyrhizobium icense

MAKIHFVDHNGEKRTIEVENGATVMEAAIRNAIPGIEAECGGACACATCHVYVDEAWREK

VGAPTPMEEDMLDFGFDVRPNSRLSCQIKVSDDLDGLVVSTPERQA

>2Fe-2SST1(rpa:RPA3956)Rhodopseudomonas palustris CGA009

MAKINFVDHTGETRTVEVEEGATVMEAAIRNAIPGVEAECGGACACATCHVYVDEAWREK

VGGPSPMEEDMLDFGYDVRPNSRLSCQIKVSNELDGLIVTTPERQA

>2Fe-2SST1(rpb:RPB_3843)Rhodopseudomonas palustris HaA2

MAKIHFVDHSGETRIVDVENGATVMEAAIRNAIPGIEAECGGACACATCHVYVDEAWREK

VGGPSPMEEDMLDFGYDVRPNSRLSCQIKVSDELDGLVISTPDRQA

>2Fe-2SST1(rpc:RPC_1457)Rhodopseudomonas palustris BisB18

MAKINFVDHSGETRSVEVEEGATVMEAAIRNAVPGIEAECGGACACATCHVYVDEAWRDK

TGAPTPMEEDMLDFGFDVRPNSRLSCQIKVTEELDGLVVTVPERQA

>2Fe-2SST1(rpd:RPD_1643)Rhodopseudomonas palustris BisB5

MAKIHFVDHSGETRIVDVENGATVMEAAIRNAIPGIEAECGGACACATCHVYVDEAWREK

VGGPSPMEEDMLDFGYDVRPSSRLSCQIKVSDELDGLVISTPDRQA

>2Fe-2SST1(rpe:RPE_1476)Rhodopseudomonas palustris BisA53

MAKINFVDHKGETRTVEVEEGATVMEAAIRNAVPGIEAECGGACACATCHVYVDEAWRDK

TGAPTPMEEDMLDFGYDVRPNSRLSCQIKVTDALDGLVVSIPERQA

>2Fe-2SST1(rpt:Rpal_4477)Rhodopseudomonas palustris TIE-1

MAKINFVDHTGETRTVEVEEGATVMEAAIRNAIPGVEAECGGACACATCHVYVDEAWREK

VGGPSPMEEDMLDFGYDVRPNSRLSCQIKVSNELDGLIVTTPERQA

>2Fe-2SST1(rpx:Rpdx1_1396)Rhodopseudomonas palustris DX-1

MAKIHFVDHTGETRTVEVEEGATVMEAAIRNAIPGVEAECGGACACATCHVYVDEAWREK

VGGPSPMEEDMLDFGYDVRPNSRLSCQIKVSNELDGLIVTTPERQA

>2Fe-2SST1(nwi:Nwi_1088)Nitrobacter winogradskyi

MTKINFVDHSGETRTVDVENGATVMEAAIRNAVPGIEAECGGACACATCHVYIDDAWTEK

VGKPTPMEEDMLDFGYDVRPNSRLSCQIKVSDELEGLVVTVPERQA

>2Fe-2SST1(nha:Nham_1320)Nitrobacter hamburgensis

MTKINFVDHSGETRTVDVENGATVMEAAIRNAVPGIEAECGGACACATCHVYIDEAWSDK

VGKPTPMEEDMLDFGYDVRPNSRLSCQIKVSDELDGLVVSTPERQA

>2Fe-2SST1(bos:BSY19_1779)Bosea sp. RAC05

MPQVTFIDAHGESRTVEGEAGATVMEVAIRNGVPGIEAECGGACACATCHVYVDEAWTAK

TGRPEPMEEDMLDFAFEVKPTSRLSCQIKLRDDLDGLVVRTPSRQG

>2Fe-2SST1(bvv:BHK69_12405)Bosea vaviloviae

MPKITYIDAQGESRTVEGETGSTVMEVAVRNAIPGIEAECGGACACATCHVYVDDAWAAK

TGHPESMEEDMLDFAFDVRPTSRLSCQIRVKDDLDGLIVRTPARQG

>2Fe-2SST1(xau:Xaut_4124)Xanthobacter autotrophicus

MVAITFIEYNGTAHQVEGPEGATAMETAVRNGVPGIVAECGGACACATCHVYVDEAWADK

VGEPSEMEEGMLDFATDVRPSSRLSCQIKLTAALDGLVLNIPETQG

>2Fe-2SST1(xau:Xaut_2914)Xanthobacter autotrophicus

MARFTVQTRNGDQMEVTGRDGATLLKAMRKAGVEEVVAQCGGGCACATCHVYVTPPEGGA

LPPVGPAEARMLATSRYRTLNSRLSCQLKLEPGLEQMLVVIAPDDGGDF

>2Fe-2SST1(azc:AZC_2219)Azorhizobium caulinodans

MPAITFVSFEGASRTVQAPVGATAMEVAVRNGVPGIDADCGGACACATCHVYVDEAWRAA
VGEPEGMEEDMLDFATDVRPSSRLSCQIKITEALDGLVLHTPERQG
>2Fe-2SST1(mex:Mext_4466)Methylorubrum extorquens PA1
MPKITFVDHAGTARTIDGEVGSTVMETAIRNNVPGIDAECGGACACATCHVYVDGEWADK
VGPAEPMEQDMLDFASDVRATSRLCCQIRVTPELDGLIVTTPARQG
>2Fe-2SST1(mea:Mex_1p4911)Methylorubrum extorquens AM1
MPKITFVDHAGTARTIDGEVGSTVMETAIRNNVPGIDAECGGACACATCHVYVDGEWADK
VGPAEPMEQDMLDFASDVRATSRLCCQIRVTPELDGLIVTTPARQG
>2Fe-2SST1(mdi:METDI5502)Methylorubrum extorquens DM4
MPKITFVDHAGTARTIDGEVGSTVMETAIRNNVPGIDAECGGACACATCHVYVDGEWADK
VGPAEPMEQDMLDFASDVRATSRLCCQIRVTPELDGLIVTTPARQG
>2Fe-2SST1(mch:Mchl_4930)Methylorubrum extorquens CM4
MPKITFVDHAGAARTIDGEVGSTVMETAIRNNVPGIDAECGGACACATCHVYVDGEWADK
VGPAEPMEQDMLDFASDVRATSRLCCQIRVTAELDGLIVTTPARQG
>2Fe-2SST1(mpo:Mpop_4980)Methylorubrum populi
MPKITFVDHAGTARTIDGEVGSTVMETAIRNNVPGIDAECGGACACATCHVYVDEAWADK
VGPAEPMEQDMLDFASDVRATSRLCCQIRVTPELDGLVVTTPARQG
>2Fe-2SST1(mrd:Mrad2831_1946)Methylobacterium radiotolerans
MPKITYVDHAGTERTVEGSVGATVMETAMRNNVPGIDAECGGACACATCHVYVADAWIDT
VGKAQDMEQDMLDFASDVRENSRLSCQIKITPALDGLVVTTPPQQG
>2Fe-2SST1(met:M446_6065)Methylobacterium sp. 4-46
MVQITYVDAAGTPRTVAGEVGSTVMETAIRNNVPGIDAECGGACACATCHVYVDPEWEAA
VGPAEPMEQDMLDFASDVRPNSRLSCQIRVRPELDGLKVTTPARQG
>2Fe-2SST1(mno:Mnod_6602)Methylobacterium nodulans
MVQITYVDAAGTPRTVEGAVGSTVMETAIRNNVPGIDAECGGACACATCHVYVDPEWAEA
VGPAEPMEQDMLDFASDVRPNSRLSCQIRLKPELDGLTVTTPTRQG

>2Fe-2SST1(mor:MOC_2358)Methylobacterium oryzae

MPKITYVDHAGTERTVEGSVGATVMETALRNNVPGIDAECGGACACATCHVYVADAWIEV

VGKAQDMEQDMLDFASDVRENSRLSCQIKITPALDGLVVTTPSQQG

>2Fe-2SST1(meta:Y590_22775)Methylobacterium sp. AMS5

MPKITFVDHAGTARTIDGEVGSTVMETAIRNNVPGIDAECGGACACATCHVYVDGEWADK

VGPAEPMEQDMLDFASDVRATSRLCCQIRVTPELDGLIVTTPARQG

>2Fe-2SST1(maqu:Maq22A_c09135)Methylobacterium aquaticum

MVQITYVDAAGTARSVKGEVGSTVMETAIRNNVPGIDAECGGACACATCHVYVGEDWAEA

VGPAEPMEQDMLDFASDVRPTSRLSCQIRLKPELDGLTVTTPARQG

>2Fe-2SST1(bid:Bind_0539)Beijerinckia indica

MTKMTFITFDGTSIPVEAENGEILMRVGQRCEIPGILGECGGTCSCGSCHVYIDESWRDR

VPPVKELEQMMIDSLMTKQDNSRLTCMILVAPELDGLILHLPNPNL

>2Fe-2SST1(bid:Bind_2346)Beijerinckia indica

MVKITFIDSFGQHRTVEAEEGTTVMENAIRNGIPEIVAECGGGCACATCHVYVDEAFLEK

TGKPSEKEEDMLDFAYKVQPNSRLSCQIKVRPELDGMTVTTPDRQG

>2Fe-2SST1(msl:Msil_3549)Methylocella silvestris

MPKITFVDSLGEARTVDAEVGSTVMEAAIRNAIPEIAAECGGGCACATCHVYVDDPWGKL

TGKASDQEEDMLDFAYAVQPNSRLSCQITVTEELDGLHVTTPDRQG

>2Fe-2SST1(hdn:Hden_1632)Hyphomicrobium denitrificans ATCC 51888

MTKITFIQPDGEGQTVEVENGLTVMEAAKLNDVAGIEAECGGACACATCHVYVDKAWREK

AGKPSEMEEDMLDFAFDVREESRLSCQIKISDALDGLIVRVPEKQF

>2Fe-2SST1(hdt:HYPDE_30043)Hyphomicrobium denitrificans 1NES1

MTKITFIQPDGESQTVEAENGLTVMEAAKLNDIAGIEAECGGACACATCHVYVDEAWREK

TGKPSEMEEDMLDFAFDVREESRLSCQIKISDALDGLVVRVPEKQF

>2Fe-2SST1(hmc:HYPMC_1912)Hyphomicrobium sp. MC1

MTKITFIQPDGESQTVEAENGLTVMEAAKLNDIAGIEAECGGACACATCHVYVDDAWRDK

VGKASDMEEDMLDFAFDVREGSRLSCQIKISDAIDGLVVRVPAKQF
>2Fe-2SST1(hni:W911_11090)Hyphomicrobium nitrativorans
MVKLTFIQPDGASQTVEADAGATVMEAAKQNDVPGIDAECGGACACATCHVYVDAAWVDK
VGTPDPMEEDMLDFAFDVRKESRLSCQIKITDALDGITFRVPEKQF
>2Fe-2SST1(phl:KKY_1900)Pelagibacterium halotolerans
MITFVEPDGARREVEAENGATLMETAIRNGVRGIVAECGGACTCATCHVYVEEEWFGVTG
GPSSMEEDMLDFAFDVKDTSRLSCQIKIRDDLDGLVVNVPSRQG
>2Fe-2SST1(fil:BN1229_v1_2936)Candidatus Filomicrobium marinum W
MAKITFIQPDGSTQVVEATPGTTVMEAAKLNGVPGIEAECGGACACATCHVYVSDDWREQ
VGAPSEMEEDMLDFAFDVRESSRLSCQIKVTDALDGLVLSVPAKQF
>2Fe-2SST1(fiy:BN1229_v1_2981)Candidatus Filomicrobium marinum Y
MAKITFIQPDGSTQVVEATPGTTVMEAAKLNGVPGIEAECGGACACATCHVYVSDDWREQ
VGAPSEMEEDMLDFAFDVRESSRLSCQIKVTDALDGLVLSVPAKQF
>2Fe-2SST1(deq:XM25_10565)Devosia sp. H5989
MTKITYVEPDGSRIEVEAENGSTVMENAIMNGVPGIVAECGGACTCATCHVYVDDAWTET
VGGPSVMEEDMLDFAFDVRENSRLSCQIKVRDELDGLVVNVPTRQG
>2Fe-2SST1(rhz:RHPLAN_49350)Rhodoplanes sp. Z2-YC6860
MPKITFIDFAGSSRTIDADVGATVMETAIKNDVPGIEAECGGACACATCHVYVDEAWREK
VGGPTPMEEDMLDFGYDVKPNSRLSCQIKVTDALDGLVVRTPERQA
>2Fe-2SST1(rhz:RHPLAN_48480)Rhodoplanes sp. Z2-YC6860
MPKITYIEQDGTERTVEAEAGSTVMETAINNDIPGILATCGGSCSCATCHVYVDEDWVDK
LPPPELDENDMLDTAHDLQENSRLSCQIKVTDELDGLIVTTPARQI
>2Fe-2SST1(msc:BN69_2605)Methylocystis sp. SC2
MAKFTLETRDGARQDIAGKNGVSLMKLIRRAGVEELVAQCGGSCACATCHVYLTLPQGIE
MASSGPGESRMLATASHRKFNSRLACQIRFDDILDGMEIRIAPEDATGF
>2Fe-2SST1(mbry:B1812_15995)Methylocystis bryophila

MTFVDSKGQARTVEGEVGSTVMETARRNDIPEIAAECGGACACATCHVYVDAAWLEKTGA
RSQMEEDMLDFAFAVEQNSRLCCQIVVKPELEGLILRTPAQQG
>2Fe-2SST1(mbry:B1812_21155)Methylocystis bryophila
MASFTLETREGARSEVKAKDGVTLMKLIRRSGAEELVAQCGGSCACATCHVYVSPREGVT
LPPMRPDESRMLATAGERRMTSRLACQIKFDAALDGMHVTIAPENLDDI
>2Fe-2SST1(mey:TM49_15150)Martelella endophytica
MLKLKFIGNDETVYDLEVSPGSSVMENAVRNSVPGIEAECGGACACATCHVYVDPDWMAK
VGEPEPMEEDMLDFAIDVRANSRLSCQIEMRDELDGLTVRIPERQG
>2Fe-2SST1(mcg:GL4_1666)Methyloceanibacter caenitepidi
MLKVTFIQPDGIENTVEAEPGMTLMEAAVKNSVRGIAAECGGACSCATCHVYVEEAWREA
TGEPEAMEEDMLDFAFDVRPSSRLSCQIRLTEELDGLTVRIPEKQF
>2Fe-2SST1(psin:CAK95_17970)Pseudorhodoplanes sinuspersici
MSKITFIEHDGTVHEVEAETGETVMEAAMRGGVSGIVAECGGSCTCATCHVYVDEAWLDK
TGERSPDEEDQLDNAYDVRANSRLSCQIKISEELDGLLVRTPSYQGR
>2Fe-2SST1(psin:CAK95_17965)Pseudorhodoplanes sinuspersici
MAKITFIDHGGESRTVDAENGATVMETAIKNGIPGIEAECGGACACATCHVYVDEAWTEV
VGAPSPMEEDMLDFGFDVKPNSRLSCQIKVTDQLDGLVLRTPERQA
>2Fe-2SST1(ccr:CC_3524)Caulobacter vibrioides CB15
MAKITYIQHDGAEQVIDVKPGLTVMEGAVKNNVPGIDADCGGACACATCHVYVDEAWLDK
TGDKSAMEESMLDFAENVEPNSRLSCQIKVSDALDGLVVRLPESQH
>2Fe-2SST1(ccs:CCNA_03639)Caulobacter vibrioides NA1000
MAKITYIQHDGAEQVIDVKPGLTVMEGAVKNNVPGIDADCGGACACATCHVYVDEAWLDK
TGDKSAMEESMLDFAENVEPNSRLSCQIKVSDALDGLVVRLPESQH
>2Fe-2SST1(cak:Caul_4835)Caulobacter sp. K31
MAKITYIEHDGTDHVIDVKPGLTVMEGAVKNNVPGIDADCGGACACATCHVYVDEAWLPK
TGEKSAMEESMLDFAENVEPNSRLSCQIKVSDALDGLVVRLPESQH

>2Fe-2SST1(cse:Cseg_0160)Caulobacter segnis
MAKITYIEHDGTEHALDVKPGLTVMEGAVKNNVPGIDADCGGACACATCHVYVDEAWLDK
TGDKSAMEESMLDFAENVEPNSRLSCQIKVTDALDGLVVRLPESQH
>2Fe-2SST1(cse:Cseg_3028)Caulobacter segnis
MPKITFIQHDGATLVVEGRPGQSLMEAAVQNNVPGIDADCGGACACGTCQVHVQAPWQEA
LTLPSAMEAEMLSFTSDPQPSSRLACQIILEERHEGLMVRVPAHQH
>2Fe-2SST1(aex:Astex_0212)Asticcacaulis excentricus
MPKITYIESNGKTHEIEVKTGNSVMEGAIKHNIPGIDADCGGACACATCHVYVDAEWFDK
TGGPSVMEESMLDFAQDVEPTSRLSCQIRVTEALDGLIVRLPESQH
>2Fe-2SST1(psf:PSE_4116)Pseudovibrio sp. FO-BEG1
MPKITFITSSGESHEVDAAAGSTVMENAIKNMVPGIEAECGGACACATCHVYVDPAWNDK
TGEPEPMEEDMLDFAQDVRDTSRLSCQIRVTDEMDGLVVHIPESQS
>2Fe-2SST1(con:TQ29_05630)Confluentimicrobium sp. EMB200-NS6
MAKIIFVDHDGTKHEVEASVGSNVMQAAVDNNIEGIDGDCGGMCACATCHVTVDPEWFAK
TGARSENEESMLTFLPEIAETARLGCQIEITDELDGLVVHLPIEQF
>2Fe-2SST1(con:TQ29_19120)Confluentimicrobium sp. EMB200-NS6
MSIQVVFIDSMGERREVSSREGQSLMEAATMAGVPGIDADCGGACACATCQVYVDPKWLS
LLPPTSEGEASMLEFAANRQDSSRLACQIRLTADLDGLTVTTPEFQF
>2Fe-2SST1(pphr:APZ00_14900)Pannonibacter phragmitetus
MPKITFITADGARHDVEAASGATVMESAIKHMVPGIEAECGGACACATCHVYVDEEWSAR
AGSPDPMEEDMLDFAYDVKPNSRLSCQIKMSDELDGIVVHVPERQA
>2Fe-2SST1(pphr:APZ00_07260)Pannonibacter phragmitetus
MPRIIFQSADGTRTEVEAASGQSLMSAAVAHGIEEIAAECGGACACGTCHCYIPEGQGIT
LPEAEEMELAMIECVIDPRPQSRLTCQVTVSEAMDGLVVELPASQH
>2Fe-2SST1(lap:ACP90_10170)Labrenzia sp. CP4
MPKITFVTADGTRTDVDAAEGSTVMENAIKNMVQGIEAECGGACACATCHVYVDDAWSGK

TGSPEPMEEDMLDFAYDVKPTSRLSCQIKVTSDLDGLVVHVPERQA

>2Fe-2SST1(lagg:B0E33_22090)Labrenzia aggregata

MPKITFVTADGTRTDVDAAEGSTVMENAIKNMVQGIEAECGGACACATCHVYVDDAWSGK

TGSPEPMEEDMLDFAYDVKPTSRLSCQIKVTSDLDGLVVHVPERQA

>2Fe-2SST1(mmr:Mmar10_0081)Maricaulis maris

MPKITYIEHNGTEHVIDVATGLTVMEGAVRNLVPGIDADCGGACACATCHVYVDPAWADK

TGSREAMEDSMLDFAEEVQETSRLSCQIKVSDELDGLVVRMPQNQG

>2Fe-2SST1(hne:HNE_1889)Hyphomonas neptunium

MAKITYVSHDGTERTVEAKNGESVMEAAIKNSIPGIDADCGGACACATCHVYVDEAFLDK

AGTQEEMEKSMLDFAENVKPNSRLSCQIKVSDALDGLRVSTPESQH

>2Fe-2SST1(ba:Hbal_2335)Hirschia baltica

MAKITYIEHDGTEHSVDAKNGSSVMEAAIRENVPGIDADCGGACACATCHVYVDASFMDK

VGAPNDMEQSMLDFAEGVESNSRLSCQITVKDDLDGLIVRMPESQT

>2Fe-2SST1(hbc:AEM38_00300)Hyphomonadaceae bacterium UKL13-1

MAKVTYIEHSGTEHVIEVPVGTTIMEGAVKNRVPGIDADCGGACACATCHVFVDAAWIEK

TGKPGSMEESMLDFAEGVTPLSRLSCQISMTSDLDGIVVRLPAAQH

>2Fe-2SST1(smaz:LH19_03020)Sphingopyxis macrogoltabida 203

MPSVNFHAADGSVETIEIEVGDSVMRGARDNMVEGIEADCGGVCACATCHIYIAPDWLER

VGPAGADESEMLDCVNDPRPNSRLSCQIAMTDDLEGLTVFLPESQR

>2Fe-2SST1(swi:Swit_0285)Sphingomonas wittichii

MMTEIRFIDADGAVTIARGDNGFSLMEVAKRHGVSGIVAECGGSCACATCHVHVDAAWLE

AVGEPNPGEADMLDFARGRRPDSRLSCQIRITPALDGLIVHVPESQG

>2Fe-2SST1(sch:Sphch_3064)Sphingobium chlorophenolicum

MANLIVVDRSGQEHVIEAANGWSVMEAIRDNGIDELLALCGGCCSCATCHVYVDETQNAR

FAAMTDDENDLLDSSDYRKTNSRLSCQLIVDGTPEIIRLSIAPED

>2Fe-2SST1(aep:AMC99_00144)Altererythrobacter epoxidivorans

MVLVSYVADNGTVHEVAASVGISLMEAAAMNDVPGIEGDCGGFCACGTCHCYVDESDAAI

LPDMHELERATLDFAYDVTERSRLACQIPITEQMDGIRVRLPARQY

>2Fe-2SST1(azl:AZL_b01710)Azospirillum sp. B510

MPTITFIQANGTEHTVTFDPGKTLMQAAVDNLVPGIQADCGGYCNCATCHCFVEPPWEAT

LPAPEQPEQDMLTCAIDPQPNSRLSCQIVLTEAMSGLVVRLPVSQT

>2Fe-2SST1(pgv:SL003B_1500)Polymorphum gilvum

MPHITYITADGSRHEVDAAEGTTVMENAIKNMVPGIEAECGGACACATCHVYVDEAWAER

VGHPEPMEEDMLDFAYDVRPTSRLSCQIKVTAALDGLVVHVPERQA

>2Fe-2SST1(pgv:SL003B_4084)Polymorphum gilvum

MTINLVFIDSSGARREITSPEGQSLMETATMAGIPGIDADCGGACACATCQVYVAEEWVG

KLPPIAEAEANMLEFAANRQANSRLACQIRLTPELDGLTVTTPEFQF

>2Fe-2SST1(pgv:SL003B_4103)Polymorphum gilvum

MAINLVFIDSTGERREISAAEGRTLMEAACLAGIPGIDADCGGACACATCQVYVDAEWQS

RLPPVGNPEANMLNFAANRRENSRLACQIRLTAEMDGLIVSTPEFQF

>2Fe-2SST1(gdi:GDI2370)Gluconacetobacter diazotrophicus PA1 5 (Brazil)

MIFIESDGTRREVDAPVGLSVLEIAHKHGVDLEGACEGSLACATCHVVVDPDWAPKLAAP

TEDEEDMLDLAFGLEKTSRLGCQIVMTEALDGLVVRLPHKA

>2Fe-2SST1(aace:A0U92_16090)Acetobacter aceti

MPHMIFVEPDGTERKVDAPVGLSVLEIAHKHGVDLEGACEGSLACATCHVIVDPSWAPKL

SEATEDEEDMLDLAFGLEATSRLGCQIIMNDELDGLTVRLPRKS

>2Fe-2SST1(apb:SAR116_1022)Candidatus Puniceispirillum marinum

MPNIIFVKPDGTELNVNVNEGVSVMEAGRDANLGIEGTCGGCLSCATCHVIVDADWFAKT

GAPSEDEEDMLDLAFGLTETSRLGCQLTMSAELDGIRLTIPDDM

>2Fe-2SST1(rtr:RTCIAT899_PC02805)Rhizobium tropici

MCEIVFIDSAGRETRVEAQEGETLMAVAVRSGIDGIVAQCGGALACGTCHCYIEQPHLDR

LPPPSEEEAMMIEFVMEPMPNSRLSCQILASSVVDGMRVIVPDSQH

>2Fe-2SST1(gdj:Gdia_0615)Gluconacetobacter diazotrophicus PA1 5 (JGI)

MPHMIFIESDGTRREVDAPVGLSVLEIAHKHGVDLEGACEGSLACATCHVVVDPDWAPKL

AAPTEDEEDMLDLAFGLEKTSRLGCQIVMTEALDGLVVRLPHKA

>2Fe-2SST1(mgy:MGMSRv2__1381)Magnetospirillum gryphiswaldense MSR-1 v2

MPKMTFITADGSRNEVEAPEGLSVLEIAHRNKIDLEGACEGSLACSTCHIVVDPDWYERL

AAAEEDEEDMLDLAFGLTSTSRLGCQIIMKQELDGLVVTVPAATRNMSVDKK

>2Fe-2SST1(magq:MGMAQ_2629)Magnetospira sp. QH-2

MPKMTFIEPNGTRREVDAPLGQSVLEIAHHNKIDLEGACEGSLACSTCHVIVDQEWYDKL

EEASEDEEDMLDLAFALTHTSRLGCQIVISEELDGLVVNLPAATRNVAVDKK

>2Fe-2SST1(azl:AZL_020110)Azospirillum sp. B510

MPKMTFIETDGTRREVDAPLGLSVLEIAHKNSLDLEGACEGSLACSTCHVVIEPEWFDVL

PEAQEDEEDMLDLAFGLTKTSRLGCQIIMTEELDGLVVRLPGGSNNAMK

>2Fe-2SST1(abq:ABAZ39_09740)Azospirillum brasilense Az39

MPKMTFIEPNGTRHEVDAPLGLSVLEVAHKHGLDLEGACEGSLACSTCHVIVEPEWFDVL

NEASEDEEDMLDLAFGLTKTSRLGCQIIISEELDGLAVRLPGGTNNAMR

>2Fe-2SST1(sphl:LPB140_08735)Sphingopyxis sp. LPB0140

MVKVIFTNISGSVIQEVEGNEGDCLLDVGQAAGQPLEGTCEGQMACSTCHVIVKDAWFDK

LPPASENEEDMLDLAAGARRTSRLSCQILLTKDLDGLTVAIPSESRNMQGL

>2Fe-2SST1(sphg:AZE99_06970)Sphingorhabdus sp. M41

MKKITVHFVSSDGEKTVTAEASEGDNLLTVAQIHDQPLEGTCEGQMACSTCHVIVESADF

DRLPEATEMEEDMLDLAAGARRTSRLACQITLTADLDGLTVHMPVESHNMQIV

>2Fe-2SST1(blas:BSY18_949)Blastomonas sp. RAC04

MIEVTFISADGKSRQTVQAQDGDRLLDVAQAHGLPLEGTCEGQMACSTCHLIIDAADFAK

LPEPVEMEEDMLDLAVAVTRTSRLSCQIFLRDEWQSLTCRVPAESYDMQGM

>2Fe-2SST1(sphi:TS85_09595)Sphingomonas sp. WHSC-8

MIRVRFVSADGARLREVEGGEGDRLLELAQNDGQPLEGTCEGQMSCSTCHVIVADEDFER
LPRASEEEEDMLDLAVGATRTSRLACQILLTPDLDGLTVRMPPGHRDMQGR
>2Fe-2SST1(sgi:SGRAN_2433)Sphingopyxis granuli
MRVTFIHADGKGRTEAEAVPGDILLDVAQAHLMPLEGTCEGQMACSTCHVIVAREDFDRL
PPASEEEEDMLDLAAGARRTSRLSCQVVLTEALDGLTVHIPGESRNMQGAR
>2Fe-2SST1(sal:Sala_0795)Sphingopyxis alaskensis
MRVTFIHADGKGRTEAEAEPGSTLLDVAQAHLIPLEGTCEGQMACSTCHVVVESQDFDRL
PPASEMEEDMLDLAAGARRTSRLSCQIVLTEDMDGLTVRIPAESRNMQGVR
>2Fe-2SST1(sphk:SKP52_07080)Sphingopyxis fribergensis
MRVTFIHADGKARTEAEAEPGSILLDIAQAHMMPLEGTCEGQMACSTCHVIVAKEDFDRL
PPASEMEEDMLDLAAGVRRTSRLSCQIVLTEALAGLTVHIPAESRNMQGPR
>2Fe-2SST1(sphp:LH20_07565)Sphingopyxis sp. 113P3
MRVTFIHADGKRRTEAEAAPGDILLDVAQAHMMPLEGTCEGQMACSTCHVIVAKEDFGRL
PPASEEEEDMLDLAAGVRRTSRLSCQIVLTEDLDGLTVHIPAESRNMQGPR
>2Fe-2SST1(smaz:LH19_10545)Sphingopyxis macrogoltabida 203
MRVTFIHADGKQRTEAEAEPGSILLDVAQAHMMPLEGTCEGQMACSTCHVIVAKEDFDRL
PEASEMEEDMLDLAAGVRRTSRLACQIVLTDALDGLTVHIPAESRNMQGPR
>2Fe-2SST1(ster:AOA14_14170)Sphingopyxis terrae
MRVTFIHADGKGRTEVVAEPGSILLDVAQAHMMPLEGTCEGQMACSTCHVIVAKEDFGRL
PPASEMEEDMLDLAAGVRRTSRLSCQIVLTEDLDGLTVHIPSESRNMQGPR
>2Fe-2SST1(sphm:G432_03420)Sphingomonas sp. MM-1
MGGMTTVRFVSADGSHIDEVEAPAGARLLDVAQANGQPLEGTCEGQMACSTCHVIVDRED
FPKLDEATEMEEDLLDLAASVTRWSRLACQVYLPEGLESLTVRIPGESRNMQGR
>2Fe-2SST1(swi:Swit_0053)Sphingomonas wittichii
MTRVVFISADGEHRSEVEASPGQHLLDVAQADGQPLEGTCEGQMACSTCHVIVDAADFAR
LPRASEMEEDLLDLASHVTRTSRLACQIVLTDALDGLTVRMPVGARNMQGR

>2Fe-2SST1(ssy:SLG_13390)Sphingobium sp. SYK-6

MTRITFISANGADVMEVEAAPGDTLLDIAQAAGQPLEGTCEGQMACSTCHVIIDPADFPK

LPRASEEEEDMLDLAAATTRTSRLACQIVLSEAMESLTVRIPPESRNMQGL

>2Fe-2SST1(sbd:ATN00_13340)Sphingobium baderi

MTRVTFISADGESRQEVDAPAGSVLLNVAQAAGQPLEGTCEGQMACSTCHVIVDAADFSR

LPRATEEEEDMLDLAASATRTSRLSCQIVLDAALESLTVRIPPETYNMQGM

>2Fe-2SST1(spmi:K663_07205)Sphingobium sp. MI1205

MIRVTFISADGENRQEVDADVGSVLLDVAQAAGQPLEGTCEGQMACSTCHVIVEAQDFPR

LKAASEAEEDMLDLAAAATRTSRLSCQIVLDDKLDGLTVRIPSESCNMQGM

>2Fe-2SST1(sphb:EP837_00295)Sphingobium sp. EP60837

MIRVTFISADGENRQEVDAAAGSVLLDVAQAAGQPLEGTCEGQMACSTCHVIVEAGDFSK

LKAASEAEEDMLDLAAAATRTSRLSCQIVLEEKLDGLTVRIPGESHNMQGM

>2Fe-2SST1(sphr:BSY17_609)Sphingobium sp. RAC03

MTRVTFISADGERRQEVDAPAGSVLLDIAQAAGQPLEGTCEGQMACSTCHVIVEATDFPR

LGRASEDEEDMLDLAASATRTSRLSCQIILADGLDQLTVRIPPESYNMQGM

>2Fe-2SST1(sjp:SJA_C1-21730)Sphingobium japonicum

MTRVVFISADGEHKQEVEAPPGAVLLDVAQAAGQPLEGTCEGQMACSTCHVIVDAADFSK

LGKASEDEEDMLDLAAAATRTSRLSCQIVLDEQLDSLTVRIPGESYNMQGM

>2Fe-2SST1(sch:Sphch_0633)Sphingobium chlorophenolicum

MTRVIFISADGENKQEVEAPAGSVLLNIAQAAGQPLEGTCEGQMACSTCHVIVDAADFPK

LKKASEDEEDMLDLAAAATRTSRLSCQIVLDEKLESLTVRIPGESYNMQGM

>2Fe-2SST1(sinb:SIDU_04495)Sphingobium indicum

MTRVVFISADGEHKQEVEAPPGAVLLDVAQAAGQPLEGTCEGQMACSTCHVIVDAADFSK

LGKASEDEEDMLDLAAAATRTSRLSCQIVLDEQLDSLTVRIPGESYNMQGM

>2Fe-2SST1(syb:TZ53_17235)Sphingobium sp. YBL2

MTRVVFISADGEHKQEVEAPTGAVLLEVAQAAGQPLEGTCEGQMACSTCHVIVDAADFPK

LKRASEDEEDMLDLAAAATRTSRLSCQIVLDEKLDSLTVRIPGESYNMQGM
>2Fe-2SST1(aep:AMC99_01768)Altererythrobacter epoxidivorans
MTITVRFIDARGGVTEATAEAGDNLLRVAQAVGMPLEGTCEGQIACSTCHVIVADDWFGK
LPDASEEEEDMLDFAAGVKRCSRLSCQIELTPEMDGLTVTIPIESYDMRRK
>2Fe-2SST1(anh:A6F65_01188)Altererythrobacter namhicola
MSITVHFTSRDGDRHTAIAEPGDNLLRVAQAAGMPLEGTCEGQMACSTCHVVVAAEWFDR
LAEPSEEEEDMLDLAANVRRTSRLSCQVDLTPELDGLEVEIPAESHDARLF
>2Fe-2SST1(ado:A6F68_01585)Altererythrobacter dongtanensis
MIKVTFTEPRGGVIEAEAEPGDNLLRLGQAKGLPLEGTCEGQMACSTCHVIVAAEWFDRL
PPASEEEEDMLDLAAGACRTSRLACQIELTPDLDGLAVRVPADSHDARRL
>2Fe-2SST1(npn:JI59_02495)Novosphingobium pentaromativorans
MLSVRFVTTDGNKLTVEAEAGACLLEVAQGAGMPLEGTCEGQMACSTCHVIVSPEWFDKL
KPASADEEDMLDLAAGVTRTSRLSCQIELTEALDGIELRIPGVSHDMQID
>2Fe-2SST1(nar:Saro_2796)Novosphingobium aromaticivorans
MVRVTFVKPDGEKVSAEGEEGQRLLEVGQNVGMPLEGTCEGQMACSTCHVVIDAAWFDRL
PPAVDDEEDMLDLAAGVTRTSRLSCQIELTEALDGLVVHVPSEVQDMQGR
>2Fe-2SST1(eli:ELI_02205)Erythrobacter litoralis HTCC2594
MKIVFETAEGAAVEAEAAPGDTLLAIAQGAGLPLEGTCEGQMACSTCHVIVAKEWFAQLP
EASEEEEDMLDLAYGVSATSRLSCQIELTEKMDGLTVRIPSDSRDMSGV
>2Fe-2SST1(cij:WG74_08040)Citromicrobium sp. JL477
MKVIFHKADGQRVEAEAQAGDVLLRVAQAAGMPLEGTCEGQMACSTCHVLVAKEWFAQLP
EASEDEEDMLDLAYGVRPTSRLSCQITLTDALDGMEVTIPADAHDMSGR
>2Fe-2SST1(amx:AM2010_1166)Altererythrobacter marensis
MSVRVRFVSARGAVTEAEGAEGTPLLELAQAAGMPLEGTCEGQMACSTCHVIVAAEWFAR
LPEASEEEEDMLDLAAGVQRTSRLSCQILLSAELDGLEVQIPSESRDMSVL
>2Fe-2SST1(cna:AB433_09775)Croceicoccus naphthovorans

MVHVNFVTAKGEKVEAEAAEGDSLLKVAQAVGMPLEGTCEGQMACSTCHVVVAPAWFGKL
PISSEDEEDMLDLAADVQGTSRLSCQIVLDAPLDGLEVRIPSQVHDVQRG
>2Fe-2SST1(pns:A9D12_09360)Porphyrobacter neustonensis
MSITITFIDPRGKPVEVAADSGDNLLRVGQAAGLPLEGTCEGQMACSTCHVIVAPEWFDR
LLPAAEEEEDMLDFAAGARRTSRLSCQIELTAEMDGLTVTIPAASTDARRM
>2Fe-2SST1(porl:BG023_111367)Porphyrobacter sp. LM 6
MSITITFLDPHGKAIETKAKPGDNLLRVGQAAGLPLEGTCEGQMACSTCHVIVAAEWFDR
LPHASEEEEDMLDFAAGARRTSRLSCQIELTAELDGLTVSVPKESNDLRRM
>2Fe-2SST1(elq:Ga0102493_1175)Erythrobacter litoralis DSM 8509
MGIKVRFIDPQGRAVEAEGEPGDSLLRVGQAAGLPLEGTCEGQMACSTCHVIVAPEWFDR
LAQASEEEEDMLDFAAGVRRTSRLSCQIELTDEMDGLTVSVPSESIDARRM
>2Fe-2SST1(sphr:BSY17_1914)Sphingobium sp. RAC03
MPKLIVVNRAGEEQAVEGDNGLSVMEVIRDNGFDELLALCGGCCSCATCHVYVDPDFADV
LPAMTEDENDLLDSSDHRNDTSRLSCQVVLSDALDGLRVTIAPED
>2Fe-2SST1(nar:Saro_1115)Novosphingobium aromaticivorans
MPKIVVVNRAGEEKTVDADAGLSVMEAIRDNGFDELLALCGGCCSCATCHVYVDPAFADK
IPAMSEDENDLLDSSDHRNESSRLSCQVQITGDLDGLRVTIAPED
>2Fe-2SST1(sinb:SIDU_09805)Sphingobium indicum
MAKLIVVNRSGEEQAVEAQSGLSVMEIIRDNGFDELLALCGGCCSCATCHVFVDPAFADS
LPAISEDENDLLDSSDHRNETSRLSCQLQFSDSLDGLRVTIAPED
>2Fe-2SST1(sjp:SJA_C1-08030)Sphingobium japonicum
MAKLIVVNRSGEEQAVEAQSGLSVMEIIRDNGFDELLALCGGCCSCATCHVFVDPAFADS
LPAISEDENDLLDSSDHRNETSRLSCQLQFSDSLDGLRVTIAPED
>2Fe-2SST1(sch:Sphch_1567)Sphingobium chlorophenolicum
MAKLIVVNRSGEEQAVEAQSGLSVMEIIRDNGFDELLALCGGCCSCATCHVFVDPAFADS
LPPVSEDENDLLDSSDHRNETSRLSCQLTFSDSLDGLRVTIAPED

>2Fe-2SST1(syb:TZ53_13050)Sphingobium sp. YBL2

MAKLIVVNRSGEEQAVEAENGLSVMEIIRDNGFDELLALCGGCCSCATCHVFVDPAFADN

LPPISDDENDLLDSSDHRNETSRLSCQLTFSDSLDGLRVTIAPED

>2Fe-2SST1(sbd:ATN00_00140)Sphingobium baderi

MPKLIVVNREGEERAVDGDNGLSVMEIVRDNGFDELLALCGGCCSCATCHVYIDPAFADK

LPAMSEDENDLLDSSDHRNEQSRLSCQIMISDDLDGLRVTIAPED

>2Fe-2SST1(spmi:K663_03095)Sphingobium sp. MI1205

MPKLIVVNRSGEEQAVEGDNGLSVMEVIRDNGFDELLALCGGCCSCATCHVYVDPAFADK

LPAISEDENDLLDSSDHRNDSSRLSCQLVMNAELDGLRVTIAPED

>2Fe-2SST1(spmi:K663_17356)Sphingobium sp. MI1205

MAKLTVVNRSGEEQTIEAQDGLSVMELLRDNGFDELLALCGGCCSCATCHVYVDPSFADS

LPTISDDENDLLDSSDHRNEHSRLSCQLPFSDSLDGLRVTIAPED

>2Fe-2SST1(sphb:EP837_01905)Sphingobium sp. EP60837

MAKLIVVNRSGEEQAVEGDNGLSVMEVIRDNGFDELLALCGGCCSCATCHVYVDPAFADK

LPALSEDENDLLDSSDHRNDSSRLSCQLVLNDDLDGLRVTIAPED

>2Fe-2SST1(sphi:TS85_16420)Sphingomonas sp. WHSC-8

MPKLIVVTREGEERTVDGDTGLSVMEVIRDNGFDELLALCGGCCSCATCHVHVDPEFAAK

LPPMSEDENDLLDSASDRNDQSRLSCQIPFTDALDGLKVTIAAED

>2Fe-2SST1(cna:AB433_13620)Croceicoccus naphthovorans

MPNIIVVDREGNEKTVEADDGLTVMEAIRDNGFDELLALCGGCCSCATCHVYVDPAFAAK

LPELSEDEDDLLDSSDHRNETSRLSCQIPLNADLDGIKVTIAPED

>2Fe-2SST1(npn:JI59_08680)Novosphingobium pentaromativorans

MPKLTIVTREGEEREIAAEEGRSVMEVIRDAGFDELLALCGGCCSCATCHVMVAPEFADR

LPPISEDENDLLDSSEHRTELSRLSCQLPVTESIDGLRITIAPED

>2Fe-2SST1(sgi:SGRAN_3239)Sphingopyxis granuli

MPSLTVVDREGTAHTLEADAGLTLMEVIRDNGFDELLALCGGCCSCATCHIHVDPAFAAK

LPAMSDDENDLLDSSEDRNETSRLSCQIPFDDALDGIKVTIARED
>2Fe-2SST1(sphl:LPB140_05125)Sphingopyxis sp. LPB0140
MPTINVIDRSGAKREVEAEMGLSVMEVIRDNGFDELLALCGGCSSCATCHVYIDAEYADK
IPAMQEDEDDLLESTDHRNATSRLSCQIEVSDAIDGMTVTIAPED
>2Fe-2SST1(blas:BSY18_2454)Blastomonas sp. RAC04
MPKINLVTRDGTASIIEAGTDLTLMEAIRDNGCDELLALCGGCCSCATCHVHIDDAFLAR
IPAMSEDENDLLDSSEHRNANSRLSCQIALTEALDGITVTLAKED
>2Fe-2SST1(syb:TZ53_18515)Sphingobium sp. YBL2
MPELNVITRDGAQQTINASADTSVMEAIRDSGFDELLALCGGCCSCATCHVYVDPAFAQS
LPPVSDDESDLLDGSPHRNDYSRLSCQVMMTEALSGLTITIAPED
>2Fe-2SST1(sbd:ATN00_17430)Sphingobium baderi
MPELNVITRDGAQQIINATADVSVMEAIRDAGFDELLALCGGCCSCATCHVYVDPAFAAS
LPPVSDDESDLLDGSPHRNDYSRLSCQVVMTDALSGLTVTIAPED
>2Fe-2SST1(sphi:TS85_13360)Sphingomonas sp. WHSC-8
MAEIRITTRDGVERPVAMTPGYSLMEAIRDAGIDELMALCGGSCSCATCHVFIDPEFADR
LPPISEDEDDLLDSSDHRGPTSRLSCQIPLSDALDGLAATIAPED
>2Fe-2SST1(sbd:ATN00_07550)Sphingobium baderi
MSTLNIVTRDGSAVRVTPEPGLSVMEIIRENGIDELMALCGGCCSCATCHVFIAEDSFDQ
LPAMSADEDDLLDSSEHRRHVSRLSCQIPWSDVLDGLTITIAPED
>2Fe-2SST1(rhi:NGR_b14760)Sinorhizobium fredii NGR234
MEEDMSNFTVISRNGQVVPIEGREGISVMEHIRDAGVDELLALCGGCCSCATCHVYVDDD
FQEHLPALTVDEDDLLDSSDHRRSQSRLSCQIRFAPELKGMIVTLAPED
>2Fe-2SST1(rhi:NGR_b16340)Sinorhizobium fredii NGR234
MSNFTVISRNGQIVPVEGRDGISIMEHIRDAGVDELLALCGGCCSCATCHVYVDDDFQEH
LPSLSVDEDDLLDSSDHRRPQSRLSCQVRFTAELAGMIVTLAPED
>2Fe-2SST1(eli:ELI_01725)Erythrobacter litoralis HTCC2594

MPKLVVTTREGETSEVQVDDGLTVMEAIRDNGFDELLALCGGCCSCATCHVHVDESFVEK
LPRMSEDEDDLLESSDHRESNSRLSCQIPFTPDLDGLKVTIAPED
>2Fe-2SST1(elq:Ga0102493_11208)Erythrobacter litoralis DSM 8509
MPKLVVTNREGETSEIDVEDGLTVMEAIRDNGFDELLALCGGCCSCATCHIHVDPAAMDK
LPAMSEDEDDLLESSDHRTETSRLSCQIPFTDALDGLKVTIAPED
>2Fe-2SST1(aep:AMC99_01878)Altererythrobacter epoxidivorans
MPKLIVTTREGETSEIQVDDGLTVMEAIRDNGFDELLALCGGCCSCATCHIHVDPSFKDK
LPAMSEDEDDLLESSDHRNENSRLSCQIPFTSDLDGLKVTIADED
>2Fe-2SST1(anh:A6F65_02274)Altererythrobacter namhicola
MPKLIVTTREGETTEVDVESGLTVMEAIRDNGFDELLALCGGCCSCATCHVHVDAQYKDK
LPAMSEDEDDLLDSSDHRDDTSRLSCQIPFTDELDGLKVTIAQED
>2Fe-2SST1(pns:A9D12_08835)Porphyrobacter neustonensis
MPRLVVTNREGATSEIEVGDGLTVMEAIRDNGFDELLALCGGCCSCATCHVVVDPAFADK
LPKISEDEDDLLESSDHRVATSRLSCQIPLTADLDGLHVTIAPED
>2Fe-2SST1(porl:BG023_111277)Porphyrobacter sp. LM 6
MPRLVVTNREGTTSEIEVSDGLTVMEAIRDNGFDELLALCGGCCSCATCHVHVDPAFADK
LPKMSEDEDDLLESSDHRAAGSRLSCQIPFTADLDGLHVTIAPED
>2Fe-2SST1(aay:WYH_01357)Altererythrobacter atlanticus
MPKLLVVTRDGEERTVEADSGLTVMEVVRDNGFDELLALCGGCCSCATCHVHVDPAFKDK
LPAMSEDEDDLLDSSEHRDENSRLSCQIPFTDELDGMKVRIAEED
>2Fe-2SST1(ado:A6F68_01490)Altererythrobacter dongtanensis
MPKLIVVNRAGEEKAVDVADGLTVMEAIRDNGFDELLALCGGCCSCATCHVHVDPAFKER
LPAMSEDEDDLLESTDHRDENSRLGCQVPFTADLDGMRVTIAQED
>2Fe-2SST1(amx:AM2010_425)Altererythrobacter marensis
MPKLIVVNRSGEESSIDVEDGLTVMEAIRDNGFDELLALCGGCCSCATCHVHVDPAFRDK
LPAISEDEDDLLESSDHRDENSRLGCQIPFTGDLDGLKVSIAQED

>2Fe-2SST1(sphm:G432_02590)Sphingomonas sp. MM-1

MPKLFVVTREGEEKVVDGQIGLTVMENIRDNGFDELLALCGGCCSCATCHVHVDAADFDK

LKPMGDDENDLLDSSDHRVATSRLSCQIPFTEELDGLRVTIAPED

>2Fe-2SST1(cij:WG74_07555)Citromicrobium sp. JL477

MPKLTVVDRSGTERTIEVGDGLTVMEAIRDNGFDELLALCGGCCSCATCHVHVDPAFGDK

LPAMSEDEDDLLESSDHRNETSRLSCQIEFTSDLDGLKVQIAPED

>2Fe-2SST1(blas:BSY18_1559)Blastomonas sp. RAC04

MPKLIVVNRAGEESTIEADTGLSVMEAIRDNGFDELLALCGGCCSCATCHVHVDAEWTDK

LPPVSADEDDLLDSSDHRNATSRLSCQIQMTAALDGLKVAIAPED

>2Fe-2SST1(sphr:BSY17_102)Sphingobium sp. RAC03

MPKLIVVNRAGDETSVDARDGVSVMEAIRDNGFDELLALCGGCCSCATCHVYVDEEWTRA

VGTPGEDENDLLDSSDHRGDRSRLSCQIMMTAGLDGLRVAIAPED

>2Fe-2SST1(smaz:LH19_14855)Sphingopyxis macrogoltabida 203

MAKLIVVTRDGTEHEIEGDTSLTVMENIRDAGFDELLALCGGCCSCATCHVHVEAGDKAA

MPAMSEDENDLLDSTTDRDDNSRLSCQLPFSDALDGLRVRIAAED

>2Fe-2SST1(sphk:SKP52_11840)Sphingopyxis fribergensis

MAKLIVVTRDGTEHEIEGDTSLTVMENIRDAGFDELLALCGGCCSCATCHVHVEAGDKGA

MPAMSEDENDLLDSTTDRDDASRLSCQIPFSDALDGLKVRIAAED

>2Fe-2SST1(sphp:LH20_08015)Sphingopyxis sp. 113P3

MAKLIVVTRDGTEHEIEGDASLTVMENIRDAGFDELLALCGGCCSCATCHVHVEAGNKDA

LPAMSEDENDLLDSTSDRDENSRLSCQIPFSDALDGLKVRIAEED

>2Fe-2SST1(ster:AOA14_16445)Sphingopyxis terrae

MAKLIVVTRDGTEHEIEGDTSLTVMENIRDAGFDELLALCGGCCSCATCHVHVEAGDAAA

LPAMSEDENDLLDSTSDRDDTSRLSCQIPFSDALDGLKVRIAAED

>2Fe-2SST1(sgi:SGRAN_2600)Sphingopyxis granuli

MAKLIVVTRDGTEHEIEGDTSLTVMENIRDAGFDELLALCGGCCSCATCHVHVEEGNRDA

IPAMSEDENDLLDSTSDRDGNSRLSCQIPFTAELDGLRVRIAEED

>2Fe-2SST1(npn:JI59_14295)Novosphingobium pentaromativorans

MPQITVVNQSGEESSVEASEGRTLMEVIRDNGFDELLALCGGCCSCATCHVHIDPAFVDK

LPEMSEDENDLLDSSEHRNEYSRLSCQIPVTGALEGCKVTIAQED

>2Fe-2SST1(sal:Sala_0845)Sphingopyxis alaskensis

MAKLIVVTRDGTEHEIEGDTSLTVMENIRDAGFDELLALCGGCCSCATCHVHVEAGDRDA

LPAMSEDENDLLDSTTDRDETSRLSCQIPFSDALDGLKVRIAAED

>2Fe-2SST1(npp:PP1Y_AT7331)Novosphingobium sp. PP1Y

MPQITVVNQSGEESSVEASEGRTLMEVIRDSGFDELLALCGGCCSCATCHVHIDPAFMEK

LPEMSEDENDLLDSSDHRNEYSRLSCQIPVTGALEGLKVTIAQED

>2Fe-2SST1(sphg:AZE99_03245)Sphingorhabdus sp. M41

MPTIHVTGRDGEEKAVSVDSGISVMEAIRDNGFDELLALCGGCCSCATCHVHIDADWKAK

LPPMSEDEDDLLESSDHRDEFSRLSCQIELSDDLEGLKVRIAEED

>2Fe-2SST1(spmi:K663_20183)Sphingobium sp. MI1205

MPRLIVTTRSGEETLIVESREVSVMEAIRDAGIDEVLALCGGCCSCATCHVHVDEDWFEK

LPPMTDDEGDLLDSSDHRTATSRLSCQLPFSEALDGLRVTIAPED

>2Fe-2SST1(sphb:EP837_03874)Sphingobium sp. EP60837

MPQLIVTTRKGAEINIVESREVSVMEAIRDAGIDEVLALCGGCCSCATCHVHVDEDWIEK

LPPMTDDEGDLLDSSDHRTATSRLSCQLPFSQALDGLRVTIAPED

>2Fe-2SST1(cij:WG74_12065)Citromicrobium sp. JL477

MINVLATLRDGSTKTISTTPDGSLMEAIRDSGIDELLALCGGCCSCATCHVHVDESWLAK

LPDISEDEDDLLESSDHRDDQSRLSCQIPLTEDLDGLKVTIAQED

>2Fe-2SST1(nar:Saro_0509)Novosphingobium aromaticivorans

MSKHAMIDLKVVKRDGTKLTLQASPGMSVMEALRDNGLDELLALCGGCCSCATCHIYVEA

GPDGVDEVGGADEDDLLESSDHRRHNSRLSCQITLGSAMSGLVVRVAPED

>2Fe-2SST1(ssy:SLG_08500)Sphingobium sp. SYK-6

MAQLKVVTRDGSLHEFEAPDGYTVMEAIRDQGIDELLAICGGCCSCATCHVFVEEAFLDK
LPPLKGDEDDLLDSSDHRQANSRLSCQLPIGPELGGMTVTIAPED
>2Fe-2SST1(ssy:SLG_18840)Sphingobium sp. SYK-6
MVQIVVTDRGGAQQSIEAKSGLSLMEVIRDHGIDELLALCGGCLSCATCHVYVELAYLPK
LPAMSADEDDLLDSSSHRTAASRLSCQIVIGDELDGLEATIAPED
>2Fe-2SST1(swi:Swit_0363)Sphingomonas wittichii
MPTIIVTTRDGQELSLEADTGLSLMEVIRDGGADELLALCGGCCSCATCHVKVDPAFLAA
LPPMSEDESDLLDSSDHRDATSRLSCQISVDDGLAGLRVAIAPED
>2Fe-2SST1(sch:Sphch_3575)Sphingobium chlorophenolicum
MPNIIVTARDGAEREIAAETGLSLMEAIRDAGIDEMLALCGGCCSCATCHVKVDPAFTAL
LPTVSEDESDLLDSSEHRDAYSRLSCQIPLTAELDGLRIAIAPED
>2Fe-2SST1(syb:TZ53_05355)Sphingobium sp. YBL2
MPNVIVTTRDGAEREIAAETGLSLMEAIRDAGIDEMLALCGGCCSCATCHVKVDPAFFRL
LPTVSEDESDLLDSSEHRDEYSRLSCQIPLTAELDGLRIAIAPED
>2Fe-2SST1(hba:Hbal_2471)Hirschia baltica
MIRIVATTRDGTRSELSADSGLSLMEVLRDRDLDVEGVCGGAVSCASCHVYVADEWVDKL
SAPDEMEEALVGDLVHAKPNSRLCCQIVLSDKFDGLEVTVAPSEM
>2Fe-2SST1(con:TQ29_17605)Confluentimicrobium sp. EMB200-NS6
MPILNVTDRDGSEKQVDVTMTESLMHVLRDAGYHIAAECGGAAACGTCHVYVDEAWLSKL
PEAGEIEAEMVDLLEETKPTSRLSCQVQMDDSLDGLKVTLAPEE
>2Fe-2SST1(magq:MGMAQ_3127)Magnetospira sp. QH-2
MPTLTVTDAQGVTKELDADTGLSVMEILREAGYPVAGECNGSLACATCHVIIDPNWFGKL
EDQAEEEEDILDTVFNLEETSRLACQIMMSDDLSGLAVSLPS

>2Fe-2SST1(aex:Astex_1069)Asticcacaulis excentricus
MSDELHILCTDRDGVQHTLPAIEGWRIMEIIRDNGLPIKAECGGALECATCHVYVGADWL

PKLIGKSDEEEEKLDEAFLVKRNSRLSCQILMRDDLNGLEVTLAPEG
>2Fe-2SST1(bvv:BHK69_21665)Bosea vaviloviae
MHVRVTDREGEQRELEALEGWRLMEIIRDWGLPIKAECGGACACATCHVYVAADWVDKLH
PPREEELERLDEAFDVRPNSRLACQILFSPELDGLTVVLAPELD
>2Fe-2SST1(hoe:IMCC20628_01988)Hoeflea sp. IMCC20628
MHIHVTDQTGTRHTLEALEGFRVMEIIRDWGLDIKAECGGACACATCHVHVSDDWTDRLY
PIEAEEEDMLDQAFDVRDNSRLSCQLLMSEELDGLEVTLAPGTETGDRAAA
>2Fe-2SST1(psin:CAK95_03715)Pseudorhodoplanes sinuspersici
MKISVTDHAGTEHELEALPGWRVMEIIRDWDLPIKAECGGACACATCHVYVDAAWFGRLP
GESEEEIDQLEILADRMPNSRLACQVLMSEELDGLKVTLAPGSRP
>2Fe-2SST1(phl:KKY_1892)Pelagibacterium halotolerans
MRIFVTDQDGTEHELEALEGWRVMEVIRDWGLNIKAECGGSCACATCHVFVDAEWQDKLN
PPTDEEEDMLDTVADYEANSRLSCQILMSEELDGLKVTLADSARQDEAA
>2Fe-2SST1(deq:XM25_10530)Devosia sp. H5989
MKIHVTDQAGVEHELEGLEGWRVMEVIRDWGLNIKAECGGACSCATCHVYVAPEWFDKVG
TRSDDEEDLLDTVGDVKPTSRLSCQILMSDELDGLKVTLAASAAQ
>2Fe-2SST1(bvv:BHK69_19925)Bosea vaviloviae
MAILNVIDRDGRAHELEAVEGWRVMEILRDYKVGIEGVCGGSCDCATCHVVVAPDWAKRL
PEPREDEIDALDTLPLIEATSRLSCQIIWSDDLDGLTLTLAEAA
>2Fe-2SST1(mbry:B1812_19095)Methylocystis bryophila
MQDAAARAPQQHAVVLVEDREGKLHELETLEGWRLMEILRDHGVGMDTNCGGSLACAECH
VLIDPAWMGRLPAPSQEELDKLDELPLIHENSRLSCQILWTDDYDGMKVTVAQAT
>2Fe-2SST1(msl:Msil_1812)Methylocella silvestris
MDEPAIEPAEPEGPPRTPLAVTLSEGGLSRRIDGFDGERLLDALLRAKAAVMPLCNGRAC
CGGCRIRIEKSWRDRVTPSGRGERSVLRYIDDPHEDDRLSCQIALSAEVEGLVVRTLERQ
PAPAQAGADAEEINAGK

>2Fe-2SST1(pzu:PHZ_c0215)Phenylobacterium zucineum
MHDGQAAHREPQAGGPRKACRPHDFHERGRRLSERLTRSAMAKITYIEHDGTEHVIDVKP
GLSVMEGAVKNNIPGIDADCGGACACATCHVYVDEAFLAKTGTRSAMEESMLDFAEGVEE
NSRLSCQIKVTDDLDGLVVRMPESQH
>2Fe-2SST1(msc:BN69_2451)Methylocystis sp. SC2
MAKMSFEDAATRYGAEPGGGAPTKVKITFIDSQGQARSVEGEVGSTVMETARRNDIPEIT
AECGGACACATCHVYVDEKWAEKTGKASQMEEDMLDFAFDVKPNSRLCCQITVRPELDGL
VLTTPAQQG
>2Fe-2SST1(same:SAMCFNEI73_Ch2044)Sinorhizobium americanum
MRDHCRFSGRSRNRASARVDWPVDLLEETALNSGWPRLPRRLVLAGGSHPFIPEREMTKL
TIVAFDGTRHDLDVENGSTVMENAVRNSVPGIEAECGGACACATCHVYVDDAWSAAVGAP
EAMEEDMLDFAYDVRPTSRLSCQIKMSEALDGLIVHVPERQA
>2Fe-2SST1(abs:AZOBR_70188)Azospirillum brasilense Sp245
MPKMTFIEPNGTRHEVDAPLGLSVLEVAHKHGLDLEGACEGSLACSTCHVIVEPEWFDVL
NEASEDEEDMLDLAFGLTKTSRLGCQIIISEELDGLAVRLPGGTNSGHALTYRRGALRGR
RGPHPPPGNRRASPLAGAAPPVLRW
>2Fe-2SST1(npp:PP1Y_AT28162)Novosphingobium sp. PP1Y
MPQRRRRESELLSVQFVTADGNKLTVQAEAGARLLEVAQAAGMPLEGTCEGQMACSTCHV
IVAPEWFERLKPASNDEEDMLDLAAGVTRTSRLSCQIELTEALDGIELRIPGVSHDMQIG
>2Fe-2SST1(nar:Saro_1477)Novosphingobium aromaticivorans
MRADCIPLHSKQDPPLTAILVTTRDGTRTEIQAEPGLSLMEALRDAGIDELLALCGGCCS
CATCHVLVAPAFADRLPALSGDENDLLDSSDHRTPHSRLSCQITINDKLEGLEVEIAPED
>2Fe-2SST1(msc:BN69_0738)Methylocystis sp. SC2
MSNIDNIVARRPAESSQQSLPTLHVEDRDGVVHELEAVEGWRLMEILRDHGVGMDNTCGG
ALACAECHVVLDAESARRVPPPREEEVEKLDELPMLYENSRLSCQIIWSDEMSGLRLKLT
QET

```
>2Fe-2SST1(bmg:BM590_A1025)Brucella melitensis M5-90
MEAAIRNGIPGIDAECGGACACATCHVYVDDDWADTVGGPDPMEEDMLDFAYEVRPTSRL
SCQIRVTDDLEGLVVQVPERQN
>2Fe-2SST1(aay:WYH_00986)Altererythrobacter atlanticus
MAEAQPGDNLLEVGQACGMPLEGTCEGQMACSTCHVIIAPEWFARLPEASEEEEDMLDLA
ADVARTSRLSCQIDLTDDLDGMEVRMPSASHDMQGR
>2Fe-2SST1(sbd:ATN00_08840)Sphingobium baderi
MLALRDEAGFEIAGECGGFCICGTCHVYIARSEQAAFSPPSEEESELAAFLQHYGPDSRL
ACQIPLASVPEGLTIHIAPEE
>2Fe-2SST1(swi:Swit_5088)Sphingomonas wittichii
MPKVVFVAADGREIETNVDIGTDLMHAGLYNSVPGLLGECSGGLACATCRVHVPAEWQGV
LPAALPAEAELLGFCEESPPEARLSCQIKMTQELDGLRLLVPEDQD
>2Fe-2SST1(swi:Swit_4893)Sphingomonas wittichii
MPKVIYVSEDGSELETTVDVGVDLMHAGLYNSIPGILGECSGGLACATCRVRVPVEWQSI
LPPAFPSEAELLGFCDEAPPEARLSCQIKMTQELDGIRLLVSNNLD
>2Fe-2SST1(sch:Sphch_3593)Sphingobium chlorophenolicum
MVHVTFITPSGVATTLDGDVGQSVMDIGKRAGVENIIGECGGSAACATCHVHVDPAWIDL
VGPPSDDEWDMLDFAHGKRADSRLSCQIRLRPALEGLIVHTPERQG
>2Fe-2SST1(syb:TZ53_05790)Sphingobium sp. YBL2
MVHVTFITPAGIATTLDGDVGQSVMDIGKRAGVENIIGECGGSAACATCHVHVDPAWIDR
VGPPSADEWDMLDFAHGKRADSRLSCQIRLRAALDGLIVHTPERQG
>2Fe-2SST1(aak:AA2016_1168)Aminobacter aminovorans
MKVEVHFRLPDNSVRKVEATCGLSLMEAGKQAGVPGIIGDCGGGAMCATCHVYVDLDWQD
RAGEPEATESLMLQLTEEQRDNSRLSCQITVEEELDGLVVEVPKEHI
>2Fe-2SST1(swi:Swit_1759)Sphingomonas wittichii
MVRVRFQRPDGSETACDVAAGLSLMEAALNNGVDEIIADCGGALSCATCHVYVDDGWMDR
```

TGAPSEVEEEMLEFAVDRRPNSRLSCQIKLGEGLDGLSVALPQRQQ

>2Fe-2SST1(aak:AA2016_1195)Aminobacter aminovorans

MSLITVHFIHPDERVESVQTDTGLTVMEVAREAGVSGILAECGGGAICSTCHVHVAQDWY

HRLTPPEATEGMLLELAPGHDAYSRLSCQIELDATHDGISVRVPECQSDY

>2Fe-2SST1(rpx:Rpdx1_3651)Rhodopseudomonas palustris DX-1

MPSITFILPDGERRTTEAAVGDTAMYAALSLGLDGVVAECGGNAVCATCHVYVEHGLEKL

PAVAGDEDDLLDGTAAERLPNSRLSCQIKLSSDLDGLILRIPDRQV

>2Fe-2SST1(rpa:RPA1872)Rhodopseudomonas palustris CGA009

MPSITFILPDGERRTTEAAVGDTAMYAALSLGLDGVVAECGGNAVCATCHVYVEHGLEKL

PAVAADEDDLLDGTAAERLPNSRLSCQIKLSSDLDGLILRIPDRQV

>2Fe-2SST1(rpt:Rpal_2078)Rhodopseudomonas palustris TIE-1

MPSITFILPDGERRTTEAAVGDTAMYAALSLGLDGVVAECGGNAVCATCHVYVEHGLEKL

PAVAGDEDDLLDGTAAERLPNSRLSCQIKLSSDLDGLILRIPDRQV

>2Fe-2SST1(swi:Swit_0998)Sphingomonas wittichii

MKISYLDQDGTRTDLDAGEGTNLMELAVRGGVTTIEGECGGALACATCHVHIPDEWRGVT

GEPSDDEREMLEFGVGVDERSRLCCQIRVTAAMDGLVVLTPASQR

>2Fe-2SST1(swi:Swit_1843)Sphingomonas wittichii

MPMITFVAPDGAEYVVDARGLHSVMEAARNHMVPGIDADCGGSGSCATCHVHVDAAWAQA

TGAADEMEQAMLDCVEEPSATSRLSCQIRVSDALDGLVVRLPARQG

>2Fe-2SST1(tmo:TMO_c0588) Tistrella mobilis
MPKITFIERDGNRREVEAPVGLSILEIAHRNSIDIEGACEGSLACSTCHVIVAPEDFERL
PDAAEEEEDMLDLAWGLTKTSRLGCQIIITEELDGLTVSLPTEVHNALID

Subtype 2

>2Fe-2SST2(rep:IE4803_PC00148)Rhizobium etli bv. phaseoli IE4803

MAEITFILPDGGHKACPASEGLSLMELALQNSVPGIVAECNGAAACGTCHVIMDDALIDV

LDPISDHENDMLDFTNAPREPGSRLSCQIRVDGRLNGAVVRVASEM

>2Fe-2SST2(rei:IE4771_PD00148)Rhizobium sp. IE4771

MAEITFILPDGGHKACPASEGLSLMELALQNSVPGIVAECNGAAACGTCHVIMDDALIEV

LDPISDHENDMLDFTNAPREPGSRLSCQIRVDGRLNGAVVRVASEM

>2Fe-2SST2(mamo:A6B35_25070)Mesorhizobium amorphae

MPTIRFLLADGAELAVEAQNGRTLMETALESSVPGIIAECNGSAACATCHVVLPEEIMVA

LGPISEHENDMLDFAEAPRESGSRLSCQVRVSDMLDNVSVRIPSA

>2Fe-2SST2(sfd:USDA257_c26490)Sinorhizobium fredii USDA 257

MPTITFITAEGKEHAVTAEIGRSVMEIALEKSIPGVVAECNGSAACATCHCYFDPAFVEA

IGPIGEHESDMLDFTASPRRPESRLSCQIRVADALHGMTVRIPAVQ

>2Fe-2SST2(same:SAMCFNEI73_pC1878)Sinorhizobium americanum

MPTITFITAEGKQHAVTAEIGRSVMEIALEKNIPGVVAECNGSAACATCHCYFDPAFAAA

IGPIGEHESDMLDFTASPRRPESRLSCQVRVADALDGMTVHIPAVQ

>2Fe-2SST2(rhi:NGR_b07650)Sinorhizobium fredii NGR234

MPTITFITAEGKQHAVTAEIGRSVMEIALEKSIPGVVAECNGSAACATCHCYFDPAFTEA

IGPVGEHESDMLDFTAAPRRPESRLSCQVRVVDTLDGMTVRIPAMQ

>2Fe-2SST2(sfh:SFHH103_06381)Sinorhizobium fredii HH103

MPTITFITAEGKEHAVTAEIGRSVMEIALEKSIPGVVAECNGSAACATCHCYFDPAYADM

IGAIGEHESDMLDFTASPRRPESRLSCQVRVVDTLDGMTVRIPAMQ

>2Fe-2SST2(pde:Pden_3464)Paracoccus denitrificans

MGAPRRTTERNTVPFITWRDADGTEISANVAAGTNLMRAAVDAGVQGIHGDCGGALACAT

CHVATDTAWAERLGPPGALEDEMLDMVEGERTPTSRLSCQILARDELEGLVLIVPACG

>2Fe-2SST2(sil:SPO0766)Ruegeria pomeroyi

MRVTWKLADGREIAAEVAPGLSLMEAALANNVPGVIGECGGCLSCATCHVVVDAAWSERT

GAPGEFEDAMLDITAAERQPRSRLSCQIEAHPDLDGLVLHVPEA

>2Fe-2SST2(lvs:LOKVESSMR4R_00421)Yoonia vestfoldensis

MKVTWTLQDGGTRTADVAPGTNMMEAAVANGINGVVGECGGCLSCATCHVYVADDWADKT

GGPEDFEDAMLDATEAERKATSRLSCQIVADDSLDGLHLIVPTP

>2Fe-2SST2(cid:P73_4762)Celeribacter indicus

MVNIVFIALDGEKHEVSATPGASVMQAAMGASVDGILAECGGAGACATCRCGVVSAPDGS

IEAPTDMELEMLEFVAEDLSNNDRLTCQLTVTPSLEGAVFQVRDNDV

>2Fe-2SST2(bra:BRADO6809)Bradyrhizobium sp. ORS 278

MTTITFIHADNRSQTVEASDGDSLMMAALTHGVDGIVAECGGNAVCATCHVYIDEAWTAR

LDPVSDDEDALLDGTAAERLPNSRLSCQIKVQPALAGLVVRIPDKQS

>2Fe-2SST2(bbt:BBta_0730)Bradyrhizobium sp. BTAi1

MTTITFIHPDNRSETVEAEDGATVMLAALTHGVDGIVAECGGNAVCATCHVYVDDAWTSK

LEPVSDDEDALLDGTAAERRPNSRLSCQIKVQPALAGLVVRIPDRQS

>2Fe-2SST2(aol:S58_06530)Bradyrhizobium oligotrophicum

MTTITFIHADNRSERVEAVDGDSLMMAALTHGVDGIVAECGGNAVCATCHVYVDEAWASK

LDPVSDDEDALLDGTAAERRPNSRLSCQIKVQPGLAGLVIRIPDRQS

>2Fe-2SST2(rpb:RPB_3614)Rhodopseudomonas palustris HaA2

MPSITFIHPDGRSEIVDAAIGDSAMFAALNHGIDSIVAECGGNAVCATCHVYVDDLWLAK

LPPVDANEDDLLDGTASDRLPNSRLSCQIKIAPELDGLVLRLPERQT

>2Fe-2SST2(brc:BCCGELA001_34410)Bradyrhizobium sp. CCGE-LA001

MPAITFIHPGGKSDRVEAIDGESAMQAATRHGLDGILAECGGNAMCATCHVYVDDAWLAR

LPDIADDEDALLDGTASERLPNSRLSCQIHLTPALDGLVLQLPERQV

>2Fe-2SST2(bic:LMTR13_36455)Bradyrhizobium icense

MPSITFVHPDGRAQRIDGGDGESAMQAATRHDVNGILAECGGNAMCATCHVYVDEDWLAR

LPAMGDDEDALLDGAAAERRANSRLSCQIKLAADLDGLVLNLPDRQL

>2Fe-2SST2(bja:blr1049)Bradyrhizobium diazoefficiens USDA 110

MPAITFIHADGKSDRVETSGGESAMQAATRHGLDGILAECGGNAMCATCHVYVDEAWLAR
LPAMADDEDALLDGTATERLPNSRLSCQIMITPALDGLVLRLPERQV
>2Fe-2SST2(bju:BJ6T_10740)Bradyrhizobium japonicum USDA 6
MPAITFIHADGKSDRVETSGGESAMQAATRHGLDGILAECGGNAMCATCHVYIDAAWLGR
LPAMADDEDALLDGTAAERLPNSRLACQIMITPVLDGLVLRLPERQV
>2Fe-2SST2(bjp:RN69_10165)Bradyrhizobium japonicum E109
MRIRFVQPDGTEKCVNEDPGVSVMVAAIRHGVAGINGDCGGSMDCATCHVYLDEAQSKLV
PPPTSHEIDLLSAVVAERRPTSRLSCQLRPFDRSDELIVHVPDAQ
>2Fe-2SST2(bjp:RN69_05240)Bradyrhizobium japonicum E109
MPAITFIHADGKSDRVETSGGESAMQAATRHGLDGILAECGGNAMCATCHVYIDAAWLGR
LPAMADDEDALLDGTAAERLPNSRLACQIMITPVLDGLVLRLPERQV
>2Fe-2SST2(mno:Mnod_8461)Methylobacterium nodulans
MPRIVFVGRDGAEHGVEVPAGISAMAAAIRSNVRGIEAECGGSLDCATCHVYVDNRFVGL
LPEPSELEREMLACVAADRRETSRLSCQIVLTPELEGLTLHLPEVQS
>2Fe-2SST2(ngg:RG540_PA03930)Neorhizobium galegae bv. orientalis HAMBI 540
MTKVIFISPDGAETAVDAIDGDNVMRTALANDIDGIVAECGGSMMCATCHCYVDAAWKER
VGPRSDGEDDMLESASSEVRPTSRLSCQIRILPDLDGLVIHLPESQT
>2Fe-2SST2(pphr:APZ00_12860)Pannonibacter phragmitetus
MVKVTYVHHDGTRTEVDGKEGDTVMHTAVAQDVEGIVGECGGAMMCATCHCYVDDEWLDA
AGERRDGEEDMLDCGAAEVKPNSRLSCQIRLSPALDGLVVHLPEEQM
>2Fe-2SST2(pphr:APZ00_24835)Pannonibacter phragmitetus
MTKIHFISATGIETVIDARDGDSVMHAAILNDVEGILAECGGSMMCATCHCYVDPAWQDR
VTPPSEPEAGMLASAASAVRATSRLSCQITVGPELEGLVIHLPEAQL
>2Fe-2SST2(lap:ACP90_00235)Labrenzia sp. CP4
MVQITFVQPNGSRKTIDASEGASVMETAVSHGVDGIFAECGGAMMCATCHCYVDEAWSDK
TGARTDGEEIMLECAAADLRETSRLSCQIRLTGALDGLVVHLPDEQA

>2Fe-2SST2(lagg:B0E33_11745)Labrenzia aggregata
MVQITFVQPNGSRKTIDASEGASVMETAVSHGVDGIFAECGGAMMCATCHCYVDEAWSDK
TGARTDGEEIMLECAAADLRETSRLSCQIRLTGALDGLVVHLPDEQA
>2Fe-2SST2(thw:BMG03_04540)Thioclava nitratireducens
MIKVTYVAQDGTRTNIDADEGDSVMHAAVSNDVDGIVGECGGSMMCATCHCYVDDAWAEK
VGPRSAGEDDMLEGAASEVTERSRLSCQIKLTPELDGLVVHLPEEQF
>2Fe-2SST2(sphm:G432_04860)Sphingomonas sp. MM-1
MITINVTTRDGDRQQIDGQEGSALMEMLRDAGLGVDGTCGGMCSCGSCHVYIASETTLPK
PSEDEADMLEGMGSIVEVRPSSRLSCQIILKPELDGLEVEIAPQF
>2Fe-2SST2(rhc:RGUI_1200)Rhodovulum sp. P5
MSAPTTGHPNEARMAKITYIEHNGTEHVVEVANGLTVMEGARDNSIPGIDADCGGACACS
TCHAYIHPDWVDRLPAIEDMEADMLDFAYATDPKRSRLTCQVKVTDALDGLIVQMPEKQI
>2Fe-2SST2(sit:TM1040_1949)Ruegeria sp. TM1040
MGLARCRRQRYTRYRVPKKGRRIMAKITYIEHNGTEHVVDVANGLTVMEGARDNNIPGIE
ADCGGACACSTCHVYIAPDWVEKLPAKDDMEEDMLDFAFEPDAARSRLTCQIKVTDALDGLVVHMPEKQI
>2Fe-2SST2(mcg:GL4_0882)Methyloceanibacter caenitepidi
MMEVIRDWGLPIKAECGGACACATCHVWVAEEWVDKLVPPTDEETEMLDGAFSVDDRRSR
LSCQLLMTPELDGLVVELAPESLTDIVDSTAATGTSG
>2Fe-2SST2(bbt:BBta_1599)Bradyrhizobium sp. BTAi1
MRTVEAAHDESAMQAAKRNGIDGIIGECGGSCICATCHCHVDQAWLTRVGPAGDIEADVL
EFEATDVRPESRLACQIPITDALAGLVLHVVGRSR
>2Fe-2SST2(cid:P73_3499)Celeribacter indicus
MVKITYIEFGGTEHVVDVPVGSTVMEGARDNGIPGIDADCGGACACSTCHVYVAPDWVDK
LPERDPMEEDMLDFAFEPDPVRSRLTCQIKVTEALDGLVVNMPEKQI
>2Fe-2SST2(rga:RGR602_PC02299)Rhizobium gallicum
MATISVRTRDGELHSFEGRVGASLMENIRNGGFDELLAMCGGCLSCATCHVFVEELPASA

SLPIPSIDETELIEGSDYRQENSRLSCQIPFDETLSGIVIGIAPED

>2Fe-2SST2(pla:Plav_2127)Parvibaculum lavamentivorans

MPRVKYVEANGREYLVEADTGISAMEAAVKNGVPGIDGDCGGAAACATCHVYVDPDWIGK

TGPAAEGLEKSMLEFAEDVNENSRLACQITLSDTLDGLVLKLPEKQH

>2Fe-2SST2(sil:SPO1348)Ruegeria pomeroyi

MAKITYIEHNGTQHTVEVANGLTVMEGARDNNIPGIEADCGGACACSTCHVYIHPDWVEK

LPAKDDMEEDMLDFAYEPDPARSRLTCQLKVTDALDGLVVQMPEKQI

>2Fe-2SST2(dsh:Dshi_2731)Dinoroseobacter shibae

MAKITYVEHNGTEHVVDVANGLTVMEGARDNNIPGIEADCGGACACSTCHVYVHPDWVGK

LPGKEAMEEDMLDFAYQPDPVRSRLTCQIKVSEALDGLVVQMPEKQI

>2Fe-2SST2(lmd:METH_05610)Leisingera methylohalidivorans

MAKITYIEHNGTSHVVDVANGLTVMEGARDNNIPGIEADCGGACACSTCHVYIAPDWVEK

LPAKDDMEEDMLDFAYEPDPARSRLTCQIKVSDALDGLVVHMPEKQI

>2Fe-2SST2(red:roselon_00079)Roseibacterium elongatum

MAKITYIEHNGTEHVVDVPNGLTVMEGARDNGIPGIEADCGGACACSTCHVYVHPDWVSK

LPAVDPMEEDMLEFAFQPDTERSRLTCQLKVTDALDGLVVQMPEKQI

>2Fe-2SST2(paby:Ga0080574_TMP4710)Pelagibaca abyssi

MAKITYIEHNGTEHVVDVPTGLTVMEGARDNGIPGIEADCGGACACSTCHVYVHPDWVGK

LPGKEEMEEDMLDFAYEPDPERSRLTCQLKVSDALDGLVVQMPEKQI

>2Fe-2SST2(rsh:Rsph17029_1996)Rhodobacter sphaeroides ATCC 17029

MAKITYVEFNGTEHVIDVAAGLTVMEGARDNGVPGIEADCGGACACSTCHVYVDPAWTDK

LPKKEAMEEDMLDFAWQPDPARSRLTCQIKVSDALDGLKVFIPEKQI

>2Fe-2SST2(rsq:Rsph17025_0944)Rhodobacter sphaeroides ATCC 17025

MAKITYVEHNGTEHVIDVANGLTVMEGARDNGVPGIEADCGGACACSTCHVYVDPAWVGK

LPRKEAMEEDMLDFAWEPDPVRSRLTCQIKVTESLDGLKVFIPEKQI

>2Fe-2SST2(rsk:RSKD131_1681)Rhodobacter sphaeroides KD131

MAKITYVEFNGTEHVIDVAAGLTVMEGARDNGVPGIEADCGGACACSTCHVYVDPAWTDK
LPKKEAMEEDMLDFAWQPDPARSRLTCQIKVSDALDGLKVFIPEKQI
>2Fe-2SST2(jan:Jann_3168)Jannaschia sp. CCS1
MAKITYIEHNGTKHDVEVANGLTVMEGARDNSIPGIEADCGGACACSTCHVYVDPAWVEK
LPAIDPMEEDMLEFAYEPDSARSRLTCQLKVTDELDGLVVQMPEKQI
>2Fe-2SST2(pga:PGA1_c23660)Phaeobacter inhibens DSM 17395
MAKITYVEHNGAEHVVEVANGLTVMEGARDNNIPGIEADCGGACACSTCHVYVDAGWVEK
LPAKDDMEEDMLDFAYEPDPARSRLTCQIKVTDDLNGLVVHMPEKQI
>2Fe-2SST2(pgl:PGA2_c21620)Phaeobacter inhibens 2.10
MAKITYVEHNGAEHVVEVANGLTVMEGARDNNIPGIEADCGGACACSTCHVYVDAGWVEK
LPAKDDMEEDMLDFAYEPDPARSRLTCQIKVTDDLNGLVVHMPEKQI
>2Fe-2SST2(pgd:Gal_01024)Phaeobacter gallaeciensis DSM 26640
MAKITYVEHNGAEHVVEVANGLTVMEGARDNNIPGIEADCGGACACSTCHVYVDAGWVEK
LPAKDDMEEDMLDFAYEPDPARSRLTCQIKVTDDLNGLVVHMPEKQI
>2Fe-2SST2(php:PhaeoP97_02237)Phaeobacter porticola
MAKITYVEHNGAEHVVDVANGLTVMEGARDNNIPGIEADCGGACACSTCHVYVDAAWVEK
LPAKDDMEEDMLDFAYEPDPARSRLTCQIKVSDDLNGLVVHMPEKQI
>2Fe-2SST2(con:TQ29_11980)Confluentimicrobium sp. EMB200-NS6
MAKITYIEHDGTEHTVDVPTGMTVMEGARDNNIPGIDADCGGACACSTCHAYIAPDWVEK
LPKMEAMEEDMLDFAYAPDPARSRLTCQIKVSDDLDGLVVNLPEKQI
>2Fe-2SST2(yan:AYJ57_11300)Yangia sp. CCB-MM3
MAKITYIEFGGTEHVVEVATGMTVMEGARDNGIPGIEADCGGACACSTCHVYVHPDWVEK
LPAKDDMEEDMLDFAFEPDPSRSRLTCQLKVTDALDGLVVQMPEKQI
>2Fe-2SST2(tom:BWR18_04810)Tateyamaria omphalii
MAKITYIEHGGTEHVVDVANGLTVMEGARDNNIPGIEADCGGACACSTCHVYVHPDWVEK
LPAKDDMETDMLDFAYEPDETRSRLTCQLKITDALDGLVVQMPEKQI

>2Fe-2SST2(rsp:RSP_0352)Rhodobacter sphaeroides 2.4.1

MAKITYVEFNGTEHVIDVAAGLTVMEGARDNGVPGIEADCGGACACSTCHVYVDPAWTDK

LPKKEAMEEDMLDFAWQPDPARSRLTCQIKVSDALDGLKVFIPEKQI

>2Fe-2SST2(rmm:ROSMUCSMR3_00986)Roseovarius mucosus

MAKITYIEHNGTRHEVEVANGLTVMEGARDNNIPGIEADCGGACACSTCHVYVDPAWVGK

LPAKDDMEEDMLDFAYQPDPARSRLTCQLKVTEALDGLVVQMPEKQI

>2Fe-2SST2(rde:RD1_1925)Roseobacter denitrificans

MAKITYIEHGGTEHVVEVANGLTVMEGARDNNIPGIEADCGGACACSTCHVYVDPAWVEK

LPGKDDMEEDMLDFAYEPDPARSRLTCQLKVTDALDGLIVQMPEKQI

>2Fe-2SST2(rli:RLO149_c010230)Roseobacter litoralis

MAKITYIEHGGTEHVVEVANGLTVMEGARDNNIPGIEADCGGACACSTCHVYVDPAWIEK

LPGKDDMEEDMLDFAYQPDPARSRLTCQLKVTDALDGLIVQMPEKQI

>2Fe-2SST2(oar:OA238_c07330)Octadecabacter arcticus

MAKITYIEHGGKEHIVEVANGLTVMEGARDNGIPGIEADCGGACACSTCHVYIDAAWADK

VPAKDAMEEDMLDFAYEPDPARSRLTCQLKVTDALDGLIVQMPEKQI

>2Fe-2SST2(otm:OSB_10470)Octadecabacter temperatus

MAKITYIEHGGKEHIVDVANGLTVMEGARDNGIPGIEADCGGACACSTCHVYVDAAWVEK

IPAKDAMEEDMLDFAYEPDAEKSRLTCQLKVTDGLDGLVVRMPEKQI

>2Fe-2SST2(ptp:RCA23_c27570)Planktomarina temperata

MAKITYIEHGGAEHVVEVANGLTVMEGARDNNIPGIEADCGGACACSTCHVYVDSAWVEK

LPAMDGMEEDMLDFAFEPKPGQSRLTCQLRVSDALDGLVVRMPERQI

>2Fe-2SST2(rsu:NHU_00961)Rhodovulum sulfidophilum

MAKITYIEHNGTEHVIDVANGLTVMEGARDNGVPGIDADCGGACACSTCHAYVHPDWVDR

LPPVEDMEADMLDFAYATDPKRSRLTCQIKVAEALDGLIVQLPEKQI

>2Fe-2SST2(suam:BOO69_05855)Sulfitobacter sp. AM1-D1

MAKITYVEHGGKEHVVEVANGMTVMEGARDNNIPGIEADCGGACACSTCHVYVDPAWVDK

LPARDDMEEDMLDFAYEPDAERSRLTCQLKVTDALDGLRVQMPEKQI
>2Fe-2SST2(tpro:Ga0080559_TMP592)Salipiger profundus
MAKITYIEHNGTEHVVEVANGMTVMEGARDNNIPGIDADCGGACACSTCHVYVHPDWVAK
LPERDDMEEDMLDFAYEPDPERSRLTCQLKVSDELDGLIVQMPEMQI
>2Fe-2SST2(lvs:LOKVESSMR4R_00927)Yoonia vestfoldensis
MAKITYVEHGGKEHVVDVANGLTVMEGARDNGIPGIEADCGGACACSTCHVYVDDAWVDK
LPAKDAMEDDMLDFAYQPDPVKSRLTCQLKVSDALDGLRVQMPEKQI
>2Fe-2SST2(pde:Pden_4027)Paracoccus denitrificans
MAKITYIEHNGTVHEIDVKPGMTVMEGARDNGVPGIDADCGGACACSTCHVYVAPEWVDR
LPTRDPMEEDMLDFAWQPDPVRSRLTCQIKVTPELDGLVVNLPERQI
>2Fe-2SST2(pami:JCM7686_1496)Paracoccus aminophilus
MAKITYIEHNGTTHEVDVKPGMTVMEGARDNGIPGIDADCGGACACSTCHVYVAEDWVER
LPEKDPMEEDMLDFAYQPDAKRSRLTCQIKVTPELDGLVVQLPERQI
>2Fe-2SST2(pye:A6J80_18515)Paracoccus yeei
MAKITYIEHNGTAHEIDVKPGMTVMEGARDNGVPGIDADCGGACACSTCHVYVAPEWVDR
LPPKDPMEEDMLDFAWQPDPARSRLTCQIKVTPDLDGLVVQLPERQI
>2Fe-2SST2(kvu:EIO_0828)Ketogulonicigenium vulgare Y25
MARITYVEFNGTEHVVDVPTGLTVMEGARDNGIPGIEADCGGACSCSTCHVYVDPAWVDQ
LPQRDPMEEDMLDFALAPDPVRSRLSCQLRVNDSLDGLRVDMPERQI
>2Fe-2SST2(kvl:KVU_0361)Ketogulonicigenium vulgare WSH-001
MARITYVEFNGTEHVVDVPTGLTVMEGARDNGIPGIEADCGGACSCSTCHVYVDPAWVDQ
LPQRDPMEEDMLDFALAPDPVRSRLSCQLRVNDSLDGLRVDMPERQI
>2Fe-2SST2(kro:BVG79_00616)Ketogulonicigenium robustum
MARITYVEFNGTEHVVDVPTGLTVMEGARDNGIPGIEADCGGACSCSTCHVYVDPAWVAQ
LPERDPMEEDMLDFALEPDPVRSRLSCQLRVTDALDGLRVNMPERQI
>2Fe-2SST2(rcp:RCAP_rcc02404)Rhodobacter capsulatus

MAKIIFIEHNGTRHEVEAKPGLTVMEAARDNGVPGIDADCGGACACSTCHAYVDPAWVDK
LPKALPTETDMIDFAYEPNPATSRLTCQIKVTSLLDGLVVHLPEKQI
>2Fe-2SST2(cmar:IMCC12053_2492)Celeribacter marinus
MAKITYIEFNGTEHVVDVPNGRTVMEGARDNGVPGIDADCGGACACSTCHVYVSPEWVEK
LTPKDAMEEDMLDFAYQPDAARSRLTCQIKVTDAIDGLVVNLPEKQI
>2Fe-2SST2(daa:AKL17_2730)Defluviimonas alba
MAKITYVEHSGTRHEIEVAPGLTVMEGARDNGVPGIEADCGGACACSTCHVYVDAAWLDR
VPPKDAMEEDMLDFAWQPDPARSRLTCQIKVTPDLDGLVVHLPERQI
>2Fe-2SST2(thw:BMG03_07110)Thioclava nitratireducens
MAKITYVEFNGTRHEIDVKPGMTVMEGARDNGVPGIDADCGGACACSTCHVYVDKDWIEK
LPPRDPMEEDMLDFAYEPDPVTSRLTCQIKVTDELDGLIVNLPEKQI
>2Fe-2SST2(10mamo:A6B35_31415)Mesorhizobium amorphae
MIKITYVEHNGREHTVGVPQGGTLMEGARDNGIPGIEADCGGACSCATCHAYIDSAWIEK
LPAKQPMEADMLDFAYEPDATRSRLTCQVRVTEALNGLVLHMPERQA
>2Fe-2SST2(shz:shn_17160)Shinella sp. HZN7
MVQITFVEHDGSRHVVEAAAGMTLMEAARDDDVRGILAECGGACSCSTCHVYVAPEWVDR
LPAISALEQDMLDFAHDPDPARSRLSCQIKLTDTLDGLLVDLPERQG
>2Fe-2SST2(abs:AZOBR_p330135)Azospirillum brasilense Sp245
MAKVTFIEHDGLEQVVDIPTGWTLMQGAVQSGVAGIEGECGGSCACATCHCYVDEALVDS
LPPPSETEEEMLDCTASERRSNSRLSCQIRVSDALDGMVVRLPEAQS
>2Fe-2SST2(abq:ABAZ39_27950)Azospirillum brasilense Az39
MAKVTFIEHDGLEQVVDIPTGWTLMQGAVQSGVAGIEGECGGSCACATCHCYVDEALVDS
LPPPSETEEEMLDCTASERRSNSRLSCQIRVSDALDGIVVRLPEAQS
>2Fe-2SST2(magq:MGMAQ_2658)Magnetospira sp. QH-2
MPKVTYIEHDGAEHHVELPAGWTLMQGATLNGIDGIEGECGGSCGCATCHCYVDEPWLGK
LEPASETEEEMLECTESPRESNSRLSCQIKATPALDGIVVRLPEAQS

>2Fe-2SST2(aol:S58_10680)Bradyrhizobium oligotrophicum
MPRITFIEPDGTVRLVDAEPHESAMQAAKRNGVDGIIGECGGSCICATCHCHVDEAWLDR
VGPAGDIEADLLEFEAADVRPESRLACQIQITEALDGLVLHVAGRSR
>2Fe-2SST2(pgv:SL003B_3177)Polymorphum gilvum
MPKIIFMEPDGSRRDLEVPVGTSLMQAAVQNGVEGIVGECGGSCMCATCHVLVDEAGLAR
LPPKSDSEDEMLEFSVGERHPGSRLGCQITVTAELDGLTVRVPG
>2Fe-2SST2(malg:MALG_02188)Marinovum algicola
MAKITYIEHNGTRHEVDVATGLTVMEGARDNNIPGIEADCGGACACSTCHVYVDKAWTEK
LPPMDAMEEDMLDFAYEPDPERSRLTCQLKVSDDLDGLVVQMPEKQI

Subtype 3

>2Fe-2SST3(bbt:BBta_1591)Bradyrhizobium sp. BTAi1
MTELSAMIDEGGARRFRIRLERPDGTFTFDAASDEYLLYSMIDAGIESPYICEQGWCLAC
AARLVSGKVDRSDALTVYAEDAEAGFLLLCSTKPCSDLILTLDERQTRRDMVQHRIEHNQ
LARAYPPGARLRFRRGRATPRMSSGD
>2Fe-2SST3(aol:S58_10600)Bradyrhizobium oligotrophicum
MTDLSTSTDEGDARRFRVRLERPDGTFTFDAASDEYLLYSMIDAGIDTPYICEQGWCLAC
AARLVSGKVDRSDALTVYPEDAEAGFLLLCSTKPCSDLVLMLDERQTRRDMMQHRIEHNQ
LARAYPPGARLHFRRGRAAPRIDPCTLSKR
>2Fe-2SST3(rde:RD1_2377)Roseobacter denitrificans
MRKHKVTLRNRDNLTFDVGEDEAIIDIVEAAGHVLPIACRYGGCISCAARMISGSVRQPK
GTALNKRQSEAGYVLLCVARPTADCVFDVGVESHTTLYQNPFASAAAASQLARASKE
>2Fe-2SST3(rli:RLO149_c020910)Roseobacter litoralis

MRKHKVTLRNRDNLSFEVGEDEAIIDIVEAAGHVLPIACRYGGCITCAARMISGSVRQPK
GTALNKRQSEAGYVLLCVARPTADCVFDVGVESHTTLYQNPFASAAAASQLARANTE
>2Fe-2SST3(11mamo:A6B35_14570)Mesorhizobium amorphae
MTSFTITLANQGGATFDVDRRKPLLQSLREQGVDLPYGCKYGGCITCAAKMISGNVDQKA
QRALNNRQINNGYIILCVARPLSDCTLEVGVESHDKLYRNPFLDPLAAHELKADIATPLE
DKK
>2Fe-2SST3(apb:SAR116_0299)Candidatus Puniceispirillum marinum
MSMITIANRENATYQVKGRKPLLDELRDQGVDLPYGCKYGGCITCAAKLIDGEIDQRAQV
ALNNWQLNNGYVILCVARAKTDCTLEIGVESHDKLYRNPFLDPLQPHELKADIATPLDAE
K
>2Fe-2SST3(oar:OA238_c13340)Octadecabacter arcticus
MNATRTITIANCGGATYQVEARRPLLDCLREQGVDLPYGCKYGGCITCAAKLTAGEVDQR
RQVALNNRQINNGYVLLCVARAVTDCTLEVGVESHDKLYRNPFIDPLEPHELKADIATPK
ER
>2Fe-2SST3(sil:SPO2377)Ruegeria pomeroyi
MTTHTVTIANREGASFQVNARRPLLEQLRDQGVDLPYGCEYGGCITCAAKLTAGEVDQRR
QVALNNRQIANGYVILCVARATSDITLEIGVESHDTLYRNPFLDPLQPHELKADIATPRP
SIGDALEQTKES
>2Fe-2SST3(ptp:RCA23_c25280)Planktomarina temperata
MIVTLSNRDGAQYKVDPRRPLLDSLRDQGVDLPYGCKYGGCITCAAKLTAGEVDQRRQVA
LNNRQINQGYVILCVARPMSDITLEIGVESHDRLYRNPFLDPLQSHELKADIAVPKEV
>2Fe-2SST3(rle:pRL100075)Rhizobium leguminosarum bv. viciae 3841
MTGEAVLAPFKVEFAKQGRTIDVTSDQTVLSCAKKAGIKIPSSCSNGLCGTCKSKLVSGT
INMHHNGGIRQREIDAGFFLPCCSKPLTDLIIER
>2Fe-2SST3(lmd:METH_03515)Leisingera methylohalidivorans
MLDAALENAMDAPYACKAGVCSTCRCKVLEGEVEMVANHALEDYEVEKGYVLSCQAYPVT

DNVVVDYDQ

Subtype 4

>2Fe-2SST4(azc:AZC_3424)Azorhizobium caulinodans azc

MKEVSMTTVTISPGGEVLEAAEGMSLLEVLLKGDIKIPHKCDGKASCGSCHIFVQEGRKG

LTKIAREENEKLDSIVGVGSKSRLACQAKVLGTENIKIELLGFGSGL

>2Fe-2SST4(msc:BN69_2649)Methylocystis sp. SC2

MPTVTIMPSGKTVDAAEGATLLQIIMSSGEEINHKCEGKAQCGSCHIYVHEGRKGISKIA

REENERLDTIVGIGSKSRLACQAKILGTENIKVELLGFASGL

>2Fe-2SST4(mbry:B1812_07990)Methylocystis bryophila

MATVTIMPSGKSVEAAEGATLLEIILSAGEGINHKCEGKAQCGSCHIFVQEGRKGVSKIG

REENEKLDTIVGVGSKSRLACQTKVLGTENIKLELLGFSSGL

>2Fe-2SST4(brs:S23_46150)Bradyrhizobium sp. S23321

MTTLTVLPEGKTIEVSEGTTLLAALLGAEIAVPHKCEGQAKCGSCHIYVQEGRKGLSKIA

RLENEKLDSIVGVGSKSRLACQATVLGTEDVKIELLGFGSGL

>2Fe-2SST4(bbt:BBta_5894)Bradyrhizobium sp. BTAi1

MATLTVMPSGKTIEVGEGTTILAALLGAEVEIPHKCEGQAKCGSCHIFLQEGRKGVSKVT

RLENEKLDTIVGVGSKSRLACQATILGTENVKIELLGFGSGL

>2Fe-2SST4(bra:BRADO5410)Bradyrhizobium sp. ORS 278

MATLTVMPSGKTIEVGEGTTILAALLAADVEIPHKCEGQAKCGSCHIFLQEGRKGVSKVS

KLENEKLDSIVGVGSKSRLACQATILGTENVKIELLGFGSGL

>2Fe-2SST4(aol:S58_22960)Bradyrhizobium oligotrophicum

MATLTVMPSGKTIEVGEGTTILAALLGAEVEIPHKCEGQAKCGSCHIFLQEGRKGVSKVS

KLENEKLDSIVGVGSKSRLACQATILGTENVKIELLGFGSGL

>2Fe-2SST4(bid:Bind_1905)Beijerinckia indica

MPTVTIKPSGKTQVAAEGSILIDAIIAAGEPIEVKCNRDAKCESCHVFILEGKKSLAKMG

RPESEKLDSMVGVASKSRLACQCVLGTENVTVELLGALSG

>2Fe-2SST4(mbry:B1812_10000)Methylocystis bryophila

MPTLTLLPSGKTTDAEPGTRLLDAILATGEKIVSKCGGEAKCGECHVFIQEGRKSLSKMQ

RAENEKLDTIPGVGSKSRLACQTLFGTENVTVELLGFGSGL

>2Fe-2SST4(sphi:TS85_14400)Sphingomonas sp. WHSC-8

MTTVTIMPSGKTVDAVVGSTLLAAILAADSKIQHNCDGKATCGTCHIFVLEGRKGVSKIG

REENEKLDSIVGVGSKSRLACQATLLGTEDVMLEVLSFR

>2Fe-2SST4(msl:Msil_3636)Methylocella silvestris

MPLLTIQPSGKTIEAAEGTSLLDALLAAGEKIVSKCGGNAKCGQCHVFVQEGRKTISKMT

RVENEMLDTIIGVGSKSRLACQAMFGTEPVTVEVLSFV

>2Fe-2SST4(ster:AOA14_03605)Sphingopyxis terrae

MPEIIIVLRDGSYRIIDTDPEYSLMEAIRDAGVDEMLAMCGGCCSCATCHVYIEESGGDL

SEPSADEDDLLDSTGCRTSQSRLACQVRLAESTIKRARIAPED

Subtype 5

>2Fe-2SST5(pla:Plav_1783)Parvibaculum lavamentivorans

MPTIIFIEPDGVEYPIAVKSGTTARDAAVNNSVPGIDGDCGGECACATCHVHVDAAWMAR

TGMAEPGGMEANLLQFAEGSTENSRLACQITMTDDLDGLMLRIPDGQH

>2Fe-2SST5(npp:PP1Y_AT31173)Novosphingobium sp. PP1Y

MIKVTFVAHDGHRFPVEIGEGLTAREAALFNDVPGIDGDCGGQCACATCHVQVDPAWIER

VGRLADDSMEADLLQFAEGTTAESRLACQIRLDAGLDGLVLHVPEQQY

>2Fe-2SST5(sphk:SKP52_24465)Sphingopyxis fribergensis

MIKVTFVSADGTRRTVEIDEGLSAREAALFNSVPGIDGDCGGVCACATCHVHVDLAWMDR

VGPPEEGGMESDLIQFAEGTTETSRLACQISMVPELDGLVLHLPELQH

>2Fe-2SST5(sphk:SKP52_13375)Sphingopyxis fribergensis

MVKVTFVSSDGTRREVEIAEGETAREAALFNDVPGIDGDCGGVCACATCHVHVDPAWIDR
VGRLVEGAAEAELLQFAEGANEYSRLACQIPMVEGVEGLILHVPEQQY
>2Fe-2SST5(ster:AOA14_00650)Sphingopyxis terrae
MVKVTFVSSDGTRREVEIAEGETAREAALFNDVPGIDGDCGGVCACATCHVHVDPAWIDR
VGRLVEGAAEAELLQFAEGANEYSRLACQIPMVEGVEGLILHVPEQQY
>2Fe-2SST5(sgi:SGRAN_1596)Sphingopyxis granuli
MIKITFVASDGERREVEIEEGETAREAALYNDVPGIDGDCGGVCACATCHVHVDPEWIDK
VGRLMHDGMEADLLQFAEGTTEYSRLACQIPMKPMLDGLVLHLPEQQY
>2Fe-2SST5(blas:BSY18_3827)Blastomonas sp. RAC04
MVKVTFVAHDGRRFPVEIGSGMTAREAALFNDVPGIDGDCGGQCACATCHVHVDPRWIGR
VGCLVDGSMEADLLQFVEGRTEESRLACQIKLDERLDGLVLYVPEQQY
>2Fe-2SST5(eli:ELI_14940)Erythrobacter litoralis HTCC2594
MIKITFVASDGERREVEIEEGETAREAALYNDVPGIDGDCGGVCACATCHVHVDPEWIDK
VGRLMHDGMEADLLQFAEGTTEYSRLACQIPMKPMLDGLVLHLPEQQY
>2Fe-2SST5(nar:Saro_3658)Novosphingobium aromaticivorans
MAKVTFVQPDGSQRTCVNFEGMTLMQLAVGNLVDGIDALCGGMMQCATCHCWIDPEWIGR
TGMAGPDERAMLEAIEGVEIRPESRLSCQVQLGEELDGLVVRIPPEQPGV
>2Fe-2SST5(npp:PP1Y_Mpl3486) Novosphingobium sp. PP1Y
MAKVTFIQPDGTPRECVNFEGMTLMQLGVANLVEGIDALCGGMMQCATCHCWIDPAWIEI
TGRAGEDERAMLESIEGVEIRPESRLTCQIQLGEELDGLVVRVPPEQPGV
>2Fe-2SST5(sphg:AZE99_00825)Sphingorhabdus sp. M41
MPKVTYISSDGESRTLDVAVGETVMRGALDNDVDGIVAECGGGLACATCHCYVDAAWAAQ
VGTPTSQDEIDMLEMAAAEIRDTSRLSCQIEISAELDGLVVHLPEEQY
>2Fe-2SST5(blas:BSY18_806)Blastomonas sp. RAC04
MPAVTYVEFDGAEHAIDLALGENVMRGALHNDLPGIVGECGGGLACATCHCYVDDAWTER
VGGPASREESEMLESTAAPIKPSSRLSCQIVMSAELDGLIVHLPEAQY

Subtype 6

>2Fe-2SST6(yan:AYJ57_00160)Yangia sp. CCB-MM3

MTKGSLTFTDVDLTVTVPAGTRIIEISEKVGAGIVFGCREGDCGTCITHVLEGSEHLSDP

SALELRVLRENLAGQSDRLACQCQVLGGAVKVKPG

>2Fe-2SST6(rhc:RGUI_2054)Rhodovulum sp. P5

MATAKLTFADVALTVNVPSGTRIIEISEKIGSGITYGCREGECGTCITRIVEGMENLSQP

SALEERVLKDNMAGHDDRLACQCQVLGGTVKVRPG

>2Fe-2SST6(daa:AKL17_1542)Defluviimonas alba

MEKATLTFTDVATTVTVPAGTRIIEMSEKVGSGITYGCREGECGTCLTHVIEGAENLSEP

SVLELRVLAENMAGRKDRLACQCRILGGAAKVRPG

>2Fe-2SST6(rsp:RSP_3190)Rhodobacter sphaeroides 2.4.1

MEKATLTFTDVAITVNVPPGSRIIEMSEKVGSGITYGCREGECGTCVTHIIEGSENLSDP

SALELRVLSENMAGKDDRLACQCRVLSGAVKVRPG

>2Fe-2SST6(rsh:Rsph17029_3929)Rhodobacter sphaeroides ATCC 17029

MEKATLTFTDVAITVNVPPGSRIIEMSEKVGSGITYGCREGECGTCVTHIIEGSENLSDP

SALELRVLSENMAGKDDRLACQCRVLSGAVKVRPG

>2Fe-2SST6(rsk:RSKD131_3366)Rhodobacter sphaeroides KD131

MEKATLTFTDVAITVNVPPGSRIIEMSEKVGSGITYGCREGECGTCVTHIIEGSENLSDP

SALELRVLSENMAGKDDRLACQCRVLSGAVKVRPG

>2Fe-2SST6(cid:P73_2169)Celeribacter indicus

MDKISITFIPNGNKTVSVDPGSNLLRASLREKGGIPFRCGGGFCGTCRCRIVEGREHADD

VKAKERRHLSEEDVANGFRMACQTFVNGDVKISWG

>2Fe-2SST6(rcp:RCAP_rcc03285)Rhodobacter capsulatus

MDKATLTFTDVSITVNVPTGTRIIEMSEKVGSGITYGCREGECGTCMTHILEGSENLSEP

TALEMRVLEENLGGKDDRLACQCRVLGGAVKVRPA

>2Fe-2SST6(aak:AA2016_0331)Aminobacter aminovorans

MINITFITNDNKQVSAPENSNLLRVSLREKGGIPFKCGGGLCGTCKCRIETGIENTDAVK

PKERNHLTSDDLAAGFRMACQTFVLGDVAVSWVPKGR

>2Fe-2SST6(psin:CAK95_12430)Pseudorhodoplanes sinuspersici

MLVSAPGNSNLLRVSLREKGGIPFKCGGGLCGTCKCRIEKGLEHTDTVKAKERKHLTEEQ

IKEGYRMACQTFVNGDVSVSW

>2Fe-2SST6(psin:CAK95_12440)Pseudorhodoplanes sinuspersici

MPKVVLHRDGQVYQDEVKDNTNLVVRAGIKQFPYPNLRYGCGMGKCAKCACRILKGGEHL

PAPNWKEKKQLGAVRLEQGYRLICQLWLNHDIELAQDLQPLEPAAPASA

Subtype 7

>2Fe-2SST7(bid:Bind_3149)Beijerinckia indica

MADITFASPLLPKNKTVYGIAGDTHTLLAVARDHRIPVPFNCEDGDCGSCLIKVTVLDGK

QPMGSTLSEKEKFTLAAHGKLSKEAKELAEIADIPPQYRLACQYIVRDEPILVEFSGEPG

VEIDPARHKHERAALNDIETTGAEDREYAESVKA

>2Fe-2SST7(mgy:MGMSRv2__0351)Magnetospirillum gryphiswaldense MSR-1 v2

MANVTFSGPTLEKNVTVYAVAGANGTLLAVAKANNIKIPFECQDGECGSCLIKVTHLGDN

APKAIHLTDKEKLTLSVNGKLTKELLAKTEVEDMPPPYRLACQYIVRDEDILVEFSGEAG

VEIDLRR

>2Fe-2SST7(msl:Msil_3778)Methylocella silvestris

MPVVTFTSETLHRDVRAYATAGDTSTVLSVALAQGVKIPHDCRDGECGSCLIEVKYVEGK

PKMAIALTEKEKIKLRELGKITAQQIQDAETNDIAPPYRLACQFIVREEEIIIHFTGEPA

GA

>2Fe-2SST7(rsu:NHU_03481)Rhodovulum sulfidophilum

MAIITFTSPQMHKDKTVYGVAGDTKTILALAEDNRIPIPFDCRDGNCGSCLIEVSYDNPA

DKKAIMLTEKEKQKLKELGKLTQQEIDDAEVRDMPPRYRLACQFIARDEDVTVSFSGMPV

GSD

>2Fe-2SST7(con:TQ29_02130)Confluentimicrobium sp. EMB200-NS6

MATITFSSPIMKRDKTVYAVAGNTDTILGLAEEHGIPIPFECRDGNCGSCLIEVTYLEDK

PKMAVALTEKEKARLKELKKVTQKEITEAEVNDLPPHYRLACQYIARHEDIRITFTGEPG

GA

>2Fe-2SST7(rcp:RCAP_rcc00573)Rhodobacter capsulatus

MPNITFTSPIMKKDKTIYAVAGNTATILALAKEHAIPIPFECGDGDCASCLIEVTHLDNK

PAMAMMLTEKEKARLKELQMITAEEIEAAEVSDLPPRFRLACQFIPRDEDVMVHFTGTPG

GSV

>2Fe-2SST7(daa:AKL17_1546)Defluviimonas alba

MPNITFHSSIMKKDKTIYAVAGNTATLLALARAHDIPIPFECGDGICGSCLIEVTLLADK

PLMGMALTEKEKARLKELQKITPAELEQAEVNDIPPRFRMACQFVPRDEDVLVSFTGTPG

GSA

>2Fe-2SST7(yan:AYJ57_00175)Yangia sp. CCB-MM3

MANITFQSPIMQRDKTVYAVAGNTATILALAKEHEIPIPFDCGDGDCGSCLIEVTSLEDK

PAMGLALTEKEKARLKELSKLTPAEIENAEVNDMPPRYRLACQFIARNEDVLVRFSGAPG

GSD

Subtype 8

>2Fe-2SST8(nar:Saro_3849)Novosphingobium aromaticivorans

MRTETHQIRIVGGGQFACPEGERVLIAMERSGGNDIGVGCRGGGCGFCVVRVVEGEYRTG
KMSTAKVSVADQAKGYVLACRLYPLNDLVIEIG

>2Fe-2SST8(cna:AB433_18105)Croceicoccus naphthovorans

MARTVTGLYQIRVVGGGEFTCGGDERVLLAMERCGAGDIGVGCRGGGCGICRVKVVEGDY
RTGKMSAAKVSDSDLQAGYALACRLFPAGNLVIAVE

>2Fe-2SST8(syb:TZ53_09045)Sphingobium sp. YBL2

MDDRDVEGLTQAAPFQIEIGGVRFPCRADQSLLAAMIASGRKALAVGCRSGGCGVCRIRI
QAGRCSTGHMNRAVVSAADEQAGIVLACRAYPSSDIRAVPLPRASFGAAPVGRAA

>2Fe-2SST8(msl:Msil_1473)Methylocella silvestris

MASERFTLTLEGHGASSGYADERVLVALERAQGFGQIKNMPCRLPVGCRRGGCGICRVRV
LAGAYRRDPMSRTHVSVEDEGAGLVLACCIYPLSDLSLRLEPPAAVKGMGQKAIQQG

>2Fe-2SST8(magq:MGMAQ_0645)Magnetospira sp. QH-2

MAHSRHSGKFAIRVEGDPRTYFCAADQKVIAALEECGQIGIRVGCREGGCGVCRVRVVSG
EYETGKMSRAHVSEEQESQGFALSCRLFPRSNLHLEAAGIRTGSGDRET

>2Fe-2SST8(ata:AWN88_11340)Agrobacterium tumefaciens S33

MALPRSIHLPQIDRSIAVAEGETVLQAALAAGIAYPHGCRMGRCGACKSRLVSGEVDLLK
HTPFSLTEEEKADGLTLACRAIPLSDVVIGWLNGADALSDSDADLLSTPER

>2Fe-2SST8(sphk:SKP52_20385)Sphingopyxis fribergensis

MTRTVDIHQAGRAIAVGDGQTILEAALDAGIAYPHGCRSGRCGTCKSRLVAGEVDMLPHT
CFALTPEEKAQGLILACRAQPLTNIEVAWLGKEDEAVAHPARRVKTDVAAPDDATRANTE
PPFQYAARQAGGKTAS

Subtype 9

>2Fe-2SST9(aak:AA2016_0333)Aminobacter aminovorans

MPTVVIHSKGEAFSGEVKEDTNLVVRAGIKQFPYPHLAYKCGMGKCGTCASRILAGAEHL

PPPNWKEKKQLGDKIDEGFRLVCQLWIKNDIELRQEKVTEQA

>2Fe-2SST9(cid:P73_2167)Celeribacter indicus

MPQEGGAASASSEAGNLMPKVILHKGGEVFEGEVDPNTNLVVRAGIKKFPFPNLAYKCGM

GKCSTCTCRILRGGDHLEAPNWKEKRQLGEKLEEGYRLACQLWVSEDIELTQDL

Subtype 10

>2Fe-2SST10(sbd:ATN00_07810)Sphingobium baderi

MQIIFRNAEAGERIVMASEGDTLMHVALANDIIGIPGDCGGQCACGTCHVYVDPAWVERV

GRVEPGSTEETMIFGAPVDARPNSRLACQVNLIAIHDGLIVEIPEGQ

>2Fe-2SST10(anh:A6F65_02151)Altererythrobacter namhicola

MPTVTVLPADIAMETQPGETLLGAAQRLGYTWPTVCGGEGQCRTCYAVIEQGEDALSPVT

ALEEEASPALAIVARRAGKPVRLACQAVPTGDITVQRSGVRKS

Subtype 11

>2Fe-2SST11(hmc:HYPMC_3657)Hyphomicrobium sp. MC1

MATLTIMPADKSIEVADGTTLLAAILSSGIDFPHNCEDQGCRGGCHVFVQEGRKGLSKIA

REENEKLDSIVGVGSKSRLACQAKVLGTENIKVELLGFGSGL

Subtype 12

>2Fe-2SST12(psin:CAK95_14295)Pseudorhodoplanes sinuspersici

MKWACEIDREKIAFAAVDPQSACRIHTAGRWSFRLQEPSPGVLAIAQKSVSVPDNCSRNS

NGPFVVKLARSQRELLWNSDERDLLSLSEKNGVELLNGCRTGQCESCLVKVLSGQVAHLA

PVELEEPGTCLTCCAVPLSDLVLDA

Subtype 13

>2Fe-2SST13(nar:Saro_0183)Novosphingobium aromaticivorans

MIASREEIAQAERALELLGGFAPKRHILLCAGPEKDKCAPRAVGDEAWNYLKKRLGELKL

GGAQGVLRNKVGCLRVCIAGPVAVVYPDNVWYHSCTPPVLERIIQEHVIGGVPVEDYRLR

QPAASLAE

Subtype 14

>2Fe-2SST14(azc:AZC_1282)Azorhizobium caulinodans

MDAVKAALHQLGVPNSQVKTEGFGTDRRDPSKKAQKLGKVIATVSFRESHLSAAAREGMT

LLDVADESGVFIDSACRSGTCGVKLTSGKVRLGTDDALSDEERAQGYILACQAQPDGDVA

LDV

Subtype 15

>2Fe-2SST15(mln:A9174_10550)Mesorhizobium loti NZP2037

MPEEAIFTETFTSLPSDQGPGADEPVTITFARSGRTVMAKRGEGMIRAAEANGIAVDYSC

RTGERGSCRCKLLAGEVEMPEGTALTSKERKAGLILACVARPISDAVTIDL

Subtype 16

>2Fe-2SST16(bid:Bind_0407)Beijerinckia indica

MPIVTIKPQDKTQEGAEGTTLREVIIAAGIPLNECTCAKGYFDNCHAFITEGKKSLGKAT

REENERLDQIVGVGSKSRLICEAKLGPENVTVELLGALSG

Subtype 17

>2Fe-2SST17(con:TQ29_00555)Confluentimicrobium sp. EMB200-NS6

MPEITFVTTAGEEITKEIPVGLSLMRGAVEAGINDILAECGGVASCGTCKIILDEANAGR

IPPADAFEGSMLEDDPEGWRLSCQIIVDDTLEGLVVRLAPTEY

Subtype 18

>2Fe-2SST18(con:TQ29_17640)Confluentimicrobium sp. EMB200-NS6

MPKITYEQPDGTRAELEVEEGMSVMRGAVDNGIDGIVAECGGACACATCQVVIGAEWRDK

LPAPGVLEEAMLEDVEGDDRRLSCQIEVTAELDGMVVKIPATQY

>2Fe-2SST18(sphk:SKP52_00895)Sphingopyxis fribergensis

MPTLTVINRAGNAQQLEAGSGLSVMEVIRDAGYDELLALCGGCCSCATCHVYVEGDVDPG

VPGNDENDLLDSSDHRTERSRLACQIRFADDLAGLTIRIAPED

Subtype 19

>2Fe-2SST19(pla:Plav_2523)Parvibaculum lavamentivorans

MRIILIDTEGVEHTLEAAEGWRVMEIIRDYGFPIVAECGGACACGTCQVEVDPDWAAKLH

EPREEELDMLDQNYGGEWSRLSCQLLMNAKLDGLRVRLADIAIRKAA

Subtype 26

>2Fe-2SST26(bja:blr1765)Bradyrhizobium diazoefficiens USDA 110

MNSEKEFHLPQLYRHHVFACNTQRPPNHPHGSCGASGAQALWDRMGKAIEAQGLDDIGFATAGCLGFCNSGPLLVVYPDGVWYRATTPEDVDEIVISHLKHGQRVDRLVIVLKRS

>2Fe-2SST26(bjp:RN69_39120)Bradyrhizobium japoniCum E109

MNSEKEFHLPQLYRHHVFACNTQRPPNHPHGSCGASGAQALWDRMGKAIEAQGLDDIGFATAGCLGFCNSGPLLVVYPDGVWYRATTPEDVDEIVISHLKHGQRVDRLVIVLKRS

>2Fe-2SST26(bra:BRADO5398)Bradyrhizobium sp. ORS 278

MSDGGVAEAEDEFEIPQLYKYHAFVCLTQRPPGHPRGSCGALGVQPLWDRLTKTIEAQRLTDVGVTAAGCFGFCSAGPMMVVYPDGIWYRPTKPEDIDEIVESHFKQGKRVDRLVM
VLKR

>2Fe-2SST26(bbt:BBta_5882)Bradyrhizobium sp. BTAi1

MSETDVAEADDAFELPQLYKYHAFVCLTQRPPGHPRGSCGALGVQPLWDRLTKTIEAQRLTDIGVTAAGCFGFCSAGPMMVVYPDGIWYRPTKPEDIDEIVESHFKQGKRVDRLVM
VLKR

>2Fe-2SST26(brs:S23_46050)Bradyrhizobium sp. S23321

MSDGVNEEEFQLPQLYRHHVFACHTQRPPGHPRGSCGAAGGAALWERFGKAIEAQRLTDIGFTASGCLGFCAAGPLMVIYPDGVWYRPTTPEDVDEIVESHLKQGKRVDRLVMVLA
RS

>2Fe-2SST26(aol:S58_23070)Bradyrhizobium oligotrophiCum

MSDSEVAEADDEFEVPQLYKYHAFVCLTQRPPGHPRGSCGALGVQPLWDRLTKTIESQRLTDIGVTAAGCFGFCSAGPMMVVYPDGIWYRPTKPEDIDEIVESHFKQGKRVDRLVM
VLKR

>2Fe-2SST26(brC:BCCGELA001_31070)Bradyrhizobium sp. CCGE-LA001

MNAEDELELPQLYRHHVFACHTERPPTHPHGSCAASGALALWDRMGKAIEAQALSDVALTPAGCLGFCNTGPLMVVYPDGVWYRPTTAEDIDEIVESHLRKGRRVDRLVIVLKRS

>2Fe-2SST26(biC:LMTR13_26520)Bradyrhizobium iCense

MNAETEFQLPQLYRHHVFACHVQRPPTHPHGSCGAAGAQVLWDRMSKAIEAQGLTDIGFTAAGCLGFCNAGPLMVVYPDGVWYRPTTADDIDEIIESHLKEGKRVDRLVMVLKRS

>2Fe-2SST26(xau:Xaut_2332)XanthobaCter autotrophiCus

MSEAAESEVLDIPQVYTHHVFCCFTQRPPQHPRGSCGANGAAPLWDRLAKKLEASGRRDIAMTSAGCLSFCQAGPIMVVYPQGIWYTPKSPEDIDEIVTSHLLGGKPVERLIIVPR
I

>2Fe-2SST26(azC:AZC_0303) Azorhizobium Caulinodans

MTETATLSLTDVPQVYKRHVFACFQQRPPTHPRGSCGAAGAQPLWERLGKQIEATGQRDIVMTATGCMGFCQAGPIMVVYPEGVWYQPRTPEDVDEIVTTHLVGGTLVERLVIVPR
I

>2Fe-2SST26(bid:Bind_3574) BeijerinCkia indiCa

MTLEVPQVFKYHIFTCSTKRPAGHPRGSCMERGAQPLWERLGQKLEAKQLPGVSMTMTGCLSFCQAGPLMVVYPEGIWYHPEKPEDIDEIVQSHFVEGNPVERLIIVPQR

>2Fe-2SST26(msl:Msil_1738) MethyloCella silvestris

MSSVAELDLPVVFKYHVIACFTKRPPGHPRGSCMESGAQPLWEHLQKKVEAQRLTDVCVTASGCLSFCRAGPLMVVYPEGVWYHPETVDDIDEIVDRHFGAGEPVERLIIVPKV

>2Fe-2SST26(hmC:HYPMC_3647) HyphomiCrobium sp. MC1

MTTTLDDDVVDVPQVYKHHIFACFTQRPPNHPRGSCGAVGAQPLWDYLSKALEAEHLSDVGFTAAGCLGFCRTGPLMVVYPEGIWYRPSTAEDIDEIVQSHFKQGKRVDRLVMVLS
KN

>2Fe-2SST26(hmC:HYPMC_3564) HyphomiCrobium sp. MC1

MEVVSGNGGKSATAQSSAANDSEPVYRIHVFCCVNERPPTHRRGSCGGKGSRQLCDYMCRLGMATGVRRIRINHAGCMNVCEHGPVLVIYPEGVWYRFETQEDVEEILRSHIVAGR
KVER
LALSIDPTTLHG

>2Fe-2SST26(msC:BN69_2661) MethyloCystis sp. SC2 msC

MSDTPEEEFELPQLYKRHVFACFTQRPPNHPRGSCGSLGAQPLWERLGKRVEADGGGEIGFTSAGCMGFCSAGPLMVVYPEGVWYRPTTAEDIDEIYDSHLKGGKRVDRLVMILSR

>2Fe-2SST26(mbry:B1812_07930) MethyloCystis bryophila

MSDASEEEFEVPQLYKRHVFACFTQRPPTHPRGSCGANGAQPLWDRLGKLVEADGSGEIGFTAAGCMGFCSAGPLMVVYPEGIWYRPTTPEDVDEIYSSHLKGGKRVDRLVMILTR
>2Fe-2SST26(mgy:MGMSRv2__3321) Magnetospirillum gryphiswaldense MSR-1 v2

MSVSTDPELFYRIHAFICTNRRADDHPRGSCAARGSEPLRDYLKAAAKKKGVGGVRVNAAGCLDRCELGPVLVIYPEGIWYGFNSREDIDEIIDTHLLGGGRVTRLMLTPDQK
>2Fe-2SST26(magq:MGMAQ_3806) Magnetospira sp. QH-2

MSEAPYYRSHVFVCSNQRVEGHPRGCCADKDSASLRTYLKVKTKQLELNDVRINNAGCLDRCENGPVMVIYPEGVWYKVATRADMDEILESHIQGGNRVEHLMLSNED

>2Fe-2SST26(azl:AZL_a04200) Azospirillum sp. B510

MPLDIPQTFRHHVFCCAQQRPPGHPRGSCAAKGAHPLWQRLDQKIQGQGLTDIGMAMTGCLGFCSAGPLMVVYPEGIWYRPETPEDIDEIVDSHLVNDTPVERLVMVLTR

>2Fe-2SST26(abs:AZOBR_140175) Azospirillum brasilense Sp245

MSLDLPQVFRHHVFCCAQQRPPGHPRGSCAAKNAHPLWEQLGQRIQAKGLLDVGMAWTGCLGFCSAGPLMVVYPEGLWYRPETPEDIDEIVDSHLVNNTPVERLVMVLTR

>2Fe-2SST26(abq:ABAZ39_05635) Azospirillum brasilense Az39

MSLDLPQVFRHHVFCCAQQRPPGHPRGSCAAKNAHPLWEQLGQRIQAKGLLDVGMAWTGCLGFCSAGPLMVVYPEGLWYRPETPADIDEIVDSHLVNNTPVERLVMVLTR

Subtype 27

>2Fe-2SST27(sphi:TS85_14385) Sphingomonas sp. WHSC-8

MELPEELPQLYKRHIFACHTQRPPQHPRGSCGAAGAGPLWERLGKRIEALDLTGEVGFTASGCLGFCSAGPLIVIYPEGVWYKPQSEADIDLIVEHHLQQDRLVEDLVVVLSR
>2Fe-2SST27(azl:AZL_000100) Azospirillum sp. B510

MTDPNPYFEAHVFCCTNRRPDGHKRGSCAAQGSEKLRDYMKARARELGFDGKVRINSAGCLDRCELGPTLVIYPEGVWYSYHSTADIDDILQTHLVEGRRVERLMLTTEQRELRPD
QRG

>2Fe-2SST27(abs:AZOBR_10399) Azospirillum brasilense Sp245

MTDPKPYFEAHVFSCTNRRPDGHKRGSCAARGSEKLRDYMKARARELGLDGRVRINAAGCLDRCELGPVLVVYPEGVWYTYRSFADVDAILETHVIGGGRVERLMLKPEQKELLPE
QVG

>2Fe-2SST27(abq:ABAZ39_14075) Azospirillum brasilense Az39

MTDPKPYFEAHVFSCTNRRPDGNKRGSCAARGSEKLRDYMKARARELGLDGRVRINAAGCLDRCELGPVLVVYPEGVWYTYHSFADVDAILETHVIGGGRVERLMLKPEQKELLPE
QVG

Subtype 28

>2Fe-2SST28(elq:Ga0102493_11923)ErythrobaCter litoralis DSM 8509

MSDNELLSARSSLARHYESAPARPDSAPVERHIFLCALSDKAKCCSREEGEAAWAFLKTRLKERGLVGPRRTADHPKGAGGGVQRTKADCLQICGKGPIAVVWPDGVWYHSCMPQA
LERIIDEHLVGGVPVEEYRLSPLGED

>2Fe-2SST28(pns:A9D12_04600) PorphyrobaCter neustonensis

MLCARTNLARHFGDPQKGTAIARHIMLCAISEKQKCCSREAGEEAWGFLKSRLKELGLVGPQRREDSKRGAGGGVQRTKADCLQLCAAGPIAVVWPDGVWYHSCSPAVLERVIQEH
LIGGVPVEDFRLKPAV

>2Fe-2SST28(porl:BG023_11413) PorphyrobaCter sp. LM 6

MNREELLCAQNSLAKHYGDAAKGTAIQRHIMLCALSEKQKCCSREAGEESWGFLKTRLKELGLVGPLRDANSPRGAGGGVQRTKADCLQICAAGPVAVVWPDGVWYHSCSPQVLER
IIQEHLIGGEPVEDFRLTPA

Subtype 29

>2Fe-2SST29(eli:ELI_12930) ErythrobaCter litoralis HTCC2594

MTSEKKLDKARRALRSIAGERVERHIFLCALSEKQKCCKLDEGERAWKYLKSRMKELGLPKAGVAQRTKADCLQVCAAGPVAVVYPDQVWYHSCNEDVLERIIQQHLIGGEPVEEF
RLRSPV

>2Fe-2SST29(aay:WYH_02285) AltererythrobaCter atlantiCus

MNKDSPEQPDLGKAQRALGKIGGEAIDRHIFLCGLSEKQECCRREVGERSWKYLKRRLKELGLAGKGGIQRSKADCLQICEAGPIAVIWPDRVWYHSCTEEVLERIIQEHLIGGRP
VEDYRLRSSESSSGSITASS
>2Fe-2SST29(Cna:AB433_16560) CroCeiCoCCus naphthovorans Can

MGGGAEFTRHIFLCADQTKPKCCPKKVGLESWDHLKKRLAELGLDRSGGVMRTKANCLRLCVAGPLAVVYPDGVWYHSCKPEVLDRIIDEHLIGGVPVEEYRLTVEGES

Subtype 30

>2Fe-2SST30(amx:AM2010_2385) AltererythrobaCter marensis

MTSPRDLARAQRALGAIGGETVARHIFLCAVSEKQKCCRREEGERAWKYLKKRLKDLRLAGTTVQRTKADCLQVCETGPIAVVWPDQVWYHSCTEDVLERIIQQHLIGGVPVEDYR
LHSAPGG

>2Fe-2SST30(ado:A6F68_00497) AltererythrobaCter dongtanensis

MTPEPDIAKARAALAKIGGERVERQIFLCAMSEKQKCCSREEGERAWKFLKRRLKELGLVGRTVQRAKADCLQVCEAGPVAVVWPDRVWYHSCNEAALERIIQEHLIGGVPVEDLR
LREL

Subtype 31
>2Fe-2SST31(bid:Bind_1539) BeijerinCkia indiCa (Thioredoxin-like)

MALKDIFGQPAEARRLVVCVGPCCNKTGEAEVFLEELRQTMLAEGLDEAMVGTASCMRRACLGKCTGEPLAYIHPEEVWYHQLSPANLLRILREHLLGLRPVAELILPEE

Subtype 32

>2Fe-2SST32(aep:AMC99_02527) AltererythrobaCter epoxidivorans (Thioredoxin-like)

MSRSGKLEEAQKALTKIGGETIERHIFLCGISDKQKCCRREEGERAWKYLKKRLKQLKLAGPLREDGGGIQRTKADCLQICAAGPIALVWPDRVWYHSADEEVLERIIQDHLIGGK
VVEEYRLKTPPAGD

Subtype 33

>2Fe-2SST33(anh:A6F65_00445) AltererythrobaCter namhiCola (Thioredoxin-like)

MSEKKLGKAEKAFAKIGGGAMSRHIFLCAISDKQKC**C**KRAEGEAAWKFLKRRMKELGLTSPAKAPDGMVVQRTKADCLQVCEAGPIAVVWPDGVWYHSCDPDALERIIQQHLIGGE
PVEDLRLRPPREA

Subtype 34

>2Fe-2SST34(msC:BN69_0624) MethyloCystis sp. SC2

MTETAIKPFRLHVFV**C**VNTRDGRSA**C**EDHGAKEALAKVKEALKALPFAKRDGVRLTQSG**C**LGQ**C**EHGPVAVVYPGGKWISYASADELIRFVLEAIEAPD

Subtype 35

>2Fe-2SST35(mgy:MGMSRv2__1348) Magnetospirillum gryphiswaldense MSR-1 v2

MSHKYHVFVCTTSRPAGHPRGCCTSKGGGTAMFDQMMARFQKGMLWEKGVSLAQASCLGFCGNGPLMVVYPEGVWYQPVEAADYDEIVDSHFKKGQVVERLRVNPGH

3Fe-4S

Subtype 1

>3Fe-4SST1(bju:BJ6T_68600)Bradyrhizobium japonicum USDA 6
MTERLRVHVDPDKCQGHARCKALAPELFELDEYGNAHEAGDGTVPPGLEDKAWLAKSNCP
EIAIDVIEE
>3Fe-4SST1(bjp:RN69_33285)Bradyrhizobium japonicum E109
MTERLRVHVDPDKCQGHARCKALAPELFELDEYGNAHEAGDGTVPPGLEDKAWLAKSNCP
EIAIDVIEE
>3Fe-4SST1(brs:S23_51090)Bradyrhizobium sp. S23321
MTERLRVHVDPDKCQGHARCKALAPELFELDEYGNAHEAGDGIVPPGLEDKAWLAKSNCP
EIAIDVIEE
>3Fe-4SST1(brc:BCCGELA001_13030)Bradyrhizobium sp. CCGE-LA001
MTERLKVHVDPDKCQGHARCKALAPELFELDEYGNAHEAGDGIVPPGLEDKAWLAKSNCP
EIAIDVIEE
>3Fe-4SST1(brad:BF49_1786)Bradyrhizobium sp. BF49
MTERLRVHVDPDKCQGHARCKALAPELFELDEYGNAHEAGDGTVPPGLEDKAWLAKSNCP
EIAIDVIEE

>3Fe-4SST1(bic:LMTR13_32160)Bradyrhizobium icense
MAGKLKIRVDQDKCQGHARCKSLAPELFELDEFGNAHEVGDGSVPAGLEDKAWLAQSNCP
EIAIEVTEE
>3Fe-4SST1(bic:LMTR13_32125)Bradyrhizobium icense
MSENRVLIVKIDPEKCQGHARCHALATELFELDEFGNARVRGSGQIPPSLEDQAWIARAN
CPELAVEIVEQAKAPD
>3Fe-4SST1(rpa:RPA1733)Rhodopseudomonas palustris CGA009
MSDHLNASETLKIHVDQDKCQGHARCKALAPELFELDDYGNAHETGDGTVPTALIDQAWL
AKSNCPENAIVIDEA
>3Fe-4SST1(rpb:RPB_3630Rhodopseudomonas palustris HaA2
MSEMLTIHVDQDKCQGHARCKALAPELFDLDDYGNAHEKGDGVVPADLIDKAWLAKSNCP
ENAIDITED
>3Fe-4SST1(rpd:RPD_1837)Rhodopseudomonas palustris BisB5
MSETLKIHVDQDKCQGHARCKALAPELFDLDEYGNAHEKGDGSVPASLIDKAWLAKSNCP
EVAIDITED
>3Fe-4SST1(rpt:Rpal_1933)Rhodopseudomonas palustris TIE-1
MSDHLNASETLKIHVDQDKCQGHARCKALAPELFELDDYGNAHETGDGTVPTALIDQAWL
AKSNCPENAIVIDEA
>3Fe-4SST1(nha:Nham_2692)Nitrobacter hamburgensis
MSGTLRIHVDPDKCQGHARCKSLAPELFELDALGNAHEIGDGTVPAGLEDKAWLAQANCP
EFAIAIKEE
>3Fe-4SST1(nar:Saro_3681)Novosphingobium aromaticivorans
MRVIANHALCQGHARCEDLCPEVFATDAVEGKVVLRMEEVPEALEAKVRLAVRNCPEGAL
RVVRSEGAEA
>3Fe-4SST1(smaz:LH19_26275)Sphingopyxis macrogoltabida 203
MKIKVDQHNCIGAGNCAQLAPKVFSQREEDGVVILLDETPGDSEAEDVAKAVKICPAQAI

SVSEG

>3Fe-4SST1(smaz:LH19_26370)Sphingopyxis macrogoltabida 203

MKVKIDFKRCAGHARCMEEAPDVFGYNNTTNFTFVHRGADLEANRAAIDLAIMACPEQAI

SWVDDNAMSVEDAAEGLD

>3Fe-4SST1(swi:Swit_1965)Sphingomonas wittichii

MRVKVDEDRCQGHAMCTLACPELFLLNDDDGHAYVEAELVPAGFEENVRQAQRGCPEAAI

IVIED

>3Fe-4SST1(sphb:EP837_03353)Sphingobium sp. EP60837

MRVIANMNVCQGHARCEDLCPEVFSTDAIEGKVVIEQPEFPPELEEKVRMAVRNCPEGAL

RIAKGTGV

>3Fe-4SST1(rga:RGR602_PB00448)Rhizobium gallicum

MRVVIDQDLCGTTWQCVLTLPGTFRQREPDGVAEVCLATVPEALHAAVRLAASQCPVAAI

RVIESDAGYGEPAIVDPAPSPAEVGRHAAKDHHNPGGHDGTV

>3Fe-4SST1(mop:Mesop_6229)Mesorhizobium opportunistum

MRVVVDQDLCGTTGQCVLTLPGTFRQREPDGVAEVCAATVPQALLAAVRLAASQCPVAAI

RVISDAGDDERASADAAPSPAEPERHAAKDQRNPEGHDGTV

>3Fe-4SST1(mam:Mesau_05698)Mesorhizobium australicum

MRVVVDQDLCGTTGQCVLTLPGTFRQREPDGVAEVCAATVPQALLAAVRLAASQCPVAAI

RVISDAGDDERASADAAPSPAEPERHAAKDQRNPEGHDGTV

>3Fe-4SST1(mamo:A6B35_33020) Mesorhizobium amorphae

MRVVVDQDLCGTTGQCVLTLPGTFRQREPDGVAEVCVATVPQALHAAVRLAASQCPVAAI

RVIESNAGDDERASADPAPSPAEAERHAAKDQRNPGGHDGTV

>3Fe-4SST1(rhi:NGR_a02720)Sinorhizobium fredii NGR234

MRVVIDQDLCGTTGQCVLTLPGTFRQREPDGVAEVCVATVPQALHAAVRLAASQCPVAAI

RVIESDAGDDERASADPARSPAEAERHAAKDQRIPGGHDGTV

>3Fe-4SST1(rtr:RTCIAT899_PB00445)Rhizobium tropici

MRVVVDQDLCGTTGQCVLTLPGVFRQRELDGVAEVCVVTVPQALHAAVRLAASQCPVAAI
WVVESAAGDGEPARADPTPSLAEAERHATKDQCNPGGHDGTV
>3Fe-4SST1(bjp:RN69_37410)Bradyrhizobium japonicum E109
MRVMVDQDLCGTSGQCVLTLPGTFRQREPDGVAEVCVATVPHALHAAVRLAASQCPVAAI
RVIESDAGDGERASADPAPSPAEAERHAAKDQHNLG
>3Fe-4SST1(bju:BJ6T_77040)Bradyrhizobium japonicum USDA 6
MRVMVDQDLCGTSGQCVLTLPGTFRQREPDGVAEVCVATVPHALHAAVRLAASQCPVAAI
RVIESDAGDGERASADPAPSPAEAERHAAKDQHNLG
>3Fe-4SST1(bja:bsl2906)Bradyrhizobium diazoefficiens USDA 110
MAERLKVHVDPDKCQGHARCKALAPELFELDEYGNAHEAGDGTVPPGLEEKAWLAKSNCP
EIAIDVIEE

Subtype 2

>3Fe-4SST2(sphr:BSY17_3857)Sphingobium sp. RAC03
MKIRIEKAGCVGNARCAAVSVTLYPLDDDGYIQTEGFTVPEGMEKAARNGARACPERIIV
VEEEDGRISWPPAAKA
>3Fe-4SST2(rtr:RTCIAT899_PB00990)Rhizobium tropici
MHIIVHTAKCQGHARCWFQAPEIFKLDEDGYILPGNIDVAAGNEFRASRGARSCPERALE
ITRSFDTVGVTA
>3Fe-4SST2(sch:Sphch_0987)Sphingobium chlorophenolicum
MKIIVHNDKCQGHARCWAMAPHIFDLDDEGYIKPGDIEVAEADEKLAWKGAKSCPERALE
IEK
>3Fe-4SST2(syb:TZ53_19620)Sphingobium sp. YBL2
MKIIVHNDKCQGHARCWAMAPNIFELDDEGYIKPGDIDVAEADEKLAWKGAKSCPERALE

IGK

>3Fe-4SST2(sphb:EP837_00047)Sphingobium sp. EP60837

MKIIVHNDKCQGHARCWAMAPNIFQLDDEGYIKPGDIEVAETDEKLAWKGAKSCPERALE

IGK

>3Fe-4SST2(same:SAMCFNEI73_pB0104)Sinorhizobium americanum

MRIIVHKNKCQGHARCWAQAPDIFNLDDEGYILPGDIEVAERDELLASRGARSCPERALE

IDATADALLTRLLFNQACGGVK

>3Fe-4SST2(ret:RHE_PD00214)Rhizobium etli CFN 42

MEYRMRVIVHTSKCQGHARCWAQAPEIFNLDDEGYILPGAIDVEERDELFASRGARSCPE

RALEIDCVSDALLDRLLFNQHCGG

>3Fe-4SST2(rec:RHECIAT_PB0000258)Rhizobium etli CIAT 652

MEYRMRVIVHTSKCQGHARCWAQAPEIFNLDDEGYILPGAIDVEERDELFASRGARSCPE

RALEIDCVSDALLDRLLFNQHCGG

>3Fe-4SST2(rel:REMIM1_PE00311)Rhizobium etli bv. mimosae Mim1

MRIIVHSAKCQGHARCWAQAPDIFKLNDEGYILPGEISVEERDELLASRGARSCPERALE

VDCPSDALLDRLLFNQHCRG

>3Fe-4SST2(rei:IE4771_PB00207)Rhizobium sp. IE4771

MRITVHSAKCQGHARCWAQAPDIFNLNDEGYILPGAIDVEERDELLASRGARSCPERALE

IDCASDALLDRLLFNQYCGG

>3Fe-4SST2(rga:RGR602_PB00272)Rhizobium gallicum

MRIIVHNAKCQGHARCWAQAPDIFDLNDEGYILPGDIDVEEKDELLASRGARSCPERALE

IDRTSDALLDRLLFNQHCGG

>3Fe-4SST2(rhn:AMJ98_PC00265)Rhizobium sp. N1341

MEYRMRVIVHTSKCQGHARCWAQAPEIFNLDDEGYILPGAIDVEERDELFASRGARSCPE

RALEIDCVSDALLDRLLFNQHCGG

>3Fe-4SST2(sgi:SGRAN_1060)Sphingopyxis granuli

MKAIVDVGKCSGHARCVAVAPHVFVLNSDGYNERAINPVPAGHEVEGRRGARACPERAIR

IVEDGEESEAGR

>3Fe-4SST2(swi:Swit_1742)Sphingomonas wittichii

MKVIVDHARCQGHALCMLKAPQVYQLNDDGYNSMPPTEVPPELEEQARLGAINCPEGAIR

LDKRAVD

>3Fe-4SST2(rhx:AMK02_PC00260)Rhizobium sp. N731

MEYRMRVIVHTSKCQGHARCWAQAPEIFNLDDEGYILPGAIDVEERDELFASRGARSCPE

RALEIDCVSDALLDRLLFNQHCGG

>3Fe-4SST2(rpha:AMC79_PC00242)Rhizobium phaseoli

MEYRMRVIVHTSKCQGHARCWAQAPEIFNLDDEGYILPGAIDVEERDELFASRGARSCPE

RALEIDCVSDALLDRLLFNQHCGG

Subtype 3

>3Fe-4SST3(mex:Mext_4002)Methylorubrum extorquens PA1

MKVVVDLNRCQAYAQCIYAAPGHFALHGREALVYDPSPDETARGEIERAIHACPVRAITA

YPDAADAAAGEAR

>3Fe-4SST3(mea:Mex_1p4390)Methylorubrum extorquens AM1

MKVVVDLNRCQAYAQCIYAAPGHFALHGREALVYDPSPDETARGEIERAIHACPVRAITA

YPDAADAAAGEAR
>3Fe-4SST3(mdi:METDI4996)Methylorubrum extorquens DM4
MKVVVDLNRCQAYAQCIYAAPGHFALHGREALVYDPSPDETARGEIERAIHACPVRAITA
YPDAADAAAGEAR
>3Fe-4SST3(mch:Mchl_4371)Methylorubrum extorquens CM4
MKVVVDLNRCQAYAQCIYAAPGHFALHGREALVYDPSPDETARGEIERAIHACPVRAITA
YPDAADAAAGEAR
>3Fe-4SST3(mpo:Mpop_4483)Methylorubrum populi
MKVVVDLNRCQAYAQCLYAAPGHFVLHGKEALVYDPSPDEAARGEIERAVHACPVRAITA
YSDDTTAGEAA
>3Fe-4SST3(mrd:Mrad2831_3878)Methylobacterium radiotolerans
MKPASDTAALRVVVDLNRCQGYAQCCYAAPDAFALRGHEILFYDPAPPAERRGAIERALQ
ACPVRAISLQAGPDDGGAP
>3Fe-4SST3(mrd:Mrad2831_1825)Methylobacterium radiotolerans
MSAPADASSGHPPGLRVSVDLNLCQAYAQCCYAAPRHFRIEGHEALFYDPAPAARDRDDI
ERARVACPVQAIRVEDPERGA
>3Fe-4SST3(met:M446_4601)Methylobacterium sp. 4-46
MRVVVDLNRCQAYAQCCFAAPESFALHGPEVLVYDAAPPPERRREIERAVQACPVRAILL
EVEPEEAGSHG
>3Fe-4SST3(mno:Mnod_0158)Methylobacterium nodulans
MRVVVDLNRCQAYAQCCFAAPDSFALHGIEVLVYDPAPPPERRREIERAVQACPVRAIML
EVEPEAERHD
>3Fe-4SST3(mor:MOC_4428)Methylobacterium oryzae
MKPAAGAGALRVVVDLNRCQGYAQCCYAAPEAFAIRGHEILFYDPAPPADRRGAVERAVE
ACPVRAISLQAGPDDGDAP
>3Fe-4SST3(mor:MOC_2233)Methylobacterium oryzae

MSVDLNLCQAYAQCCYAAPRHFRIEGHEALFYDPAPAAQDRDDIERARVACPVQAIRVED
PERGA
>3Fe-4SST3(meta:Y590_19925)Methylobacterium sp. AMS5
MKVVVDLNRCQAYAQCIYAAPGHFALHGREALVYDPSPDETARGEIERAIHACPVRAITA
YPDAADAAAGEAR
>3Fe-4SST3(maqu:Maq22A_1p34665)Methylobacterium aquaticum
MKVVVDLNRCQAYAQCIYAAPDHFALHGAEALVYDTAPDEAARAEIERAVQACPVRAITA
TSDVPAPMPHKVGER
>3Fe-4SST3(maqu:Maq22A_c00725)Methylobacterium aquaticum
MRVVVDLNRCQAYAQCCYAAPERFVLQGDEILAYDPAPPAAERAAIDRAVQACPVRAIRV
EHDETAERAA

Subtype 4

>3Fe-4SST4(sphm:G432_19810)Sphingomonas sp. MM-1
MMQVKTLPERCVSSGQCVMLAPGVFDQDDDGVVLLLRDTVDDGDEASVAAVRSSANVCPAAAIRLSATPKA
>3Fe-4SST4(sjp:SJA_C2-04950) Sphingobium japonicum
MMQVKTLPERCVSSGQCVMLAPGVFDQDDDGVVLLLRDTVDDGDEASVAAVRSSANVCPAAAIRLSATPKA
>3Fe-4SST4(sch:Sphch_3796)Sphingobium chlorophenolicum
MQVKTLPERCVSSGQCVMLAPGVFDQDDDGVVVLLQDSVDDGDEASVAAVRSSANVCPAAAIRLSANPKA

Subtype 5

>3Fe-4SST5(cak:Caul_1918)Caulobacter sp. K31
MSSEATPHRATADRSACCGYGTCVAICPEIYQLEGGLVVLTTDIVPAELLERAIEGADSC
PQSAIVIEAVAG

4Fe-4S

Subtype 1

>4Fe-4SST1(blas:BSY18_2505)Blastomonas sp. RAC04

MSGEVTLKVVINKPACCGYGVCADICPEVFKLDENGIVFVESDLVPPELEETAREAADAC

PQSALALVAA

>4Fe-4SST1(syb:TZ53_18285)Sphingobium sp. YBL2

MSQKLKVVIDKAACCGYGVCAEICPQVYKLDANGIVYVDDEIVPEGLEEQAREGAEACPQ

SALAVGAA

7Fe-8S

Subtype 1

>7Fe-8SST1(ngl:RG1141_CH23220)Neorhizobium galegae bv. officinalis bv. officinalis HAMBI 1141

MAYIITEPCIDVKDGACTAACPVDCIYEGGRMFYIHPDECINCGLCLSICPVDAIAYDEE

ATEAQMAFIPVNRDYFGAEVTGLGSPGGWDKSRTRNIDHPVVTSTPAREVAH

>7Fe-8SST1(rle:pRL110029)Rhizobium leguminosarum bv. viciae 3841

MAYVITDPCIDVKDGDCTVACPVDCIYEGGRMFYIHPGECINCGLCLSVCPVDAISWDEE

IPQSRVQFKAVNQDFFGPDVTNWGSPGGWDKNHTTNRDHPLVAAYERA

>7Fe-8SST1(ngg:RG540_CH24080)Neorhizobium galegae bv. orientalis HAMBI 540

MAYIITEPCIDVKDGACTAACPVDCIYEGGRMFYIHPDECINCGLCLSICPVDAIAYDEE

ANEAQMAFIPVNRDYFGADVTGLGSPGGWDKSRTRNIDHPVVTSAPVREAAH

>7Fe-8SST1(eah:FA04_32640)Ensifer adhaerens Casida A
MAYVITEPCIDVKDGDCTAACPVDCIYEGGRTFYIHPDECINCGLCLSICPVDAIAWDQE
IPNNQRHFIAVNKEFFGSDVTGMGSPGGWSKECSTTQDHPFVAAFEKAAAIA
>7Fe-8SST1(lagg:B0E33_29700)Labrenzia aggregata
MALIIKSECIDVKDGICTTSCPVDCIYEGERMFYIHPEECIECGMCESICPVDAIRYYDE
VTGADRAFIEINKDVFQGPDGGLVSPGGWTRGDTPFRDHPAVRDFFKRDFEEQTT
>7Fe-8SST1(jan:Jann_3749)Jannaschia sp. CCS1
MALVILSACVDVKDGICTTSCPVDCIYEGERMFYIHPTECIECGMCESICPVDAIRYDDE
VPAEEQPFADLNTSVFQSADGGVQEPGGWAKGMDPLRDPTVLADMAAKTELKREEN

Subtype 2

>7Fe-8SST2(rhn:AMJ98_CH02815)Rhizobium sp. N1341
MEKQPNISRRAPDIVRPTVDPARCEGKAACVAVCPVDVFEIVRIPDETYRDLPLLAKFKI
WAHGMKTAATPNAAACEGCGLCVSACPEHAIRLQRAG
>7Fe-8SST2(rhx:AMK02_CH02746)Rhizobium sp. N731
MEKQPNISRRAPDIVRPMVDPARCEGKAACVAVCPVDVFEIVRIPDETYRDLPLLAKFKI
WAHGMKTAATPNAAACEGCGLCVSACPEHAIRLQRAG
>7Fe-8SST2(bbt:BBta_3785)Bradyrhizobium sp. BTAi1
MTDHTEPTPSKRTNAAKVRKAAQHPKRPGAGCKAEPGAFVPVVDHNRCEAKGDCVEVCPY
DVFDVAPIRDADFRTLSFLGRMRVRFHGMKTAYTPNSQLCLACGLCVVACPERAIDLVAA
AAREDHPLSR
>7Fe-8SST2(aol:S58_42030)Bradyrhizobium oligotrophicum
MTDLTKPTLSKRTNPDKVRKAAQHPKRPGLGCKAEPGTFVPVVDRNKCEAKGDCVEVCPY
DVFDVAPISEPDFRAMSFLGRMRVRFHGMKTAYTPNSDRCLACGLCVVACPERAINLVEV
AAGSNMTTRR

Subtype 3

>7Fe-8SST3(otm:OSB_13110)Octadecabacter temperatus
MSKKLPPKPDTQKPAASVVNIDYDLCINCAICIRSCPVDVLRINRETRWLEATYWEDCML
CKLCELDCPEPGAITISADKPLGFAMSFG

Subtype 4

>7Fe-8SST4(bic:LMTR13_00130)Bradyrhizobium icense
MEPQGPSRRALFRGQLLSRPVALIGDACLAEAGIVCRSCGDACPASAIRFRPRIGLPPQA
IVNEAVCTGCGECVDACPGATITLGAAHGGDAA

2[4Fe-4S]
Subtype 1

>2[4Fe-4S]ST1(rhl:LPU83_4061)Rhizobium favelukesii
MTYVVTDNCIRCKYTDCVEVCPVDCFYEGENFLVIHPDECIDCGVCEPECPAEAIKPDTE
PGLDKWLRINAEYASVWPNITVKKDAMPEAKELDGESGKFEKYFSEKPGSGD
>2[4Fe-4S]ST1(mlo:mlr3855)Mesorhizobium japonicum MAFF 303099
MTYVVTDNCIKCKYMDCIEVCPVDCFYEGENMLVIHPDECIDCGVCEPECPADAIKPDTE

PGLDKWLQINTEYAEKWPNITAKKEPPADAKTFDGEAGKFEKYFSAEPGEGD
>2[4Fe-4S]ST1(mln:A9174_06575)Mesorhizobium loti NZP2037
MTYVVTDNCIKCKYMDCIEVCPVDCFYEGENMLVIHPDECIDCGVCEPECPADAIKPDTE
PGLDKWLQINTEYAEKWPNITAKKEPPADAKTFDGEAGKFEKYFSAEPGEGD
>2[4Fe-4S]ST1(mci:Mesci_1325)Mesorhizobium ciceri (biovar Biserrulae)
MTYVVTDNCIKCKYMDCIEVCPVDCFYEGENMLVIHPDECIDCGVCEPECPADAIKPDTE
PGLDKWLQVNTEYAEKWPNITAKKEPPADAKSFDGEAGKFEKYFSAEPGEGD
>2[4Fe-4S]ST1(mop:Mesop_1348)Mesorhizobium opportunistum
MTYVVTDNCIKCKYMDCIEVCPVDCFYEGENMLVIHPDECIDCGVCEPECPADAIKPDTE
SGLDKWLQINTEYAEKWPNITAKKEPPADAKTFDGEAGKFEKYFSAEPGEGD
>2[4Fe-4S]ST1(mam:Mesau_01363)Mesorhizobium australicum
MTYVVTDNCIKCKYMDCIEVCPVDCFYEGENMLVIHPDECIDCGVCEPECPADAIKPDTE
SGLDKWLQINTEYADKWPNITAKKEPPADAKSFDGEAGKFEKYFSAEPGEGD
>2[4Fe-4S]ST1(mamo:A6B35_01755)Mesorhizobium amorphae
MTYVVTDNCIKCKYMDCIEVCPVDCFYEGENMLVIHPDECIDCGVCEPECPADAIKPDTE
PGLEKWLQVNTEYAEKWPNITAKKEPPADAKTFDGEAGKFEKYFSAEPGEGD
>2[4Fe-4S]ST1(meso:BSQ44_00295)Mesorhizobium sp. B7
MTYVVTDNCIKCKYMDCIEVCPVDCFYEGENMLVIHPDECIDCGVCEPECPADAIKPDTE
PGLEKWLQVNTEYADKWPNITAKKEPPADAKEFDGVEGKFEKYFSAEAGEGD
>2[4Fe-4S]ST1(mes:Meso_3378)Chelativorans sp. BNC1
MAHLAIALQRGYLTRRKMRPERREPVMTYVVTDNCIKCKYMDCIEVCPVDCFYEGENMLV
IHPDECIDCGVCEPECPAEAIKPDTEPGLEKWLQVNADYAEKWPNITAKKEPPADAKDWD
GIEEKFEKYFSPEPGTGD
>2[4Fe-4S]ST1(hoe:IMCC20628_04489)Hoeflea sp. IMCC20628
MTYVVTDNCIRCKYMDCVEVCPVDCFYEGDNMLVIHPDECIDCGVCEPECPAEAIKPDTE
PGLEKWLEVNTEYADKWPNITIKREAPEDAKKFDGEAGKFEKYFSEAPGEGD

>2[4Fe-4S]ST1(aak:AA2016_1364)Aminobacter aminovorans
MTYVVTDNCIKCKYMDCIEVCPVDCFYEGENMLVIHPDECIDCGVCEPECPADAIKPDTE
SGLDKWLQINTEYAEKWPNITAKKEPPADAKEFDGVEGKFEKFFSAEPGEGN
>2[4Fe-4S]ST1(aak:AA2016_5900)Aminobacter aminovorans
MTYVVTDACVRCKFTDCVAVCPVDCFHEGENMLVIDPEVCIDCGVCEPECPADAILADTD
PLAAKWLAVNRQYSAHWPVISAQLPPPQDAHAFKGLDGKFEAHFSPAAAAR
>2[4Fe-4S]ST1(pla:Plav_1695)Parvibaculum lavamentivorans
MTYIVTDACIRCKYMDCVEVCPVDCFYEGENMLVIHPDECIDCGVCEPECPAEAIKPDTE
PGLEKWLELNTEYASKWPNITIKRDPPADADDWQGVSGKFEEHFSAEPGEGD
>2[4Fe-4S]ST1(sme:SMc03875)Sinorhizobium meliloti 1021
MTYVVTDNCIRCKYTDCVEVCPVDCFYEGENFLVIHPDECIDCGVCEPECPAGAIKPDTE
PGLDMWLKLNAEFSTQWPNITVKRDPLPEAKEMDGVEEKYEKYFSSEPGQGD
>2[4Fe-4S]ST1(smk:Sinme_3255)Sinorhizobium meliloti AK83
MTYVVTDNCIRCKYTDCVEVCPVDCFYEGENFLVIHPDECIDCGVCEPECPAGAIKPDTE
PGLDMWLKLNAEFSTQWPNITVKRDPLPEAKEMDGVEEKYEKYFSSEPGQGD
>2[4Fe-4S]ST1(smq:SinmeB_3030)Sinorhizobium meliloti BL225C
MTYVVTDNCIRCKYTDCVEVCPVDCFYEGENFLVIHPDECIDCGVCEPECPAGAIKPDTE
PGLDMWLKLNAEFSTQWPNITVKRDPLPEAKEMDGVEEKYEKYFSSEPGQGD
>2[4Fe-4S]ST1(smx:SM11_chr3386)Sinorhizobium meliloti SM11
MTYVVTDNCIRCKYTDCVEVCPVDCFYEGENFLVIHPDECIDCGVCEPECPAGAIKPDTE
PGLDMWLKLNAEFSTQWPNITVKRDPLPEAKEMDGVEEKYEKYFSSEPGQGD
>2[4Fe-4S]ST1(smi:BN406_03059)Sinorhizobium meliloti Rm41
MTYVVTDNCIRCKYTDCVEVCPVDCFYEGENFLVIHPDECIDCGVCEPECPAGAIKPDTE
PGLDMWLKLNAEFSTQWPNITVKRDPLPEAKEMDGVEEKYEKYFSSEPGQGD
>2[4Fe-4S]ST1(smeg:C770_GR4Chr3353)Sinorhizobium meliloti GR4
MTYVVTDNCIRCKYTDCVEVCPVDCFYEGENFLVIHPDECIDCGVCEPECPAGAIKPDTE

PGLDMWLKLNAEFSTQWPNITVKRDPLPEAKEMDGVEEKYEKYFSSEPGQGD
>2[4Fe-4S]ST1(smel:SM2011_c03875)Sinorhizobium meliloti 2011
MTYVVTDNCIRCKYTDCVEVCPVDCFYEGENFLVIHPDECIDCGVCEPECPAGAIKPDTE
PGLDMWLKLNAEFSTQWPNITVKRDPLPEAKEMDGVEEKYEKYFSSEPGQGD
>2[4Fe-4S]ST1(smer:DU99_17585)Sinorhizobium meliloti RMO17
MTYVVTDNCIRCKYTDCVEVCPVDCFYEGENFLVIHPDECIDCGVCEPECPAGAIKPDTE
PGLDMWLKLNAEFSTQWPNITVKRDPLPEAKEMDGVEEKYEKYFSSEPGQGD
>2[4Fe-4S]ST1(smd:Smed_3113)Sinorhizobium medicae
MTYVVTDNCIRCKYTDCVEVCPVDCFYEGENFLVIHPDECIDCGVCEPECPAGAIKPDTE
PGLDMWLKLNADFSTQWPNITVKRDPLPEATEMDGLEGKYEKYFSPEPGQGD
>2[4Fe-4S]ST1(rhi:NGR_c32680)Sinorhizobium fredii NGR234
MTYVVTDNCIRCKYTDCVEVCPVDCFYEGENFLVIHPDECIDCGVCEPECPAGAIKPDTE
PGLDMWLKLNADFATQWPNITVKRDPLPEAKEMDGVEGKYEQYFSEKPGQGD
>2[4Fe-4S]ST1(sfh:SFHH103_03286)Sinorhizobium fredii HH103
MTYVVTDNCIRCKYTDCVEVCPVDCFYEGENFLVIHPDECIDCGVCEPECPAGAIKPDTE
PGLDMWLKLNADFATQWPNITVKRDPLPEAKEMDGVEGKYEQYFSEKPGQGD
>2[4Fe-4S]ST1(sfd:USDA257_c57280)Sinorhizobium fredii USDA 257
MTYVVTDNCIRCKYTDCVEVCPVDCFYEGENFLVIHPDECIDCGVCEPECPAGAIKPDTE
PGLDMWLKLNADFATQWPNITVKRDPLPEAKEMDGVEGKYEQYFSEKPGQGD
>2[4Fe-4S]ST1(six:BSY16_3356)Sinorhizobium sp. RAC02
MTYVVTDNCIRCKYMDCVEVCPVDCFYEGENFLVIHPDECIDCGVCEPECPAEAIKPDTE
PGLDKWLKINTEFASIWPNVTVKRDAMPEAKEMDGVEGKYEQFFSPNPGQGD
>2[4Fe-4S]ST1(ead:OV14_0780)Ensifer adhaerens OV14
MTYVVTDNCIRCKYMDCVEVCPVDCFYEGENFLVIHPDECIDCGVCEPECPAEAIKPDTE
PGLDMWLKVNADFATKWPNVTIKRDAMPEAKEMDGVEGKYEKYFSAEPGQGD
>2[4Fe-4S]ST1(eah:FA04_17280)Ensifer adhaerens Casida A

MTYVVTDNCIRCKYTDCVEVCPVDCFYEGENFLVIHPDECIDCGVCEPECPAEAIKPDTE
PGLDMWLKLNADFASKWPNITVKRDPMPEAKEMDGVEGKYEKFFSTEPGQGD
>2[4Fe-4S]ST1(atu:Atu2751)Agrobacterium fabrum
MTYVVTDNCIRCKYTDCVEVCPVDCFYEGENFLAINPDECIDCGVCEPECPAEAIKPDTE
PGLDKWLKINTEYAAIWPNITIKRDPMPEAKEMDGVEGKLELYFSAEPGKGD
>2[4Fe-4S]ST1(atu:Atu4582)Agrobacterium fabrum
MPYVVTENCIACKYMDCVEVCPVECFYEGENMLVIHPDQCIDCGICERECPAAAIRPDTE
AGLHVWLDLNRHYSGIWPRVHQKRTPPDNADTMNGAAAKFSILSKNPGAGG
>2[4Fe-4S]ST1(ara:Arad_4672)Agrobacterium radiobacter
MECFMTYVVTDNCVRCKYTDCVEVCPVDCFYEGENFLVIHPDECIDCGVCEPECPAEAIK
PDTEPGLDKWLKINADYAAIWPNITVKRDALPEAKELDGEEGKFEKYFSANPGAGD
>2[4Fe-4S]ST1(atf:Ach5_26350)Agrobacterium tumefaciens Ach5
MTYVVTDNCIRCKYTDCVEVCPVDCFYEGENFLAINPDECIDCGVCEPECPAEAIKPDTE
PGLDKWLKLNTEYAAIWPNITIKRDPLPEAKEMDGVTGKLELYFSAEPGKGD
>2[4Fe-4S]ST1(atf:Ach5_42740)Agrobacterium tumefaciens Ach5
MPYVVTENCIACKYMDCVEVCPVECFYEGENMLVIHPDQCIDCGICERECPAAAIRPDTE
AGLHVWLDLNRHYSGIWPRVHQKRTPPEDADIMNGAAAKFSIFSKNPGAGG
>2[4Fe-4S]ST1(ata:AWN88_18615)Agrobacterium tumefaciens S33
MTYVVTDNCIRCKYTDCVEVCPVDCFYEGENFLAINPDECIDCGVCEPECPAEAIKPDTE
PGLDKWLKLNAEYAAIWPNITIKRDPMPEAKEMDGVEGKLELYFSAEPGKGD
>2[4Fe-4S]ST1(ata:AWN88_02865)Agrobacterium tumefaciens S33
MPYVVTENCIACKYMDCVDVCPVECFYEGENMLVIHPDQCIDCGICESECPAAAIRPDTE
PGLHVWLDLNRHYSGIWPRVHQKRAPPDNADIMNGAAAKFSIFSKNPGAGG
>2[4Fe-4S]ST1(avi:Avi_4350)Agrobacterium vitis
MEYRMTYIVTDNCIRCKYTDCVEVCPVDCFYEGENFLAINPDECIDCGVCEPECPAEAIK
PDTEPGLDKWLKINAEFAQVWPNITTKRDALPEAKEMDGVEGKFELYFSEKPGTGD

>2[4Fe-4S]ST1(agr:AGROH133_14003)Agrobacterium sp. H13-3
MSYVVTENCIACKYMDCVEVCPVECFYAGENMLVIHPDQCIDCGICERECPAAAIRPDTE
AGLHVWLDLNRHYSGIWPRVHQKRTPPDDADIMNGAAAKFSIFSKNPGAGD
>2[4Fe-4S]ST1(agr:AGROH133_09162)Agrobacterium sp. H13-3
MTYVVTDNCIRCKYTDCVEVCPVDCFYEGENFLAINPDECIDCGVCEPECPAEAIKPDTE
PGLDKWLKLNTEYAAIWPNITIKRDPLPEAKEMDGVTGKLELYFSAEPGKGD
>2[4Fe-4S]ST1(agc:BSY240_3071)Agrobacterium sp. RAC06
MTYIVTDNCIRCKYTDCVEVCPVDCFYEGENFLVIHPDECIDCGVCEPECPAEAIKPDTE
PGLDKWLKINAEYAKIWPNITVKKEPMPEAKEMDGVDGKFEQFFSDKPGSGD
>2[4Fe-4S]ST1(aro:B0909_25795)Agrobacterium rhizogenes
MTYVVTDNCIRCKYTDCVEVCPVDCFYEGENFLAINPDECIDCGVCEPECPAEAIKPDTE
PGLDKWLKLNAEYAAIWPNITIKRDPMPEAKEMDGVAGKLELYFSAEPGQGD
>2[4Fe-4S]ST1(aro:B0909_14630)Agrobacterium rhizogenes
MPYVVTENCIACKYMDCVEVCPVECFYEGENMLVIHPDQCIDCGICERECPAAAIRPDTE
AGLHVWLDLNRHYSGIWPRVHQKRTPPDNADIMNGAAAKFSIFSKNPGAGG
>2[4Fe-4S]ST1(ret:RHE_CH04024)Rhizobium etli CFN 42
MTYVVTDNCIKCKYTDCVEVCPVDCFYEGENFLVIHPDECIDCGVCEPECPAEAIKPDTE
PGLDKWLKINTEYASIWPNITVKKDPLPEAKEMDGQTGKFEKYFSEKPGSGD
>2[4Fe-4S]ST1(rec:RHECIAT_CH0004312)Rhizobium etli CIAT 652
MTYVVTDNCIKCKYTDCVEVCPVDCFYEGENFLVIHPDECIDCGVCEPECPAEAIKPDTE
PGLDKWLKINTEYASIWPNITVKKDPLPEAKEMDGQTGKFEKYFSEKPGSGD
>2[4Fe-4S]ST1(rel:REMIM1_CH04122)Rhizobium etli bv. mimosae Mim1
MTYVVTDNCIKCKYTDCVEVCPVDCFYEGENFLVIHPDECIDCGVCEPECPAEAIKPDTE
PGLDKWLKINTEYASIWPNITVKKDPLPEAKEMDGQTGKFEKYFSEKPGSGD
>2[4Fe-4S]ST1(rep:IE4803_CH04410)Rhizobium etli bv. phaseoli IE4803
MTYVVTDNCIKCKYTDCVEVCPVDCFYEGENFLVIHPDECIDCGVCEPECPAEAIKPDTE

PGLDKWLKINTEYATIWPNITVKKDPLPEAKEMDGQTGKFEKYFSEKPGSGD

>2[4Fe-4S]ST1(rei:IE4771_CH04360)Rhizobium sp. IE4771

MTYVVTDNCIKCKYTDCVEVCPVDCFYEGENFLVIHPDECIDCGVCEPECPAEAIKPDTE

PGLDKWLKINTEYATIWPNITVKKDPLPEAKEMDGQTGKFEKYFSEKPGSGD

>2[4Fe-4S]ST1(rle:RL4616)Rhizobium leguminosarum bv. viciae 3841

MTYVVTDNCIKCKYTDCVEVCPVDCFYEGENFLVIHPDECIDCGVCEPECPAEAIKPDTE

PGLDKWLKINTEYATIWPNITVKKDPLPEAKDMDGETGKFEKYFSEKPGSGD

>2[4Fe-4S]ST1(rlt:Rleg2_3815)Rhizobium leguminosarum bv. trifolii WSM2304

MTYVVTDNCIKCKYTDCVEVCPVDCFYEGENFLVIHPDECIDCGVCEPECPAEAIKPDTE

PGLDKWLKINTEYATIWPNITVKKDPLPEAKEMDGETGKFEKYFSEKPGSGD

>2[4Fe-4S]ST1(rlg:Rleg_4144)Rhizobium leguminosarum bv. trifolii WSM1325

MTYVVTDNCIKCKYTDCVEVCPVDCFYEGENFLVIHPDECIDCGVCEPECPAEAIKPDTE

PGLDKWLKINTEYATIWPNITVKKEPLPEAKDMDGETGKFEKYFSEKPGSGD

>2[4Fe-4S]ST1(rlb:RLEG3_31680)Rhizobium leguminosarum bv. trifolii WSM1689

MTYVVTDNCIKCKYTDCVEVCPVDCFYEGENFLVIHPDECIDCGVCEPECPAEAIKPDTE

PGLDKWLKINTEYATIWPNITVKKDPLPEAKDMDGETGKFEKYFSEKPGSGD

>2[4Fe-4S]ST1(rlu:RLEG12_30675)Rhizobium leguminosarum bv. trifolii CB782

MTYVVTDNCIKCKYTDCVEVCPVDCFYEGENFLVIHPDECIDCGVCEPECPAEAIKPDTE

PGLDKWLKINTEYATIWPNITVKKDPLPEAKEMDGETGKFEKYFSEKPGSGD

>2[4Fe-4S]ST1(rtr:RTCIAT899_CH17975) Rhizobium tropici

MTYVVTDNCIRCKYTDCVEVCPVDCFYEGENFLVIHPDECIDCGVCEPECPAEAIKPDTE

PGLDKWLKVNAEYAAIWPNITVKRDPMPEAKEMDGEQGKFEKYFSANPGTGD

>2[4Fe-4S]ST1(rir:BN877_I2835) Rhizobium sp. IRBG74

MCSEAQRPGLHILEFIMTYVVTDNCIRCKYTDCVEVCPVDCFYEGENFLAINPDECIDCG

VCEPECPAEAIKPDTEPGLDKWLKLNAEYAAIWPNITIKRDPMPEAKEMDGVEGKLELYF

SAEPGKGD

>2[4Fe-4S]ST1(rir:BN877_II1665) Rhizobium sp. IRBG74

MPYVVTENCIACKYMDCVDVCPVECFYEGENMLVIHPDQCIDCGICERECPAAAIRPDTE

PGLHVWLDLNRHYSGIWPRVHQKRAPPDNADIMNGAAAKFSIFSKNPGAGG

>2[4Fe-4S]ST1(rga:RGR602_CH03938) Rhizobium gallicum

MTYVVTDNCIRCKYTDCVEVCPVDCFYEGENFLVIHPDECIDCGVCEPECPAEAIKPDTE

PGLDKWLKINAEYASSWPNITVKKEPMAEAKEMDGEAGKFEKYFSEKPGSGD

>2[4Fe-4S]ST1(rhn:AMJ98_CH04252) Rhizobium sp. N1341

MTYVVTDNCIKCKYTDCVEVCPVDCFYEGENFLVIHPDECIDCGVCEPECPAEAIKPDTE

PGLDKWLKINTEYASIWPNITVKKDPLPEAKEMDGQTGKFEKYFSEKPGSGD

>2[4Fe-4S]ST1(rpha:AMC79_CH04245) Rhizobium phaseoli

MTYVVTDNCIKCKYTDCVEVCPVDCFYEGENFLVIHPDECIDCGVCEPECPAEAIKPDTE

PGLDKWLKINTEYASIWPNITVKKDPLPEAKEMDGQTGKFEKYFSEKPGSGD

>2[4Fe-4S]ST1(rht:NT26_3598) Rhizobium sp. NT-26

MTYVVTDNCVRCKYTDCVEVCPVDCFYEGENFLVIHPDECIDCGVCEPECPAEAIKPDTE

PGLDKWLKVNAEYAQIWPNITVKREPMPEAKEMDGVPGKFDQYFSPEPGKGD

>2[4Fe-4S]ST1(rhx:AMK02_CH04150)Rhizobium sp. N731

MTYVVTDNCIKCKYTDCVEVCPVDCFYEGENFLVIHPDECIDCGVCEPECPAEAIKPDTE

PGLDKWLKINTEYASIWPNITVKKDPLPEAKEMDGQTGKFEKYFSEKPGSGD

>2[4Fe-4S]ST1(ngl:RG1141_CH37300)Neorhizobium galegae bv. officinalis bv. officinalis HAMBI 1141

MTYVVTDNCIRCKYTDCVEVCPVDCFYEGENFLVIHPDECIDCGVCEPECPAEAIKPDTE

PGLDKWLKINTEFAQMWPNITVKRDALPEAKEMDGVANKYEQYFSAEPGKGD

>2[4Fe-4S]ST1(ngg:RG540_CH37920)Neorhizobium galegae bv. orientalis HAMBI 540

MTYVVTDNCIRCKYTDCVEVCPVDCFYEGENFLVIHPDECIDCGVCEPECPAEAIKPDTE

PGLDKWLKINTEFAQMWPNITVKRDALPEAKEMDGVPNKYEQYFSAEPGKGD

>2[4Fe-4S]ST1(shz:shn_19290) Shinella sp. HZN7

MTYVVTDNCIRCKYTDCVEVCPVDCFYEGENFLVIHPDECIDCGVCEPECPAEAIKPDTE

PGLDKWLQINTEFAAIWPNITVKRDAMPEAKEMDGVEGKYEKYFSPNPGQGD
>2[4Fe-4S]ST1(bme:BMEI0278) Brucella melitensis bv. 1 16M
MTYVVTDNCIRCKYTDCVEVCPVDCFYEGENMLVINPDECIDCGVCEPECPAEAISPDTE
PGLDKWLELNAEYAAKWPNITAKKDALPEAKEMDGVAGKLEQYFSPEAGSGD
>2[4Fe-4S]ST1(bmel:DK63_1155)Brucella melitensis bv. 1 16M
MTYVVTDNCIRCKYTDCVEVCPVDCFYEGENMLVINPDECIDCGVCEPECPAEAISPDTE
PGLDKWLELNAEYAAKWPNITAKKDALPEAKEMDGVAGKLEQYFSPEAGSGD
>2[4Fe-4S]ST1(bmi:BMEA_A1819)Brucella melitensis ATCC 23457
MTYVVTDNCIRCKYTDCVEVCPVDCFYEGENMLVINPDECIDCGVCEPECPAEAISPDTE
PGLDKWLELNAEYAAKWPNITAKKDALPEAKEMDGVAGKLEQYFSPEAGSGD
>2[4Fe-4S]ST1(bmz:BM28_A1763)Brucella melitensis M28
MTYVVTDNCIRCKYTDCVEVCPVDCFYEGENMLVINPDECIDCGVCEPECPAEAISPDTE
PGLDKWLELNAEYAAKWPNITAKKDALPEAKEMDGVAGKLEQYFSPEAGSGD
>2[4Fe-4S]ST1(bmg:BM590_A1762) Brucella melitensis M5-90
MTYVVTDNCIRCKYTDCVEVCPVDCFYEGENMLVINPDECIDCGVCEPECPAEAISPDTE
PGLDKWLELNAEYAAKWPNITAKKDALPEAKEMDGVAGKLEQYFSPEAGSGD
>2[4Fe-4S]ST1(bmw:BMNI_I1693) Brucella melitensis NI
MTYVVTDNCIRCKYTDCVEVCPVDCFYEGENMLVINPDECIDCGVCEPECPAEAISPDTE
PGLDKWLELNAEYAAKWPNITAKKDALPEAKEMDGVAGKLEQYFSPEAGSGD
>2[4Fe-4S]ST1(bmee:DK62_1791) Brucella melitensis bv. 3 Ether
MTYVVTDNCIRCKYTDCVEVCPVDCFYEGENMLVINPDECIDCGVCEPECPAEAISPDTE
PGLDKWLELNAEYAAKWPNITAKKDALPEAKEMDGVAGKLEQYFSPEAGSGD
>2[4Fe-4S]ST1(bmf:BAB1_1778) Brucella abortus 2308
MTYVVTDNCIRCKYTDCVEVCPVDCFYEGENMLVINPDECIDCGVCEPECPAEAISPDTE
PGLDKWLELNAEYAAKWPNITAKKDALPEAKEMDGVAGKLEQYFSPEAGSGD
>2[4Fe-4S]ST1(bmc:BAbS19_I16610) Brucella abortus S19

MTYVVTDNCIRCKYTDCVEVCPVDCFYEGENMLVINPDECIDCGVCEPECPAEAISPDTE
PGLDKWLELNAEYAAKWPNITAKKDALPEAKEMDGVAGKLEQYFSPEAGSGD
>2[4Fe-4S]ST1(baa:BAA13334_I01157) Brucella abortus A13334
MTYVVTDNCIRCKYTDCVEVCPVDCFYEGENMLVINPDECIDCGVCEPECPAEAISPDTE
PGLDKWLELNAEYAAKWPNITAKKDALPEAKEMDGVAGKLEQYFSPEAGSGD
>2[4Fe-4S]ST1(babo:DK55_1723)Brucella abortus bv. 2 86/8/59
MTYVVTDNCIRCKYTDCVEVCPVDCFYEGENMLVINPDECIDCGVCEPECPAEAISPDTE
PGLDKWLELNAEYAAKWPNITAKKDALPEAKEMDGVAGKLEQYFSPEAGSGD
>2[4Fe-4S]ST1(babr:DO74_165)Brucella abortus bv. 6 870
MTYVVTDNCIRCKYTDCVEVCPVDCFYEGENMLVINPDECIDCGVCEPECPAEAISPDTE
PGLDKWLELNAEYAAKWPNITAKKDALPEAKEMDGVAGKLEQYFSPEAGSGD
>2[4Fe-4S]ST1(babt:DK49_1480)Brucella abortus 63 75
MTYVVTDNCIRCKYTDCVEVCPVDCFYEGENMLVINPDECIDCGVCEPECPAEAISPDTE
PGLDKWLELNAEYAAKWPNITAKKDALPEAKEMDGVAGKLEQYFSPEAGSGD
>2[4Fe-4S]ST1(babb:DK48_396) Brucella abortus BDW
MTYVVTDNCIRCKYTDCVEVCPVDCFYEGENMLVINPDECIDCGVCEPECPAEAISPDTE
PGLDKWLELNAEYAAKWPNITAKKDALPEAKEMDGVAGKLEQYFSPEAGSGD
>2[4Fe-4S]ST1(babu:DK53_1706) Brucella abortus bv. 9 C68
MTYVVTDNCIRCKYTDCVEVCPVDCFYEGENMLVINPDECIDCGVCEPECPAEAISPDTE
PGLDKWLELNAEYAAKWPNITAKKDALPEAKEMDGVAGKLEQYFSPEAGSGD
>2[4Fe-4S]ST1(babs:DK51_1836) Brucella abortus BER
MTYVVTDNCIRCKYTDCVEVCPVDCFYEGENMLVINPDECIDCGVCEPECPAEAISPDTE
PGLDKWLELNAEYAAKWPNITAKKDALPEAKEMDGVAGKLEQYFSPEAGSGD
>2[4Fe-4S]ST1(babc:DO78_1627) Brucella abortus NCTC 10505
MTYVVTDNCIRCKYTDCVEVCPVDCFYEGENMLVINPDECIDCGVCEPECPAEAISPDTE
PGLDKWLELNAEYAAKWPNITAKKDALPEAKEMDGVAGKLEQYFSPEAGSGD

>2[4Fe-4S]ST1(bsui:BSSP1_II1207) Brucella suis bv. 2 Bs143CITA
MTYVVTDNCIRCKYTDCVEVCPVDCFYEGENMLVINPDECIDCGVCEPECPAEAISPDTE
PGLDKWLELNAEYAAKWPNITAKKDALPEAKEMDGVAGKLEQYFSPEAGSGD
>2[4Fe-4S]ST1(bsup:BSPT1_II1200) Brucella suis bv. 2 PT09143
MTYVVTDNCIRCKYTDCVEVCPVDCFYEGENMLVINPDECIDCGVCEPECPAEAISPDTE
PGLDKWLELNAEYAAKWPNITAKKDALPEAKEMDGVAGKLEQYFSPEAGSGD
>2[4Fe-4S]ST1(bsuv:BSPT2_II1202) Brucella suis bv. 2 PT09172
MTYVVTDNCIRCKYTDCVEVCPVDCFYEGENMLVINPDECIDCGVCEPECPAEAISPDTE
PGLDKWLELNAEYAAKWPNITAKKDALPEAKEMDGVAGKLEQYFSPEAGSGD
>2[4Fe-4S]ST1(bsuc:BSSP2_II1211) Brucella suis bv. 2 Bs364CITA
MTYVVTDNCIRCKYTDCVEVCPVDCFYEGENMLVINPDECIDCGVCEPECPAEAISPDTE
PGLDKWLELNAEYAAKWPNITAKKDALPEAKEMDGVAGKLEQYFSPEAGSGD
>2[4Fe-4S]ST1(bsz:DK67_575) Brucella suis bv. 3
MTYVVTDNCIRCKYTDCVEVCPVDCFYEGENMLVINPDECIDCGVCEPECPAEAISPDTE
PGLDKWLELNAEYAAKWPNITAKKDALPEAKEMDGVAGKLEQYFSPEAGSGD
>2[4Fe-4S]ST1(bsw:IY71_08675) Brucella suis ZW043
MTYVVTDNCIRCKYTDCVEVCPVDCFYEGENMLVINPDECIDCGVCEPECPAEAISPDTE
PGLDKWLELNAEYAAKWPNITAKKDALPEAKEMDGVAGKLEQYFSPEAGSGD
>2[4Fe-4S]ST1(bsg:IY72_14080) Brucella suis ZW046
MTYVVTDNCIRCKYTDCVEVCPVDCFYEGENMLVINPDECIDCGVCEPECPAEAISPDTE
PGLDKWLELNAEYAAKWPNITAKKDALPEAKEMDGVAGKLEQYFSPEAGSGD
>2[4Fe-4S]ST1(bsg:IY72_08440) Brucella suis ZW046
MTYVVTDNCIRCKYTDCVEVCPVDCFYEGENMLVINPDECIDCGVCEPECPAEAISPDTE
PGLDKWLELNAEYAAKWPNITAKKDALPEAKEMDGVAGKLEQYFSPEAGSGD
>2[4Fe-4S]ST1(bcs:BCAN_A1805) Brucella canis ATCC 23365
MTYVVTDNCIRCKYTDCVEVCPVDCFYEGENMLVINPDECIDCGVCEPECPAEAISPDTE

PGLDKWLELNAEYAAKWPNITAKKDALPEAKEMDGVAGKLEQYFSPEAGSGD

>2[4Fe-4S]ST1(bsk:BCA52141_I2374) Brucella canis HSK A52141

MTYVVTDNCIRCKYTDCVEVCPVDCFYEGENMLVINPDECIDCGVCEPECPAEAISPDTE

PGLDKWLELNAEYAAKWPNITAKKDALPEAKEMDGVAGKLEQYFSPEAGSGD

>2[4Fe-4S]ST1(bcar:DK60_1764) Brucella canis RM6/66

MTYVVTDNCIRCKYTDCVEVCPVDCFYEGENMLVINPDECIDCGVCEPECPAEAISPDTE

PGLDKWLELNAEYAAKWPNITAKKDALPEAKEMDGVAGKLEQYFSPEAGSGD

>2[4Fe-4S]ST1(bcas:DA85_08480) Brucella canis SVA13

MTYVVTDNCIRCKYTDCVEVCPVDCFYEGENMLVINPDECIDCGVCEPECPAEAISPDTE

PGLDKWLELNAEYAAKWPNITAKKDALPEAKEMDGVAGKLEQYFSPEAGSGD

>2[4Fe-4S]ST1(bpv:DK65_1741) Brucella pinnipedialis 6/566

MTYVVTDNCIRCKYTDCVEVCPVDCFYEGENMLVINPDECIDCGVCEPECPAEAISPDTE

PGLDKWLELNAEYAAKWPNITAKKDALPEAKEMDGVAGKLEQYFSPEAGSGD

>2[4Fe-4S]ST1(bcet:V910_100266) Brucella ceti TE10759-12

MTYVVTDNCIRCKYTDCVEVCPVDCFYEGENMLVINPDECIDCGVCEPECPAEAISPDTE

PGLDKWLELNAEYAAKWPNITAKKDALLEAKEMDGVAGKLEQYFSPEAGSGD

>2[4Fe-4S]ST1(oan:Oant_1133) Ochrobactrum anthropi ATCC 49188

MTYVVTDNCIRCKYTDCVEVCPVDCFYEGENMLVINPDECIDCGVCEPECPAEAISPDTE

PGLDKWLELNTEYAAKWPNITAKKDALPEAKEMDGVAGKLEKYFSAEPGSGD

>2[4Fe-4S]ST1(oah:DR92_684) Ochrobactrum anthropi OAB

MTYVVTDNCIRCKYTDCVEVCPVDCFYEGENMLVINPDECIDCGVCEPECPAEAISPDTE

PGLDKWLELNTEYAAKWPNITAKKDALPEAKEMDGVAGKLEKYFSAEPGSGD

>2[4Fe-4S]ST1(ops:A8A54_09000) Ochrobactrum pseudogrignonense

MTYVVTDNCIRCKYTDCVEVCPVDCFYEGENMLVINPDECIDCGVCEPECPAEAISPDTE

PGLDKWLELNAEYAAKWPNLTAKKDELPEAKEMDGVTGKLEQFFSAEPGSGD

>2[4Fe-4S]ST1(bja:bll0157) Bradyrhizobium diazoefficiens USDA 110

MTYVVTENCIKCKYTDCVEVCPVDCFYEGDNMLVIHPDECIDCGVCEPECPADAIKPDTE
PGLEKWLSVNADYAKSWPNITQKKESPADAKEFDGMEGKFEKYFSPNPGSGD
>2[4Fe-4S]ST1(bju:BJ6T_01730) Bradyrhizobium japonicum USDA 6
MTYVVTENCIKCKYTDCVEVCPVDCFYEGDNMLVIHPDECIDCGVCEPECPADAIKPDTE
PGLEKWLQVNADYAKSWPNITQKKESPPDAKEFDGQEGKFEKYFSPNPGSGD
>2[4Fe-4S]ST1(bjp:RN69_00815) Bradyrhizobium japonicum E109
MTYVVTENCIKCKYTDCVEVCPVDCFYEGDNMLVIHPDECIDCGVCEPECPADAIKPDTE
PGLEKWLQVNADYAKSWPNITQKKESPPDAKEFDGQEGKFEKYFSPNPGSGD
>2[4Fe-4S]ST1(bbt:BBta_7544 K05524 Bradyrhizobium sp. BTAi1
MTYVVTENCIKCKYTDCVEVCPVDCFYEGDNMLVIHPDECIDCGVCEPECPADAIKPDTE
PGLEKWLGVNAEYAKAWPNITQKKDPPGDAKEHDGEAGKFEKYFSPKPGAGD
>2[4Fe-4S]ST1(aol:S58_70010) Bradyrhizobium oligotrophicum
MTYVVTENCIKCKYTDCVEVCPVDCFYEGDNMLVIHPDECIDCGVCEPECPADAIKPDTE
PGLEKWLGVNAEYAKSWPNITQKKDSPADAKEHDGEDGKFEKYFSSKPGAGD
>2[4Fe-4S]ST1(brc:BCCGELA001_03065) Bradyrhizobium sp. CCGE-LA001
MTYVVTENCIKCKYTDCVEVCPVDCFYEGDNMLVIHPDECIDCGVCEPECPADAIKPDTE
PGLEKWLGVNADYAKSWPNITQKKESPADAKEFDGMEGKFEKYFSPNPGSGD
>2[4Fe-4S]ST1(brad:BF49_0621) Bradyrhizobium sp. BF49
MTYVVTEACIKCKYTDCVEVCPVDCFYEGENMLVIHPDECIDCGVCEPECPADAIKPDTE
PGLEKWLTVNADYAKSWPNITQKKESPADAKDFDGMEGKFDKYFSSNPGSGD
>2[4Fe-4S]ST1(bic:LMTR13_01825)Bradyrhizobium icense
MTYVVTEACIKCKYTDCVEVCPVDCFYEGENMLVIHPDECIDCGVCEPECPADAIKPDTE
PGLEKWLEVNTEYAKSWPNITQKKDAPPDAKEFEGVEGKFEKYFSKEPGAGD
>2[4Fe-4S]ST1(bic:LMTR13_09220)Bradyrhizobium icense
MTYVVTEACIKCKYTDCVEVCPVDCFYEGDNMLVIHPDECIDCGVCEPECPADAIKPDTE
PGLEKWLALNARFAAVWPNISDKKAPPADAEEFQGAEEKFDKFFSEAPGLGS

>2[4Fe-4S]ST1(rpa:RPA0489)Rhodopseudomonas palustris CGA009
MTYVVTENCIKCKYTDCVEVCPVDCFYEGDNMLVIHPDECIDCGVCEPECPAEAIKPDTE
PGLEKWLELNSEYAKTWPNLTQKKDAPADAKEFDGQAGKFEKYFSSEPGSGD
>2[4Fe-4S]ST1(rpc:RPC_0547)Rhodopseudomonas palustris BisB18
MTYVVTEACIKCKYTDCVEVCPVDCFYEGDNMLVIHPDECIDCGVCEPECPADAIKPDTE
PGLEKWLEVNAEYSKTWPNITQKKDAPADAKEFESVEGKFDKYFSAEPGTGD
>2[4Fe-4S]ST1(rpd:RPD_0279)Rhodopseudomonas palustris BisB5
MTYVVTENCIKCKYTDCVEVCPVDCFYEGDNMLVIHPDECIDCGVCEPECPADAIKPDTE
PGLEKWLELNTEYAKSWPNLTQKKEAPGDAKQYEGMEGKFEKFFSPEPGTGD
>2[4Fe-4S]ST1(rpe:RPE_0125)Rhodopseudomonas palustris BisA53
MTYVVTENCIKCKYTDCVEVCPVDCFYEGENMLVIHPDECIDCGVCEPECPADAIKPDTE
PGLEQWLSLNAEHAKSWPNITQKKDAPADAKSFDGAEGKFDKYFSAEPGSGD
>2[4Fe-4S]ST1(rpt:Rpal_0490)Rhodopseudomonas palustris TIE-1
MTYVVTENCIKCKYTDCVEVCPVDCFYEGDNMLVIHPDECIDCGVCEPECPAEAIKPDTE
PGLEKWLELNSEYAKTWPNLTQKKDAPADAKEFDGQAGKFEKYFSSEPGSGD
>2[4Fe-4S]ST1(rpx:Rpdx1_0135)Rhodopseudomonas palustris DX-1
MTYVVTENCIKCKYTDCVEVCPVDCFYEGENMLVIHPDECIDCGVCEPECPAEAIKPDTE
PGLEKWLELNAEHAKTWPNLTQKKDAPADAKEFDGQAGKFDKYFSSEPGSGD
>2[4Fe-4S]ST1(nwi:Nwi_2810)Nitrobacter winogradskyi
MTFVVTENCIKCKYMDCVEVCPVDCFYEGDNMLVINPDECIDCGVCEPECPAEAIKPDSE
PDLENWLKLNAEYSAVWPNITIKRDAPADAKTFDGVAGKLEQYFSANPGQGD
>2[4Fe-4S]ST1(nwi:Nwi_0411)Nitrobacter winogradskyi
MTYVVNDACIKCKYTDCVEVCPVDCFYEGENMLVIHPDECIDCGVCEPECPAEAIKPDSE
PGVEKWLEVNAEYAGRWPNITQKKETPPDAKDFEGQEGKFEKYFSPDPGTGD
>2[4Fe-4S]ST1(nha:Nham_3630)Nitrobacter hamburgensis
MRLRQGSRRSSSCSCPRGVFEMTFVVTENCIKCKYMDCVEVCPVDCFYEGDNMLVINPDE

CIDCGVCEPECPAEAIFADSEPGLENWLKLNAEYAAVWPNITIKRDAPADAKAFDGVADK
LEQYFSANPGTGD
>2[4Fe-4S]ST1(nha:Nham_0506)Nitrobacter hamburgensis
MTYVVNDACIKCKYTDCVEVCPVDCFYEGENMLVIHPDECIDCGVCEPECPAEAIKPDSE
PGLEKWLELNADYAKSWPNLTQKKDAPPDAKEFEGQEGKFEKYFSPNPGTGD
>2[4Fe-4S]ST1(bos:BSY19_153)Bosea sp. RAC05
MTYVVTENCIKCKYMDCVEVCPVDCFYEGENMLVIHPDECIDCGVCEPECPAEAIKPDTE
PGLESWLQLNSKYASSWPNITQKKDAPADAKAFDGVAGKLEAHFSANPGEGD
>2[4Fe-4S]ST1(bvv:BHK69_02370)Bosea vaviloviae
MTYVVTENCIKCKYMDCVEVCPVDCFYEGENMLVIHPDECIDCGVCEPECPAEAIKPDTE
PGLESWLQLNSKYASSWPNITQKKDAPPDAKAFDGVAGKLDEHFSPEPGEGD
>2[4Fe-4S]ST1(xau:Xaut_4208)Xanthobacter autotrophicus
MAYVVTENCIRCTYMDCVSVCPVDCFYAGENMLVIHPDECIDCGVCEPECPAAAIFPDSD
PRAGDWAALNAQYAAQWPNITEKGAPPDDADAWKDAADKRALLSAAPAAI
>2[4Fe-4S]ST1(xau:Xaut_2641)Xanthobacter autotrophicus
MTYVVTENCIRCKYMDCVSVCPVDCFYEGENMLVIHPDECIDCGVCEPECPAEAIKPDAE
LGLEKWLALNAEYAKAWPNITLKRDPPADAKEWDGKPGKEELFSPEPGQGD
>2[4Fe-4S]ST1(xau:Xaut_2087)Xanthobacter autotrophicus
MAYVVTDNCIRCKFMDCVAVCPVDCFYEGENMLVINPDECIDCGVCEPECPAAAIAADTA
PEAGPWIALNAQYAALWPNIAEKKEPPADAAQWMDVADKFESAFSPAPGQADGSSASGAA
>2[4Fe-4S]ST1(azc:AZC_0232)Azorhizobium caulinodans
MAYVVTENCILCKYTDCVAVCPVDCFYEGENMLVIHPDECIDCGVCEPECPAEAIKPDTE
PGLEKWLSLNAEYAKTWPNITLKRDALPDAKEWDGKPGKEDKFSAEPGTGD
>2[4Fe-4S]ST1(azc:AZC_2802)Azorhizobium caulinodans
MAYVVTDGCIRCKYMDCVSVCPVDCFYAGENMLVIHPDECIDCGVCEPECPAEAIVPDTD
PRAGEWLALNAEYAATWPNITEKGEPPADADDWKGKAGKLALLDPAPAA

>2[4Fe-4S]ST1(mex:Mext_3879)Methylorubrum extorquens PA1
MTYVVTDNCIKCKYMDCVEVCPVDCFYEGENMLVIHPDECIDCGVCEPECPAEAIKPDTE
GDLESWLKLNADYAKTWPNITQKKDAPSDAKQWDGVSGKLEAHFSPNPGSGD
>2[4Fe-4S]ST1(mea:Mex_1p4253)Methylorubrum extorquens AM1
MTYVVTDNCIKCKYMDCVEVCPVDCFYEGENMLVIHPDECIDCGVCEPECPAEAIKPDTE
GDLESWLKLNADYAKTWPNITQKKDAPSDAKQWDGVSGKLEAHFSPNPGSGD
>2[4Fe-4S]ST1(mdi:METDI4865)Methylorubrum extorquens DM4
MTYVVTDNCIKCKYMDCVEVCPVDCFYEGENMLVIHPDECIDCGVCEPECPAEAIKPDTE
GDLESWLKLNADYAKTWPNITQKKDAPSDAKQWDGVSGKLEAHFSPNPGSGD
>2[4Fe-4S]ST1(mch:Mchl_4189)Methylorubrum extorquens CM4
MTYVVTDNCIKCKYMDCVEVCPVDCFYEGENMLVIHPDECIDCGVCEPECPAEAIKPDTE
GDLESWLKLNADYAKTWPNITQKKDAPSDAKQWDGVSGKLEAHFSPNPGSGD
>2[4Fe-4S]ST1(mpo:Mpop_4336)Methylorubrum populi
MTYVVTDNCIKCKYMDCVEVCPVDCFYEGENMLVIHPDECIDCGVCEPECPAEAIKPDTE
GNLESWLKLNADYAKTWPNITQKKDAPTDAKQWDGVGGKLEAHFSPNPGSGD
>2[4Fe-4S]ST1(mrd:Mrad2831_3707)Methylobacterium radiotolerans
MTYVVTDNCIKCKYTDCVEVCPVDCFYEGENMLVIHPDECIDCGVCEPECPAEAIKPDTE
SNLDTWLKLNADYAKSWPNITQKKEAPADAKEWDGKTGKLEAHFSPNPGSGD
>2[4Fe-4S]ST1(met:M446_4804)Methylobacterium sp. 4-46
MTYVVTENCIKCKYMDCVEVCPVDCFYEGENMLVIHPDECIDCGVCEPECPAEAIKPDTE
PGLERWLKLNADFAKNWPNITQKKTAPSDAKEWDGVAGKFDAHFSSNPGTGD
>2[4Fe-4S]ST1(mno:Mnod_1719)Methylobacterium nodulans
MTYVVTENCIKCKYMDCVEVCPVDCFYEGENMLVIHPDECIDCGVCEPECPAEAIKPDTE
PGLEKWLKLNADLAKSWPNITQKKPAPADAKEWDGVAGKYEAHFSPNPGSGD
>2[4Fe-4S]ST1(mor:MOC_4232)Methylobacterium oryzae
MTYVVTDNCIKCKYTDCVEVCPVDCFYEGENMLVIHPDECIDCGVCEPECPAEAIKPDTE

SNLDTWLKLNADYAKTWPNITQKKEAPADAKEWDGKAGKLEAHFSPNPGSGD
>2[4Fe-4S]ST1(meta:Y590_19115)Methylobacterium sp. AMS5
MTYVVTDNCIKCKYMDCVEVCPVDCFYEGENMLVIHPDECIDCGVCEPECPAEAIKPDTE
GDLESWLKLNADYAKTWPNITQKKDAPSDAKQWDGVSGKLEAHFSPNPGSGD
>2[4Fe-4S]ST1(maqu:Maq22A_c25615)Methylobacterium aquaticum
MTYVVTDNCIKCKYMDCVEVCPVDCFYEGENMLVIHPDECIDCGVCEPECPAEAIKPDTE
PGLESWLKLNADMAKGWPNITQKKPAPADAKEWDGKTGKFEAHFSTNPGSGD
>2[4Fe-4S]ST1(msl:Msil_1370)Methylocella silvestris
MTYVVLENCIKCKYMDCVEVCPVDCFYEGENMLVIHPDECIDCGVCEPECPAEAIKPDTE
PGIEQWITLNADMAQSWPNITMKREAAPDAKQFDGRPGKFKEFFSPEPGKGD
>2[4Fe-4S]ST1(hdn:Hden_3159)Hyphomicrobium denitrificans ATCC 51888
MTYVVNEKCIKCKYTDCVEVCPVDCFYEGENMLVIHPDECIDCGVCEPECPADAIKPDTE
PGMERWLELNRQYADNWPNITAKKAALPDADDIKDEPGKFEKYFSPNAGSGD
>2[4Fe-4S]ST1(hdt:HYPDE_38973)Hyphomicrobium denitrificans 1NES1
MTYVVNDKCIKCKYTDCVEVCPVDCFYEGENMLVIHPDECIDCGVCEPECPADAIKPDTE
PGMERWLELNRQYSESWPNITSKKPALPGADDVKDEPGKFEKYFSANSGSGD
>2[4Fe-4S]ST1(hdt:HYPDE_41033)Hyphomicrobium denitrificans 1NES1
MTYVVDERCIKCKTTDCVEVCPVDCFYEGENMLVIHPDECIDCGVCRPECPVDAIKPDSE
PGMEKWLKLNSEYALKWPNITIKKDMPKDARDHWDETGKFEKYFSPNPGAGD
>2[4Fe-4S]ST1(hmc:HYPMC_0243)Hyphomicrobium sp. MC1
MTFVVTESCIRCKFQDCLQSCPATCFYEGKNMLVINPAECIDCGACEPECPAEAIVRDTA
PGAEKWVEFNRKYAALWPNIRKPTVVPDDAEDWIEVENKLETEFDPDPAAP
>2[4Fe-4S]ST1(hmc:HYPMC_4406)Hyphomicrobium sp. MC1
MTYVVNEKCIKCKYTDCVEVCPVDCFYEGENMLVIHPDECIDCGVCEPECPADAIKPDTE
PDMEKWMELNRQYADAWPNITAKKAALPDADAVKDEPNKFEKYFSPNPGSGD
>2[4Fe-4S]ST1(hni:W911_01685)Hyphomicrobium nitrativorans

MTYVVNEKCIKCKYTDCVEVCPVDCFYEGENMLVIHPDECIDCGVCEPECPADAIKPDTE
PGIERWLEVNREFADKWPNLTAKKAAYADADKYRDEEDKFDKYFSSEPGSGD
>2[4Fe-4S]ST1(phl:KKY_2936A)Pelagibacterium halotolerans
MTYVVTDNCIKCKYTDCVEVCPVDCFYEGENMLVIHPDECIDCGVCEPECPADAIKPDTE
PGLEKWLEVNTKYASIWPNLTEKRDELPEAKEMDGVEGKFEKFFSEEPGEGD
>2[4Fe-4S]ST1(fil:BN1229_v1_0756)Candidatus Filomicrobium marinum W
MTYVVNESCIKCKFTDCVEVCPVDCFYEGENMLVIHPDECIDCGVCEPECPVDAIKPDTE
PHLEKWLEVNREYADKWPNITSKKDALPEAEEHRDEDGKFEKYFSSEPGDGD
>2[4Fe-4S]ST1(fiy:BN1229_v1_0761)Candidatus Filomicrobium marinum Y
MTYVVNESCIKCKFTDCVEVCPVDCFYEGENMLVIHPDECIDCGVCEPECPVDAIKPDTE
PHLEKWLEVNREYADKWPNITSKKDALPEAEEHRDEDGKFEKYFSSEPGDGD
>2[4Fe-4S]ST1(deq:XM25_15710)Devosia sp. H5989
MTYVVTDNCIRCKYMDCVEVCPVDCFYEGENMLVIHPDECIDCGVCEPECPAEAIKPDTE
SGLDKWLEINTKYANVWPNITEKREAPADAKEMDGVADKFEKYFSEAPGEGD
>2[4Fe-4S]ST1(rhz:RHPLAN_40190)Rhodoplanes sp. Z2-YC6860
MIHVVNESCIKCKYTDCVQVCPVDCFHEGENMLVINPDDCIGCGVCVPECPLDAIKTELD
PGMERWLAVNALYAKQWPNIAMKREAPPDAKAWEDTPDKFEKYFSPNPGTGG
>2[4Fe-4S]ST1(rhz:RHPLAN_07640)Rhodoplanes sp. Z2-YC6860
MTYVVNDNCIKCKYTDCVEVCPVDCFYEGENMLVIHPDECIDCGVCEPECPAEAIKPDTE
PGLEKWLGLNAEYAKTWPNITVKKEAPADAKDWEGVAGKFEKFFSAEPGQGD
>2[4Fe-4S]ST1(mbry:B1812_01945)Methylocystis bryophila
MTYVVTENCIKCKYMDCVEVCPVDCFYEGANMLVIHPDECIDCGVCEPECPALAIKPDTE
PELEAWLTLNLEYAQVWPNVTIKREAPADAKEWDGKPGKLEAHFSPEPGQGD
>2[4Fe-4S]ST1(mey:TM49_01980)Martelella endophytica
MTYVVTDNCIKCKYTDCVEVCPVDCFYEGENFLVIHPDECIDCGVCEPECPAEAIRPDTE
PGLDKWLKLNADYADVWPNITIKKEPLEGAEEMDGVEGKFEKFFSEKPGAGD

>2[4Fe-4S]ST1(psin:CAK95_03725)Pseudorhodoplanes sinuspersici
MTFAVTEACIRCKYTDCVAVCPVDCFYEGENMLVIHPDECIDCGVCEPECPAEAIIADTA
EGAVRWVELNAKYAALWPNISDAKDPPPDAEDFNGKPDKFENHFSPQPAIEPAN
>2[4Fe-4S]ST1(psin:CAK95_10545)Pseudorhodoplanes sinuspersici
MTYVVNDSCIRCKFMDCVEVCPVDCFYEGENMLVIHPDECIDCGVCEPECPVEAIKPDTD
PGLEKWLSLNAEYAKVWPNITAKKEPPADHKEWEGVEGKEQYFSPEPGTGN
>2[4Fe-4S]ST1(oar:OA238_160p0450)Octadecabacter arcticus
MTYVVTESCIKCKYTDCVEVCPVDCFYEGENMLVIHPDECIDCGVCEPECPADAILADTD
QGAETWLELNSKFAAKWPNIIQQSEALANAEEYDGMENKFDKYFSSKPR
>2[4Fe-4S]ST1(otm:OSB_05850)Octadecabacter temperatus
MTYVVTDACINCKYQDCIEVCPVDCFYEGENTIVINTEECIDCGVCEPECPVDAIKPDTI
PNSAFWVELNQKHAESWPNISEKGTPPADADDWANKPDKLSLMSEKPAA
>2[4Fe-4S]ST1(pphr:APZ00_08695)Pannonibacter phragmitetus
MTYVVTDNCIKCKYTDCVEVCPVDCFYEGENMLVINPDECIDCGVCEPECPAEAILPDTE
PGLEKWIELNAEYAAKWPNITEKKDALPEAPEFDGIKNKLETHFSPNGPDGA
>2[4Fe-4S]ST1(lap:ACP90_01635)Labrenzia sp. CP4
MTYVVTDNCIKCKYTDCVEVCPVDCFYEGENFLVINPDECIDCGVCEPECPAEAILPDTE
PGLEKWIEVNAEYSEKWPNITEKKEPLPDAEEFDGKENKFEKYFSPNPAS
>2[4Fe-4S]ST1(lagg:B0E33_13150)Labrenzia aggregata
MTYVVTDNCIKCKYTDCVEVCPVDCFYEGENFLVINPDECIDCGVCEPECPAEAILPDTE
PGLEKWIEVNAEYSEKWPNITEKKEPLPDAEEFDGKENKFEKYFSPNPAS
>2[4Fe-4S]ST1(nar:Saro_0123)Novosphingobium aromaticivorans
MTYVVTDACIRCKFMDCVEVCPVDCFYEGENMLVINPSECIDCGVCEPECPAEAILPDTE
SGLEQWLELNAKYSAEWPNITAKKDAPADADEHKGEEGKFDKYFSAEPGEGD
>2[4Fe-4S]ST1(npp:PP1Y_AT208)Novosphingobium sp. PP1Y
MTYVVTDACIKCKYMDCVEVCPVDCFYEGENMLVINPSECIDCGVCEPECPAEAILPDTE

SGLEQWMELNTKYSAEWPNLTSKKDSPEDADEYKGVEGKFEKFFSPEPGEGD
>2[4Fe-4S]ST1(npn:JI59_11515)Novosphingobium pentaromativorans
MTYVVTDACIKCKYMDCVEVCPVDCFYEGENMLVINPSECIDCGVCEPECPAEAILPDTE
SGLEQWMELNTKYSAEWPNLTSKKDSPEDADEYKGVEGKFEKFFSPEPGEGD
>2[4Fe-4S]ST1(sal:Sala_2741)Sphingopyxis alaskensis
MTYVVTDACVRCKYMDCVEVCPVDCFYEGENMLVINPNECIDCGVCEPECPAEAILPDTE
SGLEKWLEVNSKFSAEWPNITVKKETPADADEYKGVEGKFEKFFSPEPGEGD
>2[4Fe-4S]ST1(sphk:SKP52_18275)Sphingopyxis fribergensis
MTYVVTDACVRCKYMDCVEVCPVDCFYEGENMLVINPNECIDCGVCEPECPAEAILPDTE
SGLEKWLEVNSKFSAEWPNITVKKETPADADEYKGVENKFETLFSPEPGAGD
>2[4Fe-4S]ST1(sphp:LH20_16645)Sphingopyxis sp. 113P3
MTYVVTDACIRCKYMDCVEVCPVDCFYEGENMLVINPNECIDCGVCEPECPAEAILPDTE
SGLEKWLEVNSKFSAEWPNITTKKDSPADADEYKGVDGKFEKFFSPEPGEGD
>2[4Fe-4S]ST1(smaz:LH19_18985)Sphingopyxis macrogoltabida 203
MTYVVTDACVRCKYMDCVEVCPVDCFYEGENMLVINPNECIDCGVCEPECPAEAILPDTE
SGLEKWLEVNTKFSAEWPNITVKKDSPADADEFKGVDDKFDKYFSPEPGEGD
>2[4Fe-4S]ST1(ster:AOA14_05595)Sphingopyxis terrae
MTYVVTDACIRCKYMDCVEVCPVDCFYEGENMLVINPNECIDCGVCEPECPAEAILPDTE
SGLEKWLEVNSKFSAEWPNITTKKDSPADADEYKGVDDKYEKFFSPEPGEGD
>2[4Fe-4S]ST1(sgi:SGRAN_3575)Sphingopyxis granuli
MTYVVTDACIRCKYMDCVEVCPVDCFYEGENMLVINPNECIDCGVCEPECPAEAILPDTE
NGLEQWLELNAKFSADWPNITTKKESPADADEWKGVDGKFDKFFSPEPGEGD
>2[4Fe-4S]ST1(sphl:LPB140_05885)Sphingopyxis sp. LPB0140
MTYVVTDACIRCKYMDCVEVCPVDCFYEGENMLVINPSECIDCGVCEPECPAEAILPDTE
DGLEKWLELNTVYSAEWPNITTSKEPPADADEFKNVEDKFDKYFSSEPGEGD
>2[4Fe-4S]ST1(swi:Swit_3538)Sphingomonas wittichii

MTYVVTDACIRCKYMDCVEVCPVDCFYEGDNMLVINPSECIDCGVCEPECPAEAILPDTE

SGLEQWLELNNTFAAQWPNITRKREAPADADEWKNVEGKYEKHFSPEPGQGD

>2[4Fe-4S]ST1(sphm:G432_14530)Sphingomonas sp. MM-1

MTYVVTDACIRCKYMDCVEVCPVDCFYEGENMLVINPNECIDCGVCEPECPAEAILPDTE

SGLEKWLEVNATFSAQWPNLTRKGEQPADADEHKGEEGKYEKYFSPEPGQGD

>2[4Fe-4S]ST1(sphi:TS85_18695)Sphingomonas sp. WHSC-8

MTYVVTDACIRCKYMDCVEVCPVDCFYEGENMLVINPSECIDCGVCEPECPAEAIFPDTE

NGLEKWLELNRTYSEQWPNITQKGDVPDDADSFKGVEGKEAEFSPEPGTGS

>2[4Fe-4S]ST1(sjp:SJA_C1-27340)Sphingobium japonicum

MTYVVTDNCIRCKYMDCVEVCPVDCFYEGENMLVINPNECIDCGVCEPECPAEAILPDTE

NGLEKWLELNTKFSAEWPNITVKGEAPADADDMKGIENKFEQFFSPEPGAGS

>2[4Fe-4S]ST1(sch:Sphch_1458)Sphingobium chlorophenolicum

MTYVVTDNCIRCKYMDCVEVCPVDCFYEGENMLVINPNECIDCGVCEPECPAEAILPDTE

NGLEKWLELNTKFSAEWPNITVKGDAPADADDMKGVENKFEQFFSPEPGAGS

>2[4Fe-4S]ST1(ssy:SLG_34020)Sphingobium sp. SYK-6

MTYVVTDACIRCKYMDCVEVCPVDCFYEGENMLVINPNECIDCGVCEPECPAEAILPDTE

SGLEQWLELNTRYSAEWPNITAKGEPPADADAMKGETGKFEKYFSPEPGEGS

>2[4Fe-4S]ST1(syb:TZ53_13880)Sphingobium sp. YBL2

MTYVVTDNCIRCKYMDCVEVCPVDCFYEGENMLVINPNECIDCGVCEPECPAEAILPDTE

NGLEKWLELNTKFSAEWPNITVKGEAPADADEMKGIENKLEKFFSPEPGAGS

>2[4Fe-4S]ST1(sbd:ATN00_19100)Sphingobium baderi

MTYVVTDNCIRCKYMDCVEVCPVDCFYEGENMLVINPNECIDCGVCEPECPAEAILPDTE

NGLEKWLELNTKFSAEWPNITVKGEAPADADDMRGVENKLEQFFSPEPGSGD

>2[4Fe-4S]ST1(spmi:K663_05250)Sphingobium sp. MI1205

MTYVVTDNCIRCKYMDCVEVCPVDCFYEGENMLVINPSECIDCGVCEPECPAEAILPDTE

NGLEKWLELNTKFSAEWPNITVKGDAPADADEMKGIENKLEKFFSPEPGSGS

>2[4Fe-4S]ST1(sphb:EP837_00701)Sphingobium sp. EP60837

MTYVVTDNCIRCKYMDCVEVCPVDCFYEGENMLVINPNECIDCGVCEPECPAEAILPDTE

NGLEKWLELNTKFSAEWPNITVKGEAPADADDMKGVENKLEKFFSPEPGSGS

>2[4Fe-4S]ST1(sphr:BSY17_2248)Sphingobium sp. RAC03

MTYVVTDNCIRCKYMDCVEVCPVDCFYEGENMLVINPSECIDCGVCEPECPAEAILPDTE

NGLEKWLELNTKYSAEWPNITVKGDAPADADAMKGVENKLEQYFSPEPGTGS

>2[4Fe-4S]ST1(sinb:SIDU_01160)Sphingobium indicum

MTYVVTDNCIRCKYMDCVEVCPVDCFYEGENMLVINPNECIDCGVCEPECPAEAILPDTE

NGLEKWLELNTKFSAEWPNITVKGDAPADADDMKGVENKFEQFFSPEPGAGS

>2[4Fe-4S]ST1(cij:WG74_02790)Citromicrobium sp. JL477

MTYVVTDACIKCKYTDCVEVCPVDCFYEGENMLVINPSECIDCGVCEPECPAEAILPDTE

DNLEKWLELNTKFSNEWPNITQKKDPPEDADEHKGEEGKFEKFFNPEPGEGD

>2[4Fe-4S]ST1(sphg:AZE99_01690)Sphingorhabdus sp. M41

MTYVVTEDCIKCKYMDCVEVCPVDCFYEGDNMLVINPSECIDCGVCEPECPAEAILPDTE

DGLEKWLEINTKYSEEWPNITVSRDPPADAEEFKGMEGKFEAFFSPKPGAGD

>2[4Fe-4S]ST1(blas:BSY18_2670)Blastomonas sp. RAC04

MTYVVTDACIKCKYMDCVEVCPVDCFYEGENMLVINPSECIDCGVCEPECPAEAILPDTE

DGLEKWLELNTKFSAEWPNITVKRESPADADEYKGKDGKYEAFFSPEPGEGD

>2[4Fe-4S]ST1(eli:ELI_13125)Erythrobacter litoralis HTCC2594

MTYVVTDACIKCKYTDCVEVCPVDCFYEGDNMLVINPSECIDCGVCEPECPAEAILPDTE

DGLEKWLEINTKFSADWPNITQKKEPPADADEHKGEEDKYEKYFSAEPGEGD

>2[4Fe-4S]ST1(elq:Ga0102493_11964)Erythrobacter litoralis DSM 8509

MTYVVTEDCIKCKYTDCVEVCPVDCFYEGENMLVINPSECIDCGVCEPECPAEAILPDTE

DGLEKWLELNTKYSAEWPNITSQKEPPEDADDHKGETGKFEKYFSADPGEGD

>2[4Fe-4S]ST1(aay:WYH_02318)Altererythrobacter atlanticus

MTYVVTDACIKCKYTDCVEVCPVDCFYEGENMLVINPSECIDCGVCEPECPAEAILPDTE

GGLEKWLELNTKFSAEWPNITEKKDPPADADDHKGEDGKFDKYFSEEPGEGD

>2[4Fe-4S]ST1(amx:AM2010_2224)Altererythrobacter marensis

MTYVVTDACIKCKYTDCVEVCPVDCFYEGENMLVINPSECIDCGVCEPECPAEAILPDTE

DGLEKWLELNTKYSAEWPNITTQKEPPADADDHKGEDGKFDKFFSPEPGEGD

>2[4Fe-4S]ST1(aep:AMC99_02566)Altererythrobacter epoxidivorans

MTYVVTDACIRCKYTDCVEVCPVDCFYEGENMLVINPSECIDCGVCEPECPAEAILPDTE

DGLEKWLELNTKFSAEWPNITSQKEPPADADEHKGEEGKFEKYFTPEPGEGD

>2[4Fe-4S]ST1(ado:A6F68_00457)Altererythrobacter dongtanensis

MTYVVTDACIKCKYTDCVEVCPVDCFYEGETMLVINPSECIDCGVCEPECPAEAILPDTE

DGLEKWLELNTKFSAEWPNITQKKDPPEDADEHKGEEGKFEKFFTPEPGEGD

>2[4Fe-4S]ST1(pns:A9D12_04415)Porphyrobacter neustonensis

MTYVVTEDCIKCKYTDCVEVCPVDCFYEGENMLVINPSECIDCGVCEPECPAEAILPDTE

DGLEKWLELNTQYSAIWPNITSQKAPPEDADAHKGEKGKFELYFSAEPGEGD

>2[4Fe-4S]ST1(gdi:GDI2031)Gluconacetobacter diazotrophicus PA1 5 (Brazil)

MTYVVTENCIRCKFMDCVEVCPVDCFYAGENFLVINPDECIDCGVCEPECPAEAIVPDSD

DRAAAWAEINASYSAKWPNITRKGTAPADAEEWKDKPGKKDLLSPEPHKD

>2[4Fe-4S]ST1(gdj:Gdia_0255)Gluconacetobacter diazotrophicus PA1 5 (JGI)

MTYVVTENCIRCKFMDCVEVCPVDCFYAGENFLVINPDECIDCGVCEPECPAEAIVPDSD

DRAAAWAEINASYSAKWPNITRKGTAPADAEEWKDKPGKKDLLSPEPHKD

>2[4Fe-4S]ST1(aace:A0U92_12060)Acetobacter aceti

MTYVVTENCIRCKFMDCVEVCPVDCFYAGENFLVINPDECIDCGVCEPECPAEAIFPDSD

DRATAWAEINTKYSALWPNITRKGTQPADAEEWKDKPNKAELLSPEPHKD

>2[4Fe-4S]ST1(mgy:MGMSRv2__3124)Magnetospirillum gryphiswaldense MSR-1 v2

MAYVVTENCIKCKYQDCVEVCPVDCFYEGENFLVINPDECIDCGVCEPECPAEAIVPDSD

DKAAAWAQLNRDYSGQWPNITRKGDAPADADAWKNKPDKADLLSPNPGKGTV

>2[4Fe-4S]ST1(azl:AZL_002430)Azospirillum sp. B510

MPYVVTDGCIKCKYTDCVEVCPVDCFYEGENMLVIHPDECIDCGVCEPECPAEAIVPDTD
DRATKWLELNRDYSGQWPNITRKKDAPADADTFKGVDGKFEKFFSPKAGG
>2[4Fe-4S]ST1(abs:AZOBR_10204)Azospirillum brasilense Sp245
MPYVVTEDCIKCKYTDCVEVCPVDCFYEGENMLVIHPDECIDCGVCEPECPAEAIVPDTD
GRAEKWMELNRDYAAQWPNLTRKKDALPDADAMKGVDGKFDKFFSPKAGG
>2[4Fe-4S]ST1(abq:ABAZ39_00315)Azospirillum brasilense Az39
MPYVVTEDCIKCKYTDCVEVCPVDCFYEGENMLVIHPDECIDCGVCEPECPAEAIVPDTD
GRAEKWMELNRDYAAQWPNLTRKKDALPDADAMKGVDGKFDKFFSPKAGG
>2[4Fe-4S]ST1(magq:MGMAQ_3732)Magnetospira sp. QH-2
MTYIVIENCIKCKYQDCVEVCPVDCFYEGENFLVIHPDECIDCGVCEPECPAEAIVPDTE
DGVEEWLEINTKYAEIWPNITRKGDAPADAADWDGKPDKKALLSTKPGAGTGEG
>2[4Fe-4S]ST1(pgv:SL003B_0397)Polymorphum gilvum
MTYVVTDNCIKCKYTDCVEVCPVDCFYEGENMLVINPDECIDCGVCEPECPAEAILPDTE
PGLEKWIEINAEYAAKWPNITVKKDPLPEAAEFDGKAGKFEQYFSPNGPDGK
>2[4Fe-4S]ST1(rcp:RCAP_rcc02791)Rhodobacter capsulatus
MTYVVTDNCIACKYTDCVEVCPVDCFYEGENTLVIHPDECIDCGVCEPECPADAIRPDTE
PGMEDWVEFNRTYASQWPVITIKKDPMPDHKKYDGETGKREKYFSPNPGTGD
>2[4Fe-4S]ST1(kvu:EIO_0821)Ketogulonicigenium vulgare Y25
MTYVVTENCIACKYTDCVEVCPVDCFYEGENTLVIHPDECIDCGVCEPECPADAIRPDTE
PDMEKWVEFNRKYSEMWPVITSRRDPLPGYEEMDGKPGKLALLSENPGLGG
>2[4Fe-4S]ST1(kvl:KVU_0354)Ketogulonicigenium vulgare WSH-001
MTYVVTENCIACKYTDCVEVCPVDCFYEGENTLVIHPDECIDCGVCEPECPADAIRPDTE
PDMEKWVEFNRKYSEMWPVITSRRDPLPGYEEMDGKPGKLALLSENPGLGG
>2[4Fe-4S]ST1(kro:BVG79_00609)Ketogulonicigenium robustum
MTYVVTENCIACKYTDCVEVCPVDCFYEGENTLVIHPDECIDCGVCEPECPADAIRPDTE
PGMEKWVEFNRKYAEMWPVITSRRDPLPGYQEMDGKPGKLDLLSENPGEGG

>2[4Fe-4S]ST1(otm:OSB_13120)Octadecabacter temperatus
MTYVVTDACIQCKFTDCVEVCPVDCFYEGENTLVIAPDECIDCGVCEPECPADAIIPDTE
SAAEKWIAFNGKYAALWPVIIEKKEPLENADDFVGVEGKLEKYFSEAPAE
>2[4Fe-4S]ST1(red:roselon_02010) Roseibacterium elongatum
MTYVVTENCIACKYTDCVEVCPVDCFYEGENCLVIHPDECIDCGVCEPECPADAIRPDTE
PEMEKWVEINRKYAEMWPVIITRKDPLPTAEEMDGKPGKMELLSENPGEGG
>2[4Fe-4S]ST1(cid:P73_4318) Celeribacter indicus
MTYVVTENCIACKFMDCVEVCPVDCFYEGENTLVIHPDECIDCGVCEPECPADAIRPDTE
PDMDKWVAFNRRYAEVWPVILERGEPPANADEMDGVPNKLETLFSPKPGNS
>2[4Fe-4S]ST1(con:TQ29_14735)Confluentimicrobium sp. EMB200-NS6
MTYAVTDNCIGCKYTDCVSVCPVDCFYEGVNMLVINPEECIDCGVCEPECPADAIRADTD
EEGAKWVAFNARYASLWPVIAAAKEPLPGAEANDGLPGKRDTIFSPDPDPQTA
>2[4Fe-4S]ST1(paby:Ga0080574_TMP5033) Pelagibaca abyssi
MTYVVTENCIACKYMDCVEVCPVDCFYEGENTLVIHPDECIDCGVCEPECPAEAISPDTD
PDMDRWVEFNRKYSEIWPVILERGEPPENADAMDGVPDKLKTMFSPNPGNGS
>2[4Fe-4S]ST1(apb:SAR116_1071)Candidatus Puniceispirillum marinum
MTYIVNENCINCKYTDCVEVCPVDCFYEGENMLVIHPDECIDCGVCEPECPPEAILPDSE
PEATKWLDLNRDMSEIWPNIGQKIDEMPNAKAAESETGKFDKYFTKAPGKGN
>2[4Fe-4S]ST1(rsp:RSP_2424)Rhodobacter sphaeroides 2.4.1
MTYVVTDNCIACKYTDCVEVCPVDCFYEGENMLVIHPDECIDCGVCEPECPADAIRPDTE
PDMESWVELNRKYAEVWPVIVTKKDPLPEATDLDGQPGKLATHFSEKPGEGG
>2[4Fe-4S]ST1(rsh:Rsph17029_1088)Rhodobacter sphaeroides ATCC 17029
MTYVVTDNCIACKYTDCVEVCPVDCFYEGENMLVIHPDECIDCGVCEPECPADAIRPDTE
PDMESWVELNRKYAEVWPVIVTKKDPLPEATDLDGQPGKLATHFSEKPGEGG
>2[4Fe-4S]ST1(rsq:Rsph17025_1558)Rhodobacter sphaeroides ATCC 17025
MAYVVTDNCIACKYTDCVEVCPVDCFYEGENMLVIHPDECIDCGVCEPECPADAIRPDTE

PDMESWLELNRKYAEIWPVIVTKKDPLPEAADLDGQTGKLATHFSEKPGEGG

>2[4Fe-4S]ST1(rsk:RSKD131_0739)Rhodobacter sphaeroides KD131

MTYVVTDNCIACKYTDCVEVCPVDCFYEGENMLVIHPDECIDCGVCEPECPADAIRPDTE

PDMESWVELNRKYAEVWPVIVTKKDPLPEATDLDGQPGKLATHFSEKPGEGG

>2[4Fe-4S]ST1(pde:Pden_1826)Paracoccus denitrificans

MTYVVTENCIMCKYTDCVEVCPVDCFYEGENTLVIHPDECIDCGVCEPECPADAIRPDTE

PDMDKWVEFNRKYAESWPVITRKKDPMPGYQEMDGAPGKLEKYFSEAPGEGD

>2[4Fe-4S]ST1(pami:JCM7686_1338)Paracoccus aminophilus

MTYVVTENCIMCKFTDCVEVCPVDCFYEGENTLVIHPDECIDCGVCEPECPADAIRPDTE

PDMEKWVEFNRKYSELWPVITRKKDPMPGYDDMDGKPGKLEQYFSEAPGEGD

>2[4Fe-4S]ST1(pye:A6J80_19220)Paracoccus yeei

MTYVVTDNCIMCKYTDCVEVCPVDCFYEGENTLVIHPDECIDCGVCEPECPADAIRPDTE

PDMEQWVEFNRKYSELWPVITRKKDPMPGYQEMDGQPGKLEKYFSPNPGEGD

>2[4Fe-4S]ST1(tom:BWR18_09485) Tateyamaria omphalii

MTYIVNDSCIACKYTDCVEVCPVDCFYEGENMLVIHPDECIDCGVCEPECPADAIRPDTE

PDMEKWVEFNRKYSEMWPVIITKKDPLPTAEEMDGKEGKMELFSENPGEGG

>2[4Fe-4S]ST1(thw:BMG03_16520) Thioclava nitratireducens

MTYVVIDNCINCKYTDCVEVCPVDCFYEGENTLVIHPDECIDCGVCEPECPADAIRPDTE

PGMESWVELNRKYSELWPVITQKKDPMPEAEKYDGESGKLEKYFSEAPGEGD

>2[4Fe-4S]ST1(lvs:LOKVESSMR4R_02048) Yoonia vestfoldensis

MTYIVNDNCIACKYTDCVEVCPVDCFYEGENMLVIHPDECIDCGVCEPECPADAIRPDTE

PDMEKWVEFNRKYSEMWPVIITKKDPLPGAEDMDGKPGKLDLFSEAPGEGG

>2[4Fe-4S]ST1(jan:Jann_3309)Jannaschia sp. CCS1

MTYIVNDACIACKYTDCVEVCPVDCFYEGENMLVIHPDECIDCGVCEPECPADAIRPDTE

PDMDKWVEFNRKYSEMWPVIITKKDPLPTADDMDGKPGKMELFSEAAGEGG

>2[4Fe-4S]ST1(oar:OA238_c27270)Octadecabacter arcticus

MTYIVNDSCIACKYTDCVEVCPVDCFYEGENMLVIHPDECIDCGVCEPECPADAIRPDTE
PDMEKWVEFNRKYSEMWPVIITKKDQLPNAEEMDGKEDKMELFSEAPGEGG
>2[4Fe-4S]ST1(otm:OSB_09470)Octadecabacter temperatus
MTYIVNDACIACKYTDCVEVCPVDCFYEGENMLVIHPDECIDCGVCEPECPADAIRPDTE
PDMEKWVEFNRKYSEKWPVIITKKDQLPTAEEMDGKEGKMDLFSEAPGEGG
>2[4Fe-4S]ST1(cid:P73_3279)Celeribacter indicus
MTYVVIENCIACKYTDCVEVCPVDCFYEGENMLVIHPDECIDCGVCEPECPADAIRPDTE
PDMEKWVEFNRKYSEQWPVILAKKDPLPGYEEKDGEDGKLEKYFSEAAGEGS
>2[4Fe-4S]ST1(cmar:IMCC12053_867) Celeribacter marinus
MTYVVIENCIACKYTDCVEVCPVDCFYEGENMLVIHPDECIDCGVCEPECPADAIRPDTE
PDMEKWVEFNRKYSEAWPVILSKKDPMPGYEEKDGETGKMEKYFSEAAGEGDA
>2[4Fe-4S]ST1(rsu:NHU_00572) Rhodovulum sulfidophilum
MTYVVTDNCIACKYTDCVEVCPVDCFYEGENMLVIHPDECIDCGVCEPECPADAIRPDTE
PDMDEWVEFNRKYAETWPCIITRKDPLPEAEERDGEEGKRAKYFSETPGAGG
>2[4Fe-4S]ST1(rhc:RGUI_1920) Rhodovulum sp. P5
MTYVVTDNCIACKYTDCVEVCPVDCFYEGENMLVIHPDECIDCGVCEPECPADAIRPDTE
PDMDEWVEFNRKYAEQWPCIITRKDPLPEAEAKDGETGKREKYFSETPGEGG
>2[4Fe-4S]ST1(daa:AKL17_2861) Defluviimonas alba
MTYVVTDNCIACKYTDCVEVCPVDCFYEGVNTLVIHPDECIDCGVCEPECPADAIRPDTE
PDMDKWVEFNRKYSEQWPVITQKRDPLPEAAERDGESGKLDKYFIAEPGQGD
>2[4Fe-4S]ST1(yan:AYJ57_12605) Yangia sp. CCB-MM3
MTYVVTENCIACKYTDCVEVCPVDCFYEGENTLVIHPDECIDCGVCEPECPADAIRPDTE
PDMEKWVEFNRKYAELWPVIITKKDPLPEAEERDGEAGKMTKYFSEAPGEGD
>2[4Fe-4S]ST1(suam:BOO69_12095) Sulfitobacter sp. AM1-D1
MTYIVNDSCIACKYTDCVEVCPVDCFYEGENMLVIHPDECIDCGVCEPECPADAIRPDTE
PDMEKWVEFNRKYSELWPVIITKKDPLPEAEERDGESGKLEKYFSERPGEGG

>2[4Fe-4S]ST1(tpro:Ga0080559_TMP3271) Salipiger profundus
MTYVVTDNCIACKYTDCVEVCPVDCFYEGENALVIHPDECIDCGVCEPECPADAIRPDTE
PDMEKWVEFNRKYSEMWPVIITKKDPLPEAEERDGEPGKLDKYFSEAPGEGG
>2[4Fe-4S]ST1(paby:Ga0080574_TMP3586) Pelagibaca abyssi
MTYVVTENCIACKYTDCVEVCPVDCFYEGENTLVIHPDECIDCGVCEPECPADAIRPDTE
PDMEKWVEFNRKYSEMWPVIISKKDPMPDADERDGEEGKMEKYFSEAPGEGG
>2[4Fe-4S]ST1(rmm:ROSMUCSMR3_01126) Roseovarius mucosus
MTYIVNDACIACKYTDCVEVCPVDCFYEGENMLVIHPDECIDCGVCEPECPADAIRPDTE
PDMEKWVEFNRKYAEMWPVIITKKDPLPEAEERDGEAGKLEKYFSEAPGLGG
>2[4Fe-4S]ST1(sil:SPO1419)Ruegeria pomeroyi
MTYVVTENCIACKYTDCVEVCPVDCFYEGENALVIHPDECIDCGVCEPECPADAIRPDTE
PGMEQWVEFNRKYSEMWPVIVTKKDPLPEAEERDGESGKMEKYFSEAPGEGG
>2[4Fe-4S]ST1(sit:TM1040_0575)Ruegeria sp. TM1040
MTYVVTDNCIACKYTDCVEVCPVDCFYEGENTLVIHPDECIDCGVCEPECPADAIRPDTE
PDMDQWVEFNRKYAELWPVIVSKKDPMPGHEERDGEEGKLEKYFSEAPGEGG
>2[4Fe-4S]ST1(rde:RD1_1824)Roseobacter denitrificans
MTYIVNDSCIACKYTDCVEVCPVDCFYEGENMLVIHPDECIDCGVCEPECPADAIRPDTE
PDMEKWVEFNRKYSELWPVIITKKDPLPTAEERDGETGKLEKYFSENPGEGG
>2[4Fe-4S]ST1(rli:RLO149_c028960)Roseobacter litoralis
MTYIVNDSCISCKYTDCVEVCPVDCFYEGENMLVIHPDECIDCGVCEPECPADAIRPDTE
PDMEKWVEFNRKYSELWPVIITKKDPLPTAEERDGETGKLEKYFSENPGEGG
>2[4Fe-4S]ST1(dsh:Dshi_0785)Dinoroseobacter shibae
MTYVVTDNCIACKYTDCVEVCPVDCFYEGENMLVIHPDECIDCGVCEPECPADAIRPDTE
PDMEKWVEFNRKYSEAWPVIITKKDQLPDAEERDGEQGKLEKYFSEAPGEGG
>2[4Fe-4S]ST1(pga:PGA1_c07530)Phaeobacter inhibens DSM 17395
MTYVVTDNCIACKYTDCVEVCPVDCFYEGENTLVIHPDECIDCGVCEPECPADAIRPDTE

PDMDKWVEFNRKYSEMWPVIVSKKDPLPEAEERDGEEGKLEKYFSEAPGEGG

>2[4Fe-4S]ST1(pgl:PGA2_c07040)Phaeobacter inhibens 2.10

MTYVVTDNCIACKYTDCVEVCPVDCFYEGENTLVIHPDECIDCGVCEPECPADAIRPDTE

PDMDKWVEFNRKYSEMWPVIVSKKDPLPEAEERDGEEGKLEKYFSEAPGEGG

>2[4Fe-4S]ST1(pgd:Gal_02738)Phaeobacter gallaeciensis DSM 26640

MTYVVTDNCIACKYTDCVEVCPVDCFYEGENTLVIHPDECIDCGVCEPECPADAIRPDTE

PDMDKWVEFNRKYSEMWPVIVSKKDPLPEAEERDGEEGKLEKYFSEAPGEGG

>2[4Fe-4S]ST1(php:PhaeoP97_02438)Phaeobacter porticola

MTYVVTDNCIACKYTDCVEVCPVDCFYEGENTLVIHPDECIDCGVCEPECPADAIRPDTE

PDMDKWVEFNRKYSEMWPVIVSKKDPLPDAEERDGEEGKLEKYFSEAPGEGG

>2[4Fe-4S]ST1(lmd:METH_12915)Leisingera methylohalidivorans

MTYVVTENCIACKYTDCVEVCPVDCFYEGENTLVIHPDECIDCGVCEPECPADAIRPDTE

PDMDKWVEFNRKYSELWPVIVSKKDPMPGYEERDGEEGKMEKYFSEAPGEGG

>2[4Fe-4S]ST1(ptp:RCA23_c10050)Planktomarina temperata

MTYVVTDNCIACKYTDCVEVCPVDCFYEGENMLVIHPDECIDCGVCEPECPADAIRPDTE

PDMEKWVDFNRKYSEAWPVIITKKDELPEAEARDGEEGKLEKYFSEAAGTGG

>2[4Fe-4S]ST1(malg:MALG_01115) Marinovum algicola

MTYIVNDACIACKYTDCVEVCPVDCFYEGENMLVIHPDECIDCGVCEPECPADAIRPDTE

PDMEKWVEFNRKYSEMWPVIITKKDPLPKAEEHDGEEGKLEKYFSEAPGEGG

>2[4Fe-4S]ST1(con:TQ29_13275) Confluentimicrobium sp. EMB200-NS6

MTYVVVDNCIACKYTDCVEVCPVDCFYEGENMLVIHPDECIDCGVCEPECPADAIRPDTE

PDMEKWVEFNRKYSEAWPVIISKKDPLPEAEERDGETDKLNKYFSEAPGEGG

>2[4Fe-4S]ST1(mch:Mchl_4203)Methylorubrum extorquens CM4

MTYVVTDNCIRCKYTDCVEVCPVDCFYVGDTMLVINPDECIDCGVCEPECPADAIKADTE

PGLDGWIALNAKYAALWPNISEKRDPLHDAAAWDGRPGKLESVFGVSDPATA

>2[4Fe-4S]ST1(mrd:Mrad2831_6156)Methylobacterium radiotolerans

MTYVVTENCIRCKYTDCVEVCPVDCFYVGETMLVIDPDECIDCGVCEPECPADAIKADTE
PGLEGWKAFNAKYAALWPNIAEKVDPAADAAEWDGRDGKLIAVFGAADPAAA
>2[4Fe-4S]ST1(bid:Bind_3136)Beijerinckia indica
MSYVVLENCIKCKYMDCVEVCPVDCFYEGETMLVINPDECIDCGVCEPECPAEAIKPDTV
SGLEKWQALNRKMAQYWPNVTVKREAPPEAKQFDGRPGKFDAFFTETPGQGD
>2[4Fe-4S]ST1(mcg:GL4_1145)Methyloceanibacter caenitepidi
MTYVVTDNCIRCKYMDCVEVCPVDCFYEGENMLVIHPDECIDCGVCEPECPAEAIFPDTG
ANLESWLDLNAKYAELWPNIAVKREPPVDAAAFDGVPNKLEPFFSPNPGLGDHADHEAIE
LELRTNPNERRGHP
>2[4Fe-4S]ST1(ccr:CC_0654)Caulobacter vibrioides CB15
MTYIVTDACVRCKFMDCVEVCPVDCFYEGENFLVINPDECIDCGVCEPECPVDAIKPDTE
DEADGKWLKINADYAKVWPNITVKGEPPADREDFERETGKFEKYFSEKPGKGS
>2[4Fe-4S]ST1(ccs:CCNA_00691)Caulobacter vibrioides NA1000
MTYIVTDACVRCKFMDCVEVCPVDCFYEGENFLVINPDECIDCGVCEPECPVDAIKPDTE
DEADGKWLKINADYAKVWPNITVKGEPPADREDFERETGKFEKYFSEKPGKGS
>2[4Fe-4S]ST1(cak:Caul_4113)Caulobacter sp. K31
MTYIVTDACIKCKFMDCVEVCPVDCFYEGENFLAINPDECIDCGVCEPECPIDAIKPDTE
DEPDGKWLRINSEYAKIWPNITVKGVPPADREAFERETGKFEKYFSEKPGKGS
>2[4Fe-4S]ST1(cse:Cseg_3714)Caulobacter segnis
MTYIVTDACVRCKFMDCVEVCPVDCFYEGENFLVINPDECIDCGVCEPECPVDAIKPDTE
DEPDGKWLRVNADYAKVWPNITVKGVPPEDREQFERETGKFEKYFSEKPGKGS
>2[4Fe-4S]ST1(pzu:PHZ_c0708)Phenylobacterium zucineum
MTYIVMDPCIKCKFMDCVEVCPVDCFYEGENFLVINPDECIDCGVCEPECPVDAIKPDTE
DDPDGKWLKVNSEYSRVWPNITVKGTPPADAEQFERESGKFEKYFSEKPGRGS
>2[4Fe-4S]ST1(aex:Astex_1096)Asticcacaulis excentricus
MTYIVTDPCVKCKFMDCVEVCPVDCFYEGENFLVINPDECIDCGVCEPECPVDAIKPDTE

DEPDGKWLEVNSKYARVWPNISVKGTPPADREDFERETGKFEKYFSEKPGDGK

>2[4Fe-4S]ST1(mmr:Mmar10_2295) Maricaulis maris

MTYIVTDACVRCKYTDCVEVCPVDCFYEGENFLVIHPDECIDCGVCEPECPVEAIKPDTE

DDKDGKWLAINSKFAETWPNITLRKDAPADADAMADETGKYEKYFSEKPGSGD

>2[4Fe-4S]ST1(hne:HNE_3418) Hyphomonas neptunium

MTYIVVDACIRCKYMDCVEVCPVDCFYEGENMLVIHPDECIDCGVCEPECPVEAIKPDTE

DDPDGKWLKLNSDYAKVWPNITRMKEPPADREEFAQETGKLEKYFTANPGAGD

>2[4Fe-4S]ST1(hba:Hbal_0771) Hirschia baltica

MTYIVIDACIKCKYTDCVEVCPVDCFYEGENMLVIDPEECIDCGVCEPECPAEAIVPDTE

DDKDGKWLKLNTEYAAKWPNITVRKDPPEDADEWSQVKDKLGPHFSEKPGTGD

>2[4Fe-4S]ST1(hbc:AEM38_11180)Hyphomonadaceae bacterium UKL13-1

MTYIVTDACVKCKYMDCIEVCPVDCFYEGENFLVIHPDECIDCGVCEPECPVDAIKPDTE

DEPDGKWLKVNSDYAKIWPNITVKGEPPADRDDFAKEDGKFEKYFSPNPGEGN

>2[4Fe-4S]ST1(hdn:Hden_0076)Hyphomicrobium denitrificans ATCC 51888

MTYVVTENCIKCKYTDCVEVCPVDCFYEGENMLVIHPDECIDCGVCVPECPAEAIFSDAE

PQATAHWLDLNRKHADQWPNIVAKKAAMPDADAENGRAGKAAEFSPEAATEDAK

>2[4Fe-4S]ST1(hni:W911_04350)Hyphomicrobium nitrativorans

MTFVVTENCIKCKFTDCVEVCPVDCFYEGENMLVIHPDECIDCGVCVPECPADAIFSDAE

PQATAHWLEVNRKYADLWPNLTGKKPPLPNADAENGRQGKANELSPLPASEDVA

>2[4Fe-4S]ST1(ara:Arad_12331)Agrobacterium radiobacter

MTFVVTENCIKCKFQDCVEACPVNCFHEGPNFLVINPSECIDCGVCEPVCPAEAIYPLEE

LPVEQAAFAALNSELAAEWPVITIKGPPPADASIWDGKRGKLSLLER

>2[4Fe-4S]ST1(mln:A9174_34320)Mesorhizobium loti NZP2037

MTYVVTDNCIKCKYMDCVEVCPVDCFYEGENMLVIHPDECIDCGVCVPECPAEAIFPDTD

PSATPAWLDLNREYASSWPNITRKKIPPADANVWDGVPGKFDAHFSGKPGEGD

>2[4Fe-4S]ST1(mey:TM49_09940)Martelella endophytica

MTYVVTENCIACKHMDCVEVCPVDCFYEGENFLVIHPDECIDCGVCEPECPADAIVPDIG

DPNLGFWLEINRKYAELWPNIIVKGTPPDNAEAMQDVPNKYRDLFSEKPGDGS

>2[4Fe-4S]ST1(anh:A6F65_00405) Altererythrobacter namhicola

MTYVVTDACIKCKYTDCVEVCPVDCFYEGENMLVINPSECIDCGVCEPECPAEAILPDTE

DGQEKWLEINTKFSAEWPNITEKKDPPADADEHKGEEGKYEKFFSPEPGEGD

>2[4Fe-4S]ST1(cna:AB433_16445) Croceicoccus naphthovorans

MTYVVTDACIKCKYMDCVEVCPVDCFYEGENMLVINPSECIDCGVCEPECPAEAILPDTE

SDLEKWLELNTKYSAEWPNITVKKDSPADADEHKGEEDKFDKYFSAEPGEGD

>2[4Fe-4S]ST1(porl:BG023_11449) Porphyrobacter sp. LM 6

MTYVVTEDCIKCKYTDCVEVCPVDCFYEGENMLVINPSECIDCGVCEPECPAEAILPDTE

DGLEKWLELNTQYSAVWPNITSQKAPPEDADAHKGEKGKFEKYFSTEPGEGD

>2[4Fe-4S]ST1(rpb:RPB_3415)Rhodopseudomonas palustris HaA2

MTYVVTDNCKGCRYTECVTVCPVECFHVDAAMTYIDPENCIDCGGCAPACPVGAIAPDYR

LPAHQKFWIDVNRKRAAETPVLTARLPPLPGADDRRQALGR

>2[4Fe-4S]ST1(avi:Avi_7631) Agrobacterium vitis

MTYVVTDQCSGCRYTECVTVCPVECFHIDEEMTYIDPDNCIDCGGCAPVCPVGAIHASYLLPADKQEWIEINRRRAAETPVVASRLPPLPGAEERRQALTS

>2[4Fe-4S]ST1(tmo:TMO_3055) Tistrella mobilis
MAYVVVDNCIKCKYQDCVEVCPVDCFYEGENMLVIHPDECIDCGVCEPECPAEAIKPDSD
DGLEQWAELNRKYAELWPNISRKGDAPADADEWNGVPDKLANHFSDKPGQQG

Subtype 2
>2[4Fe-4S]ST2(mci:Mesci_5841)Mesorhizobium ciceri (biovar Biserrulae)

MAFKIIASQCTQCGACEFECPSGAIKFKGESYVVEPQKCTECQGEFDTQQCASVCPVPKT

CVPA

>2[4Fe-4S]ST2(mop:Mesop_6415)Mesorhizobium opportunistum
MAFKIIASQCTQCGACEFECPSGAIKFKGESYVVEPQKCTECQGEFDTQQCASVCPVPKT
CVPA
>2[4Fe-4S]ST2(mam:Mesau_05888)Mesorhizobium australicum
MAFKIIASQCTQCGACEFECPSGAIKFKGESYVVEPQKCTECQGEFDTQQCASVCPVPKT
CVPA
>2[4Fe-4S]ST2(mamo:A6B35_32325)Mesorhizobium amorphae
MAFKILASQCTQCGACEFECPSGAIKFKGEAYVIDPKECTECKEAFDTQQCASVCPVPKT
CVPA
>2[4Fe-4S]ST2(sme:SMa0811)Sinorhizobium meliloti 1021
MAFKIIASQCTQCGACEFECPRGAVNFKGEKYVIDPTKCNECKGGFDTQQCASVCPVSNT
CVPA
>2[4Fe-4S]ST2(smi:BN406_04228)Sinorhizobium meliloti Rm41
MAFKIIASQCTQCGACEFECPRGAVNFKGEKYVIDPTKCNECKGGFDTQQCASVCPVSNT
CVPA
>2[4Fe-4S]ST2(smeg:C770_GR4pC1047)Sinorhizobium meliloti GR4
MAFKIIASQCTQCGACEFECPRGAVNFKGEKYVIDPTKCNECKGGFDTQQCASVCPVSNT
CVPA
>2[4Fe-4S]ST2(smel:SM2011_a0811)Sinorhizobium meliloti 2011
MAFKIIASQCTQCGACEFECPRGAVNFKGEKYVIDPTKCNECKGGFDTQQCASVCPVSNT
CVPA
>2[4Fe-4S]ST2(smd:Smed_6234)Sinorhizobium medicae
MAFKIIASQCTQCGACEFECPRGAVNFKGEKYVIDPTKCNECKGGFDTQQCASVCPVSNT
CVPA
>2[4Fe-4S]ST2(same:SAMCFNEI73_pB0086)Sinorhizobium americanum
MAFRIIASQCTQCGACEFECPSGAIKFKGEAYVIDPNKCTECKGAFELQQCAYVCPVPKT

CVPVPA

>2[4Fe-4S]ST2(rel:REMIM1_PE00335)Rhizobium etli bv. mimosae Mim1

MAFRIIASQCTQCGACEFECPSGAIGFKGEAYVIDPEKCTECKGSFETQQCAEVCPVPKT

CVAAAAAI

>2[4Fe-4S]ST2(rep:IE4803_PB00377)Rhizobium etli bv. phaseoli IE4803

MAFRIIASQCTQCGACEFECPSGAIRFKGEIYVIDPEKCTECKGTFETQQCAEVCPVPKT

CVPAAPAI

>2[4Fe-4S]ST2(rei:IE4771 PB00178)Rhizobium sp. IE4771

MAFRIIASQCTQCGACEFECPSGAIGFKGEAYVIDPEKCTECKGAFETQQCAEVCPVPKT

CVAAAP

>2[4Fe-4S]ST2(rlt:Rleg2_5042)Rhizobium leguminosarum bv. trifolii WSM2304

MAFKIIASQCTQCGACEFECPSGAISFKIDKFVVDPNVCTECREEFDAPKCRAICPMQNT

CVPA

>2[4Fe-4S]ST2(rlg:Rleg_4923)Rhizobium leguminosarum bv. trifolii WSM1325

MAFKIIASQCTQCGACEFECPSGAISFKTDRFVVDPKICTECRIEFDAPKCRAICPMPNT

CVPA

>2[4Fe-4S]ST2(rhn:AMJ98_PC00281)Rhizobium sp. N1341

MAFRIIASQCTQCGACEFECPSGAIRFKGEIYVIDPEKCTECKGTFETQQCAEVCPVPKT

CVLAAPAI

>2[4Fe-4S]ST2(rpha:AMC79_PC00258)Rhizobium phaseoli

MAFRIIASQCTQCGACEFECPSGAIRFKGEIYVIDPEKCTECKGTFETQQCAEVCPVPKT

CVLAAPAI

>2[4Fe-4S]ST2(rhx:AMK02_PC00276)Rhizobium sp. N731

MAFRIIASQCTQCGACEFECPSGAIRFKGEIYVIDPEKCTECKGTFETQQCAEVCPVPKT

CVLAAPAI

>2[4Fe-4S]ST2(rsp:RSP_7561)Rhodobacter sphaeroides 2.4.1

MPYRIVTALCTACGACEIECPSSAIGPAGAAFRIDPLLCTECAGRFETPQCASVCPVPKT
CVPA
>2[4Fe-4S]ST2(rsk:RSKD131_1881)Rhodobacter sphaeroides KD131
MPYRIEPARCTACGACEIECPSSAIGPAGAAFRIDPLLCTECAGRFETPQCASVCPVPKT
CVPA
>2[4Fe-4S]ST2(rhc:RGUI_2354)Rhodovulum sp. P5
MAYKIVTETCTACGACEFECPNAAIRMVKDTFVVDPNLCSECEGHFENPQCAEVCPVPNT
CIPA
>2[4Fe-4S]ST2(mlo:msl8750)Mesorhizobium japonicum MAFF 303099
MAFKIIASQCTQCGACEFECPSGAIKFKGETYVIDPKKCTECEGTFETQQCASVCPVSKT
CVPA
>2[4Fe-4S]ST2(mln:A9174_326)Mesorhizobium loti NZP2037
MAFKIIASQCTQCGACEFECPSGAIKFKGETYVIDPKKCTECEGTFETQQCASVCPVSKT
CVPA
>2[4Fe-4S]ST2(rhi:NGR_a01280)Sinorhizobium fredii NGR234
MAFKIIASQCTQCGACEFECPSNAIELKGEKYVIDPKKCTECKGVFEIQQCASVCPMPKT
CVPA
>2[4Fe-4S]ST2(rtr:RTCIAT899_PB01415)Rhizobium tropici
MAFKIIASQCTQCGACEFECPSGAIELKGENYVIDPKKCTECEGAFETQQCASVCPVPKT
CVPA
>2[4Fe-4S]ST2(bja:bsr1739) Bradyrhizobium diazoefficiens USDA 110
MAYKIIASQCTVCGACEFECPNAAISLKNDIYVINPSQCTQCEGHSDAPKCAVVCPVPDT
CVPA
>2[4Fe-4S]ST2(bju:BJ6T_80810)Bradyrhizobium japonicum USDA 6
MAYKIIASQCTVCGACEFECPNAAISLKNDIYVINPSQCTQCEGHSDAPKCAVVCPVPDT
CVPA

>2[4Fe-4S]ST2(bjp:RN69_39250)Bradyrhizobium japonicum E109

MAYKIIASQCTVCGACEFECPNAAISLKNDIYVINPSQCTQCEGHSDAPKCAVVCPVPDT

CVPA

>2[4Fe-4S]ST2(bra:BRADO5448)Bradyrhizobium sp. ORS 278

MTYKIIASQCTVCGACEFECPNAAIRLKNDMYIIDPKKCTECEGHFDAPQCAAVCPVPDT

CVPA

>2[4Fe-4S]ST2(brs:S23_46530)Bradyrhizobium sp. S23321

MAYKIIASQCTVCGACEFECPNAAISLKRDMYVIDPKKCTECEGHFDTAQCAVVCPVPDT

CVPA

>2[4Fe-4S]ST2(aol:S58_22590)Bradyrhizobium oligotrophicum

MTSSDVAGIGELHMTYKIIASQCTVCGACEFECPNAAIRLKNDMYIIDPKKCTECEGHFD

APQCAAVCPVPDTCVPA

>2[4Fe-4S]ST2(bic:LMTR13_26885)Bradyrhizobium icense

MAYKIIASQCTVCGACEFECPNAAISLKNDIYVIDPKKCTECEGQCDTPQCAVICPVPGT

CVQA

>2[4Fe-4S]ST2(xau:Xaut_0083)Xanthobacter autotrophicus

MAYKIITSQCTVCGACEFECPNAAISLKNDTYVINPKKCTECEGSFETPQCAVVCPVENT

CVPA

>2[4Fe-4S]ST2(gdi:GDI0431)Gluconacetobacter diazotrophicus PA1 5 (Brazil)

MAYRIVTSQCTVCGACEFECPNGAISLKNDLYVINAKKCTECEGQFDTPQCVSVCPVPNT

CVPA

>2[4Fe-4S]ST2(sphi:TS85_14470)Sphingomonas sp. WHSC-8

MAYKIIVSQCTGCSACEFECPNAAISMKGDTFIIDPKKCTECEGIFDHAQCDTVCPVPKT

CVPA

>2[4Fe-4S]ST2(rsu:NHU_01471)Rhodovulum sulfidophilum

MALKIVKSQCTVCGACEFECPNAAIKFKGEGYIIDAEKCTECEGQFDTPQCAAVCPVPNT

CVPA

>2[4Fe-4S]ST2(con:TQ29_04770)Confluentimicrobium sp. EMB200-NS6

MSLKIIVSQCTVCGACEFECPNAAIKFKNDTYVIDPALCTECEGSYDSPQCAEVCPVPDT

CVPA

>2[4Fe-4S]ST2(mbry:B1812_08155)Methylocystis bryophila

MAYKIVASQCTVCGACEFECPNAAIRLKNDMYIIDPKKCTECEGHFDTPKCAEVCPVENT

CVPV

>2[4Fe-4S]ST2(msc:BN69_2612)Methylocystis sp. SC2

MAYKIIASQCTVCGACEFECPNAAIRLKNDMYIIDPKKCTECEGHFDTPQCAVVCPVENT

CVPA

>2[4Fe-4S]ST2(mno:Mnod_4006)Methylobacterium nodulans

MALKIIASQCTMCGACEFECPNNAIRAKGDIYIIDPKECTECEGQFDKPQCATVCPVPNT

CVKAAPVH

>2[4Fe-4S]ST2(met:M446_3601)Methylobacterium sp. 4-46

MALKIIASQCTVCGACEFECPNGAIRMKGDIYIVDPKKCTECDGQFDEPQCAAVCPVPNT

CVKAAPAA

>2[4Fe-4S]ST2(rpa:RPA4629)Rhodopseudomonas palustris CGA009

MAYKIVASQCTGCSACEPQCPNVAIFEKAGTFVIDPSKCSECIGHYDEPQCVAVCPVDGT

CVIDNSVPRYQAA

>2[4Fe-4S]ST2(azc:AZC_1043)Azorhizobium caulinodans

MAYKIIASQCTVCGACEFECPNAAIRLKNDMYIIDPKKCTECEGSFDSPQCDAVCPVPGT

CVPAG

>2[4Fe-4S]ST2(bbt:BBta_5903) Bradyrhizobium sp. BTAi1

MTLKIIASQCTSCSACEPECPNVAISEKNGTFVIDPKKCTECIGHFDEPQCAAVCPVDNT

CVIDNSFPRYQPPA

>2[4Fe-4S]ST2(bjp:RN69_39145) Bradyrhizobium japonicum E109

MPFKIIASQCTSCSACEPLCPNVAISEKGGNFVIEAAKCSECVGHFDEPQCAAACPVDNT
CVVDRALPRYQAPV
>2[4Fe-4S]ST2(bju:BJ6T_80600) Bradyrhizobium japonicum USDA 6
MCSAACPRVGLEIGSLRRKEEYVPFKIIASQCTSCSACEPLCPNVAISEKGGNFVIEAAK
CSECVGHFDEPQCAAACPVDNTCVVDRALPRYQAPV
>2[4Fe-4S]ST2(bra:BRADO5419) Bradyrhizobium sp. ORS 278
MTLKIIASQCTSCSACEPECPNVAISEKNGTFVIDPKKCTECIGHFDEPQCAAVCPVDNT
CVIDNSFPRYQPPA
>2[4Fe-4S]ST2(azc:AZC_3415)Azorhizobium caulinodans
MALKIVSSQCTSCSACEPECPNVAISEKNGTFVIDPKKCTECIGHYDVPQCAAVCPVDDT
CIIDTSYPRYQGAA
>2[4Fe-4S]ST2(xau:Xaut_0112)Xanthobacter autotrophicus
MTLKIVASQCTSCSACEPECPNVAISEKNGTFVIDPKKCTECIGHFDVPQCAAVCPVDDT
CVIDNAYPRYQPTA
>2[4Fe-4S]ST2(bja:bsr1760) Bradyrhizobium diazoefficiens USDA 110
MPFKIIASQCTSCSACEPLCPNVAISEKGGNFVIEAAKCSECVGHFDEPQCAAACPVDNT
CVVDRALPRYQAPV
>2[4Fe-4S]ST2(aol:S58_22870) Bradyrhizobium oligotrophicum
MTLKIIASQCTSCSACEPECPNVAISEKNGTFVIDPKKCTECLGHFDEPQCAAVCPVDNT
CVIDNSFPRYQPPA
>2[4Fe-4S]ST2(bic:LMTR13_26645)Bradyrhizobium icense
MPFKIIASQCTSCSACEQECPNVAISEKGGTFVIDPKKCTECIGHFEEPQCVAVCPVDNT
CVIDTSLPRY
>2[4Fe-4S]ST2(bid:Bind_0397)Beijerinckia indica
MSLKIAVSCVNCSACEAECPNEAISAGEGIYVIDPAKCTECIGFHDDPQCALVCPVEETC
VIDDNYPRYQAA

>2[4Fe-4S]ST2(msc:BN69_2618)Methylocystis sp. SC2
MTLKIIASQCTSCSACEPECPNVAITEKNGTFVIDPKKCTECIGHFDAPQCAAVCPVDNT
CVIDNAYPRYQAPA
>2[4Fe-4S]ST2(mbry:B1812_08145)Methylocystis bryophila
MTLKIIASQCTSCSACEPECPNVAITEKNGTFVIDPKKCTECIGHFDAPQCAAVCPVDDT
CVIDNAYPRYQPPA
>2[4Fe-4S]ST2(mey:TM49_15610)Martelella endophytica
MAMKILADMCTACGDCEPNCPTDAISPKKGVYAIDPDVCTECEGVSDTPKCMQACMDDCI
EPA
>2[4Fe-4S]ST2(rsp:RSP_3189)Rhodobacter sphaeroides 2.4.1
MSMTISADLCTACGDCEPVCPTHAIVPRKGIYFIKSEVCTECEGAFDMPQCMSVCTSDSI
APLEA
>2[4Fe-4S]ST2(rsh:Rsph17029_3928)Rhodobacter sphaeroides ATCC 17029
MSMTISADLCTACGDCEPVCPTHAIVPRKGIYFIKSEVCTECEGAFDMPQCMSVCTSDSI
APLEA
>2[4Fe-4S]ST2(rsq:Rsph17025_3189)Rhodobacter sphaeroides ATCC 17025
MSMSISADLCTACGDCEPVCPTNAIIPRKGIYFIKADVCTECDGEFDMPQCMNVCTADSI
VPLEA
>2[4Fe-4S]ST2(rcp:RCAP_rcc03284)Rhodobacter capsulatus
MAMKIDPELCTSCGDCEPVCPTNAIAPKKGVYVINADTCTECEGEHDLPQCVNACMTDNC
INPAA
>2[4Fe-4S]ST2(rpa:RPA4631)Rhodopseudomonas palustris CGA009
MAYKIVTSQCTVCGACEFECPNAAISMKRGTYVIDATKCTECEGQFDKPQCVSVCPVDNT
CVPA
>2[4Fe-4S]ST2(rsk:RSKD131_3365)Rhodobacter sphaeroides KD131
MSMTISADLCTACGDCEPVCPTHAIVPRKGIYFIKSEVCTECEGEFDMPQCMSVCTSDSI

APLEA

>2[4Fe-4S]ST2(rhc:RGUI_2053)Rhodovulum sp. P5

MAMMINADMCTACGDCESVCPTGAIIPSKGVFTIVADKCTECEPDHDMPQCLNSCMEDGC

IVPAE

>2[4Fe-4S]ST2(gdj:Gdia_1575) Gluconacetobacter diazotrophicus PA1 5 (JGI)

MAYRIVTSQCTVCGACEFECPNGAISLKNDLYVINAKKCTECEGQFDTPQCVSVCPVPNT

CVPA

>2[4Fe-4S]ST2(brs:S23_46240)Bradyrhizobium sp. S23321

MPFKIISSQCTSCSACEPECPNVAIREKGGTFVIDPKKCTECLGHFDEPQCVAVCPVDNT

CVIDTSLPRYQAPA

>2[4Fe-4S]ST2(rpc:RPC_4471)Rhodopseudomonas palustris BisB18

MAYKIISSQCTSCSACEPECPNVAIFEKGGTFHINPKKCTECLGHFDMAQCVAVCPVEGT

CVIDNSVPRYQAPA

>2[4Fe-4S]ST2(rpe:RPE_4540)Rhodopseudomonas palustris BisA53

MAYKIVASQCTGCTACEVECPNAAIFEKGGAFAIDPAKCSECIGHFDVAQCAAVCPVENT

CVIDKSVPRYVLPA

>2[4Fe-4S]ST2(bbt:BBta_5932)Bradyrhizobium sp. BTAi1

MAYKIIASQCTVCGACEFECPNAAIRLKNDMYIIDPKKCTECEGHFDAPQCAAVCPVPDT

CVPA

>2[4Fe-4S]ST2(rpb:RPB_0960)Rhodopseudomonas palustris HaA2

MAYKIVASQCTSCSACEPECPNEAISERRGVFMIDPSKCTECIGHFDVPQCVAVCPVDGT

CVIDNSVPRASAA

>2[4Fe-4S]ST2(rpt:Rpal_5110)Rhodopseudomonas palustris TIE-1

MAYKIVASQCTGCSACEPQCPNVAIFEKAGTFVIDPSKCSECIGHYDEPQCVAVCPVDGT

CVIDNSVPRYQAA

>2[4Fe-4S]ST2(rpt:Rpal_5112)Rhodopseudomonas palustris TIE-1

MAYKIVTSQCTVCGACEFECPNAAISMKRGTYVIDATKCTECEGQFDKPQCVSVCPVDNT
CVPA
>2[4Fe-4S]ST2(rpx:Rpdx1_4807)Rhodopseudomonas palustris DX-1
MAYKIVASQCTGCSACEPQCPNVAIFEKAGTFAIDPSKCSECTGHYDEPQCVAVCPVDGT
CVIDNSVPRYQAA
>2[4Fe-4S]ST2(yan:AYJ57_00320)Yangia sp. CCB-MM3
MALTIVQSNCTVCGACEFECPNAAIRFEDDTYIIDADLCTECKGHYDIPQCAEICPVPET
CVPA
>2[4Fe-4S]ST2(rpd:RPD_1064)Rhodopseudomonas palustris BisB5
MAYKIVASQCTSCSACEPECPNVAITEKRGVFTIDPSTCTECLGHFDVPQCVAVCPVDGT
CVIDNSVPRARAA
>2[4Fe-4S]ST2(smk:Sinme_6360)Sinorhizobium meliloti AK83
MAFKIIASQCTQCGACEFECPRGAVNFKGEKYVIDPTKCNECKGGFDTQQCASVCPVSNT
CVPA
>2[4Fe-4S]ST2(smq:SinmeB_6022)Sinorhizobium meliloti BL225C
MAFKIIASQCTQCGACEFECPRGAVNFKGEKYVIDPTKCNECKGGFDTQQCASVCPVSNT
CVPA
>2[4Fe-4S]ST2(smx:SM11_pC1133)Sinorhizobium meliloti SM11
MAFKIIASQCTQCGACEFECPRGAVNFKGEKYVIDPTKCNECKGGFDTQQCASVCPVSNT
CVPA
>2[4Fe-4S]ST2(smer:DU99_23505)Sinorhizobium meliloti RMO17
MAFKIIASQCTQCGACEFECPRGAVNFKGEKYVIDPTKCNECKGGFDTQQCASVCPVSNT
CVPA
>2[4Fe-4S]ST2(ret:RHE_PD00230)Rhizobium etli CFN 42
MAFRIIASQCTQCGACEFECPSGAIRFKGEIYVIDPEKCTECKGTFETQQCAEVCPVPKT
CVPAAPAI

>2[4Fe-4S]ST2(rec:RHECIAT_PB0000274)Rhizobium etli CIAT 652
MAFRIIASQCTQCGACEFECPSGAIRFKGEIYVIDPEKCTECKGTFETQQCAEVCPVPKT
CVLAAPAI
>2[4Fe-4S]ST2(rir:BN877_p0303)Rhizobium sp. IRBG74
MVFKIIASQCTQCGACEFECPSGAIEFKGEKYVIDPDKCTECEGVFEVQQCASVCPTPKT
CVPA
>2[4Fe-4S]ST2(rga:RGR602_PB00242)Rhizobium gallicum
MAFRIIASQCTQCGACEFECPSDAIDFKGETYVIDPDKCTECKKTFETQQCAEVCPVPKT
CVPAAPSI
>2[4Fe-4S]ST2(rpb:RPB_0958)Rhodopseudomonas palustris HaA2
MAYKIVTSQCTVCGACEFECPNAAISMKRGTYVIDAVKCTECEGHFDKPQCVAVCPVDNT
CVPA
>2[4Fe-4S]ST2(rpc:RPC_4473)Rhodopseudomonas palustris BisB18
MAYKIVASQCSVCGACEFECPNAAIVLKRDMYIIEAAKCTECEGQFDKPQCASVCPVENT
CVPA
>2[4Fe-4S]ST2(rpd:RPD_1062)Rhodopseudomonas palustris BisB5
MAYKIITSQCTVCGACEFECPNAAIAMKRGTYVIDAVKCTECEGHFDKPQCVAVCPVDNT
CVPA
>2[4Fe-4S]ST2(rpx:Rpdx1_4809)Rhodopseudomonas palustris DX-1
MAYKIVTSQCTVCGACEFECPNAAISMKRGTYVIDATKCTECEGQFDKPQCVSVCPVDNT
CVPA
>2[4Fe-4S]ST2(rpe:RPE_4542)Rhodopseudomonas palustris BisA53
MAYKIVASQCSVCGACEFECPNAAISLKREMYVIDASKCTECEGQFDKPQCAVVCPVEKT
CIPA
>2[4Fe-4S]ST2(sphi:TS85_14445)Sphingomonas sp. WHSC-8
MAYKIIGSQCTNCSGCEAECPNLAISERGGTFVIDPALCTECIGHFDTPQCVAICPVDNTCVIDTSVPRYHES

>2[4Fe-4S]ST2(msl:Msil_3645)Methylocella silvestris

MTYKIVASQCTSCSACEAECPNVAISEKGGTFVINPKKCTECIGYFDIPQCVAVCPVDNTCVIDNSLPRYQGNAA

>2[4Fe-4S]ST2(mey:TM49_15560)Martelella endophytica

MTYSITEHCVSCFACVDVCPSGAIQETSPVFTIAEKKCTHCEGAHAVSQCASICPVEGAILDPLGEMANPPGSLTGIPPEKRLAAGLLV

Subtype 3

>2[4Fe-4S]ST3(syb:TZ53_09145) Sphingobium sp. YBL2

MIAHIFEDRCTACQACVSACPTHVLDAGPDGAPRIARLDQCQTCFLCELHCEADAIYVGP

DQRQAEAISPEAILASGHLGRLRHDHGWDREPEPGSTGHLHDFWQLGPLLREGAEIAARR

YAERHASDRA

>2[4Fe-4S]ST3(shz:shn_23395) Shinella sp. HZN7

MIEVISEDRCTGCNICVNVCPTNVFAPVADGIPVIARQDDCQTCFMCEVYCPDDALYVAP

HATEITHVTVAELEARDLFGSYRKSIGWIRGGRPRPAPEIMQRLEHR

>2[4Fe-4S]ST3(bra:BRADO4282) Bradyrhizobium sp. ORS 278

MIELILADRCTDCGACVDVCPTNVLDRAVSGPPLLARVEDCQTCFMCELYCRADAIYVAP

DCDRRVATTPDEALASGRLGQYRKHSGWDEWAGQFPNEQWLMETVFRRAGEAVRAAETDDKRTA

>2[4Fe-4S]ST3(sme:SMc02347)Sinorhizobium meliloti 1021

MIEVVSTDRCIRCDICERICPAFVFDRDETGLPVIARQDDCQTCFLCEIYCPADALYVAE

QAHGPIGITEAEVLERDLFGLYARSLGWSRGRAGGAQNDPTRHIRVAQV

>2[4Fe-4S]ST3(smk:Sinme_2626)Sinorhizobium meliloti AK83

MIEVVSTDRCIRCDICERICPAFVFDRDETGLPVIARQDDCQTCFLCEIYCPADALYVAE

QAHGPIGITEAEVLERDLFGLYARSLGWSRGRAGGAQNDPTRHIRVAQV

>2[4Fe-4S]ST3(smq:SinmeB_2405) Sinorhizobium meliloti BL225C

MIEVVSTDRCIRCDICERICPAFVFDRDETGLPVIARQDDCQTCFLCEIYCPADALYVAE

QAHGPIGITEAEVLERDLFGLYARSLGWSRGRAGGAQNDPTRHIRVAQV

>2[4Fe-4S]ST3(smx:SM11_chr2729)Sinorhizobium meliloti SM11

MIEVVSTDRCIRCDICERICPAFVFDRDETGLPVIARQDDCQTCFLCEIYCPADALYVAE

QAHGPIGITEAEVLERDLFGLYARSLGWSRGRAGGAQNDPTRHIRVAQV

>2[4Fe-4S]ST3(mam:Mesau_03271)Mesorhizobium australicum

MIEIVSATRCIECDICVKVCPTNVFDATEHGAPVIARQDDCQTCFLCEIYCPTDALYVAE

AAEGPTGITEAEVEARLLFGSYARALGWTRGKAGGSEFDPTHRIRVAQ

>2[4Fe-4S]ST3(smeg:C770_GR4Chr2676)Sinorhizobium meliloti GR4

MIEVVSTDRCIRCDICERICPAFVFDRDETGLPVIARQDDCQTCFLCEIYCPADALYVAE

QAHGPIGITEAEVLERDLFGLYARSLGWSRGRAGGAQNDPTRHIRVAQV

>2[4Fe-4S]ST3((smel:SM2011_c02347)Sinorhizobium meliloti 2011

MIEVVSTDRCIRCDICERICPAFVFDRDETGLPVIARQDDCQTCFLCEIYCPADALYVAE

QAHGPIGITEAEVLERDLFGLYARSLGWSRGRAGGAQNDPTRHIRVAQV

>2[4Fe-4S]ST3(mci:Mesci_3004)Mesorhizobium ciceri (biovar Biserrulae)

MIEIVSATRCVECDICVKVCPANVFDATESGVPVIARQEDCQTCFLCEIYCPTDALYVAE

FAEGPTGITEAQADARQLFGSYARALGWKRGKAGGSEFDPTHRIRVAQ

>2[4Fe-4S]ST3((abs:AZOBR_180293)Azospirillum brasilense Sp245

MIELILSERCIACGTCAKVCPDDVLRTDAAGEPVIAHKEDCQTCYLCELFCPVDALYVSP

RTAPDPTVDAAALIAAGLIGSYRRELGWTRGRPGGTETDLSYRMHEAFAEPFGEIWKRSGI

>2[4Fe-4S]ST3(six:BSY16_5978)Sinorhizobium sp. RAC02

MIEVLDADTCTDCNLCVQVCPTNVFEAVPDGPPLILRQDSCQTCFMCELYCPVDALYVDP

DAEAVHKLTVEGVRAGNLFGSYRKAIGWHKETRDRRYIDDHFRLPQ

>2[4Fe-4S]ST3(bid:Bind_0547)Beijerinckia indica

MIEIIDPTRCTSCNICVKVCPTNVFGSVKNSIPVIARQSDCQTCFMCELYCPADALYVAP

FTDELQIDDIHASHNNIDFGSYRRAIGWTKETNALRASDQSFELLKP

>2[4Fe-4S]ST3(cid:P73_2118)Celeribacter indicus

MIEVVSASRCIRCDICVRVCPADVFGKDGEGLPVILRQDDCQTCFLCEIYCPTDALFVAE
VADEVTGITESVVEASGLFGAYARSLGWAKGRAGGSQFDTTQHLRAAQT
>2[4Fe-4S]ST3(pami:JCM7686_pAMI6p032)Paracoccus aminophilus
MIEVISAARCTGCNICVQVCPTNVFAAVKGGIPVIARQDDCQTCYMCEVWCPDDALYVAP
NADGTEGITEPELEAQGRFGSYRRSVGWAKGGRVAPAPQIMSRLRG
>2[4Fe-4S]ST3(pami:JCM7686_pAMI5p125)Paracoccus aminophilus
MIELVSATRCVTCDVCIKACPMNVFDRAEDGLPVIARQEDCQTCFLCEAHCPTDALFVAA
ERAPLPPARLPDEAALIASGIMGSYRARLGWGKGRKPPRDFAAALTLSQAQAAAPGRVPP
GQPRAAQFGRVDANGSELQP
>2[4Fe-4S]ST3(cid:P73_2383)Celeribacter indicus
MIELVSASRCVTCDLCIAVCPMNVFDRAEDGLPVIARQEDCQTCYLCEAHCPTDALYVAA
ERTPLSDRLRPREAELAGTDLMGSYRARLGWGRGLRPPGTLNAAVELSHAPAAAAGVAPP
RQPAPAAFHEECPDV

>2[4Fe-4S]ST3(same:SAMCFNEI73_pC1420) Sinorhizobium Americanum

MIEVIDTETCTDCNLCVKVCPTNVFDAIADGPPVIARQGDCQTCFMCEVYCPVDSLYVDP
DPANSYGLTVENVKQANLFGSYRRSIGWAKETRHLRRVDDHFRLPQ
>2[4Fe-4S]ST3(eah:FA04_28520) Ensifer adhaerens Casida A

MIEVLDTNTCTDCNLCVQVCPTNVFQAQREGPPLIKRQDACQTCFMCELYCPVDALYVEP
DAEAVHGLTVEGVHERQLFGSYRKAIGWHKDTRHRRLIDDHFRLPQ
>2[4Fe-4S]ST3(rel:REMIM1_PF00314) Rhizobium etli bv. mimosae Mim1

MIEVLDPQTCTDCNLCVKVCPTNVFEAVGDGPPVILRQENCQTCFMCELYCPVDALYVDP
DPEATHGLILETVRESGLFGSYRKAIGWHKDTRDRRHIDDHFRLPQ
>2[4Fe-4S]ST3(rei:IE4771_PE00132) Rhizobium sp. IE4771

MIEVLDPQTCTDCNLCVKVCPTNVFEAVLDGPPVILRQNNCQTCFMCELYCPVDALYVDP
DPEITHDLTLETVRESSLFGSYRKAIGWHKDTRDRRHIDDHFRLPQ
>2[4Fe-4S]ST3(bbt:BBta_3288) Bradyrhizobium sp. BTAi1

MIELIVADRCTDCGACVEVCPTNVLERAPSGPPQLARVEDCQTCFMCELYCRADAIYVAP
DCDRRVAITQETALDSGRLGQYRKHSGWDEWAGQFPNEQWLMETVFRRAGEAAAGAAGAD
DKRTA
>2[4Fe-4S]ST3(cse:Cseg_2020) Caulobacter segnis

MIEVVVEDRCTGCGDCVQACPSDVLRVGEGGKAVIAHQVDCQTCLLCELYCPADALFVWP
EVDRVIGVGADQALASGQLGAFRRDHGWGEHAATHTNQHWRMGAIFERARLLAIETAAAK
TARTEDVR

>2[4Fe-4S]ST3(bbt:BBta_4871) Bradyrhizobium sp. BTAi1

MIEIIDPERCTACNICVTACPTNVFDKTEGLPVIARQADCQTCFLCELYCPEDALFVSPF
AEQPQGIDVVAARQSGLLGSYRRAVGWTKDSEHLREADRSFRLFGQ

>2[4Fe-4S]ST3(eah:FA04_29555) Ensifer adhaerens Casida A

MIEVISVDRCIRCDICERICPAFVFDRDETGLPIIARQDDCQTCFLCEIYCPTDALYVAE
AAHGPTGIAEAEVLERDLFGAYARSLGWNRGRAGGAQDDPTRHIRVAQV

>2[4Fe-4S]ST3(smi:BN406_02424) Sinorhizobium meliloti Rm41

MIEVVSTDRCIRCDICERICPAFVFDRDETGLPVIARQDDCQTCFLCEIYCPADALYVAE
QAHGPIGITEAEVLERDLFGVYARSLGWSRGRAGGAQNDPTRHIRVAQV

>2[4Fe-4S]ST3(smer:DU99_14280) Sinorhizobium meliloti RMO17

MIEVVSTDRCIRCDICERICPAFVFDRDETGLPVIARQDDCQTCFLCEIYCPADALYVAE
QAHGPIGITEAEVLERDLFGLYARSLGWSRGRAGGAQNDPTRHIRVAQV

>2[4Fe-4S]ST3(six:BSY16_4036) Sinorhizobium sp. RAC02

MIEVISEDRCTGCNICVNVCPTNVFDPVPGGNPTIARQDDCQTCFMCEVYCPEDALYVAP
LTTAVTGVTSVELEAKDIFGSYRKSIGWIRGGRPRPTPEIMKRLDHH

>2[4Fe-4S]ST3(six:BSY16_5015) Sinorhizobium sp. RAC02

MIEVISTDRCIRCDICERICPAFVFDRDETGLPVIARQDDCQTCFLCEIYCPTDALYVAE
NAHGPIGISEADVLERDLFGTYARSLGWNRGRAGGAQNDPTRHIRVAQV
>2[4Fe-4S]ST3(npp:PP1Y_AT22995) Novosphingobium sp. PP1Y

MLIAAGLYPRNTPEPGRLRSSEAQRPLAARTTFDEVVQGLSEPEARFEAGRCLSCGNCFECDNCFAACPEQAILRLGRGHGYRVEPDLCTGCGICFEQCPCHAIEMQPEPVPTDAP

LGPT

DEPLAPHRFRLRV

>2[4Fe-4S]ST3(psf:PSE_2104) Pseudovibrio sp. FO-BEG1

MANLSRRAFFTRLSAKPEVANPFRPPWTSEQRIEDK**C**TGCFE**C**VKACPEGILFSDDNRPFLKPGVGE**C**TF**C**EE**C**AKA**CP**EDGLFNLSEAPWRLTADLKQEACLLQKGVSCRTCTDCCDPR

ALRFDLRTPPAGQIQLDADQCSGCGACLVACPVDAISLNDQSNTSQESLANA

Subtype 4

>2[4Fe-4S]ST4(abs:AZOBR_110086)Azospirillum brasilense Sp245

MAISRADLFRGRFRSEARPSQPPAAPSQALEARIGAGCLSYTGIDCRMCGDHCDHGAIRF

RPLGRGRWLPIIEEGGCTGCGDCAGVCPVKAVTMEAVAA

>2[4Fe-4S]ST4(mgy:MGMSRv2__2006)Magnetospirillum gryphiswaldense MSR-1 v2

MMSHSLSRRALFGRTPPQSPPEAPVKRVAVLGGTCIATQGVACGTCADPCDPRAIKIRPM

LGGRAIPLIDAAACTGCGDCLNVCPVGALVLAPAGSGESKPCA

>2[4Fe-4S]ST4(abq:ABAZ39_06965)Azospirillum brasilense Az39

MAISRADLFRGRLRSEPVPSEAPQAMVARIGAACLSYTGTDCRMCGDHCDHAAIRFRPLG

RGRWLPIIEEGGCTGCGDCATVCPVKAVTMEVATA

>2[4Fe-4S]ST4(same:SAMCFNEI73_Ch1327)Sinorhizobium Americanum

MIGERCLARNGTDCQACRDACPTEAIRFRPRVGGPFLPELEQEACTGCGACLAVCPVAAI

GVREVRGESVYV

>2[4Fe-4S]ST4(rlt:Rleg2_2940)Rhizobium leguminosarum bv. trifolii WSM2304

MQTKALSRRQLLLGHPDPASEPDASTSLPHTAMVTEACLSRRGVACRSCDESCPENAIRF

RPRIGGPFLPEIDETLCTGCDACLAICPVGAIAIATRINGLEGRNA

Subtype 5

>2[4Fe-4S]ST5(rpa:RPA4612)Rhodopseudomonas palustris CGA009

MAIFSTRDGKPWEPTYLTAIDDKKCIGCGRCHKVCSRDVMHLMGVNEDGELVACSLDDDD

DDEEFVRKVMVLDAAGNCIGCSACVRVCPKACQTHVKASELGL

>2[4Fe-4S]ST5(rpt:Rpal_5093)Rhodopseudomonas palustris TIE-1

MAIFSTRDGKPWEPTYLTAIDDKKCIGCGRCHKVCSRDVMHLMGVNEDGELVACSLDDDD

DDEEFVRKVMVLDAAGNCIGCSACVRVCPKACQTHVKASELGL

>2[4Fe-4S]ST5(rpx:Rpdx1_4790)Rhodopseudomonas palustris DX-1

MAIFSTRDGQPWEPTYLTAIDDKKCIGCGRCHKVCSRDVMHLMGVNEDGELVGCSLDDDD

DDEEFVRKVMVLDSAGNCIGCSACVRVCPKACQTHVKASELGL

Subtype 6

>2[4Fe-4S]ST6(ngl:RG1141_PB00800)Neorhizobium galegae bv. officinalis bv. officinalis HAMBI 1141

MAFKIVTSQCSQCGACEFVCPSGAVALKGEHYVIDPRKCTECEGFGTQQCAAACPMEKTC

VPA

>2[4Fe-4S]ST6(ngg:RG540_PA10570) Neorhizobium galegae bv. orientalis HAMBI 540

MAFKIVTSQCSQCGACEFVCPSGAVALKGENYVIDPRKCTECDEFGTQQCAAACPMKETC

VPA

Subtype 7

>2[4Fe-4S]ST7(mbry:B1812_06580)Methylocystis bryophila

MAYRIIASQCTACGACEFECPNGAIRMKGETYIIDAGKCTECDGDGPRCVEVCPVENTCV

RA

>2[4Fe-4S]ST7(mgy:MGMSRv2__0355) Magnetospirillum gryphiswaldense MSR-1 v2

MALKITTSTCSACGACEFECPNAAIAMKGETYVIKASACSECDGDDPKCIAVCPVENCIV

KA

Subtype 8

>2[4Fe-4S]ST8(abq:ABAZ39_29555)Azospirillum brasilense Az39

MIELISPTRCTGCNICVSICPMDVFQTASGQAKGAAPVIARQSDCQTCFLCEAHCPDDAL

YVAPDAETVTGIAEEAVAAAGLFGSYRRAIGWSNGHRDNRSADQTFRLLAPH

>2[4Fe-4S]ST8(aak:AA2016_5898)Aminobacter aminovorans

MRQVTRGTLVIDADRCKGCELCIVACPPRVLTMSRTGNATGYLYPELAPGCTGCTACQQVCPDFVFEVYKYHEPIVSGSDAERMQ

Subtype 9

>2[4Fe-4S]ST9(rpd:RPD_1511)Rhodopseudomonas palustris BisB5

MSSHRAYPMFDPGQAGVPDDLCPIATDINPMLPGDWRSMRPVVDREKCVKCAVCWLYCPV

QCMVERPAWFDVDLRTCKGCGICANECPHRAIAMVEETAG

Subtype 10

>2[4Fe-4S]ST10(magq:MGMAQ_3495)Magnetospira sp. QH-2

MPTFVYMTRCDGCGHCVDICPSDIMHIDKTYRRAYNIEPNMCWECYSCVKACPQNAIDVRGYADFAPLGHSVRAVRDEEKGVIAWRIKFRNGKKDLNLLAPITTKPWGTCIPQLKN
APEPSKEQRDSQLLYNEPKYIRMDDGDLHTLESNGLVMKEGVYY
>2[4Fe-4S]ST10(fil:BN1229_v1_3251)Candidatus Filomicrobium marinum W

MPTFVYMTRCDGCGHCVDICPSDIMHIDPTYRRAYNIEPNMCWECYSCVKACPQNAIDVR
GYADFAPLGHSVRVQRDEERGTIAWKIKYRNGTEKEFLSPITTKPWGKHIPKLADVPAPS
RDMRDSELLYNEPKWIRMDDDGLRTLKDAGLVLKKGVYY

>2[4Fe-4S]ST10(fiy:BN1229_v1_2672)Candidatus Filomicrobium marinum Y

MPTFVYMTRCDGCGHCVDICPSDIMHIDPTYRRAYNIEPNMCWECYSCVKACPQNAIDVR
GYADFAPLGHSVRVQRDEERGTIAWKIKYRNGTEKEFLSPITTKPWGKHIPKLADVPAPS
RDMRDSELLYNEPKWIRMDDDGLRTLKDAGLVLKKGVYY
>2[4Fe-4S]ST10(aace:A0U92_03265)Acetobacter aceti

MITEILSDRCNSCNACVVVCPDHVLDMAKDGQLPVIARPDQCQTCFLCELYCSEDAIYVAVGVPRSELKNDPLGNIRRDYGWDGHEEDPLKNFWQLGGLLREGVMISSARYALRPS
GDKDTEKKI
>2[4Fe-4S]ST10(bid:Bind_3084)Beijerinckia indica

MIVHIFEDLCTGCNACVAACPTHVLDPAEAGRVPLIARQDQCQTCFMCELYCEKDAIYVGPIQQGPEPVDPELIRKSGLLGQLRRDYEWDAGPDDPAPLREFWRLGPLLMEGAQTA
EKRYEERHPESAALPPR

>2[4Fe-4S]ST10(otm:OSB_13110)Octadecabacter temperatus

MSKKLPPKPDTQKPAASVVNIDYDLCINCAICIRSCPVDVLRINRETRWLEATYWEDCMLCKLCELDCPEPGAITISADKPLGFAMSFG

Subtype 11
>2[4Fe-4S]ST11(rmm:ROSMUCSMR3_02763) Roseovarius mucosus

MWYELRDRSLPSSEGHAVLPRPPGTVREWRLADMCTGCGDCVAVCPKAIVALDKEVLPVVTAIDACGRCGLCADVCTRGAIELTKETRLGLERILKGDGLGQRAG

Subtype 12

>2[4Fe-4S]ST12(rpa:RPA4257) Rhodopseudomonas palustris CGA009

MIGFGWLEALLRVGRKLFVKPETQLYPEQKPKLYPRARGRIVLTRDPDGQERCVACNLCA
TVCPVGCIDLAKAVADDGRWYPEYFRVNFARCIFCGFCEDACPTAAIQLTPDYELSEWRR
DALVYEKHDLLISGEGKVRGYRYWSVAGKAIPGKDKGEAEHEAPPVNLKGLLP
>2[4Fe-4S]ST12(rpb:RPB_1354) Rhodopseudomonas palustris HaA2

MIGWLEAMLRVGRKLFVKAETQLYPEEKPKLFPRSRGRIVLTRDPDGQERCVACNLCAAV
CPVGCIDLSKAVADDGRWYPEHFRINFARCIFCGFCEEACPTAAIQLTPDFELGEWRRDA
LVYEKHDLLIAGEGKVRGYRYWSVAGKAIDGKDKGCAENESPPVDLKGLLP
>2[4Fe-4S]ST12(rpd:RPD_1333) Rhodopseudomonas palustris BisB5

MIGWLEAMLRVGRKLFVKAETQLYPEEKPKLYPRSRGRIVLTRDPDGQERCVACNLCAVV
CPVGCIDLTKAVADDGRWYPEHFRINFARCIFCGFCEEACPTSAIQLTPDFELGEWRRDA
LVYEKHDLLISGEGKVRGYRYWSVAGKAIDGKDKGEAEHESPPVDLRGLLP
>2[4Fe-4S]ST12(rpe:RPE_1717) Rhodopseudomonas palustris BisA53

MKGFGWLEGLLRVGRKLFVKAETVQYPEVRPYLPPRSRGRIVLTRDPDGQERCVACNLCA

VACPVGCIDLAKAVAEDGRWYPEHFRINFARCIFCGYCEEACPTAAIQLTPDFELSEWRR
DALVYDKHDLLIAGEGKVRGYRYWSVAGKAIQGKDKGEADHEAPPVDLKGLLP

>2[4Fe-4S]ST12(rpt:Rpal_4736) Rhodopseudomonas palustris TIE-1

MIGFGWLEALLRVGRKLFVKPETQLYPEQKPKLYPRARGRIVLTRDPDGQERCVACNLCA
TVCPVGCIDLAKAVADDGRWYPEHFRVNFARCIFCGFCEDACPTAAIQLTPDYELSEWRR
DALVYEKHDLLISGEGKVRGYRYWSVAGKAIPGKDKGEAEHEAPPVNLKGLLP

>2[4Fe-4S]ST12(rpx:Rpdx1_4476) Rhodopseudomonas palustris DX-1

MIGFGWLEALLRVGRKLFVKPETQLYPEEKPKLYPRARGRIVLTRDPDGQERCVACNLCA
TVCPVGCIDLSKAVADDGRWYPEHFRVNFARCIFCGFCEDACPTAAIQLTPDYELSEWRR
DALVYEKHDLLISGEGKVRGYRYWSVAGKAIPGKDKGEAEHEAPPVNLKGLLP

>2[4Fe-4S]ST12(rpc:RPC_4064) Rhodopseudomonas palustris BisB18

MIGWLEALLRVGRKLAAKTVTVQYPEVKPQLPPRSRGRIVLTRDPDGQERCVACNLCAVA
CPVGCIDLSKAVAEDGRWYPEHFRINFARCIFCGYCEEACPTAAIQLTPDFELSEWRRDA
LVYDKKDLLIAGEGKVRGYRYWSVAGKAITGKDKGDAEHEAPPVDLKGLRP

>2[4Fe-4S]ST12(rsp:RSP_0107) Rhodobacter sphaeroides 2.4.1

MKGILDSILRVGRMIFAPTKTVQYPEEKLPLAPRTRGRIVLTRDPDGQERCVACNLCAAA
CPVDCIDVVKAETPDGRWYPESFRINFARCIFCGYCEEACPTSAIQLTPDVELADYRRGF
LQYEKEDLLISGEGKHPGYRYWDVAGKAIAGKAQDPVDPKDLCP

>2[4Fe-4S]ST12(rsh:Rsph17029_1743) Rhodobacter sphaeroides ATCC 17029

MKGILDSILRVGRMIFAPTKTVQYPEEKLPLAPRTRGRIVLTRDPDGQERCVACNLCAAA
CPVDCIDVVKAETPDGRWYPESFRINFARCIFCGYCEEACPTSAIQLTPDVELADYRRGF
LQYEKEDLLISGEGKHPGYRYWDVAGKTIAGKAQDPVDPKDLCP

>2[4Fe-4S]ST12(rsk:RSKD131_1424) Rhodobacter sphaeroides KD131

MKGILDSILRVGRMIFAPTKTVQYPEEKLPLAPRTRGRIVLTRDPDGQERCVACNLCAAA
CPVDCIDVVKAETPDGRWYPESFRINFARCIFCGYCEEACPTSAIQLTPDVELADYRRGF
LQYEKEDLLISGEGKHPGYRYWDVAGKAIAGKAQDPVDPKDLCP

>2[4Fe-4S]ST12(rsq:Rsph17025_1696) Rhodobacter sphaeroides ATCC 17025

MKGILDSILRVGRMVFAPTKTVEYPEVKLPLAPRTRGRIVLTRDPDGQERCVACNLCAAA
CPVDCIDVVKAETPDGRWYPESFRINFARCIFCGYCEEACPTSAIQLTPDVELADYRRGS
LQYEKEDLLISGEGKHPGYRYWDVAGKAIAGKQSDPVDPKDLCP

>2[4Fe-4S]ST12(rga:RGR602_PC00808) Rhizobium gallicum

MSRAQDRIGTWIGWAFFADLAKALALTFGYMFARSVTMQYPDKEKWLPYPRYRGHHFLTRDEEGEIKCVACELCARICPCDCIEVIPYEDEKGKRHPAKFEIDTARCLFCGLCEDA
CPAD
AIALGQQYEFSSFSSRDLVIGRDDLLAKPGKAATGGGVIAARLNTEKDVIVETKETQGYNWWRNIRRT

>2[4Fe-4S]ST12(rpha:AMC79_CH03559) Rhizobium phaseoli

MSRAQDRIGTWIGWTFFADLANALTLTLGYMFARPVTMQYPDREKWLPYSRYRGHHFLKR
DEEGEIKCVACELCARICPCDCIEVVPYEDEKGNRHPAKFEIDTARCLFCGLCEDACPAD
AIALGQQYEFSSFSSADLVIGRDDLLAKPGKAATGGGVVTARLNTKKDVLVETKEMQGYN
WWRNIRRT

>2[4Fe-4S]ST12(ret:RHE_CH03744) Rhizobium etli CFN 42

MSRAQDRIGTWIGWTFFADLANALTLTFGYMFSSPVTMQYPDTEKWLPYSRYRGHHFLKR
DEEGEIKCVACELCARICPCDCIEVVPYEDEKGNRHPAKFEIDTARCLFCGLCEDACPAD
AIALGQQYEFSSFSSADLVIGRDDLLAKPGKAATGGGVVSARLNTKKDVLVETKEMQGYN
WWRNIRRT

>2[4Fe-4S]ST12(rec:RHECIAT_CH0003640) Rhizobium etli CIAT 652

MSRAQDRIGTWIGWTFFADLANALTLTLGYMFARPVTMQYPDREKWLPYSRYRGHHFLKR
DEEGEIKCVACELCARICPCDCIEVVPYEDEKGNRHPAKFEIDTARCLFCGLCEDACPAD
AIALGQQYEFSSFSSADLVIGRDDLLAKPGKAATGGGVVTAHLNTKKDVLVETKEMQGYN
WWRNIRRT

>2[4Fe-4S]ST12(rel:REMIM1_CH03823) Rhizobium etli bv. mimosae Mim1

MSRAQDRIGTWIGWTFFADLANALTLTFGYMFSSPVTMQYPDTEKWLPYSRYRGHHFLKR
DEEGEIKCVACELCARICPCDCIEVVPYEDEKGNRHPAKFEIDTARCLFCGLCEDACPAD
AIALGQQYEFSSFSSADLVIGRDDLLAKPGKAATGGGVVSARLNTKKDVLVETKEMQGYN

WWRNIRRT

>2[4Fe-4S]ST12(rep:IE4803_CH03631) Rhizobium etli bv. phaseoli IE4803

MSRAQDKVGTWIGWAFFADLASALVLTFGYLFSRTVTMQYPDKEKWLPYSRYRGHHFLKR
DEGGEIKCVACELCARICPCDCIEVVPYEDEKGNRHPAKFEIDTARCLFCGLCEDACPAD
AIALGQQYEFSSFSSRDLVIGRDDLLAKPGKAATGGGVVTARLTTKKDVLVETKEMQGYN
WWRNIRRT

>2[4Fe-4S]ST12(rei:IE4771_CH03668) Rhizobium sp. IE4771

MSRAQDKAGRWIGWAFFADLANGLALTFGYLFSRTVTMQYPDKEKWLPYSRYRGHHFLKR
DEAGEIKCVACELCARICPCDCIEVVPYEDEKGNRHPAKFEIDTARCLFCGLCEDACPAD
AIALGQQYEFSSFSSRDLVIGRDDLLAKPGKAATGGGVVTARLNTKKDVLVETKEKQGYN
WWRNIRRT

>2[4Fe-4S]ST12(rlu:RLEG12_28990) Rhizobium leguminosarum bv. trifolii CB782

MSRAQDRVGTWIGWAFFADLASALALTFGYLFSRTVTMQYPDKEKWLPYSRYRGHHFLTR
DEEGEIKCVACELCARICPCDCIEVVPYEDEKGNRHPAKFEIDTARCLFCGLCEDACPAD
AIALGQQYEFSSFSSRDLVIGRDDLLAKPGKAATGGGVVAARLNTTKDVLVETKDTKGYN
WWRNIRRT

>2[4Fe-4S]ST12(rhn:AMJ98_CH03609) Rhizobium sp. N1341

MSRAQDRVGTWIGWAFFADLANALALTFGYLFSRTVTMQYPDKEKWLPYSRYRGHHFLKR

DEEGEIKCVACELCARICPCDCIEVVPYEDEKGNRHPAKFEIDTARCLFCGLCEDACPAD
AIALGQQYEFSSFSSADLVIGRDDLLVKPSKAATGGGVVAARLNTKKDVLVQTKETQGYN
WWRNIRRT

>2[4Fe-4S]ST12(rhx:AMK02_CH03497) Rhizobium sp. N731

MSRAQDRVGTWIGWAFFADLANALALTFGYLFSRTVTMQYPDKEKWLPYSRYRGHHFLKR
DEEGEIKCVACELCARICPCDCIEVVPYEDEKGNRHPAKFEIDTARCLFCGLCEDACPAD
AIALGQQYEFSSFSSADLVIGRDDLLVKPGKAATGGGVVAARLNTKKDVLVQTKETQGYN
WWRNIRRT

>2[4Fe-4S]ST12(rhl:LPU83_pLPU83d0711) Rhizobium favelukesii

MSRARNSIGTWIGWTFFADLASGLALTFSYMFSRSVTMQYPDKEKWLPYSRYRGHHFLTR
DEEGEIKCVACELCARICPCDCIEVVPYEDEKGNRHPAKFEIDTARCLFCGLCEDACPAD
AIALGQQYEFSSFSSRDLVVGRDDLLSKPGKAATGGGVIAARLNTKKDVLVETKETQGYN
WWRNIRRT

>2[4Fe-4S]ST12(smx:SM11_pC0823) Sinorhizobium meliloti SM11

MPSRKLWARTCKTTLRSRSEAAMSRALDKAGRWIGWAFFADLANGLALTFGYMFSRPVTM
QYPDKEKWLPYSRYRGHHFLKRDDEGEIKCVACELCARICPCDCIEVVPYEDEKGNRRPA
KFEIDTARCLFCGLCEDACPADAIALGQQYEFSSFSSRDLVIGRDDLLAKPGKAMTGGGV
VAARLNTERDVLVEASEPRGYNWWRNIRRK

>2[4Fe-4S]ST12(smi:BN406_04563) Sinorhizobium meliloti Rm41

MSRALDKAGRWIGWAFFADLANGLALTFGYMFSRPVTMQYPDKEKWLPYSRYRGHHFLKR
DDEGEIKCVACELCARICPCDCIEVVPYEDEKGNRRPAKFEIDTARCLFCGLCEDACPAD
AIALGQQYEFSSFSSRDLVIGRDDLLAKPGKAMTGGGVVAARLNTERDVLVEASEPRGYN
WWRNIRRK

>2[4Fe-4S]ST12(smeg:C770_GR4pC0549) Sinorhizobium meliloti GR4

MSRALDKAGRWIGWAFFADLANGLALTFGYMFSRPVTMQYPDKEKWLPYSRYRGHHFLKR
DDEGEIKCVACELCARICPCDCIEVVPYEDEKGNRRPAKFEIDTARCLFCGLCEDACPAD
AIALGQQYEFSSFSSRDLVIGRDDLLAKPGKAMTGGGVVAARLNTERDVLVEASEPRGYN
WWRNIRRK

>2[4Fe-4S]ST12(smel:SM2011_a1519) Sinorhizobium meliloti 2011

MSRALDKAGRWIGWAFFADLANGLALTFGYMFSRPVTMQYPDKEKWLPYSRYRGHHFLKR
DDEGEIKCVACELCARICPCDCIEVVPYEDEKGNRRPAKFEIDTARCLFCGLCEDACPAD
AIALGQQYEFSSFSSRDLVIGRDDLLAKPGKAMTGGGVVAARLNTERDVLVEASEPRGYN
WWRNIRRK

>2[4Fe-4S]ST12(smer:DU99_21870) Sinorhizobium meliloti RMO17

MSRALDKAGRWIGWAFFADLANGLALTFGYMFSRPVTMQYPDKEKWLPYSRYRGHHFLKR
DDEGEIKCVACELCARICPCDCIEVVPYEDEKGNRRPAKFEIDTARCLFCGLCEDACPAD
AIALGQQYEFSSFSSRDLVIGRDDLLAKPGKAMTGGGVVAARLNTERDVLVEASEPRGYN
WWRNIRRK

>2[4Fe-4S]ST12(smk:Sinme_6021) Sinorhizobium meliloti AK83

MSRALDKAGRWIGWAFFADLANGLALTFGYMFSRPVTMQYPDKEKWLPYSRYRGHHFLKR
DDDGEIKCVACELCARICPCDCIEVVPYEDEKGNRRPAKFEIDTARCLFCGLCEDACPAD
AIALGQQYEFSSFSSRDLVIGRDDLLAKPGKAMTGGGVVAARLNTERDVLVEASEPRGYN
WWRNIRRK

>2[4Fe-4S]ST12(smq:SinmeB_6340) Sinorhizobium meliloti BL225C

MSRALDKAGRWIGWAFFADLANGLALTFGYMFSRPVTMQYPDKEKWLPYSRYRGHHFLKR
DDDGEIKCVACELCARICPCDCIEVVPYEDEKGNRRPAKFEIDTARCLFCGLCEDACPAD
AIALGQQYEFSSFSSRDLVIGRDDLLAKPGKAMTGGGVVAARLNTERDVLVEASEPRGYN
WWRNIRRK

>2[4Fe-4S]ST12(smd:Smed_3615) Sinorhizobium medicae

MHAAQKLWVRTCKKTLRSRSEAAMSHALDKAGRWIGWAFFADLANGLALTFGYMFSKPVT
MQYPDKEKWLPYSRYRGHHFLKRDAEGEIKCVACELCAQICPCDCIEVVPYEDEKGNRRP
AKFEIDTARCLFCGLCEDACPADAIALGQQYEFSSFSSRDLVIGRDDLLAKPGKAMTGGG
VVAARLSTERDVLVEASEPRGYNWWRNIRRK

>2[4Fe-4S]ST12(sfh:SFHH103_02098) Sinorhizobium fredii HH103

MSRARDTIGTWIGWTFFADLANGLALTFGYMFSKSVTMQYPDKEKWLPYSRYRGHHFLKR
DEEGEIKCVACELCARICPCYCIEVVPYEDEKGNRRPAKFEIDTARCLFCGLCEDACPAD

AIALGQQYEFSSFSSRDLVIGRDDLLAKPGKAATGGGVVAARLNTQKDVLVETDEQQGYN
WWRNIRRR

>2[4Fe-4S]ST12(eah:FA04_27760) Ensifer adhaerens Casida A

MSRVVDRAGTWIGWALFADLASAFTLTFGYMFSKPVTMQYPDKEKWLPYSRYRGHHFLKR
DEEGEIKCVACELCARICPCDCIEVVPYEDENGNRHPAKFEIDTARCLFCGLCEDACPAD
AIALGQQYEFSSFSSRDLVIGRDDLLEKPGKAMAGGGVVAARLNTDEDVLVETSEPHGYN
WWRNIRRT
>2[4Fe-4S]ST12(ead:OV14_b1479) Ensifer adhaerens OV14

MFFADLAKALALTFGYMFSKSVTMQYPDKEKWLPYSRYRGHHFLKRDDQGEIKCVACELC
ARICPCYCIEVVPYEDEKGNRRPAKFEIDTARCLFCGLCEDACPADAIALGQQYEFSSFS
SRDLVIGRDDLLGKPGKATTGGGVVAAHLNTDGDVLVETSELQGYNWWRNIRRT
>2[4Fe-4S]ST12(same:SAMCFNEI73_Ch2563) Sinorhizobium Americanum

MSRAQDMIGTWIGWTFFADLAKGLGLTFGYMFSKTVTMQYPDKEKWLPYSRYRGHHFLKR
DEEGEIKCVACELCARICPCYCIEVVPYEDEKGNRRPIKFEIDTARCLFCGLCEDACPAD
AIALGQQYEFSSFSSRDLVIGRDDLLAKPGKAATGGGVVAARLNTENDVLVETNEPQGYN
WWRNIRRR
>2[4Fe-4S]ST12(sfd:USDA257_c45940) Sinorhizobium fredii USDA 257

MSRAQDIIGTWIGWTFFADLANGLALTFGYMFSRTVTMQYPDKEKWLPYSRYRGHHFLKR
DEEGEIKCVACELCARICPCYCIEVVPYEDDKGNRRPAKFEIDTARCLFCGLCEDACPAD
AIALGQQYEFSSFSSRDLVIGRDDLLAKPGKAATGGGVVAARLNTTKDVLVESNERQGYN
WWRNIRRR

>2[4Fe-4S]ST12(rhi:NGR_c22040) Sinorhizobium fredii NGR234

MSRPRDTIGTWIGWTFFADLATGLALTFGYMFSKTVTMQYPDKEKWLPYSRYRGHHFLKR

DEEGEIKCVACELCARICPCYCIEVVPYEDEKGNRRPAKFEIDTARCLFCGLCEDACPAD

AIALGQQYEFSSFSSRDLVIGRDDLLAKPGKAATGGGVVAARLNTQKDVLVETDERQGYN

WWRNIRRK

>2[4Fe-4S]ST12(pde:Pden_2238) Paracoccus denitrificans
MAFDFARATKYFLMWDFIKGFGLGMRYFVSPKPTLNYPHEKGPLSPRFRGEHALRRYPNG
EERCIACKLCEAVCPAQAITIDAEPREDGSRRTTRYDIDMTKCIYCGFCQEACPVDAIVE
GPNFEYATETREELFYDKQKLLANGERWEAEIARNLQLDAPYR
>2[4Fe-4S]ST12(ccr:CC_1942) Caulobacter vibrioides CB15
MFQRITQAVKGAALLDFAGAFGLAMKYMVAPKKTVIYPNERNPQSPRFRGEHALRRYPSG
EERCIACKLCEAICPAQAITIEAEPREDGSRRTTRYDIDMVKCIYCGLCQEACPVDAIVE
GPNTEFATETREELYYDKERLLDNGDRWERLIAKNLELDAPYR
>2[4Fe-4S]ST12(ccs:CCNA_02020) Caulobacter vibrioides NA1000
MFQRITQAVKGAALLDFAGAFGLAMKYMVAPKKTVIYPNERNPQSPRFRGEHALRRYPSG
EERCIACKLCEAICPAQAITIEAEPREDGSRRTTRYDIDMVKCIYCGLCQEACPVDAIVE
GPNTEFATETREELYYDKERLLDNGDRWERLIAKNLELDAPYR
>2[4Fe-4S]ST12(cak:Caul_2824) Caulobacter sp. K31
MMMQRISQAIKGVALLDFAGAFGLGIKYMLAPKKTVIYPNERGPQSPRFRGEHALRRYPS
GEERCIACKLCEAICPAQAITIEAEPRDDGSRRTTRYDIDMVKCIYCGLCQEACPVDAIV
EGPNIEFAVETREELYYDKEKLLDNGDRWERLIAKNLELDAPYR
>2[4Fe-4S]ST12(cse:Cseg_2229) Caulobacter segnis
MFQRITQAVKGAALLDFAGAFGLAMKYMVAPKKTVIYPNERNPQSPRFRGEHALRRYPSG
EERCIACKLCEAVCPAQAITIEAEPREDGSRRTTRYDIDMVKCIYCGLCQEACPVDAIVE
GPNIEFATETREELYYDKERLLDNGDRWERLIAKNLELDAPYR
>2[4Fe-4S]ST12(pzu:PHZ_c1796) Phenylobacterium zucineum
MLNRIGQAVKGAALLDFAGAFGLAMKHMARPKKTVQYPHERNPQSPRFRGEHALRRYPNG
EERCIACKLCEAVCPAQAITIEAEPRADGSRRTTRYDIDMVKCIYCGLCQEACPVDAIVE
GPNLEFAVETREELYYDKERLLDNGDRWEREIAKNLELDAPYR
>2[4Fe-4S]ST12(aex:Astex_2812) Asticcacaulis excentricus
MIGRLVQAAKGALLLDFVGATGLAVKYMFKPKKTINYPFEKGPISPRFRGEHALRRYPNG
EERCIACKLCEAICPAQAITIEAEPREDGSRRTTRYDIDMVKCIYCGLCQEACPVDAIVE
GPNFEFATDTREELYYDKSKLLANGDRWEREIARALELDAPYR
>2[4Fe-4S]ST12(mmr:Mmar10_1363) Maricaulis maris
MGRIQQAFRGALLMDFWSAFGLGMRYFFAKKATLNYPFEKGPLSPRFRGQHALRRYANGE

ERCIACKLCEAICPAQAITIEAEPRSDGSRRTTRYDIDMTKCIYCGYCQEACPVDAIVEG
PNFEFAAETREELFYDKAKLLENGDRWEREIAKNLELDAPYR
>2[4Fe-4S]ST12(hne:HNE_1753) Hyphomonas neptunium
MSLAQTLRGALLTDFMGAAWIAVREMFRRKATVNYPFEKNPLSPRFRGEHVLRRYPSGEE
RCIACKLCEAICPAQAITIEAEPREDGARRTTRYDIDMVKCIYCGFCQEACPVDAIVEGP
NFEFATETREELFYDKERLLANGDRWERLIAKNLELDAPYR
>2[4Fe-4S]ST12(hba:Hbal_1732) Hirschia baltica
MSRISQAIKGAMLSDIVSALGLALRYMFTRKATVNYPFEKGPLSPRFRGEHALRRYANGE
ERCIACKLCEAVCPAQAITIEAEPRSDGSRRTTRYDIDMTKCIYCGFCQEACPVDAIVEG
PNFEFSTETREELFYDKDRLLSNGDRWERQIAKNLELDARYR
>2[4Fe-4S]ST12(hbc:AEM38_05520) Hyphomonadaceae bacterium UKL13-1
MSIAQALKGALMLDFAGAFGLAMKKFFEPKFTINYPFEKGHISPRFRGEHALRRYANGEE
RCIACKLCEAICPAQAITIEAEPRDDGSRRTTRYDIDMTKCIYCGFCQEACPVDAIVEGP
NFEFATETREELFYDKDKLLANGDRWEREIAKNLELDAAYR
>2[4Fe-4S]ST12(rsp:RSP_2523) Rhodobacter sphaeroides 2.4.1
MARTSAIDYGRAAKYFLLLDFIKGFGLGMKYFFAPKHTVNYPHEKGPLSPRFRGEHALRR
YPNGEERCIACKLCEAVCPAQAITIDAEPREDGSRRTTRYDIDMTKCIYCGFCQEACPVD
AIVEGPNFEFSTETREELFYNKDRLLENGARWEAEIARNLELDAPYR
>2[4Fe-4S]ST12(rsh:Rsph17029_1185) Rhodobacter sphaeroides ATCC 17029
MARTSAIDYGRAAKYFLLLDFIKGFGLGMKYFFAPKHTVNYPHEKGPLSPRFRGEHALRR
YPNGEERCIACKLCEAVCPAQAITIDAEPREDGSRRTTRYDIDMTKCIYCGFCQEACPVD
AIVEGPNFEFSTETREELFYNKDRLLENGARWEAEIARNLELDAPYR
>2[4Fe-4S]ST12(rsq:Rsph17025_1997) Rhodobacter sphaeroides ATCC 17025
MARTGAIDYGRAVKYFLLLDFIKGFGLGMKYFFAPKHTLNYPHEKGPLSPRFRGEHALRR
YPNGEERCIACKLCEAVCPAQAITIDAEPREDGSRRTTRYDIDMTKCIYCGFCQEACPVD
AIVEGPNFEFSVETREELFYTKEKLLQNGDRWEAEIARNLELDAPYR
>2[4Fe-4S]ST12(rsk:RSKD131_0838) Rhodobacter sphaeroides KD131
MARTSAIDYGRAAKYFLLLDFIKGFGLGMKYFFAPKHTVNYPHEKGPLSPRFRGEHALRR
YPNGEERCIACKLCEAVCPAQAITIDAEPREDGSRRTTRYDIDMTKCIYCGFCQEACPVD
AIVEGPNFEFSTETREELFYNKDRLLENGARWEAEIARNLELDAPYR
>2[4Fe-4S]ST12(pami:JCM7686_2499) Paracoccus aminophilus
MALDLARATKYFLMWDFLKGFGLGMRYFFSPKPTLNYPHEKGPLSPRFRGEHALRRYPNG
EERCIACKLCEAICPAQAITIDAEPREDGSRRTTRYDIDMTKCIYCGFCQEACPVDAIVE
GPNFEYSTETREELFYDKEKLLDNGARWEAEIARNLAMDAPYR
>2[4Fe-4S]ST12(pye:A6J80_15510) Paracoccus yeei
MAFDFARATKYFLMWDFIKGFGLGMRYFVSPKPTLNYPHEKGPLSPRFRGEHALRRYPNG
EERCIACKLCEAICPAQAITIDAEPREDGSRRTTRYDLDMTKCIYCGFCQEACPVDAIVE
GPNFEYATETREELFYDKQKLLDNGARWEAEIARNLQMDAPYR
>2[4Fe-4S]ST12(cid:P73_3435) Celeribacter indicus

MALDYTRAAKYFLMTDIWTGFKIGLRYFFAPKATINYPHEKGPLSPRFRGEHALRRYPNG
EERCIACKLCEAICPAQAITIDAEPREDGSRRTTRYDIDMTKCIYCGFCQEACPVDAIVE
GPNFEFSTETREELFYDKQKLLDNGARWEAEIARNLELDAPYR
>2[4Fe-4S]ST12(cmar:IMCC12053_2424) Celeribacter marinus
MAMDYTRAAKYFLMTDIIAGFRIGLRYFFSPKVTINYPHEKGPLSPRFRGEHALRRYPNG
EERCIACKLCEAICPAQAITIDAEPRDDGSRRTTRYDIDMTKCIYCGFCQEACPVDAIVE
GPNFEFATESREELFYDKDKLLDNGARWEAEIARNLELDAPYR
>2[4Fe-4S]ST12(con:TQ29_06380) Confluentimicrobium sp. EMB200-NS6
MAIDYTRATRYFLLADFINGFRLGFKYFFAPKATINYPHEKGPLSPRFRGEHALRRYPNG
EERCIACKLCEAICPAQAITIDAEPREDGSRRTTRYDIDMTKCIYCGFCQEACPVDAIVE
GPNFEFSTETREELFYDKAKLLENGEQWEAEIARNLELDAPYR
>2[4Fe-4S]ST12(daa:AKL17_2228) Defluviimonas alba
MALDYTRAAKYFLMFDFIKGFGLGMKYFFAPKETLNYPHEKGPLSPRFRGEHALRRYPNG
EERCIACKLCEAICPAQAITIDAEPREDGSRRTTRYDIDMTKCIYCGFCQEACPVDAIVE
GPNFEFSTETREELFYDKARLLDNGARWEAEIARNLEMDAPYR
>2[4Fe-4S]ST12(yan:AYJ57_02660) Yangia sp. CCB-MM3
MASIDYGRAAKYFLLADFIKGFQLGLKYFFAPKPTLNYPHEKGYLSPRFRGEHALRRYPN
GEERCIACKLCEAICPAQAITIDAEPRDDGSRRTTRYDIDMTKCIYCGFCQEACPVDAIV
EGPNFEFSTETREELFYDKDKLLANGERWEAEIARNLELDAPYR
>2[4Fe-4S]ST12(tpro:Ga0080559_TMP4774) Salipiger profundus
MTQIDYGRAAKYFLLFDFIKGFQLGLRYFFAPKPTLNYPHEKGYLSPRFRGEHALRRYPN
GEERCIACKLCEAICPAQAITIDAEPRDDGSRRTTRYDIDMTKCIYCGFCQEACPVDAIV
EGPNFEFSTETREELFYDKEKLLENGERWEAEIARNLELDAPYR
>2[4Fe-4S]ST12(paby:Ga0080574_TMP4554) Pelagibaca abyssi
MTQIDYGRAAKYFLLADFIKGFALGMKYFFAPKPTLNYPHEKGPLSPRFRGEHALRRYPN
GEERCIACKLCEAICPAQAITIDAEPRDDGSRRTTRYDIDMTKCIYCGFCQEACPVDAIV
EGPNFEFSTETREELFYDKQKLLENGERWEAEIARNLELDAPYR
>2[4Fe-4S]ST12(thw:BMG03_08295) Thioclava nitratireducens
MAFDYNRAAKYFLLVDFIKGFGLGMRYFFAPKPTLNYPHEKGPLSPRFRGEHALRRYPNG
EERCIACKLCEAICPAQAITIDAEPRADGSRRTTRYDIDMTKCIYCGFCQEACPVDAIVE
GPNFEFSTETREELFYDKQKLLENGARWEAEIARNLELDAPYR
>2[4Fe-4S]ST12(sil:SPO2770) Ruegeria pomeroyi
MTQIDYTRAAKYFLLQDFWVGMKLGLKYFFSPKATINYPHEKGPLSPRFRGEHALRRYPN
GEERCIACKLCEAICPAQAITIDAEPREDGSRRTTRYDIDMTKCIYCGFCQEACPVDAIV
EGPNFEFATETREELFYDKEKLLSNGDRWEAEIARNLELDAPYR
>2[4Fe-4S]ST12(sit:TM1040_0754) Ruegeria sp. TM1040
MTQIDYTRAAKYFLLQDVWQGFKLGLKYFFAPKATINYPHEKGPLSPRFRGEHALRRYPN
GEERCIACKLCEAVCPAQAITIDAEPREDGSRRTTRYDIDMTKCIYCGFCQEACPVDAIV
EGPNFEFATETREELFYDKDKLLANGERWEAEIARNLELDAPYR

>2[4Fe-4S]ST12(rcp:RCAP_rcc01531) Rhodobacter capsulatus
MAFDYVRAAKYFVLWDFIKGFALGMKYFVAPKPTLNYPHEKGPLSPRFRGEHALRRYPSG
EERCIACKLCEAICPAQAITIDAEPRDDGSRRTTRYDIDMTKCIYCGYCQEACPVDAIVE
GPNFEYATETREELFYTKEKLLENGARWEAEIARNIEMDAPYR
>2[4Fe-4S]ST12(rli:RLO149_c024940) Roseobacter litoralis
MTQIDYARHAKYFLLQDFWVGMKLGLKYFFAPKATLNYPHEKGPLSPRFRGEHALRRYPN
GEERCIACKLCEAVCPAQAITIDAEPRDDGSRRTTRYDIDMTKCIYCGFCEEACPVDAIV
EGPNFEFSTETREELYYDKDRLLANGERWEAEIARNLEMDAPYR
>2[4Fe-4S]ST12(pga:PGA1_c10490) Phaeobacter inhibens DSM 17395
MANIDYTRAAKYFLLQDFWVGFKLGLKYFFAPKATLNYPHEKGPLSPRFRGEHALRRYPN
GEERCIACKLCEAVCPAQAITIDAEPREDGSRRTTRYDIDMTKCIYCGFCQEACPVDAIV
EGPNFEFATETREELFYDKDKLLSNGERWEAEIARNLELDAPYR
>2[4Fe-4S]ST12(pgl:PGA2_c10330) Phaeobacter inhibens 2.10
MANIDYTRAAKYFLLQDFWVGFKLGLKYFFAPKATLNYPHEKGPLSPRFRGEHALRRYPN
GEERCIACKLCEAVCPAQAITIDAEPREDGSRRTTRYDIDMTKCIYCGFCQEACPVDAIV
EGPNFEFATETREELFYDKEKLLSNGERWEAEIARNLELDAPYR
>2[4Fe-4S]ST12(pgd:Gal_02444) Phaeobacter gallaeciensis DSM 26640
MANIDYTRAAKYFLLQDFWVGFKLGLKYFFAPKATLNYPHEKGPLSPRFRGEHALRRYPN
GEERCIACKLCEAVCPAQAITIDAEPREDGSRRTTRYDIDMTKCIYCGFCQEACPVDAIV
EGPNFEFATETREELFYDKDKLLSNGERWEAEIARNLELDAPYR
>2[4Fe-4S]ST12(php:PhaeoP97_01020) Phaeobacter porticola
MANIDYTRAAKYFLLQDFWVGFKLGLKYFFAPKATLNYPHEKGPLSPRFRGEHALRRYPN
GEERCIACKLCEAVCPAQAITIDAEPREDGSRRTTRYDIDMTKCIYCGFCQEACPVDAIV
EGPNFEFATETREELFYDKDKLLSNGERWEAEIARNLELDAPYR
>2[4Fe-4S]ST12(lmd:METH_12100) Leisingera methylohalidivorans
MTQIDYTRAAKYFLLQDFWVGFKLGLKYFFAPKATLNYPHEKGPLSPRFRGEHALRRYPN
GEERCIACKLCEAVCPAQAITIDAEPREDGSRRTTRYDIDMTKCIYCGFCQEACPVDAIV
EGPNFEFATETREELFYDKDKLLANGDRWEAEIARNLEIDAPYR
>2[4Fe-4S]ST12(rsu:NHU_01353) Rhodovulum sulfidophilum
MAAIDWQRAGKYFLLMDFLQGFRLGMKYFFAPKATLSYPHEKGPLSPRFRGEHALRRYPS
GEERCIACKLCEAICPAQAITIDAEPREDGSRRTTRYDIDMTKCIYCGFCQEACPVDAIV
EGPNFEFATETREELFYDKQKLLENGDRWEAEIARNLELDAPYR
>2[4Fe-4S]ST12(rhc:RGUI_0424) Rhodovulum sp. P5
MAAIDWTRAGKYFLLMDFLEGMRLGMKYFLAPKATVNYPHEKGPLSPRFRGEHALRRYPN
GEERCIACKLCEAICPAQAITIDAEPRADGSRRTTRYDIDMTKCIYCGFCQEACPVDAIV
EGPNFEFATETREELFYDKQKLLDNGERWEAEIARNLEMDAPYR
>2[4Fe-4S]ST12(tom:BWR18_08725) Tateyamaria omphalii
MTQIDYGRAAKYFLLQDFWVGMKLGLKYFFAPKATLNYPHEKGPLSPRFRGEHALRRYPN
GEERCIACKLCEAICPAQAITIDAEPREDGSRRTTRYDIDMTKCIYCGFCQEACPVDAIV

EGPNFEFSTETREELYYDKEKLLENGERWEAEIARNLELDAPYR
>2[4Fe-4S]ST12(jan:Jann_1187) Jannaschia sp. CCS1
MTQIDYTRAAKYFLLADFFKGFKLGMKYFFAPKVTINYPHEKGPLSPRFRGEHALRRYPN
GEERCIACKLCEAICPAQAITIDAEPREDGSRRTTRYDIDMTKCIYCGFCQEACPVDAIV
EGPNFEFATETREELYYDKEKLLANGERWESAIAHNLELDAPYR
>2[4Fe-4S]ST12(rde:RD1_3275) Roseobacter denitrificans
MTQVDYTRAAKYFLLQDFWVGMKLGMKYFFAPKATLNYPHEKGPLSPRFRGEHALRRYPN
GEERCIACKLCEAVCPAQAITIDAEPRDDGSRRTTRYDIDMTKCIYCGFCEEACPVDAIV
EGPNFEFSTETREELYYDKDRLLANGERWEAEIARNLEMDAPYR
>2[4Fe-4S]ST12(dsh:Dshi_1322) Dinoroseobacter shibae
MAQMDYTRAAKYFLLFDFFAGFKLGLKYFFKPKATLAYPHEKGPLSPRFRGEHALRRYPN
GEERCIACKLCEAICPAQAITIDAEPRDDGSRRTTRYDIDMTKCIYCGFCQEACPVDAIV
EGPNFEFATETREELFYDKEKLLDNGERWEAEIARNLELDAPYR
>2[4Fe-4S]ST12(oar:OA238_c28590) Octadecabacter arcticus
MTEIDYTRAAKYFLLADFAKGFRLGFKYFFAPKATLNYPHEKGPLSPRFRGEHALRRYPN
GEERCIACKLCEAVCPAQAITIDAEPRDDGSRRTTRYDIDMTKCIYCGFCQEACPVDAIV
EGPNFEFSTETREELYYDKEKLLDNGDRWEAEIARNLEMDAPYR
>2[4Fe-4S]ST12(otm:OSB_20590) Octadecabacter temperatus
MSQIDYTRHAKYFLMTDIIKGFALGFKYFFAPKATLNYPHEKGPLSPRFRGEHALRRYPN
GEERCIACKLCEAVCPAQAITIDAEPRDDGSRRTTRYDIDMTKCIYCGFCQEACPVDAIV
EGPNFEFSTETREELYYDKEKLLDNGDRWEAEIARNLELDAPYR
>2[4Fe-4S]ST12(red:roselon_00176) Roseibacterium elongatum
MTQIDYTRAAKYFLLADFFKGMKLGLKYFFAPKATLNYPHEKGPLSPRFRGEHALRRYPN
GEERCIACKLCEAVCPAQAITIDAEPRDDGSRRTTRYDIDMTKCIYCGFCQEACPVDAIV
EGPNFEFATETREELYYDKAKLLENGERWEAEIARNLEMDAPYR
>2[4Fe-4S]ST12(ptp:RCA23_c13650) Planktomarina temperata
MANIDYTRRVKYFLLQDFWAAFKLGMKYFFRPKATLNYPHEKGPLSPRFRGEHALRRYPN
GEERCIACKLCEAICPAQAITIDAEPRDDGSRRTTRYDIDMTKCIYCGFCQEACPVDAIV
EGPNFEFSTETREELFYDKSKLLENGDRWEAEIARNLELDAPYR
>2[4Fe-4S]ST12(malg:MALG_02350) Marinovum algicola
MADIDYGRAAKYFLLTDFIQGFKLGLKYFFAPKATLNYPHEKGPLSPRFRGEHVLRRYPN
GEERCIACKLCEAICPAQAITIDAEPREDGSRRTTRYDIDMTKCIYCGFCQEACPVDAIV
EGPNFEFATETREELFYDKAKLLENGDRWEAEIAANLAVDAPYR
>2[4Fe-4S]ST12(suam:BOO69_06655) Sulfitobacter sp. AM1-D1
MTQIDYSRAAKYFLLQDFAKGFALGFKQMFRPKATVNYPHEKGPLSPRFRGEHALRRYPN
GEERCIACKLCEAICPAQAITIDAEPREDGSRRTTRYDIDMTKCIYCGFCQEACPVDAIV
EGPNFEFSTETREELYYDKAKLLANGERWEAEIARNLELDAPYR
>2[4Fe-4S]ST12(lvs:LOKVESSMR4R_01791) Yoonia vestfoldensis
MSKTNMDYTRAAKYFLLQDFWVGFKLGMKYFFAPKATLNYPHEKGPLSPRFRGEHALRRY

PNGEERCIACKLCEAVCPAQAITIDAEPRDDGSRRTTRYDIDMTKCIYCGFCQEACPVDA
IVEGPNFEFSTETREELYYDKAKLLENGDRWESEIARNLALDAPYR
>2[4Fe-4S]ST12(kvu:EIO_2274) Ketogulonicigenium vulgare Y25
MSQIDYTRAAKYFLLKDVWDGFRLGMKYFFRPRPTVNYPHEKVPLSPRFRGEHALRRYPS
GEERCIACKLCEAICPAQAITIDAEPRDDGSRRTTRYDIDMTKCIYCGFCQEACPVDAIV
EGPNFEFATESREELFYDKDKLLANGDKWEAEIARNLAADAPYR
>2[4Fe-4S]ST12(kvl:KVU_1815) Ketogulonicigenium vulgare WSH-001
MSQIDYTRAAKYFLLKDVWDGFRLGMKYFFRPRPTVNYPHEKVPLSPRFRGEHALRRYPS
GEERCIACKLCEAICPAQAITIDAEPRDDGSRRTTRYDIDMTKCIYCGFCQEACPVDAIV
EGPNFEFATESREELFYDKDKLLANGDKWEAEIARNLAADAPYR
>2[4Fe-4S]ST12(kro:BVG79_01879) Ketogulonicigenium robustum
MSQIDYTRAAKYFLLKDVWDGFRLGMKYFFRPRPTVNYPHEKVPLSPRFRGEHALRRYPS
GEERCIACKLCEAICPAQAITIDAEPRDDGSRRTTRYDIDMTKCIYCGFCQEACPVDAIV
EGPNFEFATESREELFYDKDRLLANGDKWEAEIARNLAADAPYR
>2[4Fe-4S]ST12(rmm:ROSMUCSMR3_00221) Roseovarius mucosus
MTKIDYGRAAGYFLLSDFFKGFKLGLKYFFAPKATLNYPHEKGPLSPRFRGEHALRRYPN
GEERCIACKLCEAICPAQAITIDAEPRDDGSRRTTRYDIDMTKCIYCGFCQEACPVDAIV
EGPNFEFATETREELFYDKARLLANGDRWEAEIARNLEMDAPYR
>2[4Fe-4S]ST12(nar:Saro_2292) Novosphingobium aromaticivorans
MTVGQLIKSFTLWEFVKAHWLTLKYFFKPKATINYPFEKNPLSPRFRGEHALRRYPNGEE
RCIACKLCEAVCPAQAITIEAEPREDGSRRTTRYDIDMTKCIFCGFCQEACPVDAIVEGP
NFEYATETREELLYDKAKLLSNGDKWERAIAANLEADAPYR
>2[4Fe-4S]ST12(4npp:PP1Y_AT27349) Novosphingobium sp. PP1Y
MTVGHLIKSFTLWEFVKAHWLTLKYFFKPKATINYPFEKNPLSPRFRGEHALRRYPNGEE
RCIACKLCEAICPAQAITIEAAPRADGSRRTTRYDIDMTKCIYCGFCQEACPVDAIVEGP
NFEYATETREELLYDKAKLLANGDKWERAIAANLEADAPYR
>2[4Fe-4S]ST12(npn:JI59_02950) Novosphingobium pentaromativorans
MTVGHLIKSFTLWEFVKAHWLTLKYFFKPKATINYPFEKNPLSPRFRGEHALRRYPNGEE
RCIACKLCEAICPAQAITIEAAPRADGSRRTTRYDIDMTKCIYCGFCQEACPVDAIVEGP
NFEYATETREELLYDKAKLLANGDKWERAIAANLEADAPYR
>2[4Fe-4S]ST12(eli:ELI_06625) Erythrobacter litoralis HTCC2594
MTTATQLLKSFTLWEFLKAHALTLKYFFKPKATINYPFEKNPLSPRFRGEHALRRYPNGE
ERCIACKLCEAVCPAQAITIESEPRDDGSRRTTRYDIDMTKCIYCGFCQEACPVDAIVEG
PNFEYSTETREELLYDKAKLLANGDKWERAIAANLEADAPYR
>2[4Fe-4S]ST12(elq:Ga0102493_112422) Erythrobacter litoralis DSM 8509
MTTATQLLKSFTLWEFLKAHALTLKYFFKPKVTINYPFEKSPLSPRFRGEHALRRYPNGE
ERCIACKLCEAVCPAQAITIESEPREDGSRRTTRYDIDMTKCIYCGFCQEACPVDAIVEG
PNFEYSTETREELLYDKAKLLANGDKWERAIAANLEADAPYR
>2[4Fe-4S]ST12(aep:AMC99_01251) Altererythrobacter epoxidivorans

MTVGQLIKSFTLWEFVKAHALTLKYFFKPKVTINYPFEKNPLSPRFRGEHALRRYPNGEE
RCIACKLCEAICPAQAITIESEPREDGSRRTTRYDIDMTKCIYCGFCQEACPVDAIVEGP
NFEYATETREELLYDKAKLLANGDKWERAIAANLEADAPYR
>2[4Fe-4S]ST12(anh:A6F65_01798) Altererythrobacter namhicola
MTALQLLKSFTLWEFVKAHALTLKYFFKPKVTINYPYEKNPLSPRFRGEHALRRYPNGEE
RCIACKLCEAVCPAQAITIESEPREDGSRRTTRYDIDMTKCIYCGFCQEACPVDAVVEGP
NFEYATETREELLYDKAKLLANGDKWERAIAANLEADAPYR
>2[4Fe-4S]ST12(cna:AB433_08100) Croceicoccus naphthovorans
MTALQLIKSYTLWEFVKAHWLTLKYFFKPKATINYPFEKNPLSPRFRGEHALRRYPNGEE
RCIACKLCEAVCPAQAITIEAEPREDGSRRTTRYDIDMTKCIYCGFCQEACPVDAVVEGP
NFEYATETREELLYDKAKLLANGDKWERAIAANLEADAPYR
>2[4Fe-4S]ST12(sal:Sala_1301) Sphingopyxis alaskensis
MSYVGHLVKSFTLWEFVKAHALTLKYFFKPKATINYPFEKNPLSPRFRGEHALRRYPNGE
ERCIACKLCEAVCPAQAITIEAEPRDDGSRRTTRYDIDMTKCIYCGFCQEACPVDAIVEG
PNFEYATETREELLYDKAKLLANGDKWERAIAANLAADAPYR
>2[4Fe-4S]ST12(sphk:SKP52_08520) Sphingopyxis fribergensis
MTTIAHIVKSFTLWEFVKAHWLTLKYFFKPKATINYPFEKNPLSPRFRGEHALRRYPNGE
ERCIACKLCEAVCPAQAITIEAEPRDDGSRRTTRYDIDMTKCIYCGFCQEACPVDAVVEG
PNFEFATETREELLYDKAKLLANGDKWERAIAANLAADAPYR
>2[4Fe-4S]ST12(sphp:LH20_11430) Sphingopyxis sp. 113P3
MSSIAHFVKTFTLWEFVQAHWLTLKYFFKPKATINYPFEKNALSPRFRGEHALRRYPNGE
ERCIACKLCEAICPAQAITIEAEPRDDGSRRTTRYDIDMTKCIYCGFCQEACPVDAIVEG
PNFEFSTETREELLYDKAKLLANGDKWERTIAANLAADAPYR
>2[4Fe-4S]ST12(smaz:LH19_09475) Sphingopyxis macrogoltabida 203
MSYVAHLVKSFTLWEFVKAHALTLKYFFKPKATINYPFEKNPLSPRFRGEHALRRYPNGE
ERCIACKLCEAVCPAQAITIEAEPRDDGSRRTTRYDIDMTKCIYCGFCQEACPVDAVVEG
PNFEFATETREELLYDKAKLLANGDKWERAIAANLAADAPYR
>2[4Fe-4S]ST12(ster:AOA14_15315) Sphingopyxis terrae
MSYVAHLVKSFTLWEFVKAHALTLKYFFKPKATINYPFEKNPLSPRFRGEHALRRYPNGE
ERCIACKLCEAICPAQAITIEAVPRDDGSRRTTRYDIDMTKCIYCGFCQEACPVDAIVEG
PNFEFATETREELLYDKAKLLANGDKWERAIAANLAADAPYR
>2[4Fe-4S]ST12(sgi:SGRAN_2176) Sphingopyxis granuli
MSNIAHLVRTFTLWEFVQAHWLTLKYFFKPKATINYPFEKNPLSPRFRGEHALRRYPNGE
ERCIACKLCEAVCPAQAITIEAEPRDDGSRRTTRYDIDMTKCIYCGFCQEACPVDAIVEG
PNFEFATETREELLYDKAKLLANGDKWERAIAANLAADAPYR
>2[4Fe-4S]ST12(sphl:LPB140_07145) Sphingopyxis sp. LPB0140
MSNISHLIKSFTLWEFAKAHWLTLKYFFKPKATINYPFEKNPISPRFRGEHALRRYPNGE
ERCIACKLCEAVCPAQAITIEAEPRDDGSRRTTRYDIDMTKCIFCGFCQEACPVDAVVEG
PNFEYATETREELLYDKAKLLENGDKWEQAIAANLAADAPYR

>2[4Fe-4S]ST12(sphi:TS85_08835) Sphingomonas sp. WHSC-8
MSVAQIIRSYTLWEFLKAHWLTLQYFFKPKATINYPYEKNPISPRFRGEHALRRYANGEE
RCIACKLCEAVCPALAITIEAEPREDGSRRTTRYDIDMTKCIYCGLCQEACPVDAIVEGP
NFEFSTETREELIYQKEKLLANGDRWERAIAANLAADAPYR
>2[4Fe-4S]ST12(cij:WG74_10875) Citromicrobium sp. JL477
MSIAQTIKAFTLYEFVKAHALTLKYLFKPKVTINYPYEKNPISPRFRGEHALRRYPNGEE
RCIACKLCEAVCPAQAITIESEPRADGSRRTTRYDIDMTKCIYCGFCQEACPVDAIVEGP
NFEYSTETREELLYDKAKLLANGDKWERAIAANLEADAPYR
>2[4Fe-4S]ST12(sphg:AZE99_06140) Sphingorhabdus sp. M41
MNIAQTIKAFTLWEFIGAHMLTLKYFFKAKKTINYPYEKNPISPRFRGEHALRRYPNGEE
RCIACKLCEAVCPAQAITIEAEPREDGSRRTTRYDIDMTKCIYCGFCQEACPVDAIVEGP
NFEYATETREELIYDKSKLLENGDKWERAIAANLAADAPYR
>2[4Fe-4S]ST12(blas:BSY18_872) Blastomonas sp. RAC04
MLQTLKAFTLYEFVKAHALTLKYFFKPKATINYPFEKNPLSPRFRGEHALRRYPNGEERC
IACKLCEAVCPAQAITIEAEPREDGSRRTTRYDIDMTKCIFCGFCQEACPVDAIVEGPNF
EYATETREELIYDKGKLLENGDKWERAIAANLEADAPYR
>2[4Fe-4S]ST12(aay:WYH_00626) Altererythrobacter atlanticus
MSVAQLVKSFTLWEFLKAHSLTLKYFFKPKATINYPYEKNPLSPRFRGEHALRRYPNGEE
RCIACKLCEAVCPAQAITIEAEPREDGSRRTTRYDIDMTKCIYCGFCQEACPVDAIVEGP
NFEYSTETREELLYDKAKLLANGDKWERAIAANLEADAPYR
>2[4Fe-4S]ST12(amx:AM2010_1299) Altererythrobacter marensis
MSVAHLIKSFTLWEFVKAHALTLKYFFKPKATINYPFEKNPISPRFRGEHALRRYPNGEE
RCIACKLCEAVCPAQAITIESEPRDDGSRRTTRYDIDMTKCIYCGFCQEACPVDAIVEGP
NFEYATETREELLYDKAKLLANGDKWERAIAANLEADAPYR
>2[4Fe-4S]ST12(ado:A6F68_02231) Altererythrobacter dongtanensis
MTIAHLIKSFTLWEFVKAHALTLKYFFKPKATVNYPFEKNPLSPRFRGEHALRRYPNGEE
RCIACKLCEAVCPAQAITIEAEPREDGSRRTTRYDIDMTKCIYCGFCQEACPVDAIVEGP
NFEYATETREELLYDKAKLLANGDKWERALAANLEADAPYR
>2[4Fe-4S]ST12(pns:A9D12_12635) Porphyrobacter neustonensis
MTSVSHLIKSFTLWEFLKAHALTLKYFFRPKVTINYPFEKAPLSPRFRGEHALRRYPNGE
ERCIACKLCEAVCPAQAITIESEPREDGSRRTTRYDIDMTKCIFCGFCQEACPVDAIVEG
PNFEYSTETREELLYDKAKLLSNGDKWERAIAANLEADAPYR
>2[4Fe-4S]ST12(porl:BG023_112022) Porphyrobacter sp. LM 6
MTTVSHLIKSFTLWEFLKAHALTLKYFFKPKVTINYPFEKAPLSPRFRGEHALRRYPNGE
ERCIACKLCEAVCPAQAITIESEPREDGSRRTTRYDIDMTKCIFCGFCQEACPVDAIVEG
PNFEYATETREELLYDKAKLLANGDKWERAIAANLEADAPYR
>2[4Fe-4S]ST12(swi:Swit_2993) Sphingomonas wittichii
MSLGHIIKSFTLWEFVKAHWLTLKYFFRAKATINYPFEKNPLSPRFRGEHALRRYPNGEE
RCIACKLCEAVCPAQAITIEAEPRDDGSRRTTRYDIDMTKCIFCGFCQEACPVDAIVEGP

NFEYSTETREELIYDKAKLLANGDRWEPAIAANLAADAPYR
>2[4Fe-4S]ST12(sphm:G432_00205) Sphingomonas sp. MM-1
MSLAHYVKTFTLWEFVKAHWLTLKYFFKPKATINYPFEKNPLSPRFRGEHALRRYPNGEE
RCIACKLCEAICPAQAITIEAEPRDDGSRRTTRYDIDMTKCIYCGFCQEACPVDAIVEGP
NFEYATETREELIYDKAKLLSNGDRWESAIAANIAADAPYR
>2[4Fe-4S]ST12(sjp:SJA_C1-13310) Sphingobium japonicum
MSLGYYVKSFTLWEFVKAHALTLKYFFKPKATINYPYEKNPISPRFRGEHALRRYPNGEE
RCIACKLCEAICPAQAITIEAQPRDDGSRRTTRYDIDMTKCIYCGFCQEACPVDAIVEGP
NFEFSTETREELIYDKAKLLENGDKWERAIAANLAADAPYR
>2[4Fe-4S]ST12(sch:Sphch_1178) Sphingobium chlorophenolicum
MSLGYYVKSFTLWEFVKAHWLTLKYFFKPKATINYPYEKNPISPRFRGEHALRRYPNGEE
RCIACKLCEAICPAQAITIEAQPREDGSRRTTRYDIDMTKCIYCGFCQEACPVDAVVEGP
NFEFSTETREELIYDKAKLLENGDKWERAIAANLAADAPYR
>2[4Fe-4S]ST12(ssy:SLG_23100) Sphingobium sp. SYK-6
MSVAHLIKSFTLWEFVKAHWLTLKYFFKPKATINYPYEKNPISPRFRGEHALRRYPNGEE
RCIACKLCEAICPAQAITIEAQPRDDGSRRTTRYDIDMVKCIYCGFCQEACPVDAIVEGP
NFEFATETREELIYDKAKLLANGDKWERAIAANLAADAPYR
>2[4Fe-4S]ST12(syb:TZ53_20545) Sphingobium sp. YBL2
MSLGYYVKSFTLWEFVKAHALTLKYFFKPKATINYPYEKNPISPRFRGEHALRRYPNGEE
RCIACKLCEAICPAQAITIEAQPRDDGSRRTTRYDIDMTKCIYCGFCQEACPVDAIVEGP
NFEFSTETREELIYDKAKLLENGDKWERAIAANLAADAPYR
>2[4Fe-4S]ST12(sbd:ATN00_16765) Sphingobium baderi
MSLGYYIKSFTLWEFVKAHWLTLKYFFKPKATINYPHEKNPISPRFRGEHVLRRYPNGEE
RCIACKLCEAICPAQAITIEAQPREDGSRRTTRYDIDMTKCIYCGFCQEACPVDAVVEGP
NFEFATETREELIYDKAKLLENGDKWERAIAANLAADAPYR
>2[4Fe-4S]ST12(spmi:K663_10890) Sphingobium sp. MI1205
MSLGYYVKSFTLWEFVKAHWLTLKYFFKPKATINYPYEKNPISPRFRGEHALRRYPNGEE
RCIACKLCEAICPAQAITIEAQPREDGSRRTTRYDIDMTKCIYCGFCQEACPVDAVVEGP
NFEFATETREELIYDKAKLLENGDKWERAIAANLAADAPYR
>2[4Fe-4S]ST12(sphb:EP837_02368) Sphingobium sp. EP60837
MSLGYYVKSFTLWEFVKAHWLTLKYFFKPKATINYPYEKNPISPRFRGEHALRRYPNGEE
RCIACKLCEAICPAQAITIEAQPRDDGSRRTTRYDIDMTKCIYCGFCQEACPVDAIVEGP
NFEFATETREELIYDKAKLLENGDKWERAIAANLAADAPYR
>2[4Fe-4S]ST12(sphr:BSY17_159) Sphingobium sp. RAC03
MSLGYYVKSFTLWEFVKAHALTLKYFFKPKATINYPYEKNPISPRFRGEHALRRYPNGEE
RCIACKLCEAVCPAQAITIEAQPRDDGSRRTTRYDIDMTKCIYCGFCQEACPVDAVVEGP
NFEFATETREELIYDKGKLLENGDKWERAIAANLAADAPYR
>2[4Fe-4S]ST12(sinb:SIDU_07645) Sphingobium indicum
MSLGYYVKSFTLWEFVKAHALTLKYFFKPKATINYPYEKNPISPRFRGEHALRRYPNGEE

RCIACKLCEAICPAQAITIEAQPRDDGSRRTTRYDIDMTKCIYCGFCQEACPVDAIVEGP
NFEFSTETREELIYDKAKLLENGDKWERAIAANLAADAPYR
>2[4Fe-4S]ST12(gdi:GDI3032) Gluconacetobacter diazotrophicus PA1 5 (Brazil)
MGHFDRTVRSFLLAELVAGMGATFKAMFKPKVTINYPYEKGPLSPRFRGEHALRRYPNGE
ERCIACKLCEATCPAEAITIESEPRDDGSRRTTRYDIDMTKCIYCGLCEEACPVDAIVEG
PNYEFATETREELMYNKDKLLANGDRWESVLARRLELDAPYR
>2[4Fe-4S]ST12(gdj:Gdia_3336) Gluconacetobacter diazotrophicus PA1 5 (JGI)
MGHFDRTVRSFLLAELVAGMGATFKAMFKPKVTINYPYEKGPLSPRFRGEHALRRYPNGE
ERCIACKLCEATCPAEAITIESEPRDDGSRRTTRYDIDMTKCIYCGLCEEACPVDAIVEG
PNYEFATETREELMYNKDKLLANGDRWESVLARRLELDAPYR
>2[4Fe-4S]ST12(aace:A0U92_11150) Acetobacter aceti
MAGMDRALKAFLLTEIISGMASTFKTMFKPKVTINYPYEKGPLSPRFRGEHALRRYPNGE
ERCIACKLCEATCPAEAIVIEAEPRDDGSRRTTRYDIDMTKCIYCGLCEEACPVDAIVEG
PNYEFATETREELMYNKEKLLSNGDRWESVLARRLELDAPYR
>2[4Fe-4S]ST12(mgy:MGMSRv2__1319) Magnetospirillum gryphiswaldense MSR-1 v2
MAFLDRTARAFLLTELVQGLALTFRYMFKPSVTTNYPYEKIPMSPRFRGEHALRRYANGE
ERCIACKLCEAICPAQAITIEAEPRPDGSRRARRYDLDMTKCIYCGLCQEACPVDAIVEG
PNFEFATETRAELMYNKQKLLANGDRWEAELAMRIAADAPYR
>2[4Fe-4S]ST12(azl:AZL_021400) Azospirillum sp. B510
MAFLDRAARSLFLTELVCGMALTFRYMFKPKVTINYPYEKGPISSRFRGEHVLRRYASGE
ERCIACKLCEAVCPALAITIEAEPRDDGSRRTTRYDIDMTKCIYCGLCQEACPVDAVVMG
PNFEFSTENREELFYNKQKLLANGDRWEAEIAQNLAAEAPYR
>2[4Fe-4S]ST12(abs:AZOBR_100251) Azospirillum brasilense Sp245
MAFLDRAARSLFLTELLGGLGLTLRYMFKPKVTLNYPFEKGPISPRFRGEHVLRRYPGGE
ERCIACKLCEAVCPALAITIEAEPREDGSRRTTRYDIDMTKCIYCGYCQEACPVDAIVMG
PNFEFSTENREELFYNKQKLLANGDRWEAEIAQNLAAEAPYR
>2[4Fe-4S]ST12(abq:ABAZ39_08350) Azospirillum brasilense Az39
MAFLDRAARSLFLTELLGGLGLTLRYMFKPKVTLNYPFEKGPISPRFRGEHVLRRYPGGE
ERCIACKLCEAVCPALAITIEAEPREDGSRRTTRYDIDMTKCIYCGYCQEACPVDAIVMG
PNFEFSTENREELFYNKQKLLANGDRWEAEIAQNLAAEAPYR
>2[4Fe-4S]ST12(tmo:TMO_1970) Tistrella mobilis
MASIGQTARAFLLTEIVKGMALTLRYFFKPKVTLNYPYEKGPISPRFRGEHVLRRYPNGE
ERCIACKLCEAICPALAITIEAEPRADGSRRTTRYDIDMTKCIYCGFCQEACPVDAIVEG
PNFEFATETREELFYDKARLLANGDRWEREIAANLADVAPYR
>2[4Fe-4S]ST12(magq:MGMAQ_1973) Magnetospira sp. QH-2
MGFIDRTARAFLLAELVKGMALTFSYMFKPCVTLNYPMEKGPLSPRFRGEHALRRYPGGE
ERCIACKLCEAICPALAITIEAEPRDDGSRRTSRYDIDMTKCIYCGFCQEACPVDAIVEG
PNFEYATETREELFYNKDKLLANGDRWETELAARIRADAPYR
>2[4Fe-4S]ST12(apb:SAR116_0149) Candidatus Puniceispirillum marinum

MRMLMDSIKSFLLLELAKGMFLTLKYFFKPKVTINYPYEKGSLSPRFRGEHALRRYPNGE
ERCIACKLCEAVCPAQAITIEAEPRDDGSRRTTRYDIDMTKCIYCGFCQEACPVDAIVEG
PNFEFATETREELYYDKNKLLANGDRWETEIARNLAADAEWR

Subtype 13

>2[4Fe-4S]ST13(gdj:Gdia_0711) Gluconacetobacter diazotrophicus PA1 5 (JGI)

MFDILRTLWLTFRHMTHRRVTVGYPETKPPLPPRYRGRIILSRDPDGQERCVACGLCAVACPVDCISLQKTEQDGRWYPDYFRINFSRCIFCGFCEEACPTYAIQLTPDFEMSEYV

RPSL

VYEKEDLLISGTGKYPDYSFYLVSGVTIPGKDKGEAERESPPVDVRSLMP

>2[4Fe-4S]ST13(gdi:GDI2464) Gluconacetobacter diazotrophicus PA1 5 (Brazil)

MGYPETKPPLPPRYRGRIILSRDPDGQERCVACGLCAVACPVDCISLQKTEQDGRWYPDYFRINFSRCIFCGFCEEACPTYAIQLTPDFEMSEYVRPSLVYEKEDLLISGTGKYPD

YSFY

LVSGVTIPGKDKGEAERESPPVDVRSLMP

Subtype 14

>2[4Fe-4S]ST14(rpb:RPB_1479) Rhodopseudomonas palustris HaA2

MPLASYETSVPVVVDEAKCIADKGCTVCVDVCPLDVLRISDLTGKAFMAYDECWYCMPCEADCPTDAVKVNIPYLLR

>2[4Fe-4S]ST14(nwi:Nwi_0677) Nitrobacter winogradskyi

MPIAHTPTTVPVVVDEAKCIADKGCTVCVDVCPLDVLRISDLTGKAYMKYDECWYCMPCE

TDCPTGAVTVNIPYLLR

>2[4Fe-4S]ST14(bja:bsr3197) Bradyrhizobium diazoefficiens USDA 110

MPLASYQTSVPVVVDDAKCIADKGCTVCVDVCPLDVLRISEMTNKAYMAYDECWYCMPCE
ADCPTGAVTVNIPYLLR

>2[4Fe-4S]ST14(mlo:msr9193) Mesorhizobium japonicum MAFF 303099

MPLALSPTAVPVVVDETKCIADKGCTVCVDVCPLDVLRISDLTGKAYMKFDECWYCMPCE
KDCPTGAVTVSIPYLLR

>2[4Fe-4S]ST14(six:BSY16_4737) Sinorhizobium sp. RAC02

MPLAFSPTAVPVVVDDAKCIADKGCTVCVDVCPLDVLRISDLTGKAYMKFDECWYCMPCE
TDCPTGAVSVNIPYLLR

>2[4Fe-4S]ST14(same:SAMCFNEI73_pB0273) Sinorhizobium Americanum

MPLALSPTTVPVVVDDAKCIADKGCTVCVDVCPLDVLRISDLTGKAYMKFDECWYCMPCE
TDCPTGAVTVNIPYLLR

>2[4Fe-4S]ST14(same:SAMCFNEI73_pC1417) Sinorhizobium Americanum

MPLALSPTTVPVVVDDAKCIADKGCTVCVDVCPLDVLRISDLTGKAFMKFDECWYCMPCE
TDCPTGAVTVNIPYLLR

>2[4Fe-4S]ST14(xau:Xaut_2802) Xanthobacter autotrophicus

MPFANHPTSVPVTVDDEKCIAEKGCRVCIDVCPLDVLAINPASGKAHMKYDECWYCMPCE
VDCPTGAVKVEIPYLLR

>2[4Fe-4S]ST14(mex:Mext_3350) Methylorubrum extorquens PA1

MPLAHQPSAVPVTVDPEKCIADKGCTVCVDVCPLDVLRISDLTGKAHMKYDECWYCMPCE
TDCPTGAVTVQIPYLLR

>2[4Fe-4S]ST14(mea:Mex_1p3569) Methylorubrum extorquens AM1

MPLAHQPSAVPVTVDPEKCIADKGCTVCVDVCPLDVLRISDLTGKAHMKYDECWYCMPCE
TDCPTGAVTVQIPYLLR

>2[4Fe-4S]ST14(mdi:METDI4145) Methylorubrum extorquens DM4

MPLAHQPSTVPVTVDPEKCIADKGCTVCVDVCPLDVLRISDLTGKAYMKYDECWYCMPCE
TDCPTGAVTVQIPYLLR

>2[4Fe-4S]ST14(mch:Mchl_3675) Methylorubrum extorquens CM4

MPLAHQPSAVPVTVDPEKCIADKGCTVCVDVCPLDVLRISDLTGKAHMKYDECWYCMPCE
TDCPTGAVTVQIPYLLR

>2[4Fe-4S]ST14(mch:Mchl_4197) Methylorubrum extorquens CM4

MPIITQAKSAAVVIDDDKCIAEKGCRVCVDVCPLDILAIDETRQKAYMKYDECWYCMPCE
VDCPTNAVKVNIPYLLR

>2[4Fe-4S]ST14(mpo:Mpop_4190) Methylorubrum populi

MPLAHQPSTVPVTVDAEKCIADKGCTVCVDVCPLDVLRISDLTGKAHMKYDECWYCMPCE
TDCPTGAVTVQIPYLLR

>2[4Fe-4S]ST14(bjp:RN69_31775) Bradyrhizobium japonicum E109

MPLASYQTSVPVVVDDAKCIADKGCTVCVDVCPLDVLRISDMTGKAYMAYDECWYCMPCE
ADCPTGAVTVNIPYLLR

>2[4Fe-4S]ST14(bbt:BBta_4883) Bradyrhizobium sp. BTAi1

MPIAYTPTTLPVVVDEAKCIADKGCTVCVDVCPLDVLRISDLTGKAYMKYDECWYCMPCE
TDCPTGAVTVNIPYLLR

>2[4Fe-4S]ST14(brs:S23_48000) Bradyrhizobium sp. S23321

MPLASYQTSVPVVVDDAKCIADKGCTVCVDVCPLDVLRISDMTGKAYMAYDECWYCMPCE
ADCPTGAVTVNIPYLLR

>2[4Fe-4S]ST14(aol:S58_21790) Bradyrhizobium oligotrophicum

MPLATYQTSVPVVVDDAKCIADKGCTVCVDVCPLDVLRISDMTGKAFMAYDECWYCMPCE
ADCPTGAVTVNIPYLLR

>2[4Fe-4S]ST14(brad:BF49_1488) Bradyrhizobium sp. BF49

MPLASYQTSVPVVVDDAKCIADKGCTVCVDVCPLDVLRISDMTNKAYMAYDECWYCMPCE
ADCPTGAVTVNIPYLLR

>2[4Fe-4S]ST14(bic:LMTR13_09210) Bradyrhizobium icense

MPLASYQTSVPVVVDDAKCIADKGCTVCVDVCPLDVLRISDMTGKAYMAYDECWYCMPCE
ADCPTGAVTVNIPYLLR

>2[4Fe-4S]ST14(eah:FA04_29530) Ensifer adhaerens Casida A

MPLALSPTTVPVVVDEAKCIADKGCTVCVEVCPLDVLRISDLTGKAYMKFDECWYCMPCETDCPTGAVTVNIPYLLR

Subtype 15

>2[4Fe-4S]ST15(rsp:RSP_3193) Rhodobacter sphaeroides 2.4.1

MIEAAVSMSALGLGLGLLLGVAARRFHVESPPIVDAIEGILPGTNCGACGYPGCRGLAEAMSEGAAPVTACAPGGRDVALALAAIVETDGGGGAVPGMAEAEPTVAFIFEDHCTGC
MRCFKRCPTDAIIGANRQIHTVVTDACIGCNACIEACPTEAIVARVKPKTLKSWYWDKPRTAFEARGTEVAA

Subtype 16

>2[4Fe-4S]ST16(red:roselon_00015) Roseibacterium elongatum This one is from group 1A

MKGILDSLVNVGRKLVTKKYTRQYPEEKPDLPARYRARIVLTRDPDGQERCVACNLCAVACPVDCIDVVKAETEDGRWYPETFRINFARCIFCGYCEEACPTQAIQLTPDFETADY
TRAGLIHDKEDLLIAGQGKQEGYRYWSVAGKAIDGKPKGAAETEAAPINLKDLTP

Subtype 17

>2[4Fe-4S]ST17(pgv:SL003B_2077) Polymorphum gilvum
MGLDQAAKSLFLKEFVSAFFLAMRYFFKPKPTLNYPFEKGPVSPRFRGEHALRRYPNGEE
RCIACKLCEAICPAQAITIEAGPRRNDGTRRTTRYDIDMVKCIYCGFCQEACPVDAIVEG
PNFEFATETREELYYSKEKLLANGDRWEREIARNIEMDAPYR
>2[4Fe-4S]ST17(meso:BSQ44_12500) Mesorhizobium sp. B7
MSALAQAAKSLLLKEFFGAVVLSMRQFFAPKYTINYPHEKNPQSPRFRGEHALRRYPNGE
ERCIACKLCEAICPAQAITIEAGPRRNDGTRRTVRYDIDMVKCIYCGFCQEACPVDAIVE
GPNFEFSTETREELYYDKDRLLANGDRWEREIARNIALDSPYR
>2[4Fe-4S]ST17(mamo:A6B35_22200) Mesorhizobium amorphae
MSALSQAAKSLLLQDFVSAFFLSMRQFFAPKETINYPHEKGPISPRFRGEHALRRYPNGE
ERCIACKLCEAICPAQAITIEAGPRRNDGTRRTVRYDIDMVKCIYCGFCQEACPVDAIVE
GPNFEFATETREELYYDKDRLLANGDRWERELARNIALDSPYR
>2[4Fe-4S]ST17(rhl:LPU83_1663) Rhizobium favelukesii
MAGLSNAVSSLFLKEFVSAFWLTFRYIFKQKATINYPFEKGPVSPRFRGEHALRRYPNGE
ERCIACKLCEAICPAQAITIEAGPRRNDGTRRTVRYDIDMVKCIYCGFCQEACPVDAIVE
GPNFEFATETREELYFDKARLLENGDRWEREIARNIALDAPYR
>2[4Fe-4S]ST17(mlo:mll1359) Mesorhizobium japonicum MAFF 303099
MMSALSQAAKSLLLQDFVSAFFLSMRQFFAPKETINYPHEKGPVSPRFRGEHALRRYPNG
EERCIACKLCEAICPAQAITIEAGPRRNDGTRRTVRYDIDMVKCIYCGFCQEACPVDAIV
EGPNFEFATETREELYYDKDKLLANGDRWERELARNISLDAPYR
>2[4Fe-4S]ST17(mln:A9174_19200) Mesorhizobium loti NZP2037
MSALSQAAKSLLLQDFVSAFFLSMRQFFAPKETINYPHEKGPTSPRFRGEHALRRYPNGE
ERCIACKLCEAICPAQAITIEAGPRRNDGTRRTVRYDIDMVKCIYCGFCQEACPVDAIVE
GPNFEFATETREELYYDKDKLLANGDRWERELARNISLDAPYR
>2[4Fe-4S]ST17(mci:Mesci_3293) Mesorhizobium ciceri (biovar Biserrulae) mci
MSALSQAAKSLLLQDFVSAFFLSMRQFFAPKETINYPHEKGPTSPRFRGEHALRRYPNGE
ERCIACKLCEAICPAQAITIEAGPRRNDGTRRTVRYDIDMVKCIYCGFCQEACPVDAIVE
GPNFEFATETREELYYDKDRLLANGDRWERELARNISLDSPYR
>2[4Fe-4S]ST17(mop:Mesop_3690) Mesorhizobium opportunistum
MGALTQAAKSLLLQDFVSAFFLSMRQFFAPKETINYPHEKGPVSPRFRGEHALRRYPNGE
ERCIACKLCEAICPAQAITIEAGPRRNDGTRRTVRYDIDMVKCIYCGFCQEACPVDAIVE
GPNFEFATETREELYYDKDRLLANGDRWERELARNISLDSPYR
>2[4Fe-4S]ST17(mam:Mesau_03564) Mesorhizobium australicum
MSALSQAAKSLLLQDFVSAFFLSMRQFFAPKETINYPHEKGPISPRFRGEHALRRYPNGE
ERCIACKLCEAICPAQAITIEAGPRRNDGTRRTVRYDIDMVKCIYCGFCQEACPVDAIVE
GPNFEFATETREELYYDKDRLLANGDRWERELARNISLDSPYR
>2[4Fe-4S]ST17(mes:Meso_1030) Chelativorans sp. BNC1
MSALAQAAKSLLLKEFFGAVVLSMRQFFAPKATLNYPHEKGPLSPRFRGEHALRRYPNGE

ERCIACKLCEAICPAQAITIEAGPRRNDGTRRTVRYDIDMVKCIYCGFCQEACPVDAIVE
GPNFEFATETREELYYDKDKLLANGDRWERELARNIALDAPYR
>2[4Fe-4S]ST17(hoe:IMCC20628_01659) Hoeflea sp. IMCC20628
MLAQAAKSLFLKEFVGAFFLSMRYFFAPKATLNYPNEKGPVSPRFRGEHALRRYPNGEER
CIACKLCEAICPAQAITIEAGPRRNDGTRRTVRYDIDMVKCIYCGFCQEACPVDAIVEGP
NFEFATETREELYYDKDRLLANGDRWERELARNIAIDSPYR
>2[4Fe-4S]ST17(aak:AA2016_2936) Aminobacter aminovorans
MSALAQAAKSLLLQDFVGAFFLSMRQFFAPKATLNYPHEKGPVSPRFRGEHALRRYPNGE
ERCIACKLCEAICPAQAITIEAGPRRNDGTRRTVRYDIDMVKCIYCGFCQEACPVDAIVE
GPNFEFATETREELYYDKDKLLANGDRWEREIARNIAMDSPYR
>2[4Fe-4S]ST17(pla:Plav_3218) Parvibaculum lavamentivorans
MAWLDQSARSLFLAEFVSSFLLAMRYFFKPKVTLNYPFEKGPLSPRFRGEHALRRYPNGE
ERCIACKLCEAICPALAITIEAGPRRNDGTRRTTRYDIDMTKCIYCGLCQEACPVDAIVE
GPNFEFATETREELMYDKNRLLANGDRWEREIAKNIALDAPYR
>2[4Fe-4S]ST17(smk:Sinme_1071) Sinorhizobium meliloti AK83
MAGLSNAVSSLFLKEFVGAFLLSMRYFFRPKATLNYPFEKGPVSPRFRGEHALRRYPNGE
ERCIACKLCEAICPAQAITIEAGPRRNDGTRRTVRYDIDMVKCIYCGFCQEACPVDAIVE
GPNFEFSTETREELYYDKEKLLANGDRWEREIARNIAMDSPYR
>2[4Fe-4S]ST17(smq:SinmeB_0901) Sinorhizobium meliloti BL225C
MAGLSNAVSSLFLKEFVGAFLLSMRYFFRPKATLNYPFEKGPVSPRFRGEHALRRYPNGE
ERCIACKLCEAICPAQAITIEAGPRRNDGTRRTVRYDIDMVKCIYCGFCQEACPVDAIVE
GPNFEFSTETREELYYDKEKLLANGDRWEREIARNIAMDSPYR
>2[4Fe-4S]ST17(smx:SM11_chr2300) Sinorhizobium meliloti SM11
MAGLSNAVSSLFLKEFVGAFLLSMRYFFRPKATLNYPFEKGPVSPRFRGEHALRRYPNGE
ERCIACKLCEAICPAQAITIEAGPRRNDGTRRTVRYDIDMVKCIYCGFCQEACPVDAIVE
GPNFEFSTETREELYYDKEKLLANGDRWEREIARNIAMDSPYR
>2[4Fe-4S]ST17(smi:BN406_01009) Sinorhizobium meliloti Rm41
MAGLSNAVSSLFLKEFVGAFLLSMRYFFRPKATLNYPFEKGPVSPRFRGEHALRRYPNGE
ERCIACKLCEAICPAQAITIEAGPRRNDGTRRTVRYDIDMVKCIYCGFCQEACPVDAIVE
GPNFEFSTETREELYYDKEKLLANGDRWEREIARNIAMDSPYR
>2[4Fe-4S]ST17(smeg:C770_GR4Chr1290) Sinorhizobium meliloti GR4
MAGLSNAVSSLFLKEFVGAFLLSMRYFFRPKATLNYPFEKGPVSPRFRGEHALRRYPNGE
ERCIACKLCEAICPAQAITIEAGPRRNDGTRRTVRYDIDMVKCIYCGFCQEACPVDAIVE
GPNFEFSTETREELYYDKEKLLANGDRWEREIARNIAMDSPYR
>2[4Fe-4S]ST17(smel:SM2011_c01922) Sinorhizobium meliloti 2011
MMAGLSNAVSSLFLKEFVGAFLLSMRYFFRPKATLNYPFEKGPVSPRFRGEHALRRYPNG
EERCIACKLCEAICPAQAITIEAGPRRNDGTRRTVRYDIDMVKCIYCGFCQEACPVDAIV
EGPNFEFSTETREELYYDKEKLLANGDRWEREIARNIAMDSPYR
>2[4Fe-4S]ST17(smer:DU99_06640) Sinorhizobium meliloti RMO17

MAGLSNAVSSLFLKEFVGAFLLSMRYFFRPKATLNYPFEKGPVSPRFRGEHALRRYPNGE
ERCIACKLCEAICPAQAITIEAGPRRNDGTRRTVRYDIDMVKCIYCGFCQEACPVDAIVE
GPNFEFSTETREELYYDKEKLLANGDRWEREIARNIAMDSPYR
>2[4Fe-4S]ST17(smd:Smed_0898) Sinorhizobium medicae
MAGLSNAVSSLFLKEFVGAFLLSMRYFFRPKATLNYPYEKGPISPRFRGEHALRRYPNGE
ERCIACKLCEAICPAQAITIEAGPRRNDGTRRTVRYDIDMVKCIYCGFCQEACPVDAIVE
GPNFEFSTETREELYYDKEKLLANGDRWEREIARNIAMDSPYR
>2[4Fe-4S]ST17(rhi:NGR_c10580) Sinorhizobium fredii NGR234
MAGLSQAVSSLFLKEFVGAFFLSMRYFFRPKATLNYPFEKGPVSPRFRGEHALRRYPNGE
ERCIACKLCEAICPAQAITIEAGPRRNDGTRRTVRYDIDMVKCIYCGFCQEACPVDAIVE
GPNFEFATETREELYYDKEKLLANGDRWEREIARNIAMDSPYR
>2[4Fe-4S]ST17(sfh:SFHH103_00994) Sinorhizobium fredii HH103
MAGLSQAVSSLFLKEFVGAFFLSMRYFFRPKATLNYPFEKGPVSPRFRGEHALRRYPNGE
ERCIACKLCEAICPAQAITIEAGPRRNDGTRRTVRYDIDMVKCIYCGFCQEACPVDAIVE
GPNFEFATETREELYYDKEKLLANGDRWEREIARNIAMDSPYR
>2[4Fe-4S]ST17(sfd:USDA257_c33070) Sinorhizobium fredii USDA 257
MAGLSQAVSSLFLKEFVGAFFLSMRYFFRPKATLNYPFEKGPVSPRFRGEHALRRYPNGE
ERCIACKLCEAICPAQAITIEAGPRRNDGTRRTVRYDIDMVKCIYCGFCQEACPVDAIVE
GPNFEFATETREELYYDKEKLLANGDRWEREIARNIAMDSPYR
>2[4Fe-4S]ST17(six:BSY16_1542) Sinorhizobium sp. RAC02
MASLSQAVNSLFLKEFVGAFFLSMRYFFRPKATVNYPFEKGPVSPRFRGEHALRRYPNGE
ERCIACKLCEAICPAQAITIEAGPRRNDGTRRTVRYDIDMVKCIYCGFCQEACPVDAIVE
GPNFEFATETREELYYDKQKLLENGDRWEREIARNIAIDSPYR
>2[4Fe-4S]ST17(same:SAMCFNEI73_Ch1389) Sinorhizobium Americanum
MAGLSQAVGSLFLKEFVGAFLLSMRYFFRPKATVNYPFEKGPVSPRFRGEHALRRYPNGE
ERCIACKLCEAICPAQAITIEAGPRRNDGTRRTVRYDIDMVKCIYCGFCQEACPVDAIVE
GPNFEFATETREELYYDKEKLLANGDRWEREIARNIAMDSPYR
>2[4Fe-4S]ST17(ead:OV14_2323) Ensifer adhaerens OV14
MMAGLSQAVSSLFLKEFVGAFFLSMRYFFKPKATLNYPFEKGPVSPRFRGEHALRRYPNG
EERCIACKLCEAICPAQAITIEAGPRRNDGTRRTVRYDIDMVKCIYCGFCQEACPVDAIV
EGPNFEFATETREELYYDKDKLLANGDRWEREIARNIAMDSPYR
>2[4Fe-4S]ST17(eah:FA04_05060) Ensifer adhaerens Casida A
MAGLSQAVSSLFLKEFVGAFFLSMRYFFKPKATLNYPFEKGPVSPRFRGEHALRRYPNGE
ERCIACKLCEAICPAQAITIEAGPRRNDGTRRTVRYDIDMVKCIYCGFCQEACPVDAIVE
GPNFEFATETREELYYDKDKLLANGDRWEREIARNIAMDSPYR
>2[4Fe-4S]ST17(atu:Atu1278) Agrobacterium fabrum
MASLSQAVNSLFLKEFVGAIFLTMRHFFKQKATINYPFEKGPVSPRFRGEHALRRYPNGE
ERCIACKLCEAICPAQAITIEAGPRRNDGTRRTVRYDIDMVKCIYCGFCQEACPVDAIVE
GPNFEFATETREELYFDKQRLLDNGDRWEREIARNLALDAPYR

>2[4Fe-4S]ST17(ara:Arad_1857) Agrobacterium radiobacter
MKMAGFSNAVSSVFLKEFVGAFFLSMRYFFKQKATINYPFEKGPVSPRFRGEHALRRYPN
GEERCIACKLCEAICPAQAITIEAGPRRNDGTRRTVRYDIDMVKCIYCGFCQEACPVDAI
VEGPNFEFATETREELYFDKARLLENGDRWEREIARNIAIDSPYR
>2[4Fe-4S]ST17(atf:Ach5_11800) Agrobacterium tumefaciens Ach5
MASLSQAVNSLFLKEFVGAIFLTMRHFFKQKATVNYPFEKGPVSPRFRGEHALRRYPNGE
ERCIACKLCEAICPAQAITIEAGPRRNDGTRRTVRYDIDMVKCIYCGFCQEACPVDAIVE
GPNFEFATETREELYFDKQRLLDNGDRWEREIARNLALDAPYR
>2[4Fe-4S]ST17(ata:AWN88_11725) Agrobacterium tumefaciens S33
MASLSQAVNSLFLKEFVGAIFLTMRHFFKQKATINYPFEKGPVSPRFRGEHALRRYPNGE
ERCIACKLCEAICPAQAITIEAGPRRNDGTRRTVRYDIDMVKCIYCGFCQEACPVDAIVE
GPNFEFATETREELYFDKQKLLDNGDRWEREIARNLALDAPYR
>2[4Fe-4S]ST17(avi:Avi_1723) Agrobacterium vitis
MASVSQAVRSLFLKEFVNAFFLSMRYFFKQKATVNYPFEKGPVSPRFRGEHALRRYPNGE
ERCIACKLCEAICPAQAITIEAGPRRNDGTRRTVRYDIDMVKCIYCGFCQEACPVDAIVE
GPNFEFSTESREELYFDKAKLLANGDRWEREIARNLAQDAPYR
>2[4Fe-4S]ST17(agr:AGROH133_05642-1)Agrobacterium sp. H13-3
MASLSQAVNSLFLKEFVGAIFLTMRHFFKQKATVNYPFEKGPVSPRFRGEHALRRYPNGE
ERCIACKLCEAICPAQAITIEAGPRRNDGTRRTVRYDIDMVKCIYCGFCQEACPVDAIVE
GPNFEFATETREELYFDKQRLLDNGDRWEREIARNLALDAPYR
>2[4Fe-4S]ST17(agr:AGROH133_05642-2)Agrobacterium radiobacter
MASLSQAVNSLFLKEFVGAIFLTMRHFFKQKATVNYPFEKGPVSPRFRGEHALRRYPNGE
ERCIACKLCEAICPAQAITIEAGPRRNDGTRRTVRYDIDMVKCIYCGFCQEACPVDAIVE
GPNFEFATETREELYFDKQRLLDNGDRWEREIARNLALDAPYR
>2[4Fe-4S]ST17(agr:AGROH133_05642-3)Agrobacterium sp. RAC06
MASLSQAVNSLFLKEFVGAIFLTMRHFFKQKATVNYPFEKGPVSPRFRGEHALRRYPNGE
ERCIACKLCEAICPAQAITIEAGPRRNDGTRRTVRYDIDMVKCIYCGFCQEACPVDAIVE
GPNFEFATETREELYFDKQRLLDNGDRWEREIARNLALDAPYR
>2[4Fe-4S]ST17(aro:B0909_07015) Agrobacterium rhizogenes
MASLSQAVNSLFLKEFVGAILLTMRHFFKQKATVNYPFEKGPVSPRFRGEHALRRYPNGE
ERCIACKLCEAICPAQAITIEAGPRRNDGTRRTVRYDIDMVKCIYCGFCQEACPVDAIVE
GPNFEFATETREELYFDKQKLLDNGDRWEREIARNLALDAPYR
>2[4Fe-4S]ST17(ret:RHE_CH01613) Rhizobium etli CFN 42
MASLSSSINSLFLKEFFGAFFLSMRYFFRQKATINYPFEKGPVSPRFRGEHALRRYPNGE
ERCIACKLCEAICPAQAITIEAGPRRNDGTRRTVRYDIDMVKCIYCGFCQEACPVDAIVE
GPNFEFATETREELYFDKARLLDNGDRWEREIARNIAIDSPYR
>2[4Fe-4S]ST17(rec:RHECIAT_CH0001685) Rhizobium etli CIAT 652
MASLSSSINSLFLKEFFGAFFLSMRYFFRQKATINYPFEKGPVSPRFRGEHALRRYPNGE
ERCIACKLCEAICPAQAITIEAGPRRNDGTRRTVRYDIDMVKCIYCGFCQEACPVDAIVE

GPNFEFATETREELYFDKARLLDNGDRWEREIARNIAIDSPYR
>2[4Fe-4S]ST17(rel:REMIM1_CH01633) Rhizobium etli bv. mimosae Mim1
MASLSSSINSLFLKEFFGAFFLSMRYFFRQKATINYPFEKGPVSPRFRGEHALRRYPNGE
ERCIACKLCEAICPAQAITIEAGPRRNDGTRRTVRYDIDMVKCIYCGFCQEACPVDAIVE
GPNFEFATETREELYFDKARLLDNGDRWEREIARNIAIDSPYR
>2[4Fe-4S]ST17(rep:IE4803_CH01613) Rhizobium etli bv. phaseoli IE4803
MASLSSSINSLFLKEFFGAFFLSMRYFFRQKATINYPFEKGPVSPRFRGEHALRRYPNGE
ERCIACKLCEAICPAQAITIEAGPRRNDGTRRTVRYDIDMVKCIYCGFCQEACPVDAIVE
GPNFEFATETREELYFDKARLLDNGDRWEREIARNIAIDSPYR
>2[4Fe-4S]ST17(rei:IE4771_CH01666) Rhizobium sp. IE4771
MASLSSSINSLFLKEFFGAFFLSMRYFFRQKATINYPFEKGPVSPRFRGEHALRRYPNGE
ERCIACKLCEAICPAQAITIEAGPRRNDGTRRTVRYDIDMVKCIYCGFCQEACPVDAIVE
GPNFEFATETREELYFDKARLLDNGDRWEREIARNIAIDSPYR
>2[4Fe-4S]ST17(rle:RL1709) Rhizobium leguminosarum bv. viciae 3841
MASLSGSISSLFLKEFVGAFFLSMRYFFRQKATINYPFEKGPVSPRFRGEHALRRYPNGE
ERCIACKLCEAICPAQAITIEAGPRRNDGTRRTVRYDIDMVKCIYCGFCQEACPVDAIVE
GPNFEFATETREELYFDKARLLDNGDRWEREIARNIAIDSPYR
>2[4Fe-4S]ST17(rlt:Rleg2_1269) Rhizobium leguminosarum bv. trifolii WSM2304
MGSLSSSISSLFLKEFFGAFFLSMRYFFRQKATINYPFEKGPVSPRFRGEHALRRYPNGE
ERCIACKLCEAICPAQAITIEAGPRRNDGTRRTVRYDIDMVKCIYCGFCQEACPVDAIVE
GPNFEFATETREELYFDKARLLDNGDRWEREIARNIAIDSPYR
>2[4Fe-4S]ST17(rlg:Rleg_1361) Rhizobium leguminosarum bv. trifolii WSM2304
MASLSGSISSLFLKEFVGAFFLSMRYFFRQKATINYPFEKGPVSPRFRGEHALRRYPNGE
ERCIACKLCEAICPAQAITIEAGPRRNDGTRRTVRYDIDMVKCIYCGFCQEACPVDAIVE
GPNFEFATETREELYFDKARLLDNGDRWEREIARNIAIDSPYR
>2[4Fe-4S]ST17(rlb:RLEG3_17230) Rhizobium leguminosarum bv. trifolii WSM1689
MASLSGSISSLFLKEFVGAFFLSMRYFFRQKATINYPFEKGPVSPRFRGEHALRRYPNGE
ERCIACKLCEAICPAQAITIEAGPRRNDGTRRTVRYDIDMVKCIYCGFCQEACPVDAIVE
GPNFEFATETREELYFDKARLLDNGDRWEREIARNIAIDSPYR
>2[4Fe-4S]ST17(rlu:RLEG12_17425) Rhizobium leguminosarum bv. trifolii CB782
MGSLSSSINSLFLKEFFGAFFLSMRYFFRQKATINYPFEKGPVSPRFRGEHALRRYPNGE
ERCIACKLCEAICPAQAITIEAGPRRNDGTRRTVRYDIDMVKCIYCGFCQEACPVDAIVE
GPNFEFATETREELYFDKARLLDNGDRWEREIARNIAIDSPYR
>2[4Fe-4S]ST17(rtr:RTCIAT899_CH06930) Rhizobium tropici
MAGISNAVGSLFLKEFVGAFFLSMRYFFKQKATINYPFEKGPVSPRFRGEHALRRYPNGE
ERCIACKLCEAICPAQAITIEAGPRRNDGTRRTVRYDIDMVKCIYCGFCQEACPVDAIVE
GPNFEFATETREELYFDKARLLDNGDRWEREIAQNIAIDSPYR
>2[4Fe-4S]ST17(rir:BN877_I1259) Rhizobium sp. IRBG74
MASLSQAVNSLFLKEFVGAIFLTMRHFFKQKATINYPFEKGPVSPRFRGEHALRRYPNGE

ERCIACKLCEAICPAQAITIEAGPRRNDGTRRTVRYDIDMVKCIYCGFCQEACPVDAIVE
GPNFEFATETREELYFDKQKLLDNGDRWEREIARNLALDAPYR
>2[4Fe-4S]ST17(rga:RGR602_CH01474) Rhizobium gallicum
MASLSNAVGSLFLKEFVGAFFLSMRYFFRQKATINYPFEKGPVSPRFRGEHALRRYPNGE
ERCIACKLCEAICPAQAITIEAGPRRNDGTRRTVRYDIDMVKCIYCGFCQEACPVDAIVE
GPNFEFATETREELYFDKQRLLDNGDRWEREIARNMAIDAPYR
>2[4Fe-4S]ST17(rhn:AMJ98_CH01641) Rhizobium sp. N1341
MASLSSSINSLFLKEFFGAFFLSMRYFFRQKATINYPFEKGPVSPRFRGEHALRRYPNGE
ERCIACKLCEAICPAQAITIEAGPRRNDGTRRTVRYDIDMVKCIYCGFCQEACPVDAIVE
GPNFEFATETREELYFDKARLLDNGDRWEREIARNIAIDSPYR
>2[4Fe-4S]ST17(rpha:AMC79_CH01644) Rhizobium phaseoli
MASLSSSINSLFLKEFFGAFFLSMRYFFRQKATINYPFEKGPVSPRFRGEHALRRYPNGE
ERCIACKLCEAICPAQAITIEAGPRRNDGTRRTVRYDIDMVKCIYCGFCQEACPVDAIVE
GPNFEFATETREELYFDKARLLDNGDRWEREIARNIAIDSPYR
>2[4Fe-4S]ST17(rht:NT26_1274) Rhizobium sp. NT-26
MASLTQAVSSLFLKEFVGAFFLSMRYFFKPKATVNYPFEKNPVSPRFRGEHALRRYPNGE
ERCIACKLCEAICPAQAITIEAGPRRNDGTRRTVRYDIDMVKCIYCGFCQEACPVDAIVE
GPNFEFSTETREELYYDKARLLENGDRWEREIARNMAQDAPYR
>2[4Fe-4S]ST17(rhx:AMK02_CH01650) Rhizobium sp. N731
MASLSSSINSLFLKEFFGAFFLSMRYFFRQKATINYPFEKGPVSPRFRGEHALRRYPNGE
ERCIACKLCEAICPAQAITIEAGPRRNDGTRRTVRYDIDMVKCIYCGFCQEACPVDAIVE
GPNFEFATETREELYFDKARLLDNGDRWEREIARNIAIDSPYR
>2[4Fe-4S]ST17(ngl:RG1141_CH11880) Neorhizobium galegae bv. officinalis bv. officinalis HAMBI 1141
MASLGQAVNSLFLKEFVGAFFLSMRYFFKQKATINYPFEKGPVSPRFRGEHALRRYPNGE
ERCIACKLCEAICPAQAITIEAGPRRNDGTRRTVRYDIDMVKCIYCGFCQEACPVDAIVE
GPNFEFSTETREELYFDKARLLENGDRWEREIARNMAIDAPYR
>2[4Fe-4S]ST17(ngg:RG540_CH12150) Neorhizobium galegae bv. orientalis HAMBI 540
MASLGQAVNSLFLKEFVGAFFLSMRYFFKQKATINYPFEKGPVSPRFRGEHALRRYPNGE
ERCIACKLCEAICPAQAITIEAGPRRNDGTRRTVRYDIDMVKCIYCGFCQEACPVDAIVE
GPNFEFATETREELYFDKARLLENGDRWEREIARNMAIDAPYR
>2[4Fe-4S]ST17(lcc:B488_02650) Liberibacter crescens
MFIQSINSLFLKEFVRAFFLSMRYFFKPKATINYPFEKGPISSRFRGEHALRRYPNGEER
CIACKLCEAICPAQAITIEAGPRQSDGTRRTIRYDIDMVKCIYCGFCQEACPVDAIVEGP
NFEFATETREELYFDKARLLDNGDRWEKEIARNILIEAPYR
>2[4Fe-4S]ST17(shz:shn_07060) Shinella sp. HZN7
MASLSQAVNSLFLKEFVGAFFLSMRYFFRPKATVNYPFEKGPVSPRFRGEHALRRYPNGE
ERCIACKLCEAICPAQAITIEAGPRRNDGTRRTVRYDIDMVKCIYCGFCQEACPVDAIVE
GPNFEFSTETREELYYDKQKLLENGDRWEREIARNIAMDSPYR
>2[4Fe-4S]ST17(bme:BMEI1150) Brucella melitensis bv. 1 16M

MASIAQAAKSLLLKEFASAFALSMRQFFAPKATLNYPHEKGPVSPRFRGEHALRRYPNGE
ERCIACKLCEAICPAQAITIEAGPRRNDGTRRTVRYDIDMVKCIYCGFCQEACPVDAIVE
GPNFEFATETREELYYDKDKLLANGDHWEREIARNIAMDAPYR
>2[4Fe-4S]ST17(bmel:DK63_263) Brucella melitensis bv. 1 16M
MASIAQAAKSLLLKEFASAFALSMRQFFAPKATLNYPHEKGPVSPRFRGEHALRRYPNGE
ERCIACKLCEAICPAQAITIEAGPRRNDGTRRTVRYDIDMVKCIYCGFCQEACPVDAIVE
GPNFEFATETREELYYDKDKLLANGDHWEREIARNIAMDAPYR
>2[4Fe-4S]ST17(bmi:BMEA_A0852) Brucella melitensis ATCC 23457
MASIAQAAKSLLLKEFASAFALSMRQFFAPKATLNYPHEKGPVSPRFRGEHALRRYPNGE
ERCIACKLCEAICPAQAITIEAGPRRNDGTRRTVRYDIDMVKCIYCGFCQEACPVDAIVE
GPNFEFATETREELYYDKDKLLANGDRWEREIARNIAMDAPYR
>2[4Fe-4S]ST17(bmz:BM28_A0822) Brucella melitensis M28
MASIAQAAKSLLLKEFASAFALSMRQFFAPKATLNYPHEKGPVSPRFRGEHALRRYPNGE
ERCIACKLCEAICPAQAITIEAGPRRNDGTRRTVRYDIDMVKCIYCGFCQEACPVDAIVE
GPNFEFATETREELYYDKDKLLANGDRWEREIARNIAMDAPYR
>2[4Fe-4S]ST17(bmg:BM590_A0820) Brucella melitensis M5-90
MASIAQAAKSLLLKEFASAFALSMRQFFAPKATLNYPHEKGPVSPRFRGEHALRRYPNGE
ERCIACKLCEAICPAQAITIEAGPRRNDGTRRTVRYDIDMVKCIYCGFCQEACPVDAIVE
GPNFEFATETREELYYDKDKLLANGDRWEREIARNIAMDAPYR
>2[4Fe-4S]ST17(bmw:BMNI_I0802) Brucella melitensis NI
MASIAQAAKSLLLKEFASAFALSMRQFFAPKATLNYPHEKGPVSPRFRGEHALRRYPNGE
ERCIACKLCEAICPAQAITIEAGPRRNDGTRRTVRYDIDMVKCIYCGFCQEACPVDAIVE
GPNFEFATETREELYYDKDKLLANGDRWEREIARNIAMDAPYR
>2[4Fe-4S]ST17(bmee:DK62_596) Brucella melitensis bv. 3 Ether
MASIAQAAKSLLLKEFASAFALSMRQFFAPKATLNYPHEKGPVSPRFRGEHALRRYPNGE
ERCIACKLCEAICPAQAITIEAGPRRNDGTRRTVRYDIDMVKCIYCGFCQEACPVDAIVE
GPNFEFATETREELYYDKDKLLANGDRWEREIARNIAMDAPYR
>2[4Fe-4S]ST17(bmf:BAB1_0830) Brucella abortus 2308
MASIAQAAKSLLLKEFASAFALSMRQFFAPKATLNYPHEKGPVSPRFRGEHALRRYPNGE
ERCIACKLCEAICPAQAITIEAGPRRNDGTRRTVRYDIDMVKCIYCGFCQEACPVDAIVE
GPNFEFATETREELYYDKDKLLANGDRWEREIARNIAMDAPYR
>2[4Fe-4S]ST17(bmc:BAbS19_I07740A) Brucella abortus S19
MASIAQAAKSLLLKEFASAFALSMRQFFAPKATLNYPHEKGPVSPRFRGEHALRRYPNGE
ERCIACKLCEAICPAQAITIEAGPRRNDGTRRTVRYDIDMVKCIYCGFCQEACPVDAIVE
GPNFEFATETREELYYDKDKLLANGDRWEREIARNIAMDAPYR
>2[4Fe-4S]ST17(baa:BAA13334_I02677) Brucella abortus A13334
MASIAQAAKSLLLKEFASAFALSMRQFFAPKATLNYPHEKGPVSPRFRGEHALRRYPNGE
ERCIACKLCEAICPAQAITIEAGPRRNDGTRRTVRYDIDMVKCIYCGFCQEACPVDAIVE
GPNFEFATETREELYYDKDKLLANGDRWEREIARNIAMDAPYR

>2[4Fe-4S]ST17(babo:DK55_826) Brucella abortus bv. 2 86/8/59
MASIAQAAKSLLLKEFASAFALSMRQFFAPKATLNYPHEKGPVSPRFRGEHALRRYPNGE
ERCIACKLCEAICPAQAITIEAGPRRNDGTRRTVRYDIDMVKCIYCGFCQEACPVDAIVE
GPNFEFATETREELYYDKDKLLANGDRWEREIARNIAMDAPYR
>2[4Fe-4S]ST17(babr:DO74_1062) Brucella abortus bv. 6 870
MASIAQAAKSLLLKEFASAFALSMRQFFAPKATLNYPHEKGPVSPRFRGEHALRRYPNGE
ERCIACKLCEAICPAQAITIEAGPRRNDGTRRTVRYDIDMVKCIYCGFCQEACPVDAIVE
GPNFEFATETREELYYDKDKLLANGDRWEREIARNIAMDAPYR
>2[4Fe-4S]ST17(babt:DK49_585) Brucella abortus 63 75
MASIAQAAKSLLLKEFASAFALSMRQFFAPKATLNYPHEKGPVSPRFRGEHALRRYPNGE
ERCIACKLCEAICPAQAITIEAGPRRNDGTRRTVRYDIDMVKCIYCGFCQEACPVDAIVE
GPNFEFATETREELYYDKDKLLANGDRWEREIARNIAMDAPYR
>2[4Fe-4S]ST17(babb:DK48_1292) Brucella abortus BDW
MASIAQAAKSLLLKEFASAFALSMRQFFAPKATLNYPHEKGPVSPRFRGEHALRRYPNGE
ERCIACKLCEAICPAQAITIEAGPRRNDGTRRTVRYDIDMVKCIYCGFCQEACPVDAIVE
GPNFEFATETREELYYDKDKLLANGDRWEREIARNIAMDAPYR
>2[4Fe-4S]ST17(babu:DK53_811) Brucella abortus bv. 9 C68
MASIAQAAKSLLLKEFASAFALSMRQFFAPKATLNYPHEKGPVSPRFRGEHALRRYPNGE
ERCIACKLCEAICPAQAITIEAGPRRNDGTRRTVRYDIDMVKCIYCGFCQEACPVDAIVE
GPNFEFATETREELYYDKDKLLANGDRWEREIARNIAMDAPYR
>2[4Fe-4S]ST17(babs:DK51_647) Brucella abortus BER
MASIAQAAKSLLLKEFASAFALSMRQFFAPKATLNYPHEKGPVSPRFRGEHALRRYPNGE
ERCIACKLCEAICPAQAITIEAGPRRNDGTRRTVRYDIDMVKCIYCGFCQEACPVDAIVE
GPNFEFATETREELYYDKDKLLANGDRWEREIARNIAMDAPYR
>2[4Fe-4S]ST17(babc:DO78_732) Brucella abortus NCTC 10505
MASIAQAAKSLLLKEFASAFALSMRQFFAPKATLNYPHEKGPVSPRFRGEHALRRYPNGE
ERCIACKLCEAICPAQAITIEAGPRRNDGTRRTVRYDIDMVKCIYCGFCQEACPVDAIVE
GPNFEFATETREELYYDKDKLLANGDRWEREIARNIAMDAPYR
>2[4Fe-4S]ST17(bsui:BSSP1_I0894) Brucella suis bv. 2 Bs143CITA
MASITQAAKSLLLKEFASAFALSMRQFFAPKATLNYPHEKGPVSPRFRGEHALRRYPNGE
ERCIACKLCEAICPAQAITIEAGPRRNDGTRRTVRYDIDMVKCIYCGFCQEACPVDAIVE
GPNFEFATETREELYYDKDKLLANGDRWEREIARNIAMDAPYR
>2[4Fe-4S]ST17(bsup:BSPT1_I0906) Brucella suis bv. 2 PT09143
MASITQAAKSLLLKEFASAFALSMRQFFAPKATLNYPHEKGPVSPRFRGEHALRRYPNGE
ERCIACKLCEAICPAQAITIEAGPRRNDGTRRTVRYDIDMVKCIYCGFCQEACPVDAIVE
GPNFEFATETREELYYDKDKLLANGDRWEREIARNIAMDAPYR
>2[4Fe-4S]ST17(bsuv:BSPT2_I0893) Brucella suis bv. 2 PT09172
MASITQAAKSLLLKEFASAFALSMRQFFAPKATLNYPHEKGPVSPRFRGEHALRRYPNGE
ERCIACKLCEAICPAQAITIEAGPRRNDGTRRTVRYDIDMVKCIYCGFCQEACPVDAIVE

GPNFEFATETREELYYDKDKLLANGDRWEREIARNIAMDAPYR
>2[4Fe-4S]ST17(bsuc:BSSP2_I0822) Brucella suis bv. 2 Bs364CITA
MASITQAAKSLLLKEFASAFALSMRQFFAPKATLNYPHEKGPVSPRFRGEHALRRYPNGE
ERCIACKLCEAICPAQAITIEAGPRRNDGTRRTVRYDIDMVKCIYCGFCQEACPVDAIVE
GPNFEFATETREELYYDKDKLLANGDRWELEIARNIAMDAPYR
>2[4Fe-4S]ST17(bsz:DK67_1719) Brucella suis bv. 3
MASITQAAKSLLLKEFASAFALSMRQFFAPKATLNYPHEKGPVSPRFRGEHALRRYPNGE
ERCIACKLCEAICPAQAITIEAGPRRNDGTRRTVRYDIDMVKCIYCGFCQEACPVDAIVE
GPNFEFATETREELYYDKDKLLANGDRWEREIARNIAMDAPYR
>2[4Fe-4S]ST17(bsw:IY71_04060) Brucella suis ZW043
MASITQAAKSLLLKEFASAFALSMRQFFAPKATLNYPHEKGPVSPRFRGEHALRRYPNGE
ERCIACKLCEAICPAQAITIEAGPRRNDGTRRTVRYDIDMVKCIYCGFCQEACPVDAIVE
GPNFEFATETREELYYDKDKLLANGDRWEREIARNIAMDAPYR
>2[4Fe-4S]ST17(bsg:IY72_03790) Brucella suis ZW046
MASITQAAKSLLLKEFASAFALSMRQFFAPKATLNYPHEKGPVSPRFRGEHALRRYPNGE
ERCIACKLCEAICPAQAITIEAGPRRNDGTRRTVRYDIDMVKCIYCGFCQEACPVDAIVE
GPNFEFATETREELYYDKDKLLANGDRWEREIARNIAMDAPYR
>2[4Fe-4S]ST17(bcs:BCAN_A0825) Brucella canis ATCC 23365
MASITQAAKSLLLKEFASAFALSMRQFFAPKATLNYPHEKGPVSPRFRGEHALRRYPNGE
ERCIACKLCEAICPAQAITIEAGPRRNDGTRRTVRYDIDMVKCIYCGFCQEACPVDAIVE
GPNFEFATETREELYYDKDKLLANGDRWEREIARNIAMDAPYR
>2[4Fe-4S]ST17(bsk:BCA52141_I0434) Brucella canis HSK A52141
MASITQAAKSLLLKEFASAFALSMRQFFAPKATLNYPHEKGPVSPRFRGEHALRRYPNGE
ERCIACKLCEAICPAQAITIEAGPRRNDGTRRTVRYDIDMVKCIYCGFCQEACPVDAIVE
GPNFEFATETREELYYDKDKLLANGDRWEREIARNIAMDAPYR
>2[4Fe-4S]ST17(bcar:DK60_874) Brucella canis RM6/66
MASITQAAKSLLLKEFASAFALSMRQFFAPKATLNYPHEKGPVSPRFRGEHALRRYPNGE
ERCIACKLCEAICPAQAITIEAGPRRNDGTRRTVRYDIDMVKCIYCGFCQEACPVDAIVE
GPNFEFATETREELYYDKDKLLANGDRWEREIARNIAMDAPYR
>2[4Fe-4S]ST17(bcas:DA85_03860) Brucella canis SVA13
MASITQAAKSLLLKEFASAFALSMRQFFAPKATLNYPHEKGPVSPRFRGEHALRRYPNGE
ERCIACKLCEAICPAQAITIEAGPRRNDGTRRTVRYDIDMVKCIYCGFCQEACPVDAIVE
GPNFEFATETREELYYDKDKLLANGDRWEREIARNIAMDAPYR
>2[4Fe-4S]ST17(bpv:DK65_555) Brucella pinnipedialis 6/566
MASIAQAAKSLLLKEFASAFALSMRQFFAPKATLNYPHEKGPVSPRFRGEHALRRYPNGE
ERCIACKLCEAICPAQAITIEAGPRRNDGTRRTVRYDIDMVKCIYCGFCQEACPVDAIVE
GPNFEFATETREELYYDKDKLLANGDRWEREIARNIAMDAPYR
>2[4Fe-4S]ST17(bcet:V910_101165) Brucella ceti TE10759-12
MASIAQAAKSLLLKEFASAFALSMRQFFAPKATLNYPHEKGPVSPRFRGEHALRRYPNGE

ERCIACKLCEAICPAQAITIEAGPRRNDGTRRTVRYDIDMVKCIYCGFCQEACPVDAIVE
GPNFEFATETREELYYDKDKLLANGDRWEREIARNIAMDAPYR
>2[4Fe-4S]ST17(oan:Oant_2415) Ochrobactrum anthropi ATCC 49188
MASFAQAAKSLLLKEFVGAFFLSMRQFFAPKATLNYPHEKGPVSPRFRGEHALRRYPNGE
ERCIACKLCEAICPAQAITIEAGPRRNDGTRRTVRYDIDMVKCIYCGFCQEACPVDAIVE
GPNFEFATETREELYYDKDKLLANGDRWEREIARNIAMDAPYR
>2[4Fe-4S]ST17(oah:DR92_1876) Ochrobactrum anthropi OAB
MASFAQAAKSLLLKEFVGAFFLSMRQFFAPKATLNYPHEKGPVSPRFRGEHALRRYPNGE
ERCIACKLCEAICPAQAITIEAGPRRNDGTRRTVRYDIDMVKCIYCGFCQEACPVDAIVE
GPNFEFATETREELYYDKDKLLANGDRWEREIARNIAMDAPYR
>2[4Fe-4S]ST17(ops:A8A54_03620) Ochrobactrum pseudogrignonense
MASFAQAAKSLLLKEFVGAFFLSMRQFFAPKATLNYPHEKGPVSPRFRGEHALRRYPNGE
ERCIACKLCEAICPAQAITIEAGPRRNDGTRRTVRYDIDMVKCIYCGFCQEACPVDAIVE
GPNFEFATETREELYYDKDKLLANGDRWEREIARNIAMDAPYR
>2[4Fe-4S]ST17(bja:bll4909) Bradyrhizobium diazoefficiens USDA 110
MRAVMGININATARSLLLSEFVSAFFLAMRYFFQPKPTLNYPFEKGPISPRFRGEHALRR
YPNGEERCIACKLCEAVCPAQAITIEAGPRRNDGTRRTVRYDIDMVKCIYCGLCQEACPV
DAIVEGPNFEFATETREELFYDKAKLLANGDRWEREIAKAIELDAPYR
>2[4Fe-4S]ST17(bju:BJ6T_47790) Bradyrhizobium japonicum USDA 6
MGINVNATARSLLLSEFVSAFFLAMRYFFQPKPTLNYPFEKGPISPRFRGEHALRRYPNG
EERCIACKLCEAICPAQAITIEAGPRRNDGTRRTVRYDIDMVKCIYCGLCQEACPVDAIV
EGPNFEFATETREELFYDKAKLLANGDRWEREIAKAIELDAPYR
>2[4Fe-4S]ST17(bjp:RN69_23295) Bradyrhizobium japonicum E109
MNVNATARSLLLSEFVSAFFLAMRYFFQPKPTLNYPFEKGPISPRFRGEHALRRYPNGEE
RCIACKLCEAICPAQAITIEAGPRRNDGTRRTVRYDIDMVKCIYCGLCQEACPVDAIVEG
PNFEFATETREELFYDKAKLLANGDRWEREIAKAIELDAPYR
>2[4Fe-4S]ST17(bra:BRADO4176) Bradyrhizobium sp. ORS 278
MNISATARSLLLQEFVSAFFLAMRYFFKPKPTLNYPFEKGPISPRFRGEHALRRYPNGEE
RCIACKLCEAVCPAQAITIEAGPRRNDGTRRTVRYDIDMVKCIYCGLCQEACPVDAIVEG
PNFEFATETREELYYDKAKLLANGDRWEREISKAIALDAPYR
>2[4Fe-4S]ST17(bbt:BBta_4553) Bradyrhizobium sp. BTAi1
MNISATARSLLLSEFVSAFFLAMRYFFQPKPTLNYPFEKGPISPRFRGEHALRRYPNGEE
RCIACKLCEAVCPAQAITIEAGPRRNDGTRRTVRYDIDMVKCIYCGLCQEACPVDAIVEG
PNFEFATETREELYYDKAKLLANGDRWEREISKAIALDAPYR
>2[4Fe-4S]ST17(brs:S23_32720) Bradyrhizobium sp. S23321
MGININATARSLLLSEFVSAFFLAMRYFFQPKPTLNYPFEKGPISPRFRGEHALRRYPNG
EERCIACKLCEAVCPAQAITIEAGPRRNDGTRRTVRYDIDMVKCIYCGLCQEACPVDAIV
EGPNFEFATETREELFYDKAKLLANGDRWEREIAKAIELDAPYR
>2[4Fe-4S]ST17(aol:S58_34180) Bradyrhizobium oligotrophicum

MNISATARSLLLQEFVSAFFLAMRYFFQPKPTLNYPFEKGPISPRFRGEHALRRYPNGEE
RCIACKLCEAVCPAQAITIEAGPRRNDGTRRTVRYDIDMVKCIYCGLCQEACPVDAIVEG
PNFEFATETREELYYDKAKLLANGDRWEREISKAIALDAPYR
>2[4Fe-4S]ST17(brc:BCCGELA001_20840) Bradyrhizobium sp. CCGE-LA001
MNINATARSLLLSEFVSAFFLAMRYFFQPKPTLNYPFEKGPISPRFRGEHALRRYPNGEE
RCIACKLCEAVCPAQAITIEAGPRRNDGTRRTVRYDIDMVKCIYCGLCQEACPVDAIVEG
PNFEFATETREELFYDKAKLLANGDRWEREIAKAIELDAPYR
>2[4Fe-4S]ST17(brad:BF49_6647) Bradyrhizobium sp. BF49
MGININATARSLLLSEFVSAFFLAMRYFFQPKPTLNYPFEKGPISPRFRGEHALRRYPNG
EERCIACKLCEAVCPAQAITIEAGPRRNDGTRRTVRYDIDMVKCIYCGLCQEACPVDAIV
EGPNFEFATETREELFYDKAKLLANGDRWEREIAKAIELDAPYR
>2[4Fe-4S]ST17(bic:LMTR13_18775) Bradyrhizobium icense
MSVNVNATARALLLSEFVSAFFLAMRYFFKPKPTINYPFEKNPISPRFRGEHALRRYPNG
EERCIACKLCEAICPAQAITIEAGPRRNDGTRRTVRYDIDMVKCIYCGLCQEACPVDAIV
EGPNFEFATETREELYYDKAKLLANGDRWEREIAKAIELDAPYR
>2[4Fe-4S]ST17(rpb:RPB_2582) Rhodopseudomonas palustris HaA2
MNINATARSLLLTEFVSAFFLAMRYFFKPKPTINYPFEKNPISPRFRGEHALRRYPNGEE
RCIACKLCEAICPAQAITIEAGPRRNDGTRRTVRYDIDMVKCIYCGLCQEACPVDAIVEG
PNFEFATETREELYYDKARLLANGDRWEREIAKSIELDAPYR
>2[4Fe-4S]ST17(rpc:RPC_2412) Rhodopseudomonas palustris BisB18
MRVTAAARSLLLTEFVSAFFLAMRYFFKPKPTINYPFEKNPISPRFRGEHALRRYPNGEE
RCIACKLCEAVCPAQAITIEAGPRRNDGTRRTVRYDIDMVKCIYCGLCQEACPVDAIVEG
PNFEFATETREELYYDKAKLLANGDRWEREIAKSIELDAPYR
>2[4Fe-4S]ST17(rpd:RPD_2877) Rhodopseudomonas palustris BisB5
MNINATARSLLLTEFVSAFFLAMRYFFRPKPTINYPFEKNPISPRFRGEHALRRYPNGEE
RCIACKLCEAICPAQAITIEAGPRRNDGTRRTVRYDIDMVKCIYCGLCQEACPVDAIVEG
PNFEFATETREELYYDKARLLANGDRWEREIAKSIELDAPYR
>2[4Fe-4S]ST17(rpe:RPE_2531) Rhodopseudomonas palustris BisA53
MGVTTAARSLLLTEFVSAFFLAMRYFFKPKPTLNYPFEKGPISPRFRGEHALRRYPNGEE
RCIACKLCEAVCPAQAITIEAGPRRNDGTRRTERYDIDMVKCIYCGLCQEACPVDAIVEG
PNFEFATETREELYYDKAKLLANGDRWEREIAKHMALDAPYR
>2[4Fe-4S]ST17(rpt:Rpal_3290) Rhodopseudomonas palustris TIE-1
MNVTATARSLLLTEFVSAFFLAMRYFFKPKPTINYPFEKNPISPRFRGEHALRRYPNGEE
RCIACKLCEAVCPAQAITIEAGPRRNDGTRRTVRYDIDMVKCIYCGFCQEACPVDAIVEG
PNFEFATETREELYYDKARLLANGDRWEREIAKAISLDAPYR
>2[4Fe-4S]ST17(rpx:Rpdx1_2561) Rhodopseudomonas palustris DX-1
MNVTAAARSLLLTEFVSAFFLAMRYFFKPKPTINYPFEKNPISPRFRGEHALRRYPNGEE
RCIACKLCEAICPAQAITIEAGPRRNDGTRRTVRYDIDMVKCIYCGFCQEACPVDAIVEG
PNFEFATETREELYYDKARLLANGDRWEREIAKAISLDAPYR

```
>2[4Fe-4S]ST17(nwi:Nwi_1879 ) Nitrobacter winogradskyi
MSINATARSLLLTEFISAFFLTMRYFFKPKPTINYPFEKNPISPRFRGEHALRRYPNGEE
RCIACKLCEAVCPAQAITIEAGPRRNDGTRRTVRYDIDMVKCIYCGLCQEACPVDAIVEG
PNFEFATETREELYYDKAKLLANGDRWEREIAKAIELDAPYR
>2[4Fe-4S]ST17(nha:Nham_2212) Nitrobacter hamburgensis
MSINATARALLLTEFVSAFFLTMRYFFQPKATINYPFEKNPISPRFRGEHALRRYPNGEE
RCIACKLCEAICPAQAITIEAGPRRNDGTRRTVRYDIDMVKCIYCGLCQEACPVDAIVEG
PNFEFATETREELYYDKAKLLANGDRWEREIAKAIELDAPYR
>2[4Fe-4S]ST17(bos:BSY19_2477) Bosea sp. RAC05
MSLAQAAKGLLLKEFVGAFALSMRYFFKPKATINYPFEKNPQSPRFRGEHALRRYPNGEE
RCIACKLCEAICPAQAITIEAGPRRNDGTRRTTRYDIDMTKCIYCGFCQEACPVDAIVEG
PNFEFATETREELFYDKDRLLENGARWEREIARNIAMDAPYR
>2[4Fe-4S]ST17(bvv:BHK69_20555) Bosea vaviloviae
MSLAQAAKGLLLKEFVGAFALSMRYFFKPKATLNYPFEKGMLSPRFRGEHALRRYPNGEE
RCIACKLCEAICPAQAITIEAGPRRNDGTRRTTRYDIDMTKCIYCGFCQEACPVDAIVEG
PNFEFATETREELFYDKDRLLANGARWEREIARNIAMDAPYR
>2[4Fe-4S]ST17(xau:Xaut_4625) Xanthobacter autotrophicus
MKLDQAARGLFLTELVSGFFLAMRYFFKPKATLNYPFEKGPLSPRFRGEHALRRYPNGEE
RCIACKLCEAICPAQAITIEAGPRRNDGTRRTTRYDIDMVKCIYCGFCQEACPVDAIVEG
PNFEFAAETREELYYDKEKLLANGDRWEREIARSIAMDAPYR
>2[4Fe-4S]ST17(azc:AZC_1676) Azorhizobium caulinodans
MKLDQAARALFLTELVSGFFLAMRYFFKPKATINYPFEKNPISPRFRGEHALRRYPNGEE
RCIACKLCEAICPAQAITIEAGPRRNDGTRRTTRYDIDMVKCIYCGFCQEACPVDAIVEG
PNFEFATETREELYYDKEKLLANGDRWEREIARAIAADAPYR
>2[4Fe-4S]ST17(mex:Mext_1077) Methylorubrum extorquens PA1
MKLDQVARSLLLKEFVSGFVLAMKYFFKPKATINYPFEMGHRGPRFRGEHALRRYPNGEE
RCIACKLCEAICPAQAITIEAGPRRNDGTRRTTRYDIDMVKCIYCGMCQEACPVDAIVEG
PNFEFSVETREELLYDKQKLLENGDRWEREIARNIAVDAPYR
>2[4Fe-4S]ST17(mea:Mex_1p0847) Methylorubrum extorquens AM1
MKLDQVARSLLLKEFVSGFVLAMKYFFKPKATINYPFEMGHRGPRFRGEHALRRYPNGEE
RCIACKLCEAICPAQAITIEAGPRRNDGTRRTTRYDIDMVKCIYCGMCQEACPVDAIVEG
PNFEFSVETREELLYDKQKLLENGDRWEREIARNIAVDAPYR
>2[4Fe-4S]ST17(mdi:METDI1558) Methylorubrum extorquens DM4
MKLDQVARSLLLKEFVSGFVLAMKYFFKPKATINYPFEMGHRGPRFRGEHALRRYPNGEE
RCIACKLCEAICPAQAITIEAGPRRNDGTRRTTRYDIDMVKCIYCGMCQEACPVDAIVEG
PNFEFSVETREELLYDKQKLLENGDRWEREIARNIAVDAPYR
>2[4Fe-4S]ST17(mch:Mchl_1206) Methylorubrum extorquens CM4
MKLDQVARSLLLKEFVSGFVLAMKYFFKPKATINYPFEMGHRGPRFRGEHALRRYPNGEE
RCIACKLCEAICPAQAITIEAGPRRNDGTRRTTRYDIDMVKCIYCGMCQEACPVDAIVEG
```

PNFEFSVETREELLYDKQKLLENGDRWEREIARNIAVDAPYR
>2[4Fe-4S]ST17(mpo:Mpop_1011) Methylorubrum populi
MKLDQVARSLLLKEFVSGFFLAMKYFLKPKATINYPFEMGHRGPRFRGEHALRRYPNGEE
RCIACKLCEAICPAQAITIEAGPRRNDGTRRTTRYDIDMVKCIYCGMCQEACPVDAIVEG
PNFEFSVETREELLYDKQKLLENGDRWEREIARNIAIDAPYR
>2[4Fe-4S]ST17(mrd:Mrad2831_2051) Methylobacterium radiotolerans
MKLDQVAKSLLLKEFVSGMILGMRYFFKPKATINYPFEMGHRGPRFRGEHALRRYPNGEE
RCIACKLCEAICPAQAITIEAGPRRNDGTRRTTRYDIDMVKCIYCGMCQEACPVDAIVEG
PNFEFSVETREELLYDKQKLLLNGDRWEREIARNIAMDAPYR
>2[4Fe-4S]ST17(met:M446_4398) Methylobacterium sp. 4-46
MRLDQVARSLLLKEFVSGFALAMRYLFKPKATINYPFEMGHRSPRFRGEHALRRYPNGEE
RCIACKLCEAVCPAQAITIEAGPRRNDGTRRTTRYDIDMVKCIYCGMCQEACPVDAIVEG
PNFEFSVETREELLYDKQRLLANGDRWEREIARNIAADAPYR
>2[4Fe-4S]ST17(mno:Mnod_4134) Methylobacterium nodulans
MRLDQVARSLLLKEFVSGFALAMRYLFKPKATINYPFEMGHRSPRFRGEHALRRYPNGEE
RCIACKLCEAICPAQAITIEAGPRRNDGTRRTTRYDIDMVKCIYCGMCQEACPVDAIVEG
PNFEFSVETREELLYDKQKLLANGDRWEREIARNIAADAPYR
>2[4Fe-4S]ST17(mor:MOC_2440) Methylobacterium oryzae
MKLDQVAKSLLLKEFVSGMVLGMRYFFKPKATINYPFEMGHRGPRFRGEHALRRYPNGEE
RCIACKLCEAICPAQAITIEAGPRRNDGTRRTTRYDIDMVKCIYCGMCQEACPVDAIVEG
PNFEFSVETREELLYDKQKLLLNGDRWEREIARNIAMDAPYR
>2[4Fe-4S]ST17(meta:Y590_04475) Methylobacterium sp. AMS5
MKLDQVARSLLLKEFVSGFFLAMKYFFKPKATINYPFEMGHRGPRFRGEHALRRYPNGEE
RCIACKLCEAICPAQAITIEAGPRRNDGTRRTTRYDIDMVKCIYCGMCQEACPVDAIVEG
PNFEFSVETREELLYDKQKLLENGDRWEREIARNIAVDAPYR
>2[4Fe-4S]ST17(maqu:Maq22A_c02875) Methylobacterium aquaticum
MKLDQVARGLLLKEFVTGFALAMRYFFKPKATINYPFEMGHRGPRFRGEHALRRYPNGEE
RCIACKLCEAVCPAQAITIEAGPRRNDGTRRTTRYDIDMVKCIYCGMCQEACPVDAIVEG
PNFEFSVETREELLYDKEKLLANGDRWEREIARNIAADAPYR
>2[4Fe-4S]ST17(bid:Bind_2390 K00338 Beijerinckia indica
MRLDSAAKALFLKEFVGAFALSMRYFFKPRPTLNYPHEKNPQSPRYRGEHALRRYPNGEE
RCIACKLCEAICPAKAITIEAGPRRNDGTRRTTRYDIDMVKCIYCGFCQEACPVDAIVEG
PNAEFAVETREELYYDKDRLLQNGARWEREIARNIALDAPYR
>2[4Fe-4S]ST17(msl:Msil_2926) Methylocella silvestris
MKLDQAAKALFLSEFVGAFLLSMRYFFKPKPTLNYPHEKNPQSPRYRGEHALRRYPNGEE
RCIACKLCEAICPAQAITIEAGPRRNDGTRRTTRYDIDMVKCIYCGFCQEACPVDAIVEG
PNAEFSVETREELYYDKDRLLENGARWEREIARNIALDAPYR
>2[4Fe-4S]ST17(hdn:Hden_1934) Hyphomicrobium denitrificans ATCC 51888
MSVARAAKSLFLTEIVVAFVLTLRYFFAPKKTLNYPYEKGPLSPRFRGEHALRRYPNGEE

RCIACKLCEAICPAQAITIEAGPRRNDGTRRTTRYDIDMTKCIYCGLCQEACPVDAIVEG
PNFEFATETREELFYDKERLLSNGDRWEREIAKNIALDAKYR
>2[4Fe-4S]ST17(hdt:HYPDE_28883) Hyphomicrobium denitrificans 1NES1
MSVARATKSLLLTEIVAAFVLTVRYFFAPKKTLNYPYEKGPLSPRFRGEHALRRYPNGEE
RCIACKLCEAICPAQAITIEAGPRRNDGTRRTTRYDIDMTKCIYCGLCQEACPVDAIVEG
PNFEFATETREELFYDKERLLSNGDRWEREIAKNIALDAKYR
>2[4Fe-4S]ST17(hmc:HYPMC_2890) Hyphomicrobium sp. MC1
MNVAQGVKSLFLTEFVSAFFLTMRYFFSPKKTLNYPYEKGPLSPRFRGEHALRRYPNGEE
RCIACKLCEAICPAQAITIEAGPRRNDGTRRTTRYDIDMTKCIYCGLCQEACPVDAIVEG
PNFEFATETREELFYDKERLLSNGDRWEREIAKNIALDAKYR
>2[4Fe-4S]ST17(hni:W911_15990) Hyphomicrobium nitrativorans
MRVAQGLKSLFLAEFVSAFFLAMRYFFAPKKTLNYPFEKGPLSPRFRGEHALRRYPNGEE
RCIACKLCEAICPAQAITIEAGPRRNDGTRRTTRYDIDMVKCIYCGLCQEACPVDAIVEG
PNFEFATETREELYYDKERLLANGDRWEREIARNLALDAPYR
>2[4Fe-4S]ST17(phl:KKY_1323) Pelagibacterium halotolerans
MKALRFLDALLLREFVSTFFLAMRYFFAPKPTINYPFEKGEVSPRFRGEHALRRYPNGEE
RCIACKLCEAICPAQAITIEAGPRQNDGTRRTVRYDIDMVKCIYCGFCQEACPVDAIVEG
PNFEFATETREELYFSKDRLLANGDRWERELAANIALDAPYR
>2[4Fe-4S]ST17(fil:BN1229_v1_3009) Candidatus Filomicrobium marinum W
MAIAQAVRSMFLTEFVSAFFLAMRYFFMPKKTVNYPFEKGPLSPRFRGEHALRRYPNGQE
RCIACKLCEAICPALAITIEAGPRRNDGTRRTTRYDIDMTKCIYCGLCQEACPVEAIVEG
PNFEFATETREELFYDKERLLDNGDRWEREIAKNIGLDAKFR
>2[4Fe-4S]ST17(fiy:BN1229_v1_2907) Candidatus Filomicrobium marinum Y
MAIAQAVRSMFLTEFVSAFFLAMRYFFMPKKTVNYPFEKGPLSPRFRGEHALRRYPNGQE
RCIACKLCEAICPALAITIEAGPRRNDGTRRTTRYDIDMTKCIYCGLCQEACPVEAIVEG
PNFEFATETREELFYDKERLLDNGDRWEREIAKNIGLDAKFR
>2[4Fe-4S]ST17(deq:XM25_06955) Devosia sp. H5989
MRQFLNSLLLKEFVSAFALSMRYFFSPKPTINYPFEKGHVSPRFRGEHALRRYPNGEERC
IACKLCEAICPAQAITIEAGPRQNDGTRRTVRYDIDMVKCIYCGFCQEACPVDAIVEGPN
FEFATETREELYFSKEKLLANGDRWEREIARNIALDAPYR
>2[4Fe-4S]ST17(rhz:RHPLAN_38840) Rhodoplanes sp. Z2-YC6860
MRLDQAARSIFLQEFVSAFFLGMRYFFKPKATLNYPFEKGPISPRFRGEHALRRYPNGEE
RCIACKLCEAICPAQAITIEAGPRRNDGTRRTTRYDIDMVKCIYCGLCQEACPVDAIVEG
PNFEFATETREELYYDKEKLLANGDRWEREIAKNIALDAPYR
>2[4Fe-4S]ST17(msc:BN69_1742) Methylocystis sp. SC2
MKLDQAAKSLFLTEFVGAFLLSMRYFFKPKATINYPHEKNPQSPRYRGEHALRRYPNGEE
RCIACKLCEAICPAQAITIEAGPRRNDGTRRTTRYDIDMVKCIYCGYCQEACPVDAIVEG
PNSEFSVETREELYYNKQRLLENGDRWEREIARNIAMDAQYR
>2[4Fe-4S]ST17(mbry:B1812_03680) Methylocystis bryophila

MRLDQEAKSLFLLEFVAAFFLGIRYIFKPKATINYPHEKNPQSPRYRGEHALRRYPNGEE
RCIACKLCEAICPAQAITIEAGPRRNDGTRRTTRYDIDMVKCIYCGFCQEACPVDAIVEG
PNAEFATETREELYYNKERLLENGARWEREIARNIALDAPYR
>2[4Fe-4S]ST17(mey:TM49_13470) Martelella endophytica
MGSVAQAFNAIFLKEFVAAFFLTMRYFFGQKSTVNYPFEKGPVSPRFRGEHALRRYPNGE
ERCIACKLCEAICPAQAITIEAGPRRNDGTRRTVRYDIDMVKCIYCGFCQEACPVEAIVE
GPNFEFATETREELYYDKERLLANGDRWEREIARNIAMDAPYR
>2[4Fe-4S]ST17(mcg:GL4_2076) Methyloceanibacter caenitepidi
MTRFAQAAKSLLLLEFVSAFWLAMKYFVAPKATLNYPYEKGPLSPRFRGQHALRRYPNGE
ERCIACKLCEAICPAQAITIEAGPRRNDGTRRTTRYDIDMVKCIYCGFCQEACPVEAIVE
GPNFEFATETREELYYDKARLLSNGDRWEREIAKNIELDSAYR
>2[4Fe-4S]ST17(psin:CAK95_22835) Pseudorhodoplanes sinuspersici
MRLDQAARSVFLAEFVQAFFLGMRYFFKPKPTINYPFEKNPISPRFRGEHALRRYPNGEE
RCIACKLCEAICPAQAITIEAGPRRNDGTRRTTRYDIDMVKCIYCGLCQEACPVDAIVEG
PNFEFATETREELYYDKERLLANGDRWEREIAQNIRLDAPYR
>2[4Fe-4S]ST17(psf:PSE_3429) Pseudovibrio sp. FO-BEG1
MGLGQAAKSLLLKEFVSAFFLAMRYFFKPKPTINYPFEKGPVSPRFRGEHALRRYPNGEE
RCIACKLCEAICPAQAITIEAGPRRNDGTRRTTRYDIDMTKCIYCGFCQEACPVDAIVEG
PNFEFATETREELFYDKDKLLANGERWEREIARNIELDAPYR
>2[4Fe-4S]ST17(pphr:APZ00_17540) Pannonibacter phragmitetus
MGLDQAIKSLFLKEFVSAFFLAMRYFFKPKPTVNYPFEKGPVSPRFRGEHALRRYPNGEE
RCIACKLCEAICPAQAITIEAGPRRNDGTRRTTRYDIDMVKCIYCGFCQEACPVDAIVEG
PNFEFATETREELYYSKEKLLANGDRWEREIARNIAMDAPYR
>2[4Fe-4S]ST17(lap:ACP90_13715) Labrenzia sp. CP4
MGLSQAVKSLFLQEFVSAFVLAMRYFFKPKPTVNYPFEKGPVSPRFRGEHALRRYPNGEE
RCIACKLCEAICPAQAITIEAGPRRNDGTRRTTRYDIDMVKCIYCGFCQEACPVDAIVEG
PNFEFATETREELYYDKEKLLANGDRWEREIARNIEMDAPYR
>2[4Fe-4S]ST17(lagg:B0E33_25530) Labrenzia aggregata
MGLSQAVKSLFLQEFVSAFVLAMRYFFKPKPTVNYPFEKGPVSPRFRGEHALRRYPNGEE
RCIACKLCEAICPAQAITIEAGPRRNDGTRRTTRYDIDMVKCIYCGFCQEACPVDAIVEG
PNFEFATETREELYYDKEKLLANGDRWEREIARNIEMDAPYR

2[4Fe-4S]Alv

Subtype 1

>2[4Fe-4S]AlvST1(mgy:MGMSRv2__1443) Magnetospirillum gryphiswaldense MSR-1 v2

MSLKITEDCTSCDACVSVCPNTAISAGEVIYVIEEDRCTECVGAEDTPQCQLVCPADCIV

IGTEETPDQLQAKYEALHG

>2[4Fe-4S]AlvST1(magq:MGMAQ_1616)Magnetospira sp. QH-2

MALLINEDCTNCDACVPVCPNEAITEGDVIYEIDPARCTECVGAEDEPQCELVCPADCIF

PDPANEETQEQLQAKYEALRV

>2[4Fe-4S]AlvST1(rpe:RPE_0603)Rhodopseudomonas palustris BisA53

MALMINEDCTGCDACRPVCPNDAIKAGDPLYRVDPARCTECVGAEDEPQCQVVCPADCLV

ANPEWAETPEQLQEKYERLHS

>2[4Fe-4S]AlvST1(rpt:Rpal_0729)Rhodopseudomonas palustris TIE-1

MALMINEDCTACDACRPVCPNQAISASDTICAVDALRCTECVGAEDEPQCQLVCPADCIV

PNPDWRETPEHLQDKYQQLHS

>2[4Fe-4S]AlvST1(rpx:Rpdx1_4124)Rhodopseudomonas palustris DX-1

MALMINEDCTACDACRPVCPNQAISASDTIYAVDALRCTECVGAEDEPQCQLVCPADCIV

PNPDWRETPEQLQDKYQQLHS

>2[4Fe-4S]AlvST1(rpa:RPA0662)Rhodopseudomonas palustris CGA009

MALMINEDCTACDACRPVCPNQAISASDTIYAVDALRCTECVGAEDEPQCQLVCPADCIV

PNPDWRETPEQLQDKYQQLHS

>2[4Fe-4S]AlvST1(rpc:RPC_1024)Rhodopseudomonas palustris BisB18

MALMINEDCTACDACRPVCPNEAIAAGDPMFVIDALRCTECVGAEDEPQCRLVCPADCIV

ANLDWSETPEELQTKYDQLHG

>2[4Fe-4S]AlvST1(rpd:RPD_1533)Rhodopseudomonas palustris BisB5
MALMINDDCTACDACRPVCPNEAIAAGEPLFVIDALRCTECVGAEDEPQCRLVCPAGCVV
ANPDWSETPEELQAKYDQLHS
>2[4Fe-4S]AlvST1(abs:AZOBR_70146) Azospirillum brasilense Sp245
MAYKIKASDCTACGACEAECPNNAISFKKGAYAINADLCTECKGQFSSPQCASVCPADCC
VPA
>2[4Fe-4S]AlvST1(abq:ABAZ39_09920)Azospirillum brasilense Az39
MAYKIKASDCTACGACEAECPNNAISFKKGAYAINADLCTECKGQFSSPQCASVCPADCC
VPA

Subtype 2

>2[4Fe-4S]AlvST2(bra:BRADO5432)Bradyrhizobium sp. ORS 278
MSNSTRDGRDWRPDYLVAIDAAKCIGCGRCYKVCGREVMTLKGINDEGEFVELDDDEDDE
VEKKIMVMHDTGACIGCGACARVCPTNCQTHSPAG
>2[4Fe-4S]AlvST2(bbt:BBta_5917)Bradyrhizobium sp. BTAi1
MSNSTRDGRDWKPDYLVAINPEKCIGCGRCYKVCGREVMTLKGINDEGEFVELDDDEDDE
VEKKIMVMHDTGACIGCGACARVCPTNCQTHSPAA
>2[4Fe-4S]AlvST2(aol:S58_22740)Bradyrhizobium oligotrophicum
MSNSTRDGRDWRPDYLVAIDAGKCIGCGRCYKVCGREVMTLKGINDEGEFVELDDDEDDE
VEKKIMVMHDTGACIGCGACARVCPTNCQTHSPAA
>2[4Fe-4S]AlvST2(brc:BCCGELA001_31120)Bradyrhizobium sp. CCGE-LA001
MSSITRGGREWTPNYLDSIDAKKCIGCGRCFKVCGRNVMTLKGISEEGELVDLEDDEDDE
IEKKIMVMNDKGECIGCGACARVCPTSCQAHDPAIAAPV

>2[4Fe-4S]AlvST2(bic:LMTR13_26810)Bradyrhizobium icense
MSFATRDGRDWTPNYLISIDPKKCIGCGRCFKVCGRDVMTLKGINEEGDLVDLDDDEDDE
IEKKIMVMNDQGACIGCGACARVCPTNCQTHQPAATEAA
>2[4Fe-4S]AlvST2(xau:Xaut_0096) Xanthobacter autotrophicus
MSNETRDGRAWQPDYLLDIDADKCIGCGRCFKVCGRDVMTLKGINEDGEVVSLDDDEDDE
VEKKVMVMNDTGACIGCGACARVCPANCQVHGHEQAAA
>2[4Fe-4S]AlvST2(azc:AZC_1034) Azorhizobium caulinodans
MSQSTRDGRAWEPEYLVAIDDRKCIGCGRCYKVCGREVMTLKGITDEGEIVSLDDDEDDE
VEKKIMVMADTGACIGCGACARVCPTNCQTHVPASQAA
>2[4Fe-4S]AlvST2(msc:BN69_2631)Methylocystis sp. SC2
MSQATRDGRDWCPEYLVAIDPGKCIGCGRCFKVCGRDVMTLKGLNEDGELVALDDDEDEE
VEKKIMVMNDIGACIGCGACARVCPTNCQTHAAEDVALV
>2[4Fe-4S]AlvST2(bja:bsr1750)Bradyrhizobium diazoefficiens USDA 110
MSFATRDGRDWMPQYLASIDAKKCIGCGRCFKVCGQDVMTLKGINEEGELVNLDNDADDE
VEKKIMVLNDQGACIGCGACDRVCPASCQTHVPAAAA

Subtype 3

>2[4Fe-4S]AlvST3(smk:Sinme_6346)Sinorhizobium meliloti AK83
MISSFVTRDGSRWMPKYLSAIDGATCIGCGRCFKVCSREVMHLHGIDDVGEILGPFDGEE
DDFGGELNRMIMVVDFRGRCIGCGACARVCPRDCQTHVAADILAA
>2[4Fe-4S]AlvST3(smx:SM11_pC1117)Sinorhizobium meliloti SM11
MISSFVTRDGSRWMPKYLSAIDGATCIGCGRCFKVCSREVMHLHGIDDVGEILGPFDGEE
DDFGGELNRMIMVVDFRGRCIGCGACARVCPRDCQTHVAADILAA
>2[4Fe-4S]AlvST3(daa:AKL17_1557)Defluviimonas alba

MPAIAHTRGGAEYVPQYLMAIDAGKCIGCGRCFKVCGRGVMTLHGLTGEGQLVRPGSDEW
DELEDEIIKKVMALIAPENCIGCGACARVCPSDCQSHAALS
>2[4Fe-4S]AlvST3(rcp:RCAP_rcc03275)Rhodobacter capsulatus
MMPTVAYTRGGAEYTPVYLMKIDEQKCIGCGRCFKVCGRDVMSLHGLTEDGQVVAPGTDE
WDEVEDEIVKKVMALTGAENCIGCGACARVCPSECQTHAALS
>2[4Fe-4S]AlvST3(ngl:RG1141_PB00730)Neorhizobium galegae bv. officinalis bv. officinalis HAMBI 1141
MNRSGFTRDGCSWIPEYLNFVDAGTCIGCGRCFKVCSREVMHPYGINDAGQILGVCRGAE
EDFDGELTRVIMAVDYPGRCIGCGACARVCPKNCQVYVRADLLPSEPHK

Subtype 4

>2[4Fe-4S]AlvST4(rle:pRL100156)Rhizobium leguminosarum bv. viciae 3841
MTSSSVTRDGSRWMPEYLNSINPAMCIGCGRCFKVCSREVMHAHGIDEAGDMLGVCDGED
DFNGELSRVIMVIDYPGRCIGCGACARVCPKNCQTHIAVHHRSLHES

Subtype 5

>2[4Fe-4S]AlvST5(rhc:RGUI_2011)Rhodovulum sp. P5
MPITATTRGGTEYVPEFLVEIDMEKCIGCGRCYKVCARDVLDLKGVTDEDEIVDVDDEEY
EDDIERKIMVVSDADDCIGCGACARVCPSNCQTHAPAEAA

>2[4Fe-4S]AlvST5(rsu:NHU_01426) Rhodovulum sulfidophilum

MPTRSRRITGAPTDPHDRIGAPSRNRPRHDVNDSKDTGQALKAAPKAAKEIRAMTITATTRGGTEYIPEFLMAIDQGKCIGCGRCFKVCARNVLTLKGVTDEDEIVDLDDEEFEDEIERRIMVIADGDD
CIGCGACARVCPSSCQTHAPA

Subtype 6

>2[4Fe-4S]AlvST6(brs:S23_46360)Bradyrhizobium sp. S23321

MSSATRDGRDWRPDYLVSIDPRKCIGCGRCYKVCGREVMTLKGLNEDDEMIDLDDDDDEV
EKKVMVMNDDGACIGCGACARVCPTNCQTHAPA

Subtype 7

>2[4Fe-4S]AlvST7(mbry:B1812_08085) Methylocystis bryophila

MSEFKTRDGRDYVPQYLTDIDASTCIGCGRCFKVCPQGVMALYGVNDEGEILGAVTEDDDDDNDFDGELNRKIMKVEEAGACIGCNACARVCPKNCQTHVSADKIAA

Subtype 8

>2[4Fe-4S]AlvST8(msl:Msil_3624) Methylocella silvestris

MSEFKTRGGAAWTPLYLTEIDPVTCIGCGRCFKVCPQNVMTLYGVNDEGEILGAVTQDDDDDFDGDLNRKIMAVEHPDSCIGCNACSKVCPKNCQTHVSADALSA

Firmicutes

2Fe-2S
Subtype 20

>2Fe-2SST20(BAA_2853)Bacillus anthracis A0248
MPKLTIEDNGVNILHRCGGKARCTTCRVEIIAGDFCEASANEKNAMTEKGIEDHLRLSCQ
MRVHKDIVVRPVLTVESSGLDAGPRPAE

>2Fe-2SST20(GBAA_2790)Bacillus anthracis Ames Ancestor
MPKLTIEDNGVNILHRCGGKARCTTCRVEIIAGDFCEASANEKNAMTEKGIEDHLRLSCQ
MRVHKDIVVRPVLTVESSGLDAGPRPAE

>2Fe-2SST20(BAMEG_1806)Bacillus anthracis CDC 684
MPKLTIEDNGVNILHRCGGKARCTTCRVEIIAGDFCEASANEKNAMTEKGIEDHLRLSCQ
MRVHKDIVVRPVLTVESSGLDAGPRPAE

>2Fe-2SST20(H9401_2660)Bacillus anthracis H9401
MPKLTIEDNGVNILHRCGGKARCTTCRVEIIAGDFCEASANEKNAMTEKGIEDHLRLSCQ
MRVHKDIVVRPVLTVESSGLDAGPRPAE

>2Fe-2SST20(BAS2601)Bacillus anthracis Sterne
MPKLTIEDNGVNILHRCGGKARCTTCRVEIIAGDFCEASANEKNAMTEKGIEDHLRLSCQ
MRVHKDIVVRPVLTVESSGLDAGPRPAE

>2Fe-2SST20(BAPAT_2680)Bacillus anthracis SVA11
MPKLTIEGAGTFDVKEGTKLVLAIEDNGVNILHRCGGKARCTTCRVEIIAGDFCEASANE

KNAMTEKGIEDHLRLSCQMRVHKDIVVRPVLTVESSGLDAGPRPAE

>2Fe-2SST20(Bcell_2204)Bacillus cellulosilyticus
MPNVTVQGSKTFTVEEDKKLVLALEDNGIDILHRCGGKAKCTTCRVEVLQGNFGPLTNIE
KEAFERKGIEDTYRLSCQVYVTEDVTVRPIMTESSTNMDVGPRPEE

>2Fe-2SST20(BCA_2874)Bacillus cereus 03BB102
MPKLTIEGAGTFDVKEGTKLVLAIEDNGVNILHRCGGKARCTTCRVEIIAGDFCEASANE
KNAMTEKGIEDHLRLSCQMRVHKDIVVRPVLTVENSGLDAGPRPAE

>2Fe-2SST20(BCAH820_2797)Bacillus cereus AH820
MPKLTIEGAGTFDVKEGTKLVLAIEDNGVNILHRCGGKARCTTCRVEIIAGDFCEASANE
KNAMTEKGIEDHLRLSCQMRVHKDIVVRPVLTVENSGLDAGPRPAE

>2Fe-2SST20(BCE_2821)Bacillus cereus ATCC 10987
MPKLTIEGTGTFDVQEGTKLVLAIEDNGVNILHRCGGKARCTTCRVEIIAGDFCEANAKE
KNAITEKGIEDHLRLSCQMRVHKDIVVRPVLTVENSGLDAGPRPAE

>2Fe-2SST20(BC2795)Bacillus cereus ATCC 14579
MPKLTIEGAGTFDVKEGTKLVLAIEDSGVNILHRCGGNARCTTCRVEILAGDFCEASANE
KHAMTEKGIEDHLRLSCQMRVHKDIVVRPVLTVENSGLDAGPRPAE

>2Fe-2SST20(BCB4264_A2800)Bacillus cereus B4264
MPKLTIEGAGTFDVKEGTKLVLAIEDSGVNILHRCGGNARCTTCRVEILAGDFCEASANE
KHAMTEKGIEDHLRLSCQMRVHKDIVVRPVLTVENSGLDAGPRPAE

>2Fe-2SST20(BACI_c27540)Bacillus cereus biovar anthracis CI

MPKLTIEGAGTFDVKEGTKLVLAIEDNGVNILHRCGGKARCTTCRVEIIAGDFCEASANE

KNAMTEKGIEDHLRLSCQMRVHKDIVVRPVLTVESSGLDAGPRPAE

>2Fe-2SST20(BCE33L2520)Bacillus cereus E33L

MPKLTIEGTGTFDVKEGTKLVLAIEDNGVNILHRCGGKARCTTCRVEIIAGDFCEASANE

KNAMTEKGIEDHLRLSCQMRVHKDIVVRPVLTVENSGLDAGPRPAE

>2Fe-2SST20(bcf_13665)Bacillus cereus F837/76

MPKLTIEGAGTFDVKEGTKLVLAIEDNGVNILHRCGGKARCTTCRVEIIAGDFCEASANE

KNAMTEKGIEDHLRLSCQMRVHKDIVVRPVLTVENSGLDAGPRPAE

>2Fe-2SST20(BCK_20930)Bacillus cereus FRI-35

MPKLTIEGTGTFDVKEGTKLVLAIEDNGVNILHRCGGKARCTTCRVEIIAGDFCEANAKE

KNAMTEKGIEDHLRLSCQMRVHKDIVVRPILTVENSGLDAGPRPAE

>2Fe-2SST20(BcrFT9_02196)Bacillus cereus FT9

MPKLTIEGAGTFDVKEGTKLVLAIEDSGVNILHRCGGNARCTTCRVEIIAGDFCEASANE

KNAMTEKGIEDHLRLSCQMRVHKDIVVRPVLTVESSGLDAGPRPAE

>2Fe-2SST20(BCG9842_B2492)Bacillus cereus G9842

MPKLTIEGAGTFDVKEGTKLVLAIEDSGVNILHRCGGNARCTTCRVEILAGDFCEASANE

KHAMTEKGIEDHLRLSCQMRVHKDIVVRPVLTVENSSLDAGPRPAE

>2Fe-2SST20(BCN_2651)Bacillus cereus NC7401

MPKLTIEGAGTFDVKEGTKLVLAIEDNGVNILHRCGGKARCTTCRVEILAGDFCEASANE

KHAMTEKGIEDHLRLSCQMRVHKDIVVRPVLTVENSGLDAGPRPAE

>2Fe-2SST20(BCQ_2633)Bacillus cereus Q1
MPKLTIEGTGTFDVQEGTKLVLAIEDNGVNILHRCGGKARCTTCRVEIIAGDFCEANAKE
KNAMTEKGIEDHLRLSCQMRVHKDIVVRPVLTVENSGLDAGPRPAE

>2Fe-2SST20(Bcer98_1912)Bacillus cytotoxicus
MPKLTVIGTGTFDIEEGTKLVLALEDNGVPILHRCGGKARCTTCRVEVIAGDFCEPTPKE
THAMTEKGIEDHLRLSCQMRVYKDLTIRPILTVENSGLDAGPRPAE

>2Fe-2SST20(BMD_1901)Bacillus megaterium DSM 319
MPRVTVLGYGSFEVAKGKKLVLALEDNGINILHRCGGKAKCTTCRVEVLEGEYCDLTHAE
KNAYSESSIEDHLRLSCQIRVNEDITVRPILTVENSGREAGPRPAE

>2Fe-2SST20(BMWSH_3338)Bacillus megaterium WSH-002
MPRVTVLGHGSFEVEKGKKLVLALEDNGINILHRCGGKAKCTTCRVEVLEGEYCDLTNAE
KNAYSESSIEDHLRLSCQIRVNEDVTVRPILTVESSGREAGPRPAE

>2Fe-2SST20(BcerKBAB4_2595)Bacillus mycoides KBAB4
MPKLTVEGTGTFDIKEGTKLVLALEDNGVHILHRCGGKARCTTCRVEVIAGDFCEATNDE
KQAITEKGIEDHLRLSCQMRVHKDITVRPILTVENSGLDAGSRPAE

>2Fe-2SST20(bwei_2240)Bacillus mycoides WSBC 10204
MPKLTVEGTGTFDIKEGTKLVLALEDNGVHILHRCGGKARCTTCRVEVIAGDFCEATNDE
KQAITEKGIEDHLRLSCQMRVHKDITVRPILTVENSGLDAGPRPAE

>2Fe-2SST20(BALH_2507)Bacillus thuringiensis Al Hakam
MPKLTIEGAGTFDVKEGTKLVLAIEDNGVNILHRCGGKARCTTCRVEIIAGDFCEASANE
KNAMTEKGIEDHLRLSCQMRVHKDIVVRPVLTVENSGLDAGPRPAE

>2Fe-2SST20(BMB171_C2499)Bacillus thuringiensis BMB171
MPKLTIEGAGTFDVKEGTKLVLAIEDSGVNILHRCGGNARCTTCRVEILAGDFCEASANE
KHAMTEKGIEDHLRLSCQMRVHKDIVVRPVLTVENSGLDAGPRPAE

>2Fe-2SST20(BTB_c29080)Bacillus thuringiensis Bt407
MPKLTIEGAGTFDVKEGTKLVLAIEDSGVNILHRCGGNARCTTCRVEILAGDFCEASANE
KHAMTEKGIEDHLRLSCQMRVHKDIVVRPVLTVENSGLDAGPRPAE

>2Fe-2SST20(BTG_05425)Bacillus thuringiensis HD-771
MPKLTIEGAGTFDVKEGTKLVLAIEDSGVNILHRCGGNARCTTCRVEILAGDFCEASANE
KHAMTEKGIEDHLRLSCQMRVHKDIVVRPVLTVENSSLDAGPRPAE

>2Fe-2SST20(BTF1_11475)Bacillus thuringiensis HD-789
MPKLTIEGAGTFDVKEGTKLVLAIEDSGVNILHRCGGNARCTTCRVEILAGDFCEASANE
KHAMTEKGIEDHLRLSCQMRVHKDIVVRPVLTVENSSLDAGPRPAE

>2Fe-2SST20(MC28_1980)Bacillus thuringiensis MC28
MPKLTIEGAGTFDVKEGTKLVLAIEDNGVNILHRCGGNARCTTCRVEIIAGDFCEASANE
KNAMTEKGIEDHLRLSCQMRVHKDIVVRPVLTVENSGLAAGPRPAE

>2Fe-2SST20(CT43_CH2783)Bacillus thuringiensis serovar chinensis CT-43
MPKLTIEGAGTFDVKEGTKLVLAIEDSGVNILHRCGGNARCTTCRVEILAGDFCEASANE
KHAMTEKGIEDHLRLSCQMRVHKDIVVRPVLTVENSGLDAGPRPAE

>2Fe-2SST20(YBT020_13935)Bacillus thuringiensis serovar finitimus YBT-020
MPKLTIEGAGTFDVKEGTKLVLAIEDNGVNILHRCGGKARCTTCRVEIIAGDFCEASANE
KNAMTEKGIEDHLRLSCQMRVHKDIVVRPVLTAESSGLDAGPRPAE

>2Fe-2SST20(BTK_15830)Bacillus thuringiensis serovar kurstaki HD-1
MPKLTIEGAGTFDVKEGTKLVLAIEDSGVNILHRCGGNARCTTCRVEILAGDFCEASANE
KHAMTEKGIEDHLRLSCQMRVHKDIVVRPVLTVENSGLDAGPRPAE

>2Fe-2SST20(HD73_3192)Bacillus thuringiensis serovar kurstaki HD73
MPKLTIEGAGTFDVKEGTKLVLAIEDSGVNILHRCGGNARCTTCRVEILAGDFCEASANE
KHAMTEKGIEDHLRLSCQMRVHKDIVVRPVLTVENSGLDAGPRPAE

>2Fe-2SST20(YBT1520_15760)Bacillus thuringiensis serovar kurstaki YBT-1520
MPKLTIEGAGTFDVKEGTKLVLAIEDSGVNILHRCGGNARCTTCRVEILAGDFCEASANE
KHAMTEKGIEDHLRLSCQMRVHKDIVVRPVLTVENSGLDAGPRPAE

>2Fe-2SST20(H175_ch2833)Bacillus thuringiensis serovar thuringiensis IS5056
MPKLTIEGAGTFDVKEGTKLVLAIEDSGVNILHRCGGNARCTTCRVEILAGDFCEASANE
KHAMTEKGIEDHLRLSCQMRVHKDIVVRPVLTVENSGLDAGPRPAE

>2Fe-2SST20(YBT1518_15385)Bacillus thuringiensis YBT-1518

MPKLTIEGAGTFDVKEGTKLVLAIEDSGVNILHRCGGNARCTTCRVEILAGDFCEASANE
KHAMTEKGIEDHLRLSCQMRVHKDIVVRPVLTVENSGLDAGPRPAE

>2Fe-2SST20(Btoyo_0077)Bacillus toyonensis

MPKLTIEGAGTFDVKEGTKLVLAIEDNGVNILHRCGGNARCTTCRVEIIAGDFCEASANE
KNAMTEKGIEDHLRLSCQMRVHKDIVVRPVLTVENSGLAAGPRPAE

>2Fe-2SST20(BBR47_24550)Brevibacillus brevis

MGKVTFLPSKKSVKARTGQTLVGVASSARVVIPQRCGGHASCLMCRVVVENGLLCPPTAL
EKRKLPEKDLANGIRLACQAKTTEKDCTVRIPESKLKSVVAAALERQRKENEDGM

>2Fe-2SST20(BRLA_c018850)Brevibacillus laterosporus

MPKVTFYPSKKTIKARPGQTILQLSRVARVAIPTRCDGNAACLLCKVTIEKGTASPLSAG
EERKLAERDKARGMRLACQVRVLEEDLVVRIPESRWKSVVEKALERQRLEEEEW

>2Fe-2SST20(ERIC2_c13490)Paenibacillus larvae

MKLYTIRFEPQGKVVEVRPGTSVLDAARKAGVVIRTRCGGKAGCLMCKIQVTKPSGLSKP
NDPEKRKLGSLADKGTRLSCQAKIIGNTEVIIPEDPLKAAVRAQLEKQRKEQEEDLW

>2Fe-2SST20(SY83_18640)Paenibacillus swuensis

MLVEVTFLPEGRKAQVRPGTTLLDAARKARVNIRTRCGGKAGCLMCKVTVEDGIGVAGMN
HNEALKLGTLAETGQRLACQAKVTGACVANVPEDPLKAAVRAQLLRQQQEDEW

>2Fe-2SST20(IJ21_23580)Paenibacillus sp. 32O-W

MKCEVTFQPSGKKIVVRPGTSVLEASRKAGVAIRTRCGGKAACLMCKVYAKAGDGLSPVQ

DNERRKLAGLADEGMRLACQARVVGQAEIGLPEDPLRAAVRRQLARQAEDDLW

Subtype 18

>2Fe-2SST18(BS614_22620)Paenibacillus xylanexedens
MDYKVTFLPMNKSIALKPGATLLHAARRAGVKITTRCDGKAACLMCKVNVDEEHRAELYP
PTDAEKRKLGSLLEAGTRLSCQAKVCGSLSVHVPEDPLKAAIRKQLERQQQEDDWF

>2Fe-2SST18(PM3016_3016)Paenibacillus mucilaginosus 3016
MSMNAEVTFLPDGRKVTVRPGTTLLEASRRARVTIRTRCAGKAACLMCKVRVTEGMESGL
AEPGKNERLKLGGQEQDGYRLACQAKVTGPVQVEVPEDPLKAAIRKQLEKQQEEDW

>2Fe-2SST18(KNP414_02773)Paenibacillus mucilaginosus KNP414
MNAEVTFLPDGRKVTVRPGTTLLEASRRARVTIRTRCAGKAACLMCKVRVTEGMESGLAE
PGKNERLKLGGQEQDGYRLACQAKVTGPVQVEVPEDPLKAAIRKQLEKQQEEDW

>2Fe-2SST18(B2K_15295)Paenibacillus mucilaginosus K02
MSMNAEVTFLPDGRKVTVRPGTTLLEASRRARVTIRTRCAGKAACLMCKVRVTEGMESGL
AEPGKNERLKLGGQEQDGYRLACQAKVTGPVQVEVPEDPLKAAIRKQLEKQQEEDW

>2Fe-2SST18(B9T62_20255)Paenibacillus donghaensis
MKPHKGCTVIFQPSGRQATVSRGVSVLEAARKAGVNIATRCGGKAGCLMCKVTVEAGEAS
AVQPPGDVERRKLGSTLNTGIRLACQAAVWSDVTVQVPEDPLKAAVRRKLEAARRGEEDE

LW

>2Fe-2SST18(PRIO_4488)Paenibacillus riograndensis
MKRQKGWVVTFRPDGRTTEVMHGTPLLEAARKAGVALTTRCGGKAGCLMCKVKVEDQFAA
GLQPPGDTERRKLGSLLVEGVRLACQAAVWSNVTVHIPEDPLKAAVRRRLEAARRGDEDE
LW

>2Fe-2SST18(UB51_05390)Paenibacillus sp. IHBB 10380
MEYEVTFKPMGKKIKVRVGTTILDTARKCGVHIATRCGGKAACLMCKVELSPQEESAVST
ANDAERRKLGSLLEQGIRLSCQARIQNEISVIIPEDPLKAAVRKQLEAARNKDHDNELW

>2Fe-2SST18(ABE82_15110)Paenibacillus peoriae
MNVQIMFKPSGRRVQVGQGTSLLQAARKAGVYIPTRCDGKAACLMCKVQISPEWAEFAGQ
PSDAEQRKLGPLLDEGIRLSCQARVQGDMQVTVPEDRLKAAIRRQLERQAMDDELW

>2Fe-2SST18(X809_31095)Paenibacillus polymyxa CR1
MNVQITFRPSGRRVQVGQGTSLLQAARKAGVYIPTRCDGKAACLMCKVQISPEWAEFADQ
PSDAEQRKLGPLLDEGIRLSCQARVQGDMEVTIPEDRLKAAIRRQLERQALDDELW

>2Fe-2SST18(PPM_2950)Paenibacillus polymyxa M1
MNVQITFKPSGRCVQVSRGTSLLQAARKAGVYIPTRCDGKAACLMCKVEIAPERAELAGR
PNDAEQRKLGPLLNEGIRLSCQARAQGDMEVTIPEDRLKAAIRRQLERQGLDDELW

>2Fe-2SST18(RE92_22030)Paenibacillus polymyxa Sb3-1
MNVQITFKPSGRCVQVSRGTSLLQAARKAGVYIPTRCDGKAACLMCKVEIAPERAELAGR
PNDAEQRKLGPLLNEGIRLSCQARAQGDMEVTIPEDRLKAAIRRQLERQGLDDELW

>2Fe-2SST18(PPSC2_14675)Paenibacillus polymyxa SC2
MNVQITFKPSGRCVQVSRGTSLLQAARKAGVYIPTRCDGKAACLMCKVEIAPERAELAGR
PNDAEQRKLGPLLNEGIRLSCQARAQGDMEVTIPEDRLKAAIRRQLERQGLDDELW

>2Fe-2SST18(PPSQR21_029190)Paenibacillus polymyxa SQR-21
MNVQITFKPSGKRVQVGQGTSLLQAARKAGVYIPTRCDGKAACLMCKVEIAPERAELAGH
PNDAEQRKLGPLLNEGIRLSCQARAQGDIEVTIPEDRLKAAIRRQLERQALDDELW

>2Fe-2SST18(IJ22_23190)Paenibacillus naphthalenovorans
MASEITFMPDDKSIQARPGTTVLDAARKARVHIRTRCSGKAACLMCKVRVIAGGETGLKA
MNVNERLKLGSLYEEGYRLACQAKLSGQEPVTVFVPEDPLKSAIRRQLEKQQEEEW

>2Fe-2SST18(BME96_01300)Virgibacillus halodenitrificans
MPSITFYNGTSLLKKIEATKGRTILAIAKQGRVALRHKCGGKASCLTCKVTIPNQSAVSA
PEPKEARKLGESNLEKGIRLSCQTRVYGDIEAVIPEDPYKARIRALLEQARQE

>2Fe-2SST18(X953_01935)Virgibacillus sp. SK37
MPSITFYNGTSLLKKVEAIKGRTILETAKQGRVALRHKCGGKASCLTCKVTIANQSAVSA
PEPKEARKLGESNLEKGLRLSCQTRVYGDIEAVIPEDPYKARIRALLEQARQE

>2Fe-2SST18(CB4_03083)Aneurinibacillus soli
MAKVTFVPSGRSVEARQGETILRAASRARVPITQRCGGNGSCTMCKVRIDGDSKVSPPCE
IEKRWISSAELARGVRLACQTKIQGTTRVSLPQSKLAAVVQAQLAEQRERREGQEKTQE

>2Fe-2SST18(ACH33_12440)Aneurinibacillus sp. XH2

MPKLTFIPVNRSVQVRIGETILRAASRARVAISQRCGGKGACMMCKVQVAEGSKVSSPKE
LERRMIGAQNLARGIRLACQTRVQGETRVHLPESRLAAVVRAQLEKQRQELDEE

>2Fe-2SST18(ABC0324)Bacillus clausii
MIHAEGYHSFEAENGKKLVLALEDNGVDILHRCGGNARCTTCMCEITEGDAGPIGEAEAA
IRAKKGITAENLRLSCQIRVANDLKVKPLRTATSEQMDPGKRPEE

>2Fe-2SST18(BleG1_0216)Bacillus lehensis
MPMIHAEGYHSFRAEQGKKLVLALEDNGVDILHRCGGKARCTTCLCEIVDGDAGPISEIE
AAAREKKGITAENLRLSCQIRIEKDLKVKPLRTATSEQMDPGTRPEE

Subtype 6

>2Fe-2SST6(ERIC2_c13470)Paenibacillus larvae
MVTLKTRAMQKDIEPVPGETLLQLAIRNKMDWGHNCKRGTCARCRCFIQEGAALLEDSTE
AEELRLEPEEIEQAYSLACQAVIKDEGRLIAVHKPYFFF

>2Fe-2SST6(BBD41_26010)Paenibacillus ihbetae
MFKFWRSRKNSHSAGHPSASGGQELAEAVPDKLIELSGREVHKSVAPVLGMTVLDLAERN
DVDWNSFCKRGTCARCRCHVSVGVQHLSSPNQAEEQRLDPEELEEGYRLGCQSRIESVGP
IKIKHAPYF
>2Fe-2SST6(GYMC10_3926)Paenibacillus sp. Y412MC10
MFKFWKNKKGENSGEKSSLPAAEERMEAVPDRIIELTGREVQRSVAPVLGMTVLDLAERN
EVDWNSFCKRGTCARCRCMVVEGIEYLSEPNLAEERRLDPEEIEEGYRLGCQSRIETVGP
VKIKHAPYF

>2Fe-2SST6(KNP414_02771)Paenibacillus mucilaginosus KNP414
MITLKGRQGSKTVKPETGFTIVDMALKHGVEWGFSCLRGTCSRCRCQVVEGAEHLSAPTD
EELDNLGDEEMEEGFRLGCQATVRSAGAVTIVHKPYF

>2Fe-2SST6(PM3016_3014)Paenibacillus mucilaginosus 3016
MEPETGFTIVDMALKHGVEWGFSCLRGTCSRCRCQVVEGAEHLSAPTDEELDNLGDEEIE
EGFRLGCQATVRSAGAVTIVHKPYF

>2Fe-2SST6(B2K_15285)Paenibacillus mucilaginosus K02
MITLKGRQGSKTVEPETGFTIVDMALKHGVEWGFSCLRGTCSRCRCQVVEGAEHLSAPTD
EELDNLGDEEIEEGFRLGCQATVRSAGAVTIVHKPYF

>2Fe-2SST6(IJ21_23560)Paenibacillus sp. 32O-W
MLELKGRTKTAVVEAEQGQSILKLAVKHGVDWGFSCTRGTCARCRCLVVEGAEWLTPITD
AEWDRLEPEEFEQGYRLGCQAIVKAGAGRIVAHNKPYF

>2Fe-2SST6(SY83_18650)Paenibacillus swuensis
MITLTGKTVTKDVEEELGLSLLDLAMKHDIDWGFSCTRGTCARCRCRVSEGMELLNEVTD
EEWNRLDEDELNEGYRLGCQAVVKASGIIQAVNKTYF

>2Fe-2SST6(C1X05_11820)Laceyella sacchari
MAKVSIHIAGQTKEMEVAEGANLLLEAASRSIPIPFKCTTGRCGTCRVRVIEGSSQCSDY
TEPELHHLNETDLASGRRLACQTFIHGDVTIEIP

>2Fe-2SST6(VN24_20295)Paenibacillus beijingensis
MPSITYQNSGKTIEVDKDANILRTSIRYEGTVPYKCGGGLCGTCRVRIVDGHDNLSRVMK

KEIDRLGAERIEEGYRLACQTFVTGDVTIAWGEEDLERLNKIAKRRINASS

>2Fe-2SST6(A361_11485)Bacillus oceanisediminis
MPNIHFVNSNKTLEVPEDSNILRMSLRYDGDLPNRCGGGICGTCVFKTEEGAEYLDNVKI
QERRKLGEEWLGKGYRLGCQTFVTDGDITISWDDEVTDQVKKRKPDKLKQTVTTGK

Subtype 8

>2Fe-2SST8(Sulac_3007)Sulfobacillus acidophilus DSM 10332
MDSRVTEEFPLQVEPGGLTLSIRSDETLLDALKRHGYTVFYGCRRGGCGVCRVTLTAGQV
VMGPYAPQALDDLALEKGQVLACRAKPTSAVVIRIEESNRFRRLLGWPWGSSSAPVTDR

>2Fe-2SST8(TPY_0632)Sulfobacillus acidophilus TPY
MDSRVTEEFPLQVEPGGLTLSIRSDETLLDALKRHGYTVFYGCRRGGCGVCRVTLTAGQV
VMGPYAPQALDDLALEKGQVLACRAKPTSAVVIRIEESNRFRRLLGWPWGSSSAPVTDR

>2Fe-2SST8(CVV65_03255)Kyrpidia spormannii
MFNVEIVDSFYEKEKPFFSCTNQQSLLDAMRLQGVRIKYACKVGGCGLCKIKVVEGEYRR
GTCSVSGLPYDERLQNYTLACKTYPTSDMKILIQI

>2Fe-2SST8(CVV65_11345)Kyrpidia spormannii
MVFEEGREFTCKHEQDVLEAAIAQGVRSVKRGCRGGGCGLCKVQVVEGSYELGKSSVAVL
PEDERQRGFVLACKTTPKSDLVIRLTKDS

>2Fe-2SST8(CVV65_10365)Kyrpidia spormannii

MILTGLGCGMKPRYRVMVSDQQTEYTCRQDENLLSEALNQGIRAIKRGCLGGGCGFCKVR
VESGRYELGKCSVKVLTPEEKERGYVLACRTKPLSDLMIRIEK

>2Fe-2SST8(CVV65_10625)Kyrpidia spormannii
MANEYRIFVANKSREYLCRHGQNILSSSIEQGVRVIKRGCLGGGCGFCKIRVEAGQYELG
KVSIKALPAEERKEGFVLACKTVPLSDLVIRISGD

Subtype 21

>2Fe-2SST21(BBH88_12590)Planococcus antarcticus
MATWDLQTTEHHVLICNGGSCNQFGAEELTQAIRSEISRRELDGTIHTTRTRCNGRCHDK
CVVIDYPKGIWYKDLKPEDAHLFVDSLHANENFSTKISHSFNGEVFERSTGVVKGVAKDS
EKVSKASK

>2Fe-2SST21(ABE82_10800)Paenibacillus peoriae
MAIFELEPMKHHVLICNGGTCMRHEGEEVTQAIRDEIRKQNAEAYIHTTRTRCNGRCHDA
AVVIVYPQGDWYGQMTPDSGTQLVQKLVTGEKLEPHLFHECTGKSSSE

>2Fe-2SST21(X809_10955)Paenibacillus polymyxa CR1
MAIFELEPMKHHVLICNGGTCMRHEGEEVTQAIRDEIRKQNAEAYIHTTRTRCNGRCHDA
AVVIVYPQGDWYGQMTPDSGTQLVQKLVTGEKLEPHLFHECTGKSSSE

>2Fe-2SST21(RE92_01790)Paenibacillus polymyxa Sb3-1

MAIFDLEPMKHHVLICNGGTCMRHEGEEVTQAIRDEIRKQHAEAYIHTTRTRCNGRCHDA
AVVIVYPQGDWYGQMTPASGTELVQKLVAGEKLEPHLFHECTRKSSSQ

>2Fe-2SST21(PPSC2_10615)Paenibacillus polymyxa SC2
MAIFDLEPMKYHVLICNGGTCMRHEGEEVTQEIRDEIRKQHAEAYIHTTRTRCNGRCHDA
AVVIVYPQGDWYGQMTPASGTELVQKLVAGEKLEPHLFHECTRKSSSQ

Subtype 9

>2Fe-2SST9(VN24_15905)Paenibacillus beijingensis
MSGIRLIQDGNVYEVSFIPGKNLLESGLDNGIPLKYKCRKGSCGVCKIRLISGAGLLTEA
NKRERKLLSDDVKAGYRLACQATMGPSAIDD

>2Fe-2SST9(VN24_20275)Paenibacillus beijingensis
MPNVTLHQNGQVYQQEVGANSNLVVLAGIKKFGHLKYGCGMGKCTKCMVKVLSGAESLPE
PNWKENKMLGEKLSDGYRLACQLYIHEDLEIRQE

>2Fe-2SST9(A361_11445)Bacillus oceanisediminis
MPKVTLHVDGEIVEKQVKDNANLVVLAGIRQFPELKYGCGMGRCTKCTCIVLNGGDDLAP
PNWKEEKMLGDKVKEGYRLTCQMTIQNDIEISQENISVKPPKKTTAVITK

Subtype 4

>2Fe-2SST4(BBD41_04145)Paenibacillus ihbetae
MDYEVLFQPDGKKVRVRPLTNVLDASRRAGVRIPTRCGGKAGCLMCKVIIPEGQRESCLP

PEDKERRKLGSALLEQGYRLSCQTKITGGLQVSIPEDPLKAAIRKQLEAARNRDADDFI

>2Fe-2SST4(D5F53_18705)Paenibacillus lautus

MEYEVLFLPDQKKVKVRPNTNVLDASRRAGVRIPTRCGGKAGCLMCKILVPDEERGHCSV

PETKEIRKLGTSMMEQGYRLSCQTRITSHLKVIIPEDPLKAAIRKQLEAARNREPDDFI

>2Fe-2SST4(GYMC10_2190)Paenibacillus sp. Y412MC10

MEYEVLFLPDQKKVKVRPNTNVLDASRRAGVRIPTRCGGKAGCLMCKILVPDEERGHCSA

PETKEIRKLGTSMMEQGYRLSCQTRITSHLKVIIPEDPLKAAIRKQLEAARNREPDDFI

Subtype 2

>2Fe-2SST2(VN24_24800)Paenibacillus beijingensis

MTYEVTFLPEGRKVSVRPGMTVLGAARQARIGIRSRCGGKAGCLMCKVDVASGGGTAGDG

LSPAGLLEKRKLAGLDSSRTRLACQAKVTGNAVISVPEDPLKAAVRRQLEKAEQDEDDLG

W

Subtype 3

>2Fe-2SST3(LIP_3522)Limnochorda pilosa

MELITPSGTHRLEVAEDQFILDRALQAGLDLPHTCLQGWCTTCAGRILEGEVDPSAALRY

YREDREAGYVLLCTARPRGPLRIQTHQKEALRANRRKHRLPAPRA

Subtype 22

>2Fe-2SST22(Clopa_3476)Clostridium pasteurianum BC1

MVNPKHHIFVCTSCRLNGKQQGFCYSKNSVDIVGEFMEELDGRDLSGDIMVNNTGCFGIC

SQGPIVVVYPEGVWYGNVTADDVEEIVDSLENGEVVKRLQI

>2Fe-2SST22(CPAST_c14230)Clostridium pasteurianum DSM 525 = ATCC 6013

MVNPKHHIFVCTSCRLNGKQQGFCYSKNSVEIVETFMEELDSRDLSSEVMVNNTGCFGIC

SQGPIVVVYPEGVWYGNVTADDVEEIVESHIENGEVVKRLQI

Subtype 5

>2Fe-2SST5(AR543_16580)Paenibacillus bovis

MAGKQQCRIEFRPAGKVVQVPEGTTLLAAARRAGVHLPVRCDGRAACLMCKVMVLPEEQP

LGEISLSAPGAAETRKLGLSLQEHIRLGCQARVTGDSIVRVPEDRLKAAIRKQMEQQNDD

LSDW

4Fe-4S

Subtype 2

>4Fe-4SST2(ACH33_12610)Aneurinibacillus sp. XH2

MTTWVDKDTCIACGACGATAPDVFDYDDEGLAFNKLDDNSNTAEIPDILQDDVRDAAEGC

PTDSIKVEE

>4Fe-4SST2(CFN77_10930)Bacillus altitudinis
MAKYTIVDKDTCIACGACGAAAPDIYDYDDEGIAFVTLDDNQGTVEVPEVLEEDMLDAFE
GCPTDSIKVADETFDGDPLKHE

>4Fe-4SST2(BAA_1572)Bacillus anthracis A0248
MAKYTIVDKDTCIACGACGAAAPDIYDYDDEGIAFVTLDDNQGIVEIPDVLIEDMMDAFE
GCPTDSIKVADESFDGDALKFE

>4Fe-4SST2(A16_15470)Bacillus anthracis A16
MAKYTIVDKDTCIACGACGAAAPDIYDYDDEGIAFVTLDDNQGIVEIPDVLIEDMMDAFE
GCPTDSIKVADESFDGDALKFE

>4Fe-4SST2(A16R_15640)Bacillus anthracis A16R
MAKYTIVDKDTCIACGACGAAAPDIYDYDDEGIAFVTLDDNQGIVEIPDVLIEDMMDAFE
GCPTDSIKVADESFDGDALKFE

>4Fe-4SST2(BA_1503)Bacillus anthracis Ames
MAKYTIVDKDTCIACGACGAAAPDIYDYDDEGIAFVTLDDNQGIVEIPDVLIEDMMDAFE
GCPTDSIKVADESFDGDALKFE

>4Fe-4SST2(GBAA_1503)Bacillus anthracis Ames Ancestor
MAKYTIVDKDTCIACGACGAAAPDIYDYDDEGIAFVTLDDNQGIVEIPDVLIEDMMDAFE
GCPTDSIKVADESFDGDALKFE

>4Fe-4SST2(BAMEG_3090)Bacillus anthracis CDC 684
MAKYTIVDKDTCIACGACGAAAPDIYDYDDEGIAFVTLDDNQGIVEIPDVLIEDMMDAFE
GCPTDSIKVADESFDGDALKFE

>4Fe-4SST2(HYU01_07620)Bacillus anthracis HYU01
MAKYTIVDKDTCIACGACGAAAPDIYDYDDEGIAFVTLDDNQGIVEIPDVLIEDMMDAFE
GCPTDSIKVADESFDGDALKFE

>4Fe-4SST2(BAS1392)Bacillus anthracis Sterne
MAKYTIVDKDTCIACGACGAAAPDIYDYDDEGIAFVTLDDNQGIVEIPDVLIEDMMDAFE
GCPTDSIKVADESFDGDALKFE

>4Fe-4SST2(DJ46_325)Bacillus anthracis Vollum
MAKYTIVDKDTCIACGACGAAAPDIYDYDDEGIAFVTLDDNQGIVEIPDVLIEDMMDAFE
GCPTDSIKVADESFDGDALKFE

>4Fe-4SST2(CY96_06580)Bacillus bombysepticus
MAKYTIVDKDTCIACGACGAAAPDIYDYDDEGIAFVTLDDNQGIVEIPDVLIEDMMDAFE
GCPTDSIKVADESFDGDALKFE

>4Fe-4SST2(BCA_1542)Bacillus cereus 03BB102
MAKYTIVDKDTCIACGACGAAAPDIYDYDDEGIAFVTLDDNHGIVEIPDVLLEDMMDAFE
GCPTDSIKVADESFDGDALKFE

>4Fe-4SST2(BCAH820_1577)Bacillus cereus AH820
MAKYTIVDKDTCIACGACGAAAPDIYDYDDEGIAFVTLDDNQGIVEIPDVLLEDMMDAFE
GCPTDSIKVADESFDGDALKFE

>4Fe-4SST2(BCE_3764)Bacillus cereus ATCC 10987
MLHYTVVNKKNCKSCGLCTSIAPDIYQLDGRGKSFGVLDNNQGIVGIPKTFLQAMLAASD
GCPTSSIQVRKEPFIPL

>4Fe-4SST2(BCE_1607)Bacillus cereus ATCC 10987
MAKYTIVDKDTCIACGACGAAAPDIYDYDDEGIAFVTLDDNQGIVEIPDVLLEDMMDAFE
GCPTDSIKVADESFDGDALKFE

>4Fe-4SST2(BC1483)Bacillus cereus ATCC 14579
MAKYTIVDKDTCIACGACGAAAPDIYDYDDEGIAFVTLDDNQGIVEIPDVLIEDMMDAFE
GCPTDSIKVADESFDGDALKFE

>4Fe-4SST2(BCB4264_A1538)Bacillus cereus B4264
MAKYTIVDKDTCIACGACGAAAPDIYDYDDEGIAFVTLDDNQGIVEIPDVLIEDMMDAFE
GCPTDSIKVADESFDGDALKFE

>4Fe-4SST2(BACI_c15270)Bacillus cereus biovar anthracis CI
MAKYTIVDKDTCIACGACGAAAPDIYDYDDEGIAFVTLDDNQGIVEIPDVLLEDMMDAFE
GCPTDSIKVADESFDGDALKFE

>4Fe-4SST2(BCE33L1364)Bacillus cereus E33L
MAKYTIVDKDTCIACGACGAAAPDIYDYDDEGIAFVTLDDNQGIVEIPDVLLEDMMDAFE
GCPTDSIKVADESFDGDALKFE

>4Fe-4SST2(bcf_07505)Bacillus cereus F837/76
MAKYTIVDKDTCIACGACGAAAPDIYDYDDEGIAFVTLDDNQGIVEIPDVLLEDMMDAFE
GCPTDSIKVADESFDGDALKFE

>4Fe-4SST2(BCK_16575)Bacillus cereus FRI-35
MLHYTVVNKKNCKSCGLCTSIAPDIYQLDGRGKSFGVLDNNQGIVGIPKTFLQAMLAASD
GCPTSSIQVKKEPFIPL

>4Fe-4SST2(BCK_00965)Bacillus cereus FRI-35
MAKYTIVDKDTCIACGACGAAAPDIYDYDDEGIAFVTLDDNQGIVEIPDVLLEDMMDAFE
GCPTDSIKVADESFDGDALKFE

>4Fe-4SST2(BcrFT9_02872)Bacillus cereus FT9
MLHYTVVNKKNCKSCGLCTSIAPDIYQLDGRGKSFGVLDNNQGIVGIPKTFLQAMLAASD
GCPTSSIQVRKEPFIPL

>4Fe-4SST2(BcrFT9_01261)Bacillus cereus FT9
MAKYTIVDKDTCIACGACGAAAPDIYDYDDEGIAFVTLDDNQGIVEIPDVLLEDMMDAFE
GCPTDSIKVADESFDGDALKFE

>4Fe-4SST2(BCG9842_B3807)Bacillus cereus G9842
MAKYTIVDKDTCIACGACGAAAPDIYDYDDEGIAFVTLDDNQGIVEIPDVLIEDMMDAFE
GCPTDSIKVADESFDGDALKFE

>4Fe-4SST2(BCN_1461)Bacillus cereus NC7401
MAKYTIVDKDTCIACGACGAAAPDIYDYDDEGIAFVTLDDNQGIVEIPDVLLEDMMDAFE
GCPTDSIKVADESFDGDALKFE

>4Fe-4SST2(BCQ_1552)Bacillus cereus Q1
MAKYTIVDKDTCIACGACGAAAPDIYDYDDEGIAFVTLDDNQGIVEIPDVLLEDMMDAFE
GCPTDSIKVADESFDGDALKFE

>4Fe-4SST2(ABC1844)Bacillus clausii
MKNCYTIVELDTCIACGACGLVAPDVYDYNEEGLAYAFLDHNTGNKPLPDLLVDDAIDAK
DGCPTDSIKISDKPFNGDPTKYE

>4Fe-4SST2(Bcoa_2927)Bacillus coagulans 36D1
MAKFTIVDKDTCIACGACSASAPDIFDYDDEGLSFVILDDNKGTAEVPEILEDDLMDAFE
GCPTESIKVSDKPFDGDPLKYE

>4Fe-4SST2(Bcer98_1206)Bacillus cytotoxicus
MAKYTIVDKDTCIACGACGAAAPDIYDYDDEGIAFVTLDDNQGIVEVPDVLIEDMMDAFE
GCPTDSIKVADETFDGDALKFE

>4Fe-4SST2(BC359_05935)Priestia flexa

MPKYTIVDKDTCIACGACGAAAPDIYDYDDEGIAFVTLDDNQGIVEVPDELEDDMMDAFE
GCPTDSIRVADEKFDGDSNKFE

>4Fe-4SST2(BH0209)Bacillus halodurans

MPIFTIVDQDTCIACGACGASAPDIFDYDDEGLAFYIADHNQGNTPIDPSLLNDLEDAVE
GCPTGSIKLAEEAFNGELDKGQ

>4Fe-4SST2(BH1605)Bacillus halodurans

MPKYTIVDKDTCIACGACGAAAPDIYDYDDEGIAFVTLDDNEGTVEVPDELLEDMIDAQE
GCPTDSIKVADEPFDGDALKFE

>4Fe-4SST2(BleG1_2074)Bacillus lehensis

MTNKCFTIVDMDTCIACGACGAVAPDIYDYNDEGLAYVYIDDNTGSALIPDILVDDVIDA
KNGCPTDSIKIASAPFDGDPMKYE

>4Fe-4SST2(BMD_0889)Bacillus megaterium DSM 319

MAFTMVDKETCIACGACGGSAPDLYDYDSEGLAYAVLDNNEGTKEVPEHQQDEMMEAYEG
CPTDSIKVAEQPFYGDATKFE

>4Fe-4SST2(BMD_2329)Bacillus megaterium DSM 319

MGKYTIVDKETCIACGACGAAAPDIYDYDDEGIAFVILDDNQGTAEVPQELEDDLIDAFE
GCPTESIRIADEKFDGDSNKFQ

>4Fe-4SST2(BG04_3184)Bacillus megaterium NBRC 15308 = ATCC 14581
MAFTMVDKETCIACGACGGSAPDLYDYDSEGLAYAVLDNNEGTKEVPEHQQDEMMEAYEG
CPTESIKVAGQPFYRDATKFE

>4Fe-4SST2(BG04_4740)Bacillus megaterium NBRC 15308 = ATCC 14581
MGKYTIVDKETCIACGACGAAAPDIYDYDDEGIAFVILDDNQGTAEVPQELEDDLIDAFE
GCPTESIRIADEKFDGDSNKFQ

>4Fe-4SST2(BMQ_0889)Bacillus megaterium QM B1551
MAFTIVDKETCIACGACGGSAPDLYDYDSEGLAYAVLDNNEGTKEVPEHQQDEMMEAYEG
CPTESIKVAEQPFYGDAQKFE

>4Fe-4SST2(BMQ_2367)Bacillus megaterium QM B1551
MGKYTIVDKETCIACGACGAAAPDIYDYDDEGIAFVILDDNQGTVEVPQELEDDLIDAFE
GCPTESIRIADEKFDGDSNKFQ

>4Fe-4SST2(BG05_2297)Bacillus mycoides ATCC 6462
MLHYTVVNKETCESCGLCRSIAPDIYQLDCKSKAFGALDHNEGIIGIPTTFLNAMVAAYE
SCPTNSIRVGKESFENQKLENGMRMD

>4Fe-4SST2(BG05_4411)Bacillus mycoides ATCC 6462
MAKYTIVDKDTCIACGACGAAAPDIYDYDDEGIAFVTLDDNQGIVEIPDVLIEDMMDAFE
GCPTDSIKVADESFDGDSLKFE

>4Fe-4SST2(BcerKBAB4_1405)Bacillus mycoides KBAB4
MAKYTIVDKDTCIACGACGAAAPDIYDYDDEGIAFVTLDDNQGIVEIPDVLIEDMMDAFE
GCPTDSIKVADESFDGDSLKFE

>4Fe-4SST2(bwei_3511)Bacillus mycoides WSBC 10204
MAKYTIVDKDTCIACGACGAAAPDIYDYDDEGIAFVTLDDNQGIVEIPDVLIEDMMDAFE
GCPTDSIKVADESFDGDSLKFE

>4Fe-4SST2(A361_19670)Bacillus oceanisediminis
MAKYTIVDKETCIACGACGAAAPDIYDYDDEGIAFVTLDDNQGIVEIPDVLVDDMMDAFE
GCPTDSIKVADDAFDGDPLKYE

>4Fe-4SST2(BpOF4_15190)Bacillus pseudofirmus
MAKYTIVDKDTCIACGACGAAAPDIYDYDDEGIAFVILDDNQGTAEIPEELEEDMIDAQE
GCPTDSIKVADESFDGDALKFE

>4Fe-4SST2(DJ92_4130)Bacillus pseudomycoides 219298
MAKYTIVDKDTCIACGACGAAAPDIYDYDDEGIAFVTLDDNQGIVEIPDVLIDDMMDAFE
GCPTDSIKVADESFDGDALKFE

>4Fe-4SST2(BW16_11095)Bacillus pumilus MTCC B6033
MAKYTIVDKDTCIACGACGAAAPDIYDYDDEGIAFVTLDDNQGTVEVPEVLEEDMLDAFE
GCPTDSIKVADETFDGDPLKHE

>4Fe-4SST2(BPUM_2037)Bacillus pumilus SAFR-032
MAKYTIVDKDTCIACGACGAAAPDIYDYDDEGIAFVTLDDNQGTVEVPEVLEEDMLDAFE
GCPTDSIKVADESFEGDPLKHE

>4Fe-4SST2(UP12_10365)Bacillus pumilus SH-B9
MAKYTIVDKDTCIACGACGAAAPDIYDYDDEGIAFVTLDDNQGTVEVPEVLEEDMLDAFE
GCPTDSIKVADESFEGDPLKHE

>4Fe-4SST2(UP17_19660)Peribacillus simplex
MAKYTWVDKETCIACGACGSTGPEIYDYDDQGIAFVILDDNQGIAEVPEILHDDMQDAME
GCPTESILIQDEPFSW

>4Fe-4SST2(BSM4216_2393)Bacillus smithii
MAKYTIVDKDTCIACGACGAAAPDIYDYDDEGLAFVVLDDNQGTAEVPEVLLDDMQDAFE
GCPTDSIKVADEPFDGDPLKFE

>4Fe-4SST2(OXB_2318)Bacillus sp. OxB-1
MSKKYTIVDQDTCIACGACGAAAPDIYDYDDEGIAYVILDDNTGTTAVPEELMDDLEDAF
DGCPTDSIKMADEPFDGDPLKYE

>4Fe-4SST2(QR42_10320)Bacillus sp. WP8
MAKYTIVDKDTCIACGACGAAAPDIYDYDDEGIAFVTLDDNQGTVEVPEVLEEDMLDAFE
GCPTDSIKVADESFEGDPLKHE

>4Fe-4SST2(BMB171_C1316)Bacillus thuringiensis BMB171

MAKYTIVDKDTCIACGACGAAAPDIYDYDDEGIAFVTLDDNQGIVEIPDVLIEDMMDAFE
GCPTDSIKVADESFDGDALKFE

>4Fe-4SST2(BTB_c15230)Bacillus thuringiensis Bt407

MAKYTIVDKDTCIACGACGAAAPDIYDYDDEGIAFVTLDDNQGIVEIPDVLIEDMMDAFE
GCPTDSIKVADESFDGDALKFE

>4Fe-4SST2(BF38_2701)Bacillus thuringiensis HD1011

MAKYTIVDKDTCIACGACGAAAPDIYDYDDEGIAFVTLDDNQGIVEIPDVLLEDMMDAFE
GCPTDSIKVADESFDGDALKFE

>4Fe-4SST2(BTG_13330)Bacillus thuringiensis HD-771

MAKYTIVDKDTCIACGACGAAAPDIYDYDDEGIAFVTLDDNQGIVEIPDVLIEDMMDAFE
GCPTDSIKVADESFDGDALKFE

>4Fe-4SST2(BTF1_05115)Bacillus thuringiensis HD-789

MAKYTIVDKDTCIACGACGAAAPDIYDYDDEGIAFVTLDDNQGIVEIPDVLIEDMMDAFE
GCPTDSIKVADESFDGDALKFE

>4Fe-4SST2(MC28_0717)Bacillus thuringiensis MC28

MAKYTIVDKDTCIACGACGAAAPDIYDYDDEGIAFVTLDDNQGIVEIPDVLIEDMMDAFE
GCPTDSIKVADESFDGDALKFE

>4Fe-4SST2(CT43_CH1410)Bacillus thuringiensis serovar chinensis CT-43
MAKYTIVDKDTCIACGACGAAAPDIYDYDDEGIAFVTLDDNQGIVEIPDVLIEDMMDAFE
GCPTDSIKVADESFDGDALKFE

>4Fe-4SST2(YBT020_08060)Bacillus thuringiensis serovar finitimus YBT-020
MAKYTIVDKDTCIACGACGAAAPDIYDYDDEGIAFVTLDDNQGIVEIPDVLLEDMMDAFE
GCPTDSIKVADESFDGDALKFE

>4Fe-4SST2(BTK_08700)Bacillus thuringiensis serovar kurstaki HD-1
MAKYTIVDKDTCIACGACGAAAPDIYDYDDEGIAFVTLDDNQGIVEIPDVLIEDMMDAFE
GCPTDSIKVADESFDGDALKFE

>4Fe-4SST2(HD73_1711)Bacillus thuringiensis serovar kurstaki HD73
MAKYTIVDKDTCIACGACGAAAPDIYDYDDEGIAFVTLDDNQGIVEIPDVLIEDMMDAFE
GCPTDSIKVADESFDGDALKFE

>4Fe-4SST2(YBT1520_08515)Bacillus thuringiensis serovar kurstaki YBT-1520
MAKYTIVDKDTCIACGACGAAAPDIYDYDDEGIAFVTLDDNQGIVEIPDVLIEDMMDAFE
GCPTDSIKVADESFDGDALKFE

>4Fe-4SST2(H175_ch1427)Bacillus thuringiensis serovar thuringiensis IS5056
MAKYTIVDKDTCIACGACGAAAPDIYDYDDEGIAFVTLDDNQGIVEIPDVLIEDMMDAFE
GCPTDSIKVADESFDGDALKFE

>4Fe-4SST2(YBT1518_08435)Bacillus thuringiensis YBT-1518

MAKYTIVDKDTCIACGACGAAAPDIYDYDDEGIAFVTLDDNQGIVEIPDVLIEDMMDAFE
GCPTDSIKVADESFDGDALKFE

>4Fe-4SST2(AQ980_22395)Bacillus thuringiensis YWC2-8

MAKYTIVDKDTCIACGACGAAAPDIYDYDDEGIAFVTLDDNQGIVEIPDVLIEDMMDAFE
GCPTDSIKVADESFDGDALKFE

>4Fe-4SST2(Btoyo_4121)Bacillus toyonensis

MAKYTIVDKDTCIACGACGAAAPDIYDYDDEGIAFVTLDDNQGIVEIPDVLIEDMMDAFE
GCPTDSIKVADESFDGDALKFE

>4Fe-4SST2(BK049_16745)Bacillus xiamenensis

MAKYTIVDKDTCIACGACGAAAPDIYDYDDEGIAFVTLDDNQGTVEVPEVLEEDMLDAFE
GCPTDSIKVADESFDGDPLKHE

>4Fe-4SST2(BBR47_24240)Brevibacillus brevis

MGGETTMTTWVDKDTCIACGACGATAPDVFDYDDEGLAFNKLDDNANSVEIPDILHDDVR
DAAEGCPTDSIKVE

>4Fe-4SST2(BRLA_c018560)Brevibacillus laterosporus

MTTWVDKDTCIACGACGATAPDVFDYDDEGLAFNTIDDNTNTAEIPEILHDDVRDAADGC
PTDSIKIDG

>4Fe-4SST2(EGCR1_03845)Enterococcus gilvus

MKCEIIPERCIACGLCQTIAPEIFDYTDDGLVLFVGEPEATHEFIPESQQDAVIQSAKRC
PSHAILYQNS

>4Fe-4SST2(EAT1b_2973)Exiguobacterium sp. AT1b

MAKFTIVDKDTCIACGACGAAAPDIYDYDDEGLAFNLMDDNTGTVEIPDELYEDMMDAFE
GCPTDSIKVADESFDGDALKFE

>4Fe-4SST2(HBHAL_3321)Halobacillus halophilus

MAKYTMVDQETCIACGACGAAAPDIYDYDDEGIAYSVLDDNQGVSEVPEVWEEDMEDAFD
GCPTDSIKIAKSPFEGNAHKHE

>4Fe-4SST2(HM131_10360)Halobacillus mangrovi

MAKFTMVDQDTCIACGACGAAAPDIYDYDDEGIAYSILDQNKGNYEVPDILEEDMEDAFE
GCPTDSIKVADEPFDGDPLKHE

>4Fe-4SST2(BW727_100650)Jeotgalibaca dankookensis

MYYTKIDRERCIACGLCQLIAPDIYEYDENGIAYTKKDNNTGTYPLAKEDTLPFKKAYRS
CPTGAIMRSDTPFS

>4Fe-4SST2(EJN90_12010)Jeotgalibaca ciconiae

MHYTKVNREICIACGLCQLIAPSLYEYDKDGIAYTVKDQNKGTMPIQEQEFESFKKAYTS
CPTGAILRSTAPFEDLDTQS

>4Fe-4SST2(C1X05_07340)Laceyella sacchari

MRTWVDKDTCIACGACGATAPDVYDYDEDGIAYVILDDNTGTADVPEELHDDVRDAQEGC
PTDSIKVEE

>4Fe-4SST2(AOX59_04795)Lentibacillus amyloliquefaciens

MAKYTIIDQETCIACGACGTTAPDIYDYNDEALAYAVLDENEGAREVPGELEDDVLDAFE
GCPTDSVKVADKPFHGDPLKFET

>4Fe-4SST2(BHM04_03415)Macrococcus sp. IME1552

MAGKYTIVDMDTCIACGACGAAAPDIYDYDDEGIAYVILDDNKGTQEIPEELEEDMIDAF
EGCPTDSIKVADESFDGDALKFE

>4Fe-4SST2(CUC15_10760)Oceanobacillus sp. 160

MAKFAIVDIETCIACGACGAAAPKIFSYDDEGLSYVLIDGNTGTKKISRLLVDDLYDAYE
GCPTDSIKISDIPFNKEPTNV

>4Fe-4SST2(BBH88_10145)Planococcus antarcticus

MAKYTIVDKDTCIACGACGAAAPDIYDYDDEGIAFVILDNNMGTEQVPDELEEDMEDAFE
GCPTDSIKVADTSFEGDPLKYED

>4Fe-4SST2(BBI08_05970)Planococcus halocryophilus

MAKYTIVDKDTCIACGACGAAAPDIYDYDDEGIAFVILDNNMGTEQVPDELEEDMEDAFE
GCPTDSIKVADTSFEGDPLKYED

>4Fe-4SST2(BBI11_10285)Planococcus maritimus

MAKYTIVDKDTCIACGACGAAAPDIYDYDDEGIAFVILDDNTGTEQVPDELMEDMEDAFE
GCPTDSIKVADEAFEGDPLKYED

>4Fe-4SST2(BBI15_04830)Planococcus plakortidis

MAKYTIVDKDTCIACGACGAAAPDIYDYDDEGIAFVILDDNTGTEQVPDELMEDMEDAFE
GCPTDSIKVADEPFEGDPLKYED

>4Fe-4SST2(AUC31_00565)Planococcus rifietoensis

MAKYTIVDKDTCIACGACGAAAPDIYDYDDEGIAFVILDDNTGTEQVPDELMEDMEDAFE
GCPTDSIKVSDEPFEGDPLKYED

>4Fe-4SST2(ATY39_01175)Rummeliibacillus stabekisii

MPKYTIVDKETCIACGACEASAPDIFDYDEEGISHVIFDDNEGRVEVPEYLLEDLEDAVE
GCPTDSIKVESEPFFAVKS

>4Fe-4SST2(ATY39_03665)Rummeliibacillus stabekisii

MAKFTIVDQDTCIACGACGAAAPDIYDYDDEGIAFVILDDNTGTAVVPEDLMEDMEDAFE
GCPTDSIKVAEESFEGDALKYE

>4Fe-4SST2(AAV35_006010)Salimicrobium jeotgali

MKLYSIVDKDTCIACGACGMAAPELYNYDDEGLAYFLPDDNQGNTPIAEELELDAEDAYE
GCPTGSIKLANSNFNGSIPEED

>4Fe-4SST2(SOLI23_04880)Solibacillus silvestris DSM 12223
MPKYTIVDKDTCIACGACGAAAPDIYDYDDEGIAFVILDDNMGTAEVPEDLLEDMQDAFE
GCPTDSIKVADETFDGDSLKFE

>4Fe-4SST2(SSIL_2743)Solibacillus silvestris StLB046
MPKYTIVDKDTCIACGACGAAAPDIYDYDDEGIAFVILDDNMGTAEVPEDLLEDMQDAFE
GCPTDSIKVADETFDGDSLKFE

>4Fe-4SST2(SporoP33_15000)Sporosarcina sp. P33
MSSKKYTIVDQDTCIACGACGAAAPDIYDYDDEGIAFVILDDNTGNTEVPEELMEDLEDA
FDGCPTDSIKIADEPFEGDPLKYED

>4Fe-4SST2(SporoP37_10355)Sporosarcina sp. P37
MAQYTMVERSTCIACGACNPVAPELFDYTADGFAFALLDDNEGITEVPEDLIEDLEDAFE
GCPTNSIKMADAPFSCQGIEAS

>4Fe-4SST2(SporoP37_15335)Sporosarcina sp. P37
MSSKKYTIVDQDTCIACGACGAAAPDIYDYDDEGIAFVILDDNTGNTEVPEELMEDLEDA
FDGCPTDSIKIADEPFEGDPLKYED

>4Fe-4SST2(SporoP32a_15650)Sporosarcina ureae
MSSKKYTIVDQDTCIACGACGAAAPDIYDYDDEGIAFVILDDNTGNTEVPEELMEDLEDA
FDGCPTDSIKIADEAFEGDPLKYED

>4Fe-4SST2(DP17_133)Staphylococcus epidermidis SEI
MAKYTIVDMDTCIACGACGAAAPDIYDYDDEGIAYVILDDNQGTAEVPEELYEDMEDALE
GCPTDSIKIEDEPFDGDALKFE

>4Fe-4SST2(SH1428)Staphylococcus haemolyticus JCSC1435
MAKYTIVDMDTCIACGACGAAAPDIYDYDDEGIAFVILDDNQGTAEVPEELYEDMEDALE
GCPTDSIKIEEEPFDGDALKFE

>4Fe-4SST2(B5P37_04475)Staphylococcus lutrae
MDCYACVDREMCISCAACGATAPHLFKYDADDVAYMCLDQNSGTCPIPEAEIDHLNDAVE
NCPTAAVMVSQTPFFNAQHTI

>4Fe-4SST2(SPSE_0307)Staphylococcus pseudintermedius ED99
MMSYYAYVDRDMCIACSACGAAAPRLFRYDAQGIAYMCLDCNSGTAQIPECDLGNLDDAV
ENCPTSAITVGQQPFSLSSIILDREKEL

>4Fe-4SST2(TEH_20080)Tetragenococcus halophilus NBRC 12172
MSRLCKIVPERCIACGLCALYAPEVFDYDDNGVVLFSQEPKAYQQFIAVKDQEDVIKAYK
RCPVRAILLEK

>4Fe-4SST2(C7K43_01150)Tetragenococcus koreensis
MSRLCKIVPERCIACGLCALYAPEIFDYDDNGIVLFAQEPEANQQFIPAKDQGAVIEAYK
QCPVRAILLEK

>4Fe-4SST2(BME96_09575)Virgibacillus halodenitrificans

MSKFTAVDQETCIACGACGINAPDIFDYDDEGIAFSLLDSNSGTVIVPEHLEEDLIDAWQ
GCPTDSVKVSEAPFKNNLKCVK

>4Fe-4SST2(CFK40_10905)Virgibacillus necropolis

MAKYTIVDKETCIACGACGAAAPDIYDYDDEGLAYVVIDDNTGAAEIPEVFEEDMVDAFE
GCPTDSIKISDKKFNGDPLRFEDE

>4Fe-4SST2(CFK37_18560)Virgibacillus phasianinus

MVKYAIIDQDTCIGCGNCEGIAPDIFDLDDEGLAFVKQDQNRGYTPIPESKLDELEEAIE
ECPTDSIKVSDQPFGKQE

>4Fe-4SST2(X953_10705)Virgibacillus sp. SK37

MPKFTAVDQETCIACGACGIHAPDIFDYDDEGIAFSFLDGNTGTLIVPEHLEDDLMDAWQ
GCPTDSIKVSETPVKNNLKCFK

Subtype 3

>4Fe-4SST3(VN24_00375)Paenibacillus beijingensis

MAKYSWVDKDTCIACGACGATAPDIYDYDDEGLAEVIYDGDGNHGVKAIAEDLYDDLQDA
CDGCPTDSIKIADEPFNR

>4Fe-4SST3(AR543_10490)Paenibacillus bovis

MSKYTWVDKDTCIACGACGATAPDIYDYDDEGLAEVIFDGDNNRGVMAIPDDMFEDMQDA
CDGCPTDSIHIEDAPFNKEA

>4Fe-4SST3(B9T62_07605)Paenibacillus donghaensis
MAKYTWVEKDTCIACGACGATAPDIFDYDDEGLAEVIYENDANHGTTVIPDDLFDDLQDS
ADGCPTDSIKIADTPFNKEG

>4Fe-4SST3(BBD41_22470)Paenibacillus ihbetae
MAKYTWVDKDTCIACGACGATAPDIYDYDDEGLAEVIFNGDNNRGVTEIPEDMHDDMLDA
CDGCPTDSIRIADEPFNAE

>4Fe-4SST3(ERIC2_c12060)Paenibacillus larvae
MAKYTWVEKDTCIACGACGATAPDIYDYDDEGLAEVIYEGDNNQGITEIPEDLYDDLQDA
QDGCPTDSIKVADTPFN

>4Fe-4SST3(D5F53_07245)Paenibacillus lautus
MAKFTWVDKDTCIACGACGATAPDIYDYDDEGLAEVIFDGDNNRGVTEIPEDMHDDMLDA
CDGCPTDSIRIADEPFNAE

>4Fe-4SST3(PM3016_1936)Paenibacillus mucilaginosus 3016
MAKYTIVDKETCIACGACGATAPDIYDYDDEGLAEVIYEADGNKGVTEIPEDLYDDLQDA
QDGCPTDSIKVADTPFA

>4Fe-4SST3(B2K_09820)Paenibacillus mucilaginosus K02

MAKYTIVDKETCIACGACGATAPDIYDYDDEGLAEVIYEADGNKGVTEIPEDLYDDLQDA
QDGCPTDSIKVADTPFA

>4Fe-4SST3(KNP414_01631)Paenibacillus mucilaginosus KNP414
MAKYTIVDKETCIACGACGATAPDIYDYDDEGLAEVIYEADGNKGVTEIPEDLYDDLQDA
QDGCPTDSIKVADTPFA

>4Fe-4SST3(IJ22_06430)Paenibacillus naphthalenovorans
MAKYTWVDKETCIACGACGATAPDIYDYDDEGLAEVIYDNDGNKGVTEIPEDLYDDLQDA
QDGCPTDSIKVADAPFNK

>4Fe-4SST3(ABE82_09145)Paenibacillus peoriae
MAKYTWVEKDTCIACGACGATAPDIYDYDDEGLAEVIFEGDANQGIKAISDDLFDDMQDA
CDGCPTDSIKVADEPFNKEG

>4Fe-4SST3(X809_09305)Paenibacillus polymyxa CR1
MAKYTWVEKDTCIACGACGATAPDIYDYDDEGLAEVIFEGDANQGIKAISDDLFDDMQDA
CDGCPTDSIKVADEPFNKEG

>4Fe-4SST3(PPM_1723)Paenibacillus polymyxa M1
MAKYTWVEKDTCIACGACGATAPDIYDYDDEGLAEVIFEGDANQGIKAISEDLFDDMQDA
CDGCPTDSIKVADEPFNKEG

>4Fe-4SST3(RE92_03245)Paenibacillus polymyxa Sb3-1
MAKYTWVEKDTCIACGACGATAPDIYDYDDEGLAEVIFEGDANQGIKAISEDLFDDMQDA
CDGCPTDSIKVADEPFNKEG

>4Fe-4SST3(PPSC2_09050)Paenibacillus polymyxa SC2
MAKYTWVEKDTCIACGACGATAPDIYDYDDEGLAEVIFEGDANQGIKAISEDLFDDMQDA
CDGCPTDSIKVADEPFNKEG

>4Fe-4SST3(PPSQR21_017990)Paenibacillus polymyxa SQR-21
MAKYTWVEKDTCIACGACGATAPDIYDYDDEGLAEVIFEGDANQGIKAISEDLFDDMQDA
CDGCPTDSIKVADEPFNKEG

>4Fe-4SST3(PRIO_2569)Paenibacillus riograndensis
MAKYTWVEKDTCIACGACGATAPDIFDYDDEGLAEVIYENDGNHGVTAIPDDLFDDLQDS
ADGCPTDSIKIADAPFNKEG

>4Fe-4SST3(IJ21_35670)Paenibacillus sp. 32O-W
MAKFTWVDKDTCIACGACGATAPDIYDYDDDGLAEVIYGGDNNRGVTEIPEDLYDDLQDA
ADGCPTDSIKIADEPFDN

>4Fe-4SST3(UB51_14140)Paenibacillus sp. IHBB 10380
MAKYTWVEKDTCIACGACGATAPDIYDYDDEGLAEVIYENDGNHGVTDIPEDLFDDLQDA
CDGCPTDSIKVADTPFNKEG

>4Fe-4SST3(GYMC10_4591)Paenibacillus sp. Y412MC10
MAKFTWVDKDTCIACGACGATAPDIYDYDDEGLAEVIFDGDNNRGVTEIPEDMHDDMLDA
CDGCPTDSIRIADEPFNAE

>4Fe-4SST3(SY83_20565)Paenibacillus swuensis
MAKYTWVEKETCIACGACGATAPDIYDYDDEGLAEVIYNADANHGNTEIPEDLFDDLQDA
ADGCPTDSIKIAETAFNKEG

>4Fe-4SST3(BS614_14185)Paenibacillus xylanexedens
MSKYTWVEKDTCIACGACGATAPDIYDYDDEGLAEVIFDGDANHGVKAIPDDLFDDMQDA
CDGCPTDSIKVADEPFNKEG

>4Fe-4SST3(B9T62_05055)Paenibacillus donghaensis
MNNYASVNQDDCISCGACNSAAPDIFDLDNSGIAGVIYEGDSNRGVTAIAADLQEELQEA
YDNCPTQCIQLAAAPFA

>4Fe-4SST3(PRIO_2701)Paenibacillus riograndensis
MNSFASVNQESCIACGACNSAAPDIFDLDYNGIAGVIYEGDKNRGITAIEPELLEELQEA
FVSCPTHCIQLAAVPFA

Subtype 4

>4Fe-4SST4(CA_C3621)Clostridium acetobutylicum ATCC 824
MKAFVDKETCIGCGTCPAICPEIFEMEDDGKAVASDAEIANDLKESAQDAAESCPVDAIM

VR

>4Fe-4SST4(SMB_G3662)Clostridium acetobutylicum DSM 1731
MKAFVDKETCIGCGTCPAICPEIFEMEDDGKAVASDAEIANDLKESAQDAAESCPVDAIM
VR

>4Fe-4SST4(CEA_G3628)Clostridium acetobutylicum EA 2018
MKAFVDKETCIGCGTCPAICPEIFEMEDDGKAVASDAEIANDLKESAQDAAESCPVDAIM
VR

>4Fe-4SST4(Clopa_0518)Clostridium pasteurianum BC1
MKGFVDKDTCIGCGLCTSICPEVFIMDDKGKAERSKNEILETLVASAQEAATECPVNAIT
VE

>4Fe-4SST4(Closa_2589)Clostridium saccharolyticum WM1
MKATVDRDGCIECGLCASICPEVFRMGDDGPAEAYVDEVPESAEQTAVEAQEGCPVSVIT
VE

>4Fe-4SST4(CLSPO_c01150)Clostridium sporogenes
MKIEIDKDKCIGCGLCRDIGDGLFKIGEDGKAESTIDPVPIMKEQYGKEGEYVCPVNAIR
TII

Subtype 5

>4Fe-4SST5(CB4_02945)Aneurinibacillus soli

MAKTYVDQETCIACEACYSTCPGVYESDDDGYSFVKLPGGLKDFVEVPKEFVSKAMSARD

ECPTESIRWID

>4Fe-4SST5(CVV65_03845)Kyrpidia spormannii

MPKTWVERETCIACGACSAACPEVYGEDEDGFAFVKLPGGREGFVEIPEEYVADARDAFE

GCPSESVKWQED

Subtype 6

>4Fe-4SST6(H04402_03280)Clostridium botulinum H04402 065

MKAYVDKDTCVGCGLCPSICSEVFSMQDDGKAHVIVENENVPEELTHLAEEARDSCPVAA

IEVE

7Fe-8S

Subtype 1

>7Fe-8SST1(Aaci_1902)Alicyclobacillus acidocaldarius subsp. acidocaldarius DSM 446

MPFVITSPCIGEKAADCVETCPVDAIHEGPDQYYIDPDLCIDCAACEPVCPVNAIYQEEF

VPEDEKEFIEKNRNFFRNR

>7Fe-8SST1(TC41_2002)Alicyclobacillus acidocaldarius subsp. acidocaldarius Tc-4-1

MPFVITSPCIGEKAADCVETCPVDAIHEGPDQYYIDPDLCIDCAACEPVCPVNAIYQEEF

VPEDEKEFIEKNRNFFRNR

>7Fe-8SST1(ABC3176)Bacillus clausii

MSFVILSPCIGEKAGECAEVCPVDCIEEGDDQYFINPDICIDCGACQGVCPVDAIVEEYE

MSKEDEPFLKKAEAFFGVE

>7Fe-8SST1(ABC2655)Bacillus clausii

MAFVILSPCIGEKAGECVDVCPVDCIEEGEDQYFINPDICIDCGACQGVCPVDAIVEEYE

MAPEDQKFLKKAEEFFGIE

>7Fe-8SST1(BleG1_2647)Bacillus lehensis

MAFVILSPCIGEKAGECVDVCPVDCIEEGEDQYYINPDICIDCGACQGVCPVDAIVEEYE

MAPEDEKYLKKAEAFFGVE

>7Fe-8SST1(HBHAL_3202)Halobacillus halophilus

MAFVITSPCKDEKAGECVDVCPVDCIEEGKDMFYIDPDICIDCGACEAVCPVEAIYIEDE

VPEEENKYIELNRKFFEEQ

>7Fe-8SST1(HBHAL_2944)Halobacillus halophilus

MAFVITSPCESEKAGECVTVCPVDCIAEGPDQFYINPDVCIDCGACVAACPVDAIVEEYE

MTPDEEPYLDKAEKFFGNI

>7Fe-8SST1(HM131_10950)Halobacillus mangrovi

MAFVITSPCKDEKAGECVDVCPVDCIEEGKDMFYIDPAICIDCGACEAVCPVEAIYIEDE

VPEEETEYIEINRKFFEEQ

>7Fe-8SST1(CVV65_08060)Kyrpidia spormannii
MAFVITSPCIDEKAAECVEVCPVDAIHEGEDQYYIDPDTCIDCGACEPVCPVSAIYQEDF
VPDDQKEFIQKNRDFFKK

>7Fe-8SST1(AOX59_08255)Lentibacillus amyloliquefaciens
MAFVITSPCLNEKSAECVEVCPVDCIEEGKDMFYIDPDVCIDCGACEAACPVEAIYMEDE
VPEKEEKYIGLNRQFFE

>7Fe-8SST1(AOX59_04390)Lentibacillus amyloliquefaciens
MAFVITSPCINEKSGECVEVCPVDCIEEGKDMFYIDPDICIDCGACEAVCPVEAIYMEDE
VPEGEEKFINLNARFFEES

>7Fe-8SST1(AOX59_18005)Lentibacillus amyloliquefaciens
MAFVILDPCRGEKSGECVTVCPVDCIEEGPEQFYIDPDICIDCGACVSVCPVDAIVEEYD
MTPEQEKYLDEAEEFFANR

>7Fe-8SST1(LIP_3339)Limnochorda pilosa
MAFVIAEPCIDVKDAACVEVCPVDCIHEGEDQYYIDPEECIDCGACEPECPVEAIFPEDE
VPEEWQSFVEKNANFFK

>7Fe-8SST1(OB1730)Oceanobacillus iheyensis
MAFVITSPCKTEKAGECVEVCPVDCIEEGKDMFYIEPDICIDCGACEAVCPVEAIYMEDE
VPEEENEYISLNRLFFENR

>7Fe-8SST1(OB2580)Oceanobacillus iheyensis

MAFVILDPCRGEKAGECVSVCPVDCIEEGVKQFYIDPDICIDCGACKAVCPVSAIEEEYD
LTPNQEKYLEEAEEFFANR

>7Fe-8SST1(CUC15_03725)Oceanobacillus sp. 160
MAFVITHPCTNEKAGECVSICPVDCIVEGDDQFFIDPNTCIECGACVAACPVEAIIDEHD
LTDEQEVYLEKAESFFG

>7Fe-8SST1(CUC15_14570)Oceanobacillus sp. 160
MAFVILDPCGPEKAAECVSVCPVDCIEEGPDQFYIDPDVCIDCGACKAVCPVSAIEEEYD
LTPDQEIFLEKAEEFFLNR

>7Fe-8SST1(CUC15_10385)Oceanobacillus sp. 160
MAFVITSPCKTEKAGECVEVCPVDCIEEGEDMFFIDPDICIDCGACEAVCPVTAIYMEDE
VPEEENEYIELNRLFFQGN

>7Fe-8SST1(BBD41_25865)Paenibacillus ihbetae
MYVIGSACIEEKAGECVDVCPVDCIEEGDDQFYIDTDICISCGACEAACPVAAIYYFEDL
PEDQKHYFDKAVEYYRSK

>7Fe-8SST1(AAV35_006410)Salimicrobium jeotgali
MAFVITSPCKDEKSGECVEVCPVDCIEEGKDMFYIDPAICIDCGACEAVCPVEAIYIEDE
VPEEETPYIEMNRKFFQE

>7Fe-8SST1(AAV35_008065)Salimicrobium jeotgali

MAFVITSPCMNEKSGECVDVCPVDCIEEGEDQFYINPNTCIDCGMCVMQCPVAAIVEEFD
LTPEDEQFLQKAEQFFEKR

>7Fe-8SST1(AAV35_001290)Salimicrobium jeotgali
MAFVITAPCEQEKSGECVDVCPVDCIEEGKDMFYIDPEICIDCGACEAVCPVEAIYYEED
LIDEDMPYVEKAKEFFNRK

>7Fe-8SST1(AAT16_11345)Salinicoccus halodurans
MAYVILQPCMEEKSGDCVEVCPVDCIEEGPDQFYIDPEICIDCGACVTACPVDAIVEEYE
MTPEDEPFLAKAEKFYGIK

>7Fe-8SST1(GZ22_08010)Terribacillus goriensis
MAFVITSPCEAEKAGECMEVCPVDCIAEGKDMFYIDPDICIDCGACEAVCPVEAIFMEDE
VPAEETPYIEINRKFFQEN

>7Fe-8SST1(BME96_10065)Virgibacillus halodenitrificans
MAFIITSPCKDEKAGECVEVCPVDCIEEGKDMFYIDPDICIDCGACEAVCPVEAIYMEDE
VPEEETEYIALNRKFFEEQ

>7Fe-8SST1(BME96_13815)Virgibacillus halodenitrificans
MAFVILDPCRAEKSGECVSVCPVDCIEEGKDQFYIDPDICIDCGACKAVCPVEAIEEEYD
LSPDQEVFLEKAEEFFANK

>7Fe-8SST1(CFK40_11380)Virgibacillus necropolis

MAFVITSPCKDEKSGECVEVCPVDCIEEGKDMFYIDPDICIDCGACEAVCPVEAIFMEDE
TPEQENEYIEMNRKFFEDR

>7Fe-8SST1(CFK40_06210)Virgibacillus necropolis
MAFVILDPCGPEKSAECVSVCPVDCIEEGKDQFYIDPDICIDCGACKAVCPVDAIEEEYD
LTPDQEKFLEKAEAFFANK

>7Fe-8SST1(CFK37_15150)Virgibacillus phasianinus
MAFVITSPCKDEKSGECVEVCPVDCIEEGKDMFFIDPDICIDCGACEAVCPVEAIYMEDE
TPEEENEYIEMNRKFFEDR

>7Fe-8SST1(CFK37_10865)Virgibacillus phasianinus
MAFVILDPCGPEKSAECVSVCPVDCIEEGKDQFYIDPDICIDCGACKAVCPVDAIEEEYD
LSPDQEKFLEKAEAFFADK

>7Fe-8SST1(X953_11215)Virgibacillus sp. SK37
MAFIITSPCKDEKAGECVEVCPVDCIEEGKDMFYIDPDICIDCGACEAVCPVEAIYMEDE
VPEEETEYIALNRKFFEEQ

>7Fe-8SST1(X953_14470)Virgibacillus sp. SK37
MAFVILDPCRAEKSGECVSVCPVDCIEEGKDQFYIDPDICIDCGACKAVCPVEAIEEEYD
LSPDQEVFLEKAEEFFANK

2[4Fe-4S]

Subtype 9

>2[4Fe-4S]ST9(B6259_00210)Ruminococcaceae bacterium CPB6

MAYQISSDCISCGACAAECPVNAISEGDGKFVIDENACISCGSCAGVCPVGAPQEG

>2[4Fe-4S]ST9(Closa_0313)Clostridium saccharolyticum WM1

MARVISDACVSCGSCEAECPVSAISQGDSQFVIDADTCIDCGACEGVCPTGAISEA

>2[4Fe-4S]ST9(CA_C0303)Clostridium acetobutylicum ATCC 824

MAYKITDACVSCGSCASECPVSAISQGDTQFVIDADTCIECGNCANVCPVGAPVQE

>2[4Fe-4S]ST9(SMB_G0309)Clostridium acetobutylicum DSM 1731

MAYKITDACVSCGSCASECPVSAISQGDTQFVIDADTCIECGNCANVCPVGAPVQE

>2[4Fe-4S]ST9(CEA_G0312)Clostridium acetobutylicum EA 2018

MAYKITDACVSCGSCASECPVSAISQGDTQFVIDADTCIECGNCANVCPVGAPVQE

>2[4Fe-4S]ST9(CPAST_c01220)Clostridium pasteurianum DSM 525 = ATCC 6013

MAYKIADSCVSCGACASECPVNAISQGDSIFVIDADTCIDCGNCANVCPVGAPVQE

Subtype 15

>2[4Fe-4S]ST15(PM3016_3401)Paenibacillus mucilaginosus 3016
MNMSAKAVGQRSEASVIIDRDKCIGCDICVQVCPMGILALDKDGKAYMKYDECWYCTPCQ
TDCPVDAVRVNLPYLVR

>2[4Fe-4S]ST15(B2K_17615)Paenibacillus mucilaginosus K02
MNMSAKAVGQRSEASVIIDRDKCIGCDICVQVCPMGILALDKDGKAYMKYDECWYCTPCQ
TDCPVDAVRVNLPYLVR

>2[4Fe-4S]ST15(KNP414_03179)Paenibacillus mucilaginosus KNP414
MNMSAKAVGQRSEASVIIDRDKCIGCDICVQVCPMGILALDKDGKAYMKYDECWYCTPCQ
TDCPVDAVRVNLPYLVR

>2[4Fe-4S]ST15(IJ21_03760)Paenibacillus sp. 32O-W
MPNLSARSVVQRTEASVIIDRDKCIGCDICVQVCPMGILALDEEGKAYMKYDECWYCTPC
QTDCPVDAVRVNIPYLVR

Subtype 3

>2[4Fe-4S]ST3(B2K_20440)Paenibacillus mucilaginosus K02
MIEVVSSERCVECGLCVKVCPTNVFDAGPTGLPVIARQEDCQTCFICEAYCPADALYVSP
LADENEGVREEDLAAAGTLGSWRRTIGWGPGRTKLAAVDRTPFIDRILPPQSRRSS

>2[4Fe-4S]ST3(IJ22_07520)Paenibacillus naphthalenovorans
MIEWVSADRCVQCGICVKVCPTHVFDLGPDDTPVIARQEDCQTCYICEAYCPADALYVAP

YADEKVKVEEQELIASGILGSWRKTIGWGPGRTKLAAMDQTPFIDRILPANFNDKP

Table S3. Ferredoxin protein sequences identified by datamining of published articles and Protein Data Bank (PDB). Ferredoxins were sorted as per their Fe-S cluster type and subtypes. The name of the ferredoxins includes their nomenclature name as described in the methodology followed by protein ID (either GenBank accession number or PDB ID) database and species name. Cysteine amino acids that binds to the Fe-atom of Fe-S cluster is highlighted in the sequences. In case of two Fe-S clusters, cysteine amino acids highlighted with different colors representing their binding to different Fe-S clusters.

2Fe-2S

Subtype 1

Archaea

Bacteria

>2Fe-2SST1(3LXF)Novosphingobium aromaticivorans
TAILVTTRDGTRTEIQAEPGLSLMEALRDAGIDELLALCGGCCSCATCHVLVAPAFADRLPALSGDENDLLDSSDHRTPHSRLSCQITINDKLEGLEVEIAPED
>2Fe-2SST1(mmi:MMAR_3155)Mycobacterium marinum M
MAVVTFVSHGGEKYEAPLEEGQSLMRVATNNAVPGIDGDCGGEAACGTCHVIVDPQWSDRVGLSGANEEEMLAMNPERQPTSRLSCQMQVSEAWDGLIVHLPEFQL

Eukaryota

>2Fe-2SST1(AAL95709.1)Giardia intestinalis

MSLLSSIRRFITFRVVQQGVEHTVSGAVGQSLLDAIKAAHIPIQDACEGHLGCGTCGVYLDKKTYKRIPRATKEEAVLLDQVPNPKPTSRLSCAVKLSSMLEGATVRIPSFNKNVL
SESDILASEEKKRHGQH
>2Fe-2SST1(2WLB)Schizosaccharomyces Pombe
GTGIKVFFVTPEGREIMIEGNEGDSILDLAHANNIDLEGACEGSVACSTCHVIVDPEHYELLDPPEEDEEDMLDLAFGLEETSRLGCQVLLRKDLDGIRVRIP
>2Fe-2SST1(2MJD)Saccharomyces cerevisiae
GEELKITFILKDGSQKTYEVCEGETILDIAQGHNLDMEGACGGSCACSTCHVIVDPDYYDALPEPEDDENDMLDLAYGLTETSRLGCQIKMSKDIDGIRVALPQMTRNVNNNDFS
>2Fe-2SST1(2Y5C)HOMO SAPIENS
MASDVVNVVFVDRSGQRIPVSGRVGDNVLHLAQRHGVDLEGACEASLACSTCHVYVSEDHLDLLPPPEEREDDMLDMAPLLQENSRLGCQIVLTPELEGAEFTLPKITR

>2Fe-2SST1(3P1M)Homo sapiens (9606)
MSSSEDKITVHFINRDGETLTTKGKVGDSLLDVVVENNLDIDGFGACEGTLACSTCHLIFEDHIYEKLDAITDEENDMLDLAYGLTDRSRLGCQICLTKSMDNMTVRVPETVADAR
QSIDVGKTSAENLYFQ

>2Fe-2SST1(1AYF)Bos taurus
EDKITVHFINRDGETLTTKGKIGDSLLDVVVQNNLDIDGFGACEGTLACSTCHLIFEQHIFEKLEAITDEENDMLDLAYGLTDRSRLGCQICLTKAMDNMTVRVP

>2Fe-2SST1(2739133354)Leishmania major Friedlin V1
MHRRFILGHVLPRCMLALAPLSASRALSSAPGKVELRVKKRDGTHCHVYVPVGISLMHAL
RDVSKMDVEGTCNGAMVCATCHVKLSAASFKKIEGPSEVEEDVLARALDVEETSRLACQV
DLTPELDGLEVELPSQETDRS
>2Fe-2SST1(639624039)Trypanosoma brucei brucei 927/4 GUTat10.1
MLSATFGTLGTSCGRGFLQRQGLLTTFMGRFCHSGGGGNNQNNDSSNSGNDGSSTPGAIR
VNVTTAEGEKITFSAPSGLTLMEALRDVARVDIEAACDGTCACSTCHVILREEDFGKLTAASEDEMDMLDLAPQVTPTSRLACQVKLSKELDGITLQMPSETTNEMR

>2Fe-2SST1(2739133353)Leishmania major Friedlin V1
MRNHSLLLATGVLLRPPMALAGALYTSRVLYSIPGKVKVCARTRDGTPCDFEAPVGMSLM
HAIRDVARLEMDGACDGCAQCSTCHVYLSEACFKKLGKPSEQEQDVLDKALDLQDTSRLACQITLTPAMDGLEVVLPKNVTNLLM

>2Fe-2SST1(2740751416)Tetrapisispora blattae CBS 6284
MLSLTNCISKKSSLLWKPLTSGRLFSSLASYKYALRHSTFNDPTISLRRHPKAINFYKSQ
VRSFSSTLTTFHGHLNPPKPGEELHITFVLKDGEQKTFEVSEGDSLLDIAQAHNLDMEGA
CGGSCACSTCHVIVDPDYIDALPEPEDDENDMLDLAYGLTETSRLGCQVKMSKEVEGLRV
ALPAMTRNVSNNDFSNN
>2Fe-2SST1(639507213)Danio rerio Tuebingen
MAAAAAVRAGVNFTQRLNRISPVCRVCPLLRLNRCTGAAVRRAVDGFSAPSRRLRTSIGV
CQSEDSSAPEEDAHAQEHIVNVVYIDRSGRRIPVQARVGDNVLYLAHKHGIDLEGACEAS
LACSTCHVYVSSGHYDRLPEPEEREDDMLDMAPLLQENSRLGCQIILTPELDGMELTLPK
VTRNFYVDGHVPKPH
>2Fe-2SST1(639503139)Danio rerio Tuebingen

MAAAAAVRAGVNFTQRLNRISPVCRVCPLLRLNRCTGAAVRRAVDGFSAPSRRLRTSIGV
CQSEDSSAPEEDAHAQEHIVNVVYIDRSGRRIPVQARVGDNVLYLAHKHGIDLEXACEAS
LACSTCHVYVSSGHYDRLPEPEEREDDMLDMAPLLQENSRLGCQIILTPELDGMELTLPK
VTRNFYVDGHVPKPH

>2Fe-2SST1(2740681361)Sugiyamaella lignohabitans CBS10342
MTLRLISKLASPSALRRCALASSKSQAFGTASLLQRSAIRNAVSGPGGNIYTARSLHTTA
IAHHGHVHKPNPGEELHVTFITKEGEQIQLEVAEGDNLLDIAQAHNMDMEGACGGSCACS
TCHVIVDPDYYDKMEEPDDDENDMLDLAFGLTETSRLGCQIKMTKELDGVRVALPAMTRN
LQARDFQ
>2Fe-2SST1(2740744706)Kazachstania africana CBS 2517
MLKAVCRPNTLSRASIVLKSPNFRLLSRTMTAYQFSVRTSLNNSLVVSNSQRMHFSTAQL
LQHGHIKKPVPGEELKVTFVLKDGSQQTYDACEGETLLDIAQGHNLDMEGACGGSCACST
CHVIVDPDYYDALPEPEDDENDMLDLAYGLTETSRLGCQLKMSKDIDGIRVALPQMTRNV
SSNDFK
>2Fe-2SST1(639333365)Homo sapiens
MAASMARGGVSARVLLQAARGTWWNRPGGTSGSGEGVALGTTRKFQATGSRPAGEEDAGG
PERPGDVVNVVFVDRSGQRIPVSGRVGDNVLHLAQRHGVDLEGACEASLACSTCHVYVSE
DHLDLLPPPEEREDDMLDMAPLLQENSRLGCQIVLTPELEGAEFTLPKITRNFYVDGHVP
KPH

>2Fe-2SST1(2740596487)Ustilago bromivora UB2112
MVTPSATRTALSAATHVLRRQTLTTAPKVLTPRTIPIASSSRLFTTTPIRPHGGITRPAP
GTGVKIHFVDPKGEPLKSVEASEGDDLLSVAHEYDIDLEGACEGSIACSTCHVILEPDVY
DSLEEPCDDENDMLDLAFGLTDTSRLGCQVKVTKEQDGMKVQLPAATRNMYVDGHKAGHH
>2Fe-2SST1(2740658214)Zygosaccharomyces parabailii ATCC 60483
MFRLQRFSSRLNQVCIALRRAPPIQTYKAMPMTAGFFPLRGRVTFTQPFSTSLKLLHGHL
TPPKPGEELHVTFILRDGTQRTFEVAEGDTLLDIAQANNLEMEGACGGSCACSTCHVIVD
PDYFDALPEPSDDENDMLDLAYGLTETSRLGCQIKMTKDIDGIRVALPAMTRNVSASDFN
>2Fe-2SST1(2740651865)Zygosaccharomyces parabailii ATCC 60483
MFRLQRFSSGLNQVCIALRRASPIQTCKATPMTAGLFPLRGRVTFIQPFSTSLKLLHGHL
TPPKPGEELHVTFILRDGTQRTFEVAEGDTLLDIAQANNLEMEGACGGSCACSTCHVIVD
PDYFDALPEPSDDENDMLDLAYGLTETSRLGCQIKMTKDIDGIRVALPAMTRNVSASDFN

>2Fe-2SST1(645679239)Zygosaccharomyces rouxii CBS 732
MFRIPRINFMVNRAVLASRRAFHAQNHMRNLQQPVARRTILTHTKPFSTTSKLSHGHLTP
PKPGEELKVTFILKDGSQRTYEVAEGDSLLDIAQANNLEMEGACGGSCACSTCHVIVDPD
YFDALPESDDDENDMLDLAYGLTETSRLGCQIKMSKDIDGIRVALPAMTRNVNASDFD

>2Fe-2SST1(2740641114)Candida intermedia CBS 141442
MLSRILARQPCLPYLGQARSVNLVGLQVRSLLQQMKLTHRLASRHFQVSASALHGHIHKP
KPGEELKITFITKDGTQHTFEVAEGDNLLDIAQAYNLDMEGACGGLCACSTCHIIVDPDF
YDEIPEPDDDENDMLDLAFGLTETSRLGCQVKMSKELDGIRVALPAMTRNLQSKDFS
>2Fe-2SST1(2740648393)Candida intermedia PYCC 4715
MLSRILTRQPCLPYLGQARSVNLVGLQVRSLLQQMKLTHHLASRHFQVSASALHGHIHKP
KPGEELKITFITKDGTQHTFEVAEGDNLLDIAQAYNLDMEGACGGLCACSTCHIIVDPDF
YDEIPEPDDDENDMLDLAFGLTETSRLGCQVKMSKELDGIRVALPAMTRNLQSKDFS

>2Fe-2SST1(645669254)Candida dubliniensis CD36
MFRLIPRLKPIIPSINRISVSIKPPLPPPTSPSTPQQCHVLRAFHTSPISHHGHLKKPNP
GEELHITFITKDGKQLTYEVAEGDNILDIAQAHNLDMEGACGGSCACSTCHVIVDPEYYD
KLEEPDDDENDMLDLAFGLTETSRLGCQVKMTKELDGLRVALPAMTRNLQSRDFN
>2Fe-2SST1(2740733507)Sporisorium reilianum SRZ2
MAATLARTALTEAARQAVRRPSLAPSLALRVPTPSLRLLSTTAPRPHGGITRPAPGSGIT
IHFIDPKGEPLKTVAANEGDDLLSIAHEYDIDLEGACEGSIACSTCHVILEPDVYDSLEE
PCDDENDMLDLAFGLTDTSRLGCQVKVTKEQDGMKVQLPAATRNMYVDGHKAGHH
>2Fe-2SST1(2740773685)Saccharomyces cerevisiae YJM1078
MLKIVTRAGHTARISNIAAHLLRTSPSLLTRTTTTTRFLPFSTSSFLNHGHLKKPKPGEE
LKITFILKDGSQKTYEVCEGETILDIAQGHNLDMEGACGGSCACSTCHVIVDPDYYDALP
EPEDDENDMLDLAYGLTETSRLGCQIKMSKDIDGIRVALPQMTRNVNNNDFS
>2Fe-2SST1(638214378)Saccharomyces cerevisiae S288C
MLKIVTRAGHTARISNIAAHLLRTSPSLLTRTTTTTRFLPFSTSSFLNHGHLKKPKPGEE
LKITFILKDGSQKTYEVCEGETILDIAQGHNLDMEGACGGSCACSTCHVIVDPDYYDALP
EPEDDENDMLDLAYGLTETSRLGCQIKMSKDIDGIRVALPQMTRNVNNNDFS
>2Fe-2SST1(2740718184)Saccharomyces cerevisiae YJM1332
MLKIVTRAGHTARISNIAAHLLRTSPSLLTRTTTTTRFLPFSTSSFLNHGHLKKPKPGEE
LKITFILKDGSQKTYEVCEGETILDIAQGHNLDMEGACGGSCACSTCHVIVDPDYYDALP
EPEDDENDMLDLAYGLTETSRLGCQIKMSKDIDGIRVALPQMTRNVNNNDFS
>2Fe-2SST1(639514291)Caenorhabditis elegans Bristol N2
MSSVLRLAMRAKGFSRFLAETQAFPVKNRHFMTSSVRKTGDFEYEDPKSEDEVVNITYVL
RDGTERKIRGKVGDNVMFLAHRYDIEMEGACEASLACSTCHVYVDPAFQNKLPEPLEEED
DMLDMAPALKDNSRLGCQIVLTKELDGITVTLPTMTRNFYVDGHVPKPH
>2Fe-2SST1(2739117763)Candida albicans SC5314
MFRLIPRLKPIIPSINRISLSVKPQPSTSQHCHTLRAFHTSPISLHGHLKKPNPGEELHI
TFITKDGKQLTYEVAEGDNILDIAQAHNLDMEGACGGSCACSTCHVIVDPEYYDRLEEPD
DDENDMLDLAFGLTETSRLGCQVKMTKELDGIRVALPAMTRNLQSRDFN
>2Fe-2SST1(2740887005)Kazachstania naganishii CBS 8797

MFRLLLKNSMRPTILTVRSSGSSQLLNSSILTASRGFSRTRPRLHGHLRKPAKGEELKIS
FILKDGTSKTYEVAEGDTILDIAQGHNLDMEGACGGSCACSTCHVIVDPDYYDALQEPED
DENDMLDLAYGLTETSRLGCQVKMSKDIDGIRVALPQMTRNVNNKDFS
>2Fe-2SST1(638229415)Candida glabrata CBS 138
MQHILRRSIVPISRNIGLSRRFADRPLWQSSARLFSTSMMLSHGHIKKPNPGEELHVTYI
LKDGSQKTYEVADGDTLLDIAQANNLDMEGACGGSCACSTCHVIVDPDYYDAIPEPEDDE
NDMLDLAYGLTETSRLGCQVKMSKDIDGIRVALPAMTRNVSNNDFNQ
>2Fe-2SST1(2740687691)Kluyveromyces marxianus NBRC 1777
MLRTILPRQAVLLNQFRTGAMHSRMQMKPHTRLWTSNLIGKRFHGHLHKPKPGEELHVTF
ILKDGTQKTFEVAEGDSLLDIAQGHNLDMEGACGGSCACSTCHVIVDPEYYDALEEPDDD
ENDMLDLAYGLTETSRLGCCIKMSKDIDGIRVALPAMTRNVSSTDFE
>2Fe-2SST1(638233708)Kluyveromyces lactis NRRL Y-1140
MIRGILTRQAGHLNQLRARSITSPSISHNIWLARTNLSWKRYHGHLHKPKPGEELHVTFI
LKDGTQKTFEVAEGDSLLDIAQGHNLDMEGACGGSCACSTCHVIVDPDYYDALEEPDDDE
NDMLDLAYGLTETSRLGCCIKMNKDIDGIRVALPSMTRNVSSSDFE
>2Fe-2SST1(2740668374)Millerozyma farinosa CBS 7064
MIRSLVRITRGPSVRANIKFVPTAQRICIPFSRGFHYSAPRNHGHIKQPKPGEELKITFI
TKEGEQHTYEVAEGDNILDIAQAHNLDMEGACGGSCACSTCHIIVDPEYYDAIPEPDDDE
NDMLDLAFGLTETSRLGCQVVMTKELDGIRVALPAMTRNLQQKDFK
>2Fe-2SST1(2740667312)Millerozyma farinosa CBS 7064
MIRSLVRITRGPSVRANIKFVPTAQRICIPFSRGFHYSAPRNHGHIKQPKPGEELKITFI
TKEGEQHTYEVAEGDNILDIAQAHNLDMEGACGGSCACSTCHIIVDPEYYDAIPEPDDDE
NDMLDLAFGLTETSRLGCQVVMTKELDGIRVALPAMTRNLQQKDFK
>2Fe-2SST1(638241122)Debaryomyces hansenii var. hansenii CBS767
MFRSILRSSRCIPKTLQNNCRSPTVVTPLNLYKQFHQTVPKFHGHLHKPNPGEELHITFI
TKDGEQLSFEVAEGDNVLDIAQAHNLDMEGACGGSCACSTCHVIVDPEFYDEIPEPDDDE
NDMLDLAFGLTETSRLGCQIKMSKEIDGIRVALPAMTRNLQNKDFN
>2Fe-2SST1(2517316129)Eremothecium cymbalariae DBVPG#7215
MIGRLLVRQLRTVQSLKPLWLQGNIYNATARLRKPLIQQRHHGILHEPKKGEELHVTFIL
KDGTQKTFEVCEGDTLLDIAQGHNLDVEGACGGSCACSTCHVIVDPDYYDVLDEPGDDEN
DMLDLAYGLTETSRLGCCIKMSKDVDGLRVALPAMTRNVNSSDFE
>2Fe-2SST1(2753004395)Eremothecium sinecaudum ATCC 58844
MLGRLLPRSSNKIPYTALLRTQKTYPSPLYKRSPLLAKRYHYVLQKPKKGEELNVTFILK
DGSQKTFQVCEGDSVLDIAQSNNLDMEGACGGSCACSTCHVIVDPDYYDLLEEPNDDEND
MLDLAYGLTETSRLGCCIKMNKKIDGIRLALPAMTRNVNLSDFE
>2Fe-2SST1(645683142)Lachancea thermotolerans CBS 6340
MFRIAGLSKSLLRGPLAVGRANIMVPKYAGFAQSALRFHGHVHKPKPGEELHVTFILKDG
SQRQYEVSAGDTLLDIAQANNLEMEGACGGSCACSTCHVIVDPDYYDALEEPDDDENDML
DLAYGLTETSRLGCQIKMSKDIDGIRVALPAMTRNVSNTDFD

>2Fe-2SST1(2740615562)Yarrowia lipolytica CLIB89(W29)
MLRTFSRQVLQVSRAVPRTFRPVAPATRLFQTSAPVFHGHVHKPKPGEEIKVTFITKDGE
QIEVDTCEGDNLLDIAQANNLDMEGACGGSCACSTCHVIVDPEYYDKLEEPDDDENDMLD
LAFGLTETSRLGCQVCMSKDLDGIRIALPAMTRNLQASDFN
>2Fe-2SST1(638246243)Yarrowia lipolytica CLIB122
MLRTFSRQVLQVSRAVPRTFRPVAPATRLFQTSAPVFHGHVHKPKPGEEIKVTFITKDGE
QIEVDTCEGDNLLDIAQANNLDMEGACGGSCACSTCHVIVDPEYYDKLEEPDDDENDMLD
LAFGLTETSRLGCQVCMSKDLDGIRIALPAMTRNLQASDFN
>2Fe-2SST1(645674107)Komagataella pastoris GS115
MFKPVFTKRMALNYLGSAIKYRHNPVLRLPRIPVRFHGHLKKPNPGEELHITFITKDGTQ
KTFEVAEGDSLLDIAQGNHLDMEGACGGSCACSTCHVIIDPEFYDEIPEPDDDENDMLDL
AFGLTETSRLGCQVFMKKNLDGIRVALPAMTRNLQSSDFD
>2Fe-2SST1(2740662368)Komagataella phaffii CBS 7435
MFKPVFTKRMALNYLGSAIKYRHNPVLRLPRIPVRFHGHLKKPNPGEELHITFITKDGTQ
KTFEVAEGDSLLDIAQGNHLDMEGACGGSCACSTCHVIIDPEFYDEIPEPDDDENDMLDL
AFGLTETSRLGCQVFMKKNLDGIRVALPAMTRNLQSSDFD
>2Fe-2SST1(2740695651)Ashbya sp. FD-2008
MLGRLLPISARAVRFARAPPVVRALRAHGHLSTPRKGEELQVTFILKDGSQRTFDVAPGD
TLLDIAQGHNLDMEGACGGSCACSTCHVIVDPDYYDALEEPDDDENDMLDLAYGLTETSR
LGCCIRMSKDINGLRVALPAMTRNVSNSDFN
>2Fe-2SST1(638225492)Eremothecium gossypii ATCC 10895
MLGRLLPISARAVRFARAPPFMRALRAHGHLSTPRKGEELQVTFILKDGSQRTFDVAPGD
TLLDIAQGHNLDMEGACGGSCACSTCHVIVDPDYYDALQEPDDDENDMLDLAYGLTETSR
LGCQIRMSKDINGLRVALPAMTRNVSNSDFS
>2Fe-2SST1(649342403)Thalassiosira pseudonana CCMP 1335
ETVTITYVDPDGEEHPVKAEVGKNLLDIAHENNIELEGACGGELACSTCHLVFDRDVFDT
LPPKSDEEEDMLDLAFELTDTSRLGCQICVTKEFEGIKVRIPDDGY

>2Fe-2SST1(639494181)Danio rerio Tuebingen
MAYRMCVRVLLRSSSLLACHPGSVRSHAEFHHTVPSLCSQSQLNGSSSSKVLVHFVNQSG
VKSSVFVTEGETLLDVVIKKNLDFSGFGACEGTLACSTCHLIFEENVFDKLEPMVDEEID
MLDLAYGITKTSRLGCQVTVERWMDGMTVRVPQDIKDQRGKQEAVSSK
>2Fe-2SST1(2740677148)Encephalitozoon romaleae SJ-2008
MYEASQGTGLYSLFPPLKFLSVIFALDLPMAMSYAPDRASGKIKLFFKAMGQMVPVKAVY
GDTVLETAHKNGVNLEGACEGNLACSTCHVILEESLYKRLGEPSDKEYDLIDQAFGGTST
SRLGCQLKVDKNFENAVLTIPRATKNMAVDGFKPEPH
>2Fe-2SST1(639496254)Danio rerio Tuebingen
MSLPTLEQLQSSSSSKVLVHFVNQSGVKSSVFVTEGETLLHVVIKKSLDFIGFGACEGTL
ACSTCHLIFEENVFDKLEPMVDEEIDMLDLAYGITKTSRLGCQVTVERWMDGMTVRVPQD

IKDQRGKQEAVSSK
>2Fe-2SST1(2740826195)Encephalitozoon hellem ATCC 50504
MGMSGAPDRTPGKIEVFFKAMGQVIPAETVCGDTVLEAAHKNGISLEGACEGNLACSTCH
VILEEPLYRRLGEPSDKEYDLLDQAFGGTSTSRLGCQLKIDKSFEKAIFTIPRATKNMAV
DGFKPKPH
>2Fe-2SST1(2517314586)Encephalitozoon intestinalis ATCC 5050
MDMANAPDKTSGKVGLLFKTMGKMIPVNAVYGDTVLETAHKNGVDLEGACEGNLACSTCH
VILEEPLYRRLGEPSDKEYDLIDQAFGITSTSRLGCQLKIDKSFEKTVLTIPRATKNMAV
DGFKPKPH
>2Fe-2SST1(638215829)Encephalitozoon cuniculi GB-M1
MDMFSAPDRIPEQIRIFFKTMKQVVPAKAVCGSTVLDVAHKNGVDLEGACEGNLACSTCH
VILEEPLYRKLGEPSDKEYDLIDQAFGATGTSRLGCQLRVDKSFENAVFTVPRATKNMAV
DGFKPKPH

>2Fe-2SST1(639337880)Homo sapiens
MAAAGGARLLRAASAVLGGPAGRWLHHAGSRAGSSGLLRNRGPGGSAEASRSLSVSARAR
SSSEDKITVHFINRDGETLTTKGKVGDSLLDVVVENNLDIDGFGACEGTLACSTCHLIFE
DHIYEKLDAITDEENDMLDLAYGLTDRSRLGCQICLTKSMDNMTVRVPETVADARQSIDV
GKTS
>2Fe-2SST1(639497030)Danio rerio Tuebingen
MSACVLRAFLQRAAVMRQAAQQTISQTRAFPQSYTHIRGINTYANSLRAEEKVTVHFLNR
DGKRITVKASIGESLLDVVVDRDLDIDGFGACEGTLACSTCHLIFEEDVYKKLGPVSDEE
MDMLDLAYGLTDTSRLGCQVCLRKDLDGMILRVPDVISDARADSEKESSTAPPKI
>2Fe-2SST1(2740816765)Babesia microti RI
MQYYILSQTRAYITRYFMRQNIIYNNFLPLPQSNRAPYLQQCVYYSTNKLIKVTFIFQNG
NEKVVSVESGTSILEAAHKNDIELEGACDGCLACSTCHVILEQKVFDRLPEPSEAEFDML
DLAPCLTDTSRLGCQVKLDEGMDGIKIKLPQITRNFYVDGHIPQAH
>2Fe-2SST1(2740822904)Plasmodium knowlesi H
MTEDGENDYNFFSRNNTFKRALLNKIKNKCIYFNYIRAFTTQSQEEIDVTFVNQDNYEKT
VKAKVGDSILKVAHENSINIEGACDGFCACSTCHVIIDEKYYNLLPEALDNEIDMLELAP
CITETSRLGCQVKLRKDLDGMKIKLPPMTRNFYVDGYVPTPH
>2Fe-2SST1(2740848162)Plasmodium berghei K173
MNSKFSFPIRNNIFKRFLPSKSKNNYLYLNLKKKFTTQNSDEINVTFLNHDNHETTVKAQ
VGDSILKVAHENNINIEGACEGFCACSTCHVIIDNQFYELLPEAQDNELDMLELAPCITE
TSRLGCQVKLTKELDGMKIKLPPMTRNFYVDGYVPTPH
>2Fe-2SST1(2740784779)Plasmodium yoelii 17X
MISRFSFPIRNNIFKRVLPSTFKNNYIYLNFKKSFTTQNSDEINVTFLNHDNHETTVKAQ
VGDSILKVAHENNINIEGACEGFCACSTCHVIIDDEFYELLPEAQDNELDMLELAPCITE
TSRLGCQVKLTKELDGIKIKLPPMTRNFYVDGYVPTPH

```
>2Fe-2SST1(2740576725)Plasmodium coatneyi Hackeri
MITKFSFLSRNDTFKRALLNKIKNKCIYFNYIRAFTTESPEEIDVTFVNQDNYEKTVKAK
VGDSILKVAHENSINIEGACDGFCACSTCHVIIDEKYYDLLPEALDNELDMLELAPCITE
TSRLGCQVKLKKELDGMKIKLPPMTRNFYVDGYVPTPH
>2Fe-2SST1(2740790168)Plasmodium berghei ANKA
MNSKFSFPIRNNIFKRFLPSKSKNNYLYLNLKKKFTTQNSDEINVTFLNHDNHETTVKAQ
VGDSILKVAHENNINIEGACEGFCACSTCHVIIDNQFYELLPEAQDNELDMLELAPCITE
TSRLGCQVKLTKELDGMKIKLPPMTRNFYVDGYVPTPH
>2Fe-2SST1(2740780372)Plasmodium chabaudi AS
MISRFSFPIRNNIFKRVLPSKSKNNYIYLNFIKNFTTQNSDEINVTFLNQDNHETSVKAK
VGDSILKVAHENNINIEGACEGFCACSTCHVIIDKEFYELLPEAQDNELDMLELAPCITE
TSRLGCQVKLTKELDGIKVQLPPMTRNFYVDGHVPTPH
>2Fe-2SST1(2740829509)Theileria orientalis Shintoku
MYRLYNIINSSILLIPKPNLNTLIKAGNRNFTTNNLINISFIQYDEEINVSVPVGISILE
AAHRNNIEIEGACDGCMACSTCHVILDENVYNALPEPTEAEMDMLDLAPCLTPTSRLGCQ
VILNEKHDGIRIKLPRITRNFYVDGYTPSHH
>2Fe-2SST1(2739112146)Theileria annulata Ankara C9
MKLLCNIFNNSINKFLRPNSYFFYKNFYNHFSTQNIKITFVQYEDEITVDVPVGISILEA
AHKHNIEIEGACDGCMACSTCHVILEEDVYDALPEPSESELDMLDLAPCLTNTSRLGCQV
ILGKEHDNIRITLPRITRNFYVDGHTPTPH
>2Fe-2SST1(638271548)Plasmodium falciparum 3D7
MIIYLYFSFFSYPFSFSFYFFWYSDVTFINQDNYEKTVKAKIGDSILKVAHDNHINIEGA
CEGFCACSTCHVIIDENFHDLLPEPLDNEIDMLELAPCITETSRLGCQIKLSKELDGMKI
QLPPMTRNFYVDGHVPTPH

>2Fe-2SST1(2739131228)Leishmania major Friedlin V1
MGNGRMTRRLTGCTSLLCLASVTVQGGSLLQSRRFHGHGGDRNKPVEVEFTLPDGEKKVVMGYEGQTLLDVCAEQGLPMEGACGGSCACSTCHVYLEEKVMDLFQEPTDDENDMID
QAFYPEPTSRLGCQLTLKRGVHDGLKVKMPRATRNMYVDGAKVVPH
```

Subtype 2

Bacteria

>2Fe-2SST2(1B9R)Pseudomonas sp.
PRVVFIDEQSGEYAVDAQDGQSLMEVATQNGVPGIVAECGGSCVCATCRIEIEDAWVEIVGEANPDENDLLQSTGEPMTAGTRLSCQVFIDPSMDGLIVRVPLPA
>2Fe-2SST2(1UWM)Rhodobacter Capsulatus
AKIIFIEHNGTRHEVEAKPGLTVMEAARDNGVPGIDADCGGACACSTCHAYVDPAWVDKLPKALPTETDMIDFAYEPNPATSRLTCQIKVTSLLDGLVVHLPEKQI
>2Fe-2SST2(1PDX)Pseudomonas putida
SKVVYVSHDGTRRELDVADGVSLMQAAVSNGIYDIVGDCGGSASCATCHVYVNEAFTDKVPAANEREIGMLECVTAELKPNSRLCCQIIMTPELDGIVVDVPDRQW
>2Fe-2SST2(CCH23747.1)Corynebacterium glutamicum K051
MSTIHFIDHAGKTRTIEATVGDSVMETAVRNGVPGIVAECGGSLSCATCHVFVDPAQYDALPPMEEMEDEMLWGAAVDREDCSRLSCQIKVTEGMDLSLTTPETQV

Eukaryota

>2Fe-2SST2(ath:AT4G05450)Arabidopsis thaliana
MIGHRISRLGSTIVKQLAREGYLATYGTKNLHRSYGHYLQSLPVVPRQARTSQEAWFLKSHKFCTSSTTSSENGDEETEKITIIFVDKDGEEIPVKVPIGMSVLEAAHENDIDLEG
ACEASLACSTCHVIVMDTEYYNKLEEPTDEENDMLDLAFGLTETSRLGCQVIARPELDGVRLAIPSATRNFAVDGFVPKPH

>2Fe-2SST2(2505189252)Paulinella chromatophora
MPVIRFVREGIDVECYPGENLREVALRHGIELYGIKGKLGNCGGCGQCITCFVDIISPHG
SNSPSTLSARTQVENMKLRRRPDTWRLGCQVLVYGSVLVLTRPQLGLPDAEYRIAAARTA
KLSKGNMSWFSPEVNINPEQSTTSSEGDEEPALNSDTVS
>2Fe-2SST2(2739133352)Leishmania major Friedlin V1
MHRLALPRVVLCARHPAASAPLTTSSGASLRWCSSPPPSLSPSTTPGKVKVHIKSETEGT
EKTVEAALGLTLMEVIRDVAKMDMEAACDGTCACSTCHVIFTAASYQKLLDAPSEDEMDMLDLAPKVTKTSRLSCQVKIAPELDDISVTIPNEMENQMGY

>2Fe-2SST2(640421166)Ostreococcus lucimarinus CCE9901
IEVTFVERDGERRAVRGLIGENLLETAHRHDVELEGACEGSLACSTCHVVFEDEKVFETLPEACDDENDMLDLAYGLTATSRLGCQVTLERGALEGCVVILPRATRNFAVDGFVPK
PH
>2Fe-2SST2(640421163)Ostreococcus lucimarinus CCE9901
IEVTFVERDGERRAVRGLIGENLLETAHRHDVELEGACEGSLACSTCHVVFEDEKVFETLPEACDDENDMLDLAYGLTATSRLGCQVTLERGALEGCVVILPRATRNFAVDGFVPK
PH

>2Fe-2SST2(639625305)Trypanosoma brucei brucei 927/4 GUTat10.1
MAALRYLLAPRRALVTPFLRSVTCVSSGGIFSSQRPHTFPVLWHAVRTHSNDSGERSKPLTLHVQLPCGTMRTLTAYEGQTLLDVAMEHGLPIEGACGGSCACSTCHVYLENDEAM
ELFDEATDEENDMLDMAFFPQSTSRLGCQLTLRQQKHDGLKISLPKATRNMYVDGHTITPHH

>2Fe-2SST2(2517260843)Thielavia terrestris NRRL 8126
MSTPKLLAQTLPSLRLATRRTAPSIARHVVRCYTAASAAPRPAPPAWSSQPPIRRSLWSA
AFQSRPFSVSTAVSHGHIQPPKPGEELYVTFIDKEGVEHKLAVSKGDNLLDIAQAHDLEM
EGACGGSCACSTCHVIVMDQEYYDRMPEPDDDENDMLDLAFGLQETSRLGCQVVMTPELN
GLRVKLPAMTRNLQSSDFK
>2Fe-2SST2(2762808093)Thielavia terrestris NRRL 8126
MSTPKLLAQTLPSLRLATRRTAPSIARHVVRCYTAASAAPRPAPPAWSSQPPIRRSLWSA
AFQSRPFSVSTAVSHGHIQPPKPGEELYVTFIDKEGVEHKLAVSKGDNLLDIAQAHDLEM
EGACGGSCACSTCHVIVMDQEYYDRMPEPDDDENDMLDLAFGLQETSRLGCQVVMTPELN
GLRVKLPAMTRNLQSSDFK
>2Fe-2SST2(2762871174)Thermothelomyces thermophila ATCC 42464
MSAPKLFAQALPTLRRAPRLTTGAIGRRVRCYTQASAAARPTNSSRVVQAVARRSPALMR
AQRRTFSASPAVSHGHIKPPKPGEELYVTFVDKEGVEHKFAVSKGDNLLDIAQANDLEME
GACGGSCACSTCHVIVLDQEYYDKMPEPDDDENDMLDLAFGLQETSRLGCQVVMTPELDG
LRVKLPSMTRNLQSSDFK
>2Fe-2SST2(2517254265)Thermothelomyces thermophila ATCC 42464
MSAPKLFAQALPTLRRAPRLTTGAIGRRVRCYTQASAAARPTNSSRVVQAVARRSPALMR
AQRRTFSASPAVSHGHIKPPKPGEELYVTFVDKEGVEHKFAVSKGDNLLDIAQANDLEME
GACGGSCACSTCHVIVLDQEYYDKMPEPDDDENDMLDLAFGLQETSRLGCQVVMTPELDG
LRVKLPSMTRNLQSSDFK
>2Fe-2SST2(640296514)Fusarium graminearum PH-1
MSASRSFATCLAKLPATACRTTQPMRFSQNIMSQASRSQFAVIPRAVRFSPVSQRAFTTS
TQRRHGHIDPPKPGEELYVTFIEKDGTENKFAVSEGDNLLDIAQANDLEMEGACGGSCAC
STCHVIVADDEYFDKMPEPEDDENDMLDLAFGLTETSRLGCQVKMTKEMDGLVVKLPSMT
RNLQASDFS
>2Fe-2SST2(2740844848)Fusarium fujikuroi IMI 58289
MSASRSFATCLAKLPATAPRTIQPMRLGQNVVSQTSKGRSPTISRFTRFSPVSHRAFTTT
TQRRHGHIDPPKPGEELYVTFIEKDGTENKFAVSEGDNLLDIAQANDLEMEGACGGSCAC
STCHVIVADDAYFDKMPEPEDDENDMLDLAFGLTETSRLGCQVKMTKELDGLVVKLPSMT
RNLQASDFS
>2Fe-2SST2(639576354)Arabidopsis thaliana Columbia
MVFHRLSRLGSRIVKELPRERHLSMCGKRILQRSYGQYLQSSPMLQRQTRSFKEALFSNN
HKFCTSFSTTSEKGGEKTEKINVTFVDKDGEEIHIKVPVGMNILEAAHENDIELEGACEG
SLACSTCHVIVMDTKYYNKLEEPTDEENDMLDLAFGLTATSRLGCQVIAKPELDGVRLAI
PSATRNFAVDGFVPKPH
>2Fe-2SST2(639574862)Arabidopsis thaliana Columbia
MIGHRISRLGSTIVKQLAREGYLATYGTKNLHRSYGHYLQSLPVVPRQARTSQEAWFLKS

HKFCTSSTTSSENGDEETEKITIIFVDKDGEEIPVKVPIGMSVLEAAHENDIDLEGACEA
SLACSTCHVIVMDTEYYNKLEEPTDEENDMLDLAFGLTETSRLGCQVIARPELDGVRLAI
PSATRNFAVDGFVPKPH
>2Fe-2SST2(2507880113)Zea mays mays cv. B73
MFPRISRLGARLLRESRAETRAGNLLNSQRIFFQDHVNRHSIPVVTPAVLSMRNALLSTA
TSGDQDESSQAKDKISVTFVNKDGSEKTICVPVGMSMLEAAHENDIELEGACEGSLACST
CHVIVMDVKYYNKLEDPADEENDMLDLAFGLTETSRLGCQVIAKPELDGMRLALPVATRN
FAVDGYVPKPH
>2Fe-2SST2(2507880112)Zea mays mays cv. B73
MFPRISRLGARLLRESRAETRAGNLLNSQRIFFQDHVNRHSIPVVTPAVLSMRNALLSTA
TSGDQDESSQAKDKISVTFVNKDGSEKTICVPVGMSMLEAAHENDIELEGACEGSLACST
CHVIVMDVKYYNKLEDPADEENDMLDLAFGLTETSRLGCQVIAKPELDGMRLALPVATRN
FAVDGYVPKPH
>2Fe-2SST2(2507880116)Zea mays mays cv. B73
MIVEVIFLHTVNRISVTFVNKDGSEKTICVPVGMSMLEAAHENDIELEGACEGSLACSTC
HVIVMDVKYYNKLEDPADEENDMLDLAFGLTETSRLGCQVIAKPELDGMRLALPVATRNF
AVDGYVPKPH
>2Fe-2SST2(2507880115)Zea mays mays cv. B73
MIVEVIFLHTVNRISVTFVNKDGSEKTICVPVGMSMLEAAHENDIELEGACEGSLACSTC
HVIVMDVKYYNKLEDPADEENDMLDLAFGLTETSRLGCQVIAKPELDGMRLALPVATRNF
AVDGYVPKPH
>2Fe-2SST2(2507880114)Zea mays mays cv. B73
MIVEVIFLHTVNRISVTFVNKDGSEKTICVPVGMSMLEAAHENDIELEGACEGSLACSTC
HVIVMDVKYYNKLEDPADEENDMLDLAFGLTETSRLGCQVIAKPELDGMRLALPVATRNF
AVDGYVPKPH
>2Fe-2SST2(2507857107)Zea mays mays cv. B73
MTDVVIFLCIMHRISVTFVNKDGSEKTIRVPVGMSMLEAAHENDIELEGACEGSLACSTC
HVIVMDVNYYNKLEDPADEENDMLDLAFGLTETSRLGCQVIAKPELDGIRLALPVATRNF
AVDGYVPKPH
>2Fe-2SST2(2508320447)Micromonas pusilla NOUM17, RCC 299
MLEVAHKNDIELEGACEGSLACSTCHVIINDQAVYDALPEPDDDENDMLDLAFGLTETSR
LGCQVIAAKELDGMTLSLPKATRNFAVDGFVPKPH

>2Fe-2SST2(2507880117)Zea mays mays cv. B73
MITFPAIVATAGACEGSLACSTCHVIVMDVKYYNKLEDPADEENDMLDLAFGLTETSRLGCQVIAKPELDGMRLALPVATRNFAVDGYVPKPH

Subtype 3

Archaea

>2Fe-2SST3(2545641477)Halobiforma lacisalsi AJ5, JCM 12983
VTGSTSTTWTVELRVPEDADLEAAGESRSIEVRENQSILSAARGAGLWLSADCQQGWCIT
CGARLLEGEVDHANAKRYYPEDEAAGFVLTCVAQPRSDCVLEVERYDELLEHRAEHDRPP
GRSKLG
>2Fe-2SST3(2509883874)Natrinema pellirubrum 157
MTASTTWEVELRVPDDADLEAAGESRTIAVREDEAILAAARRAGLWLSADCQQGWCITCG
ARLLEGEVDHSDAKRYYPEDEAENFVLTCVAQPRADCVIVVEQYDELLRHRADHDRPPGR
SKLG
>2Fe-2SST3(2517201353)Natrinema sp. J7-2
MTAPTTWEVELRVPEDAALDAAGESRTVAVREDQSILTAARAADLWLSADCQQGWCITCG
AKLRSGEVDHSRAKRYYPSDEAANFVLTCVARPRSDCVIEVERSEALLRHRADHDKPPGR
SKLE
>2Fe-2SST3(2510572427)Natronobacterium gregoryi SP2(2510572427)
MTAATTWTVELRVPQDADLEAAGESRTIEVPEDQSILSAARAAGIWLTADCQQGWCITCG
ARLLEGEVDHSTAKRYYPEDERAGFVLTCVARPRSDCVLEVERDDELLRHRADHDRPPGR
SKLD

>2Fe-2SST3(648063341)Halalkalicoccus jeotgali B3, DSM 18796
MGETTYSVEIVVPEDADSQRAGETVETRVDEDDYVLASARSQDVWLAADCQQGWCTTCAA
ELLEGEVDQSDAKRYYESDEAAGMILPCTAKPRSDLTIRAFQYEEMLEHRAANDNPPGNS
KLD
>2Fe-2SST3(2507055677)Halobacterium sp. DL1
MELVVPEDCEIPQAGETVDVEVPEGEYVLWAAREAGVWLPADCQQGWCCRCAGELLSGEL
DQTDARRYFETDRDAALHLLCTAVPQSDCRIRVCQYEEMLDERAKQELPPGNSKR
>2Fe-2SST3(2540571112)Natronomonas moolapensis 8.8.11, DSM 18674
MSDADTDVWDVTIEIPADADLEEAGETHTIRVGSEEYILAAAREQGLWVPADCQQGWCIT
CAATVLEGDVDQSDAKRYYSEDADADFALICTAKPNSDVTLKVGAHEELLRHRADTDRPP
GSSKL

Bacteria

```
>2Fe-2SST3(1CZP)Nostoc sp.
ATFKVTLINEAEGTKHEIEVPDDEYILDAAEEQGYDLPFSCRAGACSTCAGKLVSGTVDQSDQSFLDDDQIEAGYVLTCVAYPTSDVVIQTHKEEDLY
>2Fe-2SST3(1RFK)Mastigocladus laminosus
ATYKVTLINEAEGLNKTIEVPDDQYILDAAEEAGIDLPYSCRAGACSTCAGKLISGTVDQSDQSFLDDDQIEAGYVLTCVAYPTSDCVIETHKEEELY
>2Fe-2SST3(ABB57529.1)Synechococcus elongatus PCC 7942 = FACHB-805
MATYKVTLVNAAEGLNTTIDVADDTYILDAAEEQGIDLPYSCRAGACSTCAGKVVSGTVDQSDQSFLDDDQIAAGFVLTCVAYPTSDVTIETHKEEDLY
>2Fe-2SST3(4FXC)Arthrospira platensis
ATYKVTLINEAEGINETIDCDDDTYILDAAEEAGLDLPYSCRAGACSTCAGTITSGTIDQSDQSFLDDDQIEAGYVLTCVAYPTSDCTIKTHQEEGLY

>2Fe-2SST3(3B2G)Leptolyngbya boryana
PSFKVTLINETEGLNTTIEVPDDEYILDAAEEQGIDLPYSCRAGACSTCAGKITAGTVDQSDQSFLDDDQIQAGYVLTCVAYPTSDCTILTHQEEDLY
>2Fe-2SST3(1OFF)SYNECHOCYSTIS SP.
MASYTVKLITPDGESSIECSDDTYILDAAEEAGLDLPYSCRAGACSTCAGKITAGSVDQSDQSFLDDDQIEAGYVLTCVAYPTSDCTIETHKEEDLY

>2Fe-2SST3(1ROE)Synechococcus elongatus
ATYKVTLVRPDGSETTIDVPEDEYILDVAEEQGLDLPFSCRAGACSTCAGKLLEGEVDQSDQSFLDDDQIEKGFVLTCVAYPRSDCKILTNQEEELY
>2Fe-2SST3(ABB58611.1)Synechococcus elongatus PCC 7942 = FACHB-805
MSDTYTVRIRDRRTDEEFTVQVPPDRYILQTAEEQGYELPFSCRNGACTACAVRVLGGAIEQTEAMGLSAPLRQRGYALLCVSYPRSDVIVETQDEDEVYMLQFGRYFGQGKVSFG
LPLDEE
>2Fe-2SST3(ABB56370.1)Synechococcus elongatus PCC 7942 = FACHB-805
MATYQVEVIYQGQSQTFTADSDQSVLDSAQAAGVDLPASCLTGVCTTCAARILSGEVDQPDAMGVGPEPAKQGYTLLCVAYPRSDLKIETHKEDELYALQFGQPG

>2Fe-2SST3(1IUE)Plasmodium falciparum
AFYNITLRTNDGEKKIECNEDEYILDASERQNVELPYSCRGGSCSTCAAKLVEGEVDNDDQSYLDEEQIKKKYILLCTCYPKSDCVIETHKEDELHDM

>2Fe-2SST3(1FRD)Nostoc sp.
ASYQVRLINKKQDIDTTIEIDEETTILDGAEENGIELPFSCHSGSCSSCVGKVVEGEVDQSDQIFLDDEQMGKGFALLCVTYPRSNCTIKTHQEPYLA
```

Eukaryota

```
>2Fe-2SST3(2MH7)Chlamydomonas reinhardtii
MYKVTLKTPSGDKTIECPADTYILDAAEEAGLDLPYSCRAGACSSCAGKVAAGTVDQSDQSFLDDAQMGNGFVLTCVAYPTSDCTIQTHQEEALYENLYFQ

>2Fe-2SST3(1AWD)Chlorella fusca
```

```
YKVTLKTPSGEETIECPEDTYILDAAEEAGLDLPYSCRAGACSSCAGKVESGEVDQSDQSFLDDAQMGKGFVLTCVAYPTSDVTILTHQEAALY
>2Fe-2SST3(3AB5)Cyanidioschyzon merolae
MYKIQLVNQKEGIDVTIQCAGDQYILDAAEEQGVDLPYSCRAGACSTCAGKLVKGSVDQSDQSFLDEDQISKGFILTCVAYPTSDCVIQTHQEEALY

>2Fe-2SST3(1FRR)Equisetum arvense
AYKTVLKTPSGEFTLDVPEGTTILDAAEEAGYDLPFSCRAGACSSCLGKVVSGSVDESEGSFLDDGQMEEGFVLTCIAIPESDLVIETHKEEELF
>2Fe-2SST3(5H57)Zea mays
AVYKVKLVGPEGEEHEFDAPDDAYILDAAETAGVELPYSCRAGACSTCAGKIESGSVDQSDGSFLDDGQQEEGYVLTCVSYPKSDCVIHTHKEGDLY
>2Fe-2SST3(4ITK)Chlamydomonas reinhardtii
MFKVTFKTPKGEKTIDVEADKYLLDAAEEAGMDLPYSCRSGGCSTCCGKLESGTVDQSDQNMLDEDQLKQGFVLTCVAYPTSDIVILTDQESKLPIGGSENLYFQ
>2Fe-2SST3(1WRI)Equisetum arvense
AYKVTLKTPDGDITFDVEPGERLIDIGSEKADLPLSCQAGACSTCLGKIVSGTVDQSEGSFLDDEQIEQGYVLTCIAIPESDVVIETHKEDEL
>2Fe-2SST3(3B2F)Zea mays
ATYNVKLITPEGEVELQVPDDVYILDQAEEDGIDLPYSCRAGSCSSCAGKVVSGSVDQSDQSYLDDGQIADGWVLTCHAYPTSDVVIETHKEEELTGA
>2Fe-2SST3(4ZHO)Arabidopsis thaliana
MATYKVKFITPEGELEVECDDDVYVLDAAEEAGIDLPYSCRAGSCSSCAGKVVSGSVDQSDQSFLDDEQIGEGFVLTCAAYPTSDVTIETHKEEAIMLEHHHHHH
>2Fe-2SST3(ath:AT1G10960)Arabidopsis thaliana
MASTALSSAIVSTSFLRRQQTPISLRSLPFANTQSLFGLKSSTARGGRVTAMATYKVKFITPEGEQEVECEEDVYVLDAAEEAGLDLPYSCRAGSCSSCAGKVVSGSIDQSDQSFL
DDEQMSEGYVLTCVAYPTSDVVIETHKEEAIM
>2Fe-2SST3(ath:AT1G32550)Arabidopsis thaliana
MALILPCTFCTSLQKKNFPINRRYITNFRRGATTATCEFRIPVEVSTPSDRGSLVVPSHKVTVHDRQRGVVHEFEVPEDQYILHSAESQNISLPFACRHGCCTSCAVRVKSGELRQ
PQALGISAELKSQGYALLCVGFPTSDLEVETQDEDEVYWLQFGRYFARGPIERDDYALELAMGDE
>2Fe-2SST3(ath:AT2G27510)Arabidopsis thaliana
MATVRISSTSMTKAVLRSQTTNKLITNKSYNLSVGSTKRVSRSFGLKCSANSGGATMSAVYKVKLLGPDGQEDEFEVQDDQYILDAAEEAGVDLPYSCRAGACSTCAGQIVSGNVD
QSDGSFLEDSHLEKGYVLTCVAYPQSDCVIHTHKETELF
>2Fe-2SST3(ath:AT5G10000)Arabidopsis thaliana
MDQVLYSSYIIKIPVISRISPSQAQLTTRLNNTTYFGLSSSRGNFGKVFAKESRKVKLISPEGEEQEIEGNEDCCILESAENAGLELPYSCRSGTCGTCCGKLVSGKVDQSLGSFL
EEEQIQKGYILTCIALPLEDCVVYTHKQSDLI
>2Fe-2SST3(1PFD)Petroselinum crispum
ATYNVKLITPDGEVEFKCDDDVYVLDQAEEEGIDIPYSCRAGSCSSCAGKVVSGSIDQSDQSFLDDEQMDAGYVLTCHAYPTSDVVIETHKEEEIV
>2Fe-2SST3(1A70)Spinacia oleracea
AAYKVTLVTPTGNVEFQCPDDVYILDAAEEEGIDLPYSCRAGSCSSCAGKLKTGSLNQDDQSFLDDDQIDEGWVLTCAAYPVSDVTIETHKKEELTA

>2Fe-2SST3(2508317128)Micromonas pusilla NOUM17, RCC 299
MSATVTMSAVAAKTGARLSSRAISKQARALAATPRVAAKPRASLTAKAMLVTIEHEGKTY
EVECDGHDNILDAALDAGIENLSYDCKMGVCMTCPSRVTAGKVDQQGSMLSDDVEEKGFA
LLCCAKPLGEGVVIKTVTEEELLDEQLCA
```

>2Fe-2SST3(640417898)Ostreococcus lucimarinus CCE9901
AVAVEIRHEGKTYNLEVADGDNILDVALDAGIDLRYDCKMGVCMMCPAKVVAGAIDQSGS
MLSDDVEEKGYALLCCAVPQGEDVVIQTVSEDELLEEQLCSSD

>2Fe-2SST3(640417588)Ostreococcus lucimarinus CCE9901
SVKVTDHETGEMLELDVPEGRYILFEAEQQGWVLPNACRMGGCTKCAVKISKGSLEQPES
LGLSKELKDQGYALLCVATATEDVECVTQDEEEVYMKQFGKSFAE

>2Fe-2SST3(2505188780)Paulinella chromatophora
MRSSHAIIVHWSQQAYTFSHKVPEGEYILNSFEQHGALLPFNCRNGCCTTCAVQVLNGDI
DHREAFGLSSQIRNRGYGLLCVACSMNPLELETQEEDEVYNSQFGIFFGRGKVIKGLPLD
EG
>2Fe-2SST3(2505188822)Paulinella chromatophora
MAQIFSISIDFNGTTYSFPCSSDQTVLMGAETAGIELPSSCCSGLCTTCASKLHEGKVYQ
PDAMGIKADLSNEGYALLCVAYPLSDLKLEANQEDALYEAQFGQYQK
>2Fe-2SST3(2507842412)Zea mays mays cv. B73
MTCPAATATATTARIGARRGPCLWRSERRASFLVRSTSLLPDRPARDTRARAASELQQAP
RPASATVPAHKVTVHDRQRGVIHEFVVPEDQYILHTAEAQDIRLPFACRHGCCTSCAVRI
KSGQIRQPEALGISAELKDKGYALLCVGFPSGDVEVETQDEDEVYWLQFGRYFARGPVER
DDYALELAMGDE
>2Fe-2SST3(639587729)Arabidopsis thaliana Columbia
MALILPCTFCTSLQKKNFPINRRYITNFRRGATTATCEFRIPVEVSTPSDRGSLVVPSHK
VTVHDRQRGVVHEFEVPEDQYILHSAESQNISLPFACRHGCCTSCAVRVKSGELRQPQAL
GISAELKSQGYALLCVGFPTSDLEVETQDEDEVYWLQFGRYFARGPIERDDYALELAMGD
E
>2Fe-2SST3(2740822756)Plasmodium knowlesi H
MTMIMLFLMLILLTEKSSTYRFSNNRTALKYMSGSRGINICAPQRRACSSFINNLVSSKK
ASCLPVGGDRGVNARNTSSNLSNDGGKRRYFKSVNRNKLFYNITLRTNDGEKKIQCDEDE
YILDASERQNVELPYSCRGGSCSTCAAKLIEGEVDNEDQSYLDEEQLKKKYILLCTCYPK
SDCVIETHKEEELHDM
>2Fe-2SST3(2740578133)Plasmodium coatneyi Hackeri
MTAIIMFLMLLLLTEKSNTYKISNKRTTLNYMYGSRGINICAPQKKACSSFISNLVNSKK
ASCLPVGGDRGVNARHTSSNLSNDGGKRRYFKSANRNKLFYNITLRTNDGEKKIQCDEDE
YILDACERQNVELPYSCRGGSCSTCAAKLIEGKVDNEDQSYLDEEQLKKKYVLLCTCYPK
SDCVIETHKEEELHDM

>2Fe-2SST3(638274858)Plasmodium falciparum 3D7
MNIVILLLILTFSIKHSNTYKLKNTYIPINYMYHNNKNILRSQKSKLFLNFLSNNQLANS
NKQTCFFKSNIKSSISNIDNYDYIRKRYINTSNKNKLFYNITLRTNDGEKKIECNEDEYI
LDASERQNVELPYSCRGGSCSTCAAKLVEGEVDNDDQSYLDEEQIKKKYILLCTCYPKSD
CVIETHKEDELHDM
>2Fe-2SST3(2740848019)Plasmodium berghei K173
MRVALFFLYYIFFFKQNNTYKLNNNPPPLNYMYGNKLIINIPRNRVCVRLINNLPNGSKH
SVFLMNSNKFNLKIGCDHNINKRRYSVSPNPGKLFYNITLRTNDGEKKIECEEDEYILDA
SERQNVELPYSCRGGSCSTCAAKLIEGEVDNEEQSYLDDEQLKKKYILLCTCYPKSDCVI
ETHKEDELHDM
>2Fe-2SST3(2740784636)Plasmodium yoelii 17X
MRVVLFFLYYIFFFKQNNTYKLNNNPPPLNYMYGNKLIINIPRNRICARLINNLANGSKH
SVFLMNSNKFNLEIGCNHNINKRRYSGSPNPGKLFYNITLRTNDGEKKIECEEDEYILDA
SERQNVELPYSCRGGSCSTCAAKLIEGEVDNEEQSYLDDEQLKQKYILLCTCYPKSDCVI
ETHKEDELHDM
>2Fe-2SST3(2740790025)Plasmodium berghei ANKA
MRVALFFLYYIFFFKQNNTYKLNNNPPPLNYMYGNKLIINIPRNRVCVRLINNLPNGSKH
SVFLMNSNKFNLKIGCDHNINKRRYSVSPNPGKLFYNITLRTNDGEKKIECEEDEYILDA
SERQNVELPYSCRGGSCSTCAAKLIEGEVDNEEQSYLDDEQLKKKYILLCTCYPKSDCVI
ETHKEDELHDM
>2Fe-2SST3(2740780229)Plasmodium chabaudi AS
MRVLLFFLYYIFFFKQHNTYKLSNKSPPLNYMYGNKLIINIPRNRICARLINNLDNGSKH
SVFLMNNNQFNLKIGCNHNINKRRYSGSPSSDKLFYNITLRTNDGEKKIECAEDEYILDA
CEKQNVELPYSCRGGSCSTCAAKLIEGEVDNEEQSYLDDEQLKQKYILLCTCYPKSDCVI
ETHKEEELHDM
>2Fe-2SST3(2740829329)Theileria orientalis Shintoku
MANLQGFKFLYACLGCILAFIRGVSGISSHRWTHSAFLIAPTNFHFKSHSNLCKRQQNNQ
LDDTDLFKELNSRDRHGRLRLRHAIRLILPEGEKVIESSEDEYILEAAENQGIELPYSCR
GGSCSTCAAALVIGEIDNCEQSYLSDEQIKQGYCLLCTSYAKSDCTIKTHKEEELHREEE
EQGKEKQE
>2Fe-2SST3(2739114173)Theileria annulata Ankara C9
MFLFFIHVCYLVFCSVVACITNRRFSTSFINSPRNFSYSLSNSNLNTFSQPFNKGIEREL
INSSKFSDRRIPLYYAVKLVLPEGEKVIESAEDEYILESAESQGVELPYSCRGGSCSTCA
ATLVSGEIDNSEQSYLDDDQVKKGYCLLCTSYAKSDCTIETHKEDKLHEEEETSTNNNII
>2Fe-2SST3(2740819274)Babesia microti RI
MTYVVYGGSIYLIFLVILHIINCNVVLSHRRTSYAFITSRSIQLQTNNIKSCHKVTFVTP
KGKKIVYCEEDEYVLDSAEAAGLDLPYSCRGGSCSTCNCMLTDGNMSNEDQSYLTDEELR
KGYRLICTAYPHSDCTVITHKEDELHKLRGESS
>2Fe-2SST3(639579225)Arabidopsis thaliana Columbia

```
MDQVLYSSYIIKIPVISRISPSQAQLTTRLNNTTYFGLSSSRGNFGKVFAKESRKVKLIS
PEGEEQEIEGNEDCCILESAENAGLELPYSCRSGTCGTCCGKLVSGKVDQSLGSFLEEEQ
IQKGYILTCIALPLEDCVVYTHKQSDLI

>2Fe-2SST3(2740742500)Babesia bigemina Bond
MRLLEAFSLLCVVVLNIHLVVNVTGERMLHHCTLPWQQFRVGYVTHCNRFRKYRMWSTSL
VPLANDRRYFRHLRPSGLFYSVKFVTPQGDVTVDCDPDEYILEAAEKAGIDLPYSCRGGS
CSTCAGKIVNGIVDNEEQSYLDNEQMSQGYCLLCTCYPRSDCTIVTHKEEELHASGSST

>2Fe-2SST3(2507852741)Zea mays mays cv. B73
MAATALSMSILRAPPPCFSSPLRLRVAVAKPLAAPMRRQLLRAQATYNVKLITPEGEVEL
QVPDDVYILDFAEEEGIDLPFSCRAGSCSSCAGKVVSGSVDQSDQSFLNDNQVADGWVLT
CAAYPTSDVVIETHKEDDLL
>2Fe-2SST3(640293778)Fusarium graminearum PH-1
MRSIDPVGNVAGVVHRTALPRISIAISDIAIGLLRLLIFHQSLNMSYKVTIKTPNEDYTF
NCGSDEYILDVAESNGIKLPYSCRAGVYSSCAGKLVSGTIQQDDQDFLDSDQVEAGYVLL
CIAYPTSDCIIKANAEDEL

>2Fe-2SST3(2507831497)Zea mays mays cv. B73
MAAAAAALVSSNSLRAPAAFSVVVRAASRPAPVVAVVTPLPAAKAAAARGARLRAQATHN
VKLITPDGEVQLKMPGDVYILDHAEERGLDLPYSCRAGACSSCVGKIVSGSVNQFDQSFL
DDDQVAEGFVLTCIAYPTSDLVIQTHKEDALVESY
>2Fe-2SST3(2507859608)Zea mays mays cv. B73
MSTATAARLPAPRSGASYHYQTAAPAANTLSFAGHARQAARASGPRLSSRFVAAAAAVLH
KVKLVGPDGTEHEFEAPDDTYILEAAETAGVELPFSCRAGSCSTCAGRMSAGEVDQSEGS
FLDDGQMAEGYLLTCISYPKADCVIHTHKEEDLY
>2Fe-2SST3(2507852735)Zea mays mays cv. B73
MATVLGSPRAPAFFFSPSSLRAAPAPTAVALPAAKVGIMGRSASSRGRLRAQATYNVKLI
TPEGEVELQVPDDVYILDQAEEDGIDLPYSCRAGSCSSCAGKVVSGSVDQSDQSYLDDGQ
IAAGWVLTCHAYPTSDVVIETHKEEELTGA
>2Fe-2SST3(2507852738)Zea mays mays cv. B73
MATVLSSPRAPAFSFSLRAAPAPTTVAMTRGGGASSRLRAQATYNVKLITPEGEVELQVP
DDVYILDYAEEEGIDLPYSCRAGSCSSCAGKVVSGSVDQSDQSYLDDGQIAAGWVLTCVA
YPTSDVVIETHKEDDLIS
>2Fe-2SST3(2507852737)Zea mays mays cv. B73
MATVLSSPRAPAFSFSLRAAPAPTTVAMTRGGGASSRLRAQATYNVKLITPEGEVELQVP
DDVYILDYAEEEGIDLPYSCRAGSCSSCAGKVVSGSVDQSDQSYLDDGQIAAGWVLTCVA
YPTSDVVIETHKEDDLIS
>2Fe-2SST3(2507878499)Zea mays mays cv. B73
MATAAAAATAMCSVPGPSGSMRRRAFCTWKKADAPRVASSSVARVVRASAAAVHRVKLVG
```

PDGSESELEVAEDTYVLDAAEEAGLELPYSCRAGSCATCAGKLASGEVDQSEGSFLDDAQ
RAEGYVLTCVSYPRADCVIYTHKEEEVH
>2Fe-2SST3(2507878482)Zea mays mays cv. B73
MATAAAAATAMCSVPGPSGSMRRRAFCTWKKADAPRVASSSVARVVRASAAAVHRVKLVG
PDGSESELEVAEDTYVLDAAEEAGLELPYSCRAGSCATCAGKLASGEVDQSEGSFLDDAQ
RAEGYVLTCVSYPRADCVIYTHKEEEVH
>2Fe-2SST3(2507843362)Zea mays mays cv. B73
MATVLSSPRAPAFSFSLRAAPAPTTVAMTRGGGASSRLRAQATYNVPDDVYILDFAEEEG
IDLPFSCRAGSCSSCAGKVVSGSVDQSDQSFLNDNQVADGWVLTCAAYPTSDVVIETHKE
DDLL
>2Fe-2SST3(639567408)Arabidopsis thaliana Columbia
MATVRISSTSMTKAVLRSQTTNKLITNKSYNLSVGSTKRVSRSFGLKCSANSGGATMSAV
YKVKLLGPDGQEDEFEVQDDQYILDAAEEAGVDLPYSCRAGACSTCAGQIVSGNVDQSDG
SFLEDSHLEKGYVLTCVAYPQSDCVIHTHKETELF
>2Fe-2SST3(2507809369)Zea mays mays cv. B73
MATFASPLLCNFMAIEQKNAPQLTNSRKPSPRLSFPTTRSMPTLPLPGFRARPDWRVAAV
YKVKLIGPEGQESVIQVPEDSYILDAAEEAGIDLPYSCRAGACSTCAGKVLEGSVDQADQ
SFLDDAQVGAGYALTCVAYPTSDCVIQTHREADLY
>2Fe-2SST3(2507892092)Zea mays mays cv. B73
MAMATFTSSPVLCSFRTIEQKKAPLLNNSTKRPLHLGFPRSVPTSPSLRERLDLRVAAVY
KVKLLGPEGQESVLDVPEDSYILDAAEEAGLDLPYSCRAGACSTCAGKLLEGSVDQADQS
FLDEAQVGAGYALTCVAYPTSDCVIQTHREEDLY
>2Fe-2SST3(2507804759)Zea mays mays cv. B73
MSTSTFATSCTLLGNVRTTQASQTAVKSPSSLSFFSQVTKVPSLKTSKKLDVSAMAVYKV
KLVGPEGEEHEFDAPDDAYILDAAETAGVELPYSCRAGACSTCAGKIESGSVDQSDGSFL
DDGQQEEGYVLTCVSYPKSDCVIHTHKEGDLY
>2Fe-2SST3(2507804758)Zea mays mays cv. B73
MSTSTFATSCTLLGNVRTTQASQTAVKSPSSLSFFSQVTKVPSLKTSKKLDVSAMAVYKV
KLVGPEGEEHEFDAPDDAYILDAAETAGVELPYSCRAGACSTCAGKIESGSVDQSDGSFL
DDGQQEEGYVLTCVSYPKSDCVIHTHKEGDLY
>2Fe-2SST3(639589425)Arabidopsis thaliana Columbia
MASTALSSAIVGTSFIRRSPAPISLRSLPSANTQSLFGLKSGTARGGRVTAMATYKVKFI
TPEGELEVECDDDVYVLDAAEEAGIDLPYSCRAGSCSSCAGKVVSGSVDQSDQSFLDDEQ
IGEGFVLTCAAYPTSDVTIETHKEEDIV
>2Fe-2SST3(639585313)Arabidopsis thaliana Columbia
MASTALSSAIVSTSFLRRQQTPISLRSLPFANTQSLFGLKSSTARGGRVTAMATYKVKFI
TPEGEQEVECEEDVYVLDAAEEAGLDLPYSCRAGSCSSCAGKVVSGSIDQSDQSFLDDEQ
MSEGYVLTCVAYPTSDVVIETHKEEAIM
>2Fe-2SST3(2505189316)Paulinella chromatophora

MASYKVTLINESDGLNKTIEVPDDQYILDAAEEQGIDLPYSCRAGACSTCAGKITAGTVD
QSDQSFLDDDQIEAGFVLTCVAYPTSDCTIKTHAEEELY
>2Fe-2SST3(649363878)Thalassiosira pseudonana CCMP 1335
MATYKVTLISEEHDINATIDCNDDVFVLDAAEEAGIDLPYSCRAGACSTCAGKVSDGTID
QSEQTFLDDDQMGAGFVLTCIAYPKSDCTILVHQEDELY
>2Fe-2SST3(2508318168)Micromonas pusilla NOUM17, RCC 299
MSAFTLSTSTVARSAFTGRKVAVRRNAPVAKARGMKVQAFTVTLETPEGKQDISCADDTY
VLDAAEEAGIDLPYSCRAGACSSCAGKVTAGTIDQSDQSFLDDDQMGNGFVLTCVAYPTS
DCTIKTHMEEELY
>2Fe-2SST3(640421541)Ostreococcus lucimarinus CCE9901
MSVTAAPVASFAGARRSSAAVARRSTIARFQVTLETPEGAQAIECADDTYILDAAEEAGIDLPYSCRAGACSSCAGKVTAGSIDQSDQSFLDDDQMGNGFVLTCVAYPTSDCTVKT
HMEEELY

Subtype 4

Archaea

Bacteria

>2Fe-2SST4(1I7H)Escherichia coli
MPKIVILPHQDLCPDGAVLEANSGETILDAALRNGIEIEHACEKSCACTTCHCIVREGFDSLPESSEQEDDMLDKAWGLEPESRLSCQARVTDEDLVVEIPRYTINHAREH
>2Fe-2SST4(3AH7)Pseudomonas putida
MPLVTFLPHEKFCPEGLTVEVKPGTNILELAHDHHIEMESACGGVKACTTCHCIVRKGFDSLEEADELEEDMLDKAWGLEAQSRLGCQVFVADEDLTIEIPKYSLNHAAEAPH

Eukaryota

Subtype 6

Archaea

>2Fe-2SST6(2678207143)Halobacterium hubeiense JI20-1
MVNTLGLLLGLGMALALVGLHLLKGTSKPVPEDIASEVLEQRASTVPETDFPEPYNRSIG
GGGGAGAVAGGAAEGELEEESEEDEGFDPESIPEDEVEYYEIEFVKEGETIEVANNESIL
DAGEEEGWDLPYACRQGQCLSCGGRVASGEDASEFIRHTNNETLSDDEMEKGYMLTCTAH
PTTSFSVETDETP
>2Fe-2SST6(638161117)Halobacterium salinarum NRC-1
MVTTLGVTLGLGVTLLFVVLHLAKGSSKPPAEDIAQDVLEHRASTVPETDFPEPYNRSIG
GGGGAGAVAGGAESELEDGEEEDDGFDPDAIADDDVEYYEIEYVKEGETIEVASNESILD
AGVDEGWDLPYACQQGQCVSCSGRVADGEDAAEYIRHSNNESLGDAEIEEGYMLTCTAHP
TASFSIETDETP
>2Fe-2SST6(641567661)Halobacterium salinarum R1, DSM 671
MVTTLGVTLGLGVTLLFVVLHLAKGSSKPPAEDIAQDVLEHRASTVPETDFPEPYNRSIG
GGGGAGAVAGGAESELEDGEEEDDGFDPDAIADDDVEYYEIEYVKEGETIEVASNESILD
AGVDEGWDLPYACQQGQCVSCSGRVADGEDAAEYIRHSNNESLGDAEIEEGYMLTCTAHP
TASFSIETDETP
>2Fe-2SST6(2507054418)Halobacterium sp. DL1
MVDTLGLALGLGMALTLVGLHFFKGTSKPVPEDIAQEVLERRASTVPETDFPEPYNRSIG
GGGGAGAVAGAGAEGELEESEEADEGFDPESIPEDDVEYYEIEFVKEGETIEVGNNENLL
DAGEEEGWDLPYACRQGQCLSCGGRVASGDDASEFIRHSNNETLGDDEIEKGYMLTCTAY
PTQAFSLETDETP
>2Fe-2SST6(651266909)Haloquadratum walsbyi C23, DSM 16854
MVDINFLGVGIGATLTLLAVSLHYARGTGWTPTADISQEVLEKRASTVPETEFPEPMNRS
IGGGAIPAGSVTGGGGELEETDTEVDESEQVPGDIPDEEAEYFEVEFEKQGSTIEVASNQ
TVLTAGENESWDLPYACRQGQCVSCAGQITSDGNAEDYVEHDNQQMLDDTELEDGYTLTC
VAYPKDDFTIETGEAP
>2Fe-2SST6(649767686)Halogeometricum borinquense PR3, DSM 11551
MVEINLVGLGIGMTLTLMAVALHFSRGTEWTPAADISQEVLERRASTVPETDFPEPMNRS
IGGGGVAAGAVASGEEGAELEGEAEEESSSPADIPEDEVEYFEVEYAKQGATIEVANNET
VLEAGEDEGMDLPYACRQGQCVSCAGQITSGGSSEDYVVHDNQQMLGDAELDDGYTLTCV
AYPKADFTIETGEAP
>2Fe-2SST6(638050380)Haloquadratum walsbyi HBSQ001, DSM 16790
MVDINFLGVGIGATLTLLAVSLHYARGTGWTPTADISQEVLEKRASTVPETEFPEPMNRS
IGGGAIPAGSVTGGGGELEETDTEVDESEQVPGDIPDEEAEYFEVEFEKQGSTIEVASNQ
TVLTAGEDESWDLPYACRQGQCVSCAGQITSGGNAEDYVEHDNQQMLDDAELEHGYTLTC
VAYPKDDFTIETGEAP

Bacteria

>2Fe-2SST6(A9EXQ0)Sorangium cellulosum So ce56
MPTVVFEDGALGPAVTAEAGAGRLIDVCDDARAPIVFSCRDARCATCRVEVVEGEQLLEPPARGERDLLERLGAPPRLRLACQAVLRAGPGLIRLRGVVGREG

Subtype 7

Bacteria

>2Fe-2SST7(5FFI)Azotobacter vinelandii
MATIYFSSPLMPHNKKVQAVAGKRSTLLGVAQENGVKIPFECQDGNCGSCLVKITHLDGERIKGMLLTDKERNVLKSVGKLPKSEEERAAVRDLPPTYRLACQTIVTDEDLLVEFT
GEPGGA

Subtype 8

Archaea

>2Fe-2SST8(2668364098)uncultured marine group II euryarchaeote
MSANAVAATLELDPSSDETRPVIRFLQGPHLLGQTPFVAGKTLVEHAIEAGVEIPTNCTS
GTCGACMVTLLLGDIPLPDPLPPGLDDFMAEEGARLGCIGLPNGDVDVDIRPPL

Bacteria

>2Fe-2SST8(1JQ4)Methylococcus capsulatus str. Bath
MQRVHTITAVTEDGESLRFECRSDEDVITAALRQNIFLMSSCREGGCATCKALCSEGDYDLKGCSVQALPPEEEEEGLVLLCRTYPKTDLEIELPYTH

Subtype 9

Archaea

>2Fe-2SST9(2730749706)Haloferax gibbonsii ARA6
VPTVHFRGREIECDRGDVLRDVLRAAGEPPHNGHSSWFNCRGGGSCGTCAVRVRGPVTYR
TKKERRRLRFPPHDSDSGLRLACQTVVLGDLWVEKYPGFWGQRVEADESETGDAQDAEDA
QGPTG

>2Fe-2SST9(2578421895)Haloferax mediterranei R-4
MPTVHFRGREIECDRGAVLRDVLRGAGEPPHNGHSSWLNCHGRGSCGTCAVRVQGPVTYR
TKKETRRLRLPPHDPDSGLRLACQTLVLGDIWVEKYPGFWGHHVDDDPID
>2Fe-2SST9(2522036473)Haloferax mediterranei R-4
MLRGAGEPPHNGHSSWLNCHGRGSCGTCAVRVQGPVTYRTKKETRRLRLPPHDPDSGLRL
ACQTLVLGDIWVEKYPGFWGHHVDDDPID
>2Fe-2SST9(646700503)Haloferax volcanii DS2, ATCC 29605
MPTVHFRGREIECDRGDVLRDVLRAAGESPHNGHSSWFNCRGGGSCGTCAVRVRGPATYR
TKKERRRLRFPPHDPDSGLRLACQTVVLGDLRVEKYPGFWGQRVEADESETGAVQDAEDA
QEPTD
>2Fe-2SST9(649769286)Halogeometricum borinquense PR3, DSM 11551
MPTVQFRDSEIQCERGAVLRDVLLAAGESPHNGSADMLNCRGRGTCGTCAVAVEGAVTDR
TKREETRLSFPPHHPDSGLRLSCQTRVLGDVTVEKHPGFWGQHVEESERSEDNGTGGGDE
>2Fe-2SST9(648063451)Halalkalicoccus jeotgali B3, DSM 18796
MPTISYEGERIECAVGTELREALLEAGLSPHNGASNYANCGGWAVCGTCAVAVDGAVSEM
SEAERKRLSKWPHDLDSGLRLACQTHIEGDVEVRKYGGFWGQNVDQ
>2Fe-2SST9(2545639667)Halobiforma lacisalsi AJ5, JCM 12983
MPTVYYRGERIECERGAILRDVLLEAGLSPHNGMADTLNCGGHATCGTCAVRVEGDVSEP
TAAERRRLSVPPLRGREGLRLACQTEVRGDLEVRKGEGFWGQREPSDDGRAESDEQSDAP
ESTG
>2Fe-2SST9(643707106) Halorubrum lacusprofundi ATCC 49239
MPTVSYQGEEIECEEGAILRDVLKEAGLSVYNGRAEQFNCRGTGSCGTCAVQVDGAVSEP
GKKEKARLWLPPHHPSHDVRLACQTRVEGDVEVRKGRGLWGQHI
>2Fe-2SST9(646512551) Haloterrigena turkmenica VKM B-1734, DSM 5511
MPTIEFRGREIECERGRILRDVLLEAGESPHNGRANWLNCRGHGTCGTCAVAIEGDASEP
TAAERRRLSLPPHDPDGGLRLSCQTRVDGDLEVRKYDGLWGQRVPDGDAPAASDGPTAPA
DER
>2Fe-2SST9(646647838)Natrialba magadii ATCC 43099
MPTITIRGRDLECERGAVLRDVLLRADESPHNGRADALNCRGLGTCGTCAVAVSGEVGEP
GPRERLRLSTPPHDADSGLRLACQVRVEDDLVVEKYPGFWGQHTDRTEKGNEDTEEP
>2Fe-2SST9(2510573059)Natronobacterium gregoryi SP2
MPTVTIHDRELECETDAVLRNVLLRADESPHNGRADTLNCRGLGSCGTCAVAVSGEVGAP
GSRERLRLSIPPHDTASGLRLACQLRVEDDLVVEKYPGYWGQHTDRTEEKADPEEP
>2Fe-2SST9(2506729650)Natronococcus occultus SP4, DSM 3396

```
MPTVTYEAEEIDCEDGAILRDVLLEAGLSPHNGRAALLNCRGHGTCGTCAVEIDGPVSEP
TTREKRRLSVPPHDPDGGLRLSCQTEVQGDLAVEKHGGFWGQHRAESSRD
>2Fe-2SST9(637723008) Natronomonas pharaonis Gabara, DSM 2160
MPTVTAFGREIECEEGAILRDILLNAGLSPHNGRSDLLNCRGLGTCGTCAVEIDGAVSNI
GRRERSRLAVPPHDPESGLRLACQTRVLGDVTVTKYPGFWGQHTRCESE
>2Fe-2SST9(2730750239)Haloferax gibbonsii ARA6
MAVISLVGVGAGLVLVLLAVALHYSKGTGWEPTADITQEVLERRAATVAETDFPEPMNRS
IGGGGGVAAVAGGEEGAELEGEEAEEEPSPADMPEDEVEYFEVEFVKQGETIELASNETV
LDQGEDAGMDLPYACRQGSCVSCAAKITNGPAEEYITHHTQETLSDEEMDNGYTLTCVAY
PKADFAIETGEAP
>2Fe-2SST9(2522037089)Haloferax mediterranei R-4
MAVISLVGVGAGLVLVLLAVALHFSKGTGWEPTADITQEVLERRAATVAETDFPEPMNRS
IGGGAVAGAVAGGEEGAELEGEEAAEEEQSPADMPEDEVEYFEVEFVKQGETIELASNET
VLDQGEDAGMDLPYACRQGQCVSCAGKITDGPADEYVTHFKQETLSGEEMDKGYTLTCVA
YPKADFAIETGEAP
>2Fe-2SST9(2578423265)Haloferax mediterranei R-4
MAVISLVGVGAGLVLVLLAVALHFSKGTGWEPTADITQEVLERRAATVAETDFPEPMNRS
IGGGAVAGAVAGGEEGAELEGEEAAEEEQSPADMPEDEVEYFEVEFVKQGETIELASNET
VLDQGEDAGMDLPYACRQGQCVSCAGKITDGPADEYVTHFKQETLSGEEMDKGYTLTCVA
YPKADFAIETGEAP
>2Fe-2SST9(646701083)Haloferax volcanii DS2, ATCC 29605
MAVISMVGVGAGLVLVLLAVALHYSKGTGWEPTADITQEVLERRAATVAETDFPEPMNRS
IGGGGGVAAVGAGEEGAELEGEEAEEEQSPADMPEDEVEYFEVEFVKQGETIELASNETV
LDQGEDAGMDLPYACRQGSCVSCAAKITDGPAEEYITHHTQETLSDEEMDNGYTLTCVAY
PKADFAIETGEAP
>2Fe-2SST9(643707096)Halorubrum lacusprofundi ATCC 49239
MLNPLITTIVNPTAIVVAVIATLLVVLVSSLKGRGWEPATDISDEVLQRRAAAVPETEFP
EPGNRSIGGGGGGGAIPAGGEGEEGELEDGAAGGSGPGDIPEDEVEYFEVEFVKQGETVE
LSNNEPILDQGEDQGWDLPYACRQGQCVSCAGRIADGPSEDFVEHDNQQMLEDAEIEDGY
TLTCVAYPRGSFSIETGEAP


>2Fe-2SST9(2598064000)Salinarchaeum sp. Harcht-Bsk1
MELGLAFAVGVGLTVLTVLVHFSRGTGWSPNEDISQAVLEQRAQTVPETDFPEPSNRTFS
AGGGAAVAAVGEGEEGTELEEGEEESSSPGDIPEDEIEYFDVEFVKEGETIEVVNNEPLL
DQGEDHGWDLPYACREGQCVSCAGHIPDGDARDFVEHDDQEMLDDPELEDGYVLTCVAYP
RADFSLETSESP
```

Eukaryota

>2Fe-2SST9(2508313318)Micromonas pusilla NOUM17, RCC 299
MALLASSFAGAAVRALSTRSTTRVDRSKGLVCRAQDKMARTTIDLDKRKIGPGTGKPIKV
TFLGANGQNVVVDCPEDQYILDAGIDAGLELPFTCRGGICGACVAKCTKGSVDHRDIADL
EFTLSEEEQEEGMALLCMCYPVEASEGEGIEIETQSDWGYSLGVAEWKGATGEIGGRSPT
PLMKGDLKGL
>2Fe-2SST9(640421700)streococcus lucimarinus CCE9901
VTFLGAGGSRVDVECGSNQYILDAGLEAGIEIPFTCRGGICGACVARCASGDVDQSDIAD
LEFTLGEDEIEAGMVLLCMARPVGDVEIETQSD

Subtype 10
Bacteria

>2Fe-2SST10(2MJ3)Ehrlichia chaffeensis
MPLITFISPDGSRKTYEAYDGETLLSLAHRNNVDLEGACEGSLACSTCHVIIDPSWYDIVEQHNEISDEENDMLDLAFGLTDTSRLGCQIILTKELDGLCVILPTETRNISFVKNS

Subtype 18

Archaea

>2Fe-2SST18(2522038754)Haloferax mediterranei R-4(2522038754)
MPTIEFKGETIEADVGDNLRQTLLDAGLSPHNGKAQYTNCRGNALCGTCAVEIVEGDVSNPTGKERRRLKLPPHSLDSGLRLSCQLTIEDDLVVEKHPGYWGQKIEQNDS
>2Fe-2SST18(2578424680)Haloferax mediterranei R-4
MPTIEFKGETIEADVGDNLRQTLLDAGLSPHNGKAQYTNCRGNALCGTCAVEIVEGDVSNPTGKERRRLKLPPHSLDSGLRLSCQLTIEDDLVVEKHPGYWGQKIEQNDS

Bacteria

>2Fe-2SST18(fri:FraEuI1c_3227)Frankia sp. EuI1c (Frankia inefficax sp.)
MSIKVTFRLADGSARVVVAEPGTSVMRVAVENGVPGIVGECGGEMSCATCHVHVDPDLAFRSMSADEEDMLEAVDDRLPTSRLGCQLILRDELGEIAVTVPS

Eukaryota

>2Fe-2SST18(2508319934)Micromonas pusilla NOUM17, RCC 299
MSLSIASPASAVRPSIACLSRRRPVGRSSRSQMRVEAASVKVTITPSDGGESITTTVDTA
SVLRTVILDTGAQLYGGMDRLMNCGGMGNCGTCLVDVVEGADLLSEQTEAELRKVKAGKL
KEGWRMSCQCLVGGDAAPEGAELSVVVRPKK

Subtype 20

Bacteria

>2Fe-2SST20(ABB56730.1)Synechococcus elongatus PCC 7942 = FACHB-805
MPSIRFIREDKEVFAADGANLRFKAVENQVDLYTFGGKMMNCGGYGQCGTCIVEIVQGAENLSPRTSFEERKLKRKPDSYRLACQATVNGPVTVLTKPNPKEAQRETLIAQDLARP
IPVTAPPALPQTETEVAGDPPSIATAET

Eukaryota

>2Fe-2SST20(2505189500)Paulinella chromatophora
MPTIRFEQEGQQVGCIEGANLRKAALDAGINPYKGLNNLNNCGGVGQCGTCVVEVIEGVR
NLSPRSDVEEVYLADRPANYRLSCRTSVNGDVTVRTRPPEGVGKGSNSLLGALKALVGRN

>2Fe-2SST20(2507867603)Zea mays mays cv. B73
MAATAHLTGVLGVSSPPLAPSHCSCSGAKKQSCSLRQGRQQRRRLRVARAVEVDAPSAGA
PEPEEAEEPSVDFAFVSPRLLPDGTPDVHYRTARGGQKLRDIMLDGYIDLYGPYDKVLLN
CSGGGVCGTCIVEVVEGKEMLSPKTDVEKELLKRKPKTWRLACQATVGNADSTGQMIIQQ
LPEWKIHEWDK
>2Fe-2SST20(2507861006)Zea mays mays cv. B73
MAATSSACACAAVALASFPSAASTSAPSRVSASSHRGQRHSRAATVRCSSTSPNVSQGAP
APAPPKPQIELEFVGPKPGADGSFPVDRAEAASGEKLLRDVMNENKIELYAAYGKVMNCG
GGGSCGTCIVEIIDGKELLNERTNTENRYLKKKPDSWRLACQTIVGNKENSGKVVVQRLP
QWKR
>2Fe-2SST20(639577863)Arabidopsis thaliana Columbia
MASLGFNLGFTFSNAQIQQHRKVSGGGRARVISCNSSSSSSSQASSPQGISAVTPLEIEL
EFFGPKPGSDGSYPVDKAKAVSGDKLLRSIMQDNKIELYAAYGKVMNCGGGGSCGTCIVE
ILDGRDLLNERTDTENRYLKKKPESWRLACQTIVGNKENSGKSALDIGCRAADSTVEEVN
>2Fe-2SST20(2507829927)Zea mays mays cv. B73
MSGAHRTVRCSLPTVGPATCRAQIPRPTVGPKPGADGSFPLDRAEATSGEKLLCDVMNEN
KIELYVAYGKVMNCGGGGSCGTCIVEIIDGKELLNERTSTENQYLKKKPDSWRLACQTIV
GNKENSGKVVVQRLPQWKR

Subtype 23

Bacteria

>2Fe-2SST23(ABB56928.1)Synechococcus elongatus PCC 7942 = FACHB-805
MTLAETLSVRVRSLGLDQIDRHLFLCADQTKPLCCDRDRSLESWEYLKRRLRELDLDRPDTGKPLVFRTKANCLRVCQEGPILLVYPEGIWYGRVTPEAIERILQEHLLGGQPVQE
LILHQHALPAVDPL

Subtype 24

Archaea

>2Fe-2SST24(1E0Z)Halobacterium Salinarium

PTVEYLNYETLDDQGWDMDDDDLFEKAADAGLDGEDYGTMEVAEGEYILEAAEAQGYDWPFSCRAGACANCASIVKEGEIDMDMQQILSDEEVEEKDVRLTCIGSPAADEVKIVYN
AKHLDYLQNRVI
>2Fe-2SST24(1DOI)Haloarcula marismortui
PTVEYLNYEVVDDNGWDMYDDDVFGEASDMDLDDEDYGSLEVNEGEYILEAAEAQGYDWPFSCRAGACANCAAIVLEGDIDMDMQQILSDEEVEDKNVRLTCIGSPDADEVKIVYN
AKHLDYLQNRVI

>2Fe-2SST24(2506732859)Natronococcus occultus SP4, DSM 3396-A
MTACDDPGHVVELRDADGAVDSLQIPPGESIVDAAESAGIDLPYGCLYGACGTCTARLLE
GRCRHLEPPRALKDDVLEDGYVLACVATPETDCTLRVGHDIQAEAIGTPWR
>2Fe-2SST24(2540570422)Natronomonas moolapensis 8.8.11, DSM 18674-A
MCANAAAHSVELVRPDGRTETIRVENGETIVDAAERADVAVPYGCLYGVCGTCTAELLDG
DVRHRASPRALKTRSLDAEYVLLCIATPETDCRIRIGHGIQAEVVGTPWK
>2Fe-2SST24(648061912)Halalkalicoccus jeotgali B3, DSM 18796-A
MHVVGLERSGKRRETVRVDPDESLLAAAERSGIGLPFGCRIGVCGTCTGRVLDGEMRHVE
PPRALKDRHLEAGYVLTCIATPEVDCTVEVGTDVRARLFENPWR
>2Fe-2SST24(2522038515)Haloferax mediterranei R-4 -A
MATRHDLTLTWRDGREETVPASENETVLEAAESAGIGLPFGCRTGACATCVGRLIDGAIL
YDRPPRALKTRHIEAGYVLCCIARPRTDCRIEVGADVQTDLVSNPWK
>2Fe-2SST24(2578421174)Haloferax mediterranei R-4-A
MATRHDLTLTWRDGREETVPASENETVLEAAESAGIGLPFGCRTGACATCVGRLIDGAIL
YDRPPRALKTRHIEAGYVLCCIARPRTDCRIEVGADVQTDLVSNPWK
>2Fe-2SST24(649769257)Halogeometricum borinquense PR3, DSM 11551-A
MASRHTLTLTRRSGREETTRASEDETILEAAESADISLPFGCRTGACATCVGRLIDGNIS
YDRPPRALKTRHIESGYVLCCIARPRTDCRIEIGPGVQAELVSNPWK
>2Fe-2SST24(2511547557)Haloarcula hispanica CGMCC 1.2049
MTEVLLDWRDSDRTETVSVPDGETILDAAEAADIGLPFGCRTGACGTCTARLLSGDVVHH
RPPRALKEQHLADGYVLLCVAEPTTDAHLAVGATVQAELVPNPWK
>2Fe-2SST24(2558552774)Haloarcula hispanica N601
MTEVLLDWRDSDRTETVSVPDGETILDAAEAADIGLPFGCRTGACGTCTARLLSGDVVHH
RPPRALKEQHLADGYVLLCVAEPTTDAHLAVGATVQAELVPNPWK
>2Fe-2SST24(2629121184)Haloarcula sp. CBA1115
MTEVLLDWRDSDRTETVSVPDGETILDAAEAADIGLPFGCRTGACGTCTARLLSGDVVHH
RPPRALKEQHLADGYVLLCVAEPTTDAHLAVGATVQAELVPNPWK

>2Fe-2SST24(638183788)Haloarcula marismortui ATCC 43049
MTEVLLDWRDSERTETIAVPDGETILDAAAAADIGLPFGCRTGACGTCTARLLSGDVVHH
RPPRALKDRHLADGYVLLCIAEPTTDTHLAVGATVQAELVPNPWK
>2Fe-2SST24(648061773)Halalkalicoccus jeotgali B3, DSM 18796
MGHEITLEWPGDRVETFSVERNETILEAAARVGIRLPYDCRSGTCAECVGQVLEGSIEHRRMPRALEESDRREGYALLCIAVPREDCRIRTGSRLKAELGSSPWG

>2Fe-2SST24(644972966)Halorhabdus utahensis AX-2, DSM 12940
MPEVEYLNFDVVDENEWSIEDEDIFEKAAAADLEDEDYGVLEVEEGEFILDAAEDEGFNW
PFYCREGKCAVCGAEVLEGEIEMPDQEIITEKEKEEDNVRLTCGGTPVEDDTKIIYNAKH
LNLDWYSYV

>2Fe-2SST24(2656728473)Halorhabdus tiamatea SARL4B
MPTVEYLNYEVLDDHGWDMDDDDLFEKAGDAGLDEGDYGTIDVAEGEYILEAAEAQGYDW
PFSCRAGACANCASIVKEGDIDMDMQQILSDEEVEDKGVRLTCVGTPTEEDVKIVYNAKH
LDYLQNRVI
>2Fe-2SST24(644972846)Halorhabdus utahensis AX-2, DSM 12940
MPTVEYLNYEALDDHGWDMDDDDLFEKAESSDLGENDYGAIDVAEGEYILEAAEAQGYDW
PFSCRAGACANCAAIVKEGDIDMDMQQILSDEEVEDKGVRLTCVGTPTEDDVKIVYNAKH
LDYLQNRVI
>2Fe-2SST24(2718530466)Halodesulfurarchaeum formicicum HSR6
MPTVEYINYEVLDDQGWDLEDGDLFEKAADAGLDDQDYGTLDVGEGEYILEAAEAAGYDW
PFSCRAGACANCAGVVKEGDIEMDMQQILSDEEMEEENVRLTCIGSPATDEVQLVYNAKH
LDFLQDRVI
>2Fe-2SST24(2718541652)Halodesulfurarchaeum formicicum HTSR1
MPTVEYINYEVLDDQGWDLEDGDLFEKAADAGLDDQDYGTLDVGEGEYILEAAEAAGYDW
PFSCRAGACANCAGVVKEGDIEMDMQQILSDEEMEEENVRLTCIGSPATDEVQLVYNAKH
LDFLQDRVI

>2Fe-2SST24(2598065433)Salinarchaeum sp. Harcht-Bsk1
MPTVEYLNYDVVDDEGWDMYDDDVFAEAADMDLSDEDYGTLDVNEGEYILEAAEAQGYDW
PFSCRAGACANCAAIVLEGDIDMDMQQILSDEEVDEKNVRLTCIGSPAADDVKIVYNAKH
LDYLQNRVI

>2Fe-2SST24(2511547860)Haloarcula hispanica CGMCC 1.2049
MDDNGWDMYDDDVFAEASDMDLDGEDYGSLEVNEGEYILEAAEAQGYDWPFSCRAGACAN
CAAIVLEGDIDMDMQQILSDEEVEDKNVRLTCIGSPDADEVKIVYNAKHLDYLQNRVI
>2Fe-2SST24(2558553081)Haloarcula hispanica N601
MPTVEYLNYEVVDDNGWDMYDDDVFAEASDMDLDGEDYGSLEVNEGEYILEAAEAQGYDW
PFSCRAGACANCAAIVLEGDIDMDMQQILSDEEVEDKNVRLTCIGSPDADEVKIVYNAKH
LDYLQNRVI

>2Fe-2SST24(2656628125)Halanaeroarchaeum sulfurireducens M27-SA2
MPTVEYLNYETLDDQGWDLDDDDIFEKAADAGFDDEDYGTLDVADGEYILEAAEAQGFDW
PFSCRAGACANCAAILKEGEIDMDMQQILSDEEVEDEGVRLTCIGSPAEDDVKIIYNAKH

LDYLQDRVI
>2Fe-2SST24(2629121471)Haloarcula sp. CBA1115
MPTVEYLNYEVVDDNGWDMYDDDVFAEASDMDLDGEDYGSLEVNEGEYILEAAEAQGYDW
PFSCRAGACANCAAIVLEGDIDMDMQQILSDEEVEDKNVRLTCIGSPDADEVKIVYNAKH
LDYLQNRVI
>2Fe-2SST24(2642205066)Halobacteriaceae archaeon HSR2
MPTVEYLNYETLDDQGWDLDDDDIFEKAADAGFDDEDYGTLDVADGEYILEAAEAQGFDW
PFSCRAGACANCAAILKEGEIDMDMQQILSDEEVEDEGVRLTCIGSPAEDDVKIIYNAKH
LDYLQDRVI
>2Fe-2SST24(651267925)Haloquadratum walsbyi C23, DSM 16854
MATVEYLNYETLDDKGWEMDDNDLFEKAADSDLDTEDYGSLEVNEGEYILEAAEAQGYDW
PFSCRAGACANCAAIVTEGEIDMDMQQILSDEEVSERNVRLTCIGSPAADNIKIVYNAKH
LDYLQNRVIG
>2Fe-2SST24(651269612)Haloquadratum walsbyi C23, DSM 16854
MATVEYLNYESLNDKGWEMDDEDLFEKAADSDLGDEDYGSLEVNEGEYILEAAEAQGYDW
PFSCRAGACANCAAIVTEGEIDMDMQQILSDEEVSDKNVRLTCIGSPAADSVQIVYNAKH
LDYLQNRVI
>2Fe-2SST24(638052699)Haloquadratum walsbyi HBSQ001, DSM 16790
MPWRTCAILMATVEYLNYESLNDKGWEMDDEDLFEKAADSDLGDEDYGSLEVNEGEYILE
AAEAQGYDWPFSCRAGACANCAAIVTEGEIDMDMQQILSDEEVSDKNVRLTCIGSPAADS
VQIVYNAKHLDYLQNRVI
>2Fe-2SST24(638051294)Haloquadratum walsbyi HBSQ001, DSM 16790
MATVEYLNYETLDDKGWEMDDNDLFEKAADSDLDNEDYGSLEVNEGEYILEAAEAQGYDW
PFSCRAGACANCAAIVTEGEIDMDMQQILSDEEVSERNVRLTCIGSPAADNIKIVYNAKH
LDYLQNRVIG
>2Fe-2SST24(648061546)Halalkalicoccus jeotgali B3, DSM 18796
MPTVEYLNYETLDDQGWDIDDDDLFDQAADAGLDEEDYGSLDVNEGEYILEAAEAQGYDW
PFSCRAGACANCAAILKEGEIEMDMQQILSDEEVDDKNVRLTCIGSPAADTVKIVYNAKH
LDYLQNRVI
>2Fe-2SST24(2730751553)Haloferax gibbonsii ARA6
MPTVTYLNYEVLDDNGWDLDDDDLFEQAADAGLDAEDYGEMEVNQGEYILEAAEAQGYDW
PFSCRAGACANCASILKEGEIEMDMQQILSDEEVNDKNVRLTCIGSPVEDEVKIVYNAKH
LDYLQNRVI
>2Fe-2SST24(2522038217)Haloferax mediterranei R-4
MPTVTYLNYEALDDQGWDLDDDDLFEKAADAGLDAEDYGEMEVNQGEYILEAAEAQGYDW
PFSCRAGACANCASILKEGEIEMDMQQILSDEEVNDKNVRLTCIGSPVADEVKIVYNAKH
LDYLQNRVI
>2Fe-2SST24(2578423133)Haloferax mediterranei R-4
MPTVTYLNYEALDDQGWDLDDDDLFEKAADAGLDAEDYGEMEVNQGEYILEAAEAQGYDW

PFSCRAGACANCASILKEGEIEMDMQQILSDEEVNDKNVRLTCIGSPVADEVKIVYNAKH
LDYLQNRVI
>2Fe-2SST24(646702237)Haloferax volcanii DS2, ATCC 29605
MPTVTYLNYEVLDDNGWDLDDDGLFEQAADAGLDAEDYGEMEVNQGEYILEAAEAQGYDW
PFSCRAGACANCAAIVKEGEIDMDMQQILSDEEVNEKNVRLTCIGSPVEDEVKIVYNAKH
LDYLQNRVI
>2Fe-2SST24(2507080798)halophilic archaeon DL31
MPTVEYLNYEVLDDHGWSMDDDDLFEQAADADLSDEDYGNLDVNQGEYILEAAEAQGYDW
PFSCRAGACANCAAIAVEGEIDMDMQQILSDEEVEERNVRLTCIGSPATDEVMIVYNAKH
LDYLQNRVI
>2Fe-2SST24(2741432121)Halorientalis sp. IM1011
MPTVEYLNYEVLDDHGWDMYDNETFDKAAEADLDDEDYGSLDVNEGEYILEAAEAQGYDW
PFSCRAGACANCAAIVVEGEIDMDMQQILSDEEVEDKNVRLTCIGSPDADEVRIVYNAKH
LDYLQNRVI
>2Fe-2SST24(643709246)Halorubrum lacusprofundi ATCC 49239
MMPTVEYLNYEVLDDHGWSMDDDDLFEEAADADLDAEDYGSLDVNQGEYILESAEAQGYD
WPFSCRAGACANCASIVMEGDIEMDMQQILSDEEVEEKNVRLTCIGSPATDEVKIVYNAK
HLDYLQNRVI
>2Fe-2SST24(2540572293)Natronomonas moolapensis 8.8.11, DSM 18674
MPTVEYLNYEVLDDHGWGMDDEDLFESAADADLGDEDYGELDVAEGEYILEAAEAQGYDW
PFSCRAGACANCAAIVKEGEIDMDMQQILSDEEVEEEMVRLTCIGSPAADEVKIVYNAKH
LDYLQNRVI
>2Fe-2SST24(637721177)Natronomonas pharaonis Gabara, DSM 2160
MPTVEYLNYEVLDDHGWEMDDDDLFENAADADLDEEDYGSLDVAEGEYILEAAEAQGYDW
PFSCRAGACANCAAIVKEGEIDMDMQQILSDEEVEEKMVRLTCIGSPAADEVKIVFNAKH
LDYLQNRVI
>2Fe-2SST24(2678209622)Halobacterium hubeiense JI20-1
MPTVEYLNYETLDDQGWDMDDDDLFEKAADAGLDDEDYGTLEVAEGEYILEAAEAQGYDW
PFSCRAGACANCASIVKEGDIDMDMQQILSDEEVEEKNVRLTCIGSPAADEVKIVYNAKH
LDYLQNRVI
>2Fe-2SST24(638160899)Halobacterium salinarum NRC-1
MPTVEYLNYETLDDQGWDMDDDDLFEKAADAGLDGEDYGTMEVAEGEYILEAAEAQGYDW
PFSCRAGACANCASIVKEGEIDMDMQQILSDEEVEEKDVRLTCIGSPAADEVKIVYNAKH
LDYLQNRVI
>2Fe-2SST24(641567444)Halobacterium salinarum R1, DSM 671
MPTVEYLNYETLDDQGWDMDDDDLFEKAADAGLDGEDYGTMEVAEGEYILEAAEAQGYDW
PFSCRAGACANCASIVKEGEIDMDMQQILSDEEVEEKDVRLTCIGSPAADEVKIVYNAKH
LDYLQNRVI
>2Fe-2SST24(649767562)Halogeometricum borinquense PR3, DSM 11551

MPTVEYLNYEVLDDNGWDLDDDDLFENAADAGLDGEDYGTLEVNQGEYILEAAEAQGYDW
PFSCRAGACANCAAIVKEGEIQMDMQQILSDEEVSEKNVRLTCIGSPETDEVKIVYNAKH
LDYLQNRVI
>2Fe-2SST24(645019192)Halomicrobium mukohataei arg-2, DSM 12286
MPTVEYLNYEVVDDKGWDMYDDGVFEDAADADLSDEDYGTLEVNEGEYILEAAEAQGYDW
PFSCRAGACANCAAIVVEGEIDMDMQQILSDEEVEEKNVRLTCIGSPDADEVKIVYNAKH
LDYLQNRVI
>2Fe-2SST24(2506688985)Halopiger xanaduensis SH-6
MPTVEYLNYEVVDDQGWDIYDDEVFEKAADAGLDDEDYGTLDVAEGEYILEAAEAQGYDW
PFSCRAGACANCAAIVVDGEIDMDMQQILSDEEVEEKNVRLTCIGSPETDEVKIVYNAKH
LDYLQNRVI
>2Fe-2SST24(2506860215)Halovivax ruber XH-70, DSM 18193
MPTVEYLNYEVLDDHGWEMDDDDLFDEAADAGLDDEDYGTLEVNEGEYILEAAEAQGYDW
PFSCRAGACANCAAILKEGELEMDMQQILSDEEVEEKNVRLTCIGHALTDEVKIVYNAKH
LDYLQNRVI
>2Fe-2SST24(2718328807)Natrialbaceae archaeon JW/NM-HA 15
MPTVEYLNYEVVDDNGWDIYDEDVFETAADEGLDEEDYGTLEVAEGEYILEAAEAQGFDW
PFSCRAGACANCAAIVVDGEIEMDMQQILSDEEVDEKNVRLTCIGSAETDEVKIVYNAKH
LDYLQNRVI
>2Fe-2SST24(648062459)Halalkalicoccus jeotgali B3, DSM 18796(648062459)
MSSSLPNIKVGRDAEADMDPEELPVHEIEYLNYEAIPEHGWDLDDDDLFEKAAAADLTEK
DHGTFEIRGTRYILDGAEDEGQKWPFECRAASCAYCTMFLYEGDVKMDMDLILTDEEVEE
RNIYLSCQSIPKSEEVKVVYNAMLADYLQENIVGVREV
>2Fe-2SST24(2507057054)Halobacterium sp. DL1
MPTVEYLNYETLDDQGWDMDDDDLFEKASDAGLDEEDYGTLDVAEGEYILEAAEAQGYDW
PFSCRAGACANCAAIVFDGEIDMDMQQILSDEEVEEKDVRLTCIGSAQTDEVKIVYNAKH
LDYLQNRVI
>2Fe-2SST24(2545639490)Halobiforma lacisalsi AJ5, JCM 12983(2545639490)
MMPTVEYLNYEVLDDQGWDMDDDDLFEQAADADLDDEDYGSLEVNEGEYILEAAEAQGYD
WPFSCRAGACANCAAIVKEGEIDMDMQQILSDEEVEEKNVRLTCIGSAETDEVKIVYNAK
HLDYLQNRVI
>2Fe-2SST24(2510276469)Halostagnicola larsenii XH-48, DSM 17691(2510276469)
MPTVEYLNYEVLDDQGWEMDDDDLFETAADAGLDEEDYGTLEVAEGEYILEAAEAQGYDW
PFSCRAGACANCAAIITEGEIEMDMQQILSDEEVEEKNVRLTCIGSAETDEVRIVYNAKH
LDYLQNRVI
>2Fe-2SST24(2741434959)Haloterrigena daqingensis JX313(2741434959)
MPTVEYLNYEVLDDQGWEMDDDDLFDQAADAGLDEEDYGSLDVAEGEYILEAAEAQGYDW
PFSCRAGACANCAAIIKEGEIQMDMQQILSDEEVEDKNVVLTCIGSAETDEVKIVYNAKH
LDYLQNRVI

>2Fe-2SST24(646510662)Haloterrigena turkmenica VKM B-1734, DSM 5511(646510662)
MPTVEYLNYEVLDDQGWDMDDDDLFEQAADAGLDDEDYGTLDVAEGEYILEAAEAQGYDW
PFSCRAGACANCAAIVFEGEIDMDMQQILSDEEVEEKDVRLTCIGSAETDEVKIVYNAKH
LDYLQNRVI
>2Fe-2SST24(646645505)Natrialba magadii ATCC 43099(646645505)
MPTVEYLNYEVLDDQGWEMDDDDLFEKAADADFGEEDYGSLEVNEGEYILEAAEAQGYDW
PFSCRAGACANCAAIVTEGEIQMDMQQILSDEEVEDKNVRLTCIGSAETDEVKMVYNAKH
LDYLQNRVI
>2Fe-2SST24(2509882554)Natrinema pellirubrum 157(2509882554)
MPTVEYLNYEVLDDHGWDMDDEDLFDEAADAGLDDEDYGSLDVAEGEYILEAAEAQGYDW
PFSCRAGACANCAAIVKEGEIDMDMQQILSDEEVEEKNVRLTCIGSPETDEVKIVYNAKH
LDYLQNRVI
>2Fe-2SST24(2517200551)Natrinema sp. J7-2(2517200551)
MPTVEYLNYEVLDDQGWDMDDDDLFEQAADAGLDEEDYGSLEVAEGEYILEAAEAQGYDW
PFSCRAGACANCAAIVFEGEIDMDMQQILSDEEVEEKNVRLTCIGSAETDEVKIVYNAKH
LDYLQNRVI
>2Fe-2SST24(2510572065) Natronobacterium gregoryi SP2(2510572065)
MPTVEYLNYEVLDDQGWDMDDDDLFEQAADADLGDEDYGSLEVNEGEYILEAAEAQGYDW
PFSCRAGACANCAAIVKEGEIDMDMQQILSDEEVEDKDVRLTCIGSAETDEVKIVYNAKH
LDYLQNRVI
>2Fe-2SST24(2506730473) Natronococcus occultus SP4, DSM 3396(2506730473)
MSPNLPHVRLGEDAAERGDSEADEGPVRTVEYLDYEVLSERGWDIEDDDLFEKAAEADLD
DTDHGRIEVTGRRYILDAAEDQGFEWPYECRAASCANCAGIVYEGDVEMDMDLILTEEEV
EERQITLTCQSIPKSDEVKLVYNAMYLDYLQDRVIGVREV
>2Fe-2SST24(2506728776)Natronococcus occultus SP4, DSM 3396
MPTVEYLNYEVLDDQGWEMDDDDLFEQAADAGLDEEDYGTLDVAEGEYILEAAEAQGYDW
PFSCRAGACANCAAIVYEGEIDMDMQQILSDEEVEDKNVRLTCIGSAETDEVQIVYNAKH
LDYLQNRVI

>2Fe-2SST24(2510276562)Halostagnicola larsenii XH-48, DSM 17691
MVETYTVEFIDEGRTIEVPANKPVLEAAEEAGLTPPYQCRMGVCGVCCGMVVEDGEVDQTE
GMFLSESEKEEGYALTCIAKPRSDLRIRTDESP
>2Fe-2SST24(646514724)Haloterrigena turkmenica VKM B-1734, DSM 5511
MVESYTVEFVDEGQAIEVPANKPILEAAEEAGLAPPYQCRMGVCGVCCGLVVEDGEVDQT
EGMFLSDSEKEEGYALTCIAKPRSDLRIRTDESP
>2Fe-2SST24(2506729454)Natronococcus occultus SP4, DSM 3396
MVETHTVEFVDEGRTLEIPENQPILEAAEEAGLTPPYQCRMGVCGVCCGMIVEDGVVEQT
EGMFLSDSEKEEGYALTCVAKPRSDLRIRTDESP

>2Fe-2SST24(2511547562)Haloarcula hispanica CGMCC 1.2049
MVELVGLAAGATLTLIVVALHYAKGTGWEAPEDISQEVLEQRAATVPETDFPEPYNRSIG
GGGATAIPAGEAEGELGEGEEAEEEDEGFDPDDIAEDEVEYYEIEFAKEGETIEVANNEP
LLDAGEEEGWDLPYACREGQCISCAGHIEDGPAEDYIRHSNNDSLMDDDMEEGYCLTCVA
YPTSDFTLETGESP
>2Fe-2SST24(2558552779)Haloarcula hispanica N601
MVELVGLAAGATLTLIVVALHYAKGTGWEAPEDISQEVLEQRAATVPETDFPEPYNRSIG
GGGATAIPAGEAEGELGEGEEAEEEDEGFDPDDIAEDEVEYYEIEFAKEGETIEVANNEP
LLDAGEEEGWDLPYACREGQCISCAGHIEDGPAEDYIRHSNNDSLMDDDMEEGYCLTCVA
YPTSDFTLETGESP
>2Fe-2SST24(638183793)Haloarcula marismortui ATCC 43049
MVELVGLAAGATLTLIVVALHYAKGTGWEAPEDISQEVLEQRAATVPETDFAEPYNRSIG
GGGAVAIPAGEAEGELGEGEAVEEEDEGFDPDDIADDEVEYYEIEFAKEGETIEVANNET
ILDAGEEEGWDLPYACREGQCISCAGHIEDGPATDHIRHSNNDSLMDDDMEEGYCLTCVA
YPTSEFTIETGESP
>2Fe-2SST24(2629121189)Haloarcula sp. CBA1115
MVELVGLAAGATLTLIVVALHYAKGTGWEAPEDISQEVLEQRAATVPETDFPEPYNRSIG
GGGATAIPAGEAEGELGEGEEAEEEDEGFDPDDIAEDEVEYYEIEFAKEGETIEVANNEP
LLDAGEEEGWDLPYACREGQCISCAGHIEDGPAEDYIRHSNNDSLMDDDMEEGYCLTCVA
YPTSDFTLETGESP
>2Fe-2SST24(645018736)Halomicrobium mukohataei arg-2, DSM 12286
MVEPVSVAAGAVLTIVVVAVHYSSGTGWEPSEDISQNVIEKRAATVPETDFPEPYNRSIG
GGGSAAAIPAGEEGAELEEGAEEADDGFDPDEIADDEVEYYEIEFAKEGETIEVANNETI
LDAGEDEGWDMPYACRQGQCVSCAGQITDGAASDYIRHSQNESLFDDDMEEGYCLTCVAY
PTDEFTIETGEQP
>2Fe-2SST24(2656727456)Halorhabdus tiamatea SARL4B
MVEVLGIALGVGFTLTAVLLHYAKGTGWEPAEDVSTTLLERRAAAVPETDFAEPMNRSIG
GGGAAIPAGTADAEGELAGDAEEAETGFDPEAIPDEEVEHYEIEFGKEGETIEVANNETI
LEAGEDEDWDLPYSCRQGQCLSCSGHIADGPAEDFVKHSNNDTLSDEEMENGYCLTCTAH
PTTDFTIETGETP

>2Fe-2SST24(644971659)Halorhabdus utahensis AX-2, DSM 12940
MVEALGIALGIGFTFTAVLLHYAKGTGWEPAEDISTTLLERRAAAVPETDFPEPMNRSIG
GGGAAIPAGGDAEGELADGDADEADQGFDPEAIPEDEVEHYEIEFAKEGKTIEVANNETI
LEAGEDEDWDLPYSCRQGQCLSCSGHVEDGPAEDFVKHSNNDTLSEEEMENGYCLTCTAH
PTTDFTIETSETP
>2Fe-2SST24(2741431742)Halorientalis sp. IM1011
MVDAGILTGAFLTLVAVALHYSKGTGWEANEDISQEVLEKRAETVPETEFPEPMNRSIGG
GGVAAGAVAGGSEGELAEGGEEEDEGFDPDAIADDEVETYEIEFAKEGETIEIANNENIL

DVGEDQGWDLPYACREGSCLSCAGHIEEGPAEEYIQHSNNDTLNEEEMDNGYCLTCTAYP
TDSFTLETGEQP

>2Fe-2SST24(2507080499)halophilic archaeon DL31
MADYTVEFVRTGETIEIPETQTILKACLEAGIAQEYSCRVGMCLACSAKIIEGEVEQTVA
EHRALTPEEAEEYALTCMARPQSDLKLDRGKYPPSIEDMGAEPGDAAVSDDD

Bacteria

Eukaryota

>2Fe-2SST24(3AV8)Aphanothece sacrum
ASYKVTLKTPDGDNVITVPDDEYILDVAEEQGLDLPYSCRAGACSTCAGKLVSGPAPDQSDQSFLDDDQIQAGYILTCVAYPTGDCVIETHKEEALY

Subtype 25

Eukaryota

>2Fe-2SST25(1L5P)Trichomonas vaginalis
GTITAVKGGVKKQLKFEDDQTLFTVLTEAGLMSADDTCQGNKACGKCICKHVSGKVAAAEDDEKEFLEDQPANARLACAITLSGENDGAVFEL

Subtype 31

Archaea

>2Fe-2SST31(2509882665)Natrinema pellirubrum 157
MTSHDVTLEWPDGRTRTIAVREDETVLEAAERDDSTLPFGCRTGACGTRTGRLLEDGDDALTHRRQPRVLKDRHRAAGYVLLCLASPRADCRIAVGSSVHSELVDNPWK

Subtype 36

Bacteria

>2Fe-2SST36(A9EY18)Sorangium cellulosum So ce56
MAKVRFLAHGRAWEVEAPVGSSVLQASKSVGAPEGDACGGVCACSTCHVYVTKGRELLSEAEEDEEDILDKAFDVRSTSRLGCQARILKDGDIEAEISRESLDAFYNEHPNVKDPR
KG

Subtype 37

Bacteria

>2Fe-2SST37(A9GDT7)Sorangium cellulosum So ce56
MPQRKRYLFVCVNRRPDGVPKGSCAQRGAEGIHVQLKAALAERGLAKVEARACSASCLDVCWAGPVIAVEPDGYFYGRVTPADVPEIVDALASGRRVERLVLPPEDFTEATAAPPL
PAPP
RGAPPQA

Subtype 38

Archaea

>2Fe-2SST38(2678207658)Halobacterium hubeiense JI20-1
MTEYTVEFVGTGEEIQVSDKETILSACLEEGIAQEYSCRVGMCLACSAKIEAGDVTQPAA
RGLTEEEAENYALTCMARPQSDLKLDRGKYPPSIEDDAAAAAPAADDD
>2Fe-2SST38(638159411)Halobacterium salinarum NRC-1
MTEYTVEFAGAGETIEVSDTETILKACLREGIAQEYSCRVGMCLACSAKIESGDVTQPAA
RGLTDAEADEYALTCMARPQTDLVLDRGKYPPSIEEEAAVADQAAADDD
>2Fe-2SST38(641565953)Halobacterium salinarum R1, DSM 671

MTEYTVEFAGAGETIEVSDTETILKACLREGIAQEYSCRVGMCLACSAKIESGDVTQPAA
RGLTDAEADEYALTCMARPQTDLVLDRGKYPPSIEEEAAVADQAAADDD
>2Fe-2SST38(2507055119)Halobacterium sp. DL1
MTEYTVEFVGTGEEIQVSGTETVLKACLREGIAQEYSCRVGMCLACSAKIEEGEVTQPAA
RGLTDEEAEEYALTCMARPQSDLVLDRGKYPPSIEQDAAAADAAAADDD

>2Fe-2SST38(2522036761)Haloferax mediterranei R-4
MTEYTVEFVGTGETITVSDKQTILKACIEEGIAQEYSCRVGMCLACSAEILEGDVTQPAA
RGLTEEESEKYALTCMARPQSDLKLERGIYPPSIEDDAVTAGAAADDD

>2Fe-2SST38(649769050)Halogeometricum borinquense PR3, DSM 11551
MTEYTVEFVGTGESIQVSDKQTILKACIEEGIAQEYSCRVGMCLACSAEILEGDVVQPAA
RGLTEEEREEYALTCMARPQSDLKLKRGVYPPSIEEDAASAAAAADDD

>2Fe-2SST38(2540570360)Natronomonas moolapensis 8.8.11, DSM 18674
MTEYTVEFVGTDETITVSDKQTILKRCIEEGIAQEYSCRVGMCLACSAEILEGDVAQPAA
RGLTDEESESYALTCMARPQSDLKLDRGKYPPSIDDTAASEGSEPASADD
>2Fe-2SST38(2598063374)Salinarchaeum sp. Harcht-Bsk1
MTDYTVEFVGTGETITVADTETILSRCIEEGIAQEYSCRVGMCLACSAEILEGEVTQPAA
RALTEDEAERFALTCMARPQSDLKLDRGVYPPSIEDDAETTGDATAAADD

>2Fe-2SST38(648064315)Halalkalicoccus jeotgali B3, DSM 18796
MATYTVEFVGTGETIEVTDKQTILSRCLEEGIAQEYSCRVGMCLACTAEIVEGEVTQPAA
RGLSEAESEAFALTCMARPQSDLKLDRGKYPPSIAVDERANHTVNGSGSGVVADDD
>2Fe-2SST38(2642205689)Halobacteriaceae archaeon HSR2
MTEYTVEFVGAGETIQVSENETILTAAVREGISQEFSCRVGMCLACSAEIVEGEVTQPAA
RGFTDEEAEDYALTCVARPESDLRIERGQYPPSIEEEATGANAAADD

>2Fe-2SST38(2718531799)Halodesulfurarchaeum formicicum HSR6
MTTHTVEFVGTGEEIEVAETETILDVAVREGISQEYSCRVGMCLACSAEIIEGEVTQPGA
RGLTEAEAENYALTCMARPETDLKLDRGSYPPSIEAEEPTGEPADD
>2Fe-2SST38(2718542893)Halodesulfurarchaeum formicicum HTSR1
MTTHTVEFVGTGEEIEVAETETILDVAVREGISQEYSCRVGMCLACSAEIIEGEVTQPGA
RGLTEAEAENYALTCMARPETDLKLDRGSYPPSIEAEEPTGEPADD

>2Fe-2SST38(2511546217)Haloarcula hispanica CGMCC 1.2049
MTEYTVTFVGTGEEITVSEKETILSRCIEEGIAQEYSCRVGMCLACSAKIVEGSVTQPAA

RGLTDREREDYALTCMARPQSDLKLDRGKYPPSIEADVDLEDGDPTPADD
>2Fe-2SST38(2558551415)Haloarcula hispanica N601
MTEYTVTFVGTGEEITVSEKETILSRCIEEGIAQEYSCRVGMCLACSAKIVEGSVTQPAA
RGLTDREREDYALTCMARPQSDLKLDRGKYPPSIEADVDLEDGDPTPADD
>2Fe-2SST38(638182364)Haloarcula marismortui ATCC 43049
MTEHTVTFVGTGEEITVSEKETILSRCIEEGIAQEYSCRVGMCLACSAEIVEGSVTQPAA
RGLTDRERENYALTCMARPQSDLKLDRGKYPPSIEGDVSPEDGDPTPADD
>2Fe-2SST38(2629119461)Haloarcula sp. CBA1115
MTEYTVTFVGTGEEITVSEKETILSRCIEEGIAQEYSCRVGMCLACSAKIVEGSVTQPAA
RGLTDREREDYALTCMARPQSDLKLDRGKYPPSIEADVDLEDGDPTPADD

>2Fe-2SST38(2545640349)Halobiforma lacisalsi AJ5, JCM 12983
MTEYTVEFVGTGETITCSDKETILSRCLEEGIAQEYSCRVGMCLACSAEIVEGEVTQPAA
RGLTEEEAEEYALTCMARPQSDLKLDRGKYPPSIESEIDAEGAEGHAAADD
>2Fe-2SST38(645021473)Halomicrobium mukohataei arg-2, DSM 12286
MTEYTVEFVGTGEEITVSEKETILSRCLEEGIAQEYSCRVGMCLACSAEIVDGDVVQPAA
RGLTEEERDGYALTCMARPASDLKLDRGKYPPSIEAAGSADAAGDEQAAADD
>2Fe-2SST38(2506691762)Halopiger xanaduensis SH-6
MTEYTVEFVGTGETITCSDTETILSRCLEEGIAQEYSCRVGMCLACSAEIVEGDVTQPAA
RALTEEEAENYALTCMARPQSDLKLDRGKYPPSIEDEIDAAGEGGSDDPAVADD
>2Fe-2SST38(651268380)Haloquadratum walsbyi C23, DSM 16854
MTEYTVEFLGTGETIEVSNKQTILKACIEAGIAQEYSCRVGMCLACSAEIVEGDVVQPAA
RGLTETERDNYALTCMARPQSDLKIRRGVYPPSIEQDATQSDMAADD
>2Fe-2SST38(638051682)Haloquadratum walsbyi HBSQ001, DSM 16790
MTEYTVEFLGTGETIEVSNKQTILKACIEAGIAQEYSCRVGMCLACSAEIVEGDVVQPAA
RGLTETERDNYALTCMARPQSDLKIRRGVYPPSIEQDATQSDMAADD
>2Fe-2SST38(2656727860)Halorhabdus tiamatea SARL4B
MTAYTVEFVGTGETIEVEDTETVLSRAIEEGIAQEYSCRVGMCLACSAKIVEGDVTQPAA
RGLTDEEKENYALTCMARPQSDLKLDRGKYPPSIEKEAAVEAGAGDEATADD
>2Fe-2SST38(644971946)Halorhabdus utahensis AX-2, DSM 12940
MAVYTVEFVGTGETIEVEDTDTILSRCLDEGIAQEYSCRVGMCLACSAKIIDGEVTQPAA
RGLTDKEKENYALTCMARPQSDLKLDRGKYPPSIEAEAAVEAGAGDEAAADD
>2Fe-2SST38(2741429520)Halorientalis sp. IM1011
MTDYTVEFAGTGEQITVSETETILRRCIEEGIAQEYSCRVGMCLACSAKVVEGEVTQPAA
HGLTDAEREEYALTCMARPQSDLVLDRGKYPPSIEADAEETAGAAADD
>2Fe-2SST38(643708491)Halorubrum lacusprofundi ATCC 49239
MTEYTVEFVGTGETIEVADTETILQPCIEEGIAQEYSCRVGMCLACSAEIVEGEVTQPAA
RGLTDEEAEEYALTCMARPQSDLKLDRGKYPPSIEDDAATDASDVDGAAADDD
>2Fe-2SST38(2510274300)Halostagnicola larsenii XH-48, DSM 17691

MTEYTVEFVGTGETITCSDKETILSRCLEEGIAQEYSCRVGMCLACSAEIVEGEVTQPAA
RGLTEAEAEEYALTCMARPNSDLKLDRGKYPPSIEAEIDSEGANGAATADD
>2Fe-2SST38(2741435756)Haloterrigena daqingensis JX313
MTEYTIEFVGTGETITCSDTETILSRCLEEGIAQEYSCRVGMCLACSAEILEGEVVQPAA
RGFTEAEAENYALTCMARPQSDLKLERGVYPPSIDGDLESEGTDGAAVADD
>2Fe-2SST38(646511953)Haloterrigena turkmenica VKM B-1734, DSM 5511
MTEYTVEFVGTGETITCSDTETILSRCLEEGIAQEYSCRVGMCLACSAEIVEGEVTQPAA
RGLTEEEAENYALTCMARPQSDLTLDRGKYPPSIESDLEADGSNAGATADD
>2Fe-2SST38(2506859546)Halovivax ruber XH-70, DSM 18193
MTEYTVEFVGTGETITCSDKETILSRCLEEGIAQEYSCRVGMCLACTAEIVEGEVTQPAA
RGFTEEEAEHYALTCMARPQSDLKLERGKYPPSIEGDATSAAGEAFGDD
>2Fe-2SST38(646646543)Natrialba magadii ATCC 43099
MTEYTIEFVGTGETITCSDKETILSRCLEEGIAQEYSCRVGMCLACSAEIIEGEVTQPAA
RGFTEEEAEAYALTCMARPQSDLKLERGKYPPSIESDLEAGEAGNAGGDTAAADD
>2Fe-2SST38(2718329544)Natrialbaceae archaeon JW/NM-HA 15
MTEYTVEFVGTGETITCSDKETILSRCLEEGIAQEYSCRVGMCLACSAEIIEGEVTQPAA
RGFTEQEAENYALTCMARPQSDLKLERGVYPPSIEGDLSGDAAPADD
>2Fe-2SST38(2509885737)Natrinema pellirubrum 157
MTEYTIEFVGTGETITCTDKETILSRCLEEGIAQEYSCRVGMCLACSAEIIAGEVTQPAA
RGLTEEEAENYALTCMARPQSDLKLDRGKYPPSIEGDLEAVSSDGAVADD

>2Fe-2SST38(2517204098)Natrinema sp. J7-2
MTEYTIEFVGTGETITCSDKETILSRCLEEGIAQEYSCRVGMCLACSAEIIDGEVTQPAA
RGLTDEEAENYALTCMARPQSDLKLDRGKYPPSIEGDLEATGAIDSAAADD
>2Fe-2SST38(2510571278)Natronobacterium gregoryi SP2
MTDYTVEFVGTGETITCSDKETILSRCLEEGIAQEYSCRVGMCLACSAEIVEGEVTQPAA
RAFTDEEAENYALTCMARPQSDLKLERGKYPPSIDDDVAAEAGGDVETGSPADD
>2Fe-2SST38(2506729413)Natronococcus occultus SP4, DSM 3396
MTEYTVEFVGTGESITCTDKETILSRCLEEGIAQEYSCRVGMCLACSAEIVEGEVTQPAA
RGFTEEEAENYALTCMARPQSDLKLERGKYPPSIEGDLAGGEPARADD
>2Fe-2SST38(637722834)Natronomonas pharaonis Gabara, DSM 2160
MTDYTVEFVGTGETITVSDKDTILSRCIEEGIAQEFSCRVGMCLACSAEIIEGEVTQPAA
RGLTDAEREDYALTCMARPQSDLKLDRGKYPPSIEDEVDVDADGTDAAAADD
>2Fe-2SST38(2730749936)Haloferax gibbonsii ARA6
MTEYTVEFVGTGETITVSDKQTILKACIEEGIAQEYSCRVGMCLACSAEILEGDVTQPAA
RGLTDEEAERFALTCMARPQSDLKLDRGVYPPSIEGDAAAAGTAADDD
>2Fe-2SST38(2578421604)Haloferax mediterranei R-4
MTEYTVEFVGTGETITVSDKQTILKACIEEGIAQEYSCRVGMCLACSAEILEGDVTQPAA

RGLTEEESEKYALTCMARPQSDLKLERGIYPPSIEDDAVTAGAAADDD
>2Fe-2SST38(646700738)Haloferax volcanii DS2, ATCC 29605
MTEYTVEFVGTGETITVSDKQTILKACIEEGIAQEYSCRVGMCLACSAEILEGDVTQPAA
RGLTEEEAERFALTCMARPQSDLKLDRGVYPPSIEDDAAAAGTAADDD
>2Fe-2SST38(2656628749)Halanaeroarchaeum sulfurireducens M27-SA2
MTEYTVEFVGAGETIQVSENETILTAAVREGISQEFSCRVGMCLACSAEIVEGEVTQPAA
RGFTDEEAEDYALTCVARPESDLRIERGQYPPSIEEEATGANAAADD

Eukaryota

>2Fe-2SST38(ath:AT4G14890)Arabidopsis thaliana
MATLPLPTQTSTISLPKPYLSNSFSFPLRNATLSTTTNRRNFLTTGRIIARAYKVVVEHD
GKTTELEVEPDETILSKALDSGLDVPYDCNLGVCMTCPAKLVTGTVDQSGGMLSDDVVERGYTLLCASYPTSDCHIKMIPEEELLSLQLATAND
>2Fe-2SST38(639575667)Arabidopsis thaliana Columbia
MATLPLPTQTSTISLPKPYLSNSFSFPLRNATLSTTTNRRNFLTTGRIIARAYKVVVEHD
GKTTELEVEPDETILSKALDSGLDVPYDCNLGVCMTCPAKLVTGTVDQSGGMLSDDVVER
GYTLLCASYPTSDCHIKMIPEEELLSLQLATAND

>2Fe-2SST38(2507802064)Zea mays mays cv. B73
MAAASASLLHLATPTLTRSTRLAVRLPSAQLSRWPSGARIAAAQPPRAYKVTIEHGGESR
VVEVEEDETILSRALDEGLDVPHDCKLGVCMTCPARLVSGTVDQSDGMLSDDVVARGYAL
LCAAYPRSDCTIRVIPEDELLKVQLATADD

Subtype 39

Archaea

>2Fe-2SST39(2545639906)Halobiforma lacisalsi AJ5, JCM 12983

MTEYDVTLEWPDGGTDTVAVDSRDTVLEAAQQEGIRLPADCRKGTCTTCVGRVVGVDGDGAAGASGPVDAAEAFDYRRQPAALTDGERADGYVLLCIALPRADCRVAVGPAIRAEV
GDSP
WA
>2Fe-2SST39(2510572047)Natronobacterium gregoryi SP2

MTQYDVTLEWPDDGTDTVAVSPTETVLEVALREGIRLPYDCREGTCITCVGRVLAAGGDETPGAEEAVDAADAFDYRRQPKALTEDERANGYVLLCIAVPRADCRVAVGPMVRSEV
GDSP
WS

Subtype 40

Archaea

>2Fe-2SST40(646645851)Natrialba magadii ATCC 43099
MTSYEVVLERPGSPDHTLEVSKRETILEAARRDGVRLPADCLKGTCTTCVGRVVGVEGEDDGDDETTDSRPDAALAVDYRRPPQALAGHERADGYVLLCIALPRADCRIEAGPQVR
AEVG
DSPWR
>2Fe-2SST40(2545639904)Halobiforma lacisalsi AJ5, JCM 12983
MTSHDVTLEWPDGRTRTIVVREGETVLEGAERADVALPFGCRTGACGTCTGRLLGSEGSEPTATATETGRVDVGDAFSSRRRPRALKDRHREAGYVLLCIATPRTDCRFAVGSSVH
TELV
DNPWK
>2Fe-2SST40(2517200684)Natrinema sp. J7-2
MVTLEWPDGRTRTVAVREDETVLDGAERADISLPFGCRTGACGTCTGRLLAVDGAESEPDSGDAEDPDIGATIAYTRPPRALKDRHRADGYVLLCLASPRADCRIAVGSSVQSELV
ENPW
K

Subtype 41

Archaea

>2Fe-2SST41(2506732578)Natronococcus occultus SP4, DSM 3396(2506732578)
MSSYDVTLEWSDGRSRTVAVEPRETILEAALREGVRLPYDCRKGTCTACAGRVVDLEDDAEANDGPPDAAAAVDYRRPPRALEESDRADGYALLCIAQPRADCRIEVGPMVRSQLG
DSPW
G

>2Fe-2SST41(2517200686)Natrinema sp. J7-2
MTSYDVTLEWTDGRTRTIDVGESQTVLEAAQRLGVRLPYDCRAGTCITCVGRLLALEDGDDGTDRPLDVADALGYQRPPQALTDDERDDGYVLLCIASPRADCRIEVGPRVRAEVG
DSPWA

Subtype 42

Archaea

>2Fe-2SST42(2506689360)Halopiger xanaduensis SH-6

MTYDVTLEWPDGRTETIDAPESESVLDAAQRAGVRLPYDCRKGTCITCVGRLLEVDGEGEREATDADSDGDGTDADSDADDSAGAPHPADAFAYRRSPAALTEGERADGYVLLCIA
QPRADCRLEVGPQIRAQVGDSPWA

Subtype 43

Archaea

>2Fe-2SST43(646514235)Haloterrigena turkmenica VKM B-1734, DSM 5511
MTSHDVTLEWADGSTQTVAVAENESVLDAAQRAGARLPYDCRKGTCITCVGRLLALEGEDAESAEGGSEQPPDPADVFTYRRSPAALTDQEQADGYVLLCVAHPQSDCRIEVGPRV
RAEVGDSPWA
>2Fe-2SST43(2545642570)Halobiforma lacisalsi AJ5, JCM 12983
MPTIRFDGERIECEDGRNLLRALPRGALSSATPVSLCGNGVCGMCAVTVEGETNELTEAERNRLSEPCEDSPCEGGDGNGDGDETDVDRSRRRLACQTEVHGDLEVTMDE

Subtype 44

Archaea

>2Fe-2SST44(2509882667)Natrinema pellirubrum 157
MTTHEVTLEWTDGRTRTLDVAESQTVLEAAQRVGARLPYDCRSGTCITCVGRLLGLEDGETDTEEGARPDVREAFAYRRQPRALTDDEQGEGYVLLCIASPQADCRIEVGPRVRAE
VGDS
PWA
>2Fe-2SST44(2506689358)Halopiger xanaduensis SH-6
MASHEVVLEWPEADRERRTIAVRADETVLEGAERADVALPFGCRTGACGTCTGRLLSVGGTDATGTNSEPTTVDEAFDYRRPPRALKDRHRAAGYVLLCIAAPRADCRLAVGSSVH
AELV
ENPWK

Subtype 45

Archaea

>2Fe-2SST45(646514233)Haloterrigena turkmenica VKM B-1734, DSM 5511
MVSQRHDVTLEWPDADRETRTIAVDEDETVLEAAERSGIALPFGCRTGACGTCTGRLLEADGAEPTAADDERTVDVDGAFSYRRSPRALKDRHRTAGYVLLCIASPRTDCRLAVGA
NVHTELVENPWK

Subtype 46

Archaea

>2Fe-2SST46(651268525)Haloquadratum walsbyi C23, DSM 16854
MTMSENTDSIPVIVIDTDTKTKITVSIGTNLRDALRNHDFSPYGRLSETLNCGGNGLCAT
CGVRVRVYNNQQNDIADTQIHTDDNDIMYPDSGPTPNHWHDRLAARFGYPRLSCQITVSE
PLVVQLLPKKLLWGGRKTKTEVTDSMRTNDADSNYR

>2Fe-2SST46(651268525)Haloquadratum walsbyi HBSQ001, DSM 16790(638051799)
MTMSENTDSIPVIVIDTDTKTKITVSIGTNLRDALRNHDFSPYGRLSETLNCGGNGLCAT
CGVRVRVYNNQQNDIADTQIHIDDNDIMYPDSGPTPDHWHDRLAARFGYPRLSCQITVSE
PLVVQLLPKKLLWGGRKTKTEVTDSMRTNDVDSNYR

Subtype 47

Archaea

>2Fe-2SST47(2540571832)Natronomonas moolapensis 8.8.11, DSM 18674
MNESVTLTVREDGAETTIEAERGAILRDVLRSHGVAVYGSVSRVANCGGRGLCGTCGVRVEGAEGGSIPAAGLHDAAAKRWGYPRLSCRIRVTEPMTVEVIEKVVWGQLRPE

Subtype 48

Archaea

>2Fe-2SST48(2730750180)Haloferax gibbonsii ARA6
MGDSESRPERDPPGPTVTVHVRDTDGKVVTDLSVERGTVLRDALLEAGISPYARFTKRAN
CGGRGLCATCGVRIRSSEPTADHWHDDLAARFGYPRLSCQIRVDEPMTAELVEKTVWGGR

E

>2Fe-2SST48(2730750180)Haloferax volcanii DS2, ATCC 29605(646700974)
MSDREPRVRTECDPADSTVTVRVRDTDGDRVTDLEVERGTVLRDALLDAGISPYARLTKR
VNCGGRGLCATCGVRVRSGEPTPDHWHDDLAARFGYPRLSCQLRVDGPLTVELVEKVVWG

Subtype 49

Archaea

>2Fe-2SST49(2511547885)Haloarcula hispanica CGMCC 1.2049
MPATLTVETPGGETHELTAERGAVLRDVLLDADLSPHGRYAKRVNCGGRGICATCGVRLA
EPPDPDHWHDDLADRFSYPRLSCQLRVRDGMRVELLDKRVWGSRQPDGRTD

>2Fe-2SST49(2558553106)Haloarcula hispanica N601
MPATLTVETPGGETHELTAERGAVLRDVLLDADLSPHGRYAKRVNCGGRGICATCGVRLA
EPPDPDHWHDDLADRFSYPRLSCQLRVRDGMRVELLDKRVWGSRQPDGRTDGRE

>2Fe-2SST49(645019210)Halomicrobium mukohataei arg-2, DSM 12286
MTTATITVETPDGAVREIDCEAGRVLRDVLVEHDLSPHSRLAVANCGGRGLCATCGVRLE
TPPEADHWHDRLADRFGYPRLSCQIEVVDGMAVRIPEKRVWGRRE

>2Fe-2SST49(638184149)Haloarcula marismortui ATCC 43049
MPATLTVETPAGETHELTAEPGAVLRDVLLDADLSPHGRYATRVNCGGRGICATCGVRLA
EPPDPDHWHDDLADRFGYPRLSCQLQVRDGMQVKLLDKRVWGSRQAGDETD

>2Fe-2SST49(2629121497)Haloarcula sp. CBA1115
MPATLTVETPGGETHELTAERGAVLRDVLLDADLSPHGRYAKRVNCGGRGICATCGVRLA
EPPDPDHWHDDLADRFSYPRLSCQLRVRDGMRVELLDKRVWGSRQPDGRTD
>2Fe-2SST49(644971717)Halorhabdus utahensis AX-2, DSM 12940
MSDESTAETGENDTVTVTVFDGETRTEVSVTRGRILRDGLLEQGFSPYTRLTASANCGGR
GLCATCGVRLRDGPPPKHWHDRLAARFGYPRLSCQVTVEADLTVELVADKRIWGSRNATE
NDSR

>2Fe-2SST49(2507080069)halophilic archaeon DL31
MSNLVSVTVVDGDEFATIEVERGATRRDALLEREFSVYGSVSKHANCGGRGLCATCGVAV
APEPEPTHWHDIAAARFGYPRLSCQLTVEEPMTVRLLEKRIWGQLLPNRADQ

>2Fe-2SST49(637722442)Natronomonas pharaonis Gabara, DSM 2160
MGDDIELTVITAADESTITVERGSVLRDVLLERGFSVYGTVSSRVNCGGRGLCATCTVEV
DPPPEPAHWHDAAAVRFGYPRLSCCIEVKEPMTVRLLDKHVWGQVLPRRPDGE

>2Fe-2SST49(2545640424)Halobiforma lacisalsi AJ5, JCM 12983
MAEGIPVRVITGSEGGETTIRVEPGANLRESLLEHGLPVYGTVSQYANCGGRGLCSTCTV
EVDPAPEPTHWHDAAAVRFGYPRLSCCITVEGPMTVRLLDKHVWGQVLPRQVAPDES

>2Fe-2SST49(2509883389)Natrinema pellirubrum 157
MSAAIPVTVITDESERVIEVASGTILRDALLEHGFPVYGTVSRVANCGGRGLCSTCTVEV
DPAPEPTHWHDAAAVRFGYPRLSCCLTVEEPLTVRCLDKHVWGQILPRRPSSKS

Subtype 50

Archaea

>2Fe-2SST50(651268286)Haloquadratum walsbyi C23, DSM 16854
MPTVTVGTTTIECDHGAILRDVLQRYDLGIDPHNGAASVINCRGHGTCGTCAVEIRSNNHHRKDTGTSNETITSTDTNNSVEMTADIDTSSSQNSTNNRESGDAAARINSRTAIER
VRLSVPPHTSDSELRLACQTRVLDDIRVIKHDGLWGHHTDDDQDK
>2Fe-2SST50(638051606)Haloquadratum walsbyi HBSQ001, DSM 16790
MPTVTVGTTTIECDHGAILRDVLQRYDLGIDPHNGAASVINCRGHGTCGTCAVEIRSNNHHRKDTGTSNETITSTDTNNSVEMTADIDTSSSQDSTNDRESGDAAVRINSRTAIER
VRLSVPPHTSDSELRLACQTRVLDDIRVIKHDGLWGHHTDDDQDK

Subtype 51

Archaea

>2Fe-2SST51(2684093833)Methanoculleus sp. MAB1
MPTATFLPGYRKAVVPSGTTILDAARAAGLPMNVVCGGQGKCGKCLVFIKDGTVSFDIEDCRRFFSNNEIASGACLACRATIVGDVRVEVPAESLIQEQKILVEIPGMKEVPLKPA
VWKYEVHLTPPSLDDTTPRPRPPAGGDRRSGRPAPPTVYTHRSRSSATSRRCSAGAGGT

Subtype 52

Archaea

>2Fe-2SST52(2506730837)Natronococcus occultus SP4, DSM 3396
MTDSSPLRSGRSETGSDASSCPAKTRFGLERRHVLLAAGTAGSIALSGCLDDDTDDDDEA
AATYDITFLEDGAETEVTIEETEELLYPALEADVEIPYSCEVGSCGECTARYDGDATDVV
SHDGNEYLDEDQIADGWLLTCVAYPRDDAELEVAHPDDA

Subtype 53

Eukaryota

>2Fe-2SST53(649342678)Thalassiosira pseudonana CCMP 1335
SLTFVNPDDTTTTISARVGETLLQSAHRTGIEMEGACEGVCACSTCHVILEQGLYDELLDGMEEGALGEDEEDMLDMAFGLSQTSRLGCQVKVGVNMDGSVITLPKATRNFYVDGH
KPKPH

Subtype 54

Eukaryota

>2Fe-2SST54(2752781104)Zymoseptoria tritici ST99CH_1A5
MHDLHLNGLDVRQDVATALSPASTEPGSKLTSRQTWLEKVEMRSESPLYCKVGYLTQPPS
IIAFSKAMLDIEDTGTAQVLFKTSNITATWLATEDLSLLELAEKAGLKPLHGCRSAMCGT
CEVKVLKGQVYGPEGDKPQGILICQSRPATTEIELEL
>2Fe-2SST54(2752796449)Zymoseptoria tritici ST99CH_3D7
MLDIEDTGTAQVLFKTSNITATWLATEDLSLLELAEKAGLKPLHGCRSAMCGTCEVKVLK
GQVYGPEGDKPQGILICQSRPATTEIELEL

Subtype 55

Archaea

>2Fe-2SST55(648064638)Halalkalicoccus jeotgali B3, DSM 18796
MVETYTIEFVNEGVTLEVAENESILEAAENAGLDLPYQCRMGVCGVCSGMCMEGEVDQLE
GMFLSESEKEEGYALTCIAKPRSDMRIRTNESP
>2Fe-2SST55(2718328477)Natrialbaceae archaeon JW/NM-HA 15
MVETYTVEFVDENRTIEVPEDKPILEAAEEAGLRPSYQCRMGVCGVCSAMLLEGEVDQSE
GMFLSDSEQEEGYVLTCIAKPRSDLRLRTDESP

3Fe-4S

Subtype 1

Archaea

>3Fe-4SST1(1SJ1)Pyrococcus furiosus
AWKVSVDQDTCIGDAICASLCPDVFEMNDEGKAQPKVEVIEDEELYNCAKEAMEACPVSAITIEEA

>3Fe-4SST1(2688749358)Thermococcus piezophilus CDGS
MVDQDTCIGDAICASLCPDVFEMNDEGKAVPVVEVIDDENLYNCAVEAAEACPVSAIVIE
EA

Bacteria

>3Fe-4SST1(Mmar_4763)Mycobacterium marinum

MKVIVDENICASSGNCVMNAPEIFDQRDEDGVVVLLNANPPAELAEGARRAAASCPALAIKIEE

>3Fe-4SST1(CAC17493.1)Streptomyces coelicolor A3(2)
MTLAGQAFGSGRVGAERDRCVGAGQCVLAAPGVFDQDEEDGLVRVLAERPSAAESDAVRAAVRACPSGALTLR
>3Fe-4SST1(ALO06604.1)Streptomyces venezuelae
MDVHVDRDRCLGAGMCALTAPQVFDQDEEEGLVVLLDARPPQERHAAVRVAAGVCPASVITLTRHGSSPAS

>3Fe-4SST1(ALO13336.1)Streptomyces venezuelae
MSAPLLSVDRERCIGAGMCAMTAPDVFDQDPDEGLVLLLDTEPPAAHRPAARMAAGVCPSGAITLHEPDS

>3Fe-4SST1(4ID8)Rhodopseudomonas palustris
MSEMLTIHVDQDKCQGHARCKALAPELFDLDDYGNAHEKGDGVVPADLIDKAWLAKSNCPENAIDITED
>3Fe-4SST1(mmi:MMAR_4716)Mycobacterium marinum M
MKVWVDPQRCQGHTLCAMIAPDSFQLSDIDGSSSAISETVPADQWDLVREAAHSCPEQAIVITDET

>3Fe-4SST1(mmi:MMAR_4730)Mycobacterium marinum M
MRIRLDRTVCDGFGLCAKQAPGYFTLDDWGYACLSGDGTVREEDRDAVLRALMDCPAHAITEIGERQPDVVQHSPASAEDPAEHLKTESNEAQWGFTR

Subtype 2

Bacteria

>3Fe-4SST2(FraEuI1c_5370)Frankia sp. EuI1c (Frankia inefficax sp.)

MKVFIDDDNCRGHGVCAAECPQVFVVNDDGYGEVIVDEVPAELEDAVRVVATHCPERAITVE

>3Fe-4SST2(fri:FraEuI1c_5464)Frankia sp. EuI1c (Frankia inefficax sp.)
MKAEVDAAKCQGHARCWEICPEVFALDEEGFGSVVDPEVPVELEAKAREAADNCPERAIVLA

>3Fe-4SST2(fri:FraEuI1c_2495)Frankia sp. EuI1c (Frankia inefficax sp.)
MKVAVDGAKCTGHARCQATAPEVFALDELGYAVPGEREIAAGAEAEARRGASACPERAITVWGDPLGRPAR

>3Fe-4SST2(Rv1786)Mycobacterium tuberculosis H37Rv

MKVRLDPSRCVGHAQCYAVDPDLFPIDDSGNSILAEHEVRPEDMQLTRDGVAACPEMALILEEDDAD

>3Fe-4SST2(mmi:MMAR_2667)Mycobacterium marinum M

MKIRLESSKCVGHAQCYAVDPDLFPIDESGYSILEEHEVRPEDEQVARDGVASCPEMALI

LDEE

>3Fe-4SST2(Mmar_2879)Mycobacterium marinum M

MRVAADREICMATGMCVMTADAFFDQDADGIVVLAAHEVPADEERRVRNAVKLCPSGALELMSD

>3Fe-4SST2(mmi:MMAR_4734)Mycobacterium marinum M
MRIEVDWDLCESNGICMGIVAEVFQLGDDDMLTVLQPEVTPENEELVREAVRQCPRQAISITR
>3Fe-4SST2(mmi:MMAR_4736)Mycobacterium marinum M
MKVRVDDQRCRGHGMCLTLCPEVFSLTDDGYAVAITSDVPMELEEAVREAIQCCPEQAISES

>3Fe-4SST2(CAC14338.1)Streptomyces coelicolor A3(2)
MHIGIDKDTCIGAGQCALTAPGVFTQDDDGYSTLRPGREDGGGSALVREAARACPVGAITVSERVG

>3Fe-4SST2(Rv0763c)Mycobacterium tuberculosis H37Rv

MGYRVEADRDLCQGHAMCELEAPEYFRVPKRGQVEILDPEPPEEARGVIKHAVWACPTQALSIRETGE
>3Fe-4SST2(Mmar_3973)Mycobacterium marinum M

MRVIVDETLCEANGFCESLAPDIFALGDADVVQIADGPVPADRQIDVRAAVDQCPKAALRLIE

>3Fe-4SST2(mmi:MMAR_4933)Mycobacterium marinum M
MSYRIEADLDLCQGHAMCELEAPDYFRVPKRGKVEIIDPEPPEQARPEIEQAVRMCPTQALSIKAKED
>3Fe-4SST2(mmi:MMAR_4991)Mycobacterium marinum M
MLHNDHWLAQVLGKIKEGAVRVIVDRDRCEGNAVCLGIAPDIFDLDDDDYAVVKTDPIPPDQEALAEQAIAECPRAALLRED

Subtype 7

Bacteria

>3Fe-4SST7(CAB59502.1)Streptomyces coelicolor A3(2)

MSVQQEAAVDGEALEVWIDQDLCTGDGICAQYAPEVFELDIDGLAYVKGADDELLQAPGATTPVPLTLLTDVVDSAKECPGECIHVRRVSDRAEIYGPDSE

>3Fe-4SST7(ALO07233.1)Streptomyces venezuelae
MTVQNEAPATGVEEALEVWIDQDLCTGDGICAQYAPEVFELDIDGLAYVKSADDELLQDSGATTPVPLPLLQDVVDSAKECPGDCIHVRRVADKVEVYGPDAE

Subtype 6

Bacteria

>3Fe-4SST6(fri:FraEuI1c_5333)Frankia sp. EuI1c (Frankia inefficax sp.)
MARHITIDRDLCMGSGQCLIYAPNTFDLDDDAIATVVDPDGDSDAELASAVTGCPTQAISVARD
>3Fe-4SST6(fri:FraEuI1c_1414)Frankia sp. EuI1c (Frankia inefficax sp.)
MTDAWRIEVDRRRCVGTGACAYAAPEVFRLDDRSLATVVGKVDGDDAFLRDVVAECPTEALRLSPIDDAR
>3Fe-4SST6(FraEuI1c_4132)Frankia sp. EuI1c (Frankia inefficax sp.)

MAGGAATIAVDRARCVGSGMCVVYAPDTFTHDEEAKVVVLDPLGDPAEVVESAVEACPTGALTLVTAGEAGG

>3Fe-4SST6(ALO08019.1)Streptomyces venezuelae
MGDRWHVEVDRSVCIGSGMCVNHAPTAFRLDTARQSHPTDPEADAGERLLAAAEGCPVEAILITLADGGEPVYPPEE
>3Fe-4SST6(1FXD)Desulfovibrio gigas

PIEVNDDCMACEACVEICPDVFEMNEEGDKAVVINPDSDLDCVEEAIDSCPAEAIVRS

Subtype 3

Bacteria

>3Fe-4SST3(mmi:MMAR_2932)Mycobacterium marinum M
MRLVVDLNKCQGYAQCVPLAPEVFKLVGEEALAYDPNPDDSQRQRVLRAVASCPVQAIILEVDPPADRDTK

>3Fe-4SST3(CAB92987.1)Streptomyces coelicolor A3(2)
MRISVDPEQCYGSGDCVHRAPSVFTQVGGLGAVIPGREHDVDAPRVREAAEGCPSAAITIARTEAEGVRGGGA

4Fe-4S

Subtype 7

Bacteria

>4Fe-4SST7(ABB57779.1)Synechococcus elongatus PCC 7942 = FACHB-805
MTTPDRSGLEPELGGSLRHGQARSGLEPELGGELRQKLVWVDEVTCIGCRYCSHVATNTFYIEPDYGRSRVVRQNGDPEELVQEAIDTCPVDCIHWVNPSELRQLEAERRNQVIMP
LGFPQERSKQRRRT
>4Fe-4SST7(1FXR)Desulfovibrio africanus
ARKFYVDQDECIACESCVEIAPGAFAMDPEIEKAYVKDVEGASQEEVEEAMDTCPVQCIHWEDE

Subtype 8

Bacteria

>4Fe-4SST8(1VJW)Thermotoga maritima

MKVRVDADACIGCGVCENLCPDVFQLGDDGKAKVLQPETDLPCAKDAADSCPTGAISVEE

Subtype 3

Bacteria

>4Fe-4SST3(1IQZ)Bacillus thermoproteolyticus
PKYTIVDKETCIACGACGAAAPDIYDYDEDGIAYVTLDDNQGIVEVPDILIDDMMDAFEGCPTDSIKVADEPFDGDPNKFE

Subtype 9

Archaea

>4Fe-4SST9(2Z8Q)Pyrococcus furiosus DSM 3638

AWKVSVDQDTCIGCAICASLCPDVFEMNDEGKAQPKVEVIEDEELYNCAKEAMEACPVSAITIEEA

Eukaryota

>4Fe-4SST9(2508314653)Micromonas pusilla NOUM17, RCC 299
LGPDSETKAIFVDESTCIGCRACVTWAAGTFEMVEDQNAGRARCTRQWNDDEETMQIAVEMCPVDCIYWVKRSQLAILE

Subtype 10

Archaea

>4Fe-4SST10(2505972394)Methanocella paludicola SANAE (reannotation)
MVPKVDKELCISCGNCVDLCPDVFVWDDEGKAEVTNPGGCSTQCNCQEAAESCPTDAISL
EE

Subtype 11

Archaea

>4Fe-4SST11(2511505707)Thermococcus sp. AM4
MAWKVTVDVDTCIGDAICASLCPDVFEMGDDGKAHPIVDVTDLECAQEAAEACPVGAIIL
EEA
>4Fe-4SST11(2636844530)Thermococcus eurythermalis A501
MAWKVTVDQDTCIGDAICASLCPDVFEMNDEGKAVPIVETTDLECAKEAAEACPVGAITL
EEV

>4Fe-4SST11(2682416982)Thermococcus guaymasensis DSM 11113
MAWKVTVDQDTCIGDAICASLCPDVFEMGDDGKAHAIVETTDLECAKEAAEACPVGAITL
EEV
>4Fe-4SST11(2687701486)Thermococcus peptonophilus OG-1
MAWKVTVDQDTCIGDAICASLCPDVFEMGDDGKAHPIVETTDLECAQEAAEACPVGAITL
EEV
>4Fe-4SST11(2758666062)Thermococcus gorgonarius W-12
MAWKVTVDQDTCIGDAICASLCPDVFEMGDDGKAHPVVETTDLECAQEAAEACPVGAITL
EEV
>4Fe-4SST11(638156651)Archaeoglobus fulgidus VC-16, DSM 4304
MKAVVDRDLCIGCGTCEEICPEVFRLNDEGISEVIGSCDSAECCEEAMESCPASAISLED
>4Fe-4SST11(644842173)Thermococcus sibiricus MM 739

MKVSVDKDTCIGCGVCASICPDVFEMDDEGKAQALVTETDLECAKEAAESCPTGSITVE
>4Fe-4SST11(2685063455)Thermococcus sp. 2319x1
MKVSVDKDTCIGCGVCASICPDVFEIDDDGKAKAIVPETDLECAKEAAESCPTGAITVE
>4Fe-4SST11(2514742512)Thermococcus litoralis DSM 5473
MKVSVDKDACIGCGVCASICPDVFEMDDDGKAKALVAETDLECAKEAAESCPTGAITVE
>4Fe-4SST11(2559338294)Thermococcus paralvinellae ES1
MKVKVDKDTCIGCGVCASICPDVFEMDDDGKAKTLVEETDLECAKEAAESCPTGAIIIEE
>4Fe-4SST11(650727821)Thermococcus barophilus MP, DSM 11836
MKVKLDKDTCIGCGVCASICPDVFEMDDDGKAKVIMEETDLECAKEAAESCPTGSITIEE
>4Fe-4SST11(2684671857)Thermococcus barophilus CH5
MKVKLDKDTCIGCGVCASICPDVFEMDDDGKAKVIMEETDLECAKEAAESCPTGSITIEE

Subtype 12

Archaea

>4Fe-4SST12(2635974192)Geoglobus acetivorans SBH6
MMKVVIDESTCTGCGTCESICPEVFQLGDDGLAHVVGECEGLEECCQEAADNCPVEAITI
E
>4Fe-4SST12(646611275)Ferroglobus placidus AEDII12DO, DSM 10642
MPAKVDENLCTGCGLCEEICPEVFKLDENGISRVVGDCEANIDCCQEAAESCPAGAITIE

7Fe-8S

Subtype 1

Bacteria

>7Fe-8SST1(2V2K)MYCOBACTERIUM SMEGMATIS
TYVIAEPCVDVKDKACIEECPVDCIYEGARMLYIHPDECVDCGACEPVCPVEAIYYEDDVPDQWSSYAQANADFFAELGSPGGASKVGQTDNDPQAIKDLPPQGE

>7Fe-8SST1(Rv1177)Mycobacterium tuberculosis strain ATCC 25618 / H37Rv
MTYTIAEPCVDIKDKACIEECPVDCIYEGARMLYIHPDECVDCGACEPVCPVEAIFYEDDVPEQWSHYTQINADFFAELGSPGGAAKVGMTENDPQAVKDLAPQSEDA

>7Fe-8SST1(1BD6)Bacillus schlegelii

AYVITEPCIGTKDASCVEVCPVDCIHEGEDQYYIDPDVCIDCGACEAVCPVSAIYHEDFVPEEWKSYIQKNRDFFKK

>7Fe-8SST1(CCH24277.1)Corynebacterium glutamicum K051
MTYTIAQPCVDVLDRACVEECPVDCIYEGKRMLYIHPDECVDCGACEPACPVEAIFYEDDVPDEWLDYNDANAAFFDDLGSPGGAAKLGPQDFDHPMIAALPPQA

>7Fe-8SST1(CCH26076.1)Corynebacterium glutamicum K051
MTYTIAQPCVDVLDRACVEECPVDCIYEGKRMLYIHPDECVDCGACEPVCPVEAIFYEDDVPHEWWDYTGANAAFFDDLGSPGGAASLGPQDFDAQLVAVLPPQNQN
>7Fe-8SST1(CAC04216.1)Streptomyces coelicolor A3(2)
MTYVIAQPCVDIKDRACVTECPVDCIYEGARTLYINPAECVDCHACEPVCPVEAIFHEDDLPRHWAHYLAVNAEYFDEAASPSRARRDAVGDHPAVEALPPQSGPHKKEISFFTVR
KEEAADADLWFPS
>7Fe-8SST1(CAC05765.1)Streptomyces coelicolor A3(2)
MTYVIAQPCVDVKDKACIEECPVDCIYEGQRSLYIHPDECVDCGACEPVCPVEAIFYEDDTPEEWKDYYKANVEFFDELGSPGGASKLGLIERDHPFVAALPPQNQ
>7Fe-8SST1(ALO10852.1)Streptomyces venezuelae
MTYVIAQPCVDVKDKACIEECPVDCIYEGKRSLYIHPDECVDCGACEPVCPVEAIFYEDDTPEEWKDYYKANVEFFDELGSPGGASKLGEIERDHPFIAALPPQNG

>7Fe-8SST1(azo:azo1031)Azoarcus olearius BH72

MAYVVTESCIRCKYTDCVDVCPVDCFREGENFLVIDPEECIDCTLCVAECPVEAIYAEDDVPADQQQFIALNAELARTWKPIVERKEPLPDAEQWAKVKGKTGELKR

>7Fe-8SST1(WP_063541473.1)Pseudomonas stutzeri
MTFVVTDNCIKCKYTDCVEVCPVDCFYEGPNFLVIHPDECIDCALCEPECPAQAIFSEDEVPEDQQEFIE
LNTDLAEVWPNITEKKDALADAEEWDGVKDKLQYLER

>7Fe-8SST1(P0CY91)Rhodobacter capsulatus OX=1061

MTYVVTDNCIACKYTDCVEVCPVDCFYEGENTLVIHPDECIDCGVCEPECPADAIRPDTEPGMEDWVEFNRTYASQWPVITIKKDPMPDHKKYDGETGKREKYFSPNPGTGD

>7Fe-8SST1(7FDR)Azotobacter vinelandii
AFVVTDNCIKCKYTDCVEVCPVDCFYEGPNFLVIHPDECIDCALCEPECPAQAIFSEDEVPEDMQEFIQLNAELAEVWPNITEKKDPLPDAEDWDGVKGKLQHLER

>7Fe-8SST1(Rv2007c)Mycobacterium tuberculosis H37Rv
MTYVIGSECVDVMDKSCVQECPVDCIYEGARMLYINPDECVDCGACKPACRVEAIYWEGDLPDDQHQHLGDNAAFFHQVLPGRVAPLGSPGGAAAVGPIGVDTPLVAAIPVECP

Subtype 5

Archaea

>7Fe-8SST5(2VKR)Acidianus Ambivalens
GIDPNYRTSRQVVGEHQGHKVYGPVDPPKVLGIHGTIVGVDFDLCIADGSCITACPVNVFQWYDTPGHPASEKKADPINEQACIFCMACVNVCPVAAIDVKPP

Subtype 6

Bacteria

>7Fe-8SST6(ABB56846.1)Synechococcus elongatus PCC 7942 = FACHB-805
MAHTIVTNTCEGVADCVDACPVACIQEGPGRNQKGTTWYWIDFSTCIDCGICLQVCPVEGAILPEERPEL
QQTP

Eukaryota

>7Fe-8SST6(2505188782)Paulinella chromatophora
MAHTIVTDICEGIADCVNACPVACIHMGNGINKKGTNFYWIDFNTCIDCGICLQVCPLEN
AILAEERSELQQIN

Subtype 7

Bacteria

>7Fe-8SST7(1H98)Thermus Aquaticus
PHVICEPCIGVKDQSCVEVCPVECIYDGGDQFYIHPEECIDCGACVPACPVNAIYPEEDVPEQWKSYIEKNRKLAGLE

Subtype 8
Archaea

>7Fe-8SST8(2509663901)Methanomethylovorans hollandica DSM 15978
MPAKVDPNICEGIGACKESCPEDVFEMKDDGQGLKSVVVHPDNCIECGLCVDACPMGAIT
IE

>7Fe-8SST8(2633313307)Methanosarcina lacustris Z-7289
MYPKIDYDKCVGSLECYDVCPADVYDEEETKDGKRAVVARPDECTECEQCIDACPTDAIE
LVE

>7Fe-8SST8(2629258817)Methanosarcina sp. WWM596
MHPKIDYDKCVGSLECYDVCPADVYDKEETKDGKRAIVARPDECTECEQCIDACPTDAIE
MVD
>7Fe-8SST8(2628411983)Methanosarcina sp. WH1
MHPKIDYDKCVGSLECYDVCPADVYDKEETKDGKRAIVARPDECTECEQCIDACPTDAIE
MVD
>7Fe-8SST8(2653181245)Methanosarcina sp. MTP4
MYPKIDYDKCVGSLECYDVCPTDVYDVKETEDGKRAVVARPDDCTECEQCIDVCPTDAIE
LVD
>7Fe-8SST8(2633313308)Methanosarcina lacustris Z-7289
MHPVIDYHKCTGALACYEVCPGDVFDIKEMEAGKKAVVARPDDCIECDQCVDACPEDAIE
LVD
>7Fe-8SST8(2629258818)Methanosarcina sp. WWM596
MHPVIDYHKCTGALACYEVCPGDVFDVKEMEAGKKAVVARPDDCIECDQCVDACPEDAIE
LVD
>7Fe-8SST8(2628411984)Methanosarcina sp. WH1
MHPVIDYHKCTGALACYEVCPGDVFDVKEMEAGKKAVVARPDDCIECDQCVDACPEDAIE
LVD
>7Fe-8SST8(2509663901)Methanomethylovorans hollandica DSM 15978
MPAKVDPNICEGIGACKESCPEDVFEMKDDGQGLKSVVVHPDNCIECGLCVDACPMGAIT
IE

Subtype 9

Archaea

>7Fe-8SST9(2502871345)Methanosalsum zhilinae WeN5, DSM 4017
MVAIIDPDKCNGYTLCADACPVDAITIREDDIAIVDPDICTDCGECVDACPVGAITIK

Subtype 10

Archaea

>7Fe-8SST10(638164976)Methanosarcina mazei Go1
MHCSLECYDTCPVDVFDAEETEEGKRAVVARPEDCIECEQCVEVCPTDAIELVED
>7Fe-8SST10(2629268101)Methanosarcina mazei S-6
MHCSLECYDTCPVDVFDAEETEEGKRAVVARPEDCIECEQCVEVCPTDAIELVED

2[4Fe-4S]

Subtype 2

Bacteria

>2[4Fe-4S]ST2(3EUN)Allochromatium vinosum
ALMITDECINCDVCEPECPNGAISQGDETYVIEPSLCTECVGHYETSQCVEVCPVDAIIKDPSHEETEDELRAKYERITG
EG

>2[4Fe-4S]ST2(2ZVS)Escherichia coli K-12
ALLITKKCINCDMCEPECPNEAISMGDHIYEINSDKCTECVGHYETPTCQKVCPIPNTIVKDPAHVETEEQLWDKFVLMH
HADKI

>2[4Fe-4S]ST2(P0CY90)Rhodobacter capsulatus
MAMKIDPELCTSCGDCEPVCPTNAIAPKKGVYVINADTCTECEGEHDLPQCVNACMTDNCINPAA

>2[4Fe-4S]ST2(A9FH21)Sorangium cellulosum So ce56
MATYITEDCINCGACEPECPNEAISEGDEIYVIDPELCTECVGFYDHEACQAVCPVECCLPNPQIVETEEVLIARAVRLHPDDGELKKRAAANDYPSRFRK

Subtype 3

Bacteria

>2[4Fe-4S]ST3(A9GFT9)Sorangium cellulosum So ce56
MIELVSDARCIGCDLCVEVCPTDVFDRVPGGAPVIARQGDCQTCFMCEAYCPVDALYVAP
EAERPVAVDAERLEASGLLGSYRAAVGWGTRRKPGAAADETFRVLTVARQT

Archaea

>2[4Fe-4S]ST3(638175208)Methanosarcina acetivorans C2A
MPAKVNKEECTACGTCVEECPVEAIVIDEDAGCAVVDEDECVDCGACEEACPVGAIKTE
>2[4Fe-4S]ST3(638166185)Methanosarcina mazei Go1
MVAKVNKEECTGCGTCVDECPVEAIIIDEDEGCAVVDEDECVDCGACEDVCPIGAIKVE
>2[4Fe-4S]ST3(2637280920)Methanosarcina thermophila TM-1
MPAKVNKEECTGCGTCVEECPVEAIVIDEDEGCAAVDEEECVECGACEEVCPIEAIEVE
>2[4Fe-4S]ST3(649737917)Methanothermus fervidus V24S, DSM 2088
MKIIVNDKCNSCGVCRLVCPKGGKIWKINKKAKATNLEYCHLCMICATRCPTQAIKIIR
>2[4Fe-4S]ST3(2649849577)Methanosarcina barkeri 3
MPATVNADECSGCGSCVDECPNDAIALDEEKGIAVVDNDECVECGACEEACPNQAIKVEE

>2[4Fe-4S]ST3(2632753581)Methanosarcina barkeri Wiesmoor
MPAIVNADECSGCGTCVDECPNDAITLDEEKGIAVVDNDECVECGACEEACPNQAIKVEE
>2[4Fe-4S]ST3(637699650)Methanosarcina barkeri Fusaro, DSM 804
MPAIVNADECSGCGTCVDECPNDAITLDEEKGIAVVDNDECVECGACEEACPNQAIKVEE
>2[4Fe-4S]ST3(2632830895)Methanosarcina barkeri 227
MPATVNADECSGCGTCVDECPNDAITLDEEKGIAVVDNDECVECGACEEACPNQAIKVEE
>2[4Fe-4S]ST3(2635680194)Methanosarcina barkeri CM1
MPATVNADECSGCGTCVDECPNDAITLDEEKGIAVVDNDECVECGACEEACPNQAIKVEE

>2[4Fe-4S]ST3(2648886128)Methanosarcina vacuolata Z-761
MPATVNADECSGCGTCVDECPNDAITLDEEKGIAVVDNDECVECGACEEACPNQAIKVEE
>2[4Fe-4S]ST3(2633008971)Methanosarcina barkeri MS
MPATVNADECSGCGTCVDECPNDAITLDEEKGIAVVDNDECVECGACEEACPNQAIKVEE
>2[4Fe-4S]ST3(2628281869)Methanosarcina sp. Kolksee
MPATVNADECSGCGTCVDECPNDAITLDEEKGIAIVDNDECVECGACEEACPNQAIKVEE
>2[4Fe-4S]ST3(2653181867)Methanosarcina sp. MTP4
MPAIVNEEECSGCGTCVDECPSEAITLDDDKGIAVVDQDECVECGACEEACPNDAIKVEE
>2[4Fe-4S]ST3(2633310938)Methanosarcina lacustris Z-7289
MPAIVNADECSGCGTCVDECPSEAITLDEEKGVAVVDQDECVECGACEEACPNQAIKVQE
>2[4Fe-4S]ST3(2630864270)Methanosarcina horonobensis HB-1
MPALVNADECSGCGTCVDECPSEAITLDEEKGCAVVDQDECVECGACEEACPNQAIKVQE
>2[4Fe-4S]ST3(2630385317)Methanosarcina thermophila CHTI-55
MPALVNADECSGCGSCVDECPSEAITLDEEKGIAVVDQDECVECGACEEACPNQAIKVEE

>2[4Fe-4S]ST3(2637280903)Methanosarcina thermophila TM-1
MPALVNADECSGCGSCVDECPSEAITLDEEKGIAVVDQDECVECGACEEACPNQAIKVEE

>2[4Fe-4S]ST3(2683957013)Methanosarcina sp. 795 OTU795
MPALVNADECSGCGSCVDECPSEAITLDEEKGIAVVDQDECVECGACEEACPNQAIKVEE
>2[4Fe-4S]ST3(2641569127)Methanosarcina mazei WWM610
MPAIVNADECSGCGTCVDECPSEAITLDEEKGIAVVDQDECVECGACEEACPNQAIKVTE
>2[4Fe-4S]ST3(638166151)Methanosarcina mazei Go1
MPAIVNADECSGCGTCVDECPSEAITLDEEKGIAVVDQDECVECGACEEACPNQAIKVTE
>2[4Fe-4S]ST3(2629270533)Methanosarcina mazei S-6
MPALVNADECSGCGTCVDECPSEAITLDEEKGIAVVDQDECVECGACEEACPNQAIKVTE
>2[4Fe-4S]ST3(2633686767)Methanosarcina mazei LYC
MPALVNADECSGCGTCVDECPSEAITLDEEKGIAVVDQDECVECGACEEACPNQAIKVTE
>2[4Fe-4S]ST3(2540563552)Methanosarcina mazei Tuc01
MPALVNADECSGCGTCVDECPSEAITLDEEKGIAVVDQDECVECGACEEACPNQAIKVTE
>2[4Fe-4S]ST3(2651207974)Methanosarcina mazei SarPi
MPALVNADECSGCGTCVDECPSEAITLDEEKGIAVVDQDECVECGACEEACPNQAIKVTE
>2[4Fe-4S]ST3(2638501146)Methanosarcina mazei C16
MPALVNADECSGCGTCVDECPSEAITLDEEKGIAVVDQDECVECGACEEACPNQAIKVTE
>2[4Fe-4S]ST3(2645882981)Methanosarcina siciliae HI350
MPAIVNADECSGCGTCVDECPSEAITLDEEKGIAVVDQDECVECGACEEACPNEAIKVEE
>2[4Fe-4S]ST3(2653161569)Methanosarcina siciliae T4/M
MPAIVNADECSGCGTCVDECPSEAITLDEEKGIAVVDQDECVECGACEEACPNEAIKVEE
>2[4Fe-4S]ST3(2628823615)Methanosarcina siciliae C2J

MPAIVNADECSGCGTCVDECPSEAITLDEEKGIAVVDQDECVECGACEEACPNEAIKVEE
>2[4Fe-4S]ST3(638175176)Methanosarcina acetivorans C2A
MPAIVNADECSGCGTCVDECPSEAITLDEEKGLAVVDQDECVECGACEEACPNQAIKVEE
>2[4Fe-4S]ST3(2628409721)Methanosarcina sp. WH1
MPAIVNADECSGCGTCVDECPSEAITLDEEKGIAVVDQDECVECGACEEACPNQAIKVEE
>2[4Fe-4S]ST3(2629256159)Methanosarcina sp. WWM596
MPAIVNADECSGCGTCVDECPSEAITLDEEKGIAVVDQDECVECGACEEACPNQAIKVEE
>2[4Fe-4S]ST3(2653181688)Methanosarcina sp. MTP4
MPAKVNKEECTGCRTCVEECPVEAITLDEEEGVAVVDEEECVECGACEETCTSEAIKVE
>2[4Fe-4S]ST3(2628409753)Methanosarcina sp. WH1
MPAKVNKEKCTGCGTCVDECPSEAITLDEKEGIAVVNEDECVDCGACEDVCPAEAIKVE
>2[4Fe-4S]ST3(2629256189)Methanosarcina sp. WWM596
MPAKVNKEKCTGCGTCVDECPSEAITLDEKEGIAVVNEDECVDCGACEDVCPAEAIKVE
>2[4Fe-4S]ST3(2630385334)Methanosarcina thermophila CHTI-55
MPAKVNKEECTGCGTCVEECPVEAIVIDEDEGCAAVDEEECVECGACEEVCPIEAIEVE
>2[4Fe-4S]ST3(2637280920)Methanosarcina thermophila TM-1
MPAKVNKEECTGCGTCVEECPVEAIVIDEDEGCAAVDEEECVECGACEEVCPIEAIEVE
>2[4Fe-4S]ST3(2649849596)Methanosarcina barkeri 3
MPAKVKKEECTGCGTCVEECPVEAIIIDEDEGCAVVDEEECVDCGACEEVCPIQAIEVK
>2[4Fe-4S]ST3(2683957030)Methanosarcina sp. 795 OTU795
MPAKVNKEECTGCGTCVEECPVEAIVIDEDEGCAAVDEEECVECGACEEVCPIEAIEVE
>2[4Fe-4S]ST3(2628281904)Methanosarcina sp. Kolksee
MPAKVNKEECTGCGTCVDECPVEAIVIDEDEGCAVVDEDECVECGACEEACPNEAITIEQ
PE
>2[4Fe-4S]ST3(2648886164)Methanosarcina vacuolata Z-761
MPAKVNKEECTGCGTCVDECPVEAIVIDEDEGCAVVDEDECVECGACEEACPNEAITIEQ
PE
>2[4Fe-4S]ST3(2632753604)Methanosarcina barkeri Wiesmoor
MPAKVKKEECTGCGTCVDECPVEAIVIDEEEGCAVVDEDECVECGACEEACPNEAITIE
>2[4Fe-4S]ST3(637699628)Methanosarcina barkeri Fusaro, DSM 804
MPAKVKKEECTGCGTCVDECPVEAIVIDEEEGCAVVDEDECVECGACEEACPNEAITIE
>2[4Fe-4S]ST3(2632830873)Methanosarcina barkeri 227
MPAKVNKEECTGCGTCVDECPVEAIVIDEEEGCAVVDEDECVECGACEEACPNGAITSE
>2[4Fe-4S]ST3(2635680168)Methanosarcina barkeri CM1
MPAKVNKEECTGCGTCVDECPVEAIVIDEEEGCAVVDEDECVECGACEEACPNGAITSE
>2[4Fe-4S]ST3(2633008995)Methanosarcina barkeri MS
MPAKVNKEECTGCGTCVDECPVEAIVIDEEEGCAVVDEDECVECGACEEACPNGAITSE
>2[4Fe-4S]ST3(638166185)Methanosarcina mazei Go1
MVAKVNKEECTGCGTCVDECPVEAIIIDEDEGCAVVDEDECVDCGACEDVCPIGAIKVE

>2[4Fe-4S]ST3(2651207934)Methanosarcina mazei SarPi
MVAKVNKEECTGCGTCVDECPVEAIIIDEDEGCAVVDEDECVDCGACEDVCPIGAIKVE
>2[4Fe-4S]ST3(638175208)Methanosarcina acetivorans C2A
MPAKVNKEECTACGTCVEECPVEAIVIDEDAGCAVVDEDECVDCGACEEACPVGAIKTE
>2[4Fe-4S]ST3(2645883005)Methanosarcina siciliae HI350
MPAKVNKEECTACGTCVEECPVEAIIIDEDAGCAVVDEDECVDCGACEEACPVGAIKVE
>2[4Fe-4S]ST3(2653161596)Methanosarcina siciliae T4/M
MPAKVNKEECTACGTCVEECPVEAIIIDEDAGCAVVDEDECVDCGACEEACPVGAIKVE

>2[4Fe-4S]ST3(2628823643)Methanosarcina siciliae C2J
MPAKVNKEECTACGTCVEECPVEAIIIDEDAGCAVVDEDECVDCGACEEACPVGAIKVE
>2[4Fe-4S]ST3(2630864246)Methanosarcina horonobensis HB-1
MAAKVNKEECTGCGTCVEECPVEAIIIDEDEGCAVVDEDECVDCGACEEACPIGAIKTE

Subtype 8

Archaea

>2[4Fe-4S]ST8(650943090)Pyrococcus yayanosii CH1
MTEVKVAPEEKVRKKPSFVKPWLGLKYLFKRPVTIKIPDEKIDPAPKYRGFHTLDWKKCV
GCNFCGQICPARAIEMTWIEGEKRPHPKIDYGRCTFCQFCVDVCPTGALSFIESYILTTT
WREEELELYDWVPIPPDKFREMQEKFGDYRFPLEKMEFNRETKEVTYYLRDGEVMKFKIL
GYGIKPPKSPAEKAYEK

>2[4Fe-4S]ST8(639772936)Thermofilum pendens Hrk 5
MGIRQILKIALREGVATLEYPFKPEEAPEDFRGKPEIDPSICMGCGACANVCPPDAITCV
DDLERGLRTWKIFYGRCIFCGRCEEACPLSAIRQSKEYELASKTREDLEVVVETPLARCS
TCGKYFPVTEREVAQAAQILAEAKTLPRSVLELLENGVECPECKRRRTVLSIVKSARPGV
KPLSVTGEEGGRHE

Subtype 9

Archaea

>2[4Fe-4S]ST9(2632831998)Methanosarcina barkeri 227
MDQFFSSLLKKSIIPLENPPKWAKLFPYAFMLFLILISAVFLIPIFCAYICPLRIIYDPP
AVTTTFQWITALIFVTGGLIFLVIGPLLTKKRLFCSFICPLMPANTLIGVINPFKVKINI
DLCKKCGLCVKSCELFAITKESLAKGKTIIECAECGKCMDKCPAGAIDYKLMFTDQNIRF
FYSAGNYL
>2[4Fe-4S]ST9(2638498581)Methanosarcina mazei C16
MSEMNGYVVVFGCKRCGKCRDVCPVGAIYEENELAKIDTEKCNLCMKCIDECTNRSIIYM
E
>2[4Fe-4S]ST9(638165095)Methanosarcina mazei Go1
VVFGCKRCGKCRDVCPVGAIYEENELAKIDTEKCNLCMKCIDECTNRSIIYME

>2[4Fe-4S]ST9(2641566654)Methanosarcina mazei WWM610
MSEMNGYVVVFGCKRCGKCRDVCPVGAIYEENELAKIDTEKCNLCMKCIDECTNRSIIYM
E
>2[4Fe-4S]ST9(2630861022)Methanosarcina horonobensis HB-1
MSDTNGYVVVFGCKRCGKCRDVCPVGAIYEENELAKIDPEKCNLCMKCIDECTNRSIIYM
D
>2[4Fe-4S]ST9(2683956731)Methanosarcina sp. 795 OTU795
MSEKTGYVVVFGCKRCGKCKNICPVDAIYEENELSKIDTEKCTLCMKCIDECTNRAIIYM
E

>2[4Fe-4S]ST9(2637280624)Methanosarcina thermophila TM-1
MSEKTGYVVVFGCKRCGKCKNICPVDAIYEENELSKIDTEKCTLCMKCIDECTNRAIIYM
E

>2[4Fe-4S]ST9(2630385038)Methanosarcina thermophila CHTI-55
MSEKTGYVVVFGCKRCGKCKNICPVDAIYEENELSKIDTEKCTLCMKCIDECTNRAIIYM
E

>2[4Fe-4S]ST9(637699166)Methanosarcina barkeri Fusaro, DSM 804
MSEQNGYVVVFGCKRCGKCKDVCPVDAIYEENELSKIDQDKCTRCMKCIDVCTNKAIIYM
>2[4Fe-4S]ST9(2628282245)Methanosarcina sp. Kolksee
MSEQNGYVVVFGCKRCGKCKDVCPVDAIYEENEISKIDQDKCTRCMKCIDVCTNKAIIYM
E
>2[4Fe-4S]ST9(2648886514)Methanosarcina vacuolata Z-761
MSEQNGYVVVFGCKRCGKCKDVCPVDAIYEENEISKIDQDKCTRCMKCIDVCTNKAIIYM
E
>2[4Fe-4S]ST9(2632831489)Methanosarcina barkeri 227
MSEQNGYVVVFGCKRCGKCKDVCPVDAIYEENELSKIDQDKCTRCMKCIDVCPNKAIIYM

E
>2[4Fe-4S]ST9(2635679565)Methanosarcina barkeri CM1
MSEQNGYVVVFGCKRCGKCKDVCPVDAIYEENELSKIDQDKCTRCMKCIDVCPNKAIIYM
E
>2[4Fe-4S]ST9(2633011636)Methanosarcina barkeri MS
MSEQNGYVVVFGCKRCGKCKDVCPVDAIYEENELSKIDQDKCTRCMKCIDVCPNKAIIYM
E
>2[4Fe-4S]ST9(2632754104)Methanosarcina barkeri Wiesmoor
MSEQNGYVVVFGCKRCGKCKDVCPVDAIYEENELSKIDQDKCTRCMKCIDVCTNKAIIYM
E
>2[4Fe-4S]ST9(2649850361)Methanosarcina barkeri 3
MSEQNGYVVVFGCKRCGKCKDVCPVGAIYEENELAKIDPKKCTLCMKCIDECTNRSIIYM
E
>2[4Fe-4S]ST9(2628412039)Methanosarcina sp. WH1
MSEKEGYVVVFGCKRCGKCKDVCPVGAIYEENELAKIDPEKCNLCMKCIDECTNRSIIYM
E
>2[4Fe-4S]ST9(2629258757)Methanosarcina sp. WWM596
MSEKEGYVVVFGCKRCGKCKDVCPVGAIYEENELAKIDPEKCNLCMKCIDECTNRSIIYM
E

>2[4Fe-4S]ST9(2629267979)Methanosarcina mazei S-6
MSEMNGYVVVFGCKRCGKCRDVCPVGAIYEENELAKIDTEKCNLCMKCIDECTNRSIIYM
E
>2[4Fe-4S]ST9(2633684230)Methanosarcina mazei LYC
MSEMNGYVVVFGCKRCGKCRDVCPVGAIYEENELAKIDTEKCNLCMKCIDECTNRSIIYM
E
>2[4Fe-4S]ST9(2651205513)Methanosarcina mazei SarPi
MSEMNGYVVVFGCKRCGKCRDVCPVGAIYEENELAKIDTEKCNLCMKCIDECTNRSIIYM
E
>2[4Fe-4S]ST9(2685268711)Methanobacterium formicicum Mb91
MIVREWCMYCGECAGVCPLNIIQVRELTLEFDEEKCNNCKICVKACPVNALEEEVV
>2[4Fe-4S]ST9(2647505556)Methanobacterium formicicum DSM 1535
MIVREWCMYCGECAGVCPLNIIQVRELTLEFDEEKCNNCKICVKACPVNALEEEVV

>2[4Fe-4S]ST9(2637993349)Methanobacterium sp. MB1
MIVREWCMYCGECAGVCPLNIIQIRELTLEFDEKKCNNCKICVKACPVNALEEEVV

>2[4Fe-4S]ST9(2637992165)Methanobacterium sp. MB1
MIVNEWCMYCGECAGVCPRNLIEVREISLKINQEGCKDCGICIQVCPVQALQGDD

>2[4Fe-4S]ST9(2647504296)Methanobacterium formicicum DSM 1535
MIVNEWCMYCGECAGVCPQNLIEVREISLKINHDECKDCGICIKVCPVQALQGDD
>2[4Fe-4S]ST9(2631418098)Methanobacterium formicicum BRM9
MIVNEWCMYCGECAGVCPQNLIEVREISLKINHDECKDCGICIKVCPVQALQGDD

>2[4Fe-4S]ST9(2685267232)Methanobacterium formicicum Mb91
MIVNEWCMYCGECAGVCPQNLIEVRELSLKINHDECKDCGICIKVCPVQALQGDD
>2[4Fe-4S]ST9(650870970)Methanobacterium paludis SWAN-1
MIVKEWCMYCGECAGVCPRTLIEVQETNLIFNKEGCKDCQICIQVCPVQALVKEE
>2[4Fe-4S]ST9(650752141)Methanobacterium lacus AL-21
MIVKEWCMYCGECAGVCPRALIEVRETSLIFNEEGCKDCRICVSVCPINALDKED
>2[4Fe-4S]ST9(638154785)Methanothermobacter thermautotrophicus Delta H
LITMKVKEWCMFCGECAGVCPRNLIEVRETSLKFREDECRECNICIQVCPVRALER
>2[4Fe-4S]ST9(2540853929)Methanobrevibacter sp. AbM4
MIVKDWCVYCGECAGVCPRNLITVRETNLEFKTDECKECSTCVDACPINALEQE
>2[4Fe-4S]ST9(2655683698)Methanobrevibacter millerae SM9
MIVKEWCSFCGECAGVCPRNLIQVREYSLVFKEDECRDCDTCIKACPINALEKED
>2[4Fe-4S]ST9(640593524)Methanobrevibacter smithii PS, ATCC 35061
MIVKDWCSFCGECAGVCPRNLIQVKEYSLVFDESECRECSTCVDACPINALEKED
>2[4Fe-4S]ST9(2522261378)Archaeoglobus sulfaticallidus PM70-1, DSM 19444 (Asulf_version1)
MLVIDRYKCAYCGACISVCKFNANELVETFVRVDEEKCTLCKMCVRVCPMGAISVEE
>2[4Fe-4S]ST9(638156977)Archaeoglobus fulgidus VC-16, DSM 4304
MARKAIVDRRKCAYCGACVAVCPADANELVETFLEIYDDRCTGCGICVKMCPMGALAMVE
VD
>2[4Fe-4S]ST9(2635973664)Geoglobus acetivorans SBH6
MKKLIVNRYRCGYCGACVAVCKFNANELVETYLEIYEDKCTLCQACVRTCPMNALEVVE
>2[4Fe-4S]ST9(646611498)Ferroglobus placidus AEDII12DO, DSM 10642
MKRIVVNRYKCAYCGACVSVCKFDANELVETYLEIYPDKCTLCMVCVKTCPMGALEVVE
>2[4Fe-4S]ST9(646366977)Methanocaldococcus vulcanius M7, DSM 12094
MVKVNYKRCGYCGACVGVCENLAINLIEHIVVIDEKKCSNCNLCIIVCPLNALEGE
>2[4Fe-4S]ST9(646622816)Methanocaldococcus sp. FS406-22
MVKINYKKCGYCGACVGVCEKLAINLIEHIIVIDEEKCSNCKLCTIVCPLNALEGE
>2[4Fe-4S]ST9(638201852)Methanocaldococcus jannaschii DSM 2661
MVKIDYKKCGYCGACVGVCEKLAINLIEHIIVIDEKKCNNCKLCTIVCPLNALEGE
>2[4Fe-4S]ST9(644969457)Methanocaldococcus fervens AG86
MVKINYKKCGYCGACVGVCENLAINLIEHIIVIDEKKCNNCKLCTIVCPLNALEGE
>2[4Fe-4S]ST9(646860138)Methanococcus voltae A3
MKVNTLKCGYCGACVGICPRNAIELIENKIEIDTEKCKGCKLCKEICPVNALEE
>2[4Fe-4S]ST9(640788721)Methanococcus aeolicus Nankai-3

MEVNIEKCGYCGACVGVCNRLAIELIENTVIIDNEKCNSCGLCAIVCPLNALNTLEE
>2[4Fe-4S]ST9(650855714)Methanotorris igneus Kol5, DSM 5666
MKVNYERCGYCGACVGVCDKMAIELVENLIIINNEKCTNCKLCMIVCPLNALVDG
>2[4Fe-4S]ST9(650917389)Methanothermococcus okinawensis IH1
MKVNHEKCGYCGACVGVCKELAIELVENMIVIDNKKCNNCKLCMIVCPLNALEE
>2[4Fe-4S]ST9(640793945)Methanococcus maripaludis C7
MKVNYSKCGYCGACVGVCKSMAIELIENKLYIDDKKCNKCTLCMIVCPLNALEE
>2[4Fe-4S]ST9(640787073)Methanococcus vannielii SB
MNVDYNKCGYCGACVGVCKSMAIELIENKIIIYDEKCNNCKLCAVVCPLNALEE
>2[4Fe-4S]ST9(2563556328)Methanococcus maripaludis S2
MKVNYSKCGYCGACVGVCKSMAIELIENKLYIEEEKCNNCKLCTVVCPVNALEE
>2[4Fe-4S]ST9(2511671152)Methanococcus maripaludis X1
MKVNYSKCGYCGACVGVCKSMAIELIENKLYIEEEKCNNCKLCTVVCPVNALEE
>2[4Fe-4S]ST9(641282982)Methanococcus maripaludis C6
MKVNYNKCGYCGACVGVCKSMAIELIENKLYIEEEKCNNCKLCAVVCPVNALEE
>2[4Fe-4S]ST9(640166740)Methanococcus maripaludis C5
MKVNYNKCGYCGACVGVCNSMAIELIENKLYIEEEKCNNCKLCAVVCPVNALEE
>2[4Fe-4S]ST9(646648629)Aciduliprofundum boonei T469
MNMMNVDPAICNYCGACVGSCPVNCMFLDETIVRIDEDKCIKCGFCIRACPVGAISADWW
SE
>2[4Fe-4S]ST9(2509054261)Aciduliprofundum sp. MAR08-339
MMKVDPSVCNYCGACVGSCPVNCMFLDETIVRIDEDKCIKCGFCIRACPVGAIDAEWWSE
>2[4Fe-4S]ST9(2632754767)Methanosarcina barkeri Wiesmoor
VSLKINRYKCGYCGACVGVCPKGALELVETWVEVDESTCITCGICGRICPVGAIEVMK
>2[4Fe-4S]ST9(637702249)Methanosarcina barkeri Fusaro, DSM 804
MSLKINRYKCGYCGACVGVCPKGALELVETWVEVDESTCITCGICGRICPVGAIEVMK
>2[4Fe-4S]ST9(2630863250)Methanosarcina horonobensis HB-1
VSININRYKCGYCGACVGVCPKGALELVETWIEVDESVCIVCGICDRICPVGAIEVMK
>2[4Fe-4S]ST9(638176262)Methanosarcina acetivorans C2A
VSVNINRYKCGYCGACVGVCPKGALELVETWIEVDESTCIKCGICDRICPVGAIEVMK
>2[4Fe-4S]ST9(2641568266)Methanosarcina mazei WWM610
VSVNINRYKCGYCGACVGVCPKGALELVETWIEVDESICIVCGICDRICPVGAIEVMK

>2[4Fe-4S]ST9(2629269631)Methanosarcina mazei S-6
VSVNINRYKCGYCGACVGVCPKGALELVETWIEVDESICIVCGICDRICPVGAIEVMK
>2[4Fe-4S]ST9(2633686608)Methanosarcina mazei LYC
VSVNINRYKCGYCGACVGVCPKGALELVETWIEVDESICIVCGICDRICPVGAIEVMK
>2[4Fe-4S]ST9(638167059)Methanosarcina mazei Go1
VSVNINRYKCGYCGACVGVCPKGALELVETWIEVDESICIVCGICDRICPVGAIEVMK
>2[4Fe-4S]ST9(2651207081)Methanosarcina mazei SarPi

VSVNINRYKCGYCGACVGVCPKGALELVETWIEVDESICIVCGICDRICPVGAIEVMK
>2[4Fe-4S]ST9(2638500251)Methanosarcina mazei C16
VSVNINRYKCGYCGACVGVCPKGALELVETWIEVDESICIVCGICDRICPVGAIEVMK

>2[4Fe-4S]ST9(648194279)Methanolacinia petrolearia DSM 11571
MVAVVDADKCTGCETCVDVCPSEAINMEDGIAVVDADACVDCESCVDECPAEAIHME

>2[4Fe-4S]ST9(640116147)Methanoculleus marisnigri JR1, DSM 1498
MAAVVDIEKCTGCETCVDVCPASAITMEDGKAKVDVDLCVDCETCVDECPSEAISME

>2[4Fe-4S]ST9(2540642432)Methanoculleus bourgensis MS2
MTAVVDPELCTGCETCVDVCPAAAIRMEEGKAEVDPGLCVDCGTCVDECPSGAIHLE

>2[4Fe-4S]ST9(640868631)Methanoregula boonei 6A8
MVAVIDMTKCTGCETCVSECPASAISMENEKAKVDKDMCVDCQTCVDACPSEAIQME

>2[4Fe-4S]ST9(2509038734)Methanoregula formicica SMSP, DSM 22288
MVAIVDNDKCTGCETCVSECPASAIAMENDKAKVDKDMCVDCQTCVDVCPSEAIHME
>2[4Fe-4S]ST9(2730776139)Methanomicrobiales archaeon Methan_06
MTAIIDKDKCTGCETCVDECPATAITMENEKAVVNNELCVDCGSCVDVCPAEAITME

>2[4Fe-4S]ST9(637897125)Methanospirillum hungatei JF-1
MTAIIDKDKCTGCETCVDECPATAITMENEKAVVNNELCVDCGSCVDVCPAEAITME
>2[4Fe-4S]ST9(640116728)Methanoculleus marisnigri JR1, DSM 1498
MAAIIDPELCTGCETCVGACPAGAIRMEDVKPKIDTGLCIDCGTCVDECPTGAVRLE

>2[4Fe-4S]ST9(643569118)Methanosphaerula palustris E1-9c, DSM 19958
MVAKVDSDLCVGCETCVDECPAEAIAMANGIAVIDKDKCVDCGSCVEVCPSSAITMD

>2[4Fe-4S]ST9(2507146443)Methanofollis liminatans GKZPZ, DSM 4140
MVAVVDPDLCVGCETCVDECPSEAITMEDGIAVIDKDNCVDCGTCVDVCPSAAIHME
>2[4Fe-4S]ST9(640099186)Methanocorpusculum labreanum Z
MPAVVNKEKCTGCATCVDICPSAAIELVDDKAVISVDDCVDCETCVDECPESAIHME

>2[4Fe-4S]ST9(2730778952)Methanomicrobiales archaeon Methan_05
MPAVVNKEKCTGCATCVDICPSAAIELVDDKAVVNADECVDCESCVDECPESAIHME
>2[4Fe-4S]ST9(2507147516)Methanofollis liminatans GKZPZ, DSM 4140
MLTIHREICGYCGACVSVCPEGALELVDAYLTVDTGACIGCGLCTKACPLGALEVTDEE
>2[4Fe-4S]ST9(2730778409)Methanomicrobiales archaeon Methan_05
MAYVINTDECIACGACEAECPEGAISEVNGAYMIDPAKCSECGTCADVCPSGSPQKA

Bacteria

>2[4Fe-4S]ST9(2FDN)Clostridium acidurici
AYVINEACISCGACEPECPVNAISSGDDRYVIDADTCIDCGACAGVCPVDAPVQA
>2[4Fe-4S]ST9(1CLF)Clostridium pasteurianum
AYKIADSCVSCGACASECPVNAISQGDSIFVIDADTCIDCGNCANVCPVGAPVQE
>2[4Fe-4S]ST9(1FCA)Gottschalkia acidurici
AYVINEACISCGACEPECPVDAISQGGSRYVIDADTCIDCGACAGVCPVDAPVQA

Eukaryota

>2[4Fe-4S]ST9(AAA29098.1)Entamoeba histolytica
MGKITIVNIDDCVACGACSGTCPQSVLEVNDHVEIKNPDDCIGCGACVDACPQGVLKVE

Subtype 10

Archaea

>2[4Fe-4S]ST10(637896763)Methanospirillum hungatei JF-1
MIEVHREICAYCGCCVSVCPEGALELVDAYLDIDTNTCKNCGICVRVCPLGALEAKK
>2[4Fe-4S]ST10(2730776450)Methanomicrobiales archaeon Methan_06
MIEVHREICAYCGCCVSVCPEGALELVDAYLDIDTNTCKNCGICVRVCPLGALEAKK

Subtype 12

Eukaryota

>2[4Fe-4S]ST12(640421311)Ostreococcus lucimarinus CCE9901
MPTDLAGVLDASAKTLVLTELLRGMSLTLKYFFDKKVTINYPFEKGPLSPRFRGEHALRR
YPTGEERCIACKLCEAICPAQAITIEAEEREDGSRRTTRYDIDMTKCIFCGFCQEACPVD
AIVEGPNFEYATFSHEELLYDKEKLLSNGDRWEKEIARNLQSEAMYR
>2[4Fe-4S]ST12(649349337)Thalassiosira pseudonana CCMP 1335
MTEIWRAFWLSGEVALKPKVTINYPYEKGYLSPRFRGEHALRRYPSGDERCIACKLCEAA
CPAQAITIDAEEREDGARRTTRYDIDMTKCIYCGFCQEACPVDAIVEGPNFEFATETHEE
LLYDKEKLLSNGDKWEKQIAKNLLSEHLYR

Archaea

>2[4Fe-4S]ST12(640897719)Ignicoccus hospitalis KIN4/I, DSM 18386
MEERRFVEVRERWGRKTNALGVLKSLKAVAEYLVQSRPTTLYPFEKNDLPENFRGVLVYD
IEKCIGCGACVLACPNNCLYRRPGPKTEKNKPGIYIAFEPTHCLFCGLCVDACPPVASSL
RHSNVVSIVSTKKKIIWEPWEWAAFTKLIEEKGWENDAIDYDRVEHLVKERAEQIKKALE
EEAEKGAKK

Subtype 15

Archaea

>2[4Fe-4S]ST15(2540562111)Methanosarcina mazei Tuc01
MKIKICIPTERIHNPIISESIIETGILLNIMVANIDSTYGELIADVKDSRFDKIKNALES
RGAIVAILDRPIHRDEEECVECGACISVCPMNVYAFDEDWSLCVDEKKCIQCGMCIKMCP
HGALKLGVFSFLSFSFFISRLCPFFFFSLSFLLFPSLCLFFPFSFFSAFYPVHKIQIRVY
ECIPISISKLPLFID
>2[4Fe-4S]ST15(2509663232)Methanomethylovorans hollandica DSM 15978
MFIAVYKNKCTGCGLCVITCQNQVLTIEDGSCRAVRTEDCRYCMDCVASCDFDAIKVYV
>2[4Fe-4S]ST15(2574367197)Uncultured Crenarchaeota C2
MVKIVVDPKCTGCETCVNTCPVGVYEIKDGKSTPTKVSECLVCRACEAQCPEGAIQVIE
>2[4Fe-4S]ST15(650798190)Methanothrix soehngenii GP-6
MPAVVNRDECVSCGTCVEECPEEAISLDDEEIAVVNKEKCTECKTCVEACPSEAISME

>2[4Fe-4S]ST15(2730780243)Methanosarcinales archaeon Methan_04
MPAVVNRDECVSCGTCVEECPEEAISLDDEEIAVVNKEKCTECKTCVEACPSEAISME
>2[4Fe-4S]ST15(639700283)Methanothrix thermoacetophila PT
MPAVVNREECVSCGTCVEECPEEAIKLDDEEIAVVDPEKCTECGTCVEACPSEAIHIE
>2[4Fe-4S]ST15(2730785794)Methanosarcinales archaeon Methan_02
MPAVVNREECVSCGTCVEECPENAITMDDEEIAVVNVDKCTECGSCVEACPSEAISIE
>2[4Fe-4S]ST15(2512008612)Methanosaeta harundinacea 6Ac
MPAVVNREECVSCGTCVEECPENAITMDDEEIAVVNVDKCTECGSCVEACPSEAISIE
>2[4Fe-4S]ST15(2730786947)Methanosarcinales archaeon Methan_01
MPAVVNREECVSCGTCVEECPENAIALDDEEIAVVNADKCTECGTCVEACPSEAISIE
>2[4Fe-4S]ST15(648056844)Methanohalobium evestigatum Z-7303, DSM 3721
MVAVIISENCVGCATCVDECPVEAISLDGENIAVVDEGECSDCGECVDVCPTEAIEIE
>2[4Fe-4S]ST15(646706178)Methanohalophilus mahii SLP, DSM 5219
MVAVINVDECVGCGACVDECPSEAISMNDENIAVVDAEECVDCGVCVDVCPTEAITME
>2[4Fe-4S]ST15(2641586738)Methanococcoides methylutens MM1
MVAVINRDECVGCGVCVDDCPAEAISMDGDNIAVVDADACTECGICVDSCPSEAISME
>2[4Fe-4S]ST15(2519473217)Methanolobus psychrophilus R15
MVAIIIVDECVGCGTCVDECPSEAISMNGDNIAVVVADECLDCGACVDVCPTDAIKME
>2[4Fe-4S]ST15(637959326)Methanococcoides burtonii DSM 6242
MVAVINRDECVGCGTCVDDCPSEAISMDGENIAVVNADECIDCGACVDSCPTDAISME
>2[4Fe-4S]ST15(2641587503)Methanococcoides methylutens MM1
MVAIINRDECVGCGTCVDDCPSEAISMDGDNIAVVDNDECLDCGACVDSCPTDAITME
>2[4Fe-4S]ST15(2502871991)Methanosalsum zhilinae WeN5, DSM 4017
MVAIVDPDECLGCGPCVDACPVDAITINDDDLAIIDPDLCTDCGECVDACPTEAISME
>2[4Fe-4S]ST15(2502871345)Methanosalsum zhilinae WeN5, DSM 4017
MVAIIDPDKCNGYTLCADACPVDAITIREDDIAIVDPDICTDCGECVDACPVGAITIK
>2[4Fe-4S]ST15(648055440)Methanohalobium evestigatum Z-7303, DSM 3721
MPAVVDSEICTGCEACVDECPVDAISMNDDGIAVVDEEECTDCEACVDICPVEAISMK
>2[4Fe-4S]ST15(2519472560)Methanolobus psychrophilus R15
MPAIVDKDLCTGCGACVDSCPVEAISMNDKDLAVVDPDTCVDCGDCVDTCPVDAISMS
>2[4Fe-4S]ST15((2641566495)Methanosarcina mazei WWM610
MVAKIDADACTGCGTCVDECPAAAISLNDDDIAVVDEDECLDCGACVDACPNGAITLE
>2[4Fe-4S]ST15((2629267826)Methanosarcina mazei S-6
MVAKIDADACTGCGTCVDECPAAAISLNDDDIAVVDEDECLDCGACVDACPNGAITLE
>2[4Fe-4S]ST15((2633684037)Methanosarcina mazei LYC
MVAKIDADACTGCGTCVDECPAAAISLNDDDIAVVDEDECLDCGACVDACPNGAITLE
>2[4Fe-4S]ST15((638165255)Methanosarcina mazei Go1
MVAKIDADACTGCGTCVDECPAAAISLNDDDIAVVDEDECLDCGACVDACPNGAITLE
>2[4Fe-4S]ST15((2540562642)Methanosarcina mazei Tuc01

MVAKIDADACTGCGTCVDECPAAAISLNDDDIAVVDEDECLDCGACVDACPNGAITLE
>2[4Fe-4S]ST15((2651205358)Methanosarcina mazei SarPi
MVAKIDADACTGCGTCVDECPAAAISLNDDDIAVVDEDECLDCGACVDACPNGAITLE
>2[4Fe-4S]ST15((2638498430)Methanosarcina mazei C16
MVAKIDADACTGCGTCVDECPAAAISLNDDDIAVVDEDECLDCGACVDACPNGAITLE
>2[4Fe-4S]ST15((2633313730)Methanosarcina lacustris Z-7289
MVAKIDADACTGCGSCIDECPAAAISLSDDDFAVVDEAECLDCGACEDVCPNGAITLE

>2[4Fe-4S]ST15((2628412522)Methanosarcina sp. WH1
MVAKIDADACTGCGSCIDECPAAAISLSDDDIAVVDEDECLDCGACEDTCPNGAITIE
>2[4Fe-4S]ST15((2629258225)Methanosarcina sp. WWM596
MVAKIDADACTGCGSCIDECPAAAISLSDDDIAVVDEDECLDCGACEDTCPNGAITIE

>2[4Fe-4S]ST15((2630860837)Methanosarcina horonobensis HB-1
MVAKIDADACTGCGSCIDECPAAAITLSDDDIAVVDEDECLDCGACEDACPNGAITLE
>2[4Fe-4S]ST15((638179083)Methanosarcina acetivorans C2A
MVAKIDADACTGCGSCIDECPAAAISLSDDDIAVVDEDECLDCGACEDACPNGAITLE
>2[4Fe-4S]ST15((2645886312)Methanosarcina siciliae HI350
MVAKIDADACTGCGSCIDECPAAAISLSDDEIAVVDEDECLDCGACEDACPNGAITLE
>2[4Fe-4S]ST15((2653164945)Methanosarcina siciliae T4/M
MVAKIDADACTGCGSCIDECPAAAISLSDDEIAVVDEDECLDCGACEDACPNGAITLE
>2[4Fe-4S]ST15((2628827425)Methanosarcina siciliae C2J
MVAKIDADACTGCGSCIDECPAAAISLSDDEIAVVDEDECLDCGACEDACPNGAITLE
>2[4Fe-4S]ST15((2630385164)Methanosarcina thermophila CHTI-55
MVAKVNVDLCTGCGSCVDECPAAAISLNDDGIATVDESECLDCGSCEDACPNNAITIE

>2[4Fe-4S]ST15((2637280752)Methanosarcina thermophila TM-1
MVAKVNVDLCTGCGSCVDECPAAAISLNDDGIATVDESECLDCGSCEDACPNNAITIE

>2[4Fe-4S]ST15((2683956857)Methanosarcina sp. 795 OTU795
MVAKVNVDLCTGCGSCVDECPAAAISLNDDGIATVDESECLDCGSCEDACPNNAITIE
>2[4Fe-4S]ST15((2649850193)Methanosarcina barkeri 3
MVAKVNAEVCTGCGSCVDECPAAAISLSDDDIAVVDESECLDCGACEDACPNNAITIE
>2[4Fe-4S]ST15((2632831652)Methanosarcina barkeri 227
MVAKVNADICTGCGTCVDECPAGAISLNDDDIAVVDESECLDCGACEDACPNNAITIE
>2[4Fe-4S]ST15((2635680791)Methanosarcina barkeri CM1
MVAKVNADICTGCGTCVDECPAGAISLNDDDIAVVDESECLDCGACEDACPNNAITIE
>2[4Fe-4S]ST15((2633011800)Methanosarcina barkeri MS
MVAKVNADICTGCGTCVDECPAGAISLNDDDIAVVDESECLDCGACEDACPNNAITIE

>2[4Fe-4S]ST15((2628282522)Methanosarcina sp. Kolksee
MVAKVNADSCTGCGTCVDECPAAAISLSDDDIAVVDENECLDCGACEDACPNNAITIE
>2[4Fe-4S]ST15((2648886797)Methanosarcina vacuolata Z-761
MVAKVNADSCTGCGTCVDECPAAAISLSDDDIAVVDENECLDCGACEDACPNNAITIE
>2[4Fe-4S]ST15((2632754257)Methanosarcina barkeri Wiesmoor
MVAKVNADSCTGCGTCVDECPAAAISLNDNDIAVVDENECLDCGACEDACPNNAITIE
>2[4Fe-4S]ST15((637699021)Methanosarcina barkeri Fusaro, DSM 804
MVAKVNADSCTGCGTCVDECPAAAISLNDNDIAVVDENECLDCGACEDACPNNAITIE
>2[4Fe-4S]ST15((2522260427)Archaeoglobus sulfaticallidus PM70-1, DSM 19444 (Asulf_version1)
MIAVLNCQGCGTCDRVCKTGAIIRNGAKVARIDHDKCLECYDCVEACPYGALIVMD

>2[4Fe-4S]ST15((638158725)Archaeoglobus fulgidus VC-16, DSM 4304
MIAVLGCQGCGTCSRVCPTDAIIREGGKVVRIDQSKCKECYECVKACPYGALIVMD

>2[4Fe-4S]ST15((2635974282)Geoglobus acetivorans SBH6
MPPAVLGCQGCGRCSQVCPTDALIRQGGKVVRIDADRCMECYKCVEVCPYGALIKMD

>2[4Fe-4S]ST15((646611290)Ferroglobus placidus AEDII12DO, DSM 10642
MSIAVLSCQGCGKCSQVCPTDAIVREGGKVVRIDESKCLKCYKCVEVCPYGALIKMD
>2[4Fe-4S]ST15(2635678339)Methanosarcina barkeri CM1
MVAKINADACTSCGSCIGECPACAISLNDNDVSFVVEDKCNDCGVCLYVCVHGAITIV

>2[4Fe-4S]ST15(2632829309)Methanosarcina barkeri 227
MVAKINADACTSCGSCIGECPACAISLNDNDVSFVVEDKCNDCGVCLYVCVHGAITIV
>2[4Fe-4S]ST15(2649848043)Methanosarcina barkeri 3
MVAKIDANSCESCGNCIGECPACAISINDDDVSFVIEDKCNDCGICLNACVHGAITIV

>2[4Fe-4S]ST15(2633010610)Methanosarcina barkeri MS
MVAKINADACTSCGSCIGECPACAISLNDNDVSFVVEDKCNDCGVCLYVCVHGAITIV
>2[4Fe-4S]ST15(2628282755)Methanosarcina sp. Kolksee
MVAKINPESCASCGSCIGECPACAISLNDDDVSFVIEDKCNDCGVCLDVCVHGAITII
>2[4Fe-4S]ST15(2648887041)Methanosarcina vacuolata Z-761
MVAKINPESCASCGSCIGECPACAISLNDDDVSFVIEDKCNDCGVCLDVCVHGAITII
>2[4Fe-4S]ST15(2632755851)Methanosarcina barkeri Wiesmoor
MVAKINPESCASCGSCIGECPVCAISINDNDVSFVLEDKCNDCGVCLYVCVHGAITIV
>2[4Fe-4S]ST15(637701322)Methanosarcina barkeri Fusaro, DSM 804
MVAKINPESCASCGSCIGECPVCAISINDNDVSFVLEDKCNDCGVCLYVCVHGAITIV

>2[4Fe-4S]ST15(638157550)Archaeoglobus fulgidus VC-16, DSM 4304

MAIIVLETCNGCKVCEEVCPFHVFEVIDGFAVAVNSDECIECCACVEACPESAIIVSGCG

Subtype 17

Eukaryota

>2[4Fe-4S]ST17(639591493)Arabidopsis thaliana Columbia
MLPMITGFMNYGQQTLRAARYIGQGFMITLSHTNRLPVTIQYPYEKLITSERFRGRIHFE
FDKCIACEVCVRVCPIDLPVVDWKLETNIRKKRLLNYSIDFGICIFCGNCVEYCPTNCLS
MTEEYEFSTYDRHELNYNQIALGRLPMSVIDDYTIRTIWNSPQTKNGVNPLI
>2[4Fe-4S]ST17(2507872390)Zea mays mays cv. B73
MFPMLTGFITYGQQTIRAARYIGQSFIITLSHTNRLPITIHYPYEKSITLERFLGRIHFE
FDKCIACEVCVRVCPIDLPFVDWRFEKDIKRKQLLNYSIDFGVCIFCGNCVEYCPTSCLS
TTEEYELSTYDRHELNYNQIALSRLP

Subtype 18

Bacteria

>2[4Fe-4S]ST18(1BWE)Bacillus schlegelii(mutantD13C)
AYVITEPCIGTKCASCVEVCPVDCIHEGEDQYYIDPDVCIDCGACEAVCPVSAIYHEDFVPEEWKSYIQKNRDFFKK

Archaea

>2[4Fe-4S]ST18(2574367207)Uncultured Crenarchaeota C2
MPEKNASAVDIFGLLSEDSPEQAKKKRRQELLEPTGVKELFKGGNISVNKYTCDGVQCKL
CVKACPTNALYWKAGEVGIIEDLCVYCGACVLNCMVDDCIKIERKREDGKVERFSKPQDV
IMLEEKVNGKKRFDRVKAIFPTADAYCEKYTVKPEDINLFLGKTAGNRNTDKQKHYFTKP
RKRRRQPKHE

Subtype 19

Bacteria

>2[4Fe-4S]ST19(ALO12934.1)Streptomyces venezuelae
MPLVPQRADVPVTIDESKCIDGCTLCVEMCPLDSLAIREDNGKAYMHVDECWYCGPCAARCPTGAVTVNM
PYLLR
>2[4Fe-4S]ST19(A9GAV0)Sorangium cellulosum So ce56
MGHLLNRESVKAQSARSVGHAEAAVRLDLPVVIDEASCIKGCRICIDSCPVDCLAIDPETKKARMAYDECWYCLACEIDCPKEAITVKIPFLIR

Subtype 20

Bacteria

>2[4Fe-4S]ST20(1DUR)Peptoniphilus asaccharolyticus

AYVINDSCIACGACKPECPVNCIQEGSIYAIDADSCIDCGSCASVCPVGAPNPED

Archaea

>2[4Fe-4S]ST20(637846037)Methanosphaera stadtmanae DSM 3091
MAGNPYKINDNCVACGLCVNACPIDAIAEGNPYVIDEEKCVGCGVCAEACPTQAIEEVA

>2[4Fe-4S]ST20(2645883269)Methanosarcina siciliae HI350
MEDPLEFLRNIKSVAVATVDDGKPAVRMNDVMLVENEKLYFLTARGKPYYRQLKENPEIA
LVGMDKNYVMVRVRGRIEFVENIFLEKIFEANPILDEIYPGDTKHILEVFCLSSGVGEMY
DLSGIPPKRERFAFGGAKVAESGYKITEKCTACGICKDLCPSGAISKGKIYKIDGSICLE
CGRCAENCPYDAIEPPSGI
>2[4Fe-4S]ST20(2653161894)Methanosarcina siciliae T4/M
MEDPLEFLRNIKSVAVATVDDGKPAVRMNDVMLVENEKLYFLTARGKPYYRQLKENPEIA
LVGMDKNYVMVRVRGRIEFVENIFLEKIFEANPILDEIYPGDTKHILEVFCLSSGVGEMY
DLSGIPPKRERFAFGGAKVAESGYKITEKCTACGICKDLCPSGAISKGKIYKIDGSICLE
CGRCAENCPYDAIEPPSGI
>2[4Fe-4S]ST20(2641586371)Methanococcoides methylutens MM1
MEDPLEFLRNIKSVSVATVNNGTPAVRIADVMLHEDDKLYFLTARGKPYYKQLKENPEIA
IVAMDENYVTVRVKGKIEFVDRALLERIFEANPVMNHIYPGDTREILDVFCLPKGIGEVF
DLSIVPPKRERFAFGGAEVVESGYRINENCIACGICKGSCPTGAISEGNIYSIDSSICLE
CGNCYEKCPNDAIDLPAEF

>2[4Fe-4S]ST20(2628412320)Methanosarcina sp. WH1
MEDPLEFLRNIKSVAVATVDEGKPAVRMSDVMLVENEKLYFLTARGKPYYKQLKKNPEIA
LVGMDNNYVMVRVRGKIEFVENSFLEKIFEINPVLDEIYPEDTKDILEVFCLFSGVGEMF
DLSGTPPRRERFSFGGANVAEPGYKITEKCTACGICEELCPSGAISKGEIYKIDGSICLE
CGRCAEYCPYDAIEPPSSI
>2[4Fe-4S]ST20(2628823930)Methanosarcina siciliae C2J
MEDPLEFLRNIKSVAVATVDDGKPAVRMNDVMLVENEKLYFLTARGKPYYRQLKENPEIA
LVGMDKNYVMVRVRGRIEFVENIFLEKIFEANPILDEIYPGDTKHILEVFCLSSGVGEMY
DLSGIPPKRERFAFGGAKVAEPGYKITEKCTACGICKDLCPSGAISKGKIYKIDGSICLE
CGRCAENCPYDAIEPPSGI
>2[4Fe-4S]ST20(2629258468)Methanosarcina sp. WWM596
MEDPLEFLRNIKSVAVATVDEGKPAVRMSDVMLVENEKLYFLTARGKPYYKQLKKNPEIA
LVGMDNNYVMVRVRGKIEFVENSFLEKIFEINPVLDEIYPEDTKDILEVFCLFSGVGEMF
DLSGTPPRRERFSFGGANVAEPGYKITEKCTACGICEELCPSGAISKGEIYKIDGSICLE
CGRCAEYCPYDAIEPPSSI
>2[4Fe-4S]ST20(2633311162)Methanosarcina lacustris Z-7289
MEDPLELLRNIKSVAFATIEGGKPAVRMSDVMLYENGKLYFLTARGKPYYKQLKKNPEIA
IVGMNKNYVMVRVRGKIEFVDNSFLEKIFEINPVLDDIYPKDTKDILEVFCISAGVGEIF
DLSVAPPKRERFAFGGATVEEPGYKITEKCTACGICEDLCPTRAISKGKIYKIDGSICLE
CGRCYENCPYDAIEPASGL
>2[4Fe-4S]ST20(2682306712)Methanobrevibacter olleyae YLM1
MPVFAGRIPETARERLSKLKGNNTKAIAVVNYGNAQVADALVELVDILQENNFDVIAAAS
TISHHSIFDGVAIGRPDDEDIAKINEFAQNAIEKIENGGSLESSIPGNRPYIDYKQLPFI
VSCDVNKCVFCLECVSVCPEKAIPDDDPADVDLDLCSRCTSCINICPENARAFTGEAFKA
KKPAFESANAERKEPEFYF
>2[4Fe-4S]ST20(2758463438)Methanopyrus sp. SNP6
MWLAITGGKGGVGKTTVAAHVHRILGWDALDLDVTTPNLHLYLDCEEIDRSDVYIPCPKL
EDEDECDLCGTCARACAPGALVVGRSWDLDPHLCHGCGLCVESCPNGALAYDCVRIGEVR
RYQVSVTGAILTSGTLDVGDRRSRHLVRTLLEGADDGKDLILDTPAGAGKDVYDALKAAD
AAIAVTQPTPAAAT
>2[4Fe-4S]ST20(638169710)Methanopyrus kandleri AV19
LKVMVERCLGCAACAAVCPKDALTISGGKPEFGPECDDCGICAKVCPTGAIENEGE

>2[4Fe-4S]ST20(640786286)Methanococcus vannielii SB
MYNIVVTMKILECCVGCGTCVPFCPNGAITSIGNAEIECEKCTKCGLCVSYCPLNAIKN
>2[4Fe-4S]ST20(650856884)Methanotorris igneus Kol5, DSM 5666
MRILDRCVGCGECVPFCPFEAIKTYGKAIIDKEKCTNCGICIKYCPISAIEEDN

>2[4Fe-4S]ST20(638201948)Methanocaldococcus jannaschii DSM 2661

MGIKILEKCVGCGNCVVFCPRRAIKTYGVAIVDENKCSNCGICARYCPINAIKVDTSL
>2[4Fe-4S]ST20(646623544)Methanocaldococcus sp. FS406-22
MGIKILEKCVGCGNCVVFCPKKAIKTYGIAIVDKNRCSNCGICVRYCPINAIKIEV

>2[4Fe-4S]ST20(2504145967)Archaeoglobus veneficus SNP6, DSM 11195
MIVTTSSCVGCAFCKLVCPEEAIEVFGRAEIDSQKCVECKKCVIYCPIEAIKVVEE

>2[4Fe-4S]ST20(637846037)Methanosphaera stadtmanae DSM 3091
MAGNPYKINDNCVACGLCVNACPIDAIAEGNPYVIDEEKCVGCGVCAEACPTQAIEEVA

>2[4Fe-4S]ST20(2566176904)Thermoplasmatales archaeon BRNA1
MPKVIEDKCVGCGNCADVCPNDAIEIKDVAVIDEAKCVECDACVDECPSGAIEPSD
>2[4Fe-4S]ST20(2637997696)Candidatus Methanoplasma termitum Mpt1
MPPKVNAAECVACGSCADVCPQDAITIDDIAVIDASKCVDCGACVDECPPGAITE

>2[4Fe-4S]ST20(2661857974)Methanogenic archaeon ISO4-H5
MPKIIAENCVACGACADACPQEAITIDDIAVIDESKCVDCGACIDECNSDAIEE

>2[4Fe-4S]ST20(2566176906)Thermoplasmatales archaeon BRNA1
MPKIIAENCVACGACADACPEGAITMEDVAVIDDSKCIDCGACVDECSSDAIEN

>2[4Fe-4S]ST20(2566176905)Thermoplasmatales archaeon BRNA1
MPKIIAENCVACGACADVCPEGAITIDDVAVIDDSKCVDCGACVDECNSDAIEN

>2[4Fe-4S]ST20(2540667538)Candidatus Methanomethylophilus alvus Mx1201
MPKVIADNCVACGACVDACPNGAITVDDIAVIDASKCTDCGACIDACPSEAIEN

>2[4Fe-4S]ST20(2661857975)Methanogenic archaeon ISO4-H5
MPKVIADNCVACGACADACPESAITVDDVAVIDESKCVDCGACVDECPSEAIEN

Subtype 21

Bacteria

>2[4Fe-4S]ST21(A9G317)Sorangium cellulosum So ce56
MAYVIAEPCVATCDTACVPVCPVDCIHGPLAADEISRIPEGERKTRLAGLQLYIDPESCICCGACENECPVGAIFDEDELPAEWQRYREINARFFDDRAKERAPEET

Subtype 22

Eukaryota

>2[4Fe-4S]ST22(639591453)Arabidopsis thaliana Columbia
MSHSVKIYDTCIGCTQCVRACPTDVLEMIPWDGCKAKQIASAPRTEDCVGCKRCESACPT
DFLSVRVYLWHETTRSMGLAY
>2[4Fe-4S]ST22(2505189083)Paulinella chromatophora
MSHSVKIYDTCIGCTQCVRACPLDVLEMVPWDGCKASQIASSPRTEDCVGCKRCETACPT
DFLSIRVYLGDETTRSMGLAY
>2[4Fe-4S]ST22(649363882)Thalassiosira pseudonana CCMP 1335
MSHTVKIYDTCIGCTQCVRACPTDVLEMVPWDGCKSGQIASSPRVEDCVGCKRCETACPT
DFLSVRVYLGAETTRSLGLAY
>2[4Fe-4S]ST22(2507813137)Zea mays mays cv. B73
MSHSVKIYDTCIGCTQCVRACPTDVLEMIPWDGCKAKQIASAPRTEDCVGCKRCESACPT
DFLSVRIYLGPETTRSMALSY
>2[4Fe-4S]ST22(2507833286)Zea mays mays cv. B73
MSHSVKIYDTCIGCTQCVRACPTDVLEMIHWDGCKAKQIASAPRTEDCVGCKRCESACPT
EFLSVSVYLGPETTRSMALS
>2[4Fe-4S]ST22(2507863669)Zea mays mays cv. B73
MSHSVKIYDTCIGCTQCVRACPTDVLEMIPWDGCKAKQIASAPRTEDCVGCKRCESACPT
DF

Subtype 23

Eukaryota

>2[4Fe-4S]ST23(2505188940)Paulinella chromatophora
MLGFLRQVGDYTRDTVDAARNIVQGLSVTFDHLKRRPITVQYPYEKLIPSPRYRGRIHFE
LDKCIACEVCVKVCPINLPVVDFVPGPTSKVGKELRNYSIDFGVCIFCANCVEYCPTQCL
SMTEEYELTSFDRHNLNYDNVALGRLPLSIRADSSVIPLRELAELPKGDLDPHTSG

Subtype 24

Archaea

>2[4Fe-4S]ST24(2730778604)Methanomicrobiales archaeon Methan_05
MTSAKAYKDAALAARLSDRYYRPKQDSDPSFTAEVEKIGGTEAHICFQCGTCTGSCPSAA
RSSYRIRNFMRRVNLGMRDVCLNDPDLWLCTTCYTCADRCPRDLIPTDVIMAMRNIAAKQ
GIVPKNFLATVNFIYNTGHGVPNNDANRAARVKLGLEAEPETTSKYPEYIPAIRKILEIY
GTKQLADRVLAEDQ
>2[4Fe-4S]ST24(648195480)Methanolacinia petrolearia DSM 11571
MAVEKNYGNPDLEKKLADRNYYTSDSHKDFSKRVEKISGTMSHMCFQCGTCTGSCPSAPR
SSYRIRLFMRRAVLGLEDEALTDPDLWLCTTCYSCSDRCPRDLFPTDVIMAMRNLAFERD
IVPRNFLQTVNLIYTSGHGVPNNDVNRAAREKLGLPRDPPTTHSYPEYLEGIRKIIDHYK
LKENADRILSEGGN
>2[4Fe-4S]ST24(648195474)Methanolacinia petrolearia DSM 11571
MAVEKNYGNPDLEKKLADRNYYTSDSHKDFSKRVEKISGTMSHMCFQCGTCTGSCPSAPR
SSYRIRLFMRRAVLGLEDEALTDPDLWLCTTCYSCSDRCPRDLFPTDVIMAMRNLAFERD
IVPRNFLQTVNLIYTSGHGVPNNDVNRAAREKLGLPRDPPTTHSYPEYLEGIRKIIDHYK
LKENADRILSEGGN
>2[4Fe-4S]ST24(640098885)Methanocorpusculum labreanum Z
MTSAKAYKDAALAARLSDRYYRPKQDSDPSFTAEVEKIGGTEAHICYQCGTCTGSCPSGA
RSTYRIRNFMRRVNLGMRDVCLNDPDLWLCTTCYTCSDRCPRNLIPTDVIMAMRNIAARQ
GIVPKNFLATVNFIYNTGHGVPNSDANRAARVKLGLEAEPETTCKYPEYIPAIRKILEAY
GTKQLADKVLAEGQ
>2[4Fe-4S]ST24(643570853)Methanosphaerula palustris E1-9c, DSM 19958
MAQSKGYPDAKLNEKLRDRYMRGSDTNPQFTQDIRDISKTTAHMCFQCGTCTGSCPSAPR
STYRIREFMRKGVLGLDDEALTDPDLWLCTTCYSCTDRCPRDIAPTDVIMAMRNLAFKRD
IIPINFLKTVLAIYKTGHGVPNNDVNRAAREKLGLTRDPPTTHMYPEYIKGIQKILDHYE
LKENADRIIAENEG
>2[4Fe-4S]ST24(640115307)Methanoculleus marisnigri JR1, DSM 1498
MAVKKDYKDQKLAEKLRDRKYYIPDSNPEFIKEVEKIGQTAAHMCYQCGTCTGSCPSAPR
SSYRIRLFMRKAILGLEEEILTDPDLWLCTTCYSCTDRCPRDLAPTDAIMAMRNLAARRD
IVPRNFLQTVQLIYKSGHGVPNNDANRAARKKLGLEEEPETTHKYPEYLPGIRKIIDHYG
LKDRADKILSEGE

>2[4Fe-4S]ST24(2509039562)Methanoregula formicica SMSP, DSM 22288
MARTKGYPEALEKKLRDQRYVREDSNPEFLKNVKSISRTIAHMCYQCGTCTGSCPSAPRS
SYRIRHFMRRAVLGLENEALTDPDLWLCTTCYSCSDRCPRDIIPTDVIMAMRNLAFKRDI
VPVNFLKTVQAIYSSGHGVPNNDVNRAAREKLGLTRDPPTTHMYPEYMPGIRKILDHYKL
KANADRIVKEREG
>2[4Fe-4S]ST24(2730773254)Methanomicrobiales archaeon Methan_07
MAVKKDYKDQKLAERLRDRKYYIPDSNPEFIKDVEKIGRTAAHMCYQCGTCTGSCPSAPR
SSYRIRLFMRKAILGLEEEALTDPDLWLCTTCYSCTDRCPRDLAPTDAIMAMRNLAFKRD
IVPRNFLKTAQLIYQSGHGVPNNAANRVAREKLGLEAEPETTHKYPEYLPGIRKIMDHYR
LKENADRILAEGE
>2[4Fe-4S]ST24(2540643946)Methanoculleus bourgensis MS2
MAVKKDYKDQKLAEKLQDRSYYVSDSNPEFIKEVERLGRTAAHMCYQCGTCTGSCPSAPR
SSYRIRLFMRKAILGLDEEVLTDPDLWLCTTCYSCTDRCPRDLAPTDAIMAMRNLAFKRD
IVPRNFLKTVQAIYKTGHGVPNNDTNRAAREKLGLEADPETTHKYPEYLPGIRKIMDHYA
MKENADRILAEGE
>2[4Fe-4S]ST24(2684091568)Methanoculleus sp. MAB1
MAVKKDYKDQKLAEKLQDRNYYASDSNPEFIKEVERLGRTAAHMCYQCGTCTGSCPSAPR
SSYRIRLFMRKAILGLDEEVLTDPDLWLCTTCYSCTDRCPRDLAPTDAIMAMRNLAFKRD
IVPRNFLKTVQAIYKTGHGVPNNDTNRAAREKLGLEADPETTHKYPEYLPGIRKIMDHYA
MKENADRILAEGE
>2[4Fe-4S]ST24(640867809)Methanoregula boonei 6A8
MVRTKGYPEALEKKLHDQRYYREDSTADFMKRVEASSRTIAHMCYQCGTCTGSCPSAPRS
TYRIRKFMRRAVLGFETEALTDPDLWLCTTCYSCTDRCPRDIAPTDVIMSMRNMAFTHDI
IPVNFLKTVTAIYASGHGVPNNDVNRAAREKLGLTRDPPTTHMYPEYMPAIRKILDHYHL
KENADRIIKEREG
>2[4Fe-4S]ST24(2688749754)Methanoculleus bourgensis MBBA
MAVKKDYKDQKLAEKLQDRNYYASDSNPEFIKEVERLGRTAAHMCYQCGTCTGSCPSAPR
SSYRIRLFMRKAILGLDEEVLTDPDLWLCTTCYSCTDRCPRDLAPTDAIMAMRNLAFKRD
IVPRNFLKTVQAIYKTGHGVPNNDTNRAAREKLGLEADPETTHKYPEYLPGIRKIMDHYA
MKENADRILAEGE
>2[4Fe-4S]ST24(637897455)Methanospirillum hungatei JF-1
MAAKSYNIPELDKKLADRRYHLSDTNPEFTQKILKTSRTIANMCYQCGTCTGSCPSAPRS
SYRIRLFMRRCVLGLENEALTDPDLWLCTTCYSCTDRCPRDIAPTDVIMAMRNLAFKRDI
VPKNFLQTVQLIYNSGHGVPNNDVNRAARTKLGLPADPPTTHSYPEFVKGIQKIIDHYEL
KENADRILKGD
>2[4Fe-4S]ST24(2507146036)Methanofollis liminatans GKZPZ, DSM 4140
MAKGYNDPAMEKKFQDRYYHAADNPEFTKEVERIGRTMAHMCYQCGTCTGSCPSAPRSSY
RIRKFVRRAVMGLEREALTDPDLWLCTTCYSCTDRCPRGIAPTDIIMAMRNLAFKWDIVP
RNFLKTIQLIYTTGHGVPNNDVNRAARVKLGLDAEPETTHKYPEFLPGIQKIMDHYKMKE

NADRILSEGEH
>2[4Fe-4S]ST24(2730775518)Methanomicrobiales archaeon Methan_06
MAAKSYNIPELDQKLADRRYHLSDTNPEFTQKIIKTSRTIANMCYQCGTCTGSCPSAPRS
SYRIRLFMRRCVLGLEDEALTDPDLWLCTTCYSCTDRCPRDIAPTDVIMAMRNLAFKRDI
VPKNFLQTCQLIYNSGHGVPNNDVNRAARTKLGLPADPPTTHSYPEFIKGIQKIMDHYEL
KENADRILKGD
>2[4Fe-4S]ST24(2730775515)Methanomicrobiales archaeon Methan_06
MAAKSYNIPELDKKLADRRYHLSDTNPEFTQNILKTSRTIANMCYQCGTCTGSCPSAPRS
SYRIRLFMRRCVLGLENEALTDPDLWLCTTCYSCTDRCPRDIAPTDVIMAMRNLAFKRDI
VPKNFLQTCQLIYNSGHGVPNNDVNRAARTKLGLPADPPTTHSYPEFIKGIQKIMDHYEL
KENADRILKGD
>2[4Fe-4S]ST24(650856113)Methanotorris igneus Kol5, DSM 5666
MLLKSEKDFDNNFVNEIIETGRTILGEGHVASFKACYQCGTCTGSCPSGRITAFKTRKLI
KYAQFGMRKFAVESEDLWMCTTCYECYERCPREVKITDIIKVIRNIAAREGRMAKAHRMT
ALYVFKTGHAVPINEETRKARKSIGLSEVPPTTHKYPEVLEVIRGIMKDMKFCDIVGICT
ESMELKPVEWKDMSE
>2[4Fe-4S]ST24(638202080)Methanocaldococcus jannaschii DSM 2661
MVIYAQKDISNDFIKEIIKTGEILGEGHVSSFKACYQCGTCTGSCPSGRITAFRTRKLIR
YAQFGMKSAIIDSEDLWMCTTCYECYERCPRTVKITDIIKVLRNIAAREGKMAEAHKKTA
LYVFKTGHAVPINDQIKKARKEIGLTEIPPTTHKYPDALEVVRGIMKDLRFCDMVGICTE
TMQLKPVEWKDMSE
>2[4Fe-4S]ST24(646366951)Methanocaldococcus vulcanius M7, DSM 12094
MVIYAQKDISNDFIEEIIKAGEILGEGHVSSFKACYQCGTCTGSCPSGRITAFRTRKLIR
YAQFGMKSAIIDSEDLWMCTTCYECYERCPRTVKITDIIKVLRNIAAREGKMAEAHKKTA
LYVFKTGHAVPINDQIKKARKEIGLTEIPPTTHKYPDALEVVRGIMKDLKFCDMVGICTE
TMQLKPMEWKDMSE
>2[4Fe-4S]ST24(638168586)Methanopyrus kandleri AV19
MVEPRDTVIREEDLNPDFLEELSELVEPVFEEEEVLSVQACYQCGTCTGSCPSGRRTSYR
TRLIMRKLQLGLVDEVIKSDELWMCTTCYTCYERCPRGVKIVDAVKAARNLAAKKGYMAK
AHRMVAMFVIKTGHAVPINDEIREVRKNIGLDEVPPTTHRYEEALEEVQKLVKINEFDKL
IGYDWEEGDLVD
>2[4Fe-4S]ST24(2758462870)Methanopyrus sp. SNP6
LVEPRDTVIREEDLNPDFLEELSELVEPVFEEEEVLSVQACYQCGTCTGSCPSGRRTSYR
TRLIMRKLQLGLVDEVIKSDELWMCTTCYTCYERCPRGVKIVDAVKAARNLAAKEGHMAK
AHRMVAMFVIKTGHAVPINDEIREVRKNIGLDEVPPTTHRYEEALEEVQKLVKINEFDKL
IGYDWEEGDLVD
>2[4Fe-4S]ST24(2758473009)Methanopyrus sp. KOL6
LVEPRDTVIREEDLNPDFLEELSELVEPVFEEEEVLSVQACYQCGTCTGSCPSGRRTSYR
TRLIMRKLQLGLVDEVIKSDELWMCTTCYTCYERCPRGVKIVDAVKAARNLAAKEGHMAK

AHRMVAMFVIKTGHAVPINDEIREVRKNIGLDEVPPTTHRYEEALEEVQKLVKINEFDKL
IGYDWEEGDLVD

Subtype 25

Archaea

>2[4Fe-4S]ST25(650916849)Methanothermococcus okinawensis IH1
MVLSSKDFNPKFPEKVIEAGKAIGEDEATKSFRRCYQCGTCTGSCPSGRITAFRTRKLIR
SAMLGLGDYIKSEDLWKCTTCYTCYERCPRNVKITDIIKAMRNVASQMGYMALAHRKTTL
YVYLTGHAVPVNDQIKEVRKNIGLTEIPPTTHKYPEALEDVRGIMNDMGLCDKVGICPTT
KKLEELKPIEWEDLSE
>2[4Fe-4S]ST25(646777310)Methanocaldococcus infernus ME
MVLTNKDFDPKFSEEIIKVAEEIGEEEVVKSFRRCYQCGTCTGGCPSGRHTAYRVRVLIR
SALMGFYKYIESEDLWMCTTCYTCYERCPRDVKITEIIKAMRNLAARLGLMAEAHRKTCL
YVYLTGHAVPINDKIKEVRKKLGLSEVPPTTYKYPEVLEHIRGIMQDMKLCEKVGICPTS
KTLKELTPVKWEEMSE
>2[4Fe-4S]ST25(640787700)Methanococcus aeolicus Nankai-3
MTIKSTDLNPKFTEKIIEAGEAIGEGDIISSFKRCYQCGTCTGGCPSGRITAYRVRQLIR
SAMFGLGDYVKTEDLWKCTTCYTCYERCPRDVKITEIIKAMRNVAAQMGYLATAHRKTCL
YVFLTGHAVPVNDEIKEVRKKIGLTEIPPTTHKYPEVVEEMRGIMEDLKLCEKVGICPTT
KKLQELTPMDWSDMSE
>2[4Fe-4S]ST25(640786137)Methanococcus vannielii SB
MVFKSSEFNPNFPKKIVDAGKEVFGANNASSFQKCYQCGTCTGACPSGRITALRTRKLIR
SALSGMDNLLKSDDLWMCTTCYECYEKCPREVKITDIIKTIRNIAAEEGYIAEPHRKTSL
LVFKTGHAVPVNDEIKKARLAIGLTEIPPTTHKYPEALEMVRDIMDDLKFCKKVGICRET
MDLEPLKVKKSKY
>2[4Fe-4S]ST25(646859226)Methanococcus voltae A3
MVLNSKDYKKEFSEAIVNAGIDFLGEDKSNSFKRCYQCGTCTGACPSGRLTGFRTRKVIR
QALLGSEELLSSDDLWMCTTCYECYEKCPRDVKITDLIKVIRNLASEKGYIADAHRKTSL
YVFKTGHAVPVNADIKKARLELGLTEIPPTTHKYPEALQMIKDIMSELEFCKKVGICEET
GELTELDLKYASE
>2[4Fe-4S]ST25(2563557097)Methanococcus maripaludis S2
MVLKSSEFNPDFPKQIIESGEWIFGDHASSFQKCYQCGTCTGACPSGRITALRTRKLIRS
ALAGIDSILSGDDLWMCTTCYECYEKCPREVKITDIIKIIRNIAAEKGYIAEPHRKTSLL

VFKTGHAVPVNDEIKKARLAIGLTEIPPTTHKYPEALEIVRDIMEDLNFCKKVGICRETM
DLEPLNVQKSEE
>2[4Fe-4S]ST25(641284018)Methanococcus maripaludis C6
MVLKSSEFNPDFPKQIIEAGEWIFGNNASSFQKCYQCGTCTGACPSGRITPLRTRKLIRS
ALGGIDSILSSDDLWMCTTCYECYEKCPREVKITDIIKIVRNIAAERGYIAEPHRKTSLL
VFKTGHAVPVNEEIKKARLAIGLTEIPPTTHKYPEALEMVRDIMDELNFCKKVGICRETM
DLEPLNVQRSGE
>2[4Fe-4S]ST25(640792923)Methanococcus maripaludis C7
MVLKSTEFNADFPKQIIEAGEWIFGKNASSFQKCYQCGTCTGACPSGRITPLRTRKLIRS
ALAGIDSILSGDDLWMCTTCYECYEKCPREVKITDIIKIIRNIAAEKGYIAEPHRKTSLL
VFKTGHAVPVNDEIKKARLAIGLTEIPPTTHKYPEALEIVRDIMDDLNFCKKVGICRETM
DLEPLNVQKSGE
>2[4Fe-4S]ST25(640165937)Methanococcus maripaludis C5
MVLKSSEFNPDFPKQIIEAGEWIFGKNASSFQKCYQCGTCTGACPSGRITPLRTRKLIRS
ALAGIDSILSSDDLWMCTTCYECYEKCPREVKITDIIKIVRNVAAEKGYIAEPHRKTSLL
VFKTGHAVPVNDEIKKARLAIGLTEIPPTTHKYPEALEMVRDIMDELNFCKKVGICRETM
DLEPLNVQKTGE
>2[4Fe-4S]ST25(2511672054)Methanococcus maripaludis X1
MVLKSSEFNPDFPKQIIESGEWIFGDHASSFQKCYQCGTCTGACPSGRITALRTRKLIRS
ALAGIDSILSGDDLWMCTTCYECYEKCPREVKITDIIKIIRNIAAEKGYIAEPHRKTSLL
VFKTGHAVPVNDEIKKARLAIGLTEIPPTTHKYPEALEIVRDIMEDLNFCKKVGICRETM
DLEPLNVQKSEE

>2[4Fe-4S]ST25(2655683303)Methanobrevibacter millerae SM9
MTSKQNIDDSPVDFAEKIIMDVKNSKDEGVLKCVQCGMCTSTCPAARHSDYNPRDIIERV
LAGDESILQDDLIWNCFYCYTCHSVCPVGNSVCEVNQILKQIAISRQIGYDKLYEYMGFA
DSYFTAAIGAIPEIFFDDIKNDVPGWWEFRTNLGEIREELELDPPLMPPKETIDEVSKIL
TITGFKEKIEKIRASQEAEK
>2[4Fe-4S]ST25(2682306669)Methanobrevibacter olleyae YLM1
MNKELINNNPIEFAVDIISHDKNTKENGVLKCVQCGMCTSTCPAARHSSYNPRDIIERVL
NGDEAILEEEDIWNCFYCYTCHSVCPVGNSVCEVNQILKQNAISKGIAYEKLYDYLGFAD
SYFNAAIGAIPEAFFEDIKDDVDGWWEFRQDLVNIRNKLNLGPLFPSEDVIDEVSIILTN
TGFKSRMDEIRKSQEDKI
>2[4Fe-4S]ST25(650751329)Methanobacterium lacus AL-21
MRTIKLNKNSHKLVDDVLKDLKASPDLGIYKCVQCGMCTSICPGASQSDYDPRDMVRRVL
EDDVSIVDDENIWNCFSCYTCNSVCPSGNNASEVNQILRQMSIDKGEGILKIQSFAAYGD
SFIELGVGSVPSKFFDDMVKDVGPEYMNLKLNIEDIRSDLGLGSYILPEKSIDEVEEILD
KSGFKARLKRIKGCKK
>2[4Fe-4S]ST25(2647504051)Methanobacterium formicicum DSM 1535

MRTLKLNEDSSKLAKEVIGDLKASPSLELFKCIQCGMCTSLCPGARYSDYNPREMVKRVL
DGDESVIYDDDIWNCFYCYTCNSVCPANNSASVVNQILRQKAINEGKQTDRLSAFLTYGD
SFLEIGIGSIPAAFFDVLVKDFGPEWLDLKMNLDDVRKELGLGSVTLPEDSVEEINEILK
ITGFTRKMEKIRGSP
>2[4Fe-4S]ST25(2685267469)Methanobacterium formicicum Mb91
MRTLKLNEDSSKLAKEVIGDLKASPSLELFKCIQCGMCTSLCPGARYSDYNPREMVKRVL
DGDESVIYDDDIWNCFYCYTCNSVCPANNSASVVNQILRQKAINEGKQTDRLSAFLTYGD
SFLEIGIGSIPAAFFDVLVKDFGPEWLDLKMNLDDVRKELGLGPVTLPEDSVEEINEILK
ITGFTRKMEKIRGSP
>2[4Fe-4S]ST25(650751372)Methanobacterium lacus AL-21
MRTIKLKKDPLKLLNDVLKHLKASPELGIFKCVQCGMCSSVCPAAKRTDYDPKEMLGRVL
QDDETVVDDDTIWYCFSCYTCNSVCPSGNNACEVNQILRQMAIDKGDVQRIESFTPYGDS
FMKKGIGTIPEKYYEDMVSDSGPECMNIKMDIQNIRSSLGLGNYILPDDAVNEIDLILKN
SGFMERIEMIKGCKK
>2[4Fe-4S]ST25(2631417857)Methanobacterium formicicum BRM9
MRTLKLNEDSSKLAKEVIGDLKASPSLELFKCIQCGMCTSLCPGARYSDYNPREMVKRVL
DGDESVIYDDDIWNCFYCYTCNSVCPANNSASVVNQILRQKAINEGKQTDRLSAFLTYGD
SFLEIGIGSIPAAFFDVLVKDFGPEWLDLKMNLDDVRKELGLGPVTLPEDSVEEINEILK
ITGFTRKMEKIRGSP
>2[4Fe-4S]ST25(2637992676)Methanobacterium sp. MB1
MRTLKLNEDSSKLAKEVVGDLKASPSLKLFKCIQCGMCTSLCPGARYSDYNPREMVKRVL
DGDESVIYDDDIWNCFYCYTCNSVCPANNSASVVNQILRQKAINEGKQTERLSAFLTYGD
SFLEIGIGSIPAAFFDVLVNDFGPKWLDLKMNLDDVRKELGLEPTTLPEDSIEEINKILK
ITGFTRKMEKIRGSP
>2[4Fe-4S]ST25(637846015)Methanosphaera stadtmanae DSM 3091
MGQSKKDDLAYKVLKDIKASPDLGLLRCVQCGMCASSCPAARHSEYDPRVVIKRVLDDDK
TLLEDKYIWNCFYCYNCHSVCPVGNSVCEVNQILRQMAIDKDTGKKEIESFMSFADSFLD
TGLGVIPKEYHKQLFKDYGPHWDRLQQQLEDVRKTLNLESMFLLPEAEKEVDKTLEAIGF
KNRVKKIKDVRDKK
>2[4Fe-4S]ST25(648117290)Acidilobus saccharovorans 345-15
MPARPSLKRVPRATFLGRAIAGNLGALIVGVKYFIDPNRITLLYPHEYIKLRQGYRGYIV
LIFDKCISCASCARICPARAMKMVTMEVKDKKLNRAMKKKYPVINYNRCIFCGYCVDVCP
TEALYHVPYHDLVYLNMQDMILNVEEFQKEPEFVTAKEGVPVTYVFDEKRGLVKVSVEES
SNQGSSSQPAGGGGSS
>2[4Fe-4S]ST25(2512784226)Methanocella conradii HZ254
MSIVFFGSMRVALRNVAEPSGDFRLEETCGYEKCMQCGRCTASCPAAFIYEDYRPRDVMR
MLQIGDREALMRVIWRCGQCYSCAARCPRNNSVGAGILALRESAMAAGLAPEGIMATAAM
IRKNLYGHGETFLPATFDFLEEFGPKTTQRCRDNASRRVRLGFDRDDARARPIPANSMKE
IRALIDMTWPGGEADA

>2[4Fe-4S]ST25(2505970216)Methanocella arvoryzae MRE50 (reannotation)
MATRIPLGISRSSNGCSTDPRLSGSGYEKCMQCGRCTASCPAAYTFDDYIPRSVMSRLAL
GMGDSLAEAVWRCGQCYSCRARCPRNNSVGEAVLALRERFGAEGRVPDTIKSVRSLLLNN
LHSRGETFLPQMLTDKLLREFGPATYSRCAGNIEKRARLGYEKEDARACRIPDDAMEEIR
HILTATGCSDTDDQK
>2[4Fe-4S]ST25(648117290)Acidilobus saccharovorans 345-15
MPARPSLKRVPRATFLGRAIAGNLGALIVGVKYFIDPNRITLLYPHEYIKLRQGYRGYIV
LIFDKCISCASCARICPARAMKMVTMEVKDKKLNRAMKKKYPVINYNRCIFCGYCVDVCP
TEALYHVPYHDLVYLNMQDMILNVEEFQKEPEFVTAKEGVPVTYVFDEKRGLVKVSVEES
SNQGSSSQPAGGGGSS

Subtype 26

Archaea

>2[4Fe-4S]ST26(650508321)Vulcanisaeta moutnovskia 768-28
MVIKIKEPRGTLAEDIVYHAKALITGFKEAIEPNRLTIQYPREVRWVPERFRGWIMLDIK
KCISCFQCAWACPVNAIQMYRAPNGKFYPGIRYTECILCHFCVDACPVGALIPTPIHDIA
YVDFDEVKFKPEDMSRSPEYIYDESERVIKYEIKNGRLLKIALPKDEIKRRLEELDKVIE
QSGGATAAAESSSA
>2[4Fe-4S]ST26(648201390)Vulcanisaeta distributa DSM 14429
MVIKIKPPKGTLAEDIIYHAKALITGFKEAVEPNRLTIQYPREVRWIPERFRGWIMLDIK
KCISCFQCAWACPVNAIQMYRAPNGKFYPGIRYTECILCHFCVDACPVGALIPTPIHDIA
YVDFDEVKFKPEDMSRPPEYVFDESERVIKYEIKDGRLLKIALPKDEIKKRIEELDKVIE
QSGGAPAAAESSSA

Subtype 27

Archaea

>2[4Fe-4S]ST27(646510256)Archaeoglobus profundus Av18, DSM 5631
MEMMEEGVPDVINLSYLAEREETELEKRVAEIIKELGAERLMYCMQCGACASICPLARVG
FEWYNKKLIKALILGLRDELLDDPTPWACVACNRCTEICPRRVSPFEVMFAMRRLMAEEY
AIGSLAIEGLRSLYEYGHAVYMAGREARKKVGLPEKPPSTESDPKALEDLRKILKQTKLA
ELGLVPME

Subtype 28

Archaea

>2[4Fe-4S]ST28(2730780308)Methanosarcinales archaeon Methan_04
MIVLIVSIGGIFYPKLGYILLLVFATLLIIAPFRGRWFCGNLCPRGSFVDFWLAPLSRRL
AIPLFLRSMWIRIPIFIALMGFMIYRLLGTQGVVDRIGMVFVTLCIITTSIAILFGVIIA
PRTWCTFCPMGTLQRIMGGSKYQLKLDGDLCIDCKRCQKVCPMQLRVFELQDLPDCIKCG
RCIDACPKDALIFH
>2[4Fe-4S]ST28(650798726)Methanothrix soehngenii GP-6
MIVLIVSIGGIFYPKLGYILLLVFATLLIIAPFRGRWFCGNLCPRGSFVDFWLAPLSRRL
AIPLFLRSMWIRIPIFIALMGFMIYRLLGTQGVVDRIGMVFVTLCIITTSIAILFGVIIA
PRTWCTFCPMGTLQRIMGGSKYQLKLDGDLCIDCKRCQKVCPMQLRVFELQDLPDCIKCG
RCVDACPKDALIFH

Subtype 29

Archaea

>2[4Fe-4S]ST29(2512783241)Methanocella conradii HZ254
MLEKLKASGELIANFFKRPVTVKESFGFVAEAFRWLPRRNDDLCTGCGACVERCSSGATS
ITDRGDTRVVSIDGLRCIFCGRCAEVCPEKALELTLEDAPPAQGSDPTARVSLSRGAEEP
RPTVDTVLKLQRCSVCGEVMPVTEKYLDAIKERTLRNLKPETAAIVEKDMERYLKTCINC
RRVHSLEWDTHPRKFI
>2[4Fe-4S]ST29(2505974063)Methanocella paludicola SANAE (reannotation)
MSIVGKLKAFGDLALNFFRKPVTVDESYGFTAENFRWLPRRNADLCTGCGACNERCSSGA
TSLTDADGERTVSIDGLRCIFCGRCADVCPERALELTLEPKKEGETDTGRVSLSHGSEEP
RPTVDTKLKLQKCRICGEYMPVTEKYVAVVRDRTLKNLKPETAAIVEKDMEKYLTVCVNC
RRKYSIEWDTHPRKFI

Subtype 30

Archaea

>2[4Fe-4S]ST30(2629270190)Methanosarcina mazei S-6
MDENFQAGKDGLMYAASKGLGIVIMEPMRGGYLVSGMPPEIQEIWDSADIQRSPVEWSLR
YLWDYPEIAVVLSGMSEIKHVEDNVKFASGGLPDSLTERERELISRVKKIYMSKTRVNCT
GCRYCMPCPSGVNIPENFKYLNNAEMFDNAEGEKALYSGLEGQASNCTECGQCEEKCPQK
IPVSQMLKEVVKLFGK

Subtype 31

Archaea

>2[4Fe-4S]ST31(641283839)Methanococcus maripaludis C6
MSVVIDYDKCKGPECAECVNTCPVEVFEIQGDKVVVARESDCTFCLVCVDVCPTGAITVK
ED
>2[4Fe-4S]ST31(640165763)Methanococcus maripaludis C5
MSVVIDYDKCKGPECAECVNTCPVEVFEIQGDKVVVARESDCTFCLVCVDVCPTGAITVK
ED

>2[4Fe-4S]ST31(640793100)Methanococcus maripaludis C7
MSVVIDYDKCKGPECAECVNTCPVEVFEIQGNKVVVARESDCTLCMVCVDVCPTNAITVK
ED

>2[4Fe-4S]ST31(2511672226)Methanococcus maripaludis X1
MSVVIDYDKCKGPECAECVNTCPVEVFEIQDDKVVIAKESDCTLCMVCVDVCPTDAITVK
ED

>2[4Fe-4S]ST31(650917829)Methanothermococcus okinawensis IH1
MSVTIDYNKCNGPECAECVNACPMEVFEIQGDKVVVVREEDCTFCLVCEDVCPTGAVKVK
ED
>2[4Fe-4S]ST31(2563557278)Methanococcus maripaludis S2
MSVVIDYDKCKGPECAECVNTCPVEVFEIQDDKVVVAKESDCTLCMVCVDVCPTDAITVK
ED

>2[4Fe-4S]ST31(640786308)Methanococcus vannielii SB
MSVIIDYNKCKGPECAECVNTCPTEVFEIKENKVIVMRESDCTFCLVCIDVCPTDAIIVK
ED

>2[4Fe-4S]ST31(640788487)Methanococcus aeolicus Nankai-3
MSVIINYNKCDGVDCAECMNACPMEVFTIEDNKVVVSKEDECTFCMICEDVCPNGAVKVK
D

>2[4Fe-4S]ST31(640788851)Methanococcus aeolicus Nankai-3
MVVEIDYNNCDGIDCSGCKDACPTDVFGVEGSKIVVSNADECTFCMMCEDLCPADAVKVK
N

>2[4Fe-4S]ST31(646368154)Methanocaldococcus vulcanius M7, DSM 12094
MAVTIDYNLCKGAECAECANNCPMEVFEIDGDKVVVARPDDCTYCGVCEDVCPTGAVKVE
PE

>2[4Fe-4S]ST31(646622590)Methanocaldococcus sp. FS406-22
MAVTIDYNLCKGAECAECVNNCPMEVFEIEGDRVVVAREEDCTYCGVCEDVCPTGAVKVE
PE

>2[4Fe-4S]ST31(646777180)Methanocaldococcus infernus ME
MAVTIDYSKCKGPDCAECVNNCPMEVFEIEGDKVVVANPDECTYCGVCEDVCPTSAVKVE
PE

>2[4Fe-4S]ST31(650856556)Methanotorris igneus Kol5, DSM 5666
MAVTIDYSKCKGPECAECVNTCPMEVFEIQGDKVVVAKEDECTFCGVCEDVCPTKAIKVT
QE

>2[4Fe-4S]ST31(650855791)Methanotorris igneus Kol5, DSM 5666
MKVTINYDLCKGPECAECVNACPMEVFEIQGDKVVVAREEDCSGCGVCMDVCPTNAVKVE
D

>2[4Fe-4S]ST31(646622552)Methanocaldococcus sp. FS406-22
MAVKINYDLCKGAECAECVDACPMEVFEVDGDKIIVAKEEDCTYCGVCVDSCPNNAIILE
E

>2[4Fe-4S]ST31(637846751)Methanosphaera stadtmanae DSM 3091
MIININYEDCRGIDCLDCLDICPMNVFDTVDDVLIIKKLDNCCGCQVCVDVCPNNAISIE
Y

>2[4Fe-4S]ST31(2647503509)Methanobacterium formicicum DSM 1535
MPNVIIDYEKCEGTECGECAEVCSMEVLAIDGDKITVKNQDGCSLCEICTDVCPNEAIKL
VD
>2[4Fe-4S]ST31(2685266675)Methanobacterium formicicum Mb91
MPNVIIDYEKCEGTECGECAEVCSMEVLAIDGDKITVKNQDGCSLCEICTDVCPNEAIKL
VD
>2[4Fe-4S]ST31(2631416651)Methanobacterium formicicum BRM9
MPNVIIDYEKCEGTECGECAEVCSMEVLAIDGDKITVKNQDGCSLCEICTDVCPNEAIKL
VD

>2[4Fe-4S]ST31(2637991649)Methanobacterium sp. MB1
MPNVIIDYEKCEGTECGECAEVCSMEVLAIDGDKITVKNHDGCSLCEICTDVCPNEAIKL
VD
>2[4Fe-4S]ST31(2631416840)Methanobacterium formicicum BRM9
MVKITVDQEKCEGADCAECVDVCPMEVLILDGEKVVVRNTEDCSLCEVCMDVCPNEAVKV
EDE
>2[4Fe-4S]ST31(2637992333)Methanobacterium sp. MB1
MVKITIDQEKCEGADCAECVDVCPMEVIILDGEKVKVRNTEDCSLCEVCMDVCPNEAINV
EDE
>2[4Fe-4S]ST31(650871787)Methanobacterium paludis SWAN-1
MVKITIDYDKCEGADCAECTDVCPMEILVLEGDKIVIKNKEDCSLCEVCMDVCPNEAVNV
EE
>2[4Fe-4S]ST31(2730764719)Methanobacterium congolense Buetzberg
MVKITIDYDKCDGADCAECVDVCPMEVLIIDGEKIVIQNKEECSLCEVCMDVCPNEAVNV
EE

Subtype 32

Archaea

>2[4Fe-4S]ST32(2758472322)Methanopyrus sp. KOL6
VPKVVIDYDACVGVSECGECIEACPMDVLDEEDDKPVVVNEDDCTGCGLCEQACPHGAIV
VEV

>2[4Fe-4S]ST32(2758462178)Methanopyrus sp. SNP6
VPKVVIDYDACVGVSECGECIEACPMDVLDEEDDKPVVVNEDDCTGCGLCEQACPHGAIV
VEV
>2[4Fe-4S]ST32(640788362)Methanococcus aeolicus Nankai-3

MVSVTINYDTCKGAKECGECEKNCPMEVFEVDGEKVVVAHEDECTGCGVCEDVCPTGAVK
VKF

Subtype 33

Archaea

>2[4Fe-4S]ST33(2512783147)Methanocella conradii HZ254
MRISQACVGCGHCKAICPVDAIETCGVSRITGNCIECGKCKGYCAIGAIEEDA
>2[4Fe-4S]ST33(2505974308)Methanocella paludicola SANAE (reannotation)
MRITQACVGCGHCKVFCPAGAIETCGVSRINDKCIECGKCKGYCALGAITEEP

>2[4Fe-4S]ST33(2509662603)Methanomethylovorans hollandica DSM 15978
MKIDERCVGCGQCTAFCPKNAIIVKGNAWITADCIQCKICIAYCPMKAIEESV
>2[4Fe-4S]ST33(2630384638)Methanosarcina thermophila CHTI-55
MKINDNCVGCGQCASFCMKEAIEVKGKARTTNACIDCGICAVYCPVKAIEVPA
>2[4Fe-4S]ST33(638176270)Methanosarcina acetivorans C2A
VDRMKINDNCVGCGQCASFCKKGAIEVRGRARATDACVECGLCVPYCPVKAIEVLV
>2[4Fe-4S]ST33(2641568259)Methanosarcina mazei WWM610
MKINDNCVGCGQCASFCKKGAIEVRGRARATDACVDCGMCIPYCPVKAIEVPA
>2[4Fe-4S]ST33(2629269638)Methanosarcina mazei S-6
MKINDNCVGCGQCASFCKKGAIEVRGRARATDACVDCGMCIPYCPVKAIEVPA
>2[4Fe-4S]ST33(2633686601)Methanosarcina mazei LYC
MKINDNCVGCGQCASFCKKGAIEVRGRARATDACVDCGMCIPYCPVKAIEVPA

>2[4Fe-4S]ST33(638167052)Methanosarcina mazei Go1
MKINDNCVGCGQCASFCKKGAIEVRGRARATDACVDCGMCIPYCPVKAIEVPA
>2[4Fe-4S]ST33(2645884218)Methanosarcina siciliae HI350
MKINDNCVGCGQCASFCKKGAIEVRGRARATDACVDCGLCVPYCPVKAIEVPA

>2[4Fe-4S]ST33(2653162866)Methanosarcina siciliae T4/M
MKINDNCVGCGQCASFCKKGAIEVRGRARATDACVDCGLCVPYCPVKAIEVPA
>2[4Fe-4S]ST33(2632829510)Methanosarcina barkeri 227
MKINDNCVGCGQCASFCKKGAIEVRGKARATDACIDCGICVLYCPVKAIEVVA
>2[4Fe-4S]ST33(2628283144)Methanosarcina sp. Kolksee
MKINDNCVGCGQCASFCKKGAIEVKGKAQATDACIDCGICALYCPVKAIEVLA
>2[4Fe-4S]ST33(646707657)Methanohalophilus mahii SLP, DSM 5219
MRVNEKCVGCGQCTAFCKQDAIVVKGNAYITEKCTNCGVCAFYCPLKAIEAEK
>2[4Fe-4S]ST33(637959063)Methanococcoides burtonii DSM 6242
MKINDNCVGCGQCTAFCKIDAIIVKSKAHITDACVNCGICATYCPMKAIEADE

>2[4Fe-4S]ST33(2641587135)Methanococcoides methylutens MM1
MKVNENCVGCGQCTAFCKKDAIIVKSKARITDKCVECGICAAYCPMKAIEVGE

Subtype 11

Archaea

>2[4Fe-4S]ST11(2730777729)Methanomicrobiales archaeon Methan_05
MIHIRRDVCGYCGACVSVCPRGALELIDAYLTVDETLCKNKCKICSTVCPLGAIELEEQ
>2[4Fe-4S]ST11(640099088)Methanocorpusculum labreanum Z
MIHIRRDVCGYCGACVSVCPRGALELIDAYLTVDETLCKNKCKICQTVCPLGAIEQEEQ

2[4Fe-4S]Alv

Subtype 1

Bacteria

>2[4Fe-4S]AlvST1(2FGO)Pseudomonas aeruginosa
SLKITDDCINCDVCEPECPNGAISQGEEIYVIDPNLCTECVGHYDEPQCQQVCPVDCIPLDDANVESKDQLMEKYRKITGKA

>2[4Fe-4S]AlvST1(1BLU)Allochromatium vinosum
ALMITDECINCDVCEPECPNGAISQGDETYVIEPSLCTECVGHYETSQCVEVCPVDCIIKDPSHEETEDELRAKYERITGEG
>2[4Fe-4S]AlvST1(3EXY)Allochromatium vinosum
ALMITDECINCDGCEPECPNGAISQGDETYVIEPSLCTECVGHYETSQCVEVCPVDCIIKDPSHEETEDELRAKYERITG
EG
>2[4Fe-4S]AlvST1(1RGV)Thauera aromatica K172
ALYINDDCTACDACVEECPNEAITPGDPIYVIDPTKCSECVGAFDEPQCRLVCPADCIPDNPDYRETREELQEKYDRLHG

>2[4Fe-4S]AlvST1(Q9I6D2_PSEAE)Pseudomonas aeruginosa (strain ATCC 15692)
MSLKITDDCINCDVCEPECPNGAISQGEEIYVIDPNLCTECVGHYDEPQCQQVCPVDCIPLDDANVESKDQLMEKYRKITGKA

Subtype 3

Bacteria

>2[4Fe-4S]AlvST3(P20624)Rhodobacter capsulatus
MPTVAYTRGGAEYTPVYLMKIDEQKCIGCGRCFKVCGRDVMSLHGLTEDGQVVAPGTDEWDEVEDEIVKKVMALTGAENCIGCGACARVCPSECQTHAALS

Subtype 9

Archaea

>2[4Fe-4S]AlvST9(650798943)Methanothrix soehngenii GP-6
MLKRKMIKNFTWPLKTASREIEVTRLYPEFMMELPDAERGIHELDATICIGCGSCARVCP
NSCIEMVHFKFGNPLKNKKMQFPQIDYGRCMFCGLCVDECPVECLKMGKKVIMAGWERKD
IVKGPDFLATKRFSAKEVADLEAEAKRIAAEKAAAKKAAAKDAAAAGDKKPAKEGANAEK
KKAVAKPAEGGAS
>2[4Fe-4S]AlvST9(2730779891)Methanosarcinales archaeon Methan_04
MLKRKMIKNFTWPLKTASREIEVTRLYPEFMMELPDAERGIHELDATICIGCGSCARVCP
NSCIEMVHFKFGNPLKNKKMQFPQIDYGRCMFCGLCVDECPVECLKMGKKVIMAGWERKD
IVKGPDFLATKRFSAKEVADLEAEAKRIAAEKAAAKKAAAKDAAAAGDKKPAKEGANAEK
KKAVAKPAEGGAS

Subtype 10

Archaea

>2[4Fe-4S]AlvST10(646859885)Methanococcus voltae A3
MVYAVNAEECIACGACVPVCAVEAISEMDDGKAVIDANKCNDCGDCADVCPVECIKQQ

>2[4Fe-4S]AlvST10(640786788)Methanococcus vannielii SB
MVFKVIEDECIACGACVPSCPVDAISEKDDGKAVIDASKCNSCGDCVDICPVDCIKEEKG
CC

>2[4Fe-4S]AlvST10(640793644)Methanococcus maripaludis C7
MAYKVNEDECIACGACVPSCPENAISEKADGKAVIDPAKCTGCGDCADICPVACIKEE

>2[4Fe-4S]AlvST10(641283324)Methanococcus maripaludis C6
MAYKVNEDDCIACGACVPSCPENAISEKADGKAVIDPAKCTSCGDCADICPVACIKEE
>2[4Fe-4S]AlvST10(640167082)Methanococcus maripaludis C5
MAYVVKEDECIACGACVPSCPENAISEKDDGKAVIDPAKCTGCGDCADICPVACIKEE
>2[4Fe-4S]AlvST10(2730767665)Methanothermobacter wolfeii SIV6
MAKIIIDYDECDACGECVDVCPMEVLIIVDGKLKVQHPEECNECEVCMDVCPNECIEVQE
D
>2[4Fe-4S]AlvST10(648151922)Methanothermobacter marburgensis Marburg DSM 2133
MAKIIIDYDECDACGECVDVCPMEVLIIVDGKLKVQHPEECNECEVCMDVCPNECIEVEE
D
>2[4Fe-4S]AlvST10(640591992)Methanobrevibacter smithii PS, ATCC 35061
MADIIIDNNECDNCGDCADVCPMEVLILEDEKLTVNEPDECSYCESCVDICPNECITIE
